# Photoredox-catalyzed cyclopropanation of allenes towards vinyl-cyclopropanes

**DOI:** 10.1039/d5sc05057j

**Published:** 2025-08-14

**Authors:** Hui Xie, Yan Zhang, Bernhard Breit

**Affiliations:** a Institut für Organische Chemie, Albert-Ludwigs-Universität Freiburg Albertstraße 21 79104 Freiburg im Breisgau Germany bernhard.breit@chemie.uni-freiburg.de

## Abstract

While the vinyl cyclopropane (VCP) scaffold exhibits unique reactivity in chemical transformations, its synthesis presents certain challenges. Herein, we report the visible-light photoredox-catalyzed radical-polar crossover cyclization (RPCC) of terminal and internal allenes with carboxylic acids, realizing the construction of functionalized vinyl cyclopropanes (VCPs) with highly chemo-, and regioselectivities under mild conditions. Moreover, this photoredox protocol exhibits good functional group tolerance, a broad substrate scope, facile scalability and easy rearrangement to give various cyclopentene units.

## Abstract 2

Visible-light photoredox-catalyzed regioselective radical-polar crossover cyclization (rRPCC) of terminal and internal allenes with carboxylic acids, access to functionalized vinyl cyclopropanes (VCPs).

## Graphical abstract

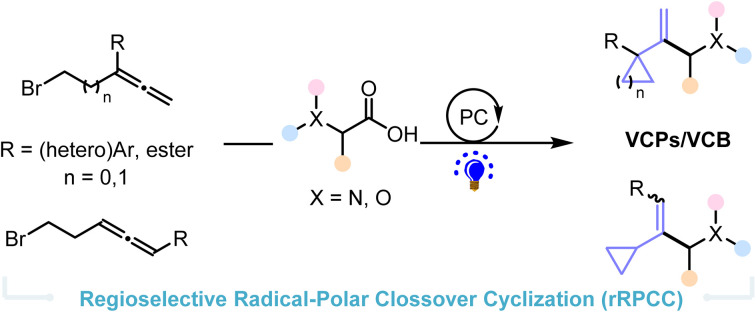

## Introduction

**Scheme 1:**
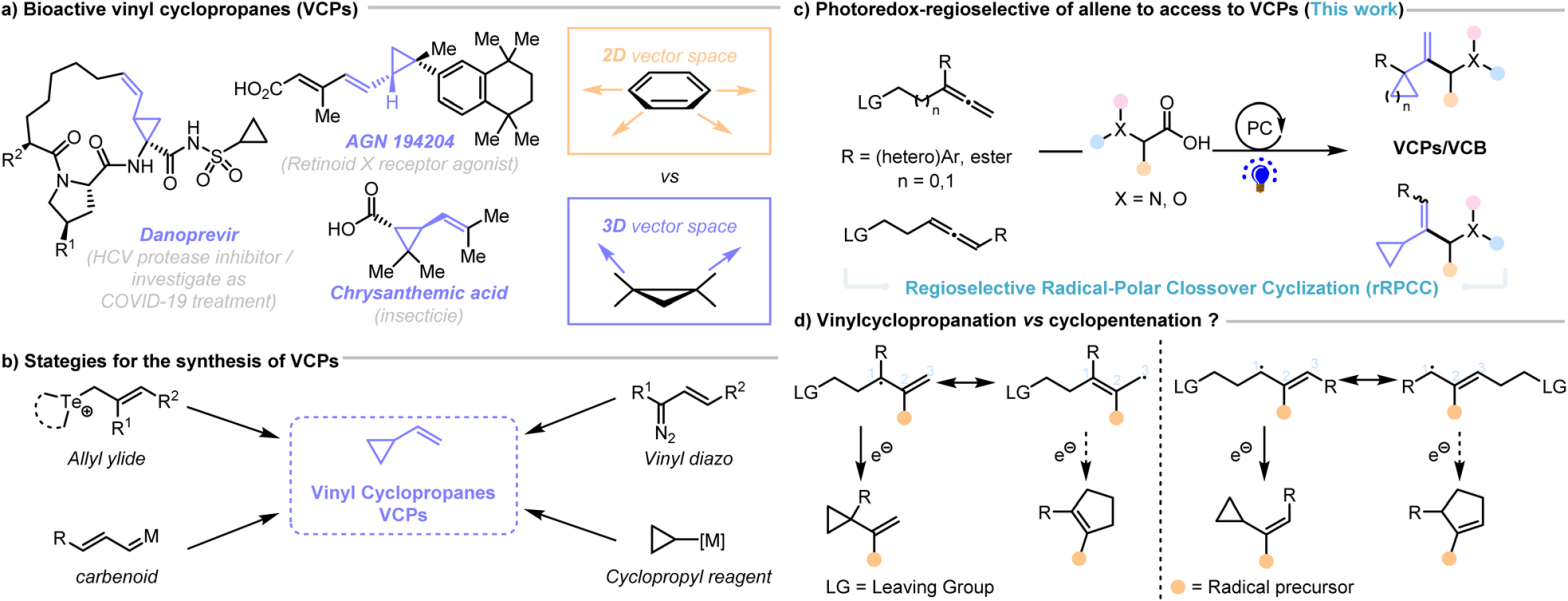
Strategies to construct vinyl cyclopropanes, a privileged motif in medicine.

Vinyl cyclopropanes (VCPs) serve as versatile synthetic precursors for cycloadditions or rearrangements^1–5^ and are also key structural motifs in natural products, bioactive compounds and pharmaceuticals,^6–8^ such as Danoprevir (against COVID-19),^9,10^ AGN 194204.^11,12^ Moreover, the inherent ring strain of cyclopropane yields strong core C–C and peripheral C–H bonds that preclude oxidative metabolism of a drug molecule and allow their use as bioisosteres for various alkyl, aryl, and vinyl substitution patterns^7,13^ (Scheme 1a). While the VCPs scaffold exhibits unique reactivity in organic synthesis,^1,2^ its synthesis presents certain challenges. The inherent reactivity of the VCP structure, particularly the ring strain and the vinyl double bond, require careful control of reaction conditions to prevent premature ring-opening or undesirable side reactions. Recent advancements in synthetic organic chemistry,^14–16^ including ylides,^17–19^ Zn-mediated carbenoid,^20–23^ metal-mediated cycloaddition reactions (vinyl diazo precursors),^24,25^ and allylic cyclization methodologies as well as selective functionalization strategies,^26–29^ have facilitated the efficient construction of VCP-containing compounds (Scheme 1b). Despite significant advance, more efficient and economical synthesis straight from feedstock materials (alkenes, allenes, dienes) still remains underrepresented, and the more challenging cyclization of allenes has not been realized up to date.

Recently, catalytic transformations involving photoinduced radical-polar crossover cyclization (RPCC) have become highly valuable and powerful processes for transforming simple and readily available alkene precursors into structurally complex cyclic molecular scaffolds.^30–33^ Molander pioneered an annulation process enabling the rapid and efficient formation of 1,1-disubstituted cyclopropanes though RPCC process.^34,35^ Meanwhile, Aggarwal described an interesting photoredox-catalyzed decarboxylative RPCC approach to functionalized cyclopropanes under mild conditions.^36^ Furthermore, Li, Yu, Fang, and others successfully accomplished the RPCC of alkenes for the synthesis of various functionalized cyclopropanes.^37–42^ Despite these advances, these methods are limited to electrophilic alkenes, and their development for the construction of synthetically important vinyl cyclopropanes (VCPs) is lagged behind. With our continuous efforts on the hydrofunctionalization of allenes,^43–46^ we reasoned that addition of α-amino/hydroxy radicals derived from carboxylic acids followed by addition to allenes through a regioselective RPCC (rRPCC) process could construct various functionalized vinyl cyclopropanes (VCPs) (Scheme 1c).

To the best of our knowledge, regioselective photocatalytic RPCC (rRPCC) with allenes had not been reported to date. Nevertheless, such a reaction faces several challenges (Scheme 1d).^47–49^ The versatile reactivities of allenes could results in different regioselectivities for the initial radical attack, which in addition could lead to a number of vinyl products. However, we hypothesized that the initial radical might preferentially occur at to the internal allene carbon atom to generate a stabilized allyl radical intermediate.^48^ From that electron transfer followed by either a 5-*exo-tet* or a 3-*exo-tet* anionic cyclization reaction could deliver either cyclopentenes or VCPs, respectively. We are delighted that despite these challenges we can report herein the successful implementation of a visible-light photoredox-catalyzed highly chemo- and regioselective cyclopropanation of internal and terminal allenes, enabling the construction of multi-substituted vinyl cyclopropanes (Scheme 1c).

## Results and discussion

We began our studies by investigating the decarboxylative rRPCC of allene 1a with commercial *N*-phenyl glycine 2a (Table 1). In the presence of 2 mol% 4-CzIPN, and 2.0 equiv. of K_2_HPO_4_, cyclopropanation of 1a in acetonitrile delivered 3 in 18% yield under blue light irradiation (entry 1). Subsequent screening of bases did not lead to an improvement (entries 2–3, Table S1). Recognizing the critical role of leaving groups in controlling cyclopropanation and catalytic efficiency, we conducted a thorough screening of various substituted allenes (entries 4–7, Table S2). Although allenyl chloride 1c and iodide 1d showed higher conversion, allenyl bromide 1e significantly improved the yield to 46% with a >20 : 1 regioselectivity. Next, further screening of various metal-based and organic photocatalysts with different redox properties was conducted (entries 8–9, Table S3). Pleasingly, the use of [Ir(ppy)_2_dtbbpy]PF_6_ (PC2) as photocatalyst efficiently promoted the targeted transformation to provide product 3 in good isolated yield (70%) with exclusive selectivity (entry 8). To further enhance reactivity, we explored varied solvents (Table S4) and found MeCN/DMSO (9 : 1) as the ideal choice, which improved the yield to 80% when 2.0 equiv. of 2a was used (entry 13). As anticipated, control experiments revealed that light, base, and photocatalyst were all essential components for this transformation (see Table S5 in the SI for details).

**Table 1:** Reaction condition optimizations^a^

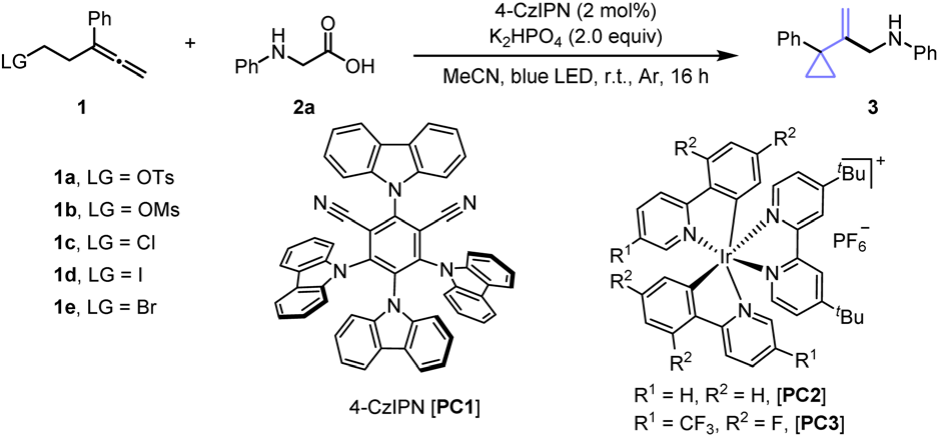				
Entry	Variation from conditions^a^	Allene	Conversion^b^	Yield^b^
1	None	1a	20	18(16)
2	Cs_2_CO_3_ as base	1a	12	11
3	K_2_HPO_4_ (1.0 equiv.)	1a	18	10
4	None	1b	45	16
5	None	1c	55	18
6	None	1d	75	23
7	None	1e	50	45(46)
8	PC2 used	1e	91	70(70)
9	PC3 used	1e	81	68(66)
10^c^	DMSO	1e	100	56
11^c^	MeCN/DMSO (9 : 1)	1e	100	68
12^c^	2.0 equiv. 2a was used	1e	98	75
13^c^^,^^d^	MeCN/DMSO (9 : 1)	1e	100	81(80)

a Reaction condition (unless otherwise specified): 1 (0.1 mmol, 1.0 equiv.), 2a (0.12 mmol, 1.2 equiv.), 4-CzIPN (2 mol%), K_2_HPO_4_ (2.0 equiv.), MeCN (2 mL), blue LED strips (452 nm), r.t. under Ar atmosphere for 16 h.

b NMR yields are reported by using dibromomethane as internal standard, isolated yield is presented in parenthesis.

c PC2 (2 mol%) was used.

d 2.0 equiv. of 2a was used. All regioselectivity >20 : 1, no cyclopentene product was detected. See the SI for full experimental details.

With the optimized reaction conditions, we then explored the substrate scope with respect to carboxylic acids (Table 2, top). Aniline derivatives bearing various substituents at the *para*-position, including halogens, electron-withdrawing groups, and electron-donating groups, also underwent the reaction with high to moderate yield (4–8). Substrates with substituents at the *meta*- and *ortho*-positions similarly exhibited successful reactivity (9–10). Structurally diverse α-amino acids reacted efficiently with 1e to yield corresponding VCPs, including those possessing various methyl (11), as well as allyl substituted (12) amino acids. Gladly, a naphthyl-functionalized amino acid was also well-tolerated (68% yield). α-Alkyl-substituted α-amino acid gave a α-substituted allylic amine derivative 14. Methionine (15), glutamic acid (16), proline (18), and α-phenyl Boc-glycine (20), also gave desired products in reasonable yields, demonstrating the ability of this methodology for late-stage functionalization of amino acids. Notably, alkyl amines, such as tetrahydroquinoline, was suitable substrate (17). Moreover, *O-p*-methoxyphenyl α-hydroxycarboxylic acids were also suitable radical precursors, and the corresponding products were obtained smoothly in the present of 4-CzIPN as catalyst (21–22).

**Table 2:** Photoredox-catalyzed cyclopropanation of allenes^a^

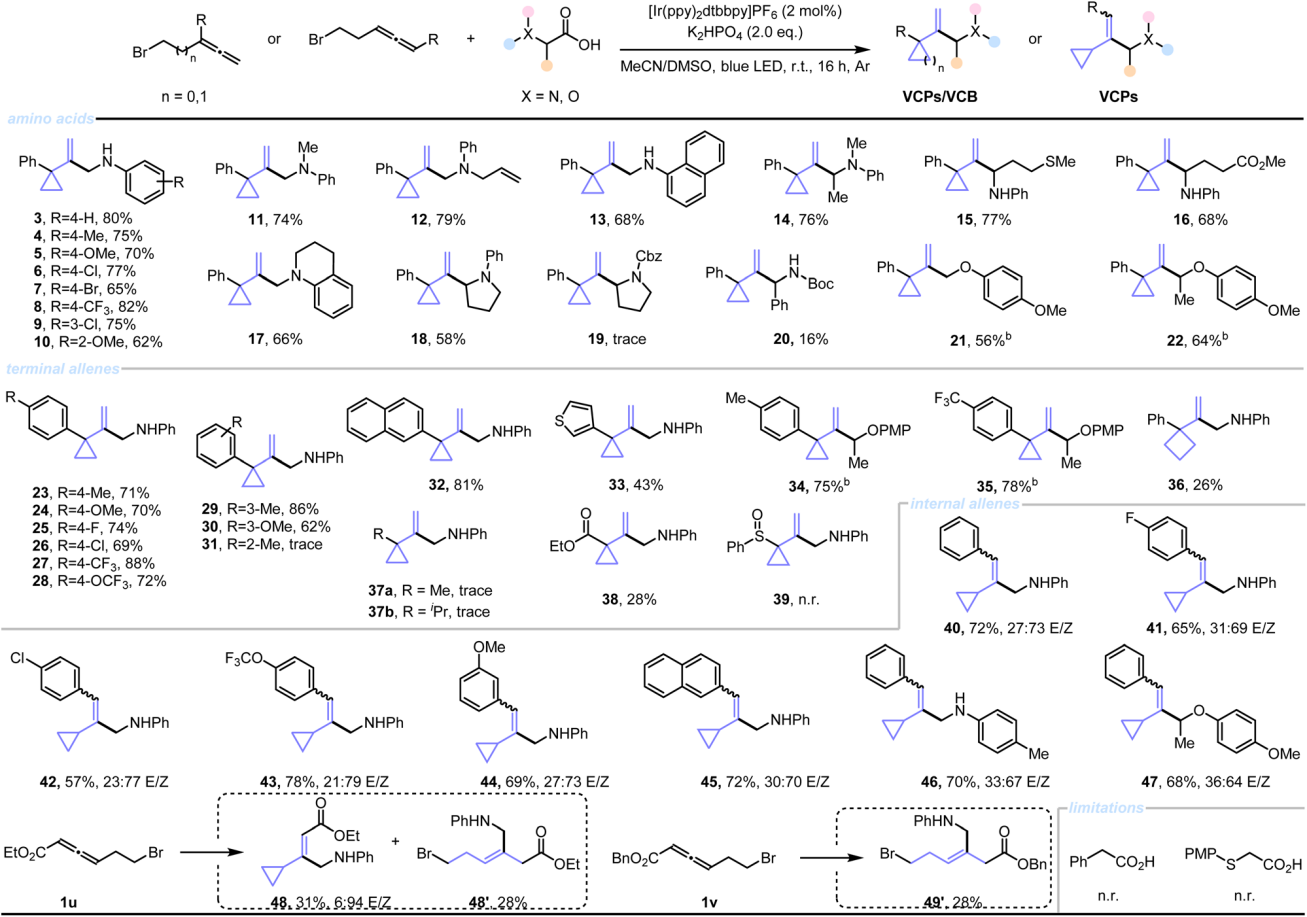

a Reaction conditions: see entry 13 of Table 1. 0.1 mmol scale. Yields reported are of the isolated, column-purified products.

b 4-CzIPN (2 mol%) was used. All regioselectivity >20 : 1.

Then, we turned our attention to determining the generality of the reaction with respect to allene substrates (Table 2, middle). It was found that diversely substituted allenes could be successfully converted to VCPs (23–30) in good to excellent yields, regardless of substitution patterns or electronic properties of substituents. Particularly, allenyl bromide bearing a naphthalene-substituent (32) and aromatic heterocycle, thiophene (33) proved to be compatible with this transformation. Moreover, α-hydroxycarboxylic acids performed well with substituted allenes under the reaction conditions, furnishing corresponding VCPs products in high yields (34–35). Interestingly, a propylbromide-substituted allene also participated in this transformation, delivering functionalized vinyl cyclobutane (VCB, 36), albeit with decreased efficiency. However, alkyl substituted allenes only gave trace amounts of products; even no reaction occurred with sulfoxide allene (37, 39). For more details, see SI, Table S7. Delightedly, the allenyl bromide equipped with an ester function delivered the VCP 38 with 28% isolated yield.

Of note, the preparation of multi-substituted olefins is one of the most challenging tasks in modern organic synthesis,^50,51^ with our strategy, the trisubstituted alkenes could be built up conveniently (Table 2, bottom). Thus, internal allene substrates were subjected to this transformation, delivering desired substituted VCPs smoothly, without the eventual formation of cyclopentene products. Various substituents on the aryl ring of aniline (40–44), as well as naphthylallene (45) were all compatible. Moreover, glycine derivative and α-hydroxycarboxylic acid also underwent the above reaction conditions to furnish desired VCPs 46–47 with good results. Gratifyingly, the substrate containing an ester function also demonstrated good reactivity, albeit giving the protonated Giese addition products 48′ and 49′.

**Scheme 2:**
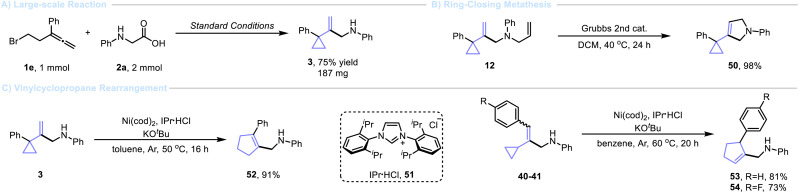
Large scale reaction and preparation of cyclopentenes.

To demonstrate the practical utility of these transformations, a 5 mmol scale reaction was performed, furnishing the corresponding cyclization product 3 in 62% yields (Scheme 2A). In order to showcase the practical value of this strategy, a series of transformations of VCPs were conducted. For example, a simple ring-closing metathesis (RCM) of 12 produced 2-pyrroline 50 (Scheme 2B), a privileged heterocycle found in many pharmacologically active natural products.^52,53^ The rearrangement of vinylcyclopropanes to cyclopentenes (the vinylcyclopropane rearrangement, VCPR) is becoming an important transformation in the synthesis of a variety of complex natural products.^4,54,55^ Ni-catalyzed VCPR reaction was performed with VCP (3) under mild conditions furnishing cyclopentene 52 in excellent yields (91% yield) (Scheme 2C). In addition, cyclopentenes 53 and 54 were smoothly obtained in 81% and 73% yields, respectively.

**Scheme 3:**
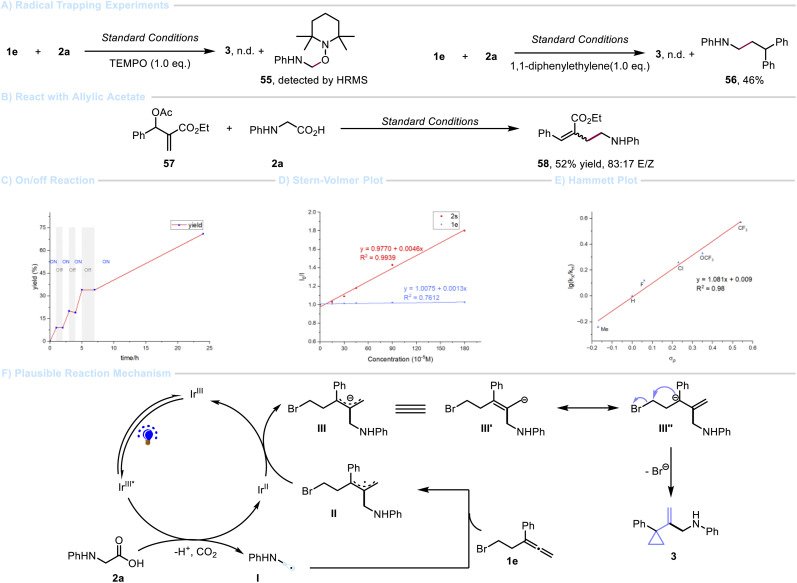
Mechanistic studies.

To gain insights into the mechanism of this method, a series of experiments was performed (Scheme 3). Both radical trapping reaction with TEMPO and Giese-type addition suggested the involvement of an α-amino carbon radical intermediate in this transformation (Scheme 3A). The formation of carbanion intermediates was confirmed by submitting allyl acetate 57 to the standard reaction conditions, during which elimination of the acetate group occurred to give alkene 58 in moderate yield (Scheme 3B). The light on/off experiment of the reaction was conducted, and its result suggested that a radical-chain propagation mechanism could be excluded (Scheme 3C), further supported by the quantum yield (*ϕ* = 0.11).^56,57^ Furthermore, Stern–Volmer luminescence quenching studies revealed that the *N*-phenyl glycine salt 2s quenches the excited-state photocatalyst, while allene 1e shows no significant quenching effect. The result indicates that the oxidation of the conjugated base of α-amino acids by the excited-state photocatalyst is likely to be the initial step of the photoredox catalytic cycle (Scheme 3D). In addition, we have performed Hammett analysis for a series of allenyl bromide by competition experiments (Scheme 3E). A positive *ρ* value (1.08) was observed, indicating the accumulation of negative charge during the rate-determining step.^58^ It is obvious that electron-withdrawing groups (EWGs) can modestly improve the reactivity, which is in agreement with an intermediate carbanion formed in the course of the reaction (Scheme 3B). Hence, the cyclization occurs through an anionic 3-*exo-tet* pathway (S_N_2), and seems more favourable than an alternative radical-based homolytic substitution (S_H_2) mechanism.^59^

On the basis of the above observations, a plausible reaction mechanism was proposed (Scheme 3F). Initially, irradiation of photocatalyst Ir^III^ affords the excited state catalyst Ir^III^*, which is reductively quenched by the carboxylate generated from *in situ* deprotonation of carboxylic acid 2, leading to Ir^II^ species^60^ and the corresponding α-amino radical I.^61^ Then, regioselective addition of α-amino radical I to allene 1e gives a radical delocalized intermediate II. A subsequent single-electron transfer (SET) between II and the reduced photocatalyst Ir^II^ gives the delocalized carbanion III and the ground state photocatalyst in order to complete the photoredox catalytic cycle.^62^ Finally, a favourable intermediate III′′ will undergo a polar 3-*exo-tet* cyclization to afford the cyclopropane product 3.

## Conclusions

In conclusion, we have described a general, modular cyclopropanation of allenes with α-amino/hydroxy radicals derived from carboxylic acid *via* a photoredox-catalyzed radical-polar crossover annulation process. Various functionalized vinyl cyclopropanes could be synthesized from terminal and internal allenes through this unified photoredox system. Moreover, this synthetic method exhibits mild reaction conditions, high chemo- and regioselectivities, a broad substrate scope, and facile scalability and derivatization, including mild isomerization to cyclopentenes, and so provides great potential for applications in organic synthesis, pharmaceutical chemistry and materials science. Further applications of this strategy in other functionalizations of alkynes, allenes, and unsaturated substrates are underway in our laboratory.

## Author contributions

H. X. and B. B. conceived and designed the experiment. H. X. performed all the experiments and analyzed all the data. Y. Z. performed the mechanistic study. H. X., B. B. wrote the manuscript.

## Conflicts of interest

The authors declare no competing financial interest.

## Supplementary Material

SC-016-D5SC05057J-s001

## Data Availability

The data supporting this article have been included in the SI. See DOI: https://doi.org/10.1039/d5sc05057j.
