# Supplementary material for: Photoredox-catalyzed cyclopropanation of allenes towards vinyl-cyclopropanes
Source: Chem Sci. 2025 Aug 14;16(36):16813–8. doi: 10.1039/d5sc05057j (PMC12366832; doi:10.1039/d5sc05057j)
Supplement: SC-016-D5SC05057J-s001 [file SC-016-D5SC05057J-s001.pdf]

Supplementary Information (SI) for Chemical Science.
This journal is © The Royal Society of Chemistry 2025

Supporting Information

Photoredox-Catalyzed Cyclopropanation of Allenes towards Vinylcyclopropanes

Hui Xie,[a] Yan Zhang[a] and Bernhard Breit*,[a]

[a]Institut für Organische Chemie, Albert-Ludwigs-Universität Freiburg, Albertstraße 21, 79104 Freiburg im Breisgau, Germany

E-mail: bernhard.breit@chemie.uni-freiburg.de

Table of Contents

1. General Information S2

2. List and Preparation of Allenes S4

3. List and Preparation of Carbonic Acids. S32

4. Optimization of the Reaction Conditions. S35

5. General Procedure E for the Cyclization Reaction. S39

6. Product Characterization. S40

7. Synthetic Application. S66

8. Mechanism Studies. S70

9. Reaction Limitations. S79

10. Reference. S80

11. Copies of NMR Spectr S80

1. General Information

1.1 Analytical Techniques

TLC (Analytical thin layer chromatography) was performed on aluminum plates pre-coated with silica gel (MERCK, 60 F-254), compounds were visualized by exposure to UV fluorescence (λ_{max} = 254 nm) and/or by staining with 10% phosphomolybdic acid in EtOH and/or by staining with 1% w/v $KMnO_4$ in 0.5 M aqueous K_2CO_3.

NMR (Nuclear Magnetic Resonance) spectra were recorded on BRUKER Avance III HD 300 MHz, BRUKER Avance II 400 MHz, Bruker 500 MHz DRX NMR or Bruker Avance III Neo 700 MHz spectrometer at ambient temperature. Chemical shifts were given in dimensionless δ values and were frequency referenced relative to TMS in ^{1}H and ^{13}C NMR spectroscopy. Chemical shifts are quoted as parts per million (ppm) relative to tetramethylsilane (s = singlet, d = doublet, t = triplet, q = quartet, quint = quintet, m = multiplet, brs = broad singlet) and coupling constants (*J*) are quoted as Hertz (Hz) and intergration.

HRMS (High Resolution Mass Spectra) was measured on a THERMO SCIENTIFIC Advantage and a THERMO SCIENTIFIC Exactive instrument equipped with an APCI or ESI source.

Stern-Volmer quenching experiment were performed using a PerkinElmer LS45 Fluorescence Spectrometer.

Melting points for solids were measured on a BÜCHI Dr. Tottoli melting point apparatus and are given uncorrected.

1.2. Compound Purification

Chromatographic purification was accomplished by flash column chromatography was performed with Macherey-Nagel silica gel 60® (0.04-0.063mm, 230-400 mesh) or on an Ultra Performance Flash Purification System-puriFlash® XS 430 and a PF-30C18AQ-F0025 column.

1.3. Reagents, Solvents and Experimental Conditions

All reagents were purchased from Sigma-Aldrich, TCI, Fisher Scientific, Carbolution, ABCR, VWR, BLD Pharmatech GmbH and used without further purification, except otherwise stated. All other solvents were bought from Acros in AcroSeal® bottles and were directly stored under 3 or 4Å molecular sieves, replacing the collected volume with argon. Unless specified otherwise, all reactions were carried out under nitrogen atmosphere using flame-dried glassware.

1.4. Photochemical Set-up

Photochemical reactions, unless otherwise stated, were performed in a 4.8 W, 3528 300 Blue Leds strip (λ_{max} = 452 nm) and a computer case fan (95 mm) as cooling system was used to run the catalysis, see Figure S1, left and middle. The strip was wrapped around a 125 × 65 mm Pyrex crystallization dish 4-5 times. The photocatalysis reactions were carried out in a 10 mL Schlenk tube as shown in figure S1, right. Reactions was generally placed ~1 cm away from the lights (the walls of the dish), by placing them in a vial holder. The wavelength of blue LEDs strips was shown in Figure S2.

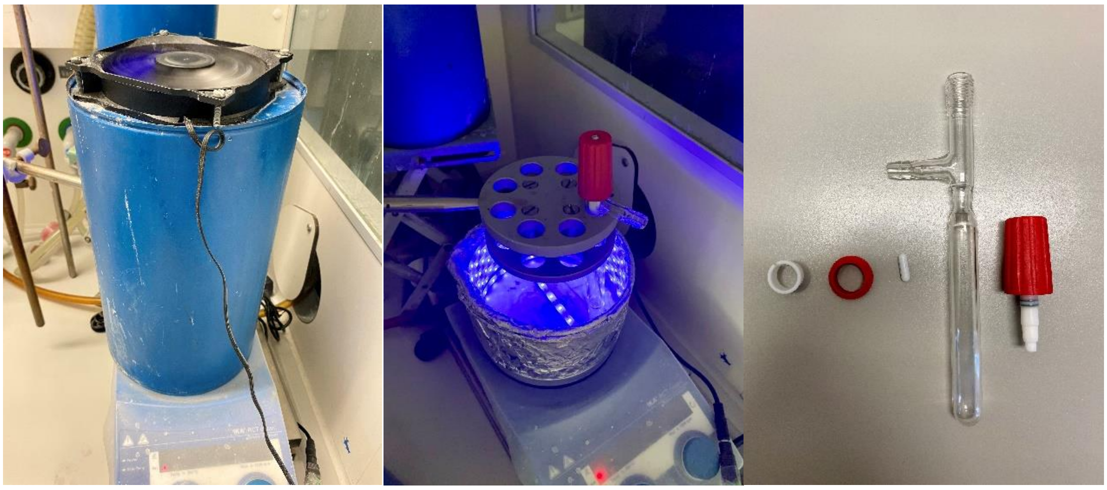

Figure S1. Photochemical reaction setup and 10 mL Schlenk tube.

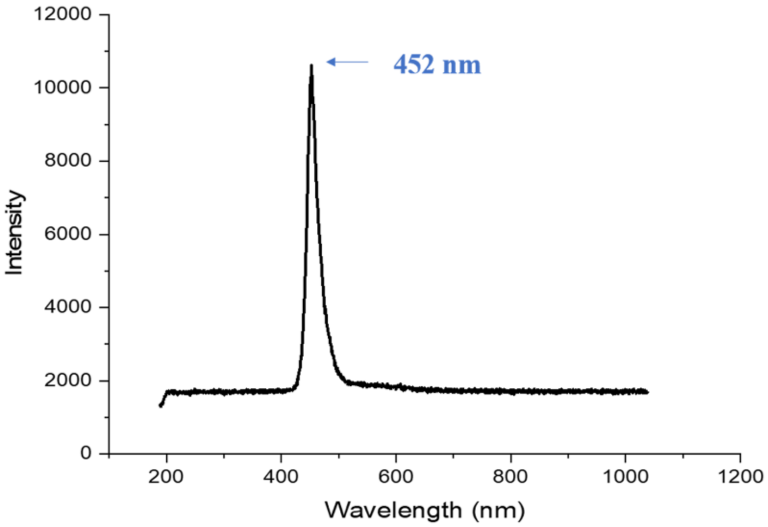

Figure S2. Wavelength of blue LEDs strips.

2. List and Preparation of Allenes .

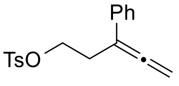

1a

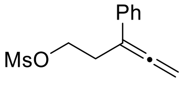

1b

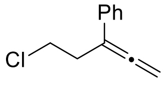

1c

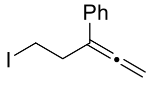

1d

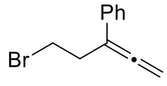

1e

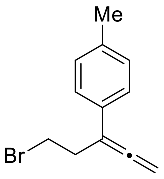

1f

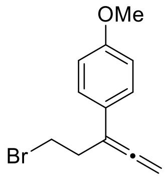

1g

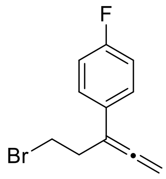

1h

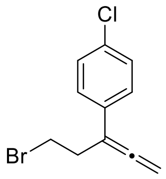

1i

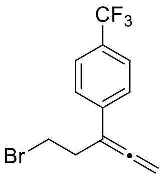

1j

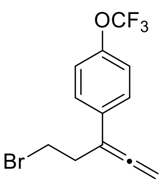

1k

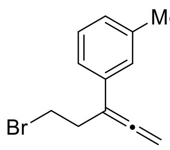

1l

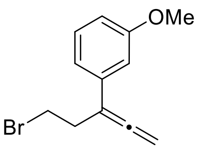

1m

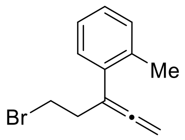

1n

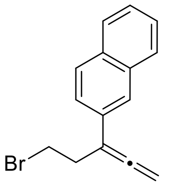

1o

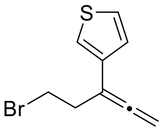

1p

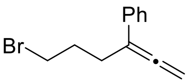

1q

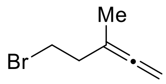

1r

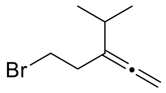

1s

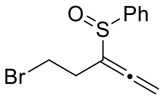

1t

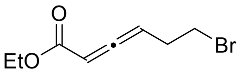

1u

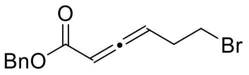

1v

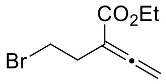

1w

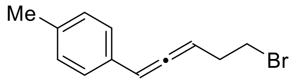

1f'

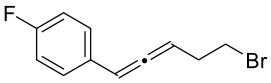

1h'

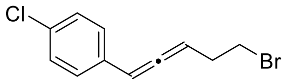

1i'

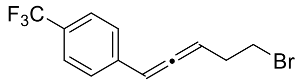

1j'

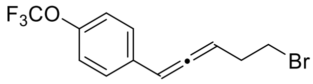

1k'

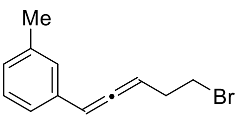

1l'

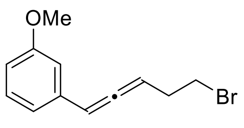

1m'

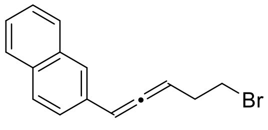

1o'

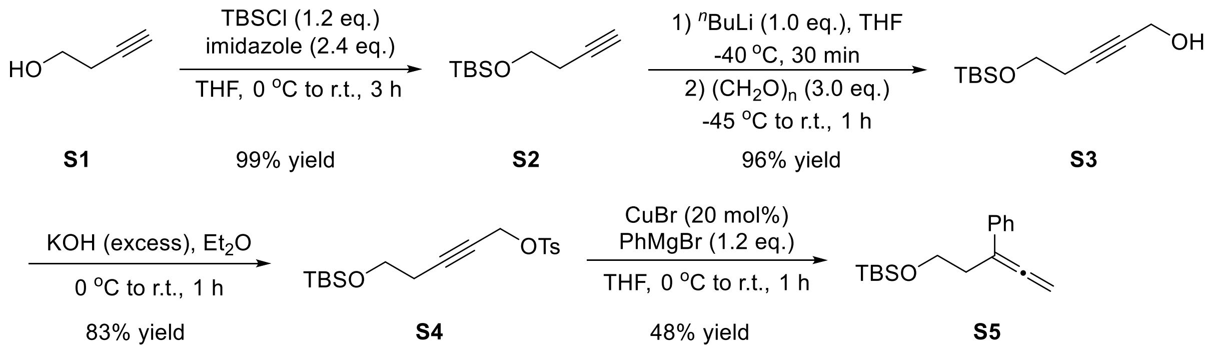

(but-3-yn-1-yloxy)(tert-butyl)dimethylsilane (S2)[1]

To a solution of 3-butyn-1-ol **S1** (4.60 g, 66 mmol, 1.0 equiv.) in THF (100 mL) was added imidazole (10.8 g, 158.5 mmol, 2.4 equiv.) and *tert*-butyl dimethylsilyl chloride (TBSCl) (11.95 g, 79.3 mmol, 1.2 equiv.) at 0 °C. After stirring at ambient temperature for 3 h, the reaction mixture was filtered through a pad of Celite and washed with 20 mL of EtOAc. The filtrate was concentrated under reduced pressure to give the desired product **S2** (12.15 g, 99% yield) as a colorless oil with quantity yield, which used in next step without further purification.

5-((tert-butyldimethylsilyl)oxy)pent-2-yn-1-ol (S3)[2]

An oven-dried three-necked flask was charged with (but-3-yn-1-yloxy)(*tert*-butyl)dimethylsilane **S2** (9.22 g, 50 mmol, 1.0 equiv.) and dry anhydrous THF (100 mL) under an argon atmosphere and cooled to -40 °C. ^{n}BuLi (22 mL, 2.5 M in toluene, 55 mmol, 1.1 equiv.) was added dropwise to the reaction solution, and the reaction mixture was stirred for 1 h before the solution was transferred through a teflon tube to a suspension of paraformaldehyde (3.75 g, 125 mmol, 2.5 equiv.) in THF (50 ml) at −45 °C. The reaction mixture was stirred at ambient temperature for 1 h. The reaction was then quenched by addition of sat. aq. NH_4Cl solution, and extracted with EtOAc (2 x 100 mL), dried over with anhydrous Na_2SO_4, filtered and concentrated *in vacuo*. The crude product was purified by flash column chromatography on silica gel (Et_2O) to give the desired product **S3** (10.275 g, 96% yield) as a colorless oil.

5-((tert-butyldimethylsilyl)oxy)pent-2-yn-1-yl 4-methylbenzenesulfonate (S4)[3]

To a solution of propargyl alcohol **S3** (10.72 g, 50 mmol, 1.0 equiv.) in Et_2O (75 mL) at 0 °C was added TsCl (1.2 equiv.). Then, KOH (fresh pestled, 5.0 equiv.) was added slowly in small portions. The solution was allowed to warm to room temperature and stirred for 30 min. The reaction mixture was poured on ice. The aqueous phase was extracted with Et_2O (3 × 50 mL). The combined organic phases were washed with brine and dried over Na_2SO_4. The solvents

were removed under reduced pressure and the crude product was purified by flash column chromatography on silica gel (Pentanes/Et_2O – 5:1) to give the desired product **S4** (15.27 g, 83% yield) as a colorless oil.

R*f* = 0.35 (pentane/Et_2O – 4:1).

^{1}H NMR (400 MHz, CDCl3) δ = 7.83 – 7.79 (m, 2H), 7.36 – 7.32 (m, 2H), 4.69 (t, *J* = 2.2 Hz, 2H), 3.60 (t, *J* = 7.1 Hz, 2H), 2.45 (s, 3H), 2.30 (tt, *J* = 7.1, 2.2 Hz, 2H), 0.88 (s, 9H), 0.04 (s, 6H).

^{13}C NMR (101 MHz, $CDCl_3$) δ = 145.0, 133.5, 129.8, 128.2, 87.6, 73.1, 61.4, 58.6, 25.9, 23.2, 21.7, 18.4, -5.2.

HRMS (+ p ESI) m/z: $[M+Na]^+$ Calcd for $C_{18}H_{28}O_4NaSi^+$: 391.1370; Found: 391.1375

tert-butyldimethyl((3-phenylpenta-3,4-dien-1-yl)oxy)silane (S5)

An oven-dried two-neched flask was charged with CuBr (20 mol%) and dry anhydrous THF (0.33 M) under an argon atmosphere and cooled to 0 °C. Tosylate **S4** (1.0 equiv.) was added to the reaction mixture. Then, fresh Grignard reagent (1.2 equiv.) was added drop wise to the reaction solution. The reaction mixture was allowed to warm to room temperature and stirred for 2 h. It was quenched by addition of a saturated aqueous solution of NH_4Cl, followed by extraction with EtOAc (3 × 20 ml). The combined organic phases were washed with brine and dried over Na_2SO_4. The solvents were removed under reduced pressure and the crude product was purified by column chromatography on silica gel (Pentanes) to give the allene **S5** (48% yield) as a colorless oil.

R*f* = 0.45 (pentane).

^{1}H NMR (400 MHz, $CDCl_3$) δ = 7.43 – 7.39 (m, 2H), 7.34 – 7.29 (m, 2H), 7.22 – 7.16 (m, 1H), 5.06 (td, *J* = 3.0, 0.6 Hz, 2H), 3.81 (dd, *J* = 7.6, 7.0 Hz, 2H), 2.67 (ddt, *J* = 7.7, 7.1, 3.0 Hz, 2H), 0.90 (s, 9H), 0.05 (s, 6H).

^{13}C NMR (101 MHz, $CDCl_3$) δ = 208.9, 136.2, 128.5, 126.7, 126.1, 101.9, 78.1, 62.2, 34.2, 26.0, 14.1, -5.2.

HRMS (+ p APCI) m/z: $[M+H]^+$ Calcd for $C_{17}H_{26}OSi^+$: 275.1826; Found: 275.1824.

Note: In the copper-catalyzed Grignard reaction to prepare allenes (S_N2'), we cannot avoid the formation of S_N2 coupling products (alkyne). By adding fresh Grignard reagent slowly and keep the temperature, the proportion of S_N2' could be increased. We have optimized the solvent (Et_2O, THF, toluene) and the temperature (from -10 ^oC to -78 ^oC), the optimal conditions (THF, 0 ^oC) could give the better result (allene S_N2' : alkyne S_N2 = 1:1.2).

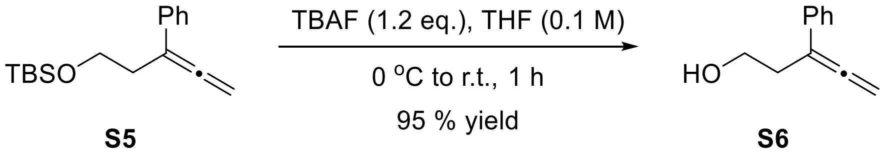

3-phenylpenta-3,4-dien-1-ol (S6)

To a solution of TBS protected allene **S5** (2.74 g, 10 mmol, 1.0 equiv.) in THF (100 mL, 0.1 M) at 0 °C was added TABF, tetrabutylammonium fluoride (12 mL, 12 mmol, 1.0 M in THF, 1.2 equiv.) slowly. The solution was allowed to warm to room temperature and stirred for additional 60 min, and the substrate was monitored by TLC. The solvents were removed under reduced pressure and the crude product was purified by flash column chromatography on silica gel (Pentanes/Et_2O – 3:2, 1% Et_3N) to give the desired product **S6** (1.52 g, 95% yield) as a colorless oil.

$\mathbf{R}_f$ = 0.25 (pentane/Et_2O – 2:1).

^{1}**H NMR** (300 MHz, $CDCl_3$) δ = 7.39 – 7.05 (m, 5H), 5.06 (t, J = 3.3 Hz, 2H), 2.62 (tt, J = 6.4, 3.3 Hz, 2H), 2.41 (tt, J = 6.2, 2.4 Hz, 2H).

HRMS (+ p APCI) m/z: $[M+H]^+$ Calcd for $C_{11}H_{12}O^+$: 161.0961; Found: 161.0959.

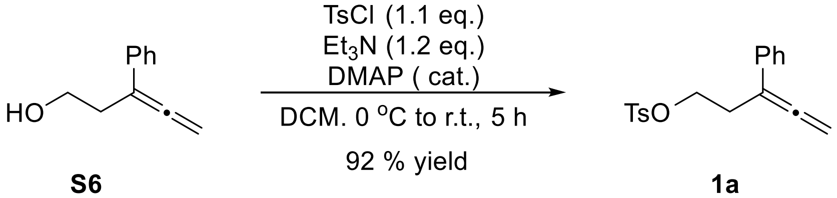

3-phenylpenta-3,4-dien-1-yl 4-methylbenzenesulfonate (1a)

To a solution of alcohol allene **S6** (800 mg, 5 mmol, 1.0 equiv.) in DCM (20 mL) at 0 °C was added Et_3N (0.84 mL, 6 mmol, 1.2 equiv.), and the solution was stirred for 5 min. Then, TsCl (1.048 g, 5.5 mmol, 1.1 equiv.) was added, and DMAP, 4-dimethylaminopyridine (*cat.* amount) was added subsequently. The solution was allowed to warm to room temperature and stirred for additional 5 hours. The reaction mixture was quenched by water (15 mL). The aqueous phase was extracted with DCM (3 × 15 mL). The combined organic phases were washed with brine and dried over Na_2SO_4. The solvents were removed under reduced pressure and the crude product was purified by flash column chromatography on silica gel (Pentanes/Et_2O – 4:1) to give the desired product **1a** (1.445g, 92% yield) as a white solid (m.p.: 112 ± 2, pentane).

$\mathbf{R}_f$ = 0.25 (pentane/Et_2O – 4:1).

^{1}H NMR (400 MHz, $CDCl_3$) δ = 7.80 – 7.75 (m, 2H), 7.35 – 7.26 (m, 6H), 7.23 – 7.17 (m, 1H), 5.07 (td, J = 3.2, 0.5 Hz, 2H), 4.24 (t, J = 7.1 Hz, 2H), 2.79 (tt, J = 7.1, 3.2 Hz, 2H), 2.44 (s, 3H).
^{13}C NMR (101 MHz, $CDCl_3$) δ = 208.3, 144.8, 135.1, 133.4, 129.9, 128.6, 128.0, 127.1, 125.8, 100.4, 79.6, 68.6, 29.0, 21.7.
HRMS (+ p ESI) m/z: $[M+Na]^+$ Calcd for $C_{18}H_{18}O_3NaS^+$: 337.0869; Found: 337.0867.

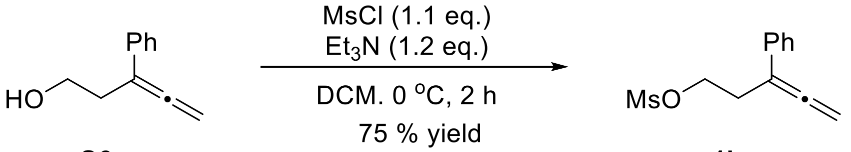

S6 **1b**

3-phenylpenta-3,4-dien-1-yl methanesulfonate (1b)
To a solution of alcohol allene **S6** (800 mg, 5 mmol, 1.0 equiv.) in DCM (15 mL) at 0 °C was added Et_3N (0.78 mL, 5.5 mmol, 1.1 equiv.), and the solution was stirred for 10 min. Then, MsCl (0.46 mL, 6 mmol, 1.2 equiv.) was added dropwise. The solution stirred for additional 2 hours at the same temperature. Then, the reaction mixture was quenched by water (15 mL). The aqueous phase was extracted with DCM (3 × 15 mL). The combined organic phases were washed with brine and dried over Na_2SO_4. The solvents were removed under reduced pressure and the crude product was purified by flash column chromatography on silica gel (Pentanes/Et_2O – 4:1) to give the desired product **1b** (892 mg, 75% yield) as a colorless oil.
R_f = 0.35 (pentane/Et_2O – 4:1).
^{1}H NMR (400 MHz, $CDCl_3$) δ = 7.40 – 7.31 (m, 4H), 7.26 – 7.21 (m, 1H), 5.18 (td, J = 3.3, 0.5 Hz, 2H), 4.43 (t, J = 7.1 Hz, 2H), 2.99 (s, 2H), 2.90 (tt, J = 7.1, 3.3 Hz, 2H).
^{13}C NMR (101 MHz, $CDCl_3$) δ = 208.4, 135.1, 128.7, 127.3, 125.9, 100.6, 79.8, 68.1, 37.6, 29.3.
HRMS (+ p ESI) m/z: $[M+Na]^+$ Calcd for $C_{12}H_{14}O_3S^+$: 239.0376; Found: 239.0378.

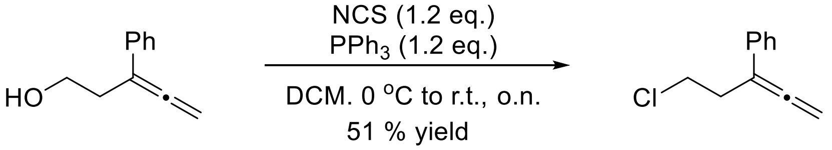

S6 **1c**

(5-chloropenta-1,2-dien-3-yl)benzene (1c)
To a solution of alcohol allene **S6** (800 mg, 5 mmol, 1.0 equiv.) in DCM (18 mL) at 0 °C was

added NCS, N-chlorosuccinimide (800 mg, 6 mmol, 1.2 equiv.) and triphenylphosphine (1.573 g, 6 mmol, 1.2 equiv.). Then, the solution was allowed to warm to room temperature and stirred overnight. The reaction mixture was quenched by water (15 mL), and the aqueous phase was extracted with DCM (3 × 15 mL). The combined organic phases were washed with brine and dried over Na_2SO_4. The solvents were removed under reduced pressure and the crude product was purified by flash column chromatography on silica gel (Pentanes) to give the desired product **1c** (455 mg, 51% yield) as a colorless oil.

R*f* = 0.45 (pentane).

^{1}H NMR (400 MHz, $CDCl_3$) δ = 7.40 – 7.31 (m, 4H), 7.25 – 7.20 (m, 1H), 5.17 (td, *J* = 3.2, 0.5 Hz, 2H), 3.70 (t, *J* = 7.4 Hz, 2H), 2.90 (ddt, *J* = 7.9, 7.3, 3.2 Hz, 2H).

^{13}C NMR (101 MHz, $CDCl_3$) δ = 208.5, 135.4, 128.7, 127.1, 125.9, 102.2, 79.5, 42.5, 33.0.

HRMS (+ p APCI) m/z: $[M+H]^+$ Calcd for $C_{11}H_{11}{}^{35}Cl^+$: 179.0622; Found: 179.0621.

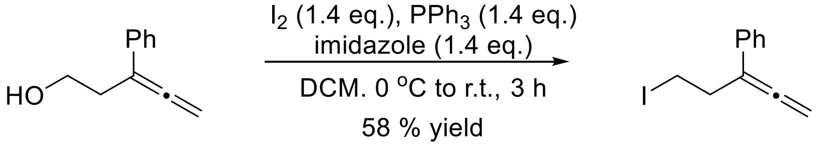

S6 **1e**

(5-iodopenta-1,2-dien-3-yl)benzene (1e)

To a solution of alcohol allene **S6** (800 g, 5 mmol, 1.0 equiv.) in DCM (18 mL) at 0 °C was added I_2 (1.776 g, 7 mmol, 1.4 equiv.), triphenylphosphine (1.836 g, 7 mmol, 1.4 equiv.) and imidazole (477 mg, 7 mmol, 1.4 equiv.) under dark. Then, the solution was allowed to warm to room temperature and stirred for additional 3 hours. The reaction mixture was quenched by water, and the aqueous phase was extracted with DCM (3 × 20 mL). The combined organic phases were washed with brine and dried over Na_2SO_4. The solvents were removed under reduced pressure and the crude product was purified by flash column chromatography on silica gel (Pentanes) to give the desired product **1e** (780 mg, 58% yield) as a colorless oil.

R*f* = 0.45 (pentane).

^{1}H NMR (400 MHz, $CDCl_3$) δ = 7.39 – 7.31 (m, 4H), 7.25 – 7.19 (m, 2H), 5.17 (td, *J* = 3.2, 0.5 Hz, 2H), 3.33 (td, *J* = 7.4, 0.4 Hz, 2H), 3.06 – 2.96 (m, 2H).

^{13}C NMR (101 MHz, $CDCl_3$) δ = 208.4, 135.2, 128.7, 127.1, 126.0, 105.1, 79.9, 34.0, 2.7.

HRMS (+ p APCI) m/z: $[M+H]^+$ Calcd for $C_{11}H_{11}{}^{126}I^+$: 270.9978; Found: 270.9977.

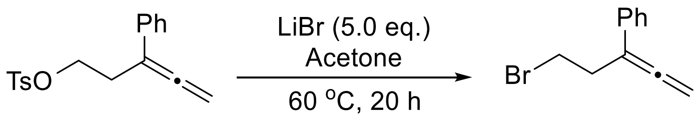

1a **1d**

(5-bromopenta-1,2-dien-3-yl)benzene (1d)

To a solution of tosylate allene **1a** (1.572 g, 5 mmol, 1.0 equiv.) in acetone (40 mL, 0.12 M) was added LiBr (2.175g, 25 mmol, 5.0 equiv.). Then, the solution was stirred at 60 °C for 20 hours. The solvents were removed under reduced pressure and the crude product was purified by flash column chromatography on silica gel (Pentanes) to give the desired product **1d**(970 mg, 87% yield) as a colorless oil.

R*f* = 0.45 (pentane).

^{1}H NMR (400 MHz, $CDCl_3$) δ = 7.40 – 7.31 (m, 4H), 7.25 – 7.20 (m, 1H), 5.17 (td, *J* = 3.2, 0.5 Hz, 2H), 3.55 (d, *J* = 7.6 Hz, 2H), 2.99 (ddt, *J* = 8.0, 7.4, 3.2 Hz, 2H).

^{13}C NMR (101 MHz, $CDCl_3$) δ = 208.5, 135.3, 128.7, 127.1, 125.9, 103.3, 79.7, 33.2, 30.4.

HRMS (+ p APCI) m/z: $[M+H]^+$ Calcd for $C_{11}H_{11}{}^{79}Br^+$: 223.0117; Found: 223.0117.

General Procedure A:

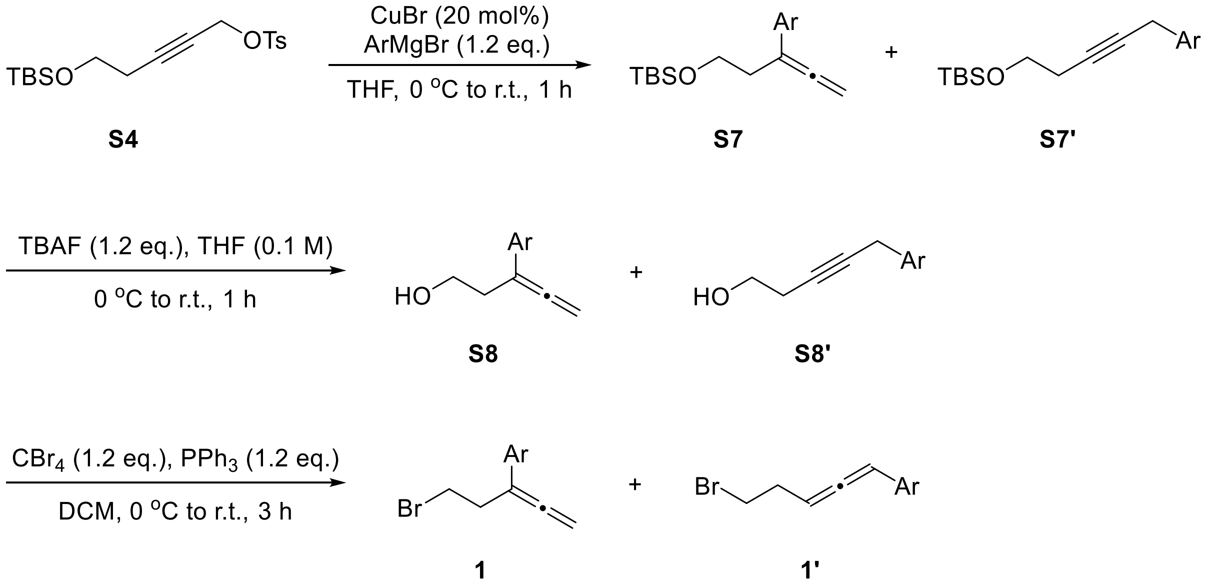

Step 1. Cu-catalyzed Grignard Reaction. An oven-dried two-neched flask was charged with CuBr (72 mg, 1.0 mmol, 10 mol%) and dry anhydrous THF (15 mL, 0.33 M) under an argon atmosphere and cooled to 0 °C. Tosylate **S4** (5.0 mmol, 1.0 equiv.) was added to the reaction mixture. Then, fresh Grignard reagent (1.2 equiv.) was added drop wise to the reaction solution. The reaction mixture was allowed to warm to room temperature and stirred for 2 h. It was quenched by addition of a saturated aqueous solution of NH_4Cl, followed by extraction with

EtOAc (3 × 20 ml). The combined organic phases were washed with brine and dried over Na_2SO_4. The solvents were removed under reduced pressure and the crude product was purified by column chromatography on silica gel (Pentanes) to give the allene and alkyne mixture **S7** as a colorless oil.

(*Most case, both isomers couldn't separate by silica gel column chromatography, which were subjected to the next step subsequently.*)

Step 2. Desilylation of Allenes and Alkynes. To a solution of TBS protected allene and alkyne **S7** (1.0 equiv.) in THF (0.1 M) at 0 °C was added TABF, tetrabutylammonium fluoride (1.0 M in THF, 1.2 equiv.) slowly. The solution was allowed to warm to room temperature and stirred for additional 60 min, and the substrate was monitored by TLC. The solvents were removed under reduced pressure and the crude product was purified by flash column chromatography on silica gel (Pentanes/Et_2O – 3:2, 1% Et_3N) to give the alcohol allene and alkyne **S8** mixtures a colorless oil.

(*Most case, both isomers couldn't separate by column chromatography, which were subjected to the next step subsequently.*)

Step 3. Appel Reaction. To a solution of alcohol allene and alkyne **S8** (1.0 equiv.) in DCM (0.12 M) at 0 °C was added CBr_4 (1.2 equiv.) and triphenylphosphine (1.2 equiv.). Then, the solution was allowed to warm to room temperature and stirred for 3 hours. The reaction mixture was quenched by water (15 mL), and the aqueous phase was extracted with DCM (3 × 15 mL). The combined organic phases were washed with brine and dried over Na_2SO_4. The solvents were removed under reduced pressure and the crude product was purified by flash column chromatography on silica gel (Pentanes) to give the desired product **1** as a colorless oil.

(Both isomers, internal and terminal allenes, could be separated by column chromatography)

Note: The alkyne was subjected to the Appel reaction condition, and the basic atmosphere allows it to isomerize to an internal allene.

(5-bromopenta-1,2-dien-3-yl)benzene (1d')

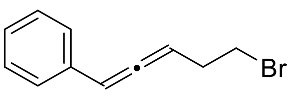

1e'

Following the **General Procedure A**, after concentration, the crude material was purified by flash column chromatography (SiO_2, Pentane) to furnish the title compound as a colorless oil (245 mg, 22% yield, 3 steps).

R_f = 0.40 (pentane).

^{1}H NMR (400 MHz, $CDCl_3$) δ = 7.33 – 7.28 (m, 4H), 7.24 – 7.17 (m, 1H), 6.22 (dt, *J* = 6.4, 2.9 Hz, 1H), 5.61 (q, *J* = 6.6 Hz, 1H), 3.49 (t, *J* = 7.1 Hz, 2H), 2.75 – 2.65 (m, 2H).

^{13}C NMR (101 MHz, $CDCl_3$) δ = 205.9, 134.2, 128.7, 127.2, 126.9, 96.0, 92.6, 32.3, 31.8.

HRMS (+ p APCI) m/z: $[M+H]^+$ Calcd for $C_{11}H_{11}{}^{79}Br^+$: 223.0117; Found: 223.0116.

1-(5-bromopenta-1,2-dien-3-yl)-4-methylbenzene (1f)

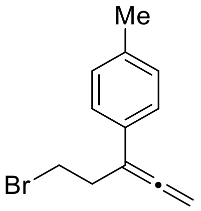

1f

Following the **General Procedure A**, after concentration, the crude material was purified by flash column chromatography (SiO_2, Pentane) to furnish the title compound as a colorless oil (282 mg, 24% yield, 3 steps).

R_f = 0.40 (pentane).

^{1}H NMR (400 MHz, $CDCl_3$) δ = 7.28 – 7.25 (m, 2H), 7.16 – 7.13 (m, 2H), 5.15 (t, *J* = 3.1 Hz, 2H), 3.54 (t, *J* = 7.6 Hz, 2H), 2.97 (tt, *J* = 7.5, 3.2 Hz, 2H), 2.34 (s, 3H).

^{13}C NMR (101 MHz, $CDCl_3$) δ = 208.3, 136.9, 132.2, 129.4, 125.8, 103.1, 79.5, 33.2, 30.5, 21.1.

HRMS (+ p APCI) m/z: $[M+H]^+$ Calcd for $C_{12}H_{13}{}^{81}Br^+$: 239.0254; Found: 239.0252.

1-(5-bromopenta-1,2-dien-1-yl)-4-methylbenzene (1f')

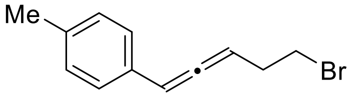

1f'

Following the **General Procedure A**, after concentration, the crude material was purified by flash column chromatography (SiO_2, Pentane) to furnish the title compound as a colorless oil (355 mg, 30% yield, 3 steps).

R_f = 0.38 (pentane).

^{1}H NMR (500 MHz, $CDCl_3$) δ = 7.23 – 7.18 (m, 2H), 7.14 – 7.09 (m, 2H), 6.19 (dt, *J* = 6.0, 2.9 Hz, 1H), 5.59 (q, *J* = 6.6 Hz, 1H), 3.66 – 3.44 (m, 2H), 2.68 (qt, *J* = 7.0, 2.9 Hz, 2H), 2.33

(s, 3H).

^{13}C NMR (126 MHz, $CDCl_3$) δ = 205.6, 137.0, 131.2, 129.4, 126.8, 95.8, 92.5, 32.3, 31.9, 21.3.

HRMS (+ p APCI) m/z: $[M+H]^+$ Calcd for $C_{12}H_{13}{}^{79}Br^+$: 237.0273; Found: 237.0273.

1-(5-bromopenta-1,2-dien-3-yl)-4-methoxybenzene (1g)

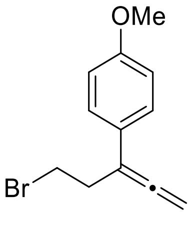

1g

Following the **General Procedure A**, after concentration, the crude material was purified by flash column chromatography (SiO_2, Pentane/DCM – 10:1) to furnish the title compound as a colorless oil (226 mg, 18% yield, 3 steps).

R*f* = 0.35 (pentane/DCM – 10:1).

^{1}H NMR (500 MHz, $CDCl_3$) δ = 7.32 – 7.27 (m, 2H), 6.90 – 6.85 (m, 2H), 5.14 (t, *J* = 3.2 Hz, 2H), 3.81 (s, 3H), 3.54 (dd, *J* = 7.9, 7.3 Hz, 2H), 2.99 – 2.92 (m, 2H).

^{13}C NMR (126 MHz, $CDCl_3$) δ = 208.1, 158.9, 127.4, 127.0, 114.2, 102.8, 79.6, 55.4, 33.3, 30.5.

HRMS (+ p APCI) m/z: $[M+H]^+$ Calcd for $C_{12}H_{13}O^{79}Br^+$: 253.0220; Found: 253.0223.

1-(5-bromopent-2-yn-1-yl)-4-methoxybenzene (1g')

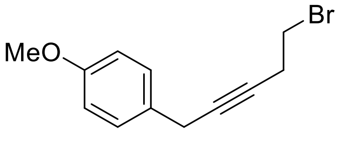

1g'

Following the **General Procedure A**, after concentration, the crude material was purified by flash column chromatography (SiO_2, Pentane/DCM – 10:1) to furnish the title compound as a colorless oil (378 mg, 30% yield, 3 steps).

(Note: due to the electricity of the substrate, the para-methoxyl substituted alkyne didn't isomerize to the internal allene)

R*f* = 0.30 (pentane/DCM – 10:1).

^{1}H NMR (500 MHz, $CDCl_3$) δ = 7.28 – 7.22 (m, 2H), 6.88 – 6.83 (m, 2H), 3.80 (s, 3H), 3.52

(t, J = 2.4 Hz, 2H), 3.45 (t, J = 7.3 Hz, 2H), 2.79 (tt, J = 7.3, 2.4 Hz, 2H).

^{13}C NMR (126 MHz, $CDCl_3$) δ = 158.4, 129.0, 128.9, 114.0, 80.6, 79.0, 55.4, 30.3, 24.3, 23.5.

HRMS (+ p APCI) m/z: $[M+H]^+$ Calcd for $C_{12}H_{13}O^{79}Br^+$: 253.0220; Found: 253.0219.

1-(5-bromopenta-1,2-dien-3-yl)-4-fluorobenzene (1h)

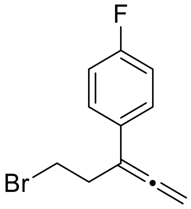

1h

Following the **General Procedure A**, after concentration, the crude material was purified by flash column chromatography (SiO_2, Pentane) to furnish the title compound as a colorless oil (216 mg, 18% yield, 3 steps).

R*f* = 0.40 (pentane).

^{1}H NMR (700 MHz, $CDCl_3$) δ = 7.35 – 7.31 (m, 2H), 7.05 – 7.00 (m, 2H), 5.18 (tdd, J = 3.3, 1.1, 0.5 Hz, 2H), 3.53 (dd, J = 7.7, 7.2 Hz, 2H), 2.96 (tt, J = 7.3, 3.2 Hz, 2H).

^{19}F NMR (659 MHz, $CDCl_3$) δ = -115.48.

^{13}C NMR (176 MHz, $CDCl_3$) δ = 208.3, 162.0 (d, J = 246.5 Hz), 131.3 (d, J = 3.4 Hz), 127.5 (d, J = 8.0 Hz), 115.6 (d, J = 21.7 Hz), 102.5, 80.0, 33.2, 30.2.

HRMS (+ p APCI) m/z: $[M+H]^+$ Calcd for $C_{11}H_{10}F^{79}Br^+$: 241.0023; Found: 241.0021.

1-(5-bromopenta-1,2-dien-1-yl)-4-fluorobenzene (1h')

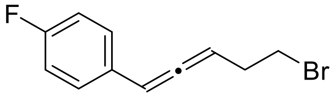

1h'

Following the **General Procedure A**, after concentration, the crude material was purified by flash column chromatography (SiO_2, Pentane) to furnish the title compound as a colorless oil (264 mg, 22% yield, 3 steps).

R*f* = 0.38 (pentane/Et_2O – 40:1).

^{1}H NMR (500 MHz, $CDCl_3$) δ = 7.30 – 7.24 (m, 2H), 7.04 – 6.96 (m, 2H), 6.19 (dt, J = 6.4, 2.9 Hz, 1H), 5.61 (qd, J = 6.6, 1.3 Hz, 1H), 3.56 (dtd, J = 74.3, 6.8, 0.8 Hz, 2H), 2.76 – 2.55 (m, 2H).

^{19}**F NMR** (476 MHz, $CDCl_3$) δ = -115.22.

^{13}**C NMR** (126 MHz, $CDCl_3$) δ = 205.7, 162.1 (d, *J* = 246.2 Hz), 130.2 (d, *J* = 3.4 Hz), 128.4 (d, *J* = 8.1 Hz), 115.7 (d, *J* = 21.8 Hz), 95.0, 92.8, 32.2, 31.8.

HRMS (+ p APCI) m/z: $[M+H]^+$ Calcd for $C_{11}H_{10}F^{79}Br^+$: 241.0023; Found: 241.0022.

1-(5-bromopenta-1,2-dien-3-yl)-4-chlorobenzene (1i)

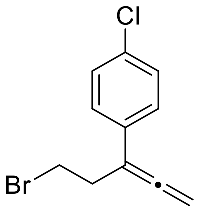

1i

Following the **General Procedure A**, after concentration, the crude material was purified by flash column chromatography (SiO_2, Pentane) to furnish the title compound as a colorless oil (190 mg, 15% yield, 3 steps).

R*f* = 0.40 (pentane).

^{1}**H NMR** (400 MHz, $CDCl_3$) δ = 7.30 – 7.29 (m, 4H), 5.19 (t, *J* = 3.2 Hz, 2H), 3.53 (dd, *J* = 7.7, 7.2 Hz, 2H), 2.95 (ddt, *J* = 7.8, 7.3, 3.2 Hz, 2H).

^{13}**C NMR** (101 MHz, $CDCl_3$) δ = 208.4, 133.9, 132.9, 128.8, 127.2, 102.5, 80.1, 33.0, 30.1.

HRMS (+ p APCI) m/z: $[M+H]^+$ Calcd for $C_{11}H_{10}{}^{35}Cl^{79}Br^+$: 258.9705; Found: 258.9706.

1-(5-bromopenta-1,2-dien-1-yl)-4-chlorobenzene (1i')

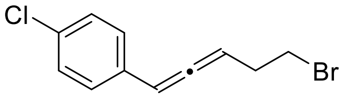

1f'

Following the **General Procedure A**, after concentration, the crude material was purified by flash column chromatography (SiO_2, Pentane) to furnish the title compound as a colorless oil (320 mg, 25% yield, 3 steps).

R*f* = 0.38 (pentane).

^{1}**H NMR** (400 MHz, $CDCl_3$) δ = 7.30 – 7.21 (m, 4H), 6.17 (dtd, *J* = 6.7, 2.9, 1.0 Hz, 1H), 5.62 (qd, *J* = 6.2, 0.9 Hz, 1H), 3.67 – 3.44 (m, 2H), 2.76 – 2.54 (m, 2H).

^{13}**C NMR** (101 MHz, $CDCl_3$) δ = 206.0, 132.8, 128.9, 128.1, 128.1, 95.1, 93.0, 32.1, 31.7.

HRMS (+ p APCI) m/z: $[M+H]^+$ Calcd for $C_{11}H_{10}{}^{35}Cl^{79}Br^+$: 258.9705; Found: 258.9705.

1-(5-bromopenta-1,2-dien-3-yl)-4-(trifluoromethyl)benzene (1j)

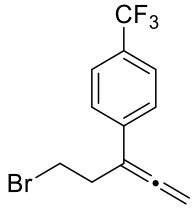

1j

Following the **General Procedure A**, after concentration, the crude material was purified by flash column chromatography (SiO_2, Pentane) to furnish the title compound as a colorless oil (231 mg, 16% yield, 3 steps).

R*f* = 0.40 (pentane).

^{1}H NMR (400 MHz, $CDCl_3$) δ = 7.60 – 7.56 (m, 2H), 7.49 – 7.45 (m, 2H), 5.25 (tq, *J* = 3.3, 0.6 Hz, 2H), 3.55 (dd, *J* = 7.6, 7.2 Hz, 2H), 3.00 (tt, *J* = 7.3, 3.2 Hz, 2H).

^{19}F NMR (471 MHz, $CDCl_3$) δ = -62.51.

^{13}C NMR (101 MHz, $CDCl_3$) δ = 209.0, 139.3 (q, *J* = 1.5 Hz), 129.1 (q, *J* = 32.4 Hz), 126.1, 125.6 (q, *J* = 3.8 Hz), 121.6 (q, *J* = 271.8 Hz), 102.6, 80.4, 32.9, 29.9.

HRMS (+ p APCI) m/z: $[M+H]^+$ Calcd for $C_{12}H_{10}F_3{}^{79}Br^+$: 290.9991; Found: 290.9991.

1-(5-bromopenta-1,2-dien-1-yl)- 4-(trifluoromethyl)benzene (1j')

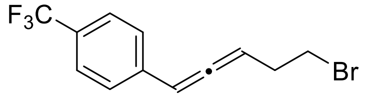

1j'

Following the **General Procedure A**, after concentration, the crude material was purified by flash column chromatography (SiO_2, Pentane) to furnish the title compound as a colorless oil (231 mg, 16% yield, 3 steps).

R*f* = 0.38 (pentane).

^{1}H NMR (500 MHz, $CDCl_3$) δ = 7.58 – 7.53 (m, 2H), 7.43 – 7.38 (m, 2H), 6.25 (dt, *J* = 6.1, 2.9 Hz, 1H), 5.67 (q, *J* = 6.6 Hz, 1H), 3.50 (td, *J* = 6.8, 1.3 Hz, 2H), 2.78 – 2.66 (m, 2H).

^{19}F NMR (471 MHz, $CDCl_3$) δ = -62.45.

^{13}C NMR (126 MHz, $CDCl_3$) δ = 206.7, 138.2 (d, *J* = 1.2 Hz), 129.0 (q, *J* = 32.3 Hz), 127.1, 125.6 (q, *J* = 3.8 Hz), 124.3 (q, *J* = 271.7 Hz), 95.2, 93.2, 32.0, 31.6.

HRMS (+ p APCI) m/z: $[M+H]^+$ Calcd for $C_{12}H_{10}F_3{}^{79}Br^+$: 290.9991; Found: 290.9990.

1-(5-bromopenta-1,2-dien-3-yl)-4-(trifluoromethoxy)benzene (1k)

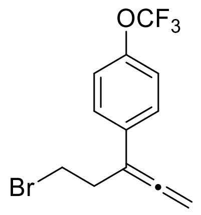

1k

Following the **General Procedure A**, after concentration, the crude material was purified by flash column chromatography (SiO_2, Pentane) to furnish the title compound as a colorless oil (273 mg, 18% yield, 3 steps).

R*f* = 0.40 (pentane).

^{1}H NMR (500 MHz, $CDCl_3$) δ = 7.41 – 7.36 (m, 2H), 7.21 – 7.15 (m, 2H), 5.21 (t, *J* = 3.3 Hz, 2H), 3.54 (t, *J* = 7.4 Hz, 2H), 2.97 (tt, *J* = 7.3, 3.2 Hz, 2H).

^{19}F NMR (471 MHz, $CDCl_3$) δ = -57.90.

^{13}C NMR (126 MHz, $CDCl_3$) δ = 208.5, 148.2, 134.2, 127.2, 120.6 (q, *J* = 257.2 Hz), 121.2, 102.4, 80.1, 33.0, 30.1.

HRMS (+ p APCI) m/z: $[M+H]^+$ Calcd for $C_{12}H_{10}OF_3{}^{79}Br^+$: 306.9940; Found: 306.9939.

1-(5-bromopenta-1,2-dien-1-yl)-4-(trifluoromethoxy)benzene (1k')

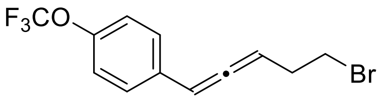

1k'

Following the **General Procedure A**, after concentration, the crude material was purified by flash column chromatography (SiO_2, Pentane) to furnish the title compound as a colorless oil (427 mg, 28% yield, 3 steps).

R*f* = 0.38 (pentane).

^{1}H NMR (500 MHz, $CDCl_3$) δ = 7.33 (dq, *J* = 8.7, 2.3, 1.7 Hz, 2H), 7.15 (dq, *J* = 8.7, 1.0 Hz, 2H), 6.20 (dt, *J* = 6.5, 2.8 Hz, 1H), 5.64 (qd, *J* = 6.6, 1.5 Hz, 1H), 3.56 (dtd, *J* = 74.8, 6.8, 1.3 Hz, 2H), 2.78 – 2.56 (m, 2H).

^{19}F NMR (471 MHz, $CDCl_3$) δ = -57.91.

^{13}C NMR (126 MHz, $CDCl_3$) δ = 206.1, 148.2, 133.1, 128.1, 121.3, 120.6 (q, *J* = 256.9 Hz), 94.9, 93.0, 32.1, 31.7.

HRMS (+ p APCI) m/z: $[M+H]^+$ Calcd for $C_{12}H_{10}OF_3{}^{79}Br^+$: 306.9940; Found: 306.9930.

1-(5-bromopenta-1,2-dien-3-yl)-3-methylbenzene (1l)

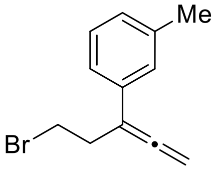

1l

Following the **General Procedure A**, after concentration, the crude material was purified by flash column chromatography (SiO_2, Pentane) to furnish the title compound as a colorless oil (238 mg, 20% yield, 3 steps).

R*f* = 0.40 (pentane).

^{1}H NMR (400 MHz, $CDCl_3$) δ = 7.25 – 7.15 (m, 3H), 7.05 (dddt, *J* = 7.3, 1.8, 1.3, 0.6 Hz, 1H), 5.16 (td, *J* = 3.2, 0.5 Hz, 2H), 3.59 – 3.47 (m, 2H), 2.98 (ddt, *J* = 8.1, 7.4, 3.2 Hz, 2H), 2.36 (s, 3H).

^{13}C NMR (101 MHz, $CDCl_3$) δ = 208.5, 138.3, 135.2, 128.6, 128.0, 126.7, 123.0, 103.3, 79.5, 33.2, 30.5, 21.6.

HRMS (+ p APCI) m/z: $[M+H]^+$ Calcd for $C_{12}H_{13}{}^{81}Br^+$: 239.0254; Found: 239.0254.

1-(5-bromopenta-1,2-dien-3-yl)-3-methoxybenzene (1m)

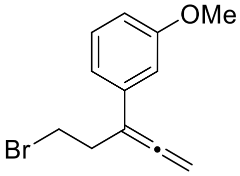

1m

Following the **General Procedure A**, after concentration, the crude material was purified by flash column chromatography (SiO_2, Pentane/Et_2O – 10:1) to furnish the title compound as a colorless oil (152 mg, 12% yield, 3 steps).

R*f* = 0.45 (pentane /Et_2O – 10:1).

^{1}H NMR (400 MHz, $CDCl_3$) δ = 7.25 (ddd, *J* = 8.2, 7.7, 0.4 Hz, 1H), 6.97 (dddd, *J* = 7.7, 2.2, 0.9, 0.5 Hz, 1H), 6.93 (ddd, *J* = 2.6, 1.7, 0.5 Hz, 1H), 6.80 – 6.76 (m, 1H), 5.17 (ddt, *J* = 3.2, 2.7, 0.5 Hz, 2H), 3.82 (s, 3H), 3.71 – 3.51 (m, 2H), 3.01 – 2.85 (m, 2H).

^{13}C NMR (101 MHz, $CDCl_3$) δ = 208.6, 160.0, 136.9, 129.6, 118.4, 112.5, 112.0, 103.2, 79.7, 55.3, 33.2, 30.4.

HRMS (+ p APCI) m/z: $[M+H]^+$ Calcd for $C_{12}H_{13}O^{81}Br^+$: 253.0223; Found: 253.0226.

1-(5-bromopenta-1,2-dien-1-yl)-3-methoxybenzene (1m')

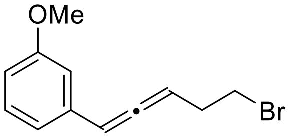

1m'

Following the **General Procedure A**, after concentration, the crude material was purified by flash column chromatography (SiO_2, Pentane/Et_2O – 10:1) to furnish the title compound as a colorless oil (252mg, 20% yield, 3 steps).

R*f* = 0.43 (pentane/Et_2O – 10:1).

^{1}H NMR (400 MHz, $CDCl_3$) δ = 7.24 – 7.19 (m, 1H), 6.91 – 6.87 (m, 2H), 6.78 – 6.75 (m, 1H), 6.19 (ddd, *J* = 6.4, 3.2, 2.5 Hz, 1H), 5.61 (q, *J* = 6.6 Hz, 1H), 3.81 (s, 3H), 3.57 (dtd, *J* = 59.2, 6.8, 1.5 Hz, 2H), 2.74 – 2.55 (m, 2H).

^{13}C NMR (101 MHz, $CDCl_3$) δ = 206.0, 160.0, 135.7, 129.7, 119.6, 113.1, 112.1, 96.0, 92.7, 55.3, 32.2, 31.9.

HRMS (+ p APCI) m/z: $[M+H]^+$ Calcd for $C_{12}H_{13}O^{81}Br^+$: 253.0223; Found: 253.0227.

Note: ortho-Toluene substituted Grignard regent was subject to the Cupper-catalyzed S_N addition reaction, both of the silyl-protected allene and the silyl-protected alkyne could be separated by column chromatography.

tert-butyldimethyl((3-(o-tolyl)penta-3,4-dien-1-yl)oxy)silane (S7n)

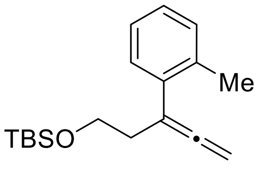

S7n

Following the **General Procedure A**, after concentration, the crude material was purified by flash column chromatography (SiO_2, Pentane) to furnish the title compound as a colorless oil (432 mg, 30% yield).

R*f* = 0.40 (pentane).

^{1}H NMR (700 MHz, $CDCl_3$) δ = 7.21 – 7.13 (m, 4H), 4.78 (t, *J* = 3.0 Hz, 2H), 3.71 (t, *J* = 7.1 Hz, 2H), 2.54 (tt, *J* = 7.0, 3.0 Hz, 2H), 2.34 (s, 3H), 0.88 (s, 9H), 0.03 (s, 6H).

^{13}C NMR (176 MHz, $CDCl_3$) δ = 207.3, 137.1, 136.2, 130.5, 128., 127.0, 125.9, 100.8, 75.3, 61.5, 36.9, 26.0, -5.2.

HRMS (+ p APCI) m/z: $[M+H]^+$ Calcd for $C_{18}H_{28}OSi^+$: 289.1982; Found: 289.1984.

tert-butyldimethyl((5-(o-tolyl)pent-3-yn-1-yl)oxy)silane (S7n')

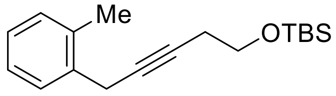

S7n'

Following the **General Procedure A**, after concentration, the crude material was purified by flash column chromatography (SiO_2, Pentane) to furnish the title compound as a colorless oil (648 mg, 45% yield).

R*f* = 0.38 (pentane).

^{1}H NMR (700 MHz, $CDCl_3$) δ = 7.43 (ddt, *J* = 6.6, 1.4, 0.7 Hz, 1H), 7.19 – 7.12 (m, 3H), 3.73 (t, *J* = 7.2 Hz, 2H), 3.50 – 3.47 (m, 2H), 2.43 (tt, *J* = 7.1, 2.4 Hz, 3H), 2.31 (s, 3H), 0.90 (s, 9H), 0.07 (s, 6H).

^{13}C NMR (176 MHz, $CDCl_3$) δ = 136.0, 135.6, 130.1, 128.3, 126.8, 126.2, 79.6, 78.5, 65.9, 62.4, 26.0, 23.4, 23.4, 19.3, -5.2.

HRMS (+ p APCI) m/z: $[M+H]^+$ Calcd for $C_{18}H_{28}OSi^+$: 289.1982; Found: 289.1983.

3-(o-tolyl)penta-3,4-dien-1-ol (S8n)

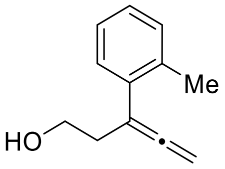

S8n

Following the **General Procedure A**, from **S7n**, after concentration, the crude material was purified by flash column chromatography (SiO_2, Pentane/Et_2O – 3:2) to furnish the title compound as a colorless oil (230 mg, 90% yield).

R*f* = 0.30 (pentane/Et_2O – 3:2).

^{1}H NMR (500 MHz, $CDCl_3$) δ = 7.22 – 7.14 (m, 4H), 4.87 (t, *J* = 3.2 Hz, 2H), 3.78 (q, *J* = 6.1 Hz, 2H), 2.59 (tt, *J* = 6.3, 3.2 Hz, 2H), 2.35 (s, 3H).

^{13}C NMR (126 MHz, $CDCl_3$) δ = 206.8, 136.7, 136.1, 130.7, 128.1, 127.3, 126.1, 100.9, 76.0, 61.0, 36.9, 20.3.

HRMS (+ p APCI) m/z: $[M+H]^+$ Calcd for $C_{12}H_{15}O^+$: 175.1117; Found: 175.1116.

1-(5-bromopenta-1,2-dien-3-yl)-2-methylbenzene (1n)

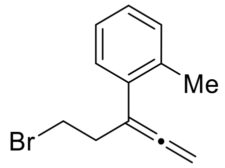

1n

Following the **General Procedure A**, from **S8n**, after concentration, the crude material was purified by flash column chromatography (SiO_2, Pentane) to furnish the title compound as a colorless oil (201mg, 66% yield).

R*f* = 0.40 (pentane).

^{1}H NMR (500 MHz, $CDCl_3$) δ = 7.22 – 7.15 (m, 4H), 4.89 (t, *J* = 3.1 Hz, 2H), 3.46 (t, *J* = 7.2 Hz, 2H), 2.88 (tt, *J* = 7.3, 3.1 Hz, 2H), 2.36 (s, 3H).

^{13}C NMR (126 MHz, $CDCl_3$) δ = 207.1, 136.4, 135.9, 130.8, 128.0, 127.4, 126.1, 101.6, 76.7, 36.8, 30.6, 20.3.

HRMS (+ p APCI) m/z: $[M+H]^+$ Calcd for $C_{12}H_{13}{}^{79}Br^+$: 237.0273; Found: 237.0278.

2-(5-bromopenta-1,2-dien-3-yl)naphthalene (1o)

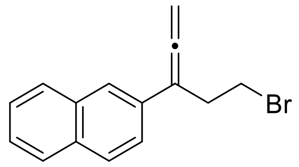

1o

Following the **General Procedure A**, after concentration, the crude material was purified by flash column chromatography (SiO_2, Pentane) to furnish the title compound as a colorless oil (340 mg, 25% yield, 3 steps).

R*f* = 0.40 (pentane).

^{1}H NMR (500 MHz, $CDCl_3$) δ = 7.83 – 7.77 (m, 3H), 7.72 (dt, *J* = 2.0, 0.7 Hz, 1H), 7.59 (dd, *J* = 8.6, 1.9 Hz, 1H), 7.46 (pd, *J* = 6.9, 1.7 Hz, 2H), 5.27 (td, *J* = 3.2, 0.8 Hz, 2H), 3.62 (dd, *J* = 7.8, 7.2 Hz, 2H), 3.12 (tt, *J* = 7.4, 3.1 Hz, 2H).

^{13}C NMR (126 MHz, $CDCl_3$) δ = 209.1, 133.6, 132.6, 132.6, 128.2, 128.1, 127.7, 126.4, 126.0, 125.0, 123.5, 103.5, 80.1, 33.1, 30.5.

HRMS (+ p APCI) m/z: $[M+H]^+$ Calcd for $C_{15}H_{13}{}^{79}Br^+$: 273.0273; Found: 273.0273.

2-(5-bromopenta-1,2-dien-1-yl)naphthalene (1o')

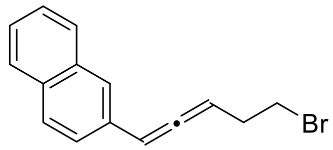

1o'

Following the **General Procedure A**, after concentration, the crude material was purified by flash column chromatography (SiO_2, Pentane) to furnish the title compound as a colorless oil (435 mg, 32% yield, 3 steps).

R*f* = 0.40 (pentane).

^{1}H NMR (500 MHz, $CDCl_3$) δ = 7.79 (tdd, *J* = 8.2, 2.6, 1.5 Hz, 3H), 7.68 (dd, *J* = 1.8, 0.8 Hz, 1H), 7.52 (dd, *J* = 8.5, 1.8 Hz, 1H), 7.48 – 7.40 (m, 2H), 6.40 (dt, *J* = 6.1, 2.9 Hz, 1H), 5.69 (qd, *J* = 6.6, 0.7 Hz, 1H), 3.53 (td, *J* = 6.9, 1.0 Hz, 2H), 2.75 (qt, *J* = 7.0, 2.9 Hz, 2H).

^{13}C NMR (126 MHz, $CDCl_3$) δ = 206.4, 133.8, 132.8, 131.7, 128.3, 127.8, 127.8, 126.3, 125.8, 125.8, 124.9, 96.3, 92.8, 32.3, 31.9.

HRMS (+ p APCI) m/z: $[M+H]^+$ Calcd for $C_{15}H_{13}{}^{79}Br^+$: 273.0273; Found: 273.0276.

3-(5-bromopenta-1,2-dien-3-yl)thiophene (1p)

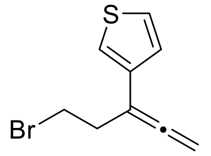

1p

Following the **General Procedure A**, after concentration, the crude material was purified by flash column chromatography (SiO_2, Pentane) to furnish the title compound as a colorless oil (340 mg, 11% yield, 3 steps).

R*f* = 0.40 (pentane).

^{1}H NMR (400 MHz, $CDCl_3$) δ = 7.28 (dd, *J* = 5.1, 2.9 Hz, 1H), 7.11 (dd, *J* = 5.1, 1.4 Hz, 1H), 7.07 (ddt, *J* = 3.0, 1.3, 0.9 Hz, 1H), 5.15 (td, *J* = 3.1, 0.9 Hz, 2H), 3.56 (dd, *J* = 7.8, 7.2 Hz, 2H), 2.95 (ddt, *J* = 7.9, 7.3, 3.2 Hz, 2H).

^{13}C NMR (101 MHz, $CDCl_3$) δ = 208.6, 137.1, 126.7, 125.9, 119.1, 99.7, 79.5, 33.9, 30.2.

HRMS (+ p APCI) m/z: $[M+H]^+$ Calcd for $C_9H_9S^{81}Br^+$: 230.9660; Found: 230.9659.

Note: Preparation of 3-Thiophenylmagnesium Bromide:[4]

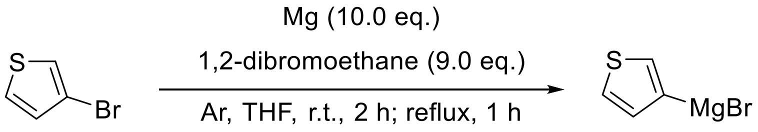

S9 **S10**

A 100 mL 3-neck round-bottomed flask was charged with Mg (1.440 g, 60 mmol) and a stir bar, a condenser was attached and the flask was sealed with a septum. The flask was evacuated and heated with a heat gun for 5 min, cooled to r.t. and refilled with argon. Dry anhydride THF (6 mL) was added. In a separate flask 1,2-dibromoethane (4.66 mL, 54 mmol) was dissolved in ether (20 mL) under argon. A portion of this solution (approx. 3 mL) was added to the flask with Mg slowly until the solvent started boiling. Then, 3-bromothiophene (0.56 mL, 6 mmol) was added to the rest of the solution of 1,2-dibromoethane in ether and the resulting mixture was added slowly to the reaction flask in such a rate the solvent was slowly boiling (over approx. 2 h). Upon addition, the solution turned dark brown. When the addition had ended, the flask was placed into an oil bath (T = 60 °C) for 1 h. After cooling to room temperature, the fresh thiophenyl Grignard regent was subjected to the cupper-catalyzed S_N addition reaction.

(6-bromohexa-1,2-dien-3-yl)benzene (1q)

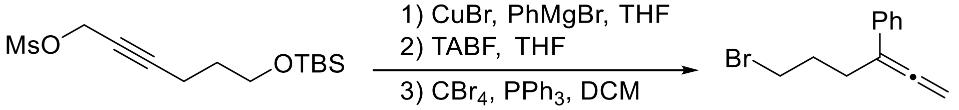

S11 **1q**

Following the **General Procedure A**, from **S11**, after concentration, the crude material was purified by flash column chromatography (SiO_2, Pentane) to furnish the title compound as a colorless oil (189 mg, 16% yield, 3 steps).

R*f* = 0.40 (pentane).

^{1}H NMR (400 MHz, $CDCl_3$) δ = 7.43 – 7.39 (m, 2H), 7.36 – 7.30 (m, 2H), 7.24 – 7.19 (m, 1H), 5.12 (td, *J* = 3.3, 0.5 Hz, 2H), 3.51 (t, *J* = 6.6 Hz, 2H), 2.64 – 2.55 (m, 2H), 2.18 – 2.05 (m, 2H).

^{13}C NMR (101 MHz, $CDCl_3$) δ = 208.5, 136.0, 128.6, 126.9, 126.0, 103.8, 78.9, 33.4, 31.0, 28.0.

HRMS (+ p APCI) m/z: $[M+H]^+$ Calcd for $C_{12}H_{13}{}^{79}Br^+$: 237.0273; Found: 237.0276.

Preparation of alkyl substituted allenes:

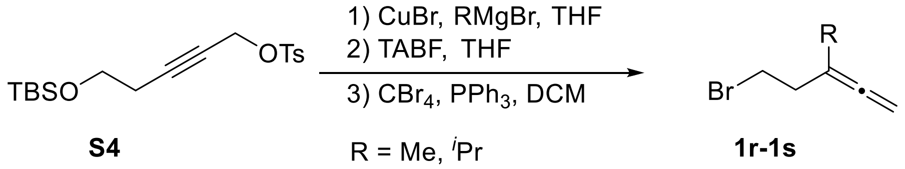

5-bromo-3-methylpenta-1,2-diene (1r)

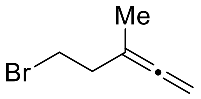

1r

Following the **General Procedure A**, from **S4** and methylmagnesium bromide, after concentration, the crude material was purified by flash column chromatography (SiO_2, Pentane) to furnish the title compound as a colorless oil (97 mg, 12% yield, 3 steps). Analytical data are in accordance with literature.[5]

R*f* = 0.50 (pentane).

^{1}H NMR (400 MHz, $CDCl_3$) δ = 4.57 (h, *J* = 3.1 Hz, 2H), 3.79 – 3.58 (m, 2H), 2.17 (ddtd, *J* = 7.4, 6.9, 2.9, 0.5 Hz, 1H), 1.70 (s, 3H).

^{13}C NMR (101 MHz, $CDCl_3$) δ = 206.7, 95.5, 73.9, 36.9, 26.0, 22.4.

HRMS (+ p APCI) m/z: $[M+H]^+$ Calcd for $C_6H_{10}{}^{79}Br^+$: 160.9960; Found: 160.9959.

tert-butyl((3-isopropylpenta-3,4-dien-1-yl)oxy)dimethylsilane (S12s)

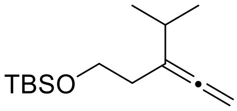

S12s

Following the **General Procedure A**, from **S4** and isopropylmagnesium bromide, after concentration, the crude material was purified by flash column chromatography (SiO_2, Pentane) to furnish the title compound as a colorless oil (624 mg, 52% yield).

R*f* = 0.50 (pentane).

^{1}H NMR (500 MHz, $CDCl_3$) δ = 4.67 (q, *J* = 3.0 Hz, 2H), 3.70 – 3.67 (m, 2H), 2.21 – 2.16 (m, 2H), 2.15 – 2.07 (m, 1H), 1.02 (d, *J* = 6.8 Hz, 6H), 0.89 (s, 9H), 0.06 (s, 6H).

^{13}C NMR (126 MHz, $CDCl_3$) δ = 204.9, 106.3, 76.7, 62.3, 33.5, 30.7, 26.0, 21.6, 18.4, -5.2.

HRMS (+ p APCI) m/z: $[M+H]^+$ Calcd for $C_{14}H_{28}OSi^+$: 241.1984; Found: 241.1984.

5-bromo-3-isopropylpenta-1,2-diene (1s)

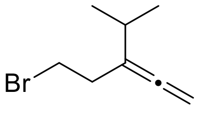

1s

Following the **General Procedure A**, from **S12s**, after concentration, the crude material was purified by flash column chromatography (SiO_2, Pentane) to furnish the title compound as a colorless oil (240 mg, 48% yield, 2 steps).

$\mathbf{R_f}$ = 0.50 (pentane).

^{1}H NMR (400 MHz, $CDCl_3$) δ = 4.79 (td, J = 3.3, 2.6 Hz, 21H), 3.43 (dd, J = 8.0, 7.4 Hz, 2H), 2.57 – 2.38 (m, 2H), 1.03 (d, J = 6.8 Hz, 6H).

^{13}C NMR (101 MHz, $CDCl_3$) δ = 207.8, 105.5, 76.9, 34.7, 30.7, 28.4, 21.2.

HRMS (+ p APCI) m/z: $[M+H]^+$ Calcd for $C_8H_{14}{}^{79}Br^+$: 189.0273; Found: 189.0272.

Preparation of ((5-bromopenta-1,2-dien-3-yl)sulfinyl)benzene (1t)

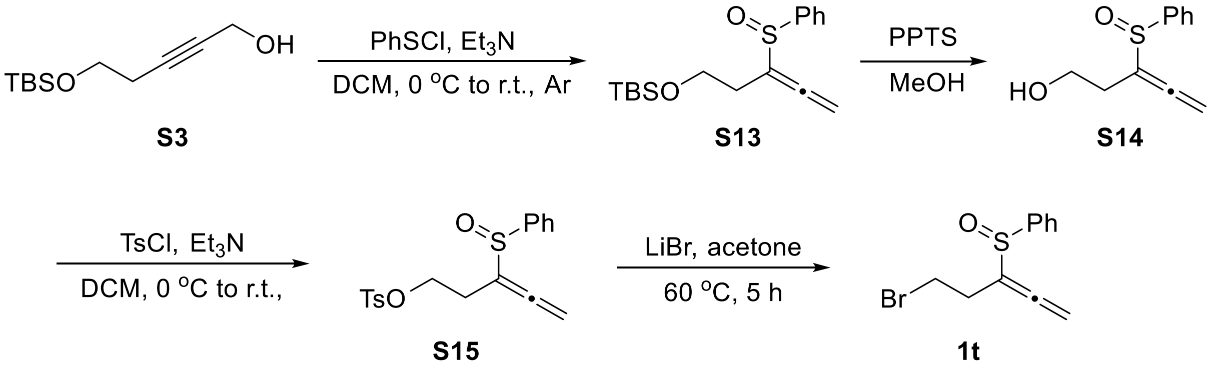

Preparation of Phenylsulfenyl chloride, PhSCl: An oven-dried two-neched flask was charged with NCS (2.323 g, 17.4 mmol, 1.25 equiv.) and dry anhydrous DCM (51 mL) under an argon atmosphere and cooled to 0 °C. Thiophenol (1.4 mL, 13.92 mmol, 1.0 equiv.) was added slowly to the reaction mixture. A color changes to deep orange was observed after 10 min. The reaction mixture was stirred for additional 30 min at the same temperature. Then, the solvent was removed under reduced pressure (bath temp.: 42°C, 550 mbar). n-Pentane (10 mL) was added to the flask, a white precipitate was formed. The reaction mixture was filtered through a pad of Celite, and washed with n-pentane (3 x 10 mL). The filtrate was concentrated under reduced pressure (bath temp.: 42°C, 550 mbar) giving the fresh phenylsulfenyl chloride as an orange oil with quantity yield, which used in next step without further purification.

tert-butyldimethyl((3-(phenylsulfinyl)penta-3,4-dien-1-yl)oxy)silane (S13):

An oven-dried two-neched flask was charged with **S3** (3.680 g, 10 mmol, 1.0 equiv.) and dry anhydrous DCM (33 mL) under an argon atmosphere and cooled to 0 °C. Et_3N (1.54 mL, 10 mmol, 1.3 equiv.) was added to the solution and stirring for 15 min. Then, fresh prepared phenylsulfenyl chloride (13 mmol, 1.3 equiv.) diluted in dry anhydrous DCM (6 mL) was added dropwise at the same temperature. The reaction mixture was allowed to warm to room temperature and stirred for additional 3 hours. Then, the reaction was quenched with $NaHCO_3$ solution and extracted with DCM (3 x 20 mL). The combined organic phases were washed with brine and dried over Na_2SO_4. The solvents were removed under reduced pressure and the crude product was purified by column chromatography on silica gel (Pentane/EtOAc – 6:1) to give the allenyl sulfoxide **S13** (2.870 g, 89% yield) as a yellowish oil. The analytical data is in accordance with reported literature values.[6]

R_f = 0.50 (pentane/EtOAc – 5:1).

^{1}H NMR (400 MHz, $CDCl_3$) δ = 7.92 – 7.88 (m, 2H), 7.65 – 7.59 (m, 1H), 7.56 – 7.50 (m, 2H), 5.35 (t, J = 3.0 Hz, 2H), 3.67 (t, J = 6.7 Hz, 2H), 2.46 (tt, J = 6.7, 3.0 Hz, 2H), 0.83 (s, 9H), -0.01 (s, 6H).

^{13}C NMR (101 MHz, $CDCl_3$) δ = 208.8, 140.4, 133.5, 129.1, 128.2, 110.1, 84.0, 60.8, 30.6, 25.9, 18.2, -5.3.

3-(phenylsulfinyl)penta-3,4-dien-1-ol (S14):

To a solution of allenyl sulfoxide **S13** (1.612 g, 5 mmol, 1.0 equiv.) in MeOH (50 mL) at 0 °C was added PPTS (126 mg, 0.5 mmol, 0.1 equiv.). The solution was allowed to warm to room temperature and stirred for 5 h. The solvent was removed under reduced pressure. Water (10 mL) was added and extracted with EtOAc (3 x 15 mL). The combined organic phases were washed with brine and dried over Na_2SO_4. The solvents were removed under reduced pressure and the crude product was purified by column chromatography on silica gel (Pentane/EtOAc – 1:1) to give the allenyl sulfoxide **S14** (707 mg, 68% yield) as a colorless oil. The analytical data is in accordance with reported literature values.

R_f = 0.15 (pentane/EtOAc – 3:2).

^{1}H NMR (700 MHz, $CDCl_3$) δ = 7.63 – 7.60 (m, 2H), 7.54 – 7.48 (m, 3H), 5.33 (dt, J = 12.9, 2.3 Hz, 1H), 5.26 (dt, J = 12.7, 2.4 Hz, 1H), 3.73 – 3.54 (m, 2H), 3.25 (brs, 1H), 2.35 (dddt, J = 15.3, 5.5, 3.8, 2.3 Hz, 1H), 2.24 (dddt, J = 15.3, 8.5, 4.6, 2.4 Hz, 1H).

^{13}C NMR (176 MHz, $CDCl_3$) δ = 207.9, 142.3, 131.2, 129.3, 124.7, 111.0, 81.2, 61.1, 29.7.

3-(phenylsulfinyl)penta-3,4-dien-1-yl 4-methylbenzenesulfonate (S15):

To a solution of alcohol allene **S14** (625 mg, 3 mmol, 1.0 equiv.) in DCM (15 mL) at 0 °C was added Et_3N (0.50 mL, 3.6 mmol, 1.2 equiv.), and the solution was stirred for 5 min. Then, TsCl (630 mg, 3.3 mmol, 1.1 equiv.) was added, and DMAP, 4-dimethylaminopyridine (*cat.* amount) was added subsequently. The solution was allowed to warm to room temperature and stirred for additional 5 hours. The reaction mixture was quenched by water (15 mL). The aqueous phase was extracted with DCM (3 × 15 mL). The combined organic phases were washed with brine and dried over Na_2SO_4. The solvents were removed under reduced pressure and the crude product was purified by flash column chromatography on silica gel (Pentanes/EtOAc – 1:1) to give the desired product **S15** (565 mg, 52% yield) as a colorless oil.

R*f* = 0.30 (pentane/EtOAc – 1:1).

^{1}H NMR (700 MHz, $CDCl_3$) δ = 7.72 – 7.69 (m, 2H), 7.57 – 7.55 (m, 2H), 7.51 – 7.48 (m, 3H), 7.33 – 7.31 (m, 2H), 5.30 (dt, *J* = 12.7, 3.1 Hz, 1H), 5.24 (dt, *J* = 12.7, 3.0 Hz, 1H), 3.99 (dd, *J* = 6.9, 6.5 Hz, 2H), 2.51 (dtt, *J* = 15.9, 6.5, 3.0 Hz, 1H), 2.45 (s, 3H), 2.25 (dtt, *J* = 15.9, 7.0, 3.1 Hz, 1H).

^{13}C NMR (176 MHz, $CDCl_3$) δ = 205.9, 145.0, 143.0, 133.0, 131.2, 129.9, 129.3, 128.0, 124.4, 108.2, 83.0, 67.7, 23.0, 21.7.

HRMS (+ p ESI) m/z: $[M+H]^+$ Calcd for $C_{18}H_{18}O_4S_2^+$: 363.0723; Found: 363.0723.

((5-bromopenta-1,2-dien-3-yl)sulfinyl)benzene (1t):

To a solution of tosylate allene S**15** (550 mg, 1.5 mmol, 1.0 equiv.) in acetone (12 mL, 0.12 M) was added LiBr (653 mg, 7.5 mmol, 5.0 equiv.). Then, the solution was stirred at 60 °C for 12 hours. The solvents were removed under reduced pressure and the crude product was purified by flash column chromatography on silica gel (Pentanes/Et_2O – 1:1) to give the desired product **1t** (260 mg, 65% yield) as a colorless oil.

R*f* = 0.45 (pentane/Et_2O – 1:1).

^{1}H NMR (700 MHz, $CDCl_3$) δ = 7.40 – 7.31 (m, 4H), 7.25 – 7.20 (m, 1H), 5.17 (td, *J* = 3.2, 0.5 Hz, 2H), 3.55 (d, *J* = 7.6 Hz, 2H), 2.99 (ddt, *J* = 8.0, 7.4, 3.2 Hz, 2H).

^{13}C NMR (176 MHz, $CDCl_3$) δ = 206.0, 143.2, 131.2, 129.3, 124.5, 110.6, 83.2, 29.4, 26.8.

HRMS (+ p APCI) m/z: $[M+H]^+$ Calcd for $C_{11}H_{11}OS^{79}Br^+$: 270.9788; Found: 270.9788.

Preparation of Internal Allenyl Esters:

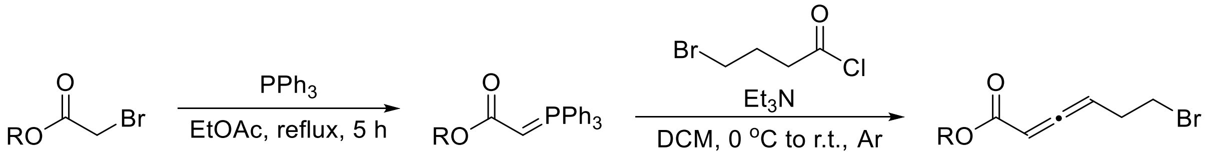

S16 **S17** **1u-1v**

General Procedure B:

Step 1. To an oven dried two necked flask was charged PPh_3 (1.578 g, 6.0 mmol, 1.0 equiv.) and dry anhydride EtOAc (12 mL, 0.5 M) under argon atmosphere, 2-bromoacetate (6.3 mmol, 1.05 equiv.) was added slowly. Then, the reaction mixture was refluxed for 8 h and cooled to r.t. The precipitate was filtered, washed with hexane (2 × 20 mL) and the solid dried under vacuum to give phosphonium salt as a fine white powder. The freshly made phosphonium salt was dissolved in DCM (40 mL) and washed with aq. KOH (1.0 g, 18 mmol in 40 mL). The phases were separated and the organic phase dried (Na_2SO_4) to give phosphorane **S17** as a white solid (quant.). All spectra data was in accord with that reported.[7]

Step 2.

Preparation of acyl chloride: To a solution of bromoacetic acid (1.0 g, 6 mmol, 1.0 equiv.) in dry anhydride DCM (18 mL) was added oxalyl chloride (0.57 mL, 7.2 mmol, 1.2 equiv.) and DMF (50 µL) at 0 °C. Then, the reaction mixture was allowed to warm to room temperature and stir for an additional 8 h. The solvent was removed under reduced pressure and the residue used in next step without further purification.

To an oven-dried two-necked flask was charged triphenylphosphorane **S17** (5 mmol, 1.0 equiv.) and dry anhydrous DCM (15 mL) under an argon atmosphere and cooled to 0 °C. Et_3N (1.0 mL, 7.5 mmol, 1.5 equiv.) and 4-bromobutanoyl chloride (6 mmol, 1.2 equiv.) was added subsequently. Then, the reaction mixture was allowed to warm to room temperature and stirred for 2 h. The solvent was removed under reduced pressure (35 °C, 500 mbar), and pentane (30 mL) was added. Then, the flask was put into ultrasonic for 20 min, white precipitate was formed and was filtered through a pad of Celite. The filtrate was concentrated under reduced pressure (35 °C, 500 mbar) and the crude product was purified by flash column chromatography on silica gel (Pentanes/Et_2O – 20:1) to give the desired product **1u** (260 mg, 65% yield) as a colorless oil.

ethyl 6-bromohexa-2,3-dienoate (1u)

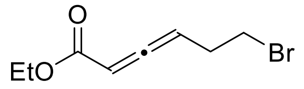

1u

Following the **General Procedure B**, from **S16u**, after concentration, the crude material was purified by flash column chromatography (SiO_2, Pentane/Et_2O – 20:1) to furnish the title compound as a colorless oil (596 mg, 55% yield, 2 steps).

R*f* = 0.35 (pentane/Et_2O – 20:1).

^{1}H NMR (500 MHz, $CDCl_3$) δ = 5.75 – 5.56 (m, 2H), 4.19 (qd, *J* = 7.2, 0.8 Hz, 2H), 3.45 (t, *J* = 6.9 Hz, 2H), 2.72 – 2.67 (m, 2H), 1.28 (t, *J* = 7.1 Hz, 3H).

^{13}C NMR (126 MHz, $CDCl_3$) δ = 212.4, 165.7, 93.0, 89.5, 61.1, 30.96, 30.92, 14.3.

HRMS (+ p ESI) m/z: $[M+H]^+$ Calcd for $C_8H_{11}O_2{}^{79}Br^+$: 219.0017; Found: 219.0017.

benzyl 6-bromohexa-2,3-dienoate (1v)

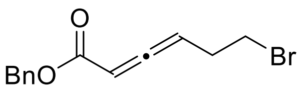

1v

Following the **General Procedure B**, from **S16v**, after concentration, the crude material was purified by flash column chromatography (SiO_2, Pentane/Et_2O – 20:1) to furnish the title compound as a colorless oil (474 mg, 34% yield, 2 steps).

R*f* = 0.35 (pentane/Et_2O – 20:1).

^{1}H NMR (500 MHz, $CDCl_3$) δ = 7.39 – 7.27 (m, 3H), 7.23 – 7.19 (m, 2H), 5.53 (tt, *J* = 7.0, 2.5 Hz, 1H), 5.18 (d, *J* = 3.8 Hz, 1H), 5.14 (s, 2H), 3.66 – 3.54 (m, 2H), 2.60 – 2.52 (m, 2H).

^{13}C NMR (126 MHz, $CDCl_3$) δ = 211.2, 166.7, 139.0, 129.0, 128.4, 128.0, 126.5, 102.0, 93.3, 66.8, 31.4, 31.1.

HRMS (+ p APCI) m/z: $[M+H]^+$ Calcd for $C_{13}H_{13}O_2{}^{79}Br^+$: 281.0174; Found: 281.0172.

Preparation of Terminal Allenyl Ester

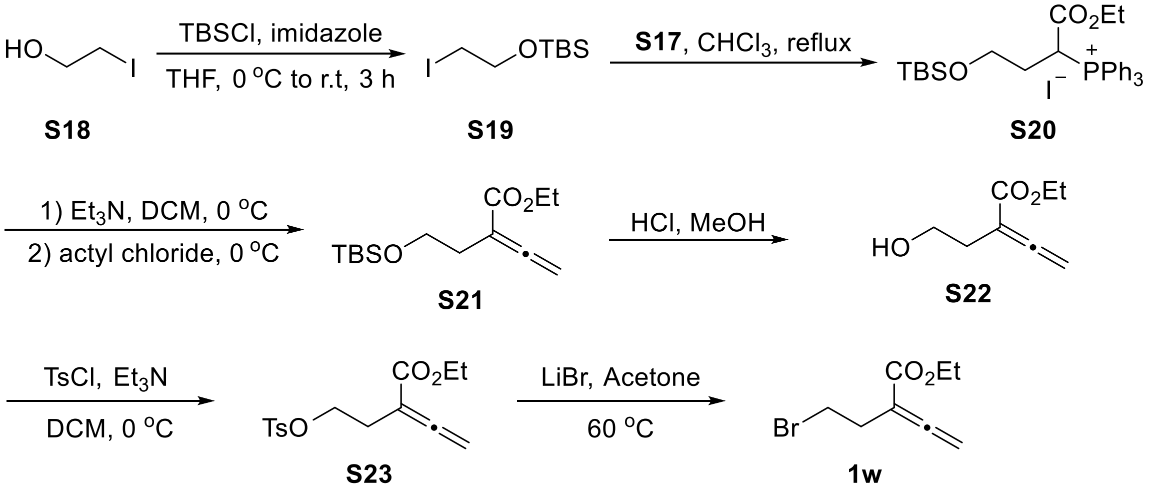

Protection of Primary Alcohol: To a solution of 2-iodoethanol **S18** (4.60 g, 10 mmol, 1.0 equiv.) in THF (15 mL) was added imidazole (24 mmol, 2.4 equiv.) and tert-butyl dimethylsilyl chloride (TBSCl) (12 mmol, 1.2 equiv.) at 0 °C. After stirring at ambient temperature for 3 h, the reaction mixture was filtered through a pad of Celite and washed with 10 mL of EtOAc. The filtrate was concentrated under reduced pressure to give the desired product **S19** (99% yield) as a colorless oil with quantity yield, which used in next step without further purification.

Preparation of Ylide: To an oven-dried two-necked flask with condenser was charged triphenylphosphorane **S17u** (10 mmol, 1 equiv) and dry anhydrous $CHCl_3$ (5 mL) under an argon atmosphere. Then, *tert*-butyl(2-iodoethoxy)dimethylsilane **S19** (10 mmol) in dry anhydrous $CHCl_3$ (5 mL) was added to the reaction flask. The reaction mixture was reflux for overnight and cooling to room temperature. The solvent was removed under reduced pressure and used in next step without further purification.

Wittig Reaction for the Preparation of Allene: To an oven-dried two-necked flask was charged triphenylphosphorane **S20** (10 mmol, 1.0 equiv.) and dry anhydrous DCM (15 mL) under an argon atmosphere and cooled to 0 °C. Et_3N (1.0 mL, 15 mmol, 1.5 equiv.) was added and the reaction mixture was stirring for 2 h at the same temperature. Then, actyl chloride (12 mmol, 1.2 equiv.) was added dropwise at 0 °C. Then, the reaction mixture was allowed to warm to room temperature and stirred for 2 h. The solvent was removed under reduced pressure (35 °C, 500 mbar), and pentane (30 mL) was added. The solution was stirring strongly for 30 min, then, the white precipitate was filtered through a pad of Celite. The filtrate was concentrated under reduced pressure (35 °C, 500 mbar) and the crude product was purified by flash column chromatography on silica gel (Pentanes/Et_2O – 20:1) to give the TBS-protected allene **S21** (260 mg, 65% yield) as a colorless oil.

$\mathbf{R}_f$ = 0.30 (pentane/Et_2O – 20:1).

^{1}H NMR (400 MHz, $CDCl_3$) δ = 5.10 (t, *J* = 2.5 Hz, 2H), 4.20 (q, *J* = 7.1 Hz, 2H), 3.72 (t, *J* = 6.7 Hz, 2H), 2.46 (tt, *J* = 6.8, 2.5 Hz, 2H), 1.28 (t, *J* = 7.1 Hz, 3H), 0.89 (s, 9H), 0.04 (s, 6H).

^{13}C NMR (101 MHz, $CDCl_3$) δ = 214.7, 167.2, 97.2, 78.5, 61.7, 61.1, 32.1, 26.0, 18.4, 14.4, -5.2.

HRMS (+ p APCI) m/z: $[M+H]^+$ Calcd for $C_{14}H_{26}O_3Si^+$: 271. 1725; Found: 271. 1724.

Deprotection of TBS-allene: To a solution of allenyl ester **S21** (1.612 g, 8 mmol, 1.0 equiv.) in MeOH (20 mL) at 0 °C was added HCl (0.8 mL, 0.8 mmol, 0.1 equiv.). The solution was allowed to warm to room temperature and stirred for 1 h. The solvent was removed under reduced pressure. Water (10 mL) was added and extracted with Et_2O (3 x 15 mL). The combined organic phases were washed with brine and dried over Na_2SO_4. The solvents were removed under reduced pressure and the crude product was purified by column chromatography on silica gel (Pentane/Et_2O – 1:1) to give the allene **S22** (870 mg, 70% yield) as a colorless oil.

Protection of OH-allene: To a solution of alcohol allene **S22** (870 mg, 5.6 mmol, 1.0 equiv.) in DCM (30 mL) at 0 °C was added Et_3N (0.93 mL, 6.72 mmol, 1.2 equiv.), and the solution was stirred for 5 min. Then, TsCl (1.178 g, 1,16 mmol, 1.1 equiv.) was added, and DMAP, 4-dimethylaminopyridine (*cat.* amount) was added subsequently. The solution was allowed to warm to room temperature and stirred for additional 5 hours. The reaction mixture was quenched by water (15 mL). The aqueous phase was extracted with DCM (3 × 15 mL). The combined organic phases were washed with brine and dried over Na_2SO_4. The solvents were removed under reduced pressure and the crude product was purified by flash column chromatography on silica gel (Pentanes/EtOAc – 3:1) to give the desired product **S23** (1.13 g, 65% yield) as a colorless oil.

Bromination of OTs-allene: To a solution of tosylate allene S**23** (1.13 g, 3.6 mmol, 1.0 equiv.) in acetone (30 mL, 0.12 M) was added LiBr (1.566 g, 18 mmol, 5.0 equiv.). Then, the solution was stirred at 60 °C for 12 hours. The solvents were removed under reduced pressure and the crude product was purified by flash column chromatography on silica gel (Pentanes/Et_2O – 20:1) to give the desired product **1t** (397 mg, 51% yield) as a colorless oil.

R*f* = 0.35 (pentane/Et_2O – 20:1).

^{1}H NMR (400 MHz, $CDCl_3$) δ = 5.22 (t, *J* = 2.6 Hz, 2H), 4.21 (q, *J* = 7.1 Hz, 2H), 3.50 (t, *J* = 7.0 Hz, 2H), 2.80 (tt, *J* = 7.0, 2.6 Hz, 2H), 1.29 (t, *J* = 7.1 Hz, 3H).

^{13}C NMR (101 MHz, $CDCl_3$) δ = 214.3, 166.5, 98.0, 80.1, 61.4, 31.9, 30.8, 14.3.

HRMS (+ p ESI) m/z: $[M+H]^+$ Calcd for $C_8H_{11}O^{79}Br^+$: 219.0015; Found: 219.0017.

3. List and Preparation of Carbonic Acids.

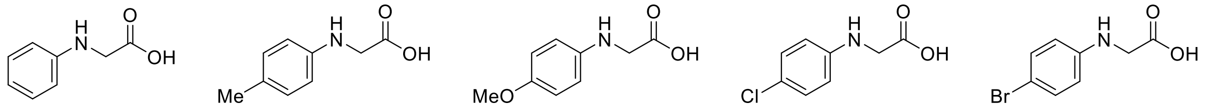

2a 2b 2c 2d 2e

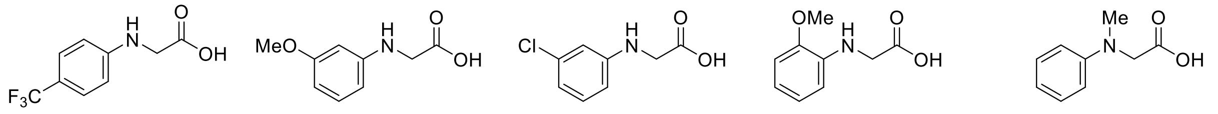

2f 2g 2h 2i 2j

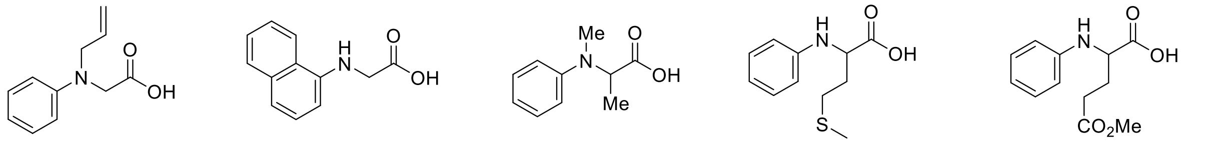

2k 2l 2m 2n 2o

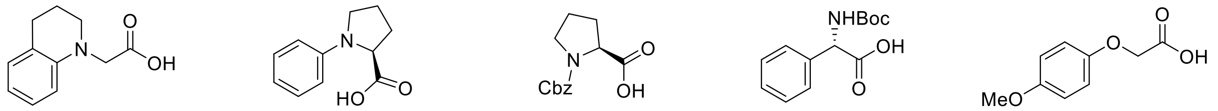

2p 2q 2l 2m 2n

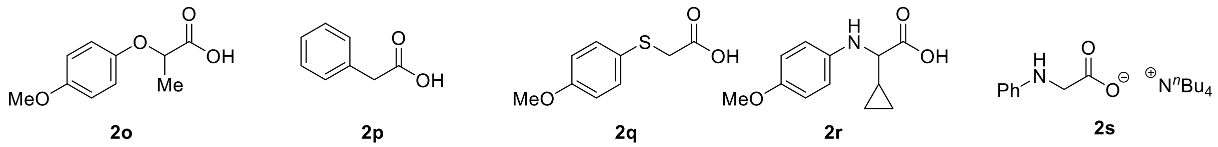

2o 2p 2q 2r 2s

General Procedure C:

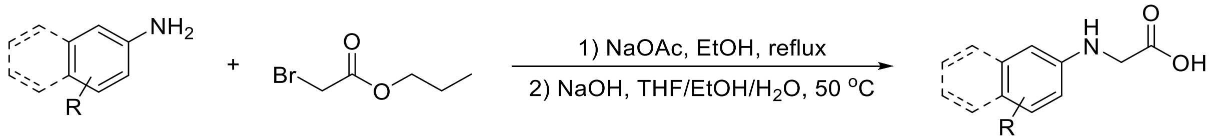

S24 S25 2

A mixture of substituted amine **S24** (10.0 mmol, 1.0 equiv.), ethyl bromoacetate **S25** (12.0 mmol, 1.2 equiv.) and anhydrous sodium acetate (15.0 mmol, 1.5 equiv.) in 30 mL ethanol was refluxed for about 10 h until the substituted aniline disappeared. After cooling to room temperature, the precipitated salts were removed by filtration. The filtrate was concentrated under reduced pressure. The concentrate was dissolved in a mixed solvent of H_2O (10 mL), EtOH (10 mL), and THF (30 mL), NaOH (33.0 mmol, 3.3 equiv.) was added subsequently. The mixture was stirred for 3 h at 50 °C. The organic solvent was then removed by rotary evaporation. The residue was extracted with ethyl acetate (3 × 10 mL). The aqueous layer was

acidified with *conc.* HCl until pH = 2~3 and extracted with ethyl acetate (3 × 20 mL). The combined organic layers were concentrated by rotary evaporation to afford the amino acid **2**.

General Procedure D:

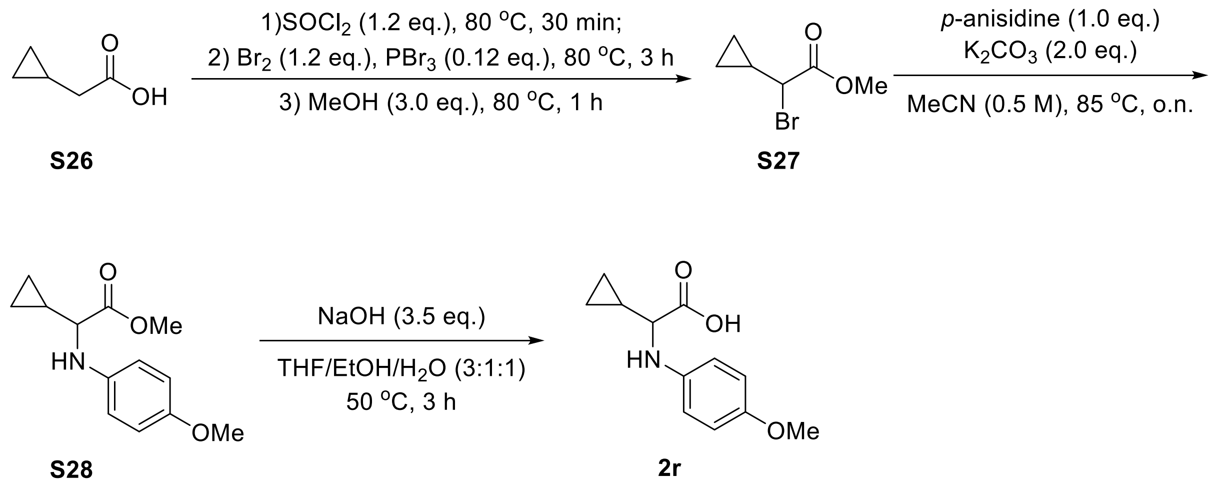

Step 1. Cyclopropylacetic acid **S26** (1.00 mL, 10.0 mmol, 1.0 equiv.) was mixed with $SOCl_2$ (0.87 mL, 12.0 mmol, 1.2 equiv.) and the mixture was stirring at 80 °C for 30 min. Then, Br_2 (0.6 mL, 12.0 mmol, 1.2 equiv.) and PBr_3 (116 μL, 1.20 mmol, 0.12 equiv.) were added and refluxing continued until the red color disappeared (approximately 3 h). Subsequently, dry methanol (1.22 mL, 30.0 mmol, 3.0 equiv.) was added dropwise and the solution was refluxed for another 1 hour. After cooling, a few crystals of $Na_2S_2O_3$ were added. The mixture was filtered and the excess MeOH was evaporated under reduced pressure. The crude product was purified by short column chromatography (silica, n-pentane/EtOAc 10:1) yielding impure **S27** which was used for the subsequent step.

Step 2. A stirred solution of α-bromo ester **S27** (5. 0 mmol, 1.0 euqiv.), *p*-anisidine (615 mg, 5.0 mmol, 1.0 equiv.) and K_2CO_3 (1.38 g, 10.0 mmol, 2.0 equiv.) in dry anhydrous MeCN (10 mL) was stirred at 85°C for 15 h. Solvent was removed under reduced pressure and the residue was purified by flash column chromatography (silica, n-pentane/Et_2O 5:1) yielding **S28** (717 mg, 61% yield) as a yellow oil.

R_f = 0.30 (pentane/Et_2O – 4:1).

^{1}H NMR (500 MHz, $CDCl_3$) δ = 6.77 – 6.72 (m, 2H), 6.59 – 6.54 (m, 2H), 3.73 (s, 6H), 3.49 (d, *J* = 7.6 Hz, 1H), 1.22 – 1.14 (m, 1H), 0.62 – 0.49 (m, 3H), 0.41 – 0.35 (m, 1H).

^{13}C NMR (126 MHz, $CDCl_3$) δ = 174.4, 152.9, 141.2, 115.0, 115.0, 61.2, 55.8, 52.2, 14.2, 3.3, 2.7.

HRMS (+ p ESI) m/z: $[M+H]^+$ Calcd for $C_{13}H_{17}NO_3$: 236.1286; Found: 236.1286.

Step 3. The ester **S28** (1.0 equiv.) was dissolved in a mixed solvent of H_2O (3 mL), EtOH (3 mL), and THF (9 mL), NaOH (420 mg, 10.5 mmol, 3.3 equiv.) was added subsequently. The mixture was stirred for 3 h at 50 °C. The organic solvent was then removed by rotary evaporation. The residue was extracted with ethyl acetate (3 × 10 mL). The aqueous layer was acidified with *conc.* HCl until pH = 2~3 and extracted with ethyl acetate (3 × 20 mL). The combined organic layers were concentrated by rotary evaporation to afford the amino acid **2r** (504 mg, 75% yield) as brown solid.

^{1}H NMR (500 MHz, DMSO-d_6) δ = 8.94 (brs, 1H), 6.71 – 6.67 (m, 2H), 6.54 – 6.49 (m, 2H), 3.62 (s, 3H), 3.25 (d, J = 8.0 Hz, 1H), 1.18 – 1.08 (m, 1H), 0.54 – 0.48 (m, 2H), 0.43 – 0.38 (m, 1H), 0.38 – 0.29 (m, 1H).

^{13}C NMR (126 MHz, CDCl3) δ = 175.0, 151.0, 142.1, 114.5, 113.5, 60.1, 55.3, 13.7, 3.6, 2.4.

HRMS (– p APCI) m/z: $[M–H]^–$ Calcd for $C_{12}H_{15}NO_3$: 220.0797; Found: 220.0796.

Procedure of amino acid salt 2s:

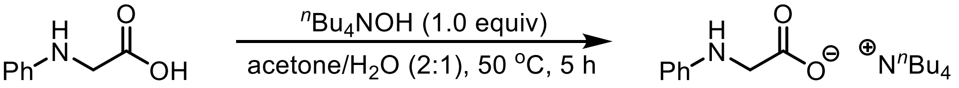

2a **2s**

N-Phenylglycine (302 mg, 2 mmol) and ^{n}Bu$_4$NOH (2 mL, 1 M in H_2O, 2 mmol, 1.0 equiv) were dissolved in acetone (6 mL) and H_2O (3 mL) and stirred at 50°C for 3 h. Then, the solvent was evaporated and the residue was dried in vacuum. *N*-phenylglycine ^{n}Bu$_4$N salt **2s** as a lightgrey solid was obtained (784 mg, >99% yield).

^{1}H NMR (400 MHz, $CDCl_3$) δ = 7.11 – 7.02 (m, 2H), 6.57 – 6.47 (m, 3H), 3.54 (s, 2H), 3.26 – 3.13 (m, 8H), 1.64 – 1.49 (m, 8H), 1.37 (h, J = 7.3 Hz, 8H), 0.94 (t, J = 7.3 Hz, 12H).

^{13}C NMR (101 MHz, $CDCl_3$) δ = 173.6, 148.9, 129.0, 115.5, 112.5, 58.8, 48.6, 24.1, 19.8, 13.7.

HRMS (– p ESI) m/z: $[M–{}^nBu_4N]^–$ Calcd for $C_8H_9NO_2$: 150.0563; Found: 150.0563.

4. Optimization of the Reaction Conditions.

Table S1. Initial exploration[a]

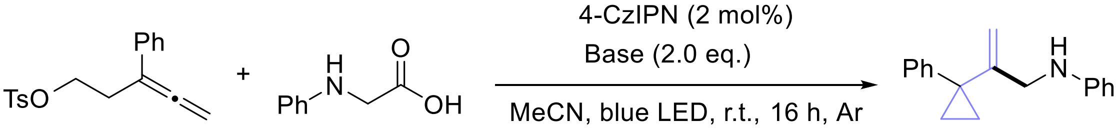

1a **2a** **3**

entry	base	conversion of **1a**/%[b]	yield/%[c]
1	K_2HPO_4	20	18 (16)
2	KH_2PO_4	1	n.d.
3	Cs_2CO_3	12	11
4	K_2CO_3	10	10
5	Na_2CO_3	20	8
6	NaOAc	25	13
7	K_2HPO_4 (1.0 eq.)	18	10

[a]Reaction conditions (unless otherwise specified): **1a** (0.1 mmol,1.0 equiv.), **2a** (0.12 mmol, 1.2 equiv.), 4-CzIPN (0.002 mmol, 2 mol %), base (0.2 mmol, 2 equiv.), MeCN (2 mL, 0.05 M), blue LED, r.t. under Ar atmosphere for 16 h. [b]NMR yields are reported by using dibromomethane as internal standard. [c]NMR yields are reported by using dibromomethane as internal standard, isolated yield is presented in parenthesis. n.d. is no product **3** was detected.

Table S2. Screening of leaving-groups[a]

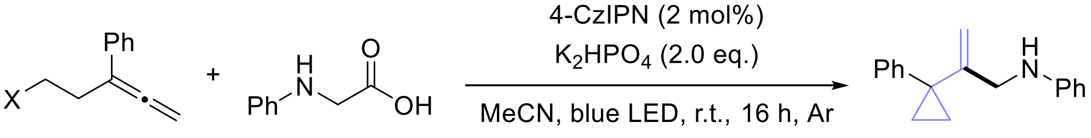

1 **2a** **3**

entry	X (leaving-group)	conversion of **1**/%[b]	yield/%[c]
1	OTs (**1a**)	20	18
2	OMs (**1b**)	45	16
3	Cl (**1c**)	55	18
4	I (**1d**)	75	23
5	Br (**1e**)	50	45 (46)

[a]Reaction conditions (unless otherwise specified): **1** (0.1 mmol,1.0 equiv.), **2a** (0.12 mmol, 1.2 equiv.), 4-CzIPN (0.002 mmol, 2 mol %), base (0.2 mmol, 2 equiv.), MeCN (2 mL, 0.05 M), blue LED, r.t. under Ar atmosphere for 16 h. [b]NMR yields are reported by using dibromomethane as internal standard. [c]NMR yields are reported by using dibromomethane as internal standard, isolated yield is presented in parenthesis.

Table S3. Optimization of photocatalysts[a]

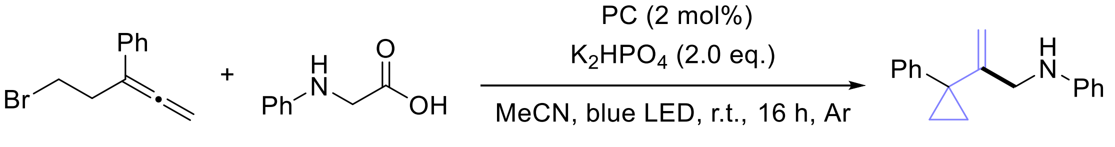

1e **2a** **3**

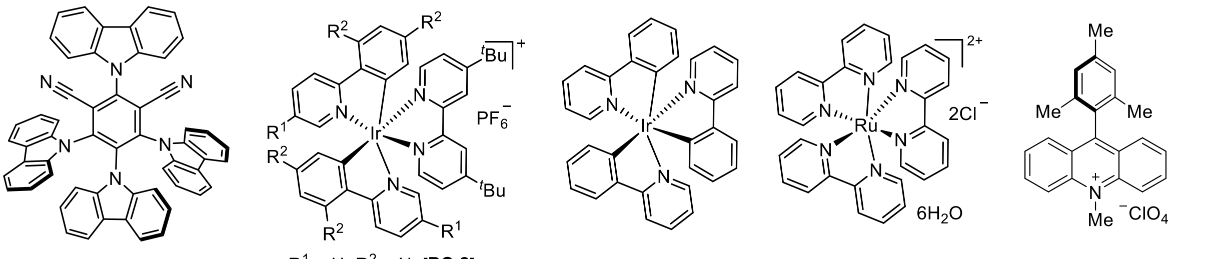

4-CzIPN [**PC1**]
R[1] = H, R[2] = H, [**PC 2**]
R[1] = CF_3, R[2] = F, [**PC 3**]
fac-Ir(ppy)$_3$ [**PC5**]
Ru(bpy)$_3$Cl$_2$·6H$_2$O [**PC4**]
[Acr-Mes]$^+$ClO$_4^-$ [**PC6**]

entry	PC	conversion of **1e**/%[b]	yield/%[c]
1	4-CzIPN [**PC1**]	50	45
2	[Ir(ppy)$_2$dtbbpy]PF$_6$ [**PC2**]	91	70 (70)
3	[Ir(dFCF$_3$ppy)$_2$dtbbpy]PF$_6$ [**PC3**]	81	68 (66)
4	Ir(ppy)$_3$ [**PC4**]	15	trace
5	Ru(bpy)$_3$Cl•6H$_2$O [**PC5**]	45	36
6	[Acr-Mes]$^+$ClO$_4^-$ [**PC6**]	30	12

[a]Reaction conditions (unless otherwise specified): **1e** (0.1 mmol,1.0 equiv.), **2a** (0.12 mmol, 1.2 equiv.), [PC] (0.002 mmol, 2 mol %), K_2HPO_4 (0.2 mmol, 2 equiv.), MeCN (2 mL, 0.05 M), blue LED, r.t. under Ar atmosphere for 16 h. [b]NMR yields are reported by using dibromomethane as internal standard. [c]NMR yields are reported by using dibromomethane as internal standard, isolated yield is presented in parenthesis.

Table S4. Optimization of solvents[a]

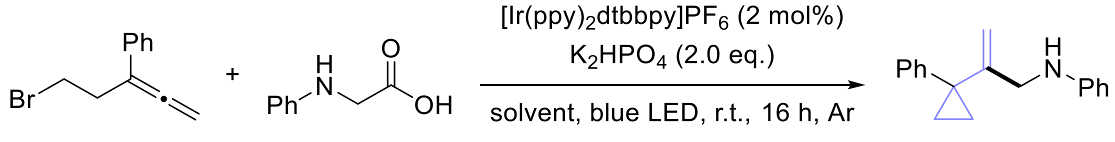

1e **2a** **3**

entry	solvent	conversion of **1e**/%[b]	yield/%[c]
1	MeCN	91	70
2	DMF	85	55
3	THF	76	43
4	acetone	74	58
5	DMSO	100	56
6	1,4-dioxane	39	26
7	MeOH	82	48
8	MeCN/DMSO (9:1)	100	68
9[d]	MeCN	90	59

[a]Reaction conditions (unless otherwise specified): **1e** (0.1 mmol,1.0 equiv.), **2a** (0.12 mmol, 1.2 equiv.), [Ir(ppy)$_2$dtbbpy]PF$_6$ (0.002 mmol, 2 mol %), K_2HPO_4 (0.2 mmol, 2 equiv.), MeCN (2 mL, 0.05 M), blue LED, r.t. under Ar atmosphere for 16 h. [b]NMR yields are reported by using dibromomethane as internal standard. [c]NMR yields are reported by using dibromomethane as internal standard, isolated yield is presented in parenthesis. [d]MeCN (1.0 mL).

Table S5. Control experiments

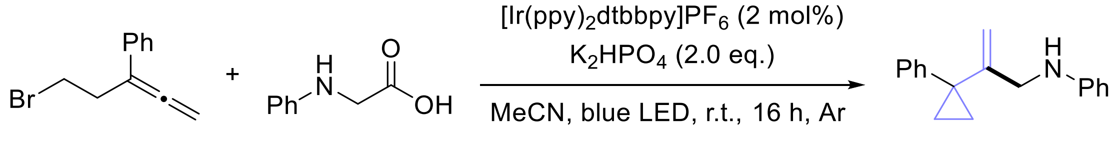

1e, 1.0 equiv **2a**, 1.2 equiv **3**

entry	variations[a]	conversion of **1e**/%[b]	yield/%[c]
1	none	91	70
2	**2a**, 2.0 equiv	98	75
3 [d]	MeCN/DMSO (9:1)	100	81 (80)
4 [d,e]	MeCN/DMSO (9:1)	100	68 (69)
5	w/o base	10	n.d

6	under air	48	15
7	w/o [Ir]	15	n.d.
8	dark	5	n.d.

[a]Reaction conditions (unless otherwise specified): **1e** (0.1 mmol,1.0 equiv.), **2a** (0.12 mmol, 1.2 equiv.), $[Ir(ppy)_2dtbbpy]PF_6$ (0.002 mmol, 2 mol %), K_2HPO_4 (0.2 mmol, 2 equiv.), MeCN (2 mL, 0.05 M), blue LED, r.t. under Ar atmosphere for 16 h. [b]NMR yields are reported by using dibromomethane as internal standard. [c]NMR yields are reported by using dibromomethane as internal standard, isolated yield is presented in parenthesis. [d]**2a** (2.0 equiv.). [e]MeCN (1.0 mL). n.d. is no product **3** was detected.

5. General Procedure E for the Cyclization Reaction.

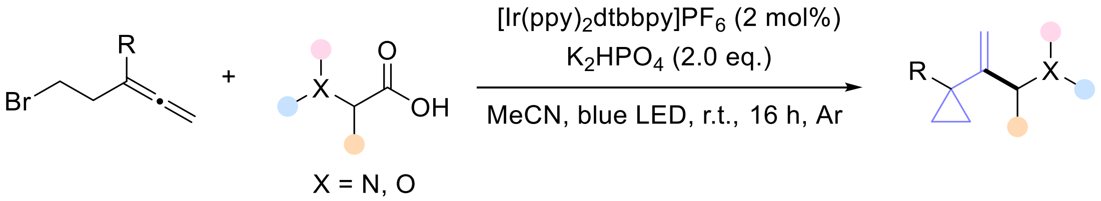

An oven-dried 10 mL Schlenk tube was charged with acid (0.2 mmol, 2.0 equiv), $[Ir(ppy)_2dtbbpy]PF_6$ (1.8 mg, 0.002 mmol, 2 mol %), K_2HPO_4 (34.8 mg, 0.2 mmol, 2 equiv.). The Schlenk tube was put on vacuum and backfilled with argon three times. Afterwards MeCN (1.8 mL) and DMSO (0.2 mL) was added by syringe under a flow of argon. Sequentially, allene **1** (0.1 mmol, 1.0 equiv.) was added by syringe under a flow of argon. The Schlenk tube was sealed by screw cap and the resulting mixture was placed approximately 1 inch from blue led strips and irradiated and stirred for 16 hours at room temperature. After filtration through a pad of Celite and evaporation of the solvents under reduced pressure. The residue was purified by flash column chromatography on silica gel afford the desired vinyl cyclopropane (**VCP**) products.

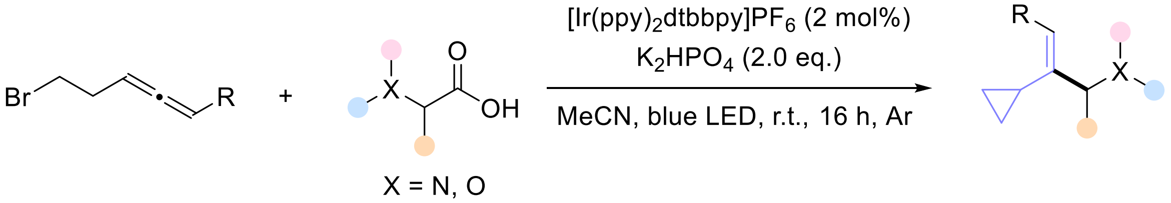

An oven-dried 10 mL Schlenk tube was charged with amino acid (0.2 mmol, 2.0 equiv), $[Ir(ppy)_2dtbbpy]PF_6$ (1.8 mg, 0.002 mmol, 2 mol %), K_2HPO_4 (34.8 mg, 0.2 mmol, 2 equiv.). The Schlenk tube was put on vacuum and backfilled with argon three times. Afterwards MeCN (1.8 mL) and DMSO (0.2 mL) was added by syringe under a flow of argon. Sequentially, allene **1** (0.1 mmol, 1.0 equiv.) was added by syringe under a flow of argon. The Schlenk tube was sealed by screw cap and the resulting mixture was placed approximately 1 inch from blue led strips and irradiated and stirred for 16 hours at room temperature. After filtration through a pad of Celite and evaporation of the solvents under reduced pressure. The residue was purified by flash column chromatography on silica gel afford the desired vinyl cyclopropane (**VCP**) products.

6. Product Characterization.

N-(2-(1-phenylcyclopropyl)allyl)aniline (3)

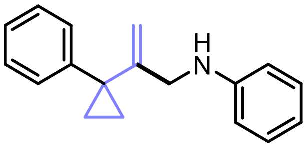

3

Following the **General Procedure E**, after concentration, the crude material was purified by flash column chromatography (SiO_2, Pentanes/Et_2O – 40:1) to furnish the title compound as a colorless oil (19.8 mg, 80% yield).

R_f = 0.35 (pentane/Et_2O = 40:1).

^{1}H NMR (400 MHz, $CDCl_3$) δ = 7.36 – 7.28 (m, 4H), 7.24 – 7.19 (m, 1H), 7.16 – 7.10 (m, 2H), 6.67 (tt, J = 7.3, 1.1 Hz, 1H), 6.49 – 6.45 (m, 2H), 5.17 (q, J = 1.3 Hz, 1H), 5.15 (q, J = 1.5 Hz, 1H), 3.80 (brs, 1H), 3.70 (t, J = 1.5 Hz, 2H), 1.07 – 1.06 (m, 2H), 1.05 – 1.03 (m, 2H).

^{13}C NMR (101 MHz, $CDCl_3$) δ = 149.3, 148.2, 143.7, 129.2, 128.4, 128.4, 126.4, 117.3, 112.8, 111.1, 47.3, 30.1, 13.2.

HRMS (+ p APCI) m/z: $[M+H]^+$ Calcd for $C_{18}H_{20}N^+$: 250.1590; Found: 250.1594.

4-methyl-N-(2-(1-phenylcyclopropyl)allyl)aniline (4)

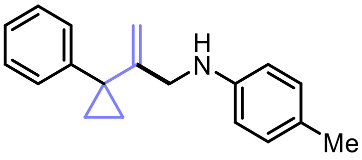

4

Following the **General Procedure E**, after concentration, the crude material was purified by flash column chromatography (SiO_2, Pentanes/Et_2O – 40:1) to furnish the title compound as a colorless oil (19.7 mg, 75% yield).

R_f = 0.35 (pentane/Et_2O = 40:1).

^{1}H NMR (500 MHz, $CDCl_3$) δ = 7.36 – 7.26 (m, 4H), 7.25 – 7.18 (m, 1H), 6.97 – 6.90 (m, 2H), 6.43 – 6.36 (m, 2H), 5.15 (q, J = 1.3 Hz, 1H), 5.13 (q, J = 1.6 Hz, 1H), 3.69 (brs, 1H), 3.67 (t, J = 1.5 Hz, 2H), 2.22 (s, 3H), 1.08 – 1.05 (m, 2H), 1.04 – 1.01 (m, 2H).

^{13}C NMR (126 MHz, $CDCl_3$) δ = 149.4, 145.9, 143.7, 129.7, 128.4, 126.4, 112.9, 111.0, 47.5, 30.1, 20.4, 13.2.

HRMS (+ p ESI) m/z: $[M+H]^+$ Calcd for $C_{19}H_{22}N^+$: 264.1747; Found: 264.1746.

4-methoxy-N-(2-(1-phenylcyclopropyl)allyl)aniline (5)

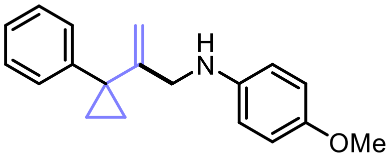

5

Following the **General Procedure E**, after concentration, the crude material was purified by flash column chromatography (SiO_2, Pentanes/Et_2O – 40:1) to furnish the title compound as a colorless oil (19.5 mg, 70% yield).

R_f = 0.30 (pentane/Et_2O = 30:1).

^{1}H NMR (500 MHz, $CDCl_3$) δ = 7.35 – 7.27 (m, 4H), 7.23 – 7.19 (m, 1H), 6.75 – 6.70 (m, 2H), 6.45 – 6.41 (m, 2H), 5.15 (q, *J* = 1.3 Hz, 1H), 5.13 (q, *J* = 1.6 Hz, 1H), 3.73 (s, 3H), 3.65 (t, *J* = 1.5 Hz, 2H), 3.57 (brs, 1H), 1.07 – 1.00 (m, 4H).

^{13}C NMR (126 MHz, $CDCl_3$) δ = 152.0, 149.5, 143.7, 142.5, 128.4, 126.4, 114.9, 114.0, 111.1, 55.9, 48.1, 30.1, 13.2.

HRMS (+ p APCI) m/z: $[M+H]^+$ Calcd for $C_{19}H_{21}NO^+$: 280.1697; Found: 280.1697.

4-chloro-N-(2-(1-phenylcyclopropyl)allyl)aniline (6)

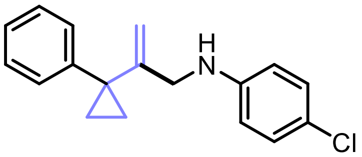

6

Following the **General Procedure E**, after concentration, the crude material was purified by flash column chromatography (SiO_2, Pentanes/Et_2O – 40:1) to furnish the title compound as a colorless oil (21.6 mg, 77% yield).

R_f = 0.35 (pentane/Et_2O = 40:1).

^{1}H NMR (500 MHz, $CDCl_3$) δ = 7.35 – 7.27 (m, 4H), 7.24 – 7.19 (m, 1H), 7.07 – 7.02 (m, 2H), 6.37 – 6.32 (m, 2H), 5.16 (q, *J* = 1.3 Hz, 1H), 5.09 (q, *J* = 1.5 Hz, 1H), 3.83 (brs, 1H), 3.66 (t, *J* = 1.5 Hz, 2H), 1.05 – 1.01 (m, 4H).

^{13}C NMR (126 MHz, $CDCl_3$) δ = 148.9, 146.7, 143.5, 129.0, 128.5, 128.4, 126.5, 121.7, 113.8, 111.2, 47.3, 30.0, 13.1.

HRMS (+ p APCI) m/z: $[M+H]^+$ Calcd for $C_{18}H_{19}N^{35}Cl^+$: 282.1201; Found: 282.1202.

4-bromo-N-(2-(1-phenylcyclopropyl)allyl)aniline (7)

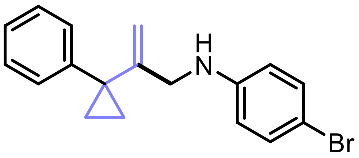

7

Following the **General Procedure E**, after concentration, the crude material was purified by flash column chromatography (SiO_2, Pentanes/Et_2O – 40:1) to furnish the title compound as a colorless oil (21.3 mg, 65% yield).

R_f = 0.35 (pentane/Et_2O = 40:1).

^{1}H NMR (500 MHz, $CDCl_3$) δ = 7.34 – 7.27 (m, 4H), 7.24 – 7.20 (m, 1H), 7.19 – 7.16 (m, 2H), 6.33 – 6.28 (m, 2H), 5.16 (q, *J* = 1.3 Hz, 1H), 5.09 (q, *J* = 1.6 Hz, 1H), 3.84 (brs, 1H), 3.66 (t, *J* = 1.5 Hz, 2H), 1.06 – 1.00 (m, 4H).

^{13}C NMR (126 MHz, $CDCl_3$) δ = 148.8, 147.1, 143.4, 131.8, 128.5, 128.4, 126.5, 114.3, 111.2, 108.7, 47.2, 30.0, 13.1.

HRMS (+ p APCI) m/z: $[M+H]^+$ Calcd for $C_{18}H_{19}N^{79}Br^+$: 328.0695; Found: 328.0693.

N-(2-(1-phenylcyclopropyl)allyl)-4-(trifluoromethyl)aniline (8)

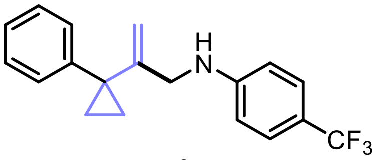

8

Following the **General Procedure E**, after concentration, the crude material was purified by flash column chromatography (SiO_2, Pentanes/Et_2O – 40:1) to furnish the title compound as a colorless oil (25.8 mg, 82% yield).

R_f = 0.35 (pentane/Et_2O = 40:1).

^{1}H NMR (500 MHz, $CDCl_3$) δ = 7.35 – 7.28 (m, 6H), 7.25 – 7.21 (m, 1H), 6.45 – 6.39 (m, 2H), 5.18 (q, *J* = 1.3 Hz, 1H), 5.09 (q, *J* = 1.6 Hz, 1H), 4.15 (brs, 1H), 3.72 (t, *J* = 1.5 Hz, 2H), 1.04 (s, 4H).

^{19}F NMR (471 MHz, $CDCl_3$) δ = -60.97.

^{13}C NMR (126 MHz, $CDCl_3$) δ = 150.6, 148.4, 143.3, 128.5, 128.4, 126.6, 126.5 (d, *J* = 4.1 Hz), 125.1 (q, *J* = 270.2 Hz), 118.7 (q, *J* = 32.7 Hz), 111.8, 111.3, 46.9, 30.0, 13.1.

HRMS (– p ESI) m/z: $[M–H]^–$ Calcd for $C_{19}H_{17}NF_3^–$: 316.1317; Found: 316.1319.

3-chloro-N-(2-(1-phenylcyclopropyl)allyl)aniline (9)

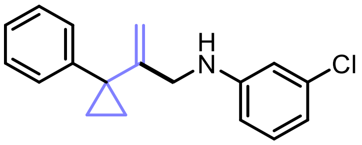

9

Following the **General Procedure E**, after concentration, the crude material was purified by flash column chromatography (SiO_2, Pentanes/Et_2O – 40:1) to furnish the title compound as a colorless oil (21.0 mg, 75% yield).

$\boldsymbol{R_f}$ = 0.35 (pentane/Et_2O = 40:1).

^{1}H NMR (400 MHz, $CDCl_3$) δ = 7.35 – 7.28 (m, 4H), 7.24 – 7.19 (m, 1H), 7.00 (t, *J* = 8.0 Hz, 1H), 6.62 (ddd, *J* = 7.9, 2.0, 0.9 Hz, 1H), 6.42 – 6.39 (m, 1H), 6.31 (ddd, *J* = 8.2, 2.3, 0.9 Hz, 1H), 5.17 (q, *J* = 1.3 Hz, 1H), 5.11 (q, *J* = 1.6 Hz, 1H), 3.89 (brs, 1H), 3.67 (s, 2H), 1.06 – 1.03 (m, 4H).

^{13}C NMR (101 MHz, $CDCl_3$) δ = 149.3, 148.7, 143.4, 135.0, 130.1, 128.5, 128.4, 126.5, 117.1, 112.4, 111.3, 111.1, 47.1, 30.0, 13.1.

HRMS (+ p ESI) m/z: $[M+H]^+$ Calcd for $C_{18}H_{19}N^{35}Cl^+$: 282.1201; Found: 282.1200.

2-methoxy-N-(2-(1-phenylcyclopropyl)allyl)aniline (10)

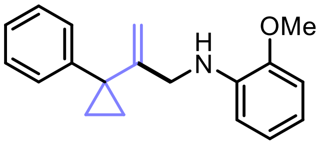

10

Following the **General Procedure E**, after concentration, the crude material was purified by flash column chromatography (SiO_2, Pentanes/Et_2O – 40:1) to furnish the title compound as a colorless oil (17.3 mg, 62% yield).

$\boldsymbol{R_f}$ = 0.35 (pentane/Et_2O = 40:1).

^{1}H NMR (500 MHz, $CDCl_3$) δ = 7.36 – 7.27 (m, 4H), 7.23 – 7.19 (m, 1H), 6.82 – 6.73 (m, 2H), 6.66 – 6.59 (m, 1H), 6.36 (dd, *J* = 7.8, 1.5 Hz, 1H), 5.15 (q, *J* = 1.4 Hz, 1H), 5.13 (q, *J* = 1.6 Hz, 1H), 4.49 (brs, 1H), 3.84 (s, 3H), 3.72 (t, *J* = 1.6 Hz, 2H), 1.10 – 1.00 (m, 4H).

^{13}C NMR (126 MHz, $CDCl_3$) δ = 149.1, 146.8, 143.7, 138.2, 128.37, 128.36, 126.3, 121.3, 116.3, 111.0, 110.1, 109.5, 55.6, 47.0, 30.1, 13.2.

HRMS (+ p APCI) m/z: $[M+H]^+$ Calcd for $C_{19}H_{21}NO^+$: 280.1697; Found: 280.1698.

N-methyl-N-(2-(1-phenylcyclopropyl)allyl)aniline (11)

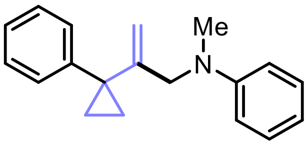

11

Following the **General Procedure E**, after concentration, the crude material was purified by flash column chromatography (SiO_2, Pentanes/Et_2O – 40:1) to furnish the title compound as a colorless oil (19.5 mg, 74% yield).

R_f = 0.35 (pentane/Et_2O = 40:1).

^{1}H NMR (500 MHz, $CDCl_3$) δ = 7.36 – 7.29 (m, 4H), 7.24 – 7.20 (m, 1H), 7.19 – 7.12 (m, 2H), 6.64 (tt, *J* = 7.3, 1.1 Hz, 1H), 6.53 – 6.48 (m, 2H), 5.13 (q, *J* = 1.6 Hz, 1H), 4.91 (q, *J* = 1.8 Hz, 1H), 3.81 (t, *J* = 1.8 Hz, 2H), 2.88 (s, 3H), 1.09 – 1.06 (m, 2H), 1.06 – 1.02 (m, 2H).

^{13}C NMR (126 MHz, $CDCl_3$) δ = 149.4, 147.2, 143.6, 129.0, 128.4, 128.2, 126.4, 115.8, 111.5, 110.6, 56.2, 38.5, 29.7, 13.2.

HRMS (+ p ESI) m/z: $[M+H]^+$ Calcd for $C_{19}H_{22}N^+$: 264.1747; Found: 264.1745.

N-allyl-N-(2-(1-phenylcyclopropyl)allyl)aniline (12)

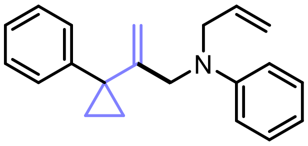

12

Following the **General Procedure E**, after concentration, the crude material was purified by flash column chromatography (SiO_2, Pentanes/Et_2O – 40:1) to furnish the title compound as a colorless oil (22.8 mg, 79% yield).

R_f = 0.35 (pentane/Et_2O = 40:1).

^{1}H NMR (400 MHz, $CDCl_3$) δ = 7.37 – 7.28 (m, 4H), 7.24 – 7.19 (m, 1H), 7.16 – 7.09 (m, 2H), 6.63 (tt, *J* = 7.3, 1.0 Hz, 1H), 6.52 – 6.47 (m, 2H), 5.80 (ddt, *J* = 17.1, 10.3, 4.9 Hz, 1H), 5.17 – 5.00 (m, 3H), 4.95 (q, *J* = 1.8 Hz, 1H), 3.83 (dt, *J* = 4.9, 1.7 Hz, 2H), 3.79 (t, *J* = 1.8 Hz, 2H), 1.12 – 1.01 (m, 4H).

^{13}C NMR (101 MHz, $CDCl_3$) δ = 148.8, 147.0, 143.5, 133.9, 129.0, 128.4, 128.2, 126.4, 116.1, 116.0, 111.8, 110.5, 53.7, 53.0, 29.7, 13.2.

HRMS (+ p APCI) m/z: $[M+H]^+$ Calcd for $C_{21}H_{24}N^+$: 290.1903; Found: 290.1903.

N-(2-(1-phenylcyclopropyl)allyl)naphthalen-1-amine (13)

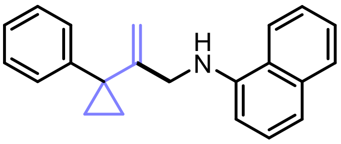

13

Following the **General Procedure E**, after concentration, the crude material was purified by flash column chromatography (SiO_2, Pentanes/Et_2O – 40:1) to furnish the title compound as a colorless oil (20.3 mg, 68% yield).

R_f = 0.35 (pentane/Et_2O = 40:1).

^{1}H NMR (400 MHz, $CDCl_3$) δ = 7.80 – 7.75 (m, 1H), 7.68 – 7.62 (m, 1H), 7.46 – 7.37 (m, 4H), 7.35 – 7.29 (m, 2H), 7.28 – 7.18 (m, 3H), 6.39 (dd, *J* = 7.5, 1.1 Hz, 1H), 5.24 (t, *J* = 1.4 Hz, 2H), 4.54 (brs, 1H), 3.90 (t, *J* = 1.4 Hz, 2H), 1.14 – 1.10 (m, 2H), 1.09 – 1.05 (m, 2H).

^{13}C NMR (101 MHz, $CDCl_3$) δ = 148.9, 143.7, 143.2, 134.4, 128.7, 128.5, 128.4, 126.6, 126.5, 125.7, 124.6, 123.4, 119.8, 117.2, 111.8, 104.4, 47.8, 30.2, 13.3.

HRMS (+ p ESI) m/z: $[M+H]^+$ Calcd for $C_{22}H_{22}N^+$: 300.1747; Found: 300.1748.

N-methyl-N-(3-(1-phenylcyclopropyl)but-3-en-2-yl)aniline (14)

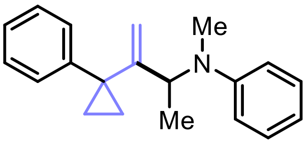

14

Following the **General Procedure E**, after concentration, the crude material was purified by flash column chromatography (SiO_2, Pentanes/Et_2O – 40:1) to furnish the title compound as a colorless oil (21.1 mg, 76% yield).

R_f = 0.35 (pentane/Et_2O = 40:1).

^{1}H NMR (400 MHz, $CDCl_3$) δ = 7.29 – 7.22 (m, 4H), 7.20 – 7.13 (m, 3H), 6.66 (tt, *J* = 7.2, 1.0 Hz, 1H), 6.57 – 6.51 (m, 2H), 5.34 (t, *J* = 1.2 Hz, 1H), 5.12 (t, *J* = 1.4 Hz, 1H), 4.38 (qt, *J* = 6.9, 1.4 Hz, 1H), 2.54 (s, 3H), 1.20 (d, *J* = 6.8 Hz, 3H), 1.10 – 0.91 (m, 4H).

^{13}C NMR (101 MHz, $CDCl_3$) δ = 152.2, 149.7, 144.2, 129.0, 128.6, 128.1, 126.1, 116.0, 112.8, 112.6, 55.5, 31.3, 30.6, 15.4, 13.2, 13.0.

HRMS (+ p ESI) m/z: $[M+H]^+$ Calcd for $C_{20}H_{24}N^+$: 278.1903; Found: 278.1904.

N-(5-(methylthio)-2-(1-phenylcyclopropyl)pent-1-en-3-yl)aniline (15)

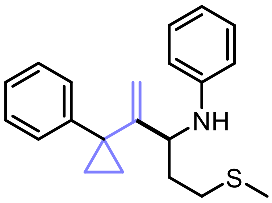

15

Following the **General Procedure E**, after concentration, the crude material was purified by flash column chromatography (SiO_2, Pentanes/Et_2O – 40:1) to furnish the title compound as a colorless oil (24.5 mg, 77% yield).

R_f = 0.35 (pentane/Et_2O = 40:1).

^{1}H NMR (700 MHz, $CDCl_3$) δ = 7.38 – 7.35 (m, 2H), 7.32 – 7.28 (m, 2H), 7.24 – 7.21 (m, 1H), 7.08 – 7.04 (m, 2H), 6.62 (tt, *J* = 7.2, 1.1 Hz, 1H), 6.33 – 6.28 (m, 2H), 5.16 (d, *J* = 1.2 Hz, 1H), 5.12 (t, *J* = 1.1 Hz, 1H), 3.94 (ddd, *J* = 8.6, 4.4, 1.0 Hz, 1H), 3.71 (brs, 1H), 2.48 (ddd, *J* = 13.0, 8.5, 5.4 Hz, 1H), 2.43 (ddd, *J* = 13.1, 8.2, 7.0 Hz, 1H), 2.02 (s, 3H), 2.03 – 1.98 (m, 1H), 1.65 (dtd, *J* = 14.0, 8.4, 5.4 Hz, 1H), 1.20 – 1.11 (m, 2H), 0.94 – 0.86 (m, 2H).

^{13}C NMR (176 MHz, $CDCl_3$) δ = 152.2, 147.4, 143.8, 129.3, 129.1, 128.4, 126.7, 117.1, 113.1, 111.2, 55.9, 34.8, 31.2, 30.3, 15.7, 13.9, 12.3.

HRMS (+ p ESI) m/z: $[M+H]^+$ Calcd for $C_{21}H_{26}NS^+$: 324.1780; Found: 324.1781.

methyl 4-(phenylamino)-5-(1-phenylcyclopropyl)hex-5-enoate (16)

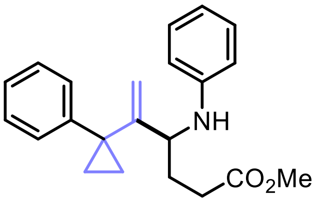

16

Following the **General Procedure E**, after concentration, the crude material was purified by flash column chromatography (SiO_2, Pentanes/Et_2O – 40:1) to furnish the title compound as a colorless oil (22.8 mg, 68% yield).

R_f = 0.35 (pentane/Et_2O = 40:1).

^{1}H NMR (700 MHz, $CDCl_3$) δ = 7.37 – 7.35 (m, 2H), 7.32 – 7.28 (m, 2H), 7.25 – 7.20 (m, 1H), 7.06 – 7.02 (m, 2H), 6.61 (tt, *J* = 7.2, 1.1 Hz, 1H), 6.28 – 6.24 (m, 2H), 5.16 (d, *J* = 1.1 Hz, 1H), 5.11 (t, *J* = 1.1 Hz, 1H), 3.78 (dd, *J* = 8.8, 4.5 Hz, 1H), 3.72 (brs, 1H), 3.63 (s, 3H), 2.30 (td, *J* = 7.2, 1.4 Hz, 2H), 2.09 (dtd, *J* = 14.5, 7.3, 4.5 Hz, 1H), 1.76 (ddt, *J* = 14.3, 8.7, 7.1 Hz, 1H),

1.22 – 1.13 (m, 2H), 0.94 – 0.85 (m, 2H).

^{13}C NMR (176 MHz, $CDCl_3$) δ = 174.3, 151.9, 147.3, 143.7, 129.3, 129.0, 128.4, 126.7, 117.0, 113.0, 111.2, 56.2, 51.7, 31.2, 30.3, 30.2, 13.9, 12.3.

HRMS (+ p ESI) m/z: $[M+H]^+$ Calcd for $C_{22}H_{26}ON^+$: 336.1958; Found: 336.1956.

1-(2-(1-phenylcyclopropyl)allyl)-1,2,3,4-tetrahydroquinoline (17)

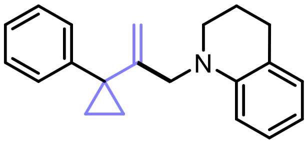

17

Following the **General Procedure E**, after concentration, the crude material was purified by flash column chromatography (SiO_2, Pentanes/Et_2O – 40:1) to furnish the title compound as a colorless oil (19.1 mg, 66% yield).

R_f = 0.35 (pentane/Et_2O = 40:1).

^{1}H NMR (400 MHz, $CDCl_3$) δ = 7.37 – 7.28 (m, 4H), 7.25 – 7.19 (m, 1H), 6.96 – 6.89 (m, 2H), 6.51 (td, *J* = 7.3, 1.1 Hz, 1H), 6.19 (ddd, *J* = 8.1, 1.1, 0.5 Hz, 1H), 5.12 (q, *J* = 1.6 Hz, 1H), 5.00 (q, *J* = 1.8 Hz, 1H), 3.73 (t, *J* = 1.7 Hz, 2H), 3.24 – 3.15 (m, 2H), 2.74 (t, *J* = 6.3 Hz, 2H), 1.97 – 1.87 (m, 2H), 1.13 – 1.01 (m, 4H).

^{13}C NMR (101 MHz, $CDCl_3$) δ = 146.6, 145.5, 143.6, 128.8, 128.4, 128.2, 127.1, 126.3, 121.8, 115.3, 110.5, 110.2, 55.0, 49.8, 29.7, 28.3, 22.4, 13.3.

HRMS (+ p ESI) m/z: $[M+H]^+$ Calcd for $C_{21}H_{24}N^+$: 290.1903; Found: 290.1904.

1-phenyl-2-(1-(1-phenylcyclopropyl)vinyl)pyrrolidine (18)

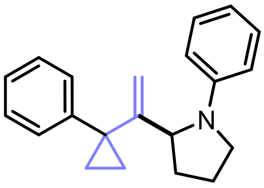

18

Following the **General Procedure E**, after concentration, the crude material was purified by flash column chromatography (SiO_2, Pentanes/Et_2O – 40:1) to furnish the title compound as a colorless oil (16.8 mg, 58% yield).

R_f = 0.35 (pentane/Et_2O = 40:1).

^{1}H NMR (500 MHz, $CDCl_3$) δ = 7.46 – 7.42 (m, 2H), 7.35 – 7.31 (m, 2H), 7.26 – 7.22 (m, 1H), 7.10 – 7.06 (m, 2H), 6.58 (tt, *J* = 7.3, 1.1 Hz, 1H), 6.25 – 6.21 (m, 2H), 5.02 (d, *J* = 1.6 Hz, 1H), 4.83 (t, *J* = 1.4 Hz, 1H), 4.03 – 3.95 (m, 1H), 3.47 – 3.41 (m, 1H), 3.23 – 3.16 (m, 1H), 1.94 – 1.85 (m, 4H), 1.33 – 1.22 (m, 2H), 0.97 (ddd, *J* = 9.3, 6.2, 3.8 Hz, 1H), 0.90 (ddd, *J* = 9.0, 6.2, 4.0 Hz, 1H).

^{13}C NMR (126 MHz, $CDCl_3$) δ = 150.9, 147.0, 144.2, 129.8, 128.8, 128.3, 126.7, 115.3, 111.9, 110.1, 61.9, 48.4, 31.7, 30.3, 22.9, 14.3, 11.9.

HRMS (+ p ESI) m/z: $[M+H]^+$ Calcd for $C_{21}H_{24}N^+$: 290.1903; Found: 290.1904.

1-methoxy-4-((2-(1-phenylcyclopropyl)allyl)oxy)benzene (21)

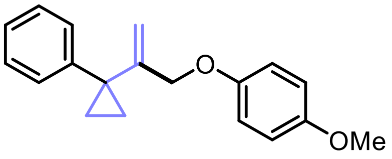

21

Following the **General Procedure E**, after concentration, the crude material was purified by flash column chromatography (SiO_2, Pentanes/Et_2O – 30:1) to furnish the title compound as a colorless oil (15.6 mg, 56% yield).

R_f = 0.30 (pentane/Et_2O = 30:1).

^{1}H NMR (500 MHz, $CDCl_3$) δ = 7.34 – 7.25 (m, 4H), 7.21 – 7.17 (m, 1H), 6.80 – 6.72 (m, 4H), 5.31 (q, *J* = 1.6 Hz, 1H), 5.24 (q, *J* = 1.3 Hz, 1H), 4.38 (t, *J* = 1.4 Hz, 2H), 3.75 (s, 3H), 1.12 – 1.09 (m, 2H), 1.05 – 1.02 (m, 2H).

^{13}C NMR (126 MHz, $CDCl_3$) δ = 153.9, 153.0, 147.6, 143.6, 128.41, 128.38, 126.4, 115.7, 114.6, 112.4, 69.8, 55.8, 29.2, 13.2.

HRMS (+ p APCI) m/z: $[M+H]^+$ Calcd for $C_{19}H_{20}O_2^+$: 281.1535; Found: 281.1536.

1-methoxy-4-((3-(1-phenylcyclopropyl)but-3-en-2-yl)oxy)benzene (22)

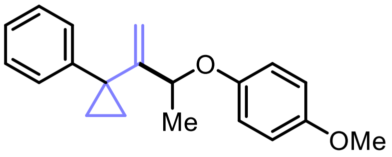

22

Following the **General Procedure E**, after concentration, the crude material was purified by flash column chromatography (SiO_2, Pentanes/Et_2O – 20:1) to furnish the title compound as a

colorless oil (18.8 mg, 64% yield).

Rf = 0.25 (pentane/Et_2O = 20:1).

^{1}H NMR (400 MHz, $CDCl_3$) δ = 7.37 – 7.27 (m, 4H), 7.25 – 7.20 (m, 1H), 6.71 – 6.66 (m, 2H), 6.55 – 6.50 (m, 2H), 5.26 (t, *J* = 1.3 Hz, 1H), 5.18 (dd, *J* = 1.4, 0.4 Hz, 1H), 4.54 (qd, *J* = 6.4, 1.1 Hz, 1H), 3.74 (s, 3H), 1.38 (d, *J* = 6.4 Hz, 3H), 1.24 – 1.15 (m, 2H), 0.99 – 0.84 (m, 2H).

^{13}C NMR (101 MHz, $CDCl_3$) δ = 153.7, 152.3, 152.1, 143.7, 129.2, 128.3, 126.6, 116.8, 114.4, 110.9, 75.3, 55.7, 29.9, 22.0, 14.0, 12.4.

HRMS (+ p APCI) m/z: $[M+H]^+$ Calcd for $C_{20}H_{23}O_2^+$: 295.1963; Found: 295.1963.

When 2-(4-methoxyphenoxy)propanoic acid 2o reaction with the allene 1d under optimal condition, a S_N2 by-product 4b-bP was formed.

3-phenylpenta-3,4-dien-1-yl 2-(4-methoxyphenoxy)propanoate (22-bP)

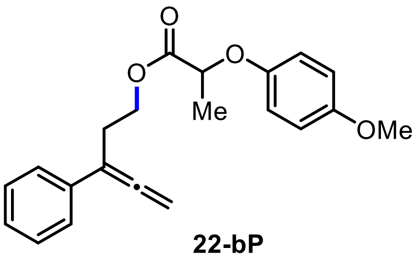

22-bP

Following the **General Procedure E**, after concentration, the crude material was purified by flash column chromatography (SiO_2, Pentanes/Et_2O – 20:1) to furnish the title compound as a colorless oil (7.4 mg, 28% yield).

Rf = 0.20 (pentane/Et_2O = 20:1).

^{1}H NMR (500 MHz, $CDCl_3$) δ = 7.39 – 7.36 (m, 2H), 7.35 – 7.30 (m, 2H), 7.24 – 7.20 (m, 1H), 6.86 – 6.75 (m, 4H), 5.08 (t, *J* = 3.3 Hz, 2H), 4.65 (q, *J* = 6.8 Hz, 1H), 4.37 (t, *J* = 6.9 Hz, 2H), 3.75 (s, 3H), 2.75 (tt, *J* = 6.8, 3.3 Hz, 2H), 1.59 (d, *J* = 6.8 Hz, 3H).

^{13}C NMR (126 MHz, $CDCl_3$) δ = 208.4, 172.5, 154.6, 151.8, 135.6, 128.6, 127.0, 125.9, 116.6, 114.7, 101.2, 79.3, 73.7, 63.5, 55.7, 28.6, 18.7.

HRMS (+ p APCI) m/z: $[M+H]^+$ Calcd for $C_{21}H_{22}O_4^+$: 339.1591; Found: 339.1591.

N-(2-(1-(p-tolyl)cyclopropyl)allyl)aniline (23)

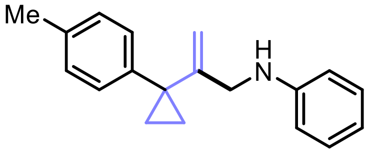

23

Following the **General Procedure E**, after concentration, the crude material was purified by flash column chromatography (SiO_2, Pentanes/Et_2O – 40:1) to furnish the title compound as a colorless oil (18.6 mg, 71% yield).

$\boldsymbol{R_f}$ = 0.35 (pentane/Et_2O = 40:1).

^{1}H NMR (500 MHz, $CDCl_3$) δ = 7.24 – 7.21 (m, 2H), 7.15 – 7.09 (m, 4H), 6.67 (tt, *J* = 7.3, 1.1 Hz, 1H), 6.50 – 6.45 (m, 2H), 5.14 (q, *J* = 1.3 Hz, 1H), 5.12 (q, *J* = 1.6 Hz, 1H), 3.82 (brs, 1H), 3.69 (t, *J* = 1.5 Hz, 2H), 2.34 (s, 3H), 1.06 – 0.98 (m, 4H).

^{13}C NMR (126 MHz, $CDCl_3$) δ = 149.4, 148.2, 140.6, 136.0, 129.2, 129.1, 128.3, 117.2, 112.8, 110.8, 47.3, 29.7, 21.1, 13.1.

HRMS (+ p ESI) m/z: $[M+H]^+$ Calcd for $C_{19}H_{22}N^+$: 264.1747; Found: 264.1744.

N-(2-(1-(4-methoxyphenyl)cyclopropyl)allyl)aniline (24)

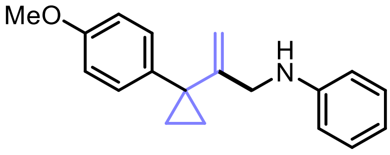

24

Following the **General Procedure E**, after concentration, the crude material was purified by flash column chromatography (SiO_2, Pentanes/Et_2O – 30:1) to furnish the title compound as a colorless oil (20.1 mg, 70% yield).

$\boldsymbol{R_f}$ = 0.30 (pentane/Et_2O = 30:1).

^{1}H NMR (500 MHz, $CDCl_3$) δ = 7.28 – 7.23 (m, 2H), 7.15 – 7.09 (m, 2H), 6.85 – 6.82 (m, 2H), 6.66 (tt, *J* = 7.3, 1.1 Hz, 1H), 6.49 – 6.44 (m, 2H), 5.12 (q, *J* = 1.3 Hz, 1H), 5.09 (q, *J* = 1.6 Hz, 1H), 3.80 (s, 3H), 3.68 (t, *J* = 1.5 Hz, 2H), 2.85 (s, 1H), 1.04 – 0.95 (m, 4H).

^{13}C NMR (126 MHz, $CDCl_3$) δ = 158.2, 149.5, 148.2, 135.7, 129.7, 129.2, 117.2, 113.8, 112.8, 110.6, 55.4, 47.2, 29.5, 12.9.

HRMS (+ p APCI) m/z: $[M+H]^+$ Calcd for $C_{19}H_{21}NO^+$: 280.1697; Found: 280.1694.

N-(2-(1-(4-fluorophenyl)cyclopropyl)allyl)aniline (25)

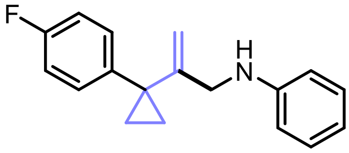

25

Following the **General Procedure E**, after concentration, the crude material was purified by flash column chromatography (SiO_2, Pentanes/Et_2O – 40:1) to furnish the title compound as a colorless oil (19.8 mg, 74% yield).

R$_f$ = 0.35 (pentane/Et_2O = 40:1).

^{1}H NMR (500 MHz, $CDCl_3$) δ = 7.32 – 7.28 (m, 2H), 7.15 – 7.10 (m, 2H), 7.00 – 6.95 (m, 2H), 6.67 (tt, *J* = 7.3, 1.1 Hz, 1H), 6.47 – 6.44 (m, 2H), 5.14 (q, *J* = 1.2 Hz, 1H), 5.12 (q, *J* = 1.6 Hz, 1H), 3.80 (brs, 1H), 3.67 (t, *J* = 1.5 Hz, 2H), 1.08 – 0.97 (m, 4H).

^{19}F NMR (471 MHz, $CDCl_3$) δ = -116.52.

^{13}C NMR (126 MHz, $CDCl_3$) δ = 161.5 (d, *J* = 244.7 Hz), 149.1, 148.1, 139.4 (d, *J* = 3.2 Hz), 130.1 (d, J = 8.1 Hz), 129.2, 117.4, 115.1 (d, *J* = 21.2 Hz), 112.8, 111.2, 47.2, 29.5, 13.1.

HRMS (+ p APCI) m/z: $[M+H]^+$ Calcd for $C_{18}H_{18}FN^+$: 268.1493; Found: 268.1493.

N-(2-(1-(4-chlorophenyl)cyclopropyl)allyl)aniline (26)

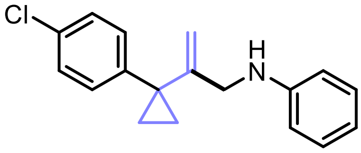

26

Following the **General Procedure E**, after concentration, the crude material was purified by flash column chromatography (SiO_2, Pentanes/Et_2O – 40:1) to furnish the title compound as a colorless oil (19.5 mg, 69% yield).

R$_f$ = 0.35 (pentane/Et_2O = 40:1).

^{1}H NMR (500 MHz, $CDCl_3$) δ = 7.28 – 7.24 (m, 4H), 7.16 – 7.10 (m, 2H), 6.68 (tt, *J* = 7.3, 1.1 Hz, 1H), 6.49 – 6.45 (m, 2H), 5.16 – 5.14 (m, 2H), 3.80 (brs, 1H), 3.67 (t, *J* = 1.5 Hz, 2H), 1.10 – 0.98 (m, 4H).

^{13}C NMR (126 MHz, $CDCl_3$) δ = 148.8, 148.1, 142.2, 132.2, 129.8, 129.2, 128.5, 117.4, 112.8, 111.6, 47.2, 29.6, 13.3.

HRMS (+ p APCI) m/z: $[M+H]^+$ Calcd for $C_{18}H_{18}N^{35}Cl^+$: 284.1201; Found: 284.1205.

N-(2-(1-(4-(trifluoromethyl)phenyl)cyclopropyl)allyl)aniline (27)

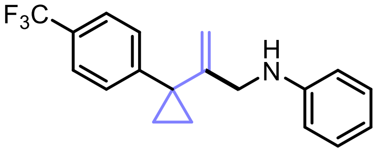

27

Following the **General Procedure E**, after concentration, the crude material was purified by flash column chromatography (SiO_2, Pentanes/Et_2O – 40:1) to furnish the title compound as a colorless oil (28.0 mg, 88% yield).

R

R_f = 0.35 (pentane/Et_2O = 40:1).

^{1}H NMR (500 MHz, $CDCl_3$) δ = 7.57 – 7.51 (m, 2H), 7.46 – 7.40 (m, 2H), 7.16 – 7.10 (m, 2H), 6.68 (tt, *J* = 7.3, 1.1 Hz, 1H), 6.49 – 6.44 (m, 2H), 5.22 (q, *J* = 1.5 Hz, 1H), 5.20 (q, *J* = 1.3 Hz, 1H), 3.81 (brs, 1H), 3.69 (t, *J* = 1.5 Hz, 2H), 1.17 – 1.11 (m, 2H), 1.09 – 1.04 (m, 2H).

^{19}F NMR (471 MHz, $CDCl_3$) δ = -62.35.

^{13}C NMR (126 MHz, $CDCl_3$) δ = 148.4, 148.0, 147.9, 129.2, 128.7 (q, *J* = 32.6 Hz), 128.5, 125.4 (q, *J* = 3.7 Hz),124.3 (q, *J* = 271.8 Hz), 117.5, 112.8, 112.3, 47.2, 29.9, 13.7.

HRMS (+ p APCI) m/z: $[M+H]^+$ Calcd for $C_{19}H_{18}F_3N^+$: 318.1464; Found: 318.1467.

N-(2-(1-(4-(trifluoromethoxy)phenyl)cyclopropyl)allyl)aniline (28)

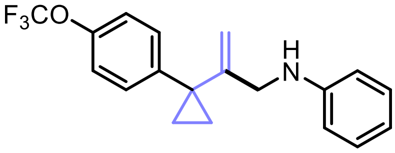

28

Following the **General Procedure E**, after concentration, the crude material was purified by flash column chromatography (SiO_2, Pentanes/Et_2O – 40:1) to furnish the title compound as a colorless oil (24.0 mg, 72% yield).

R_f = 0.35 (pentane/Et_2O = 40:1).

^{1}H NMR (500 MHz, $CDCl_3$) δ = 7.37 – 7.32 (m, 2H), 7.16 – 7.10 (m, 4H), 6.68 (tt, *J* = 7.3, 1.1 Hz, 1H), 6.47 – 6.43 (m, 2H), 5.17 (t, *J* = 1.4 Hz, 2H), 3.80 (brs, 1H), 3.68 (t, *J* = 1.5 Hz, 2H), 1.12 – 1.00 (m, 4H).

^{19}F NMR (471 MHz, $CDCl_3$) δ = -57.88.

^{13}C NMR (126 MHz, $CDCl_3$) δ = 148.8, 148.1, 147.7 (q, *J* = 2.0 Hz), 129.7, 129.2, 120.9, 120.6 (q, *J* = 256.8 Hz), 117.4, 112.8, 111.8, 47.2, 29.6, 13.3.

HRMS (+ p APCI) m/z: $[M+H]^+$ Calcd for $C_{19}H_{18}F_3NO^+$: 334.1413; Found: 334.1409.

N-(2-(1-(m-tolyl)cyclopropyl)allyl)aniline (29)

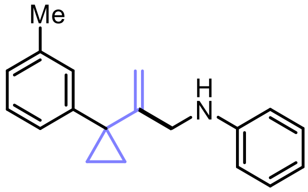

29

Following the **General Procedure E**, after concentration, the crude material was purified by flash column chromatography (SiO_2, Pentanes/Et_2O – 40:1) to furnish the title compound as a colorless oil (22.6 mg, 86% yield).

***R**f* = 0.35 (pentane/Et_2O = 40:1).

^{1}H NMR (500 MHz, $CDCl_3$) δ = 7.23 – 7.16 (m, 1H), 7.18 – 7.09 (m, 4H), 7.03 (dddt, *J* = 6.7, 1.9, 1.2, 0.7 Hz, 1H), 6.67 (tt, *J* = 7.3, 1.1 Hz, 1H), 6.51 – 6.44 (m, 2H), 5.16 (q, *J* = 1.3 Hz, 1H), 5.14 (q, *J* = 1.6 Hz, 1H), 3.83 (brs, 1H), 3.70 (t, *J* = 1.5 Hz, 2H), 2.35 (s, 3H), 1.08 – 0.99 (m, 4H).

^{13}C NMR (126 MHz, $CDCl_3$) δ = 149.3, 148.2, 143.5, 138.0, 129.2, 128.3, 127.2, 125.3, 117.2, 112.8, 111.0, 47.3, 30.0, 21.5, 13.2.

HRMS (+ p ESI) m/z: $[M+H]^+$ Calcd for $C_{19}H_{22}N^+$: 264.1747; Found: 264.1747.

N-(2-(1-(3-methoxyphenyl)cyclopropyl)allyl)aniline (30)

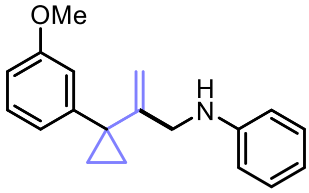

30

Following the **General Procedure E**, after concentration, the crude material was purified by flash column chromatography (SiO_2, Pentanes/Et_2O – 40:1) to furnish the title compound as a colorless oil (17.3 mg, 62% yield).

***R**f* = 0.30 (pentane/Et_2O = 30:1).

^{1}H NMR (500 MHz, $CDCl_3$) δ = 7.22 (t, *J* = 7.9 Hz, 1H), 7.16 – 7.10 (m, 2H), 6.93 (ddt, *J* = 7.6, 1.8, 0.9 Hz, 1H), 6.90 – 6.88 (m, 1H), 6.77 (ddd, *J* = 8.2, 2.6, 0.9 Hz, 1H), 6.67 (tq, *J* = 7.3, 1.0 Hz, 1H), 6.48 (dq, *J* = 7.6, 1.0 Hz, 2H), 5.16 (q, *J* = 1.2 Hz, 1H), 5.15 (q, *J* = 1.3 Hz, 1H), 3.83 (brs, 1H), 3.80 (s, 3H), 3.71 (q, *J* = 1.3 Hz, 2H), 1.07 – 1.02 (m, 4H).

^{13}C NMR (126 MHz, $CDCl_3$) δ = 158.8, 149.5, 146.2, 145.5, 129.7, 129.2, 123.4, 117.6, 113.8,

113.1, 112.6, 111.5, 55.2, 47.8, 29.3, 12.6.

HRMS (+ p APCI) m/z: $[M+H]^+$ Calcd for $C_{19}H_{21}NO^+$: 280.1697; Found: 2, n.d.80.1700.

N-(2-(1-(naphthalen-2-yl)cyclopropyl)allyl)aniline (32)

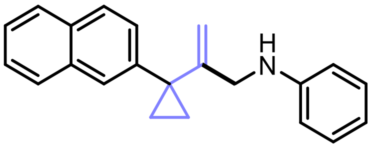

32

Following the **General Procedure E**, after concentration, the crude material was purified by flash column chromatography (SiO_2, Pentanes/Et_2O – 40:1) to furnish the title compound as a colorless oil (24.1 mg, 81% yield).

R_f = 0.35 (pentane/Et_2O = 40:1).

^{1}H NMR (500 MHz, $CDCl_3$) δ = 7.84 – 7.78 (m, 3H), 7.76 (dd, *J* = 1.7, 0.8 Hz, 1H), 7.52 – 7.43 (m, 3H), 7.14 – 7.09 (m, 2H), 6.67 (tt, *J* = 7.3, 1.1 Hz, 1H), 6.50 – 6.44 (m, 2H), 5.24 (q, *J* = 1.3 Hz, 1H), 5.20 (q, *J* = 1.5 Hz, 1H), 3.83 (brs, 1H), 3.73 (t, *J* = 1.5 Hz, 2H), 1.16 – 1.14 (m, 4H).

^{13}C NMR (126 MHz, $CDCl_3$) δ = 149.1, 148.2, 141.1, 133.5, 132.3, 129.2, 128.1, 127.7, 127.6, 127.1, 126.5, 126.2, 125.7, 117.3, 112.8, 111.3, 47.3, 30.3, 13.2.

HRMS (+ p ESI) m/z: $[M+H]^+$ Calcd for $C_{22}H_{22}N^+$: 300.1747; Found: 300.1740.

N-(2-(1-(thiophen-3-yl)cyclopropyl)allyl)aniline (33)

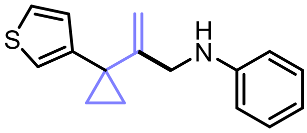

33

Following the **General Procedure E**, after concentration, the crude material was purified by flash column chromatography (SiO_2, Pentanes/Et_2O – 40:1) to furnish the title compound as a colorless oil (10.9 mg, 43% yield).

R_f = 0.40 (pentane/Et_2O = 40:1).

^{1}H NMR (500 MHz, $CDCl_3$) δ = 7.24 (dd, *J* = 4.9, 3.0 Hz, 1H), 7.16 – 7.11 (m, 2H), 6.99 (dd, *J* = 3.0, 1.4 Hz, 1H), 6.96 (dd, *J* = 5.0, 1.3 Hz, 1H), 6.67 (tt, *J* = 7.3, 1.2 Hz, 1H), 6.53 – 6.46 (m, 2H), 5.16 (t, *J* = 1.4 Hz, 2H), 3.84 (brs, 1H), 3.75 (t, *J* = 1.5 Hz, 2H), 1.05 – 0.98 (m, 4H).

^{13}C NMR (126 MHz, $CDCl_3$) δ = 148.82, 148.22, 145.42, 129.21, 127.63, 125.68, 120.54, 117.30, 112.80, 111.53, 47.36, 25.98, 13.93.
HRMS (+ p APCI) m/z: $[M+H]^+$ Calcd for $C_{16}H_{17}NS^+$: 256.1154; Found: 256.1156.

1-methoxy-4-((3-(1-(p-tolyl)cyclopropyl)but-3-en-2-yl)oxy)benzene (34)

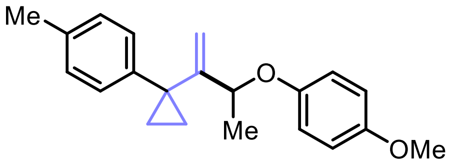

34

Following the **General Procedure E**, after concentration, the crude material was purified by flash column chromatography (SiO_2, Pentanes/Et_2O – 20:1) to furnish the title compound as a colorless oil (23.1 mg, 75% yield).
R_f = 0.25 (pentane/Et_2O = 20:1).
^{1}H NMR (500 MHz, $CDCl_3$) δ = 7.25 – 7.19 (m, 2H), 7.12 – 7.07 (m, 2H), 6.71 – 6.65 (m, 2H), 6.56 – 6.50 (m, 2H), 5.22 (t, *J* = 1.3 Hz, 1H), 5.14 (d, *J* = 1.5 Hz, 1H), 4.52 (qd, *J* = 6.4, 1.1 Hz, 1H), 3.73 (s, 3H), 2.34 (s, 3H), 1.37 (d, *J* = 6.4 Hz, 3H), 1.21 – 1.12 (m, 2H), 0.93 – 0.88 (m, 1H), 0.86 – 0.81 (m, 1H).
^{13}C NMR (126 MHz, $CDCl_3$) δ = 153.6, 152.4, 152.1, 140.7, 136.2, 129.1, 129.0, 116.8, 114.4, 110.5, 75.3, 55.8, 29.5, 22.0, 21.1, 14.0, 12.4.
HRMS (+ p APCI) m/z: $[M+H]^+$ Calcd for $C_{21}H_{24}O_2^+$: 309.1850; Found: 309.1850.

1-methoxy-4-((3-(1-(4-(trifluoromethyl)phenyl)cyclopropyl)but-3-en-2-yl)oxy)benzene (35)

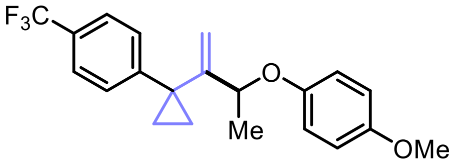

35

Following the **General Procedure E**, after concentration, the crude material was purified by flash column chromatography (SiO_2, Pentanes/Et_2O – 20:1) to furnish the title compound as a colorless oil (28.3 mg, 78% yield).
R_f = 0.25 (pentane/Et_2O = 20:1).
^{1}H NMR (500 MHz, $CDCl_3$) δ = 7.54 – 7.50 (m, 2H), 7.43 – 7.39 (m, 2H), 6.71 – 6.67 (m, 2H), 6.55 – 6.51 (m, 2H), 5.36 (t, *J* = 1.2 Hz, 1H), 5.24 (d, *J* = 1.2 Hz, 1H), 4.52 (qd, *J* = 6.4, 1.1 Hz,

1H), 3.74 (s, 3H), 1.37 (d, J = 6.4 Hz, 3H), 1.27 – 1.22 (m, 1H), 1.16 (ddd, J = 9.4, 6.1, 4.4 Hz, 1H), 1.04 (ddd, J = 9.6, 6.3, 4.4 Hz, 1H), 0.92 (ddd, J = 9.4, 6.3, 4.5 Hz, 1H).

^{19}F NMR (471 MHz, $CDCl_3$) δ = -62.35.

^{13}C NMR (126 MHz, $CDCl_3$) δ = 153.9, 151.9, 151.5, 148.0, 129.1, 128.7 (q, J = 32.3 Hz), 125.4 (q, J = 271.8 Hz), 125.2 (q, J = 3.8 Hz), 117.0, 114.4, 112.8, 75.6, 55.7, 29.7, 21.8, 14.1, 13.1.

HRMS (+ p APCI) m/z: $[M+H]^+$ Calcd for $C_{21}H_{21}F_3O_2^+$: 363.1566; Found: 363.1566.

ethyl 1-(3-(phenylamino)prop-1-en-2-yl)cyclopropane-1-carboxylate (38)

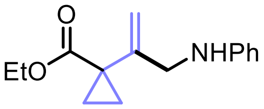

38

Following the **General Procedure E**, after concentration, the crude material was purified by flash column chromatography (SiO_2, Pentanes/Et_2O – 4:1) to furnish the title compound as a colorless oil (6.9 mg, 28% yield).

R_f = 0.4 (pentane/Et_2O = 4:1).

^{1}H NMR (400 MHz, $CDCl_3$) δ = 7.20 – 7.13 (m, 2H), 6.69 (tt, J = 7.3, 1.1 Hz, 1H), 6.66 – 6.60 (m, 2H), 5.24 (q, J = 1.6 Hz, 1H), 5.08 (q, J = 1.4 Hz, 1H), 4.15 (q, J = 7.1 Hz, 2H), 4.06 (brs, 1H), 3.93 (d, J = 4.2 Hz, 2H), 1.38 (q, J = 3.9 Hz, 2H), 1.35 – 1.33 (m, 1H), 1.28 (t, J = 7.1 Hz, 3H), 1.03 – 0.96 (m, 2H).

^{13}C NMR (101 MHz, $CDCl_3$) δ = 174.1, 148.2, 144.5, 129.2, 117.2, 114.3, 112.7, 61.1, 48.0, 27.8, 15.8, 14.2.

HRMS (+ p APCI) m/z: $[M+H]^+$ Calcd for $C_{15}H_{19}NO_2^+$: 246.1489; Found: 246.1492.

N-(2-cyclopropyl-3-phenylallyl)aniline (40)

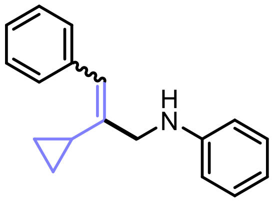

40

Following the **General Procedure E**, after concentration, the crude material was purified by flash column chromatography (SiO_2, Pentanes/Et_2O – 40:1) to furnish the title compound as a

colorless oil (19.2 mg, 77% yield, 1:2.7 E/Z).

***R**$_f$* = 0.35 (pentane/Et_2O = 40:1).

^{1}H NMR (400 MHz, $CDCl_3$) δ = 7.44 – 7.40 (m, 1H), 7.35 – 7.28 (m, 3H), 7.27 – 7.14 (m, 7H), 6.72 (tq, *J* = 7.4, 1.1 Hz, 1.6H), 6.67 – 6.60 (m, 1H), 6.61 (s, 0.5H, minor), 6.62 – 6.54 (m, 2H), 6.43 (s, 1H, major), 3.85 (s, 3H, major, CH_2, NH), 3.67 (s, 0.24H, minor, NH), 3.64 (d, *J* = 1.4 Hz, 1H, minor, CH_2), 1.85 (ttd, *J* = 8.6, 5.5, 1.3 Hz, 0.5H, CH), 1.61 (ttd, *J* = 8.2, 5.3, 1.1 Hz, 1H, major, CH), 0.83 – 0.72 (m, 3H), 0.67 – 0.57 (m, 3H).

^{13}C NMR (101 MHz, $CDCl_3$, major) δ = 148.3, 140.4, 137.2, 129.3, 129.3, 128.7, 128.4, 128.0, 126.7, 117.5, 113.0, 47.8, 16.7, 6.7, 6.2.

^{13}C NMR (101 MHz, $CDCl_3$, minor) δ = 146.5, 138.7, 137.7, 129.3, 128.7, 128.4, 127.8, 126.6, 126.4, 117.5, 112.5, 44.4, 12.5, 6.7, 6.2.

HRMS (+ p ESI) m/z: $[M+H]^+$ Calcd for $C_{18}H_{22}N^+$: 250.1590; Found: 250.1592.

***N*-(2-cyclopropyl-3-(4-fluorophenyl)allyl)aniline (41)**

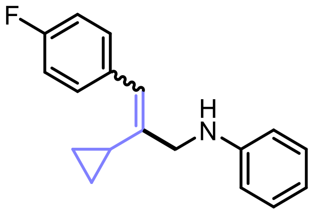

41

Following the **General Procedure E**, after concentration, the crude material was purified by flash column chromatography (SiO_2, Pentanes/Et_2O – 40:1) to furnish the title compound as a colorless oil (17.3 mg, 65% yield, 1:2.2 E/Z).

***R**$_f$* = 0.35 (pentane/Et_2O = 40:1).

^{1}H NMR (700 MHz, $CDCl_3$) δ = 7.39 – 7.36 (m, 1H), 7.24 – 7.20 (m, 2H), 7.20 – 7.16 (m, 3H), 7.03 – 6.97 (m, 3H), 6.72 (tdt, *J* = 7.2, 5.0, 1.1 Hz, 1.5H), 6.64 – 6.62 (m, 1H), 6.58 – 6.56 (m, 2H), 6.55 (s, 1H, minor), 6.38 (s, 1H, major), 3.83 (brs, 1H, NH), 3.79 (s, 2H, major), 3.64 (d, *J* = 1.4 Hz, 2H, minor), 1.77 (ttd, *J* = 8.4, 5.6, 1.3 Hz, 1H, minor), 1.59 (ttd, *J* = 8.3, 5.4, 1.2 Hz, 1H, major), 0.79 – 0.74 (m, 3H), 0.62 – 0.58 (m, 3H).

^{19}F NMR (659 MHz, $CDCl_3$) δ = - 115.55 (major), -155.90 (minor).

^{13}C NMR (176 MHz, $CDCl_3$, major) δ = 161.72 (d, *J* = 246.1 Hz), 148.15, 140.23, 133.19 (d, *J* = 3.4 Hz), 130.26 (d, *J* = 7.9 Hz), 129.33, 125.56, 117.66, 115.30 (d, *J* = 21.2 Hz), 112.95, 44.28, 16.71, 6.15.

^{13}**C NMR** (176 MHz, $CDCl_3$, minor) δ = 161.72 (d, J = 246.1 Hz), 148.15, 140.23, 133.19 (d, J = 3.4 Hz), 130.26 (d, J = 7.9 Hz), 129.33, 125.56, 117.66, 115.30 (d, J = 21.2 Hz), 112.95, 44.28, 16.71, 6.15.

HRMS (+ p ESI) m/z: $[M+H]^+$ Calcd for $C_{18}H_{18}FN^+$: 268.1493; Found: 268.1501.

N-(3-(4-chlorophenyl)-2-cyclopropylallyl)aniline (42)

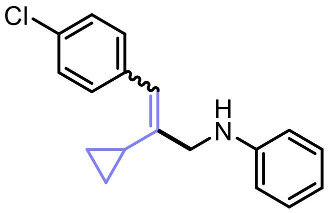

42

Following the **General Procedure E**, after concentration, the crude material was purified by flash column chromatography (SiO_2, Pentanes/Et_2O – 40:1) to furnish the title compound as a colorless oil (16.1 mg, 57% yield, 1:3.2 E/Z).

R_f = 0.35 (pentane/Et_2O = 40:1).

^{1}**H NMR** (400 MHz, $CDCl_3$) δ = 7.36 – 7.32 (m, 1H, minor), 7.30 –7.25 (m, 2H), 7.22 – 7.15 (m, 2H), 6.75 – 6.70 (m, 2H), 6.65 – 6.60 (m, 1H, minor), 6.59 – 6.55 (m, 2H, major), 6.54 (s, 1H, minor), 6.37 (s, 1H, major), 3.82 (brs, 1H), 3.79 (s, 2H, major), 3.65 (d, J = 1.5 Hz, 2H, minor), 1.77 (ttd, J = 8.5, 5.6, 1.3 Hz, 1H, minor), 1.60 (ttd, J = 8.3, 5.4, 1.2 Hz, 1H, major), 0.81 – 0.75 (m, 3H), 0.64 – 0.58 (m, 3H).

^{13}**C NMR** (101 MHz, $CDCl_3$, major) δ = 148.11, 141.17, 135.62, 132.55, 129.98, 129.33, 128.57, 125.42, 117.71, 112.96, 44.30, 16.77, 6.26.

^{13}**C NMR** (101 MHz, $CDCl_3$, minor) δ = 148.20, 139.46, 136.11, 132.08, 130.54, 129.33, 128.17, 126.34, 117.63, 112.96, 47.84, 12.49, 6.74.

HRMS (+ p ESI) m/z: $[M+H]^+$ Calcd for $C_{18}H_{18}N^{35}Cl^+$: 284.1201; Found: 284.1205.

(Z)-N-(2-cyclopropyl-3-(4-(trifluoromethoxy)phenyl)allyl)aniline (43-major)

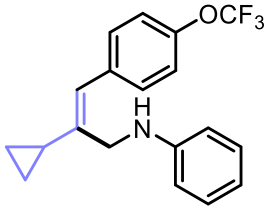

43-major

Following the **General Procedure E**, after concentration, the crude material was purified by flash column chromatography (SiO_2, Pentanes/Et_2O – 40:1) to furnish the title compound as a colorless oil (19.3 mg, 58% yield).

R_f = 0.35 (pentane/Et_2O = 40:1).

^{1}H NMR (500 MHz, $CDCl_3$) δ = 7.29 – 7.26 (m, 2H), 7.20 – 7.12 (m, 4H), 6.73 (tt, *J* = 7.3, 1.1 Hz, 1H), 6.61 – 6.54 (m, 2H), 6.39 (s, 1H), 3.83 (brs, 1H), 3.79 (s, 2H), 1.61 (ttd, *J* = 8.3, 5.4, 1.1 Hz, 1H), 0.82 – 0.74 (m, 2H), 0.65 – 0.58 (m, 2H).

^{19}F NMR (471 MHz, $CDCl_3$) δ = -57.83.

^{13}C NMR (126 MHz, $CDCl_3$) δ = 148.1, 147.9 (d, *J* = 2.0 Hz), 141.3, 135.9, 130.0, 129.4, 125.2, 120.9, 120.6(q, *J* = 257.0 Hz), 117.8, 113.0, 44.2, 16.8, 6.3.

HRMS (+ p ESI) m/z: $[M+H]^+$ Calcd for $C_{19}H_{18}F_3NO^+$: 334.1413; Found: 334.1416.

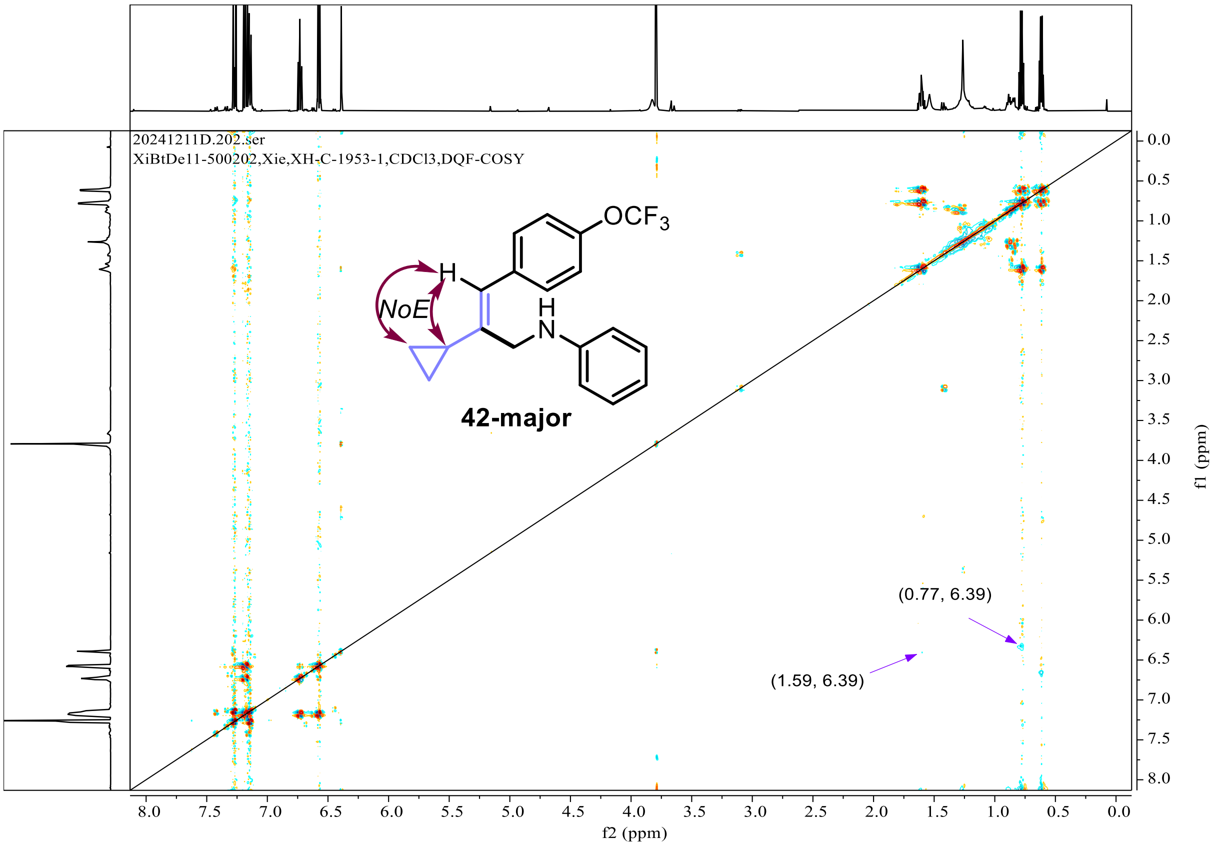

(E)-N-(2-cyclopropyl-3-(4-(trifluoromethoxy)phenyl)allyl)aniline (43-major)

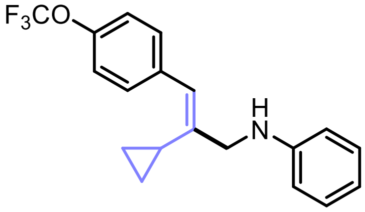

43-minor

Following the **General Procedure E**, after concentration, the crude material was purified by flash column chromatography (SiO_2, Pentanes/Et_2O – 40:1) to furnish the title compound as a colorless oil (6.7 mg, 20% yield).

$\boldsymbol{R_f}$ = 0.35 (pentane/Et_2O = 40:1).

^{1}H NMR (500 MHz, $CDCl_3$) δ = 7.44 – 7.40 (m, 2H), 7.20 – 7.14 (m, 4H), 6.72 (tt, J = 7.3, 1.1 Hz, 1H), 6.64 – 6.60 (m, 2H), 6.56 (s, 1H), 3.83 (brs, 1H), 3.64 (d, J = 1.5 Hz, 2H), 1.78 (ttd, J = 8.6, 5.6, 1.3 Hz, 1H), 0.81 – 0.76 (m, 2H), 0.64 – 0.60 (m, 2H).

^{19}F NMR (471 MHz, $CDCl_3$) δ = -57.83.

^{13}C NMR (126 MHz, $CDCl_3$) δ = 148.2, 147.6 (d, J = 1.7 Hz), 139.6, 136.4, 130.5, 129.4, 126.0, 120.5, 120.6 (q, J = 256.7 Hz), 117.7, 112.9, 47.7, 12.5, 6.7.

HRMS (+ p ESI) m/z: $[M+H]^+$ Calcd for $C_{19}H_{18}F_3NO^+$: 334.1413; Found: 334.1418.

(Z)-N-(2-cyclopropyl-3-(3-methoxyphenyl)allyl)aniline (44-major)

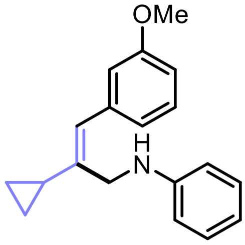

44-major

Following the **General Procedure E**, after concentration, the crude material was purified by flash column chromatography (SiO_2, Pentanes/Et_2O – 20:1) to furnish the title compound as a colorless oil (14.0 mg, 50% yield).

$\boldsymbol{R_f}$ = 0.35 (pentane/Et_2O = 20:1).

^{1}H NMR (500 MHz, $CDCl_3$) δ = 7.21 (t, J = 7.9 Hz, 1H), 7.18 – 7.14 (m, 2H), 6.87 – 6.80 (m, 2H), 6.77 (ddd, J = 8.3, 2.6, 1.0 Hz, 1H), 6.71 (tt, J = 7.3, 1.1 Hz, 1H), 6.61 – 6.55 (m, 2H), 6.41 (s, 1H), 3.86 (brs, 1H), 3.83 (s, 1H), 3.67 (s, 3H), 1.61 (ttd, J = 8.3, 5.4, 1.1 Hz, 1H), 0.80 – 0.73 (m, 2H), 0.65 – 0.58 (m, 2H).

^{13}C NMR (126 MHz, $CDCl_3$) δ = 159.6, 148.2, 140.6, 138.6, 129.4, 129.3, 126.6, 121.2, 117.5, 113.6, 112.9, 112.9, 55.1, 44.4, 16.8, 6.2.

HRMS (+ p ESI) m/z: $[M+H]^+$ Calcd for $C_{19}H_{21}NO^+$: 280.1697; Found: 280.1699.

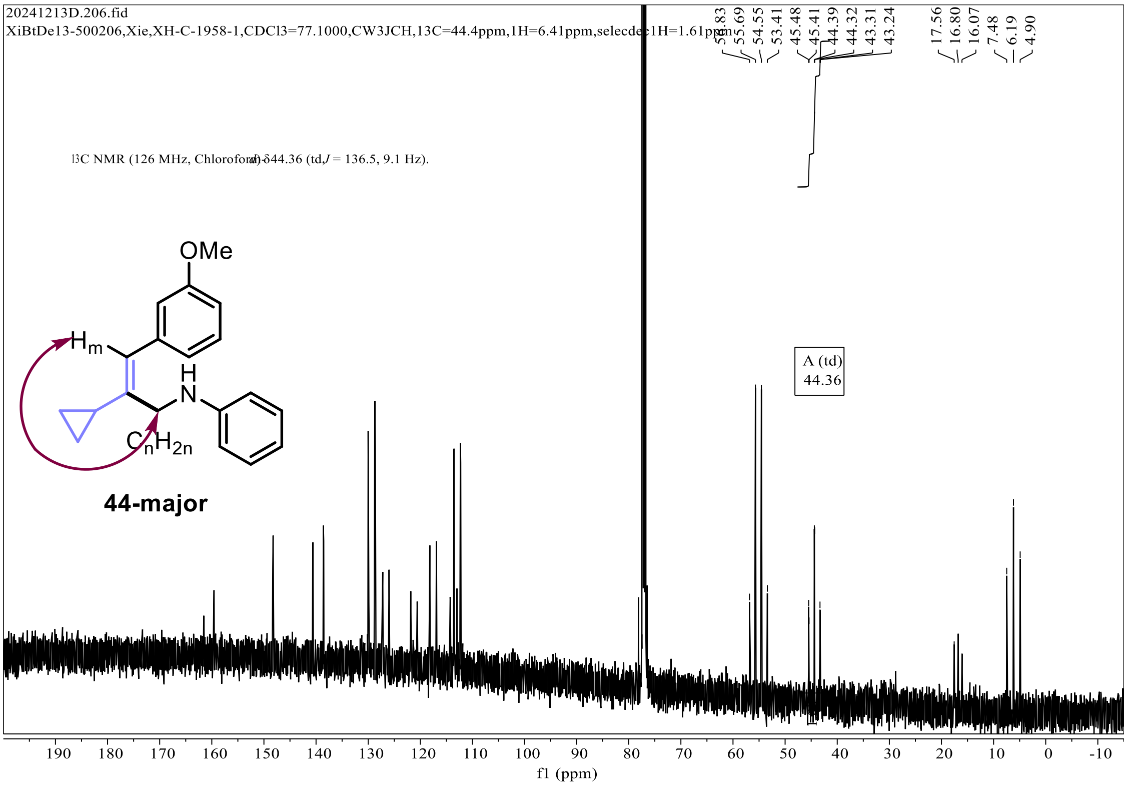

The coupling constant of C_n with H_m is 9.1 (relatively larger), revealing that the structure is trans.

(E)-N-(2-cyclopropyl-3-(3-methoxyphenyl)allyl)aniline (44-minor)

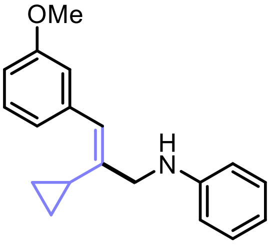

44-minor

Following the **General Procedure E**, after concentration, the crude material was purified by flash column chromatography (SiO_2, Pentanes/Et_2O – 20:1) to furnish the title compound as a colorless oil (5.3 mg, 19% yield).

R_f = 0.32 (pentane/Et_2O = 20:1).

^{1}H NMR (500 MHz, $CDCl_3$) δ = 7.24 (dd, J = 8.3, 7.5 Hz, 1H), 7.21 – 7.16 (m, 2H), 7.03 – 6.97 (m, 2H), 6.77 (ddd, J = 8.3, 2.6, 1.0 Hz, 1H), 6.71 (tt, J = 7.3, 1.1 Hz, 1H), 6.65 – 6.61 (m,

2H), 6.57 (s, 1H), 3.81 (s, 3H), 3.67 (s, 1H), 3.62 (d, J = 1.4 Hz, 2H), 1.86 (ttd, J = 8.6, 5.6, 1.2 Hz, 1H), 0.82 – 0.71 (m, 3H), 0.67 – 0.58 (m, 2H).

^{13}C NMR (126 MHz, $CDCl_3$) δ = 159.3, 148.3, 139.1, 138.9, 129.32, 129.29, 129.0, 127.6, 121.9, 117.5, 114.8, 112.9, 112.1, 55.3, 47.7, 12.6, 6.7.

HRMS (+ p ESI) m/z: $[M+H]^+$ Calcd for $C_{19}H_{21}NO^+$: 280.1697; Found: 280.1699.

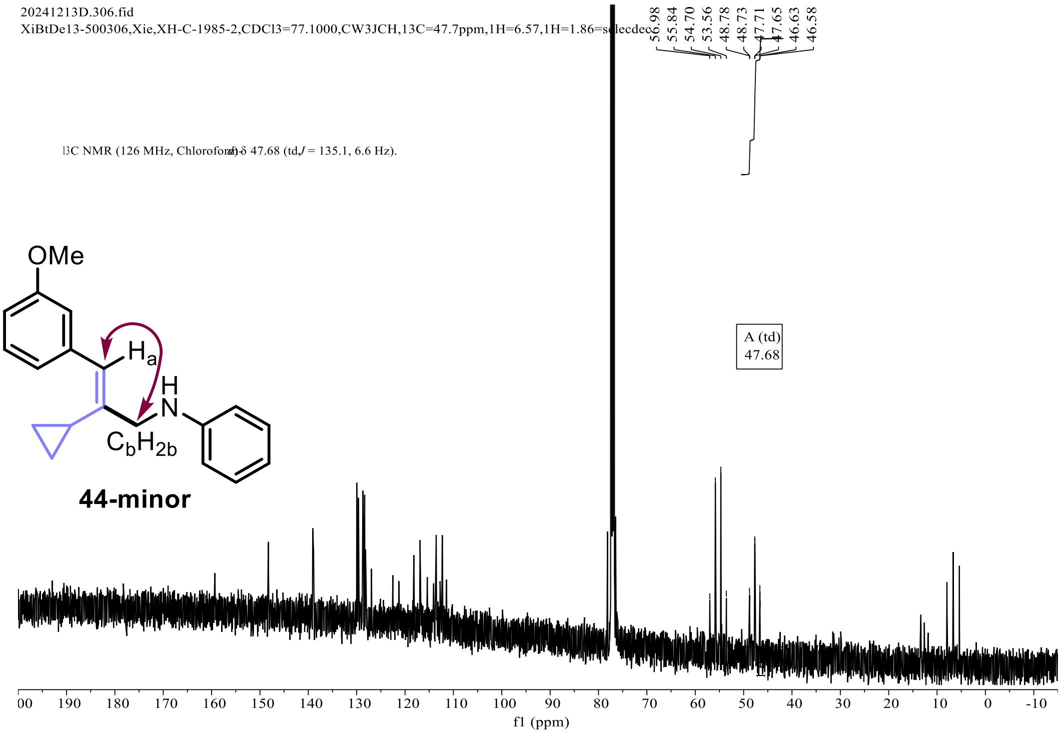

The coupling constant of C_n with H_m is 9.1 (relatively smaller), revealing that the structure is cis.

N-(2-cyclopropyl-3-(2,3-dihydronaphthalen-2-yl)allyl)aniline (45)

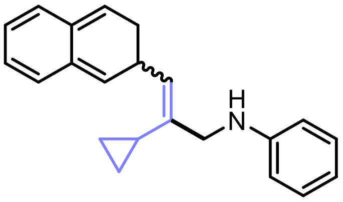

45

Following the **General Procedure E**, after concentration, the crude material was purified by flash column chromatography (SiO_2, Pentanes/Et_2O – 40:1) to furnish the title compound as a colorless oil (21.6 mg, 72% yield, 1:2.3 E/Z).

R_f = 0.35 (pentane/Et_2O = 40:1).

^{1}H NMR (400 MHz, $CDCl_3$) δ = 7.87 (s, 1H, minor), 7.83 – 7.76 (m, 3H), 7.73 – 7.68 (m, 2H), 7.57 (dd, J = 8.5, 1.8 Hz, 1H, minor), 7.49 – 7.41 (m, 3H), 7.39 (dd, J = 8.5, 1.6 Hz, 1H, major), 7.24 – 7.14 (m, 3H), 6.77 – 6.70 (m, 2H), 6.69 – 6.65 (m, 1H, minor), 6.61 – 6.57 (m, 3H), 3.93

(s, 2H, major), 3.89 (brs, 1H), 3.70 (d, *J* = 1.4 Hz, 2H, minor), 1.94 (ttd, *J* = 8.5, 5.6, 1.3 Hz, 1H, minor), 1.66 (ttd, *J* = 8.3, 5.4, 1.2 Hz, 1H, major), 0.85 – 0.77 (m, 3H), 0.70 – 0.62 (m, 3H).

^{13}C NMR (101 MHz, $CDCl_3$, major) δ = 148.3, 141.0, 134.7, 133.5, 132.3, 129.3, 128.1, 127.9, 127.6, 127.4, 127.1, 126.6, 126.2, 125.8, 117.6, 113.0, 44.5, 16.8, 6.3.

^{13}C NMR (101 MHz, $CDCl_3$, major) δ = 148.3, 139.2, 135.2, 133.4, 132.2, 129.4, 128.0, 127.9, 127.8, 127.7, 127.7, 127.4, 126.1, 125.7, 117.6, 113.0, 47.9, 12.7, 6.8.

HRMS (+ p ESI) m/z: $[M+H]^+$ Calcd for $C_{22}H_{22}N^+$: 300.1747; Found: 300.1753.

N-(2-cyclopropyl-3-phenylallyl)-4-methylaniline (46)

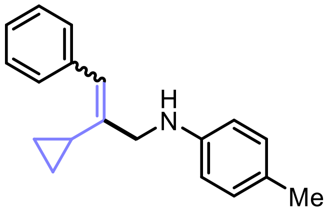

46

Following the **General Procedure E**, after concentration, the crude material was purified by flash column chromatography (SiO_2, Pentanes/Et_2O – 40:1) to furnish the title compound as a colorless oil (18.4 mg, 70% yield, 1:2 E/Z).

R_f = 0.35 (pentane/Et_2O = 40:1).

^{1}H NMR (500 MHz, $CDCl_3$) δ = 7.44 – 7.41 (m, 1H, minor), 7.35 – 7.28 (m, 3H), 7.27 – 7.24 (m, 2H), 7.23 – 7.19 (m, 1.6H), 7.03 – 6.95 (m, 3H), 6.60 (s, 1H, minor), 6.58 – 6.55 (m, 1H, minor), 6.53 – 6.49 (m, 2H, major), 6.42 (s, 1H, major), 3.82 (s, 2H, major), 3.73 (brs, 1H), 3.61 (d, *J* = 1.4 Hz, 1H, minor), 2.25 (s, 4.5H), 1.85 (ttd, *J* = 8.6, 5.6, 1.2 Hz, 1H, minor), 1.61 (ttd, *J* = 8.4, 5.4, 1.2 Hz, 1H, major), 0.81 – 0.72 (m, 3H), 0.62 (ddt, *J* = 6.2, 5.3, 3.9 Hz, 3H).

^{13}C NMR (126 MHz, $CDCl_3$, mix) δ = 146.06, 146.03, 140.51, 138.85, 137.71, 137.23, 129.79, 129.77, 129.26, 128.68, 128.40, 128.01, 127.62, 126.69, 126.48, 126.38, 113.08, 48.09, 44.63, 20.46, 16.73, 12.54, 6.66, 6.16.

HRMS (+ p ESI) m/z: $[M+H]^+$ Calcd for $C_{19}H_{22}N^+$: 264.1747; Found: 264.1752.

1-((3-cyclopropyl-4-phenylbut-3-en-2-yl)oxy)-4-methoxybenzene (47)

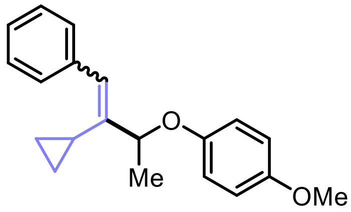

47

Following the **General Procedure E**, after concentration, the crude material was purified by flash column chromatography (SiO_2, Pentanes/Et_2O – 20:1) to furnish the title compound as a colorless oil (20.0 mg, 68% yield).

***R**$_f$* = 0.35 (pentane/Et_2O = 20:1).

^{1}H NMR (400 MHz, $CDCl_3$) δ = 7.45 – 7.37 (m, 2H), 7.40 – 7.21 (m, 4H), 7.24 – 7.15 (m, 1H), 7.12 (ddt, J = 7.8, 1.4, 0.8 Hz, 1H), 6.89 – 6.75 (m, 4H), 6.72 – 6.63 (m, 2H), 6.60 – 6.51 (m, 1H), 6.20 (s, 1H, minor), 5.25 (q, J = 6.5 Hz, 1H, minor), 4.58 (qd, J = 6.5, 1.0 Hz, 1H, major), 3.76 (s, 3H, major), 3.72 (s, 3H, minor), 1.73 – 1.60 (m, 5H), 1.55 (d, J = 6.4 Hz, 3H, minor), 0.87 – 0.70 (m, 4H), 0.67 – 0.40 (m, 4H).

^{13}C NMR (101 MHz, $CDCl_3$, major) δ = 153.95, 152.41, 141.65, 137.48, 129.45, 127.88, 126.49, 123.59, 117.26, 114.64, 72.41, 55.78, 21.91, 11.82, 7.26, 7.09.

^{13}C NMR (101 MHz, $CDCl_3$, minor) δ = 153.72, 151.93, 144.47, 137.30, 128.83, 128.36, 127.35, 126.78, 116.87, 114.40, 72.41, 55.73, 20.91, 11.28, 8.55, 4.96.

HRMS (+ p ESI) m/z: $[M+H]^+$ Calcd for $C_{20}H_{22}O_2^+$: 295.1693; Found: 295.1690.

ethyl (Z)-3-cyclopropyl-4-(phenylamino)but-2-enoate (48)

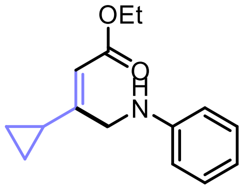

48

Following the **General Procedure E**, after concentration, the crude material was purified by flash column chromatography (SiO_2, Pentanes/Et_2O – 4:1) to furnish the title compound as a colorless oil (7.6 mg, 31% yield).

***R**$_f$* = 0.35 (pentane/Et_2O = 4:1).

^{1}H NMR (400 MHz, $CDCl_3$, major-Z) δ = 7.20 – 7.13 (m, 2H), 6.73 – 6.63 (m, 3H), 5.59 (d, J = 0.9 Hz, 1H), 4.28 (s, 2H), 4.24 (s, brH), 4.17 (q, J = 7.1 Hz, 2H), 1.63 (ttd, J = 8.3, 5.3, 0.9 Hz, 1H), 1.28 (t, J = 7.1 Hz, 3H), 0.88 – 0.78 (m, 2H), 0.67 – 0.58 (m, 2H).

^{13}C NMR (101 MHz, $CDCl_3$) δ = 166.7, 163.0, 148.3, 129.3, 117.7, 114.0, 113.3, 60.0, 44.4, 16.7, 14.4, 8.3.

HRMS (+ p ESI) m/z: $[M+H]^+$ Calcd for $C_{15}H_{19}NO_2^+$: 246.1493; Found: 246.1493.

A radical addition product 48' without cyclization was isolated:

ethyl (Z)-6-bromo-3-((phenylamino)methyl)hex-3-enoate (48')

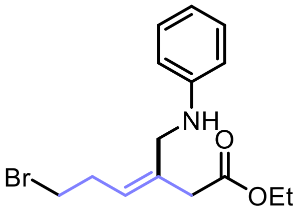

47'

Following the **General Procedure E**, after concentration, the crude material was purified by flash column chromatography (SiO_2, Pentanes/Et_2O – 4:1) to furnish the title compound as a colorless oil (9.1 mg, 28% yield).

R_f = 0.35 (pentane/Et_2O = 4:1).

^{1}H NMR (400 MHz, $CDCl_3$) δ = 7.20 – 7.12 (m, 2H), 6.70 (tt, *J* = 7.4, 1.1 Hz, 1H), 6.63 – 6.59 (m, 2H), 5.66 (tq, *J* = 8.1, 7.3, 1.5, 0.7 Hz, 1H), 4.13 (q, *J* = 7.2 Hz, 2H), 3.99 (brs, 1H), 3.81 (q, *J* = 1.2 Hz, 2H), 3.38 (t, *J* = 7.0 Hz, 2H), 3.14 (s, 2H), 2.67 (qt, *J* = 7.0, 1.1 Hz, 2H), 1.26 (t, *J* = 7.1 Hz, 3H).

^{13}C NMR (101 MHz, $CDCl_3$) δ = 171.2, 148.2, 133.1, 129.3, 126.8, 117.6, 113.0, 61.0, 50.5, 34.9, 32.0, 31.5, 14.3.

HRMS (+ p ESI) m/z: $[M+H]^+$ Calcd for $C_{15}H_{20}{}^{79}BrNO_2^+$: 326.0750; Found: 326.0754.

7. Synthetic Application.

7.1 Larger-scale Preparation of 3a

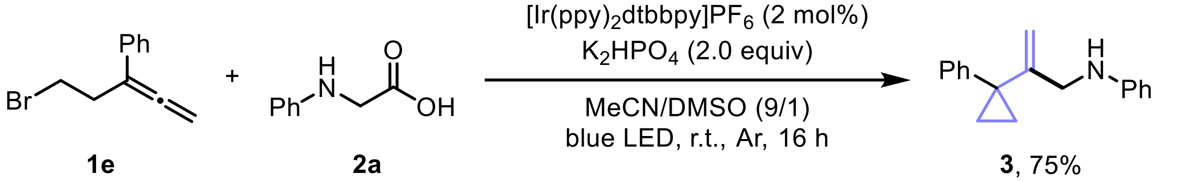

An oven-dried 50 mL Schlenk tube was charged with phenyl glycine **2a** (302 mg, 2.0 mmol, 2.0 equiv), [Ir(ppy)$_2$dtbbpy]PF$_6$ (18 mg, 0.02 mmol, 2 mol %), K_2HPO_4 (348 mg, 2.0 mmol, 2 equiv.). The Schlenk tube was put on vacuum and backfilled with argon three times. Afterwards MeCN (18 mL) and DMSO (2.0 mL) was added by syringe under a flow of argon. Sequentially, allenyl bromide **1e** (223 mg, 1.0 mmol, 1.0 equiv.) in 8 mL MeCN was added by syringe under a flow of argon. The Schlenk tube was sealed by screw cap and the resulting mixture was placed approximately 2.5 inch from blue led strips and irradiated and stirred for 16 hours at room temperature. After filtration through a pad of Celite and evaporation of the solvents under reduced pressure. The residue was purified by flash column chromatography on silica gel afford the desired vinyl cyclopropane (**VCP**) products **3** (187 mg, 75% yield) as a colorless oil.

5 mmol scale:

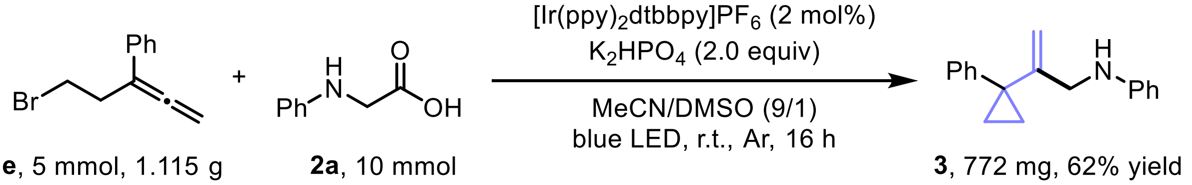

An oven-dried 50 mL Schlenk tube was charged with phenyl glycine **2a** (10.0 mmol, 2.0 equiv), [Ir(ppy)$_2$dtbbpy]PF$_6$ (2 mol %), K_2HPO_4 (2 equiv.). The Schlenk tube was put on vacuum and backfilled with argon three times. Afterwards MeCN (90 mL) and DMSO (10 mL) was added by syringe under a flow of argon. Sequentially, allenyl bromide **1e** (1.115 g, 5.0 mmol, 1.0 equiv.) in 8 mL MeCN was added by syringe under a flow of argon. The Schlenk tube was sealed by screw cap and the resulting mixture was placed approximately 2.5 inch from Kessil Led lamp (Figure S3) and irradiated and stirred for 16 hours at room temperature. After filtration through a pad of Celite and evaporation of the solvents under reduced pressure. The residue was purified by flash column chromatography on silica gel afford the desired vinyl cyclopropane (**VCP**) products **3** (772 mg, 62% yield) as a colorless oil.

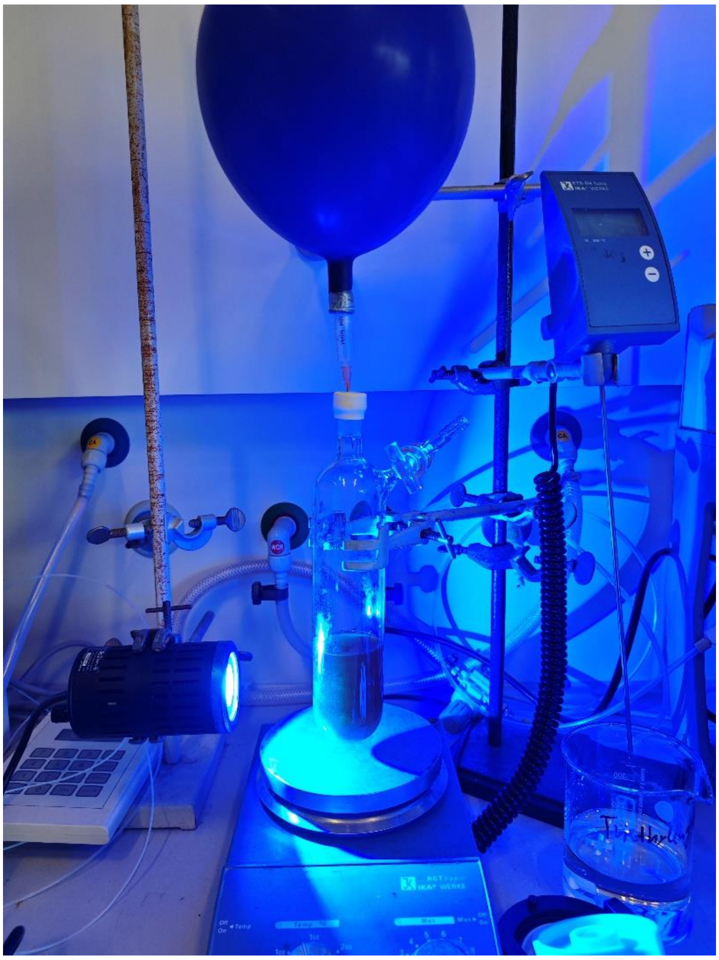

Figure S3. 5 mmol Scale reaction irradiated by Kessil LED Lamp.

7.2 Ring-closing Metathesis of 12

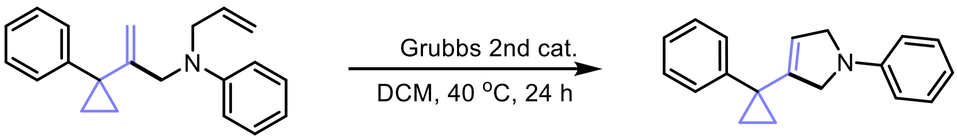

To an oven-dried flask equipped with a reflux condenser and a stir bar was charged with 3a (14.5 mg, 0.05 mmol, 1.0 equiv.), Grubbs catalyst 2nd generation (4.2 mg, 0.005 mmol, 10 mol%) and DCM (2 mL). The reaction mixture was allowed was heated to 40 °C and stirred for 24 h. The reaction was allowed to cool to room temperature, filtrated through a pad of Celite and washed with 10 mL of DCM. The filtrate was concentrated in vacuo. The residue was purified by flash column chromatograph (SiO_2, Pentanes/Et_2O – 80:1) to provide **50** (12.7 mg, 98%) of as colorless oils.

R_f = 0.25 (pentane/Et_2O = 80:1).

^{1}H NMR (400 MHz, $CDCl_3$) δ = 7.36 – 7.29 (m, 4H), 7.25 – 7.19 (m, 3H), 6.66 (tt, J = 7.3, 1.1 Hz, 1H), 6.50 – 6.44 (m, 2H), 5.47 (p, J = 1.9 Hz, 1H), 4.11 – 4.05 (m, 2H), 3.98 – 3.90 (m,

2H), 1.19 – 1.15 (m, 2H), 1.13 – 1.10 (m, 2H).

^{13}C NMR (101 MHz, $CDCl_3$) δ = 147.3, 144.3, 143.4, 129.3, 129.0, 128.4, 126.6, 120.0, 115.6, 111.1, 55.1, 54.9, 25.9, 13.8.

HRMS (+ p ESI) m/z: $[M+H]^+$ Calcd for $C_{19}H_{20}N^+$: 262.1590; Found: 262.1591.

7.2 Ring Arrangements of Vinyl Cyclopropanes (VCPs)

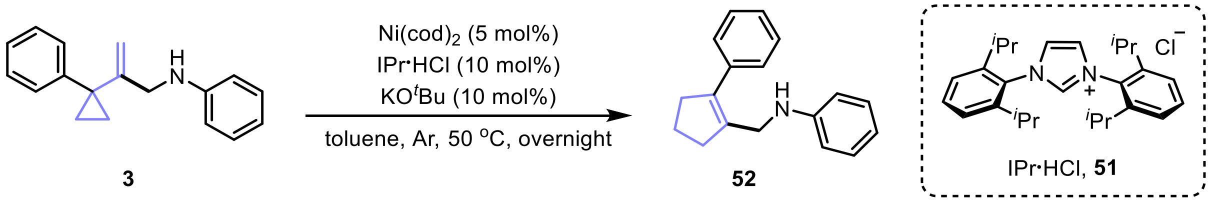

In the glovebox, an oven-dried 10 mL Schlenk tube was charged with $Ni(cod)_2$ (0.009 mmol, 5 mol %), IPr·HCl **51** (0.018 mmol, 10 mol %), KO^{t}Bu (0.018 mmol, 10 mol%) and dry anhydrous toluene (1 mL). The reaction mixture was stirred for 30 mins. Then, vinyl cyclopropane **3** (45 mg, 0.18 mmol, 1.0 equiv) in dry anhydrous toluene (1 mL) was added to solution. The Schlenk tube was sealed by screw cap and the resulting mixture was stirred at 50 °C for 16 hours. After filtration through a pad of Celite and evaporation of the solvents under reduced pressure, the residue was purified by flash column chromatography on silica gel afford the desired cyclopentane **52** (40.7 mg, 91% yield) as colorless oil.

R$_f$ = 0.30 (pentane/Et_2O = 40:1).

^{1}H NMR (500 MHz, $CDCl_3$) δ = 7.39 – 7.34 (m, 2H), 7.32 – 7.25 (m, 3H), 7.19 – 7.14 (m, 2H), 6.71 (tt, J = 7.3, 1.1 Hz, 1H), 6.58 – 6.54 (m, 2H), 3.93 (t, J = 1.2 Hz, 2H), 3.75 (brs, 1H), 2.85 – 2.78 (m, 2H), 2.64 (tdt, J = 7.7, 2.3, 1.2 Hz, 2H), 2.03 – 1.92 (m, 2H).

^{13}C NMR (126 MHz, $CDCl_3$) δ = 148.4, 139.5, 137.8, 135.9, 129.2, 128.3, 127.7, 127.0, 117.4, 112.9, 43.0, 37.8, 36.2, 22.1.

HRMS (+ p ESI) m/z: $[M+H]^+$ Calcd for $C_{18}H_{19}N^+$: 250.1590; Found: 250.1590.

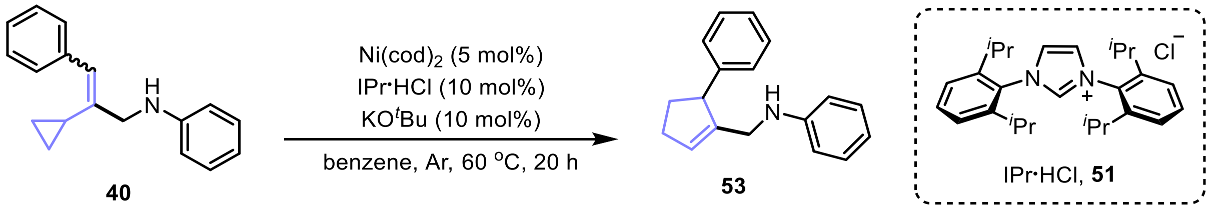

In the glovebox, an oven-dried 10 mL Schlenk tube was charged with $Ni(cod)_2$ (0.003 mmol, 5 mol %), IPr·HCl **50** (0.006 mmol, 10 mol %), KO^{t}Bu (0.006 mmol, 10 mol%) and dry anhydrous benzene (0.5 mL). The reaction mixture was stirred for 30 mins. Then, vinyl cyclopropane **40**

(15 mg, 0.06 mmol, 1.0 equiv) in dry anhydrous benzene (0.5 mL) was added to solution. The Schlenk tube was sealed by screw cap and the resulting mixture was stirred at 60 °C for 20 hours. After filtration through a pad of Celite and evaporation of the solvents under reduced pressure, the residue was purified by flash column chromatography on silica gel afford the desired cyclopentane **53** (12.0 mg, 81% yield) as colorless oil.

R$_f$ = 0.30 (pentane/Et_2O = 40:1).

^{1}H NMR (500 MHz, $CDCl_3$) δ = 7.34 – 7.28 (m, 2H), 7.24 – 7.11 (m, 5H), 6.71 (tt, *J* = 7.4, 1.0 Hz, 1H), 6.59 – 6.53 (m, 2H), 5.84 (h, *J* = 2.0 Hz, 1H), 3.82 – 3.76 (m, 1H), 3.65 – 3.53 (m, 2H), 2.57 – 2.35 (m, 3H), 1.93 – 1.84 (m, 1H).

^{13}C NMR (126 MHz, $CDCl_3$) δ = 147.6, 145.3, 144.1, 129.2, 128.7, 127.9, 127.6, 126.4, 118.0, 113.6, 52.6, 43.8, 34.4, 31.6.

HRMS (+ p ESI) m/z: $[M+H]^+$ Calcd for $C_{18}H_{19}N^+$: 250.1590; Found: 250.1595.

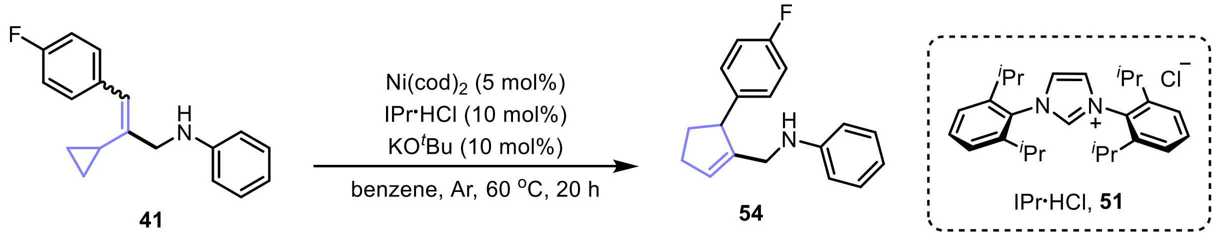

In the glovebox, an oven-dried 10 mL Schlenk tube was charged with $Ni(cod)_2$ (0.003 mmol, 5 mol %), IPr·HCl **51** (0.006 mmol, 10 mol %), KO^{t}Bu (0.006 mmol, 10 mol%) and dry anhydrous benzene (0.5 mL). The reaction mixture was stirred for 30 mins. Then, vinyl cyclopropane **41** (17 mg, 0.06 mmol, 1.0 equiv) in dry anhydrous benzene (0.5 mL) was added to solution. The Schlenk tube was sealed by screw cap and the resulting mixture was stirred at 60 °C for 20 hours. After filtration through a pad of Celite and evaporation of the solvents under reduced pressure, the residue was purified by flash column chromatography on silica gel afford the desired cyclopentane **54** (11.8 mg, 73% yield) as colorless oil.

R$_f$ = 0.30 (pentane/Et_2O = 40:1).

^{1}H NMR (500 MHz, $CDCl_3$) δ = 7.19 – 7.15 (m, 2H), 7.13 – 7.09 (m, 2H), 7.00 – 6.94 (m, 2H), 6.80 (t, *J* = 7.4 Hz, 1H), 6.72 – 6.65 (m, 2H), 5.88 (q, *J* = 1.9 Hz, 1H), 3.83 – 3.77 (m, 1H), 3.62 (dt, *J* = 15.1, 1.4 Hz, 1H), 3.57 – 3.49 (m, 1H), 2.54 – 2.31 (m, 3H), 1.85 – 1.77 (m, 1H).

^{19}F NMR (471 MHz, $CDCl_3$) δ = -116.97.

^{13}C NMR (126 MHz, $CDCl_3$) δ = 161.6 (d, *J* = 244.0 Hz), 140.7 (d, *J* = 3.4 Hz), 140.2, 129.4, 129.3, 129.02, 128.95, 116.4, 115.5 (d, *J* = 20.9 Hz), 114.9, 51.8, 34.4, 31.5, 29.8.

HRMS (+ p ESI) m/z: $[M+H]^+$ Calcd for $C_{18}H_{19}NF^+$: 268.1496; Found: 268.1494.

8. Mechanism Studies.

8.1 Radical Trapping Experiment

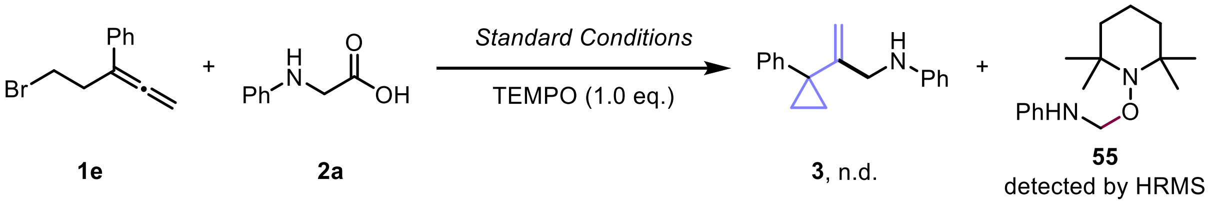

1e **2a** **3**, n.d. **55** detected by HRMS

An oven-dried 10 mL Schlenk tube was charged with phenyl glycine **2a** (0.2 mmol, 2.0 equiv), $[Ir(ppy)_2dtbbpy]PF_6$ (1.8 mg, 0.002 mmol, 2 mol %), K_2HPO_4 (34.8 mg, 0.2 mmol, 2 equiv.). The Schlenk tube was put on vacuum and backfilled with argon three times. Afterwards MeCN (1.8 mL) and DMSO (0.2 mL) was added by syringe under a flow of argon. Sequentially, allenyl **1e** (0.1 mmol, 1.0 equiv.) was added by syringe under a flow of argon. The Schlenk tube was sealed by screw cap and the resulting mixture was placed approximately 1 inch from blue led strips and irradiated and stirred for 16 hours at room temperature. After filtration through Celite and evaporation of the solvents under reduced pressure. The vinyl cyclopropane product **3** did not detected by ^1H NMR spectra analysis of the crude reaction mixture. The TEMPO adduct product **55** was detected by HRMS.

HRMS (+ p APCI) m/z: $[M+H]^+$ Calcd for $C_{16}H_{26}N_2O^+$: 263.2118; Found: 263.2116.

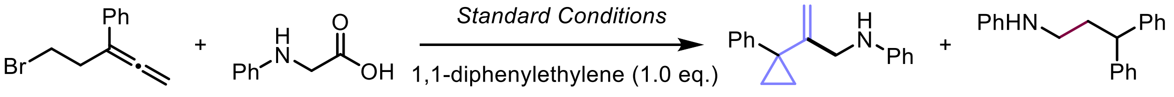

1e **2a** **3**, n.d. **56**, 46% yield

An oven-dried 10 mL Schlenk tube was charged with phenyl glycine **2a** (0.2 mmol, 2.0 equiv), $[Ir(ppy)_2dtbbpy]PF_6$ (1.8 mg, 0.002 mmol, 2 mol %), K_2HPO_4 (34.8 mg, 0.2 mmol, 2 equiv.). The Schlenk tube was put on vacuum and backfilled with argon three times. Afterwards MeCN (1.8 mL) and DMSO (0.2 mL) was added by syringe under a flow of argon. Sequentially, allenyl **1e** (0.1 mmol, 1.0 equiv.) and 1,1-diphenylethylene (0.1 mmol, 1.0 equiv.) was added by syringe under a flow of argon. The Schlenk tube was sealed by screw cap and the resulting mixture was placed approximately 1 inch from blue led strips and irradiated and stirred for 16 hours at room temperature. After filtration through Celite and evaporation of the solvents under reduced pressure. The residue was purified by flash column chromatography on silica gel afford the product **56** as a colorless oil (10.0 mg, 46% yield). The vinyl cyclopropane product **3** did not

detected by ^{1}H NMR spectra analysis of the crude reaction mixture.

R$_f$ = 0.30 (pentane/Et_2O = 40:1).

^{1}H NMR (500 MHz, $CDCl_3$) δ = 7.35 – 7.28 (m, 8H), 7.25 – 7.21 (m, 2H), 7.20 – 7.15 (m, 2H), 6.72 (tt, J = 7.3, 1.1 Hz, 1H), 6.57 – 6.53 (m, 2H), 4.10 (t, J = 7.8 Hz, 1H), 3.53 (brs, 1H), 3.14 (t, J = 7.1 Hz, 2H), 2.40 (td, J = 7.7, 6.7 Hz, 2H).

^{13}C NMR (126 MHz, $CDCl_3$) δ = 148.2, 144.5, 129.3, 128.7, 127.9, 126.4, 117.3, 112.9, 49.0, 42.5, 35.4.

HRMS (+ p APCI) m/z: $[M+H]^+$ Calcd for $C_{21}H_{22}N^+$: 288.1747; Found: 288.1747.

These results clearly indicate that the α-amino carbon radical intermediate generated through single electron transfer under the photo irradiation.

8.2 Radical Clock Experiment

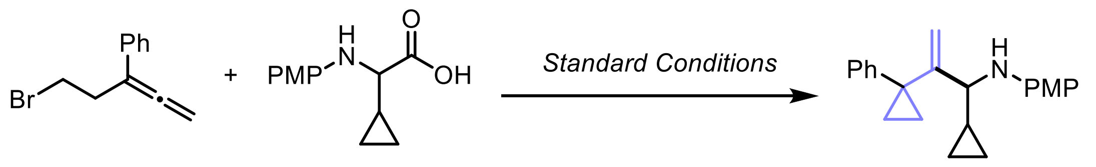

1e **2r** **S29**, 12% NMR yield

An oven-dried 10 mL Schlenk tube was charged with amino acid **2r** (0.2 mmol, 2.0 equiv), $[Ir(ppy)_2dtbbpy]PF_6$ (1.8 mg, 0.002 mmol, 2 mol %), K_2HPO_4 (34.8 mg, 0.2 mmol, 2 equiv.). The Schlenk tube was put on vacuum and backfilled with argon three times. Afterwards MeCN (1.8 mL) and DMSO (0.2 mL) was added by syringe under a flow of argon. Sequentially, allenyl **1e** (0.1 mmol, 1.0 equiv.) was added by syringe under a flow of argon. The Schlenk tube was sealed by screw cap and the resulting mixture was placed approximately 1 inch from blue led strips and irradiated and stirred for 16 hours at room temperature. After filtration through Celite and evaporation of the solvents under reduced pressure. The vinyl cyclopropane product **S29** was detected by ^{1}H NMR spectra analysis and HRMS analysis of the crude reaction mixture.

HRMS (+ p APCI) m/z: $[M+H]^+$ Calcd for $C_{22}H_{26}NO^+$: 320.2009; Found: 320.2010.

No obvious ring-opened side products could be detected. We assume that a C-centered radical (derived from 2r) is not formed, an "olefin first" mechanism would be significantly more thermodynamically favorable.

8.3 On/Off Experiment

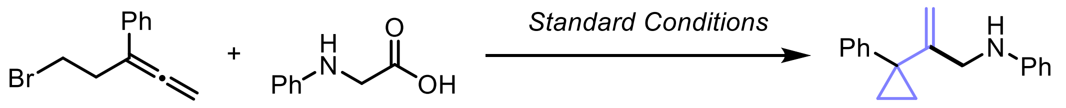

1e **2a** **3**

An oven-dried 10 mL Schlenk tube was charged with phenyl glycine **2a** (0.2 mmol, 2.0 equiv), [Ir(ppy)$_2$dtbbpy]PF$_6$ (1.8 mg, 0.002 mmol, 2 mol %), K_2HPO_4 (34.8 mg, 0.2 mmol, 2 equiv.) and 1,3,5-trimethoxybenzene (11.2 mg, 66.6 µmol). The Schlenk tube was sealed by a screw cap and the resulting mixture was placed approximately 1 inch from blue led strips and irradiated and stirred at room temperature. For each indicated time 0.3 mL reaction mixture was taken from the Schlenk tube under a flow of argon. After dried in vacuo, the yield of product **3** was monitored by ^{1}H NMR using 1,3,5-trimethoxybenzene as internal standard.

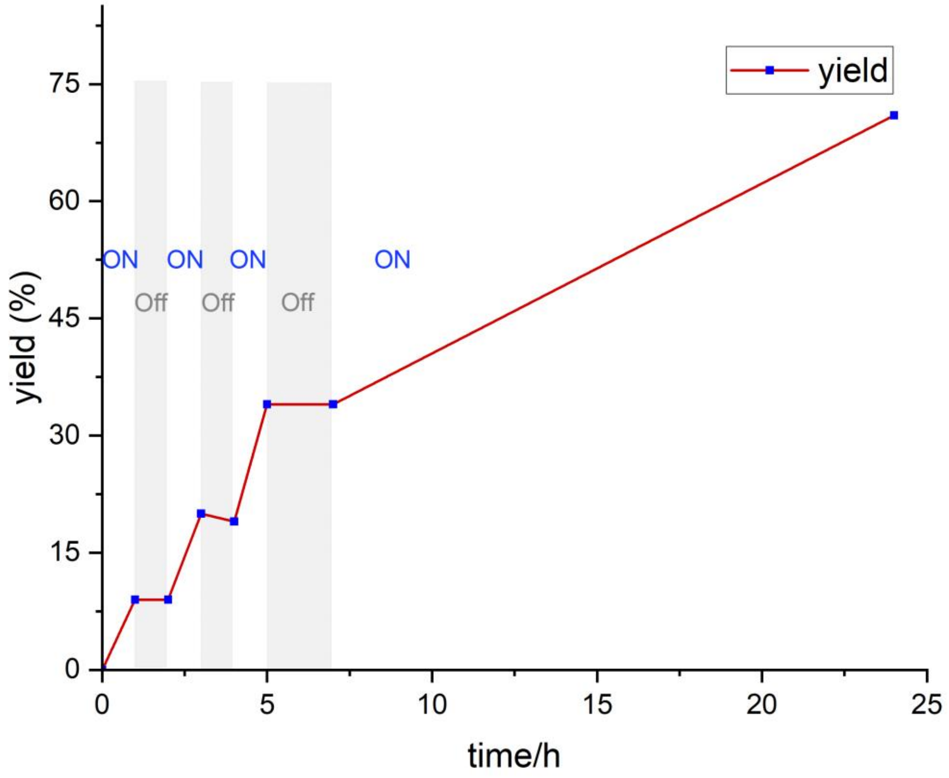

Figure S4. Monitor the formation of **3** with light on/off.

To make a comparison, under the same conditions a continuous irradiation was carried out.

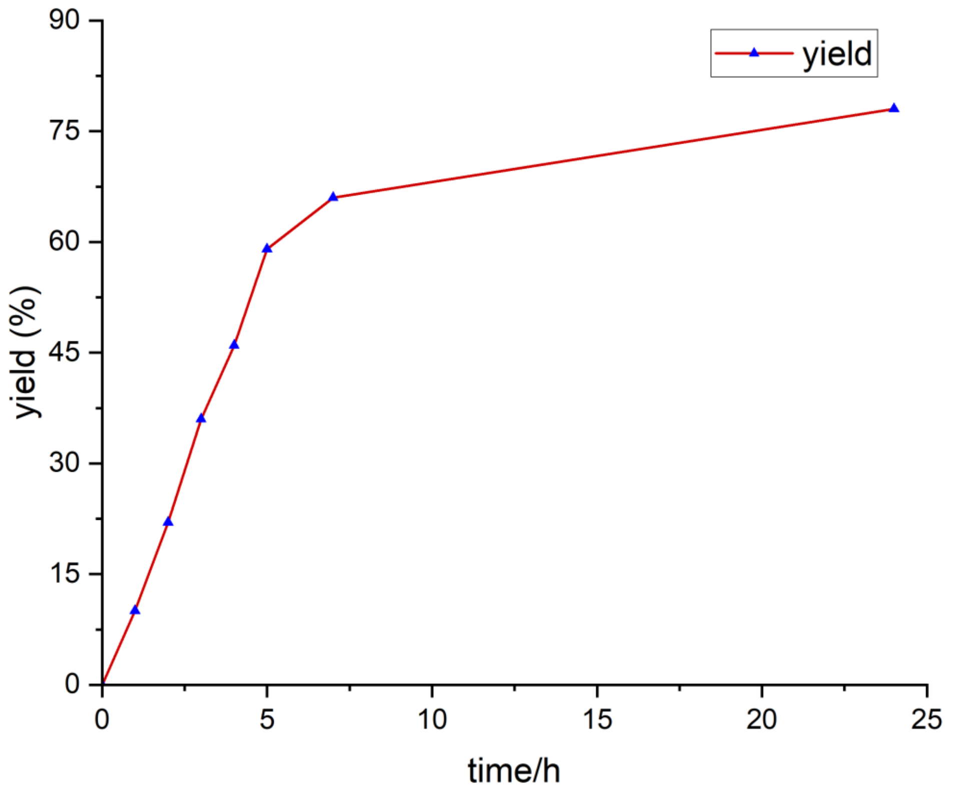

Figure S5. Monitor the formation of **3** under continuous irradiation.

8.4 Hammett Plot Study

The Hammett study was carried out by plotting log[k_X/k_H] against Hammett Substituent Constants[8] (see Table S6 and Figure S6 below).

Procedure: An oven-dried 10 mL Schlenk tube was charged with amino acid (0.2 mmol, 2.0 equiv), [Ir(ppy)$_2$dtbbpy]PF_6 (1.8 mg, 0.002 mmol, 2 mol %), K_2HPO_4 (34.8 mg, 0.2 mmol, 2 equiv.). The Schlenk tube was put on vacuum and backfilled with argon three times. Afterwards MeCN (1.8 mL) and DMSO (0.2 mL) was added by syringe under a flow of argon. Sequentially, allene**s** were added by syringe under a flow of argon. The Schlenk tube was sealed by screw cap and the resulting mixture was placed approximately 1 inch from blue led strips and irradiated and stirred for 2 hours at room temperature. After filtration through a pad of Celite and evaporation of the solvents under reduced pressure. The crude mixture was analyzed by ^{1}H NMR.

Table S6. Hammett plot of product *para*-substituent

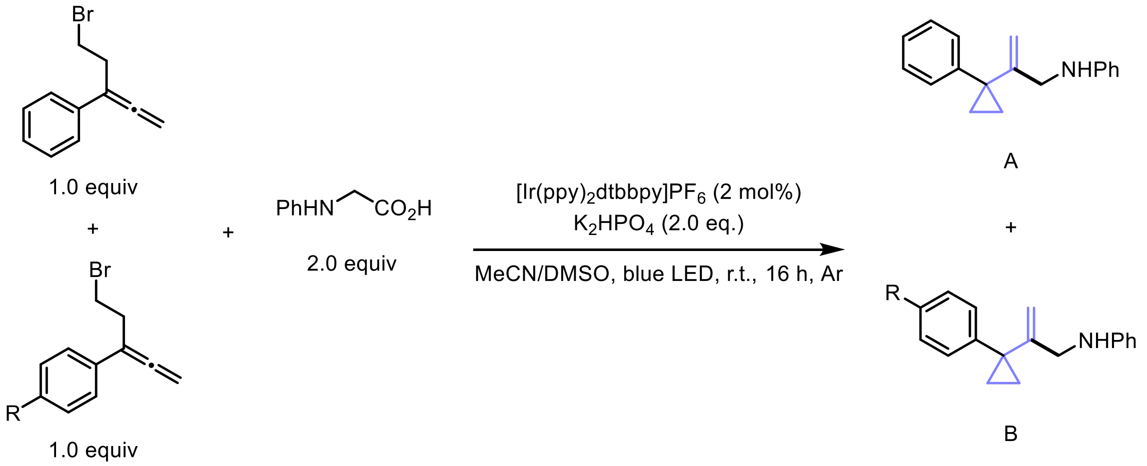

R =		Me	H	F	Cl	OCF_3	CF_3
yield	A	33	-	13	12	17	10
	B	19	-	17	22	37	37
k_X/k_H		0.58	1	1.31	1.83	2.12	3.70
$lg(k_X/k_H)$		-0.24	0	0.12	0.26	0.33	0.57
σ_{para}		-0.17	0	0.06	0.23	0.35	0.54

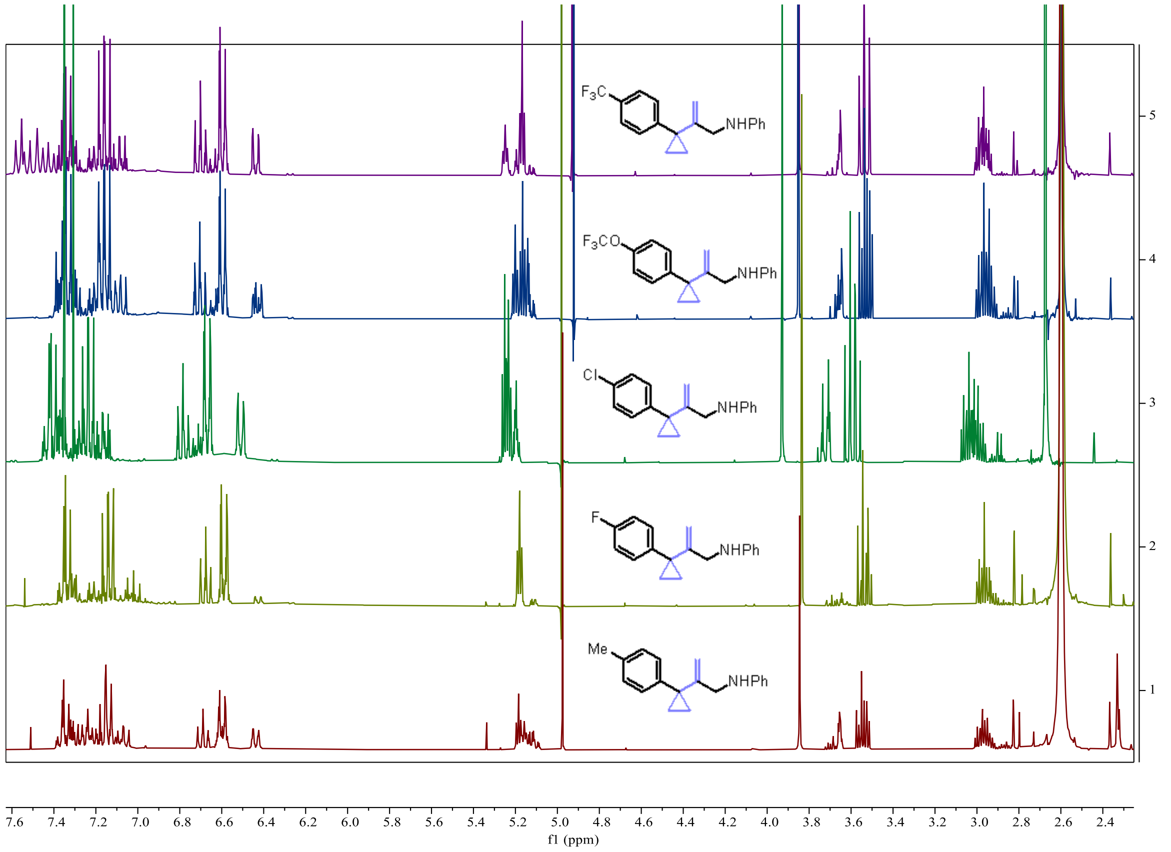

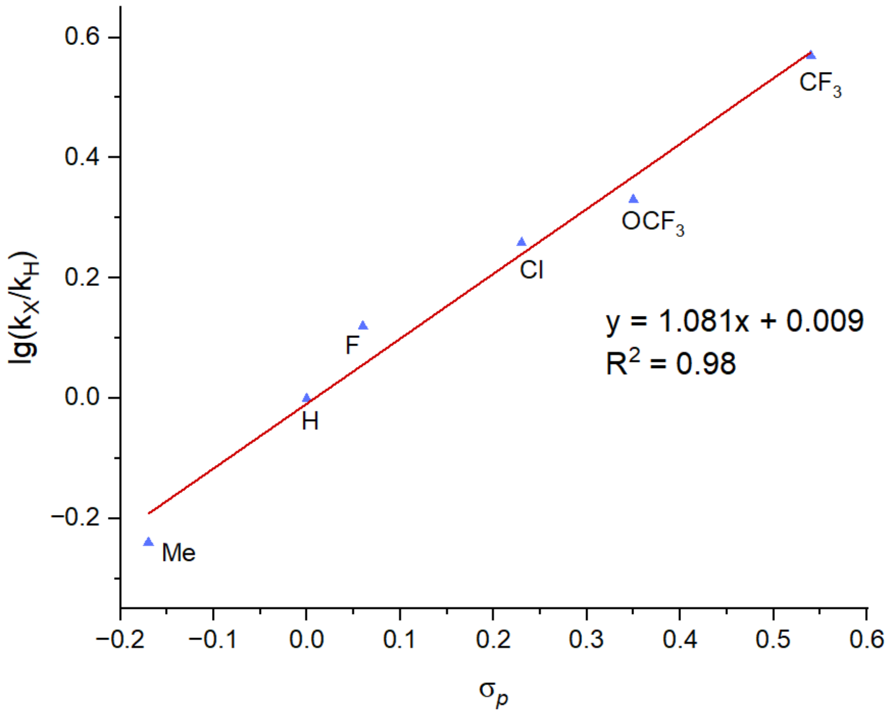

Figure S6. Hammett plot of product *para*-substituents (ρ=1.08)

8.5 Trapping Carbanion by Reaction of Allyl Acetate Substrate

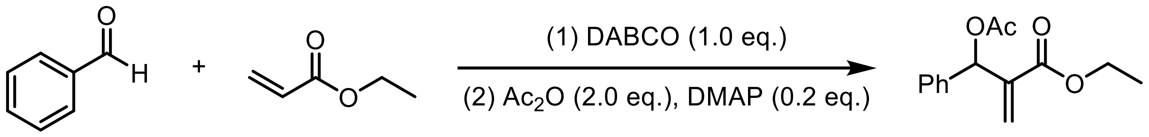

57

Benzaldehyde (1.0 mL, 10 mmol), methyl acrylate (3.4 g, 40.0 mmol, 2 equiv.) and 1,4-diazabicyclo[2.2.2]octane (DABCO, 1.1 g, 10.0 mmol, 1 equiv.) was stirred at room temperature for 24 h. The reaction was subsequently diluted with ethyl acetate (20 mL) and washed with 1 M HCl (3 x 20 mL), brine (30 mL) and dried over Na_2SO_4, then concentrated to give a yellow liquid. This yellow liquid was dissolved in dichloromethane (20 mL), followed by addition of acetic anhydride (1.85 mL, 20 mmol, 2 equiv.) and DMAP (244 mg, 2.0 mmol, 0.2 equiv.). The reaction mixture was stirred at room temperature for 2 h. The solvent was then removed under reduced pressure. The residue was purified by flash column chromatography on silica gel (Pentanes/Et_2O – 20:1) to give the desired product **57** (1.44 g, 58% yield) as a colorless oil.

R_f = 0.25 (pentane/Et_2O – 20:1).

^{1}H NMR (400 MHz, $CDCl_3$) δ = 7.40 – 7.24 (m, 5H), 6.67 (s, 1H), 6.38 (s, 1H), 5.82 (s, 1H),

4.13 (q, J = 7.2 Hz, 2H), 2.08 (s, 3H), 1.19 (t, J = 7.1 Hz, 3H).

^{13}C NMR (101 MHz, $CDCl_3$) δ = 169.4, 164.9, 139.9, 137.9, 128.4, 128.3, 127.7, 125.5, 73.2, 60.9, 21.1, 14.0.

HRMS (+ p ESI) m/z: $[M+H]^+$ Calcd for $C_{14}H_{16}O_4$: 249.1121; Found: 249.1125.

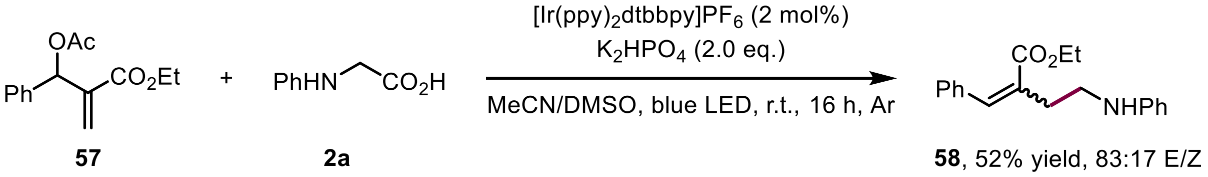

An oven-dried 10 mL Schlenk tube was charged with *N*-phenyl glycine **2a** (0.2 mmol, 2.0 equiv), $[Ir(ppy)_2dtbbpy]PF_6$ (1.8 mg, 0.002 mmol, 2 mol %), K_2HPO_4 (34.8 mg, 0.2 mmol, 2 equiv.). The Schlenk tube was put on vacuum and backfilled with argon three times. Afterwards MeCN (1.8 mL) and DMSO (0.2 mL) was added by syringe under a flow of argon. Sequentially, allyl acetate **57** (0.1 mmol, 1.0 equiv) was added by syringe under a flow of argon. The Schlenk tube was sealed by screw cap and the resulting mixture was placed approximately 1 inch from blue led strips and irradiated and stirred for 16 hours at room temperature. After filtration through a pad of Celite and evaporation of the solvents under reduced pressure. The residue was purified by flash column chromatography on silica gel (Pentanes/EtOAc – 20:1) to give the desired product **58** (15.2 mg, 52% yield) as a colorless oil.

R_f = 0.30 (pentane/EtOAc – 20:1).

^{1}H NMR (400 MHz, $CDCl_3$) δ = 7.31 – 7.11 (m, 8H), 6.69 (tt, J = 7.3, 1.1 Hz, 1H, major), 6.58 – 6.53 (m, 2H), 4.07 (q, J = 7.1 Hz, 2H, major), 3.61 (brs, 1H), 3.21 – 3.06 (m, 2H), 3.04 – 2.94 (m, 1H), 2.03 – 1.92 (m, 1H), 1.86 – 1.76 (m, 1H), 1.14 (t, J = 7.2 Hz, 3H, major).

^{13}C NMR (101 MHz, $CDCl_3$) δ = 175.4, 148.1, 139.01, 138.99, 129.3, 129.01, 128.97, 128.5, 126.6, 117.4, 112.8, 60.6, 42.1, 31.5, 14.2.

HRMS (+ p ESI) m/z: $[M+H]^+$ Calcd for $C_{19}H_{21}NO_2$: 296.1645; Found: 296.1641.

The formation of carbanion intermediates was confirmed by both the Hammett analysis and the result from the reaction between allylic acetate and amino radical indicate.

8.5 Stern-Volmer Quenching Study

The rate of quenching (kq) was determined using Stern-Volmer relationship:

$$\frac{I_0}{I} = K_q \times \tau_0 \times [\text{quencher}] + 1$$

Where I_0 is the luminescence intensity without quenching substrates, and I is the intensity in the presence of quenching substrates, and τ_0 is the lifetime of the photoexcited state of the photocatalyst. The excited state lifetime of $[Ir(ppy)_2(dtbbpy)]PF_6$ in MeCN is 557 ns.[9] In this study the compounds below are tested.

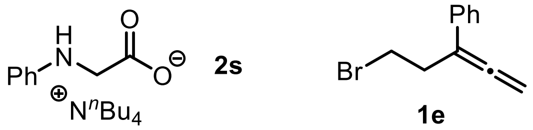

The solution of $[Ir(ppy)_2dtbbpy]PF_6$ ($1*10^{-4}$ M, 50 mL), **2s** ($1*10^{-3}$ M, 10 mL), and **1e** ($1*10^{-3}$ M, 10 mL) were prepared in glovebox using a mixture of dry MeCN. A 2.0 mL quartz cuvette equipped with a PTFE stopper was used. The excitation wavelength was λ = 375 nm according to the photocatalyst absorbance. The determined luminescence was taken from λ = 542 nm. The ratio of I_0/I was plotted as function of the quencher concentration. 2 mL solution was added into the cuvette, and the solution was degassed with argon for 1 minute before the measure. Add 100 μL of $[Ir(ppy)_2dtbbpy]PF_6$ solution and 0 μL, 100 μL, 200L, 300 μL, 600 μL and 1200 μL quencher solution respectively in the quartz cuvette and then diluted the solution to 2 mL by using dry MeCN.

Stern-Volmer quenching experiment of amino acid salt **2s** K_q is 8.751 x 10^8 $M^{-1}s^{-1}$; no quenching observed for allene **1e**.

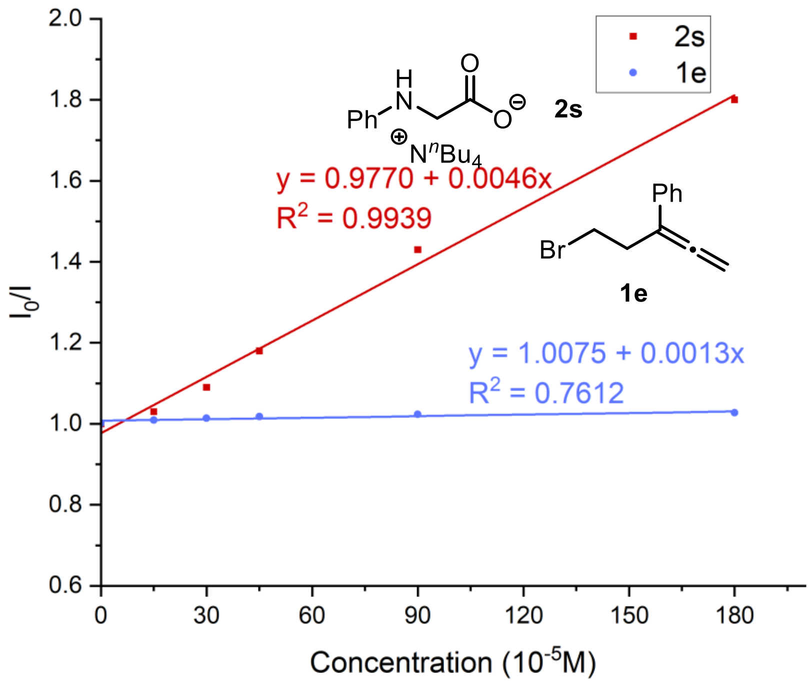

Figure S6. The overlap of all the Stern-Volmer quenching experiments.

8.6 Quantum Yield Measurements

Determination of the light intensity of the blue LED

The photon flux of blue LED was determined by standard ferrioxalate actinometry.[10]

$$photo\ flux\ (Einstein \cdot s) = 1.03 \times 10^{-6}$$

Measurement of quantum yield:

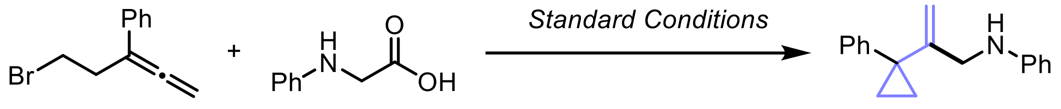

1e **2a** **3**

An oven-dried 10 mL Schlenk tube was charged with acid (0.2 mmol, 2.0 equiv), $[Ir(ppy)_2dtbbpy]PF_6$ (1.8 mg, 0.002 mmol, 2 mol %), K_2HPO_4 (34.8 mg, 0.2 mmol, 2 equiv.). The Schlenk tube was put on vacuum and backfilled with argon three times. Afterwards MeCN (1.8 mL) and DMSO (0.2 mL) was added by syringe under a flow of argon. Sequentially, allene **1** (0.1 mmol, 1.0 equiv.) was added by syringe under a flow of argon. The Schlenk tube was sealed by a screw cap and the resulting mixture was placed approximately 2.5 cm away from one 34 W blue LED and irradiated and stirred for 30 min at room temperature. The solvents were removed under reduced pressure. The moles of product (**3**) formed (**17** μmol) were measured by ^1H NMR using dibromomethane as internal standard. The quantum yield calculation is then as following:

$$\Phi = \frac{\text{moles of product}}{\text{moles of absorbed photons}} = \frac{\text{moles of product}}{\text{flux} \cdot \text{t} \cdot \text{f}}$$

Where flux is the photon flux determined by ferrioxalate actinometry (1.03×10^{-6} Einstein/s), t is the time (1800 s), and f is the fraction of light absorbed by $[Ir(ppy)_2(dtbbpy)]PF_6$ at 450 nm. A 1×10^{-3} M solution of $[Ir(ppy)_2(dtbbpy)]PF_6$ in MeCN was prepared, and the absorbance of the solution at 450 nm was 0.9882. The fraction of light absorbed at 450 nm was calculated: $f = 1.0000 - 10^{-A} = 1.0000 - 10^{-0.9882} = 0.8972$.

$$\Phi = \frac{\text{moles of product}}{\text{moles of absorbed photons}} = \frac{\text{moles of product}}{\text{flux} \cdot \text{t} \cdot \text{f}}$$

$$= \frac{1.7 \times 10^{-5}}{1.03 \times 10^{-6} \times 1800 \times 0.8972} = 0.0102$$

9. Reaction Limitations.

Table S7. Reaction limitations

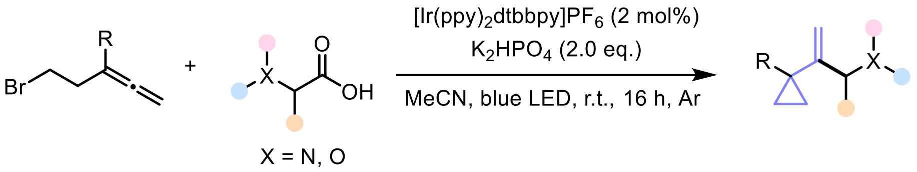

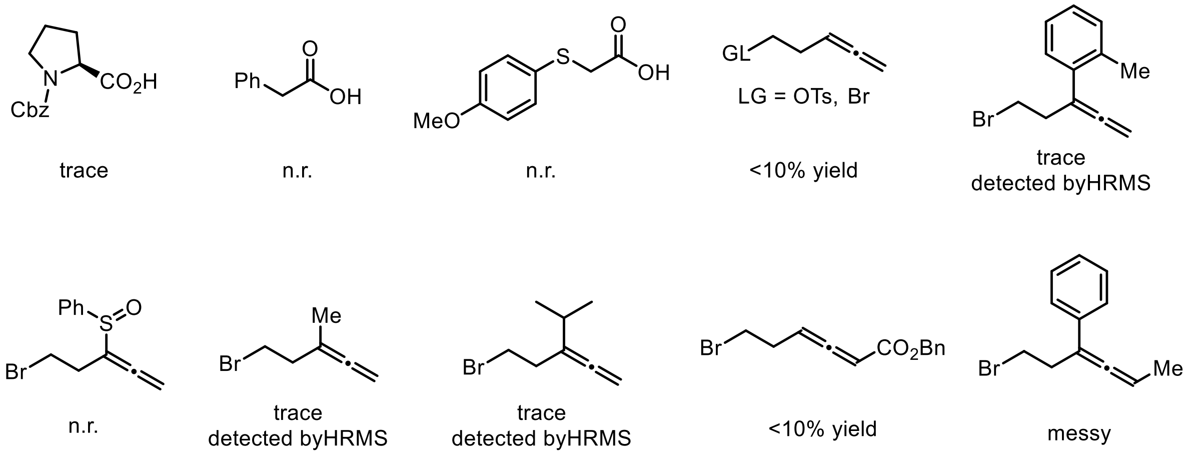

Note:

1. Phenylacetic acid was subjected to the standard conditions or in the present of other photocatalysts, while no reaction was detected, which indicated the heteroatom stabilized the carbon radical species.

2. 2-((4-methoxyphenyl)thio)acetic acid was tried under the photocatalytic conditions, no desired product was formed.

3. *ortho*-Substituted phenyl allene gave trace product (**31**), due to the steric hindrance of the substrate.

4. Alkyl substituted allenes could not proceed on our rRPCC reaction, in the same time, indicating the stabilized carbanion intermediates is the key intermediate.

5. Trisubstituted allene was conducted on optimal conditions, the reaction was messy and no desired product was detected.

10. Reference.

1. Yi, J.; Lu, X.; Sun, Y-Y.; Xiao, B.; Liu, L. *Angew. Chem. Int. Ed.*, **2013**, *52*, 12409–12413.
2. Efskind, J., Römming, C., Undheim, K., *J. Chem. Soc., Perkin Trans. 1,* **2001**, 2697-2703
3. Köpfer, A., Breit, B. *Angew. Chem. Int. Ed.,* **2015**, *54*, 6913-6917.
4. Makarov, I. S., Brocklehurst, C. E., Karaghiosoff, K., Koch, G., Knochel, P., *Agnew. Chem. Int. Ed.,* **2017**, *56*, 12774-12777.
5. Dong, Y., Breit, B. *Org. Lett.*, **2021**, *23*, 6765-6769.
6. Martzel, T., Lohier, J. F., Gaumont, A. C., Brière, J. F., Perrio, S., *Adv. Synth. Catal.,* **2017**, *359*, 96-106.
7. Mikan, C. P., Matthews, A., Harris, D., McIvor, C. E., Waddell, P. G., Sims, M. T., Knowles, J. P., *Chem. Sci.,* **2023**, *14*, 6992-6996.
8. Hansch, C., Leo, A., Taft, R. W. *Chem. Rev.*, **1991**, *91*, 165–195.
9. Slinker, J. D.; Gorodetsky, A. A.; Lowry, M. S.; Wang, J.; Parker, S.; Rohl, R.; Bernhard, S.; Malliaras, G. G., *J. Am. Chem. Soc.,* **2014**, *126*, 2763-2767.
10. Zheng, J.; Breit, B., *Angew. Chem. Int. Ed.,* **2019**, *58*, 3392-3397.

11. Copies of NMR Spectra

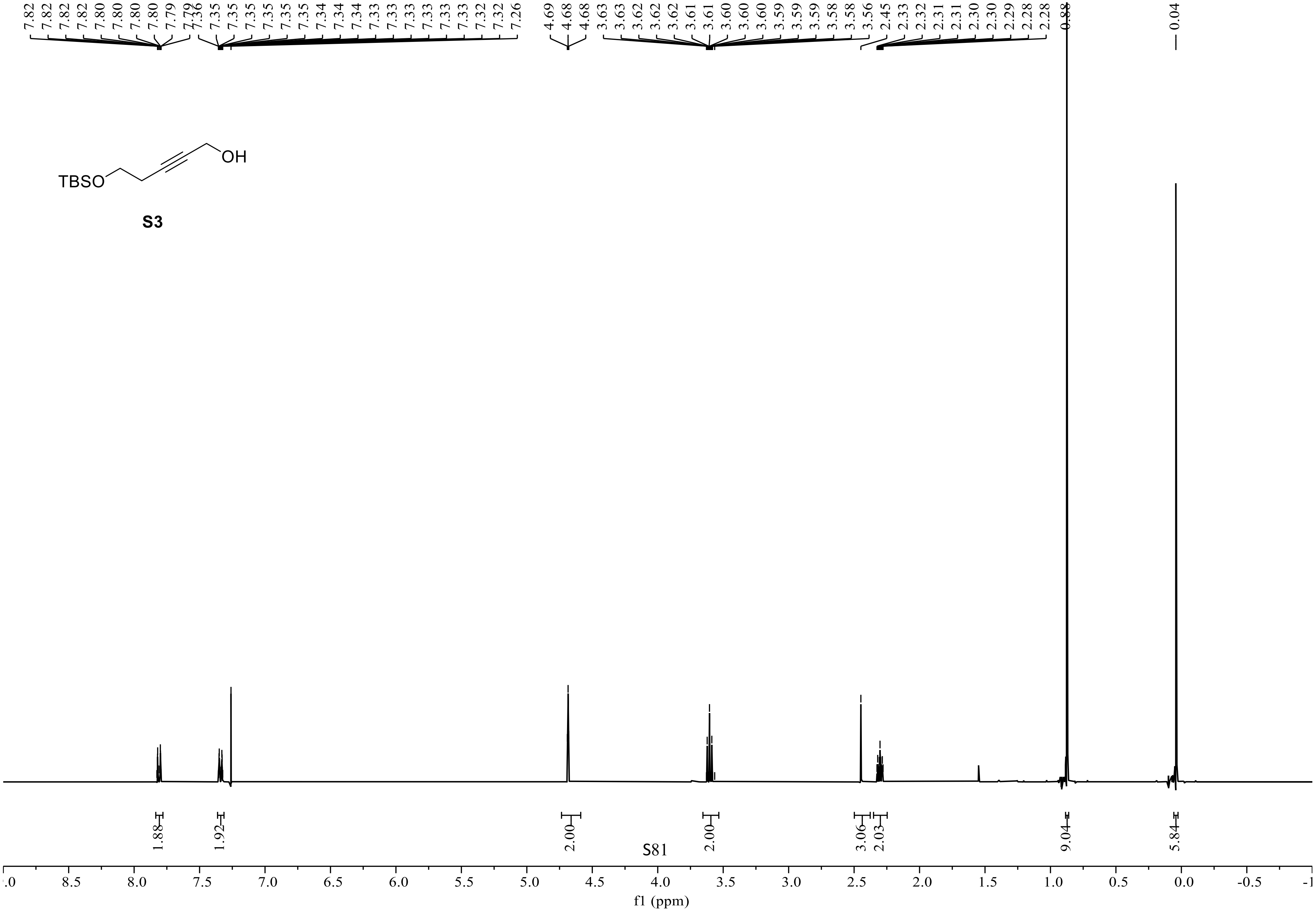
7.82 7.82 7.82 7.82 7.80 7.80 7.80 7.80 7.79 7.36 7.35 7.35 7.35 7.35 7.35 7.35 7.34 7.34 7.34 7.33 7.33 7.33 7.33 7.33 7.33 7.32 7.32 7.26
4.69 4.68 4.68
3.63 3.63 3.62 3.62 3.62 3.61 3.61 3.60 3.60 3.60 3.59 3.59 3.59 3.58 3.58 3.56
2.45 2.33 2.32 2.31 2.31 2.30 2.30 2.29 2.28 2.28
0.88
0.04
TBSO
OH
S3
1.88
1.92
2.00
2.00
3.06
2.03
9.04
5.84
f1 (ppm)

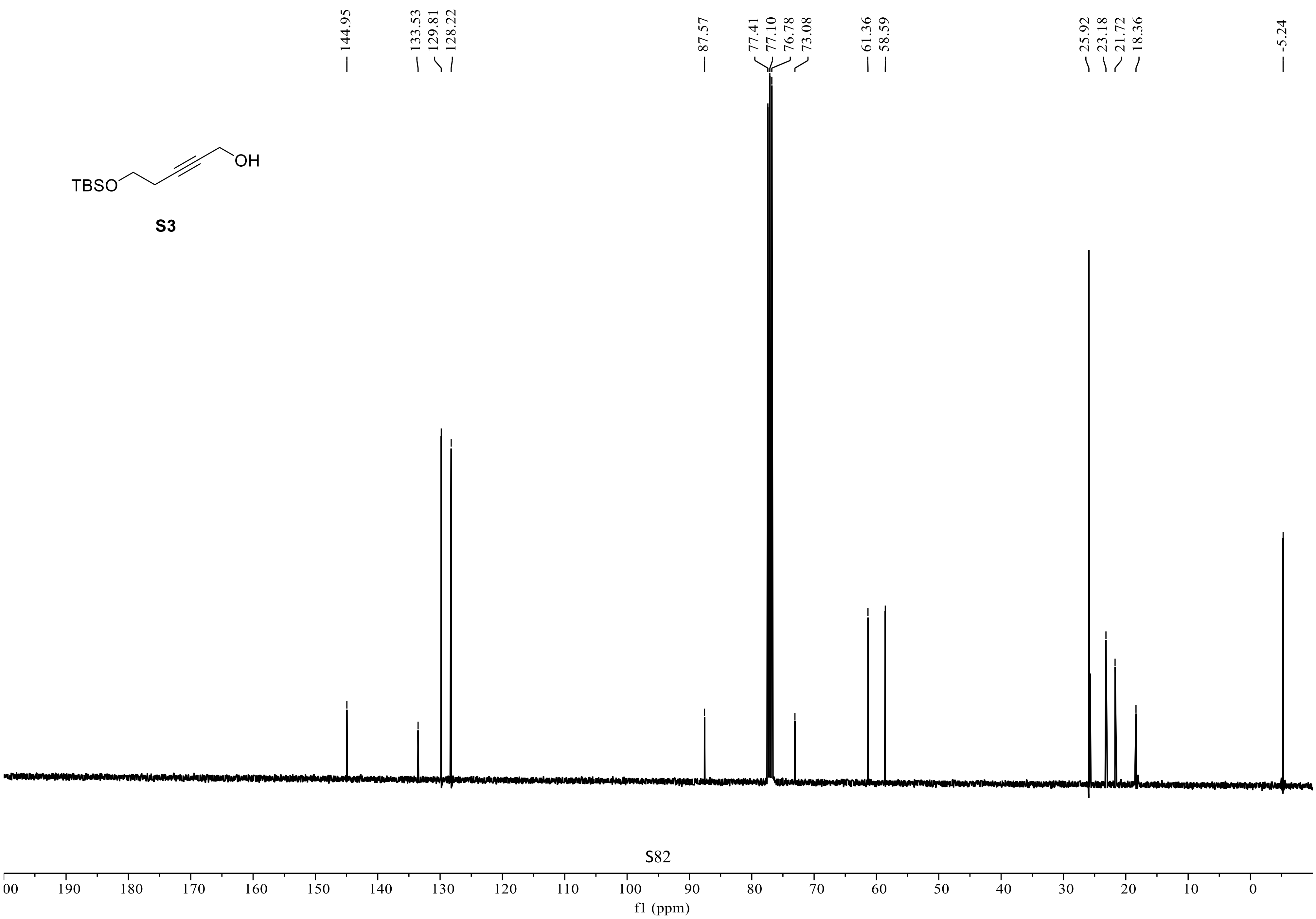
144.95
133.53
129.81
128.22
87.57
77.41
77.10
76.78
73.08
61.36
58.59
25.92
23.18
21.72
18.36
-5.24
TBSO
OH
S3
00 190 180 170 160 150 140 130 120 110 100 90 80 70 60 50 40 30 20 10 0
f1 (ppm)

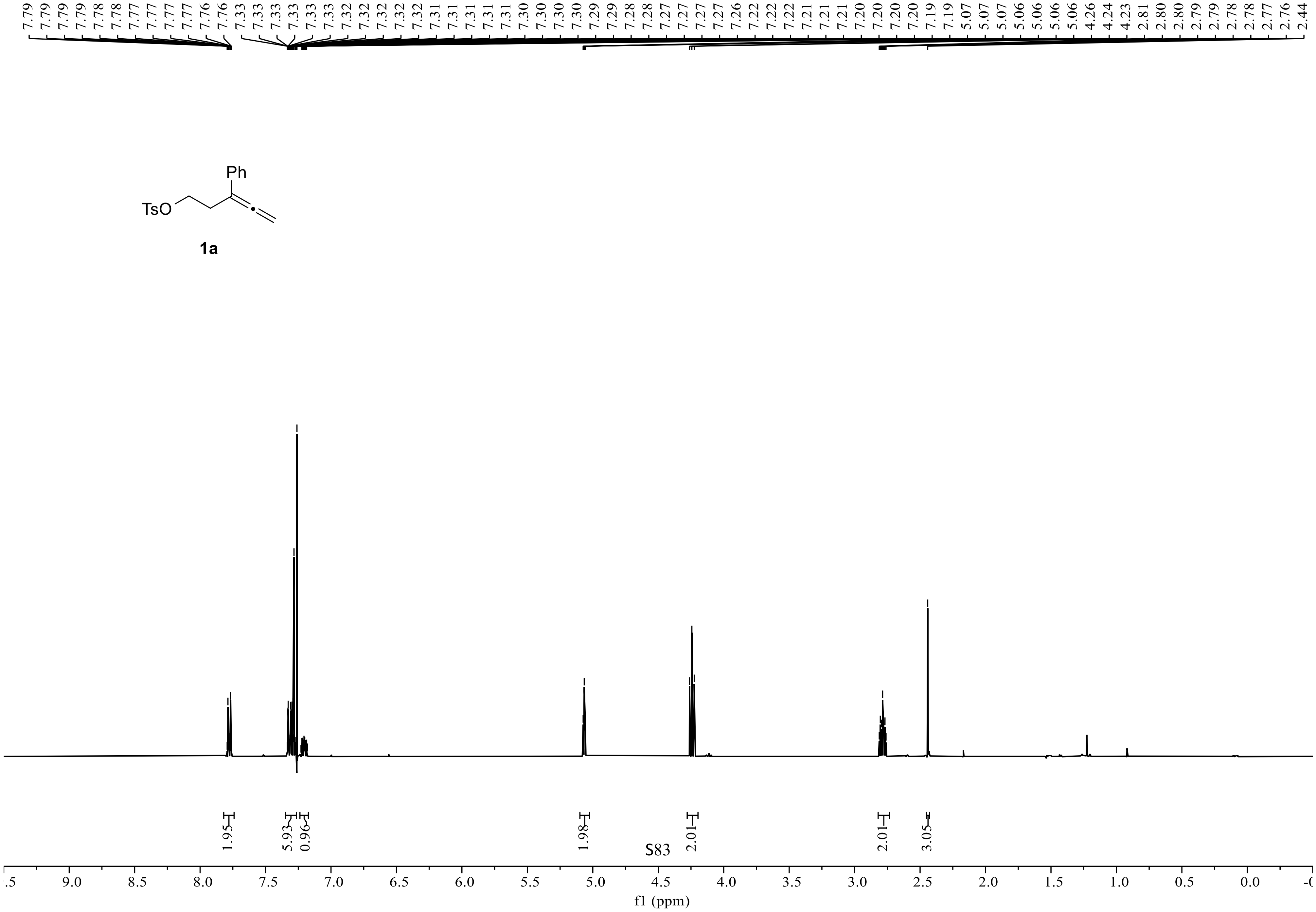
Ph
TsO
1a
f1 (ppm)

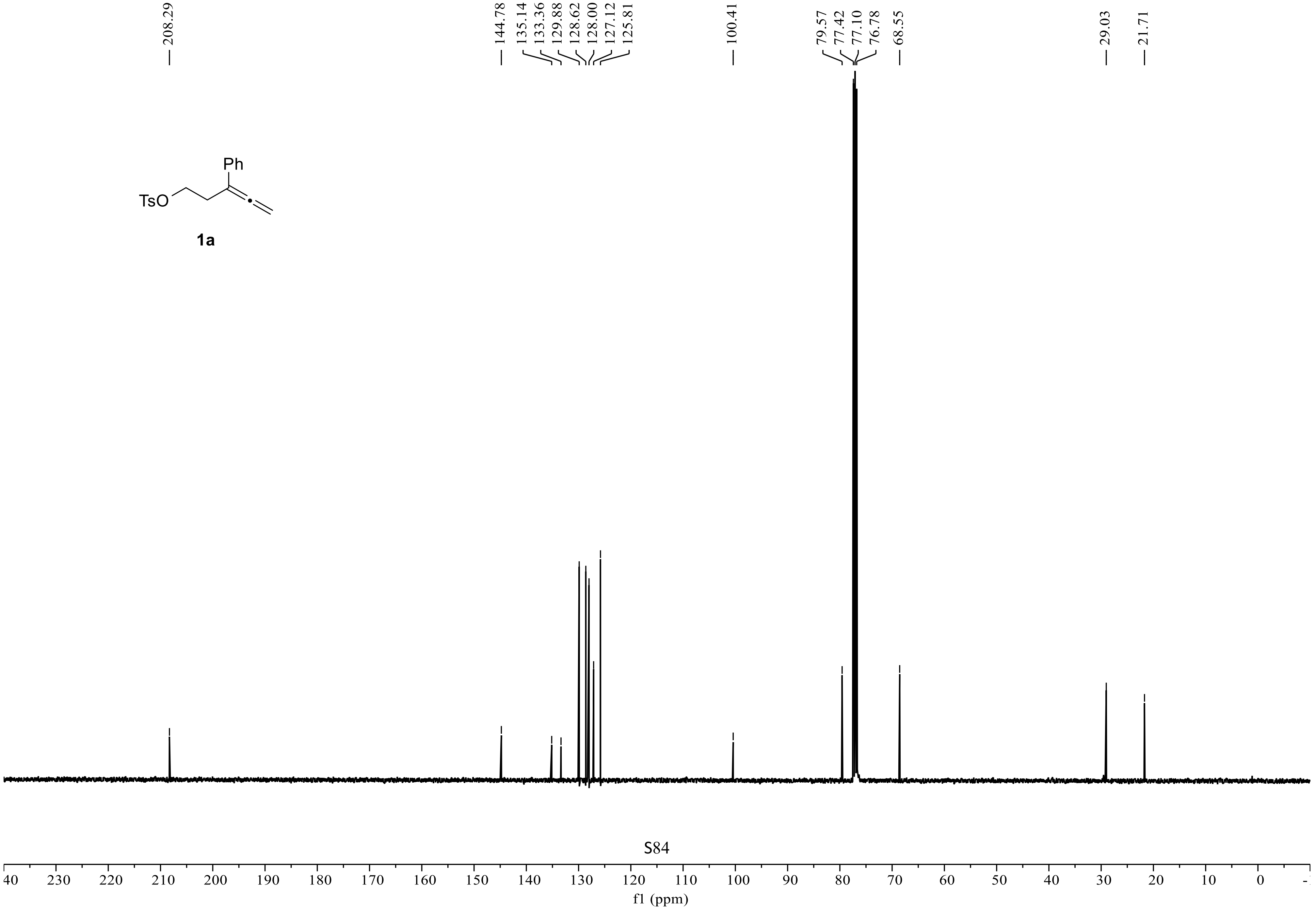
208.29
144.78
135.14
133.36
129.88
128.62
128.00
127.12
125.81
100.41
79.57
77.42
77.10
76.78
68.55
29.03
21.71
Ph
TsO
1a
40 230 220 210 200 190 180 170 160 150 140 130 120 110 100 90 80 70 60 50 40 30 20 10 0
f1 (ppm)

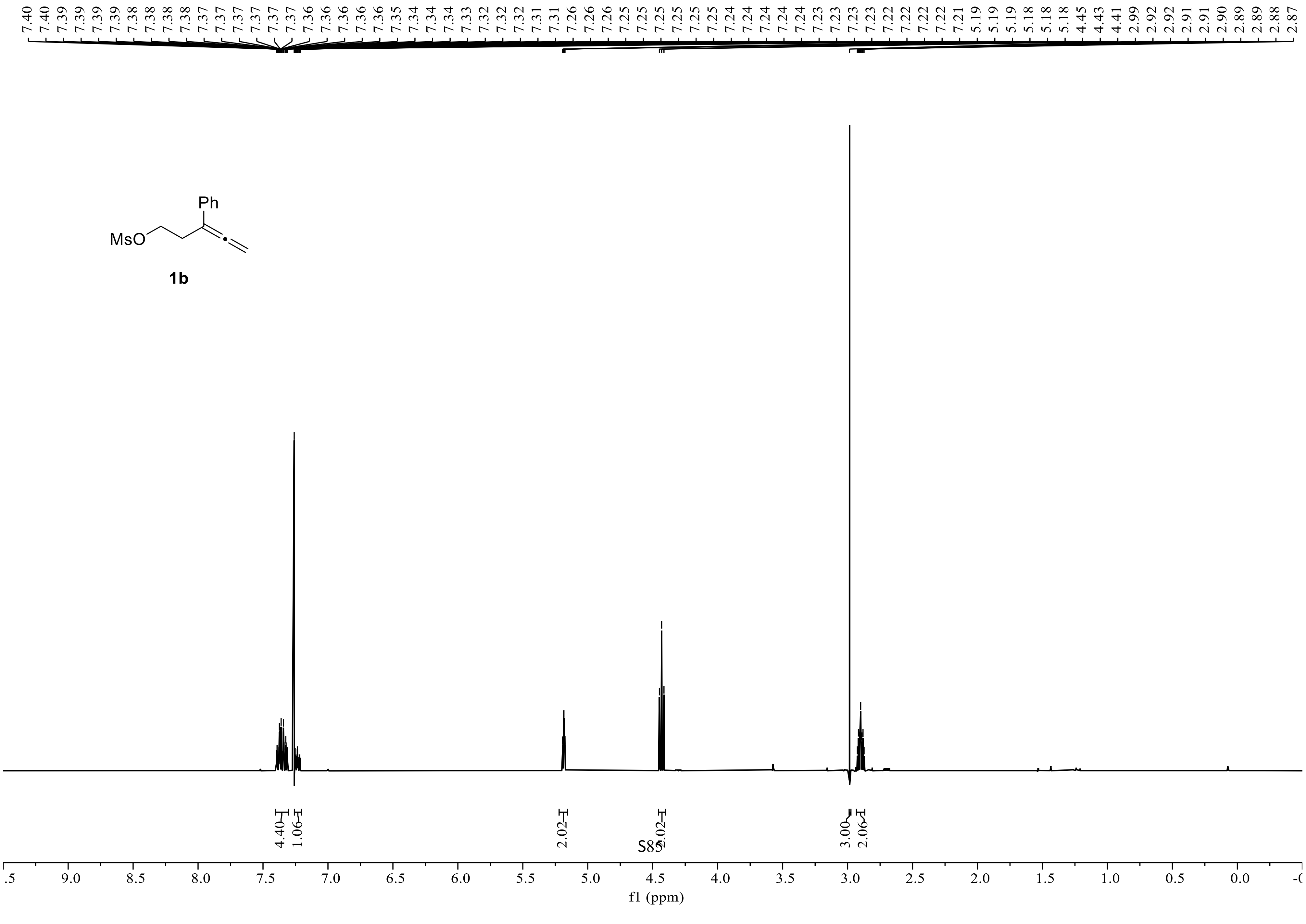
7.40 7.40 7.39 7.39 7.39 7.39 7.38 7.38 7.38 7.38 7.37 7.37 7.37 7.37 7.37 7.37 7.36 7.36 7.36 7.36 7.35 7.34 7.34 7.34 7.33 7.32 7.32 7.32 7.31 7.31 7.26 7.26 7.26 7.25 7.25 7.25 7.25 7.25 7.25 7.24 7.24 7.24 7.24 7.24 7.23 7.23 7.23 7.23 7.22 7.22 7.22 7.22 7.22 7.21 5.19 5.19 5.19 5.18 5.18 5.18 4.45 4.43 4.41 2.99 2.92 2.92 2.91 2.91 2.90 2.89 2.89 2.88 2.87
Ph
MsO
1b
4.40
1.06
2.02
2.02
3.00
2.06
9.0 8.5 8.0 7.5 7.0 6.5 6.0 5.5 5.0 4.5 4.0 3.5 3.0 2.5 2.0 1.5 1.0 0.5 0.0
f1 (ppm)

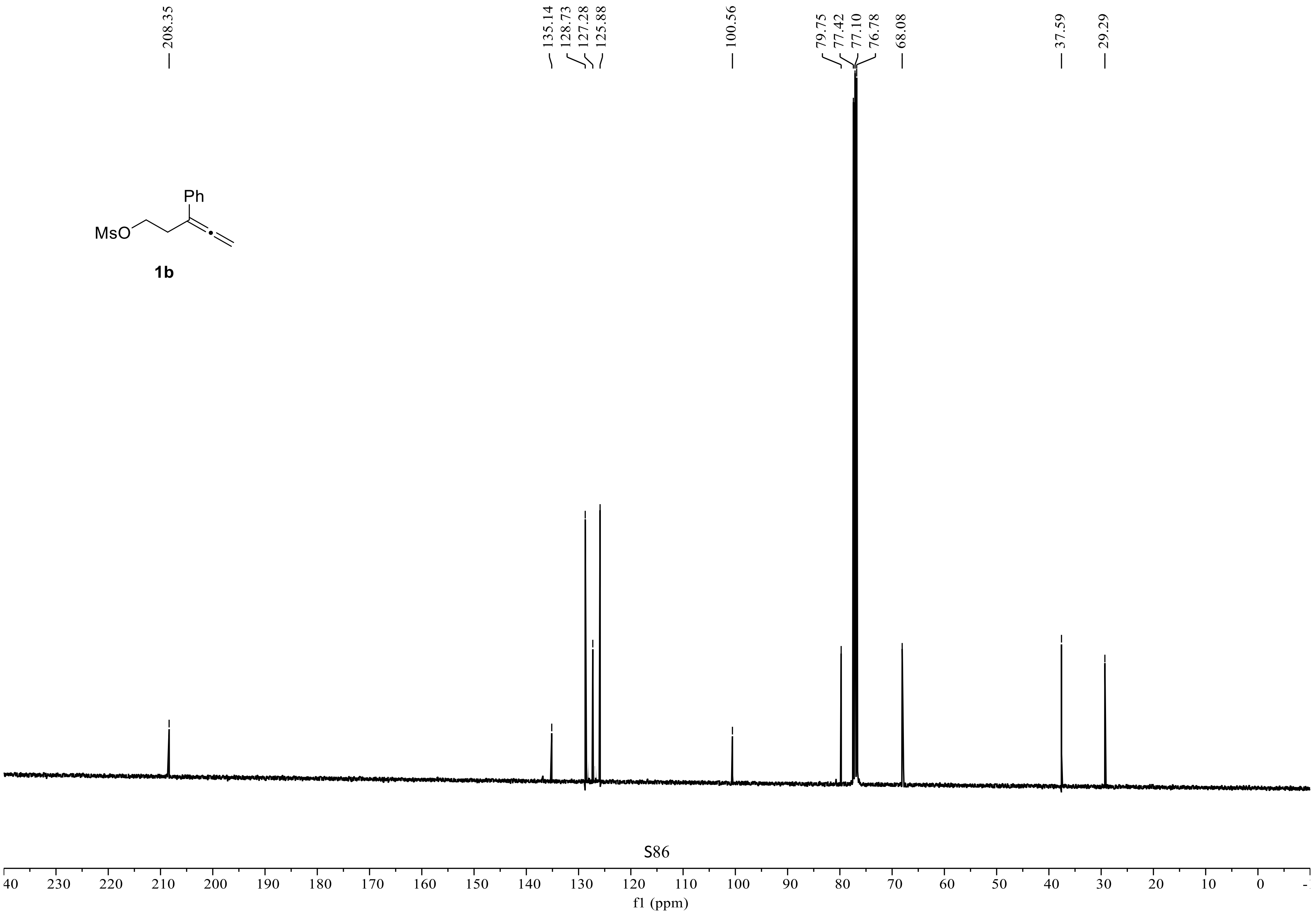
208.35
135.14
128.73
127.28
125.88
100.56
79.75
77.42
77.10
76.78
68.08
37.59
29.29
Ph
MsO
1b
40 230 220 210 200 190 180 170 160 150 140 130 120 110 100 90 80 70 60 50 40 30 20 10 0
f1 (ppm)

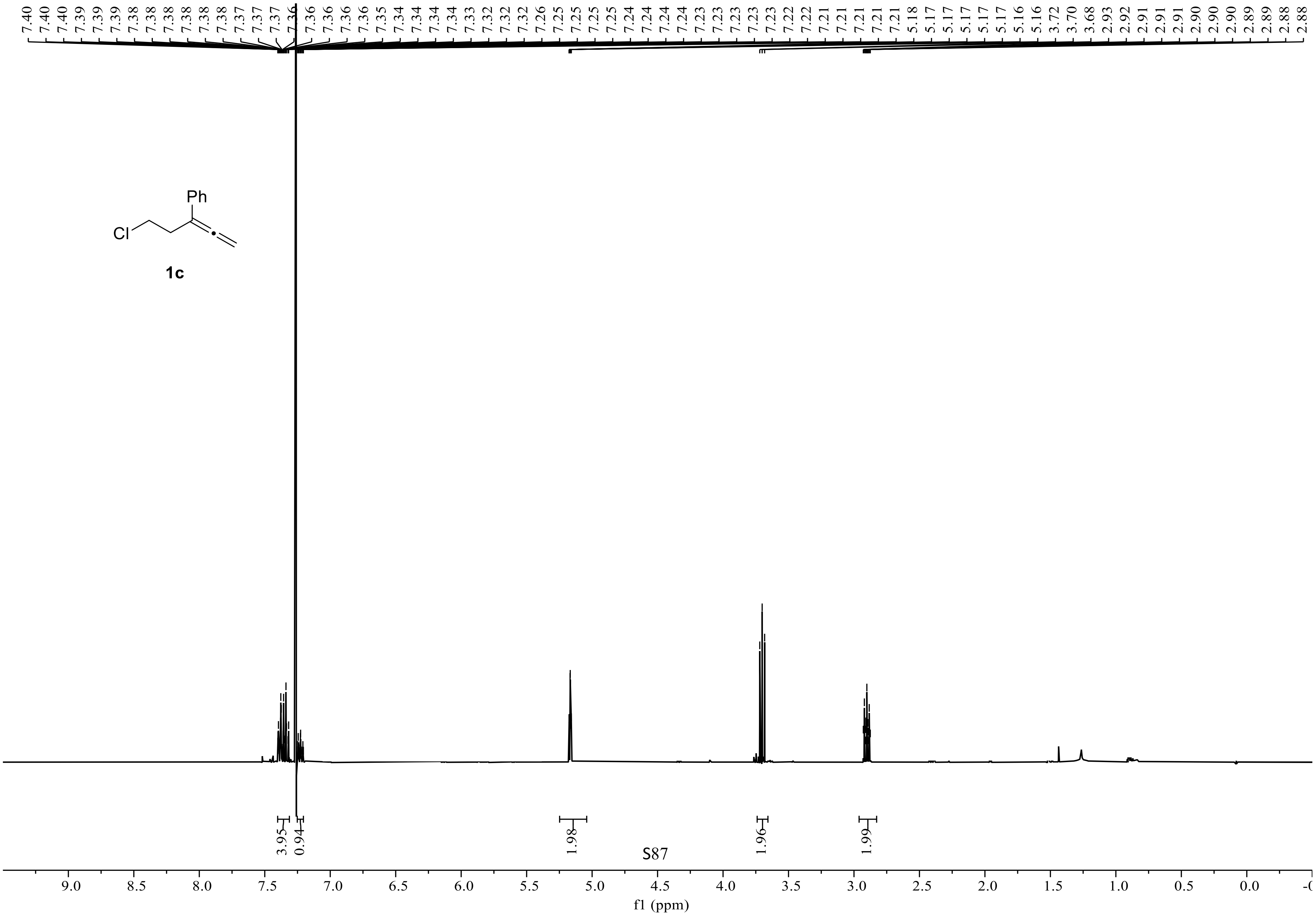
7.40 7.40 7.40 7.39 7.39 7.39 7.38 7.38 7.38 7.38 7.38 7.38 7.37 7.37 7.37 7.36 7.36 7.36 7.36 7.36 7.35 7.34 7.34 7.34 7.34 7.33 7.32 7.32 7.32 7.26 7.25 7.25 7.25 7.25 7.24 7.24 7.24 7.24 7.23 7.23 7.23 7.23 7.23 7.22 7.22 7.21 7.21 7.21 7.21 7.21 5.18 5.17 5.17 5.17 5.17 5.17 5.16 5.16 3.72 3.70 3.68 2.93 2.92 2.91 2.91 2.91 2.90 2.90 2.89 2.89 2.88 2.88
Ph
Cl
1c
3.95
0.94
1.98
1.96
1.99
9.0 8.5 8.0 7.5 7.0 6.5 6.0 5.5 5.0 4.5 4.0 3.5 3.0 2.5 2.0 1.5 1.0 0.5 0.0
f1 (ppm)

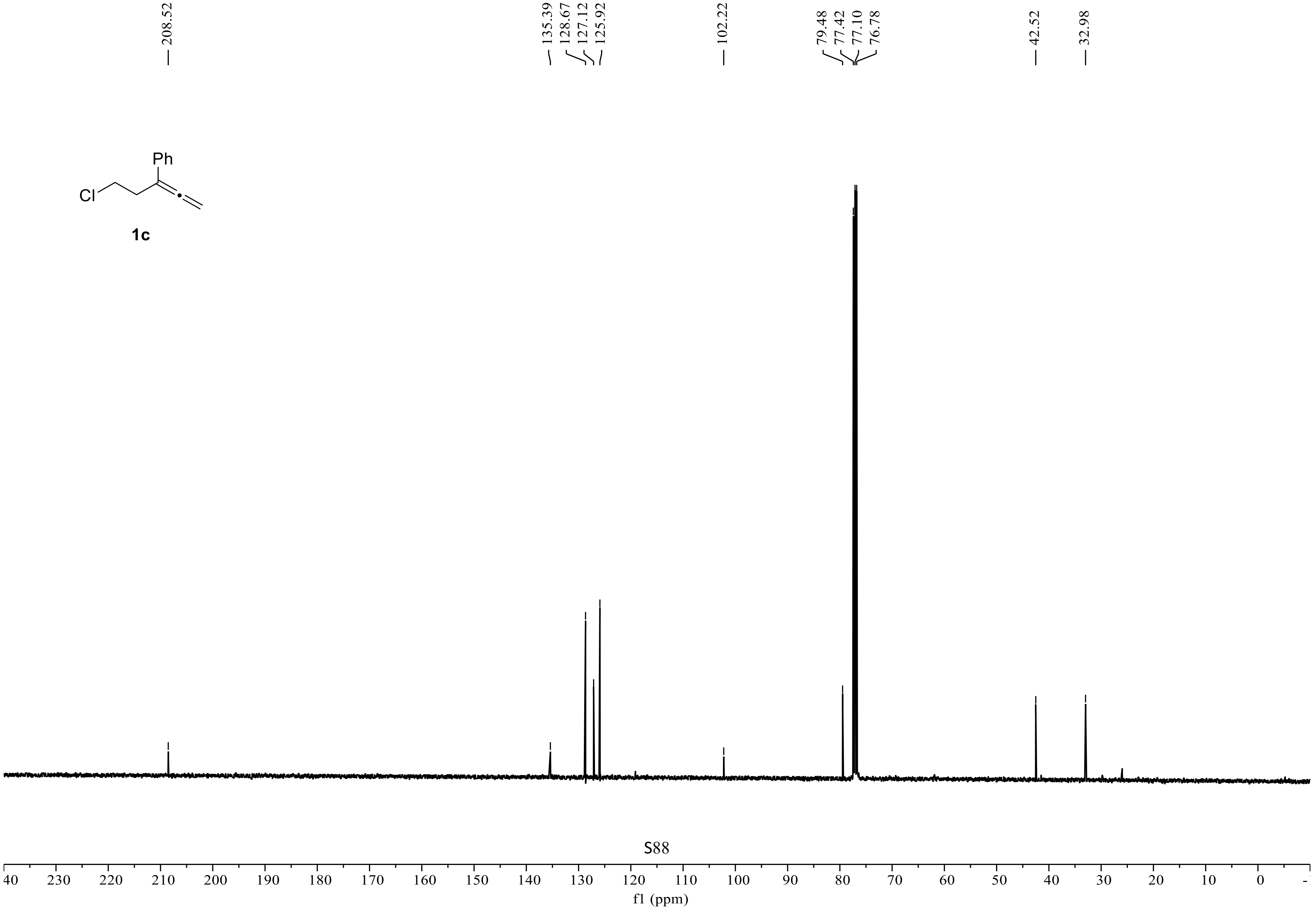
208.52
135.39
128.67
127.12
125.92
102.22
79.48
77.42
77.10
76.78
42.52
32.98
Ph
Cl
1c
f1 (ppm)

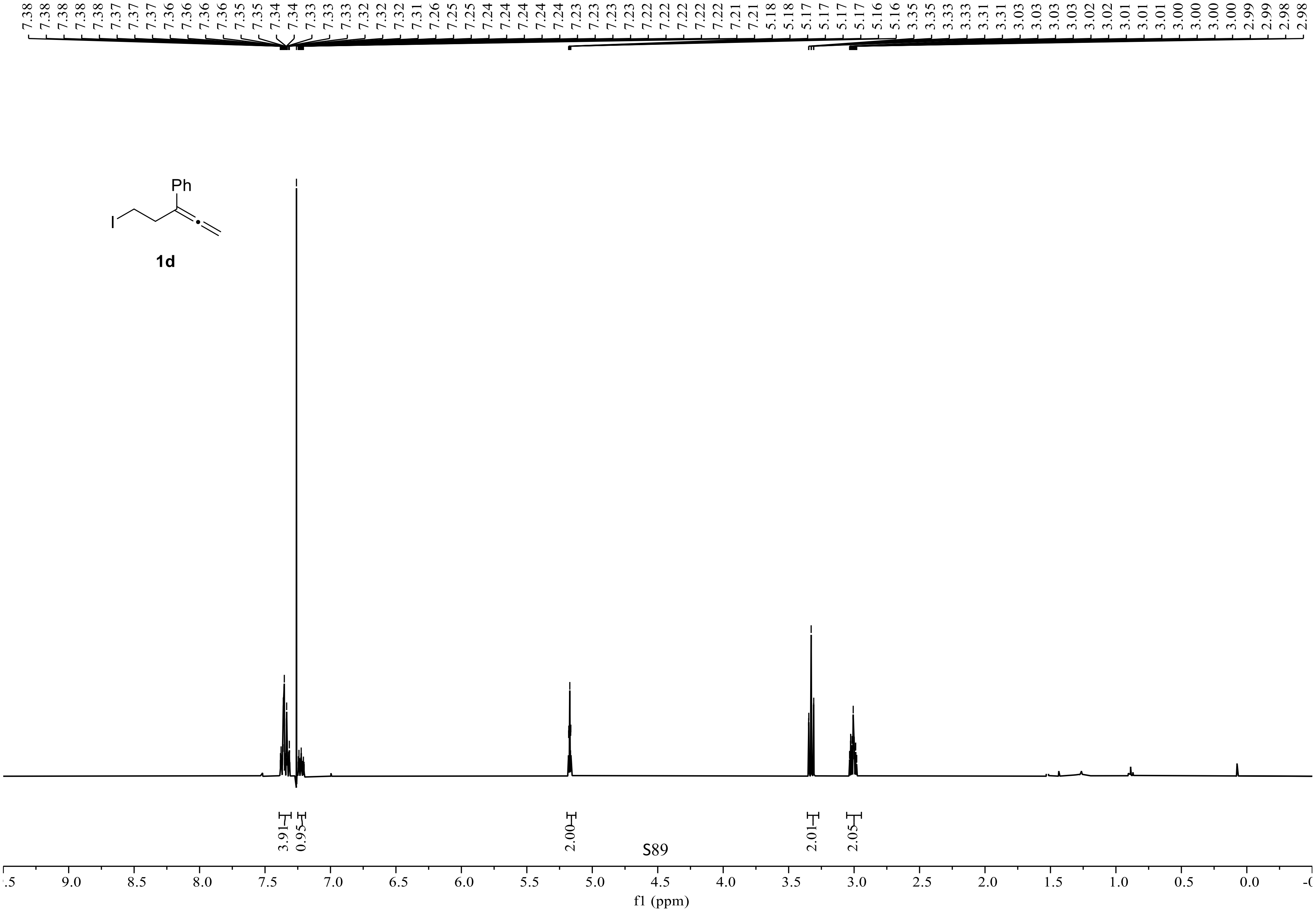
7.38 7.38 7.38 7.38 7.38 7.37 7.37 7.37 7.36 7.36 7.36 7.36 7.35 7.35 7.34 7.34 7.33 7.33 7.33 7.32 7.32 7.32 7.31 7.26 7.25 7.25 7.24 7.24 7.24 7.24 7.24 7.23 7.23 7.23 7.23 7.22 7.22 7.22 7.22 7.22 7.21 7.21 5.18 5.18 5.17 5.17 5.17 5.17 5.16 5.16 3.35 3.35 3.33 3.33 3.31 3.31 3.03 3.03 3.03 3.02 3.02 3.01 3.01 3.00 3.00 3.00 2.99 2.99 2.98 2.98
Ph
I
1d
3.91
0.95
2.00
2.01
2.05
9.0 8.5 8.0 7.5 7.0 6.5 6.0 5.5 5.0 4.5 4.0 3.5 3.0 2.5 2.0 1.5 1.0 0.5 0.0
f1 (ppm)

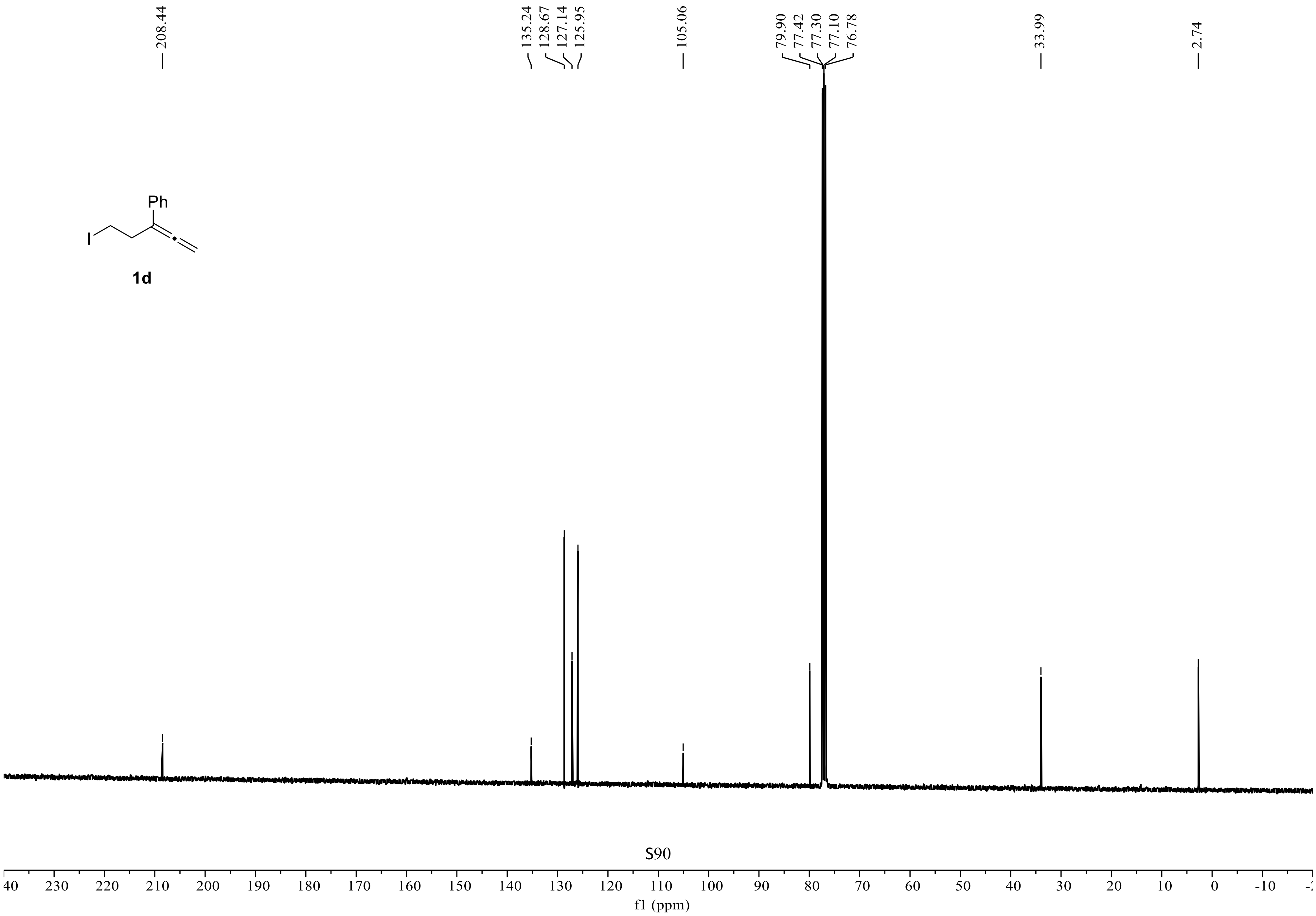

208.44
135.24
128.67
127.14
125.95
105.06
79.90
77.42
77.30
77.10
76.78
33.99
2.74
Ph
I
1d
f1 (ppm)

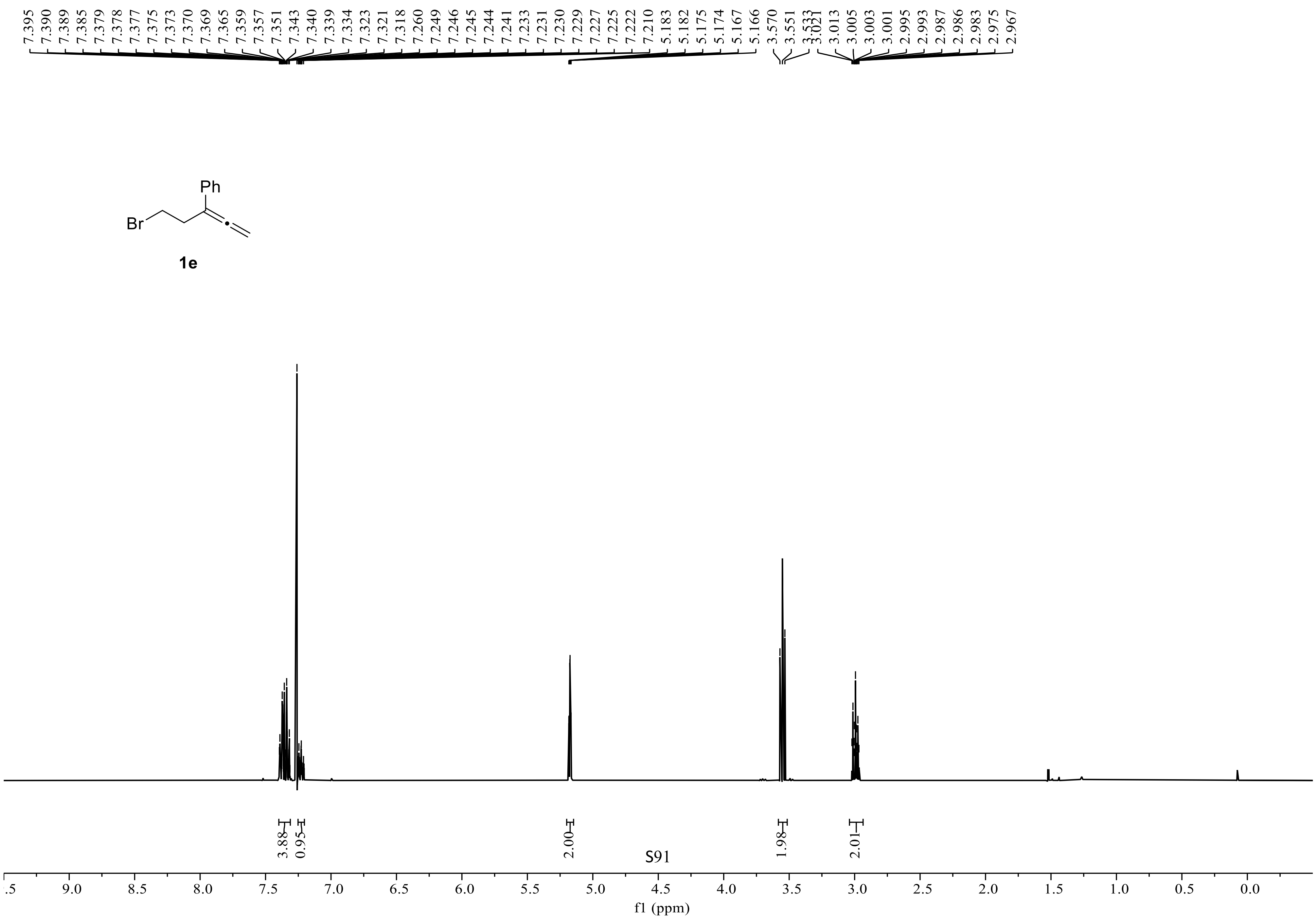
7.395
7.390
7.389
7.385
7.379
7.378
7.377
7.375
7.373
7.370
7.369
7.365
7.359
7.357
7.351
7.343
7.340
7.339
7.334
7.323
7.321
7.318
7.260
7.249
7.246
7.245
7.244
7.241
7.233
7.231
7.230
7.229
7.227
7.225
7.222
7.210
5.183
5.182
5.175
5.174
5.167
5.166
3.570
3.551
3.533
3.021
3.013
3.005
3.003
3.001
2.995
2.993
2.987
2.986
2.983
2.975
2.967
Ph
Br
1e
3.88
0.95
2.00
1.98
2.01
9.0
8.5
8.0
7.5
7.0
6.5
6.0
5.5
5.0
4.5
4.0
3.5
3.0
2.5
2.0
1.5
1.0
0.5
0.0
f1 (ppm)

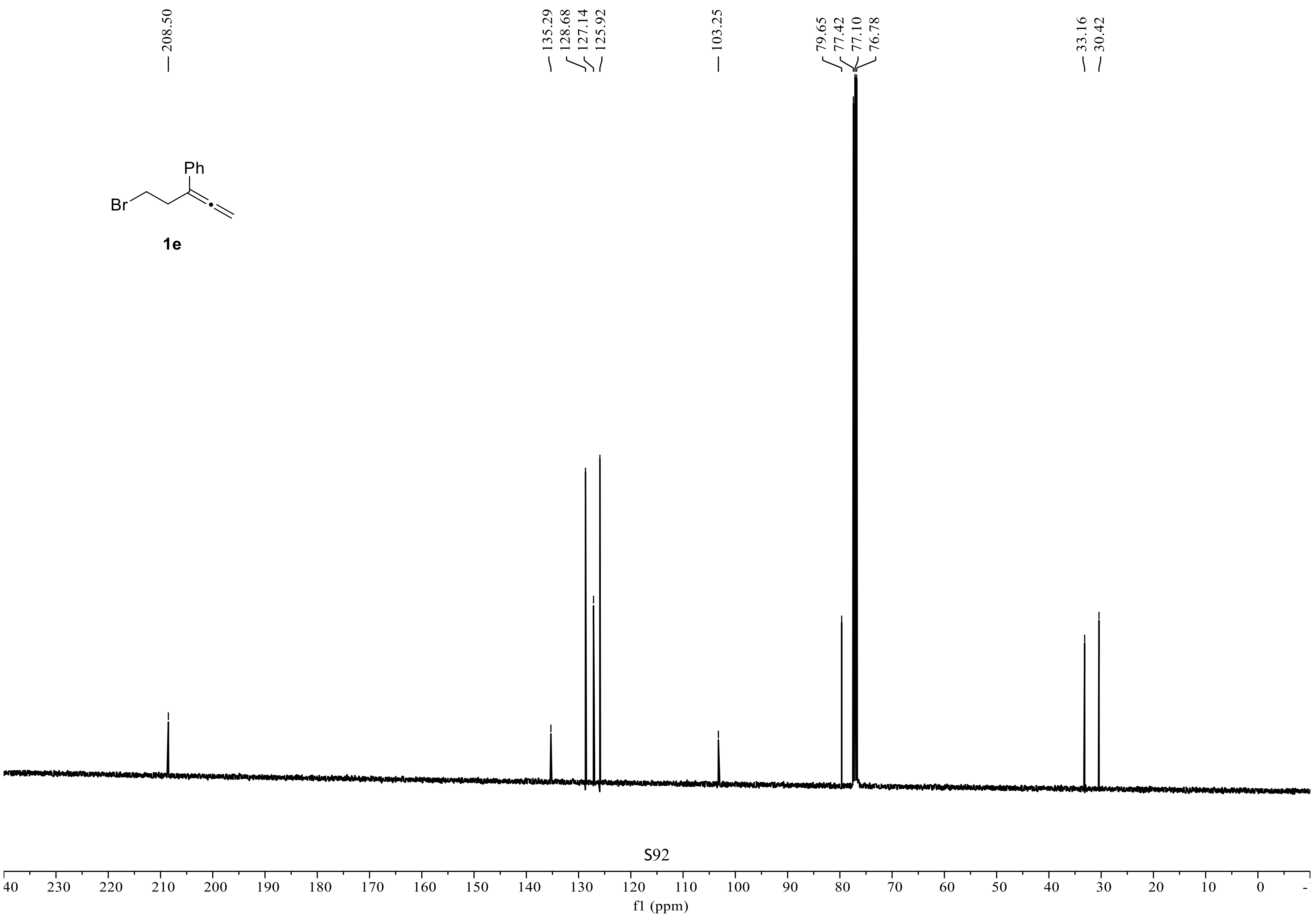
208.50
135.29
128.68
127.14
125.92
103.25
79.65
77.42
77.10
76.78
33.16
30.42
Ph
Br
1e
f1 (ppm)

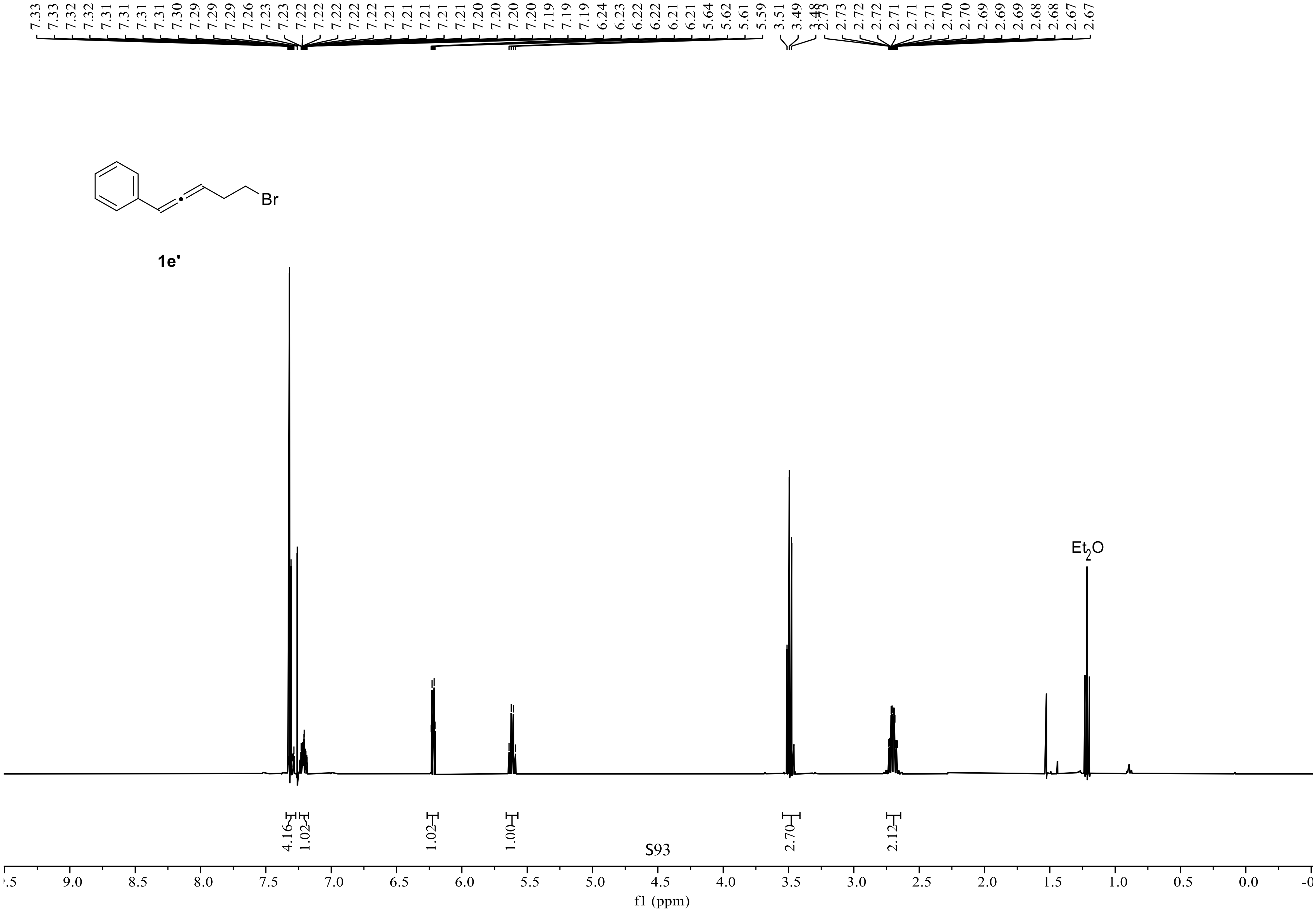
7.33 7.33 7.32 7.32 7.31 7.31 7.31 7.31 7.30 7.29 7.29 7.29 7.26 7.23 7.23 7.22 7.22 7.22 7.22 7.22 7.21 7.21 7.21 7.21 7.21 7.20 7.20 7.20 7.19 7.19 7.19 6.24 6.23 6.22 6.22 6.21 6.21 5.64 5.62 5.61 5.59 3.51 3.49 3.48 2.73 2.73 2.72 2.72 2.71 2.71 2.71 2.70 2.70 2.69 2.69 2.69 2.68 2.68 2.67 2.67
Br
1e'
Et2O
4.16 1.02 1.02 1.00 2.70 2.12
f1 (ppm)

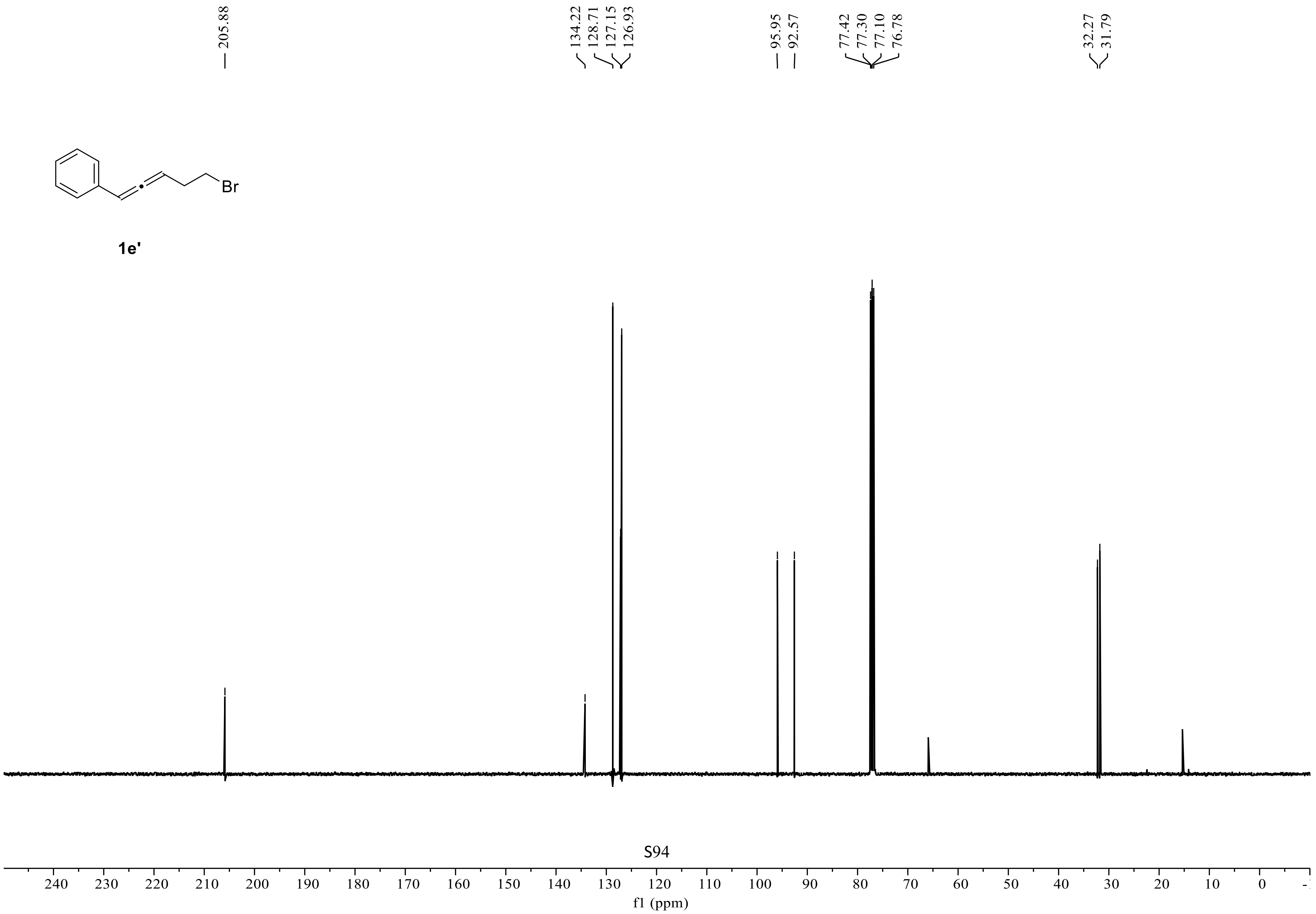
205.88
134.22
128.71
127.15
126.93
95.95
92.57
77.42
77.30
77.10
76.78
32.27
31.79
Br
240 230 220 210 200 190 180 170 160 150 140 130 120 110 100 90 80 70 60 50 40 30 20 10 0
f1 (ppm)

1e'

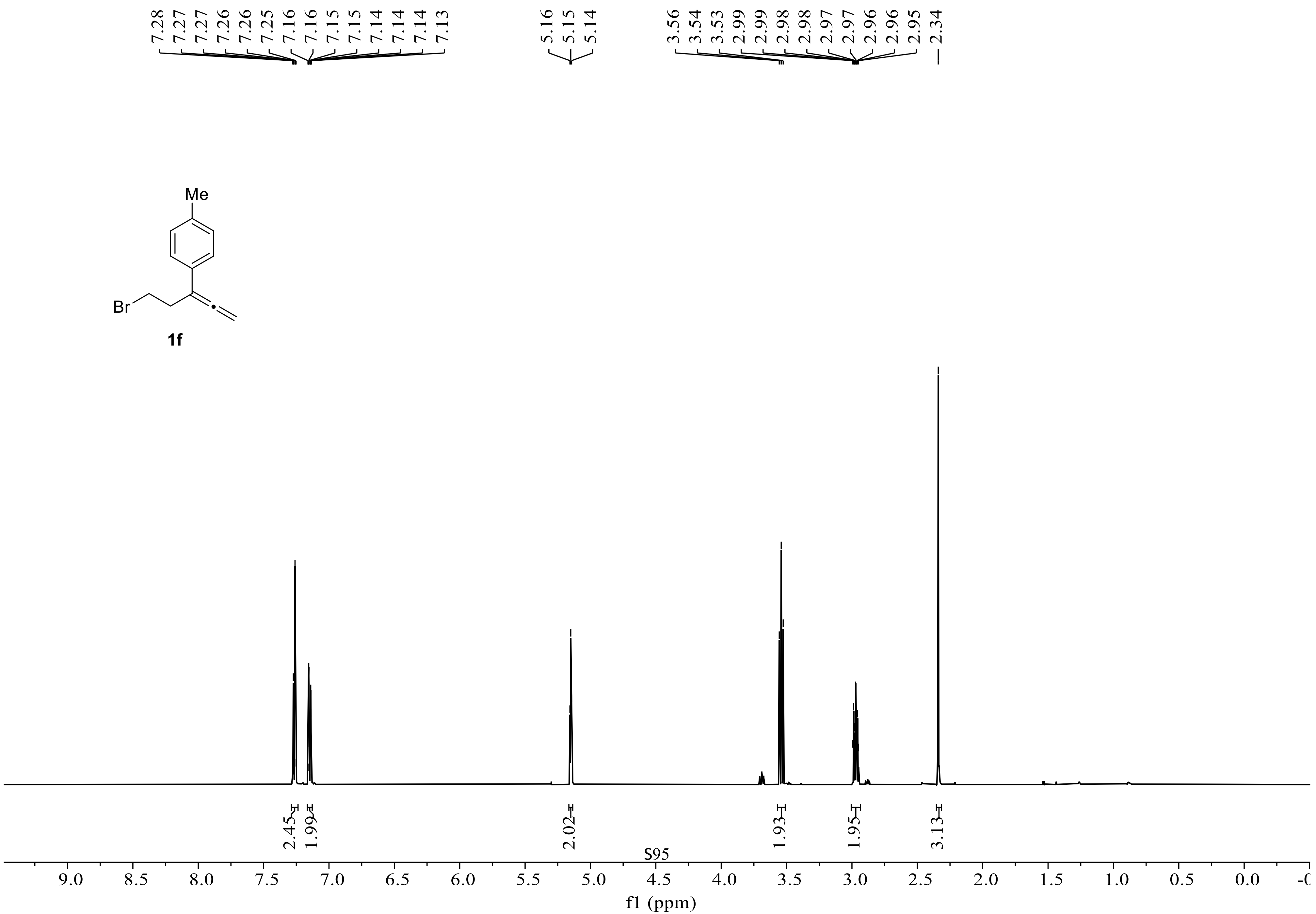

7.28 7.27 7.27 7.26 7.26 7.25 7.16 7.16 7.15 7.15 7.14 7.14 7.14 7.13
5.16 5.15 5.14
3.56 3.54 3.53 2.99 2.99 2.98 2.98 2.97 2.97 2.96 2.96 2.95 2.34
Me
Br
1f
2.45
1.99
2.02
1.93
1.95
3.13
9.0 8.5 8.0 7.5 7.0 6.5 6.0 5.5 5.0 4.5 4.0 3.5 3.0 2.5 2.0 1.5 1.0 0.5 0.0
f1 (ppm)

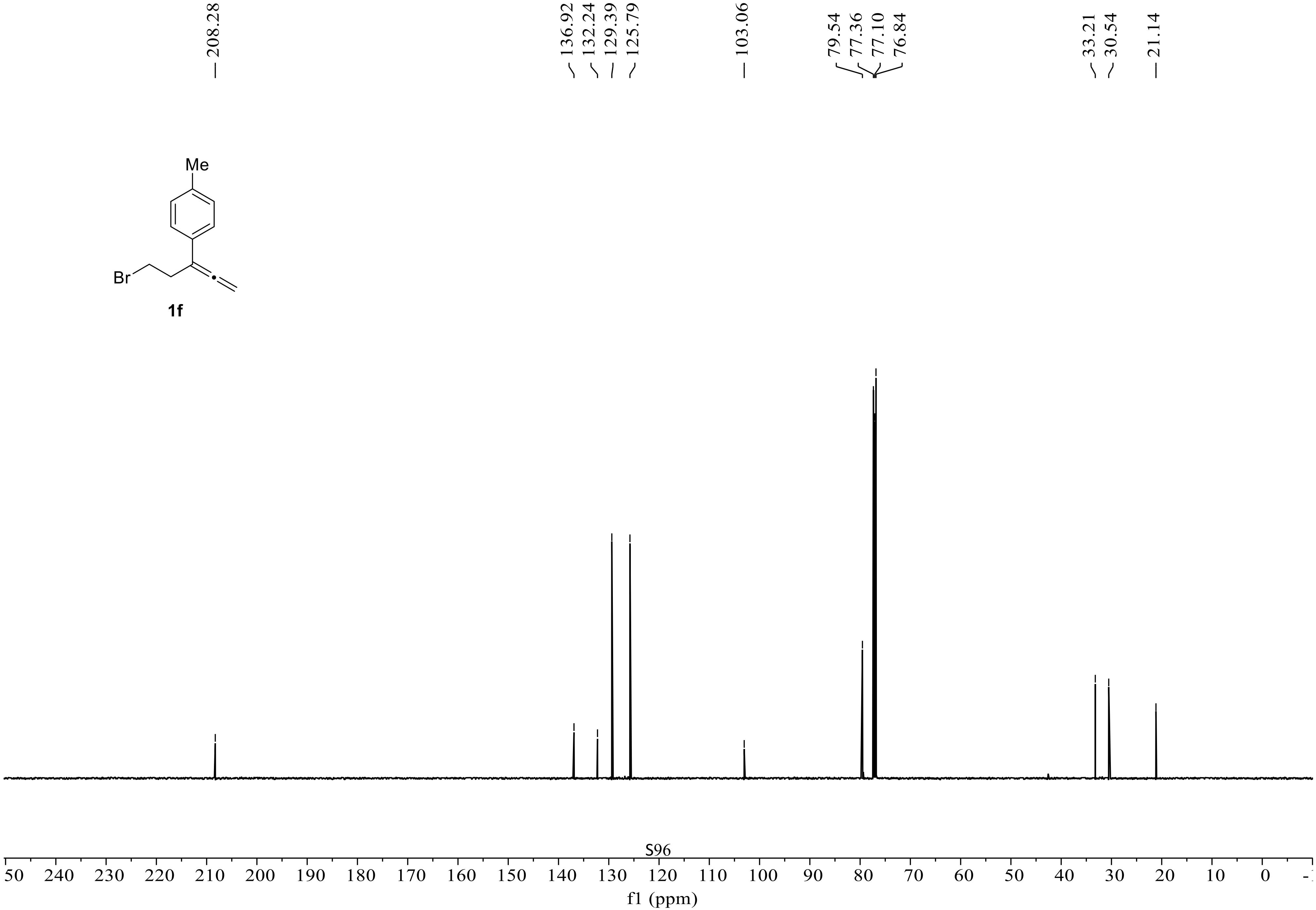
Me
Br
1f
208.28
136.92
132.24
129.39
125.79
103.06
79.54
77.36
77.10
76.84
33.21
30.54
21.14
50 240 230 220 210 200 190 180 170 160 150 140 130 120 110 100 90 80 70 60 50 40 30 20 10 0
f1 (ppm)

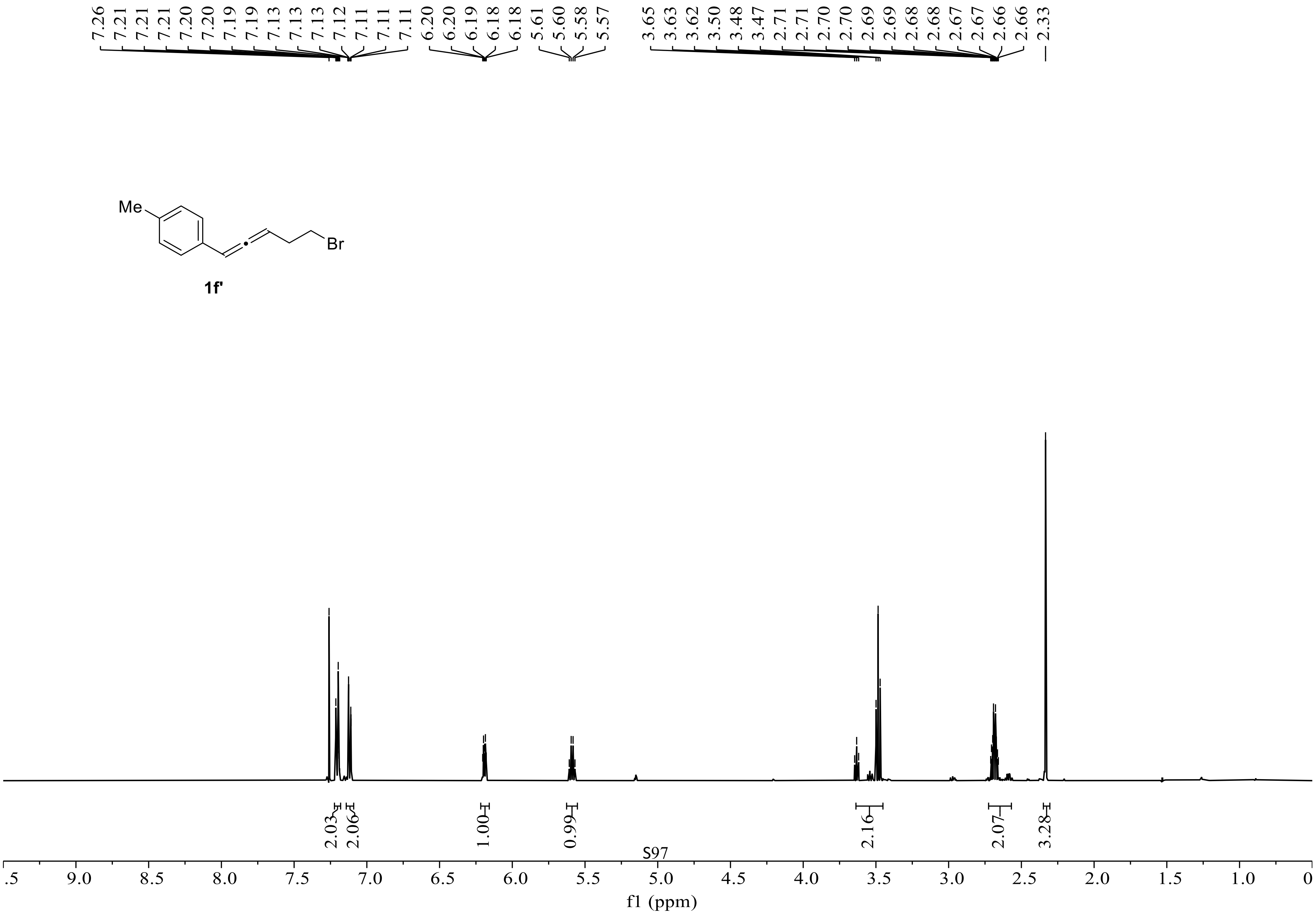
7.26 7.21 7.21 7.21 7.20 7.20 7.19 7.19 7.13 7.13 7.13 7.12 7.11 7.11 7.11
6.20 6.20 6.19 6.18 6.18
5.61 5.60 5.58 5.57
3.65 3.63 3.62 3.50 3.48 3.47 2.71 2.71 2.70 2.70 2.69 2.69 2.68 2.68 2.67 2.67 2.66 2.66
2.33
Me
Br
1f'
2.03
2.06
1.00
0.99
2.16
2.07
3.28
.5 9.0 8.5 8.0 7.5 7.0 6.5 6.0 5.5 5.0 4.5 4.0 3.5 3.0 2.5 2.0 1.5 1.0 0
f1 (ppm)

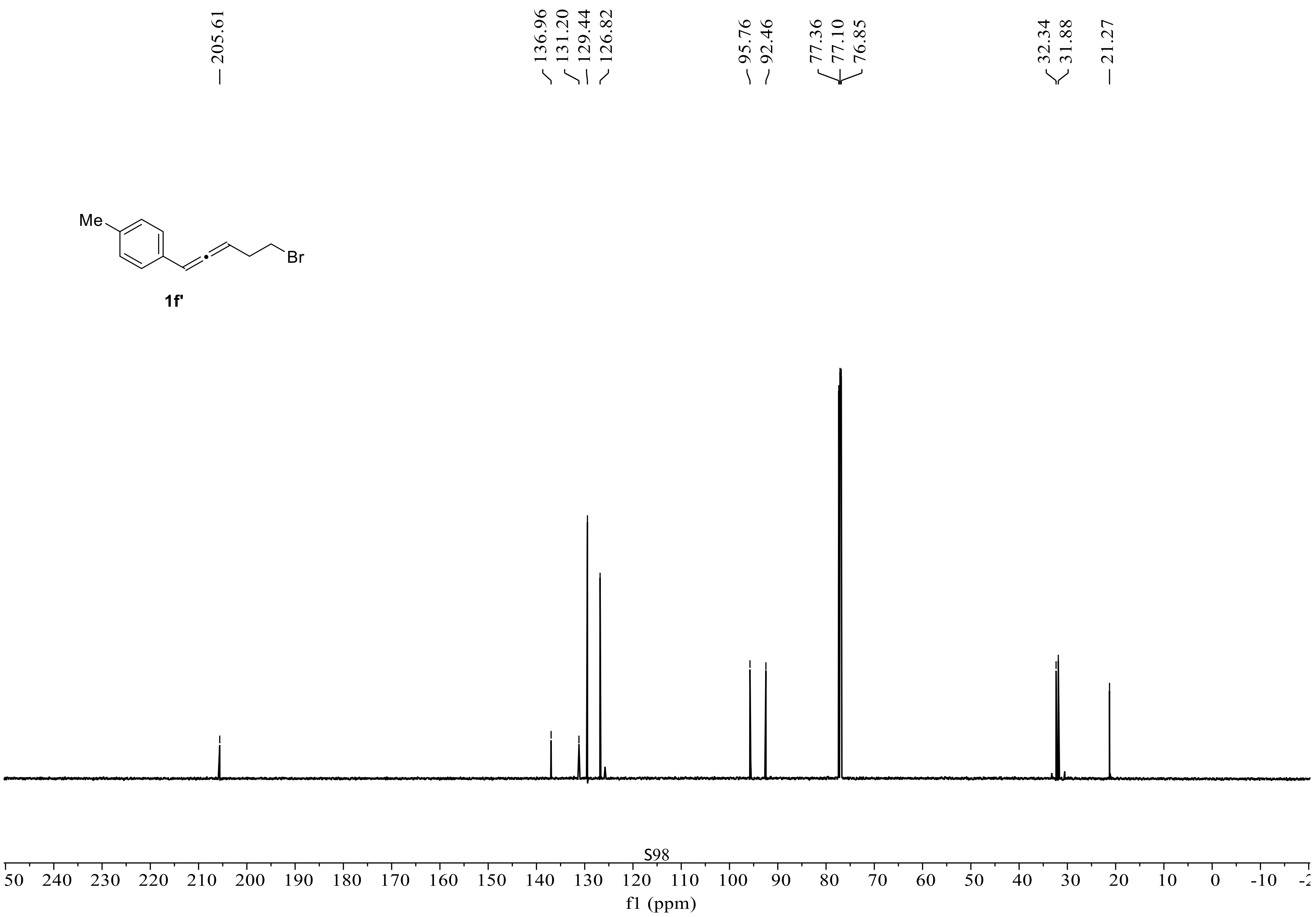

205.61
136.96
131.20
129.44
126.82
95.76
92.46
77.36
77.10
76.85
32.34
31.88
21.27
Me
Br
1f'
f1 (ppm)

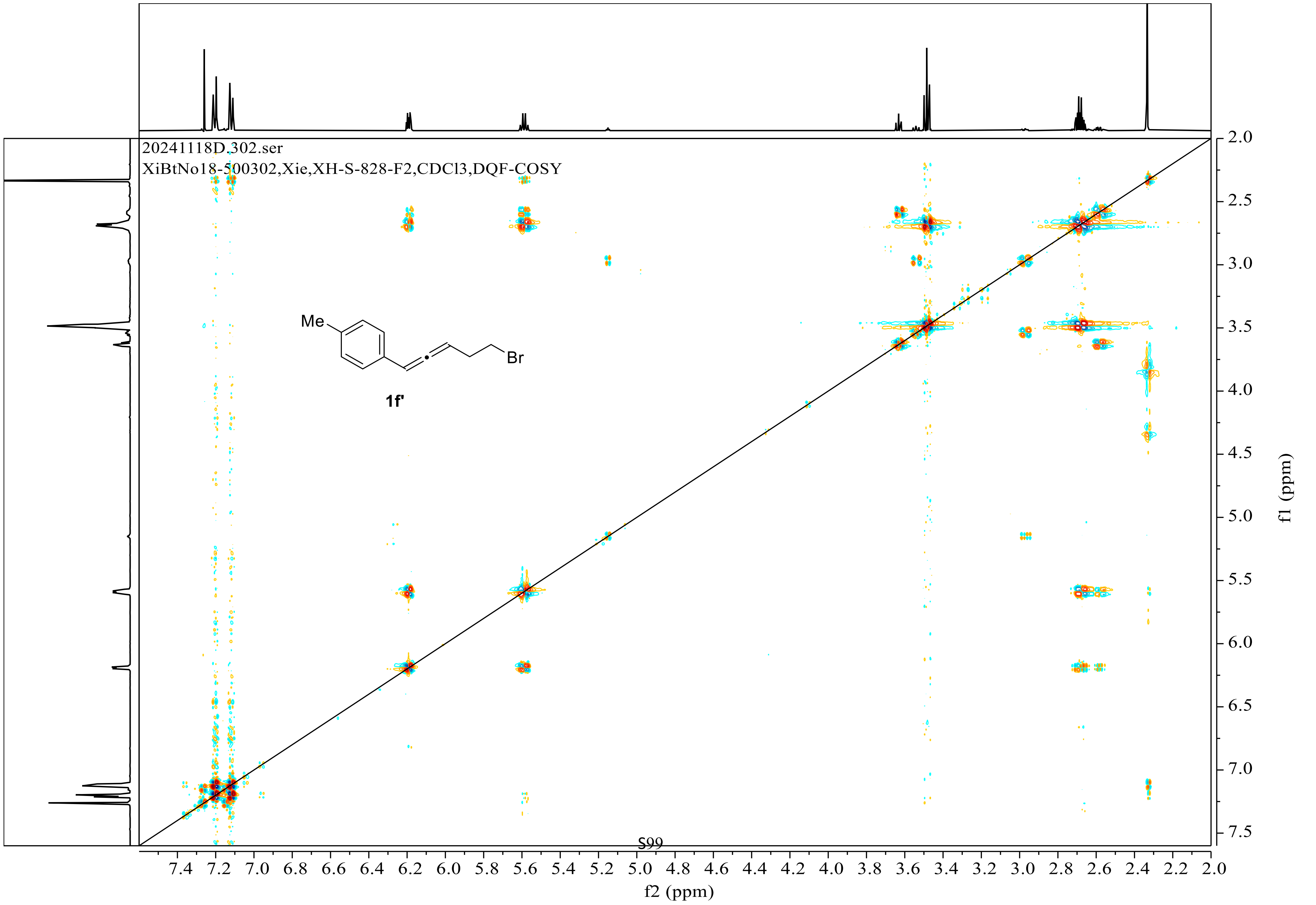
20241118D.302.ser
XiBtNo18-500302,Xie,XH-S-828-F2,CDCl3,DQF-COSY
Me
Br
1f'
f2 (ppm)
f1 (ppm)

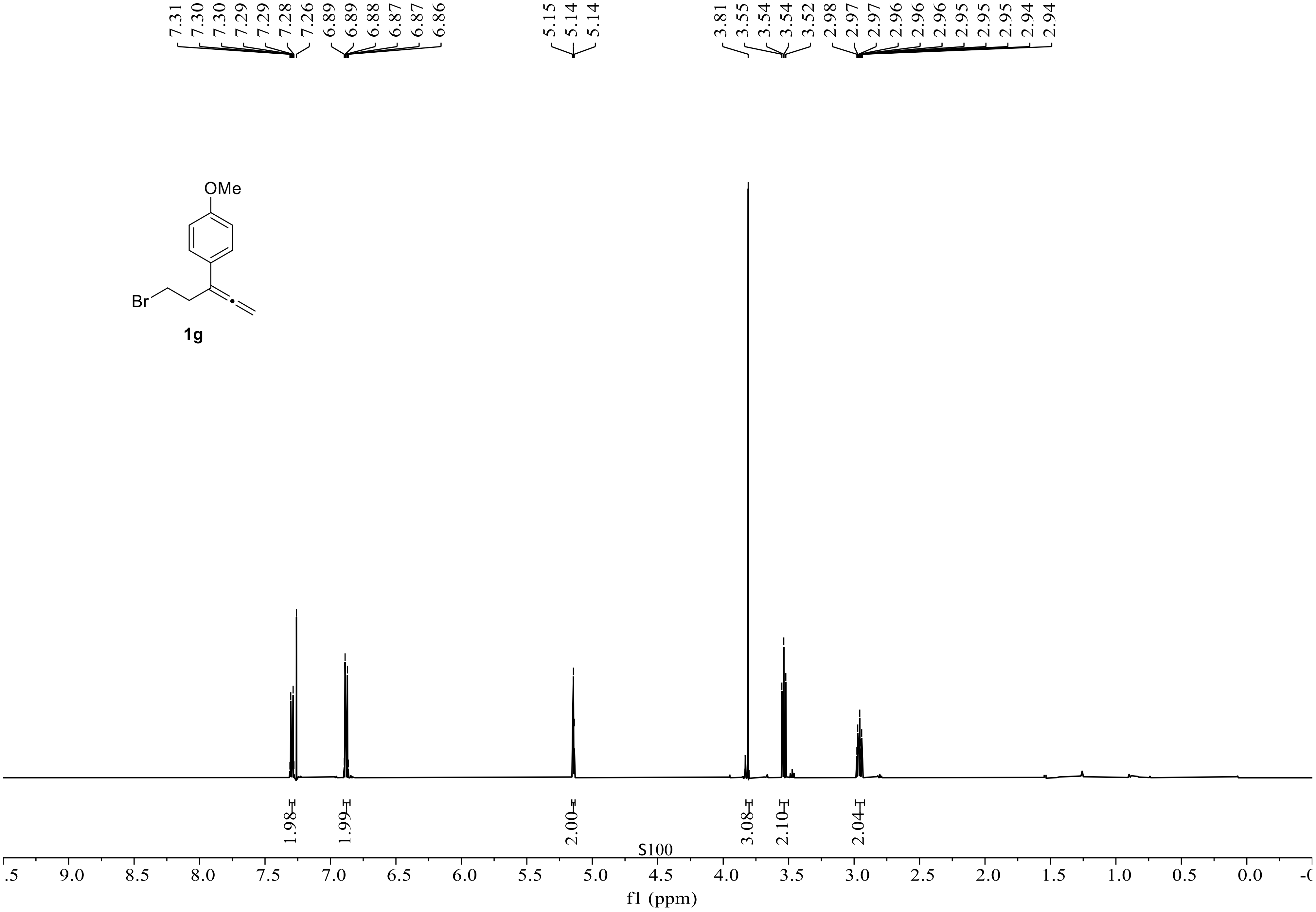
7.31
7.30
7.30
7.29
7.29
7.28
7.26
6.89
6.89
6.88
6.87
6.87
6.86
5.15
5.14
5.14
3.81
3.55
3.54
3.54
3.52
2.98
2.97
2.97
2.96
2.96
2.96
2.95
2.95
2.95
2.94
2.94
OMe
Br
1g
1.98
1.99
2.00
3.08
2.10
2.04
f1 (ppm)

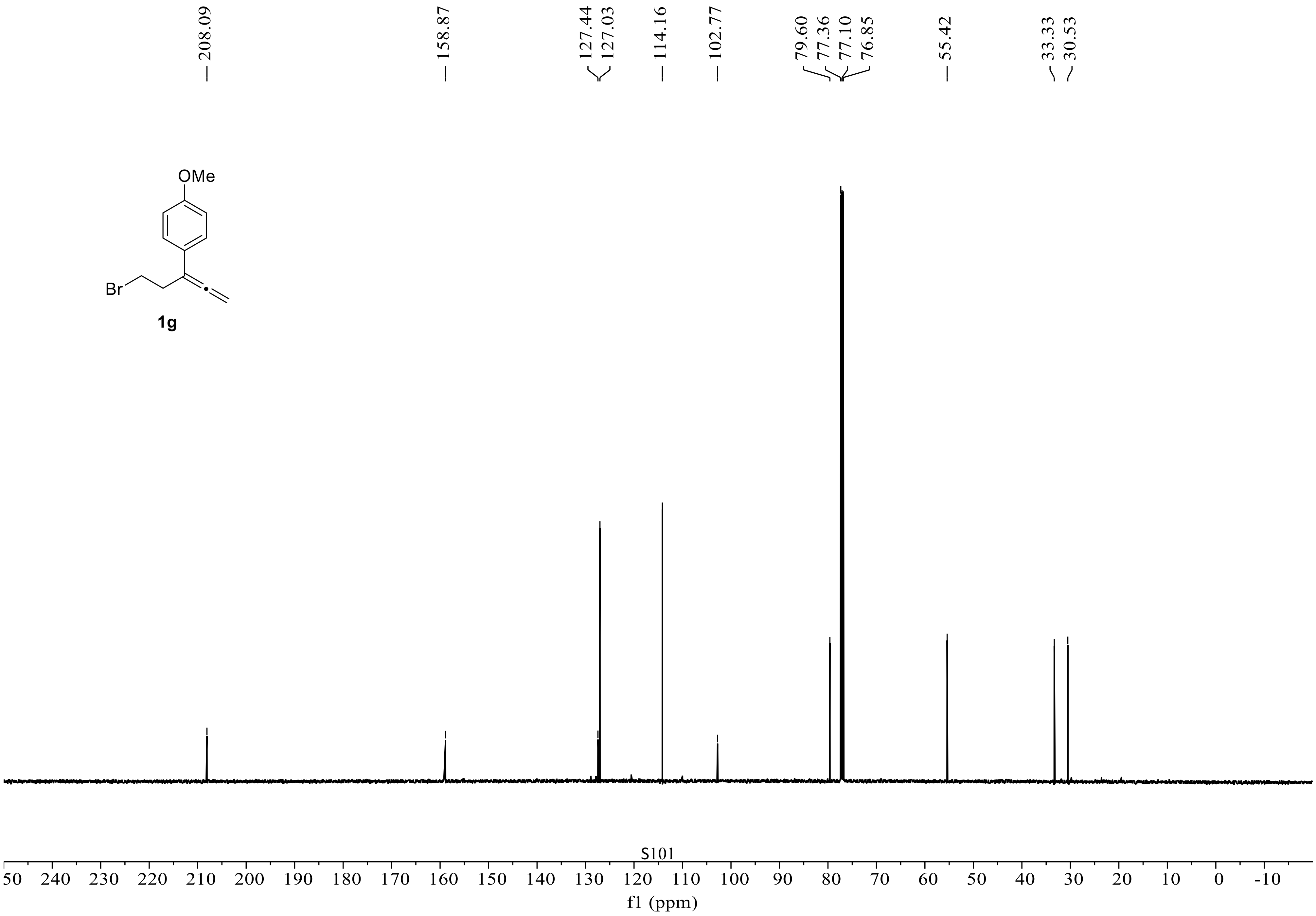
208.09
158.87
127.44
127.03
114.16
102.77
79.60
77.36
77.10
76.85
55.42
33.33
30.53
OMe
Br
1g
50 240 230 220 210 200 190 180 170 160 150 140 130 120 110 100 90 80 70 60 50 40 30 20 10 0 -10
f1 (ppm)

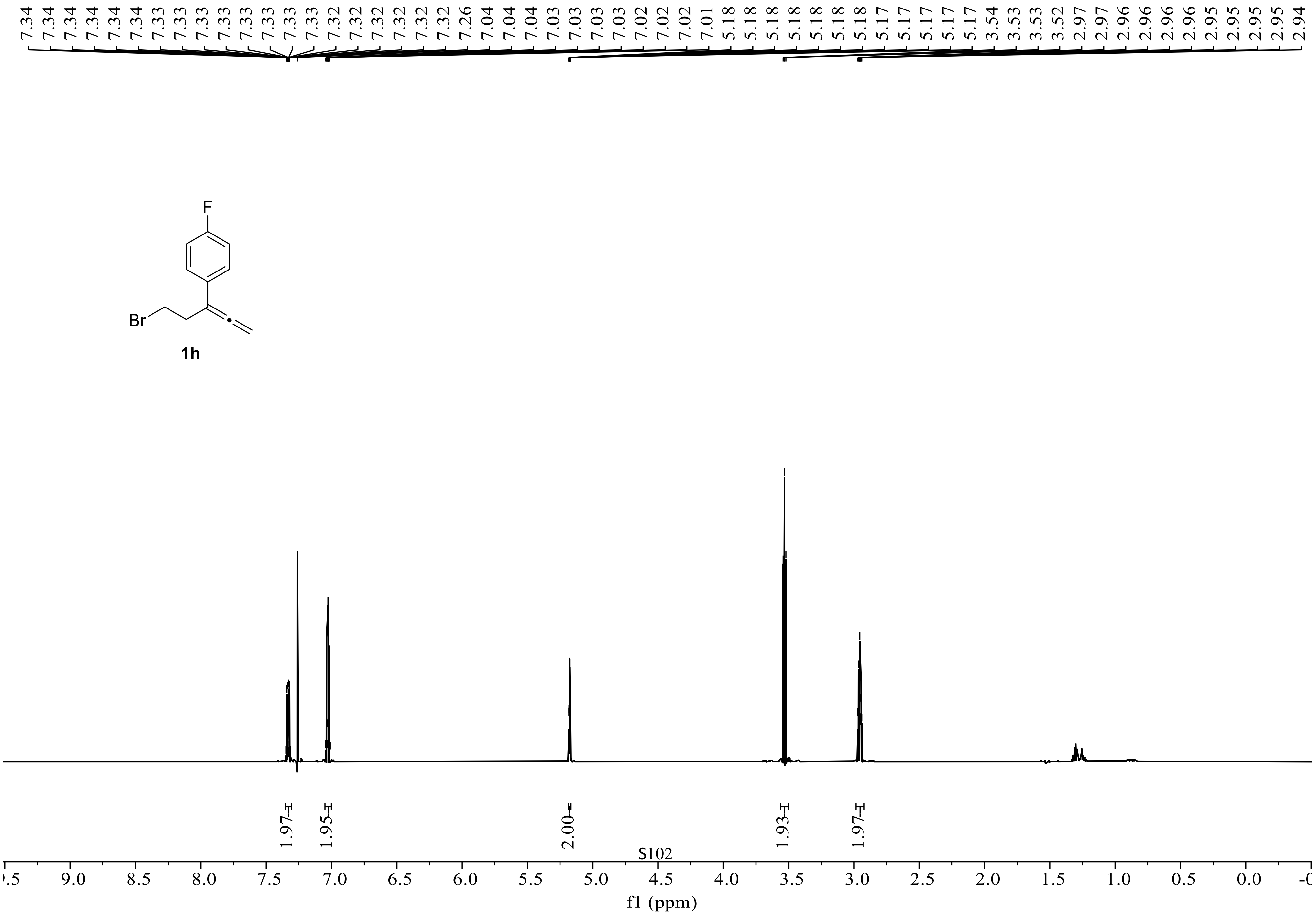
7.34 7.34 7.34 7.34 7.34 7.34 7.33 7.33 7.33 7.33 7.33 7.33 7.33 7.32 7.32 7.32 7.32 7.32 7.32 7.26 7.04 7.04 7.04 7.03 7.03 7.03 7.02 7.02 7.02 7.01 5.18 5.18 5.18 5.18 5.18 5.18 5.18 5.17 5.17 5.17 5.17 5.17 3.54 3.53 3.53 3.52 2.97 2.97 2.96 2.96 2.96 2.96 2.95 2.95 2.95 2.95 2.94
F
Br
1h
1.97
1.95
2.00
1.93
1.97
9.0 8.5 8.0 7.5 7.0 6.5 6.0 5.5 5.0 4.5 4.0 3.5 3.0 2.5 2.0 1.5 1.0 0.5 0.0
f1 (ppm)

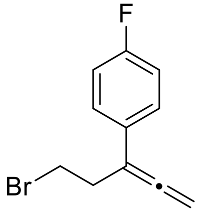

1h

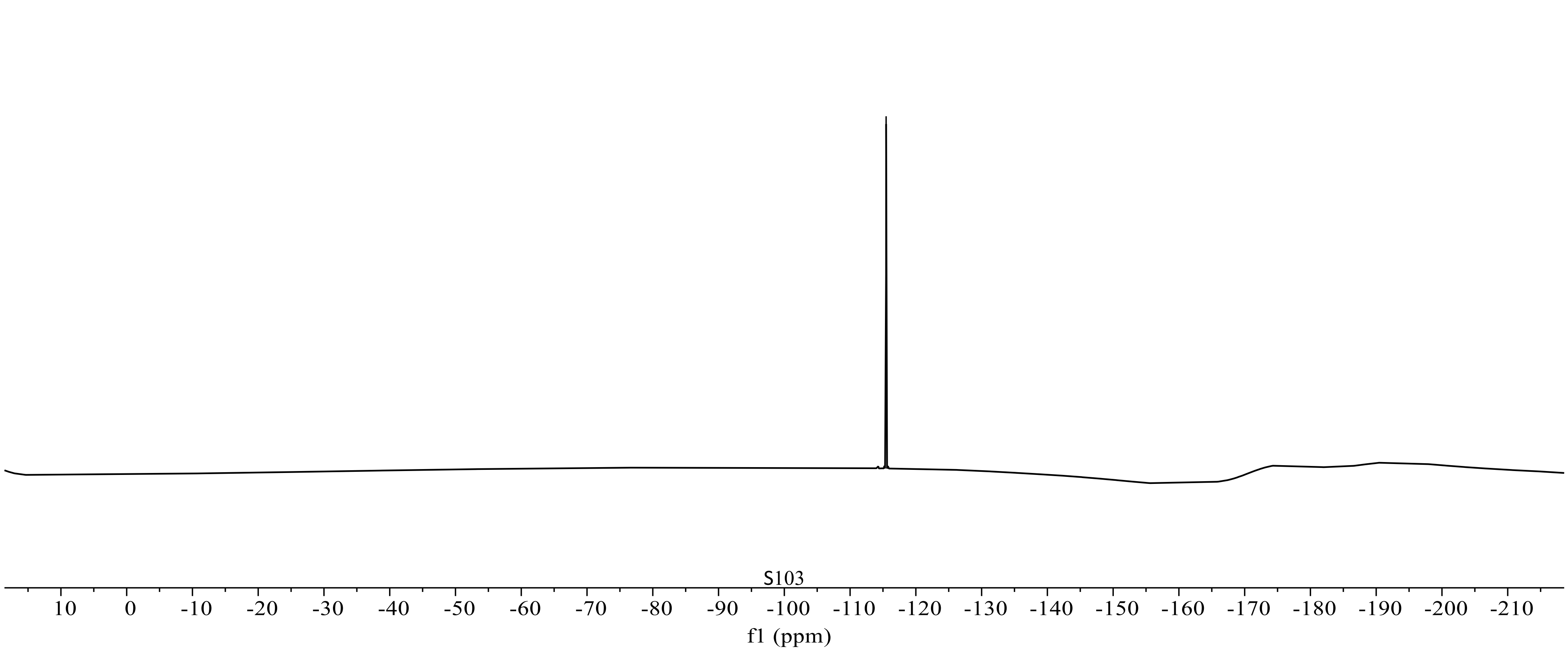
-115.48
10 0 -10 -20 -30 -40 -50 -60 -70 -80 -90 -100 -110 -120 -130 -140 -150 -160 -170 -180 -190 -200 -210
f1 (ppm)

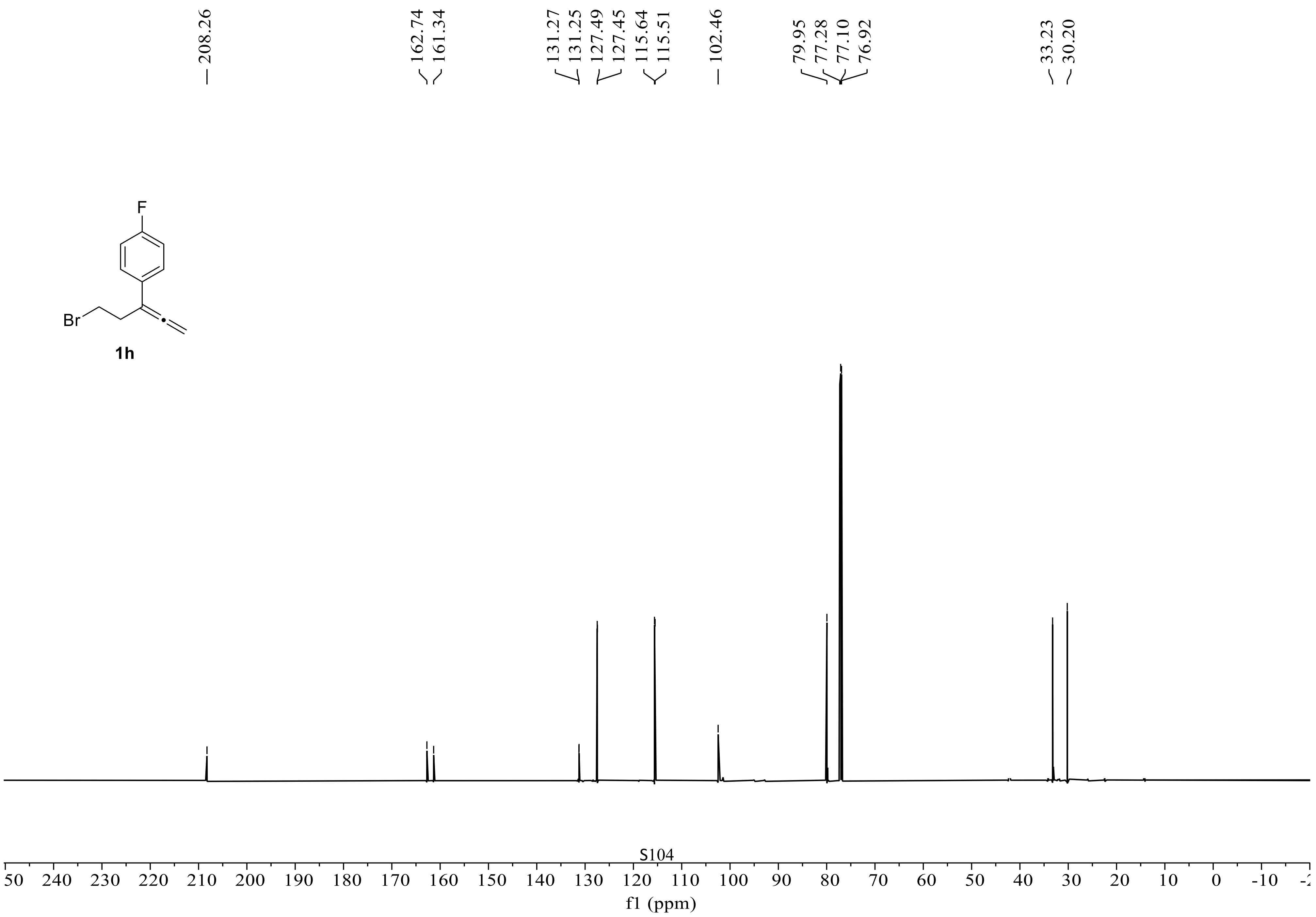
208.26
162.74
161.34
131.27
131.25
127.49
127.45
115.64
115.51
102.46
79.95
77.28
77.10
76.92
33.23
30.20
F
Br
1h
50 240 230 220 210 200 190 180 170 160 150 140 130 120 110 100 90 80 70 60 50 40 30 20 10 0 -10
f1 (ppm)

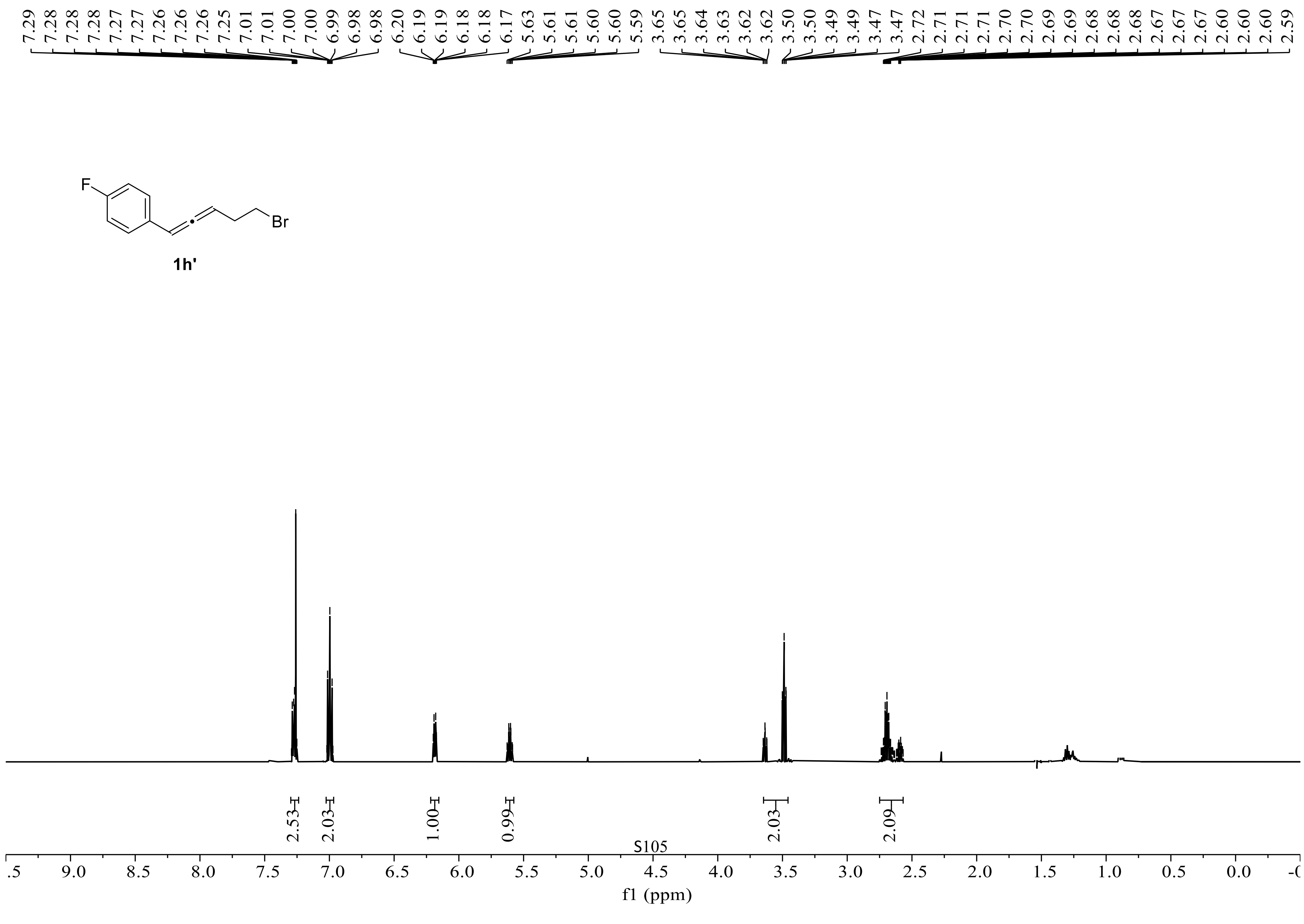

7.29 7.28 7.28 7.28 7.27 7.27 7.26 7.26 7.26 7.25 7.01 7.01 7.00 7.00 6.99 6.98 6.98 6.20 6.19 6.19 6.18 6.18 6.17 5.63 5.61 5.61 5.60 5.60 5.59 3.65 3.65 3.64 3.63 3.62 3.62 3.50 3.50 3.49 3.49 3.47 3.47 2.72 2.71 2.71 2.71 2.70 2.70 2.69 2.69 2.68 2.68 2.68 2.67 2.67 2.67 2.60 2.60 2.60 2.59
F
Br
1h'
2.53
2.03
1.00
0.99
2.03
2.09
.5 9.0 8.5 8.0 7.5 7.0 6.5 6.0 5.5 5.0 4.5 4.0 3.5 3.0 2.5 2.0 1.5 1.0 0.5 0.0
f1 (ppm)

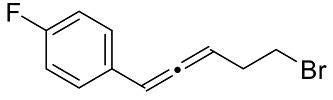
F
Br

1h'

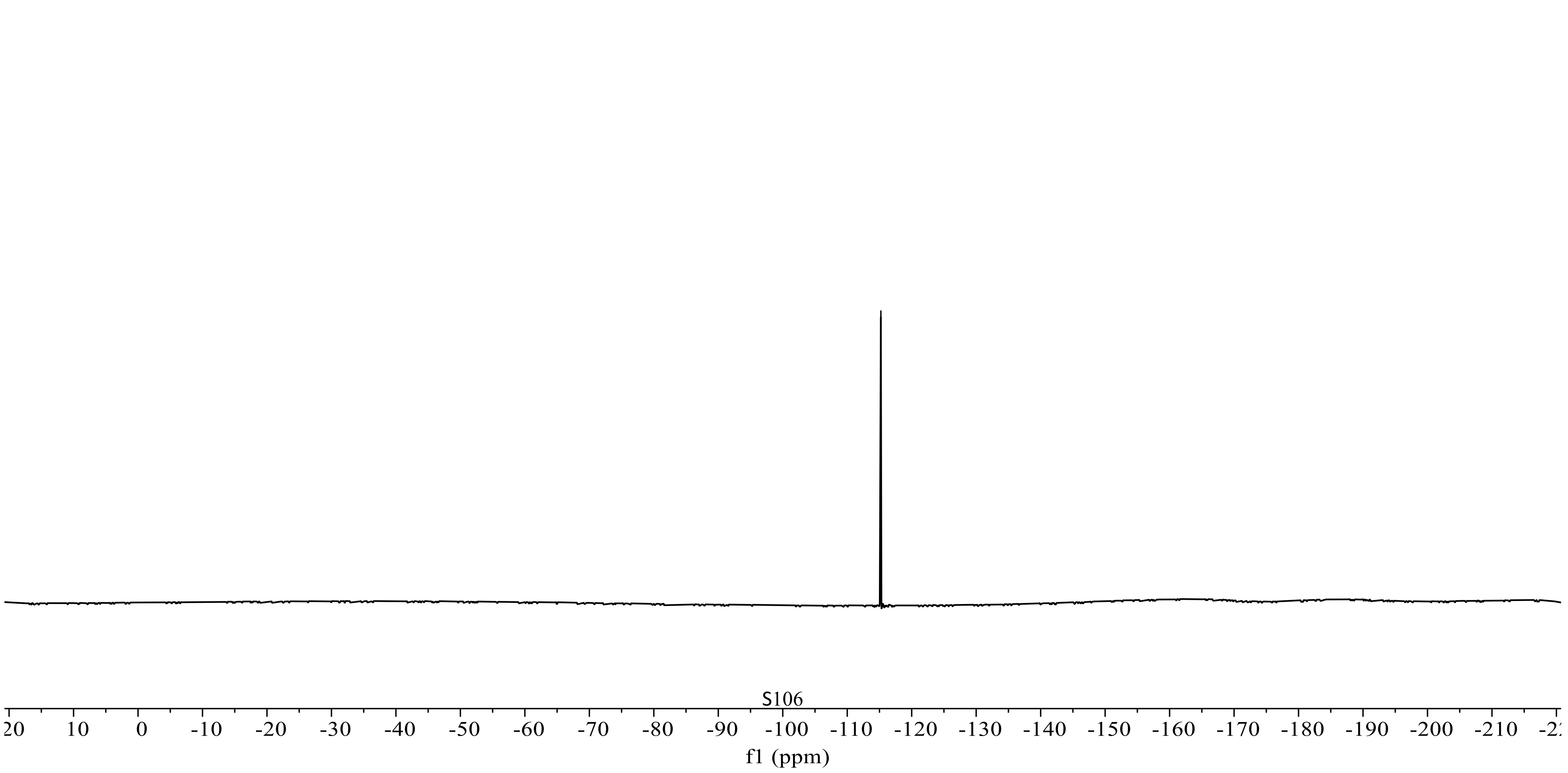
-115.22
20 10 0 -10 -20 -30 -40 -50 -60 -70 -80 -90 -100 -110 -120 -130 -140 -150 -160 -170 -180 -190 -200 -210
f1 (ppm)

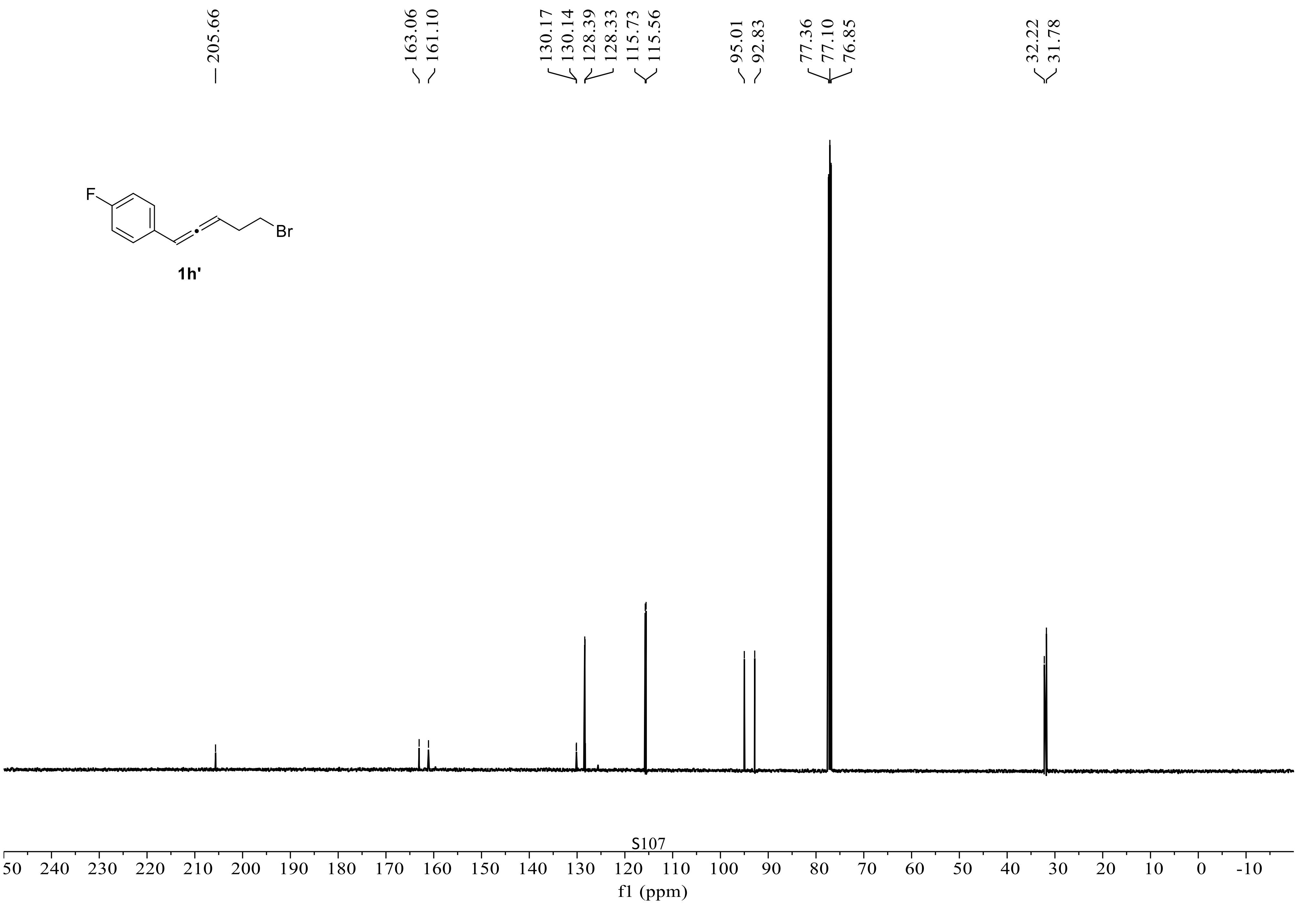
205.66
163.06
161.10
130.17
130.14
128.39
128.33
115.73
115.56
95.01
92.83
77.36
77.10
76.85
32.22
31.78
F
Br
1h'
50 240 230 220 210 200 190 180 170 160 150 140 130 120 110 100 90 80 70 60 50 40 30 20 10 0 -10
f1 (ppm)

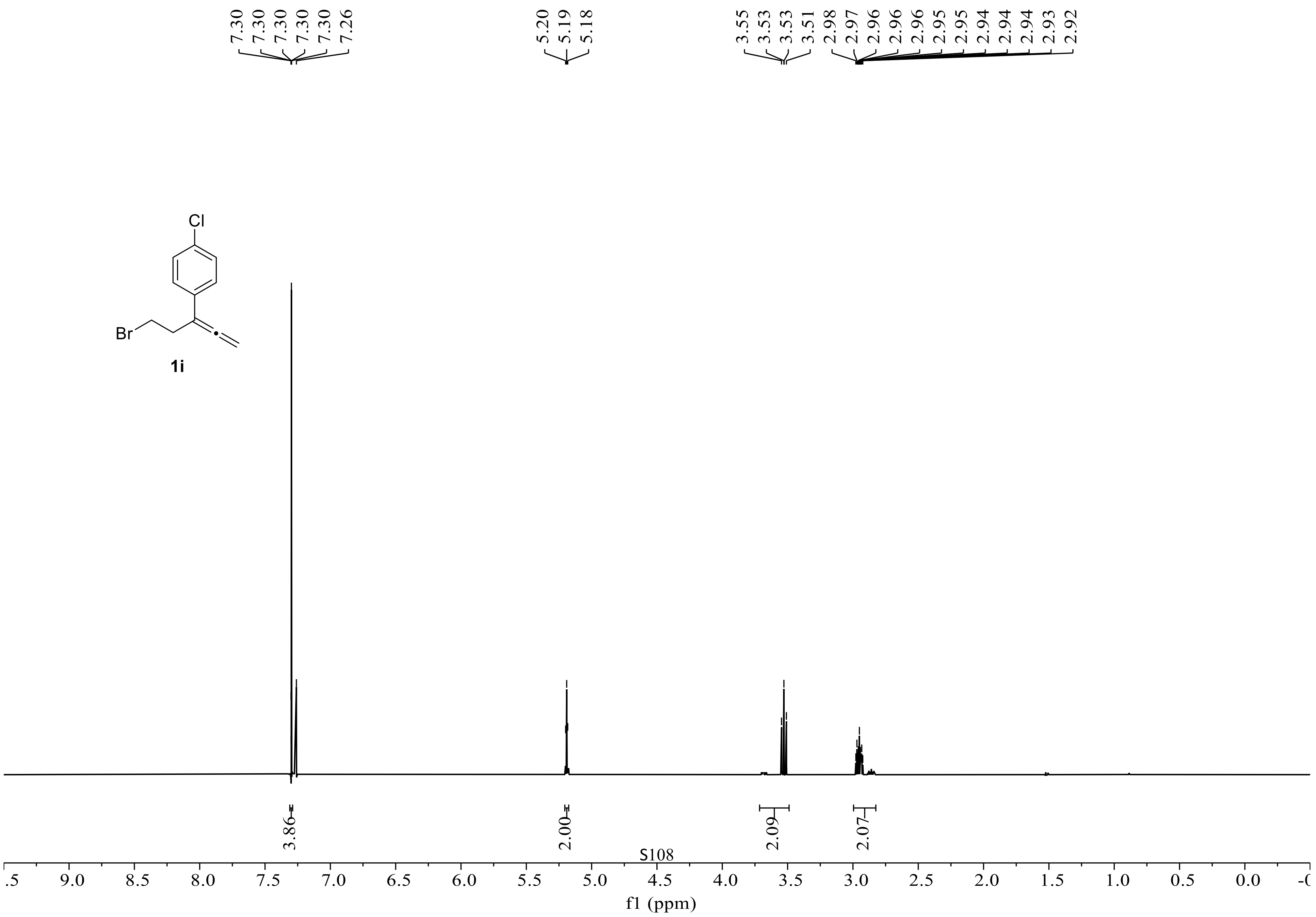

7.30
7.30
7.30
7.30
7.30
7.26
5.20
5.19
5.18
3.55
3.53
3.53
3.51
2.98
2.97
2.96
2.96
2.96
2.95
2.95
2.94
2.94
2.94
2.93
2.92
Cl
Br
1i
3.86
2.00
2.09
2.07
9.0
8.5
8.0
7.5
7.0
6.5
6.0
5.5
5.0
4.5
4.0
3.5
3.0
2.5
2.0
1.5
1.0
0.5
0.0
f1 (ppm)

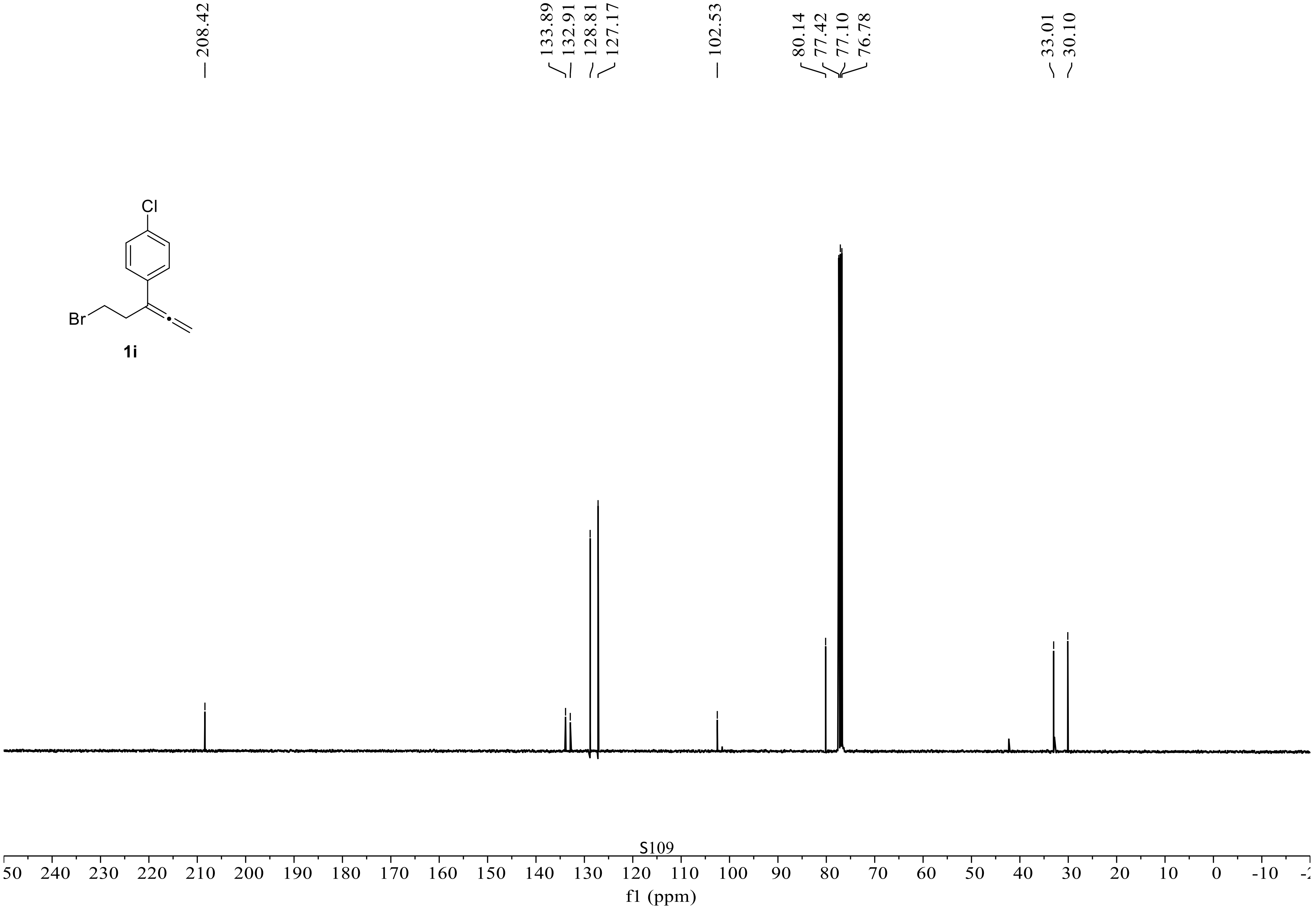
208.42
133.89
132.91
128.81
127.17
102.53
80.14
77.42
77.10
76.78
33.01
30.10
Cl
Br
1i
50 240 230 220 210 200 190 180 170 160 150 140 130 120 110 100 90 80 70 60 50 40 30 20 10 0 -10
f1 (ppm)

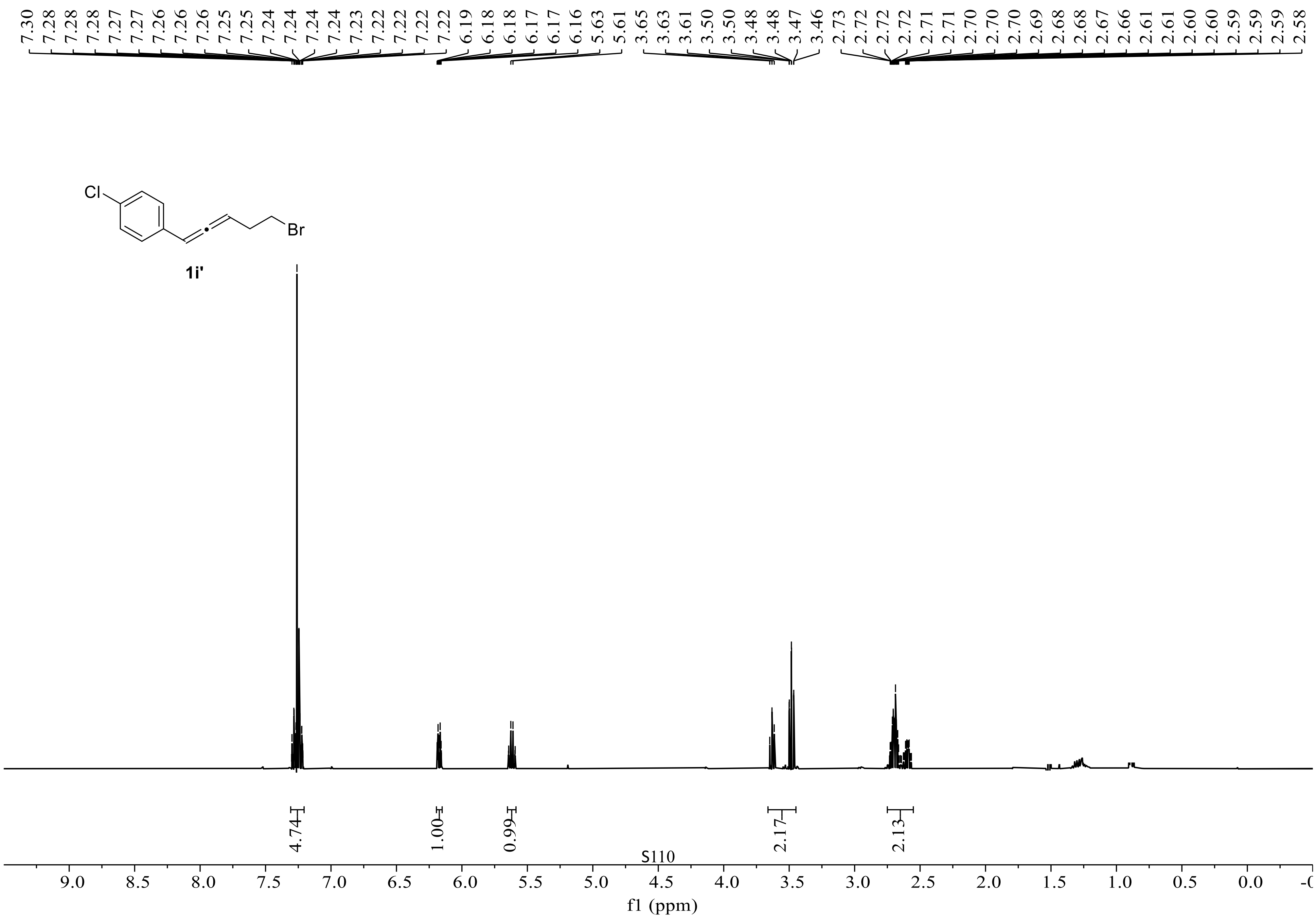
7.30
7.28
7.28
7.28
7.27
7.27
7.26
7.26
7.26
7.25
7.25
7.24
7.24
7.24
7.24
7.23
7.22
7.22
7.22
7.22
6.19
6.18
6.18
6.17
6.17
6.16
5.63
5.61
3.65
3.63
3.61
3.50
3.50
3.48
3.48
3.47
3.46
2.73
2.72
2.72
2.72
2.71
2.71
2.70
2.70
2.70
2.69
2.68
2.68
2.67
2.66
2.61
2.61
2.60
2.60
2.59
2.59
2.59
2.58
Cl
Br
1i'
4.74
1.00
0.99
2.17
2.13
9.0
8.5
8.0
7.5
7.0
6.5
6.0
5.5
5.0
4.5
4.0
3.5
3.0
2.5
2.0
1.5
1.0
0.5
0.0
f1 (ppm)

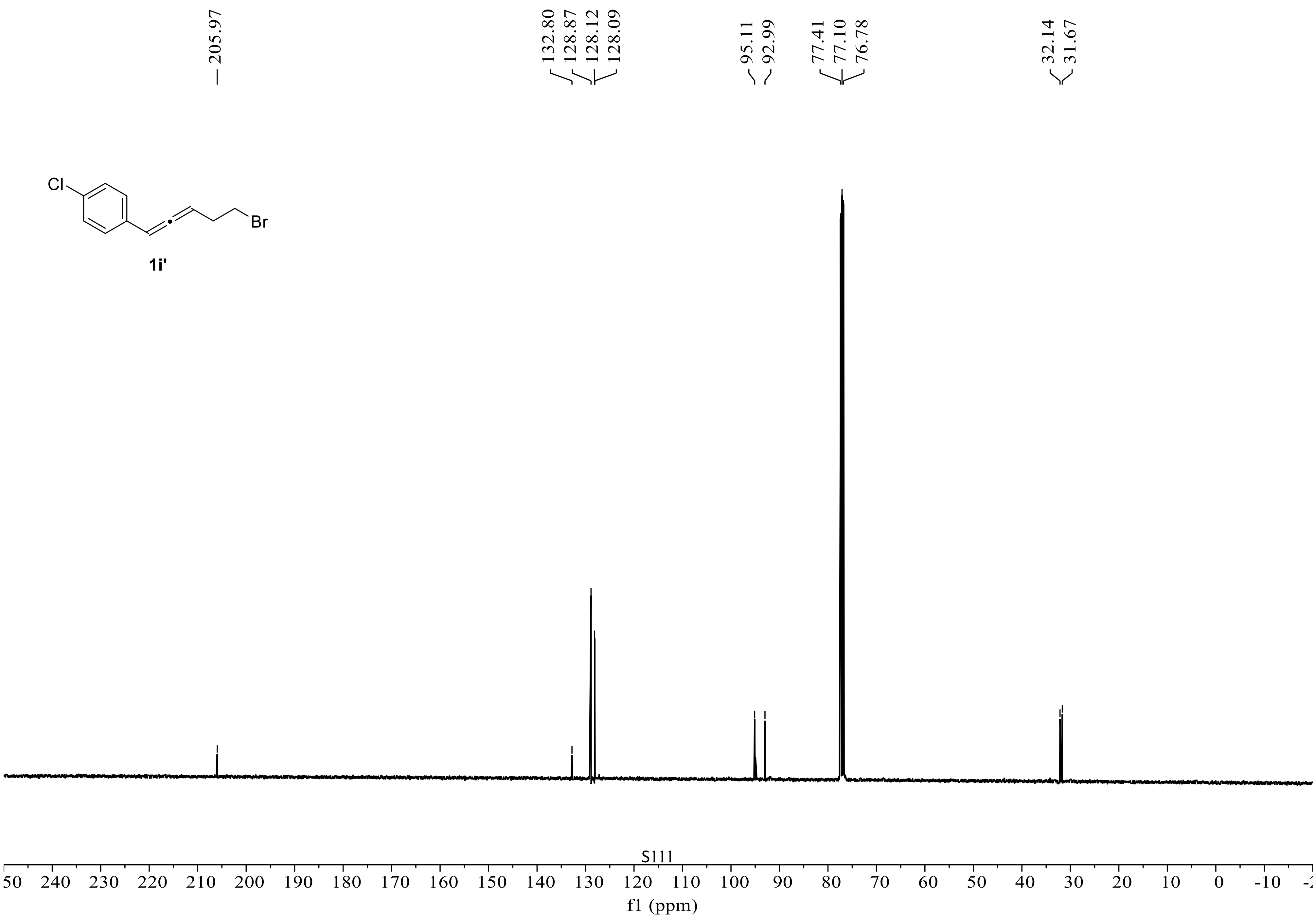
205.97
132.80
128.87
128.12
128.09
95.11
92.99
77.41
77.10
76.78
32.14
31.67
Cl
Br
1i'
f1 (ppm)

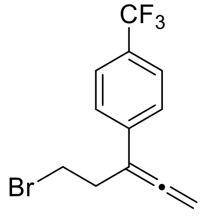

1j

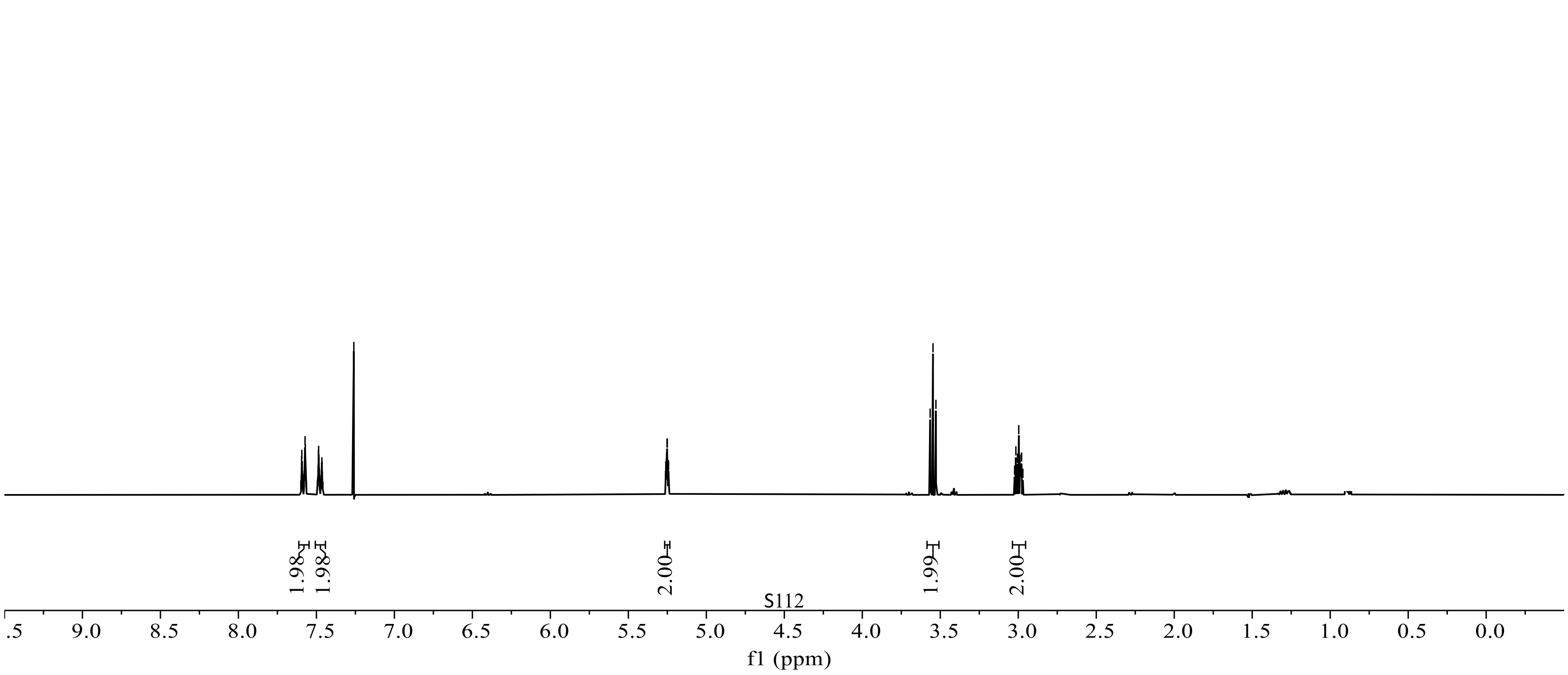
7.60 7.60 7.60 7.59 7.59 7.59 7.59 7.59 7.58 7.58 7.58 7.57 7.57 7.57 7.57 7.56 7.49 7.49 7.49 7.49 7.49 7.48 7.48 7.48 7.47 7.47 7.47 7.47 7.46 7.46 7.46 7.26 5.26 5.26 5.26 5.26 5.25 5.25 5.25 5.25 5.25 5.24 5.24 5.24 3.56 3.55 3.55 3.53 3.02 3.02 3.01 3.00 3.00 2.99 2.99 2.98 2.97
1.98
1.98
2.00
1.99
2.00
.5 9.0 8.5 8.0 7.5 7.0 6.5 6.0 5.5 5.0 4.5 4.0 3.5 3.0 2.5 2.0 1.5 1.0 0.5 0.0
f1 (ppm)

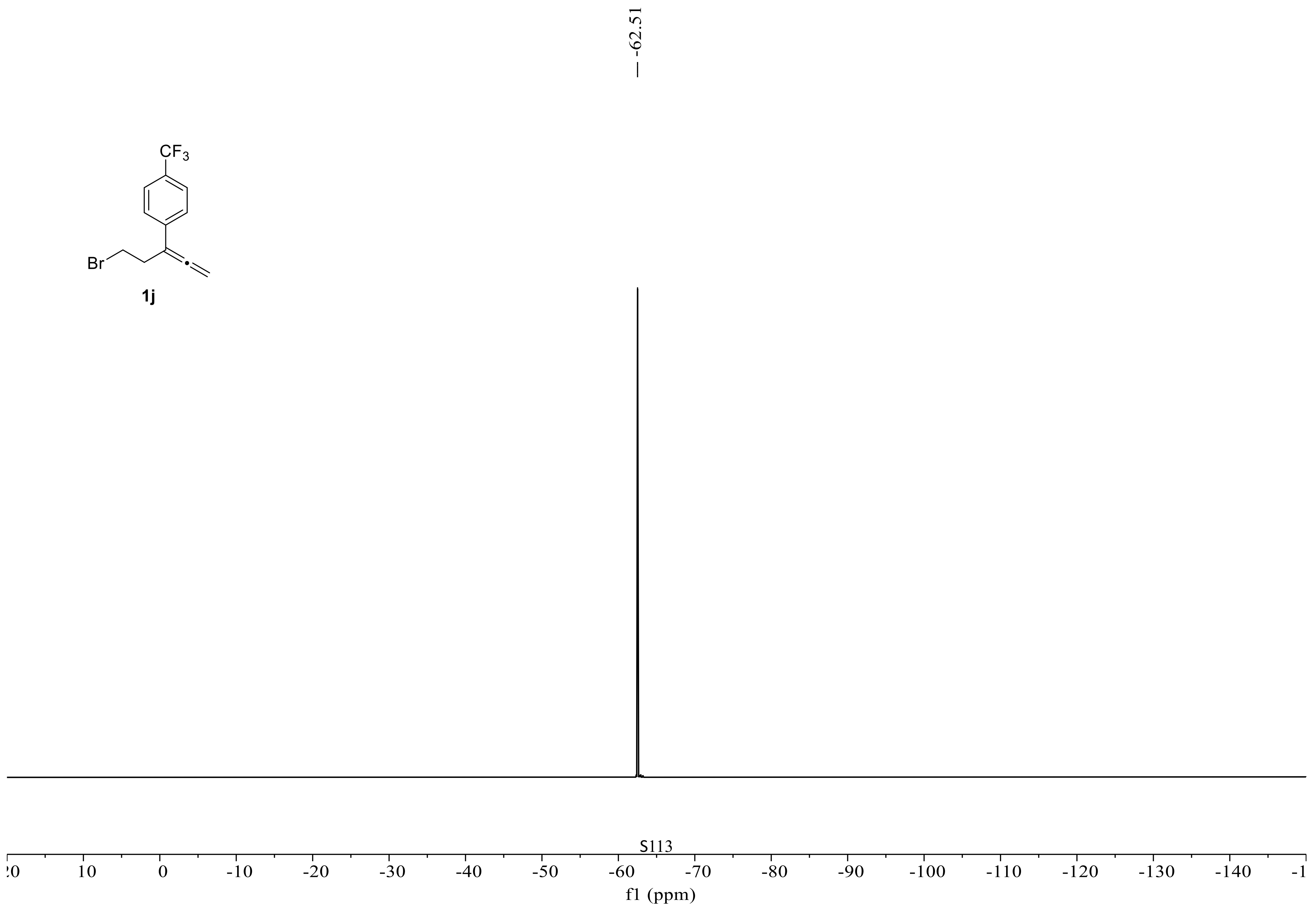
-62.51
CF3
Br
1j
10
0
-10
-20
-30
-40
-50
-60
-70
-80
-90
-100
-110
-120
-130
-140
f1 (ppm)

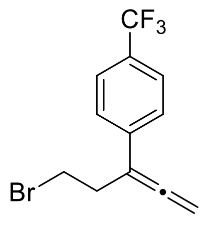
CF3
Br

1j

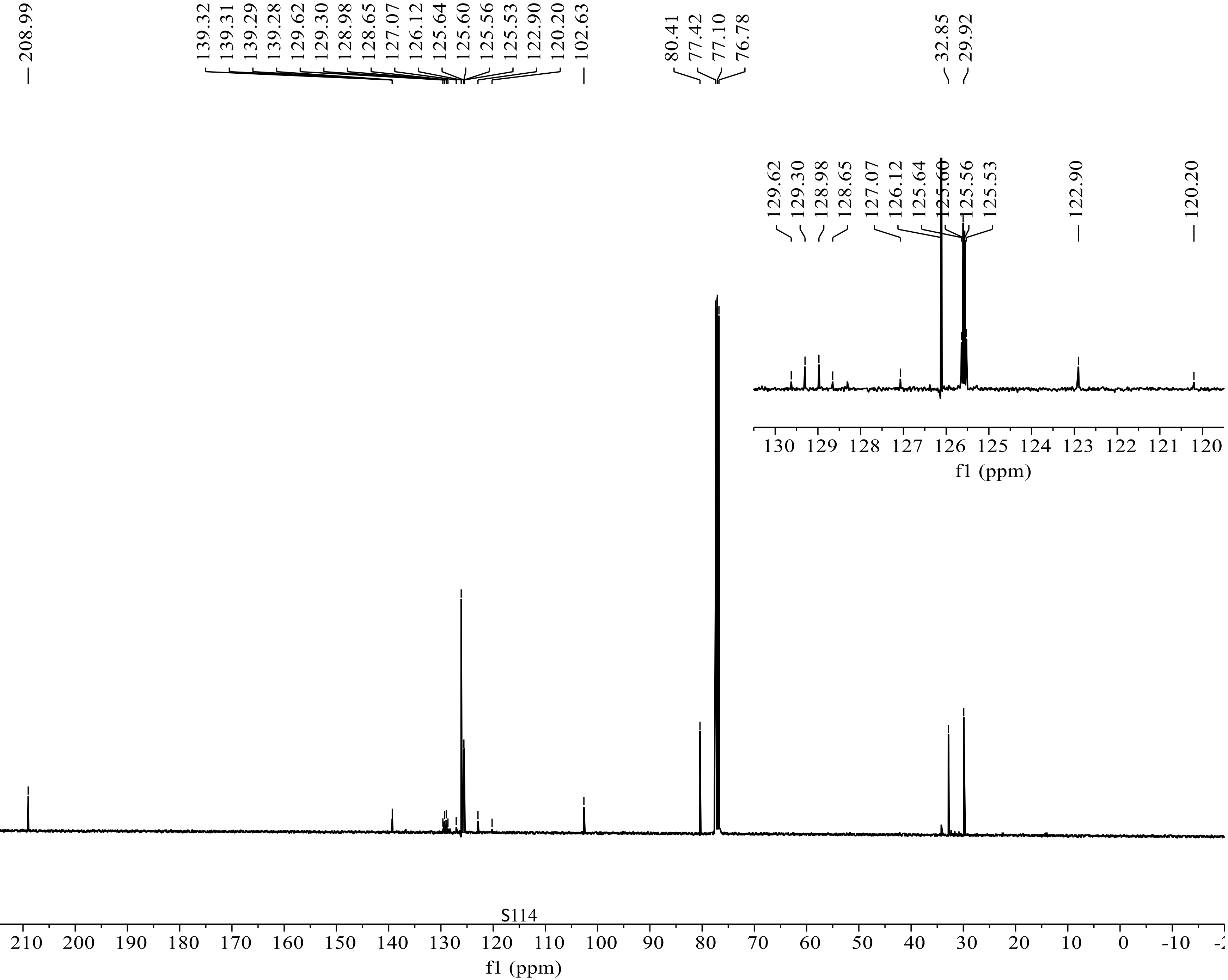
208.99
139.32
139.31
139.29
139.28
129.62
129.30
128.98
128.65
127.07
126.12
125.64
125.60
125.56
125.53
122.90
120.20
102.63
80.41
77.42
77.10
76.78
32.85
29.92
129.62
129.30
128.98
128.65
127.07
126.12
125.64
125.60
125.56
125.53
122.90
120.20
130 129 128 127 126 125 124 123 122 121 120
f1 (ppm)
240 230 220 210 200 190 180 170 160 150 140 130 120 110 100 90 80 70 60 50 40 30 20 10 0 -10
f1 (ppm)

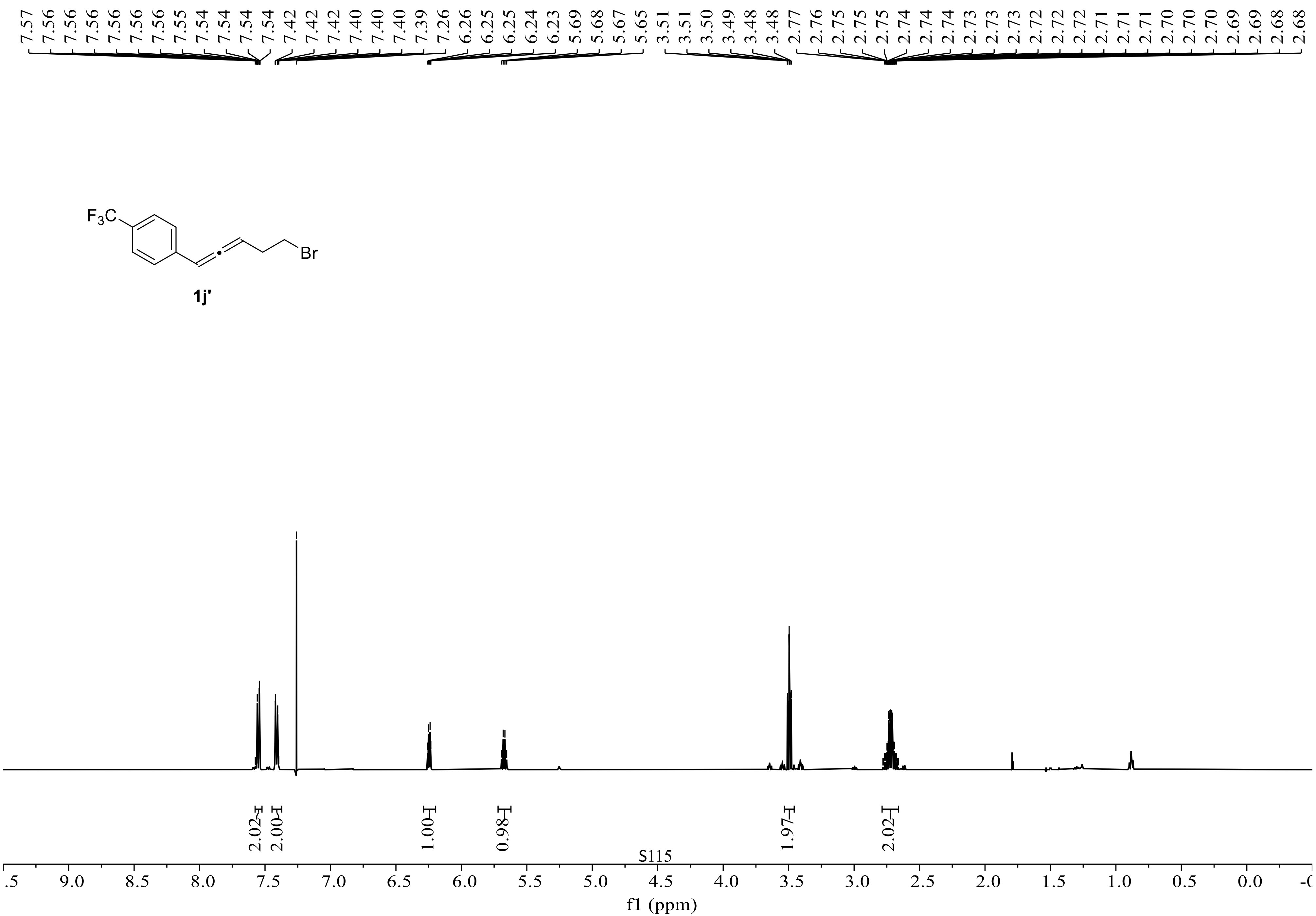
7.57
7.56
7.56
7.56
7.56
7.56
7.56
7.55
7.54
7.54
7.54
7.54
7.42
7.42
7.42
7.40
7.40
7.40
7.39
7.26
6.26
6.25
6.25
6.24
6.23
5.69
5.68
5.67
5.65
3.51
3.51
3.50
3.49
3.48
3.48
2.77
2.76
2.75
2.75
2.75
2.74
2.74
2.74
2.73
2.73
2.73
2.72
2.72
2.72
2.71
2.71
2.71
2.70
2.70
2.70
2.69
2.69
2.68
2.68
F3C
Br
1j'
2.02
2.00
1.00
0.98
1.97
2.02
f1 (ppm)

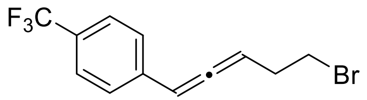

1j'

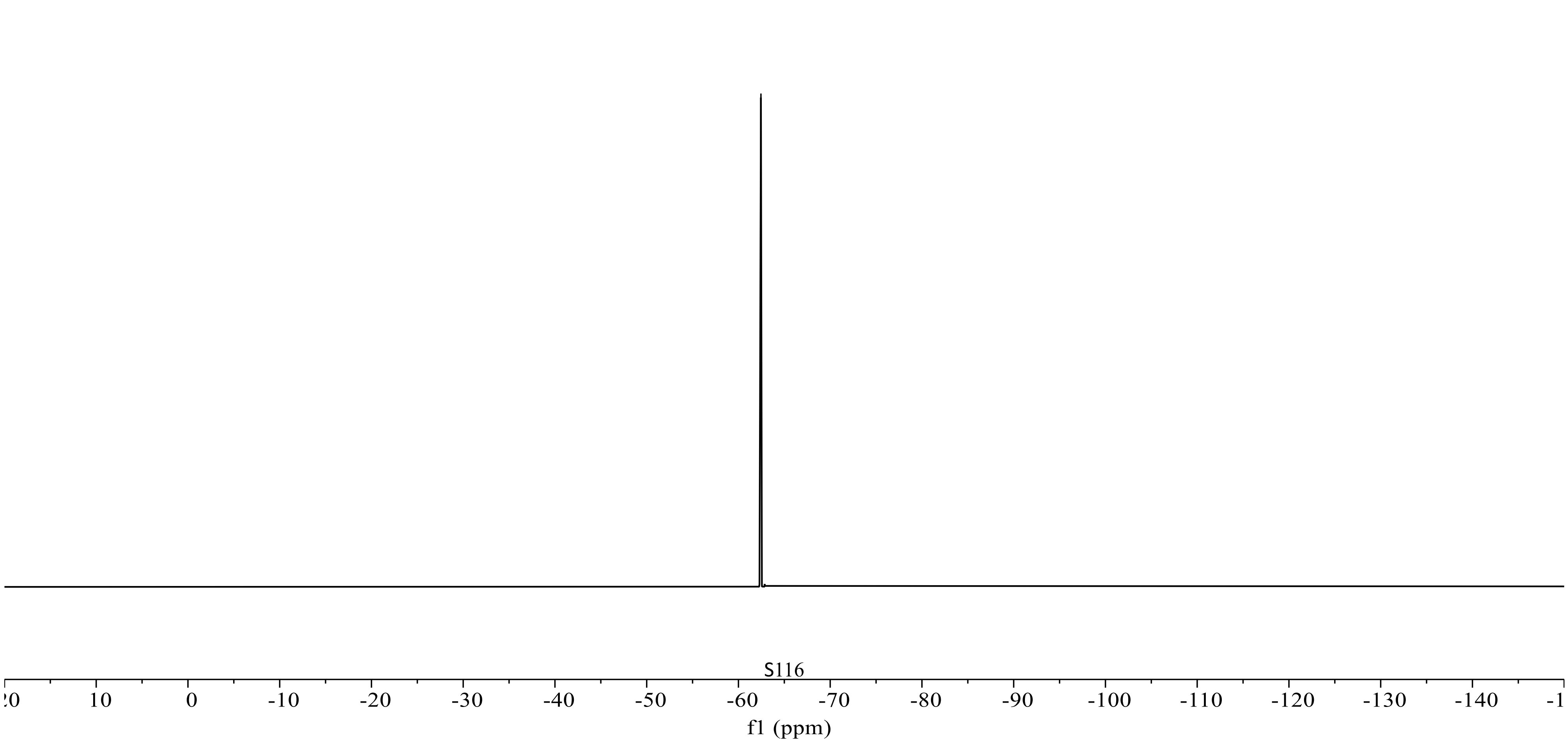

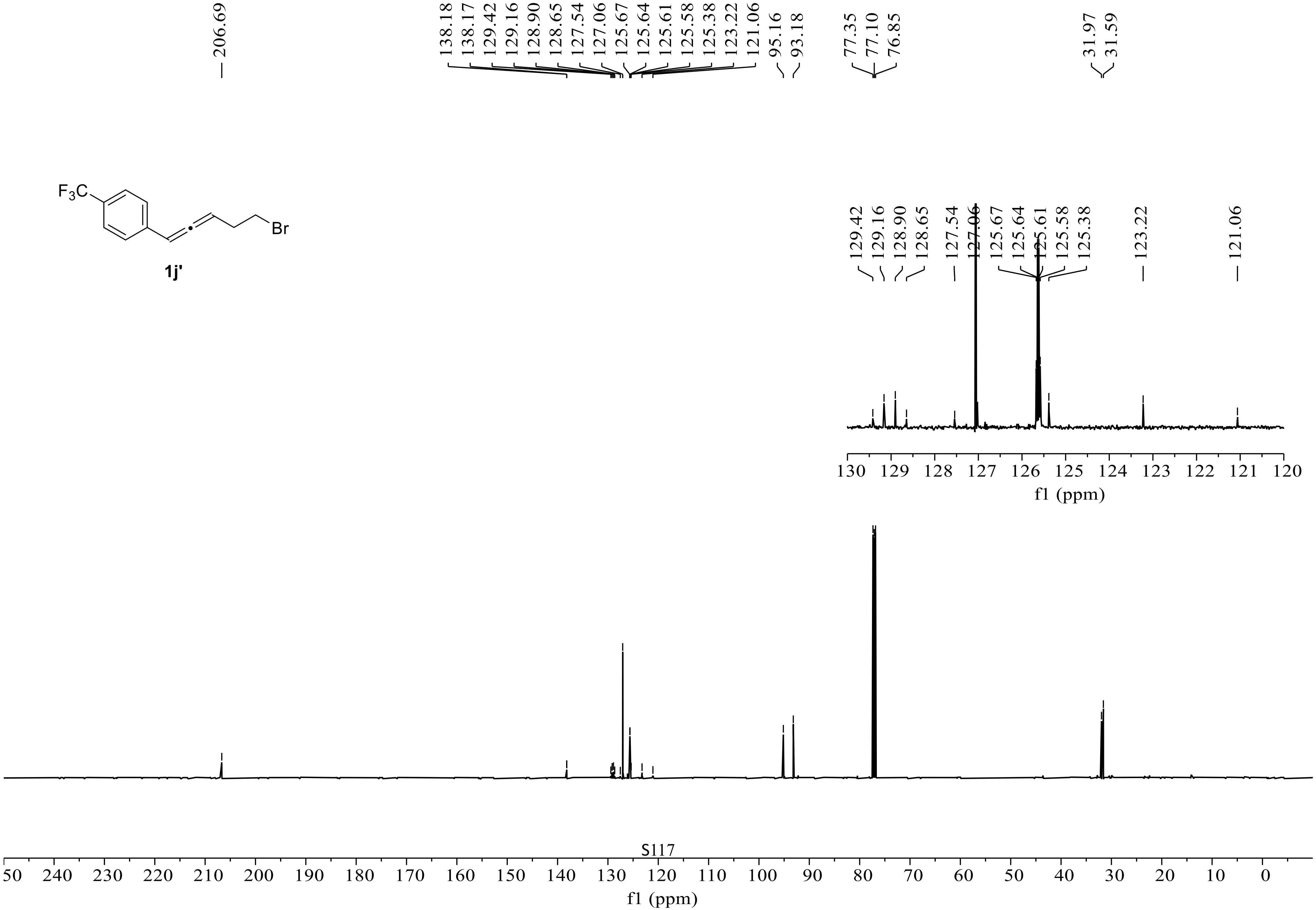

F3C
Br
206.69
138.18
138.17
129.42
129.16
128.90
128.65
127.54
127.06
125.67
125.64
125.61
125.58
125.38
123.22
121.06
95.16
93.18
77.35
77.10
76.85
31.97
31.59
130 129 128 127 126 125 124 123 122 121 120
f1 (ppm)
50 240 230 220 210 200 190 180 170 160 150 140 130 120 110 100 90 80 70 60 50 40 30 20 10 0
f1 (ppm)

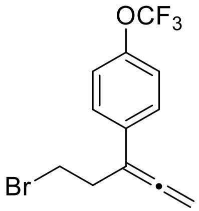
OCF3
Br

1k

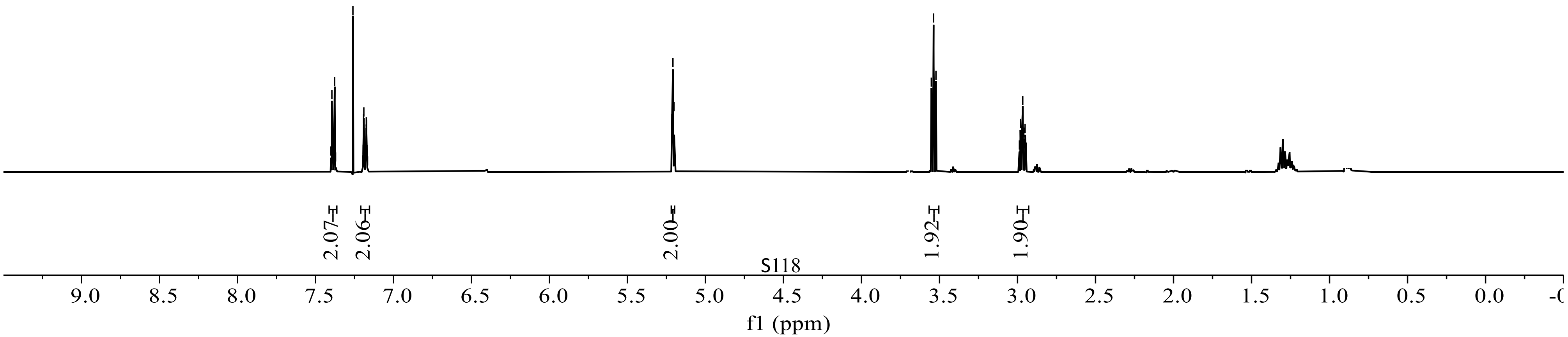
7.40 7.39 7.39 7.39 7.38 7.38 7.38 7.37 7.26 7.20 7.19 7.19 7.19 7.19 7.19 7.18 7.18 7.18 7.18 7.17 7.17 7.17 7.17 7.17 5.22 5.21 5.20
3.55 3.54 3.52 2.99 2.98 2.97 2.97 2.97 2.96 2.96 2.95 2.95
2.07
2.06
2.00
1.92
1.90
9.0 8.5 8.0 7.5 7.0 6.5 6.0 5.5 5.0 4.5 4.0 3.5 3.0 2.5 2.0 1.5 1.0 0.5 0.0
f1 (ppm)

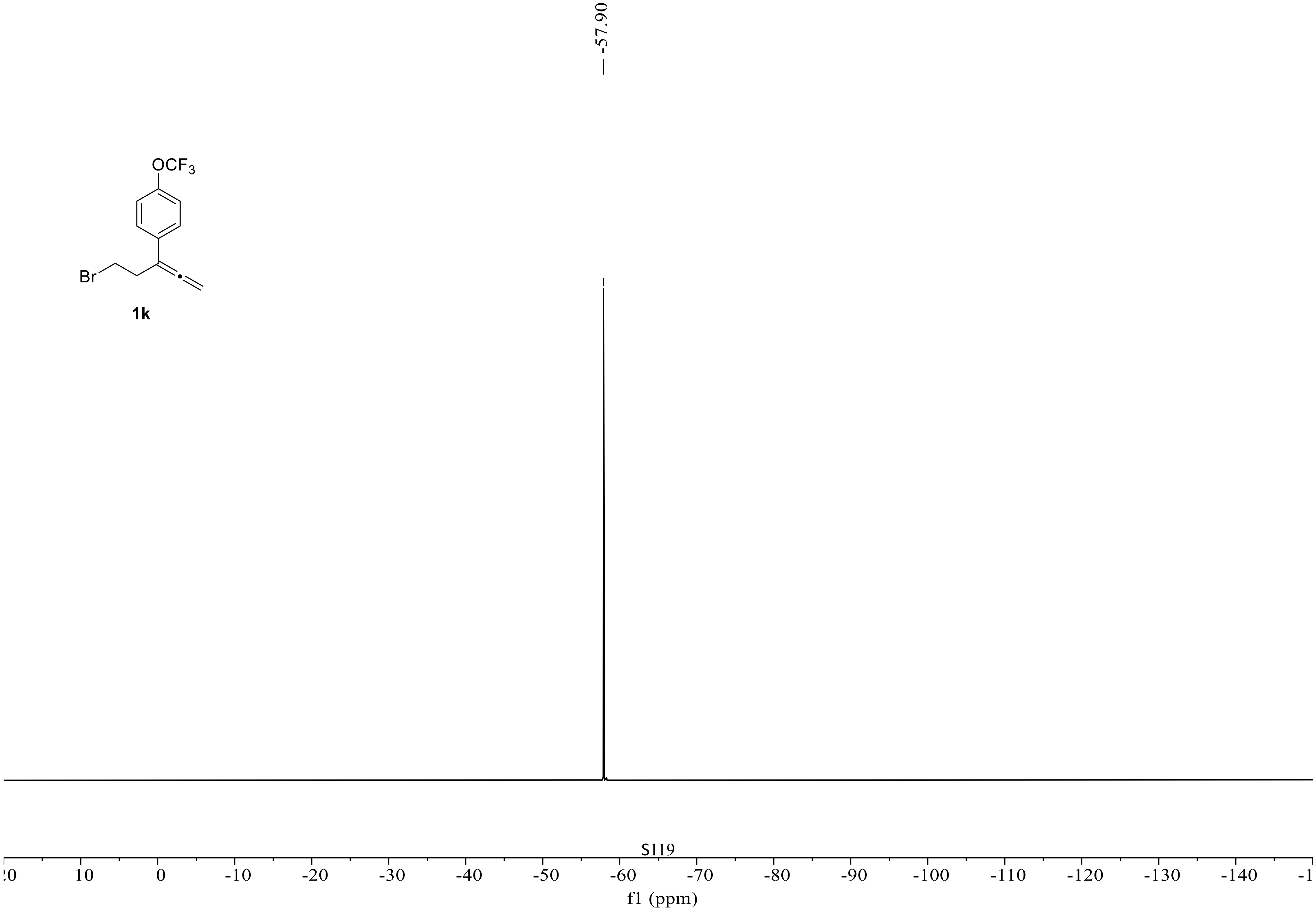
-57.90
OCF3
Br
1k
20
10
0
-10
-20
-30
-40
-50
-60
-70
-80
-90
-100
-110
-120
-130
-140
f1 (ppm)

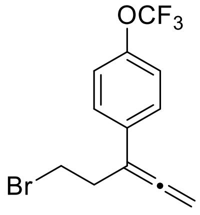
OCF3
Br

1k

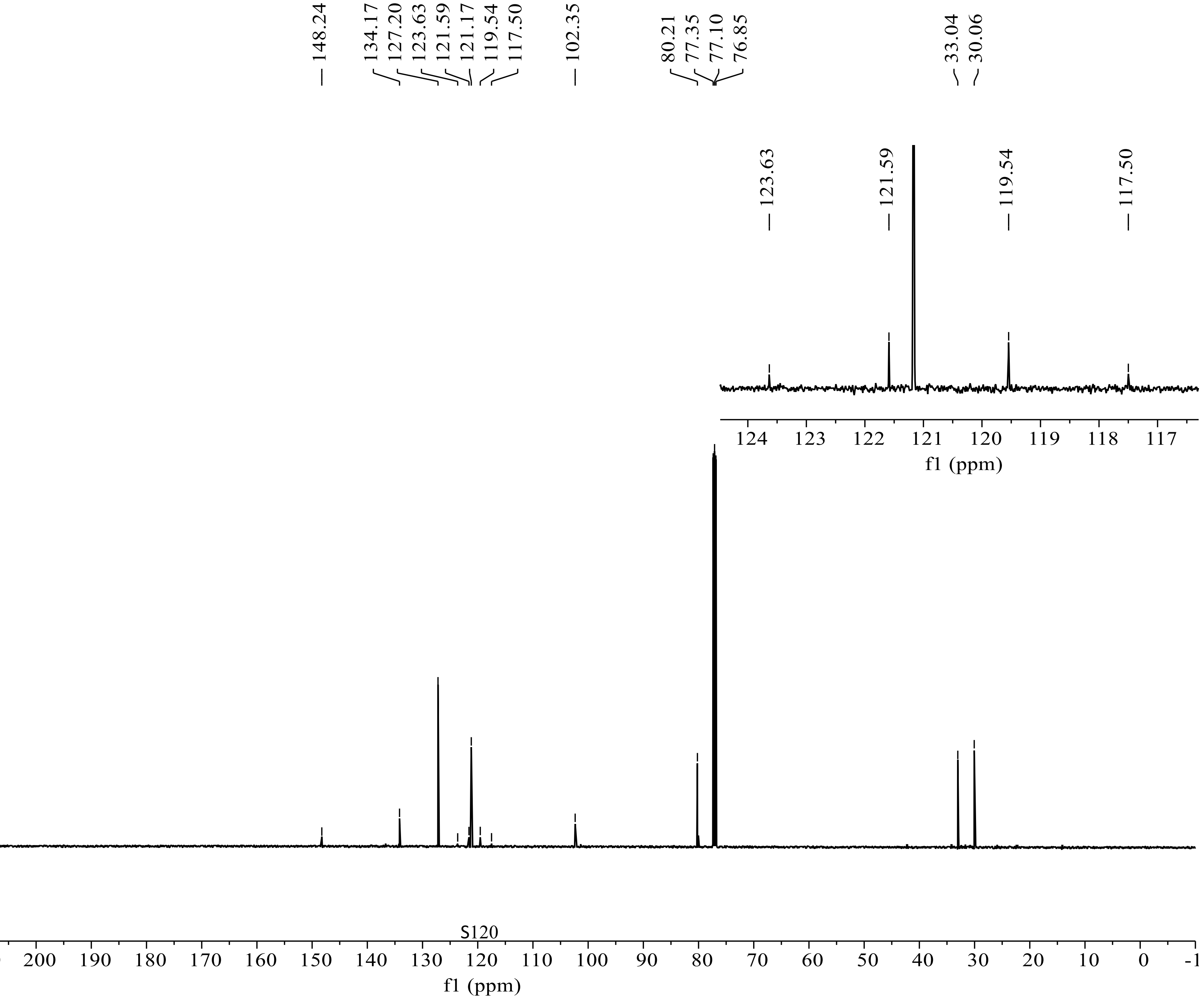
208.51
148.24
134.17
127.20
123.63
121.59
121.17
119.54
117.50
102.35
80.21
77.35
77.10
76.85
33.04
30.06
123.63
121.59
119.54
117.50
124 123 122 121 120 119 118 117
f1 (ppm)
50 240 230 220 210 200 190 180 170 160 150 140 130 120 110 100 90 80 70 60 50 40 30 20 10 0 -1
f1 (ppm)

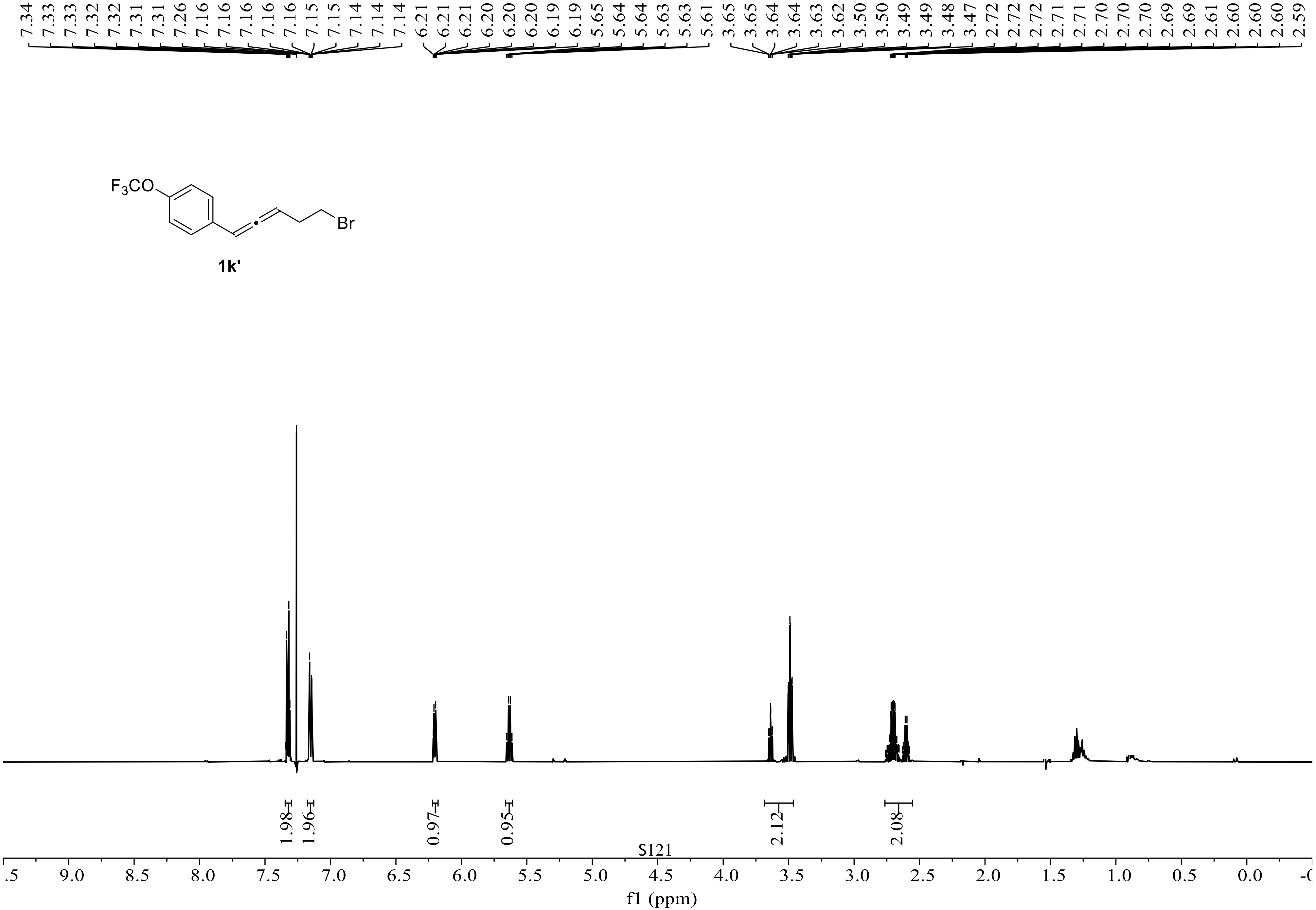
7.34 7.33 7.33 7.32 7.32 7.31 7.31 7.26 7.16 7.16 7.16 7.16 7.16 7.15 7.15 7.14 7.14 7.14 6.21 6.21 6.21 6.20 6.20 6.20 6.19 6.19 5.65 5.64 5.64 5.63 5.63 5.61 3.65 3.65 3.64 3.64 3.63 3.62 3.50 3.50 3.49 3.49 3.48 3.47 2.72 2.72 2.72 2.71 2.71 2.70 2.70 2.70 2.69 2.69 2.61 2.60 2.60 2.60 2.59
F3CO
Br
1k'
1.98
1.96
0.97
0.95
2.12
2.08
.5 9.0 8.5 8.0 7.5 7.0 6.5 6.0 5.5 5.0 4.5 4.0 3.5 3.0 2.5 2.0 1.5 1.0 0.5 0.0
f1 (ppm)

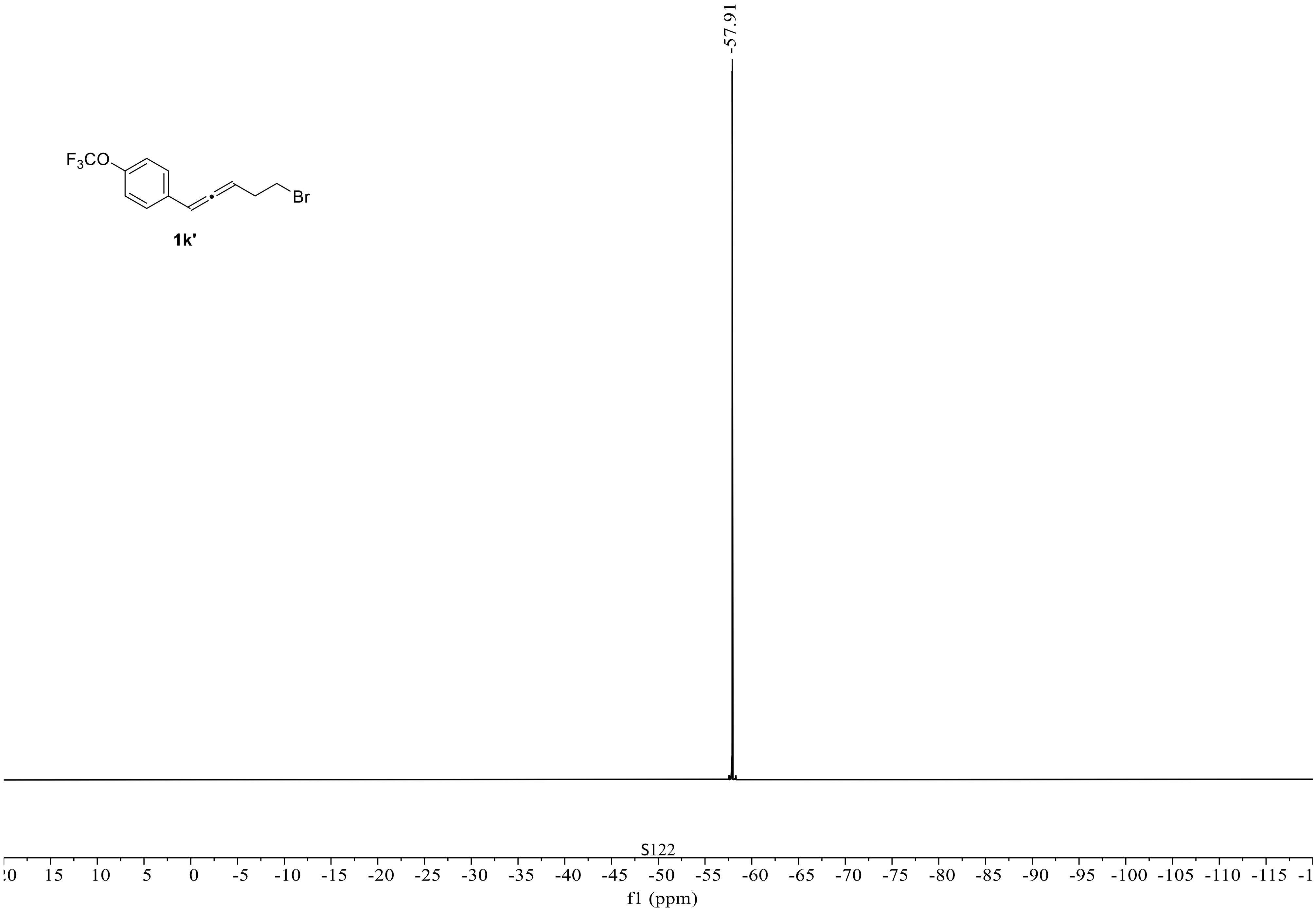
-57.91
F3CO
Br
1k'
15 10 5 0 -5 -10 -15 -20 -25 -30 -35 -40 -45 -50 -55 -60 -65 -70 -75 -80 -85 -90 -95 -100 -105 -110 -115
f1 (ppm)

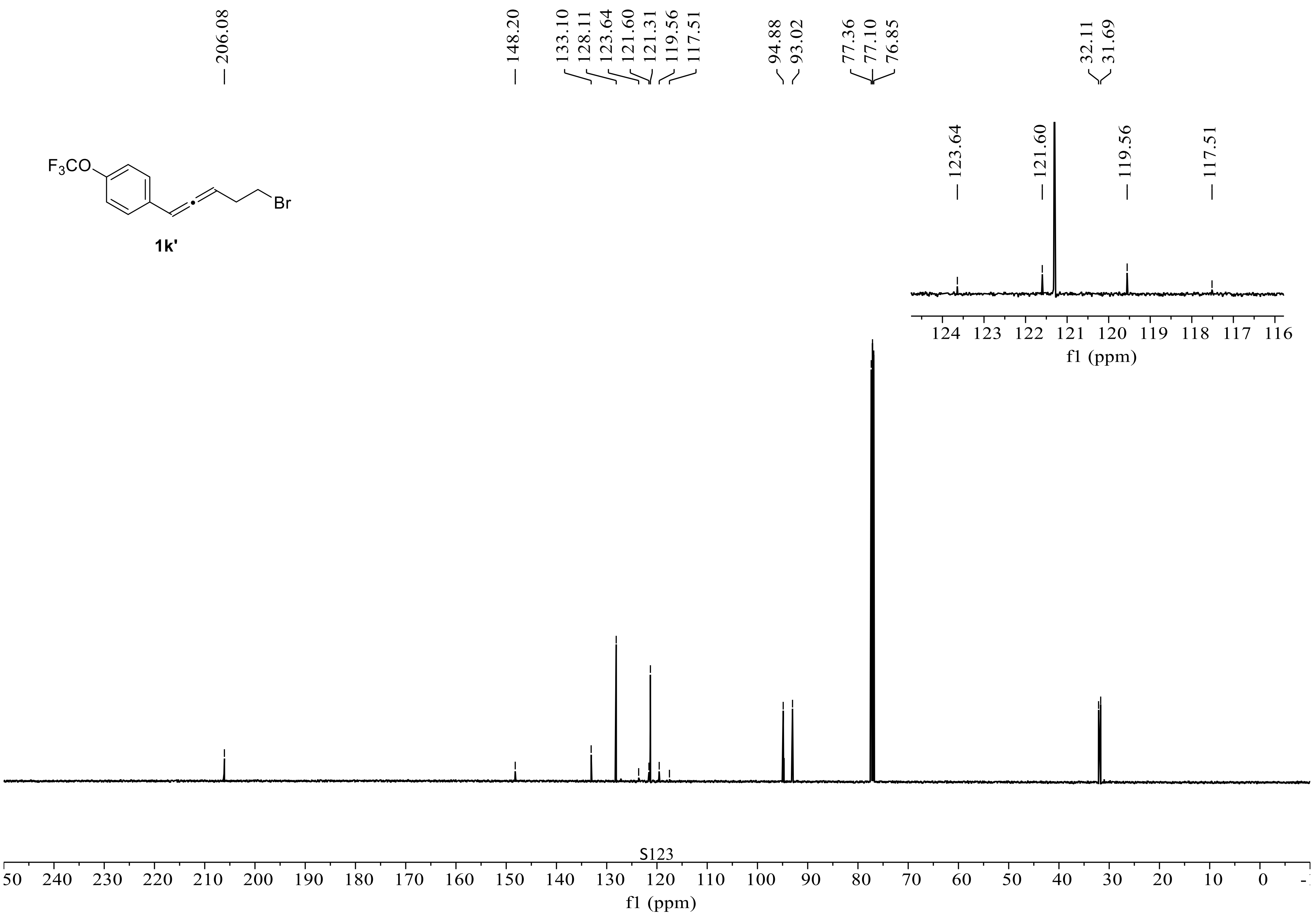
206.08
148.20
133.10
128.11
123.64
121.60
121.31
119.56
117.51
94.88
93.02
77.36
77.10
76.85
32.11
31.69
F3CO
Br
1k'
124 123 122 121 120 119 118 117 116
f1 (ppm)
50 240 230 220 210 200 190 180 170 160 150 140 130 120 110 100 90 80 70 60 50 40 30 20 10 0
f1 (ppm)

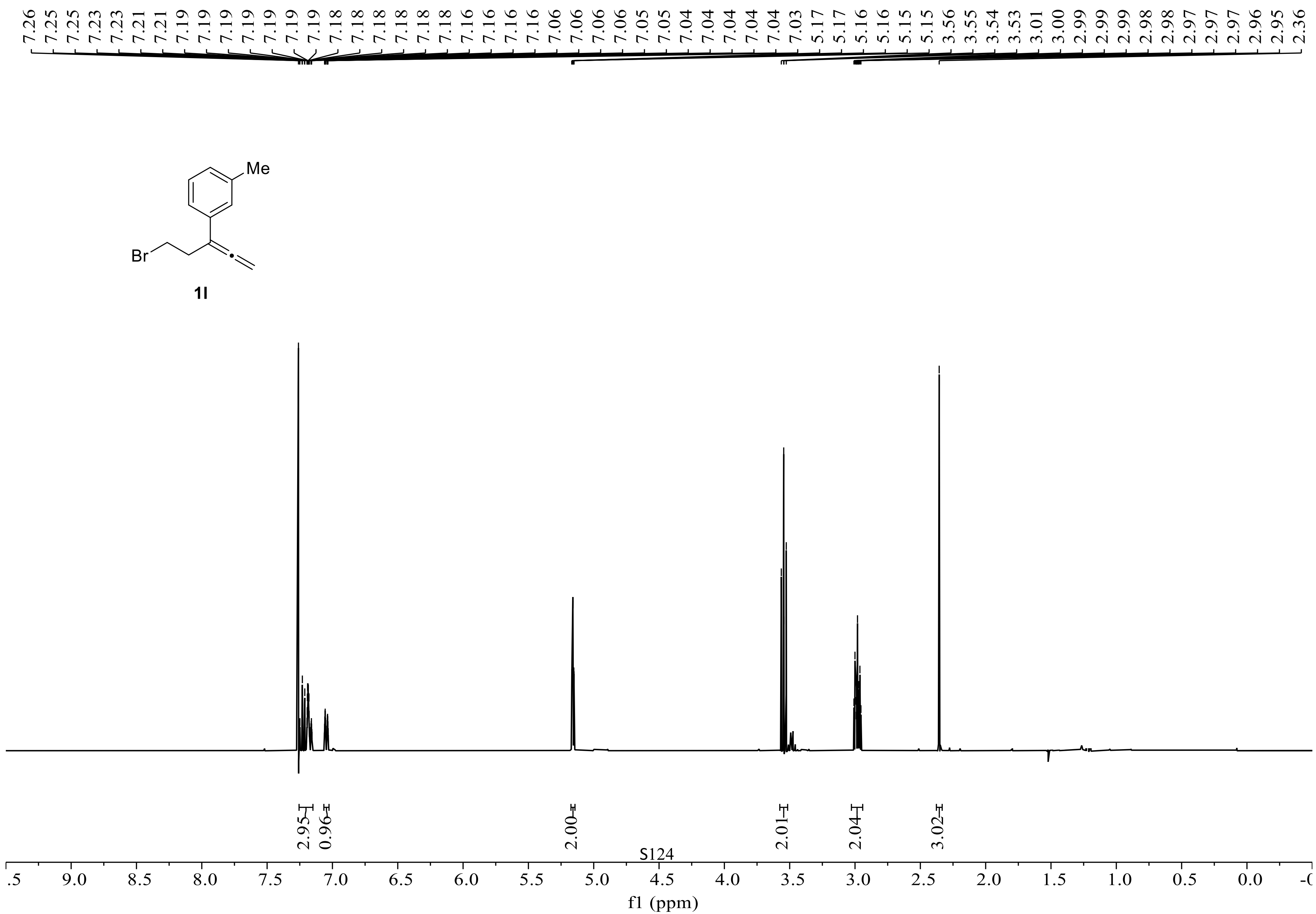
7.26 7.25 7.25 7.23 7.23 7.21 7.21 7.19 7.19 7.19 7.19 7.19 7.19 7.18 7.18 7.18 7.18 7.18 7.16 7.16 7.16 7.16 7.06 7.06 7.06 7.05 7.05 7.04 7.04 7.04 7.04 7.03 5.17 5.17 5.16 5.16 5.15 5.15 3.56 3.55 3.54 3.53 3.01 3.00 2.99 2.99 2.99 2.98 2.98 2.97 2.97 2.97 2.96 2.95 2.36
Me
Br
1l
2.95
0.96
2.00
2.01
2.04
3.02
f1 (ppm)

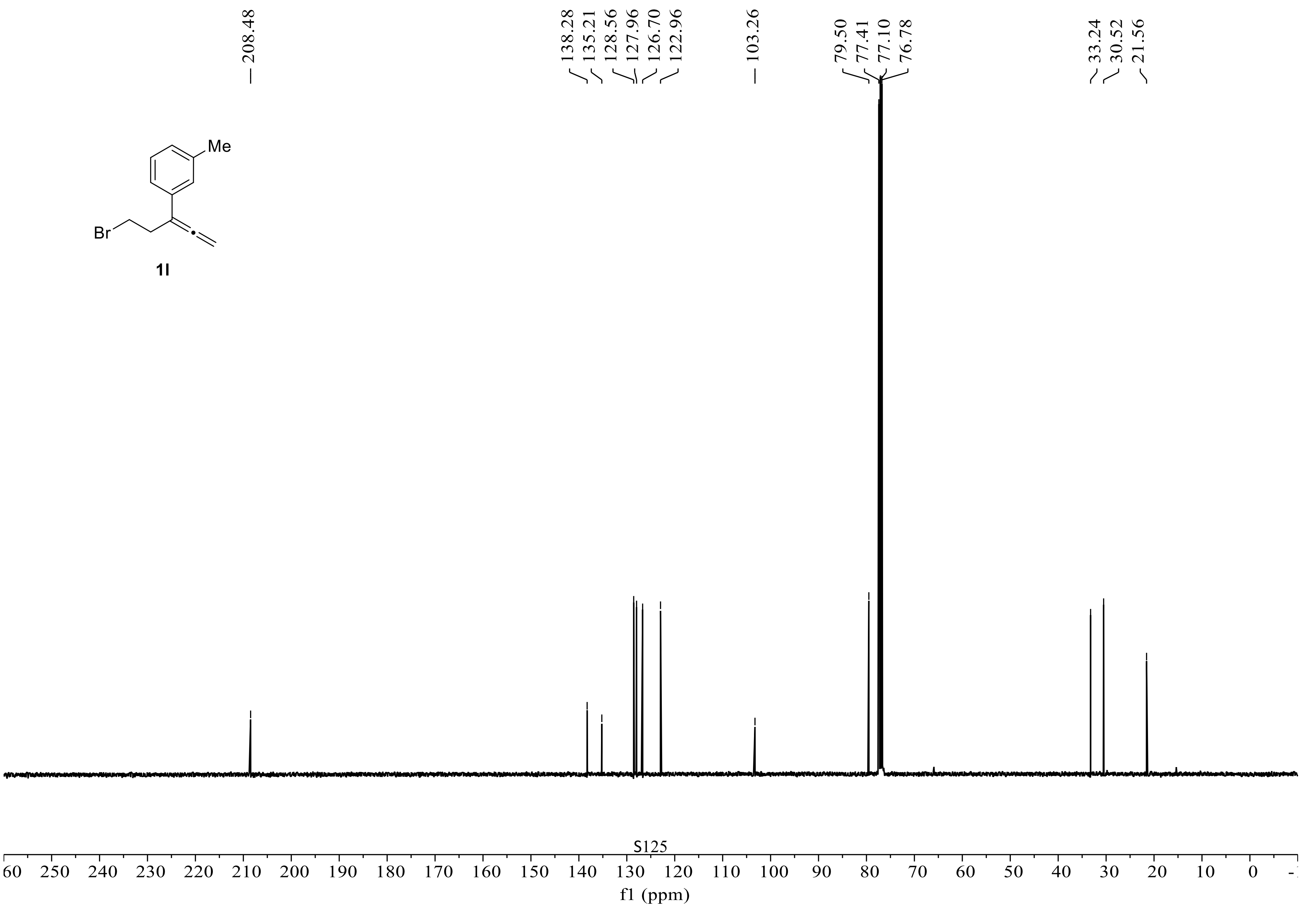
208.48
138.28
135.21
128.56
127.96
126.70
122.96
103.26
79.50
77.41
77.10
76.78
33.24
30.52
21.56
Me
Br
1l
f1 (ppm)

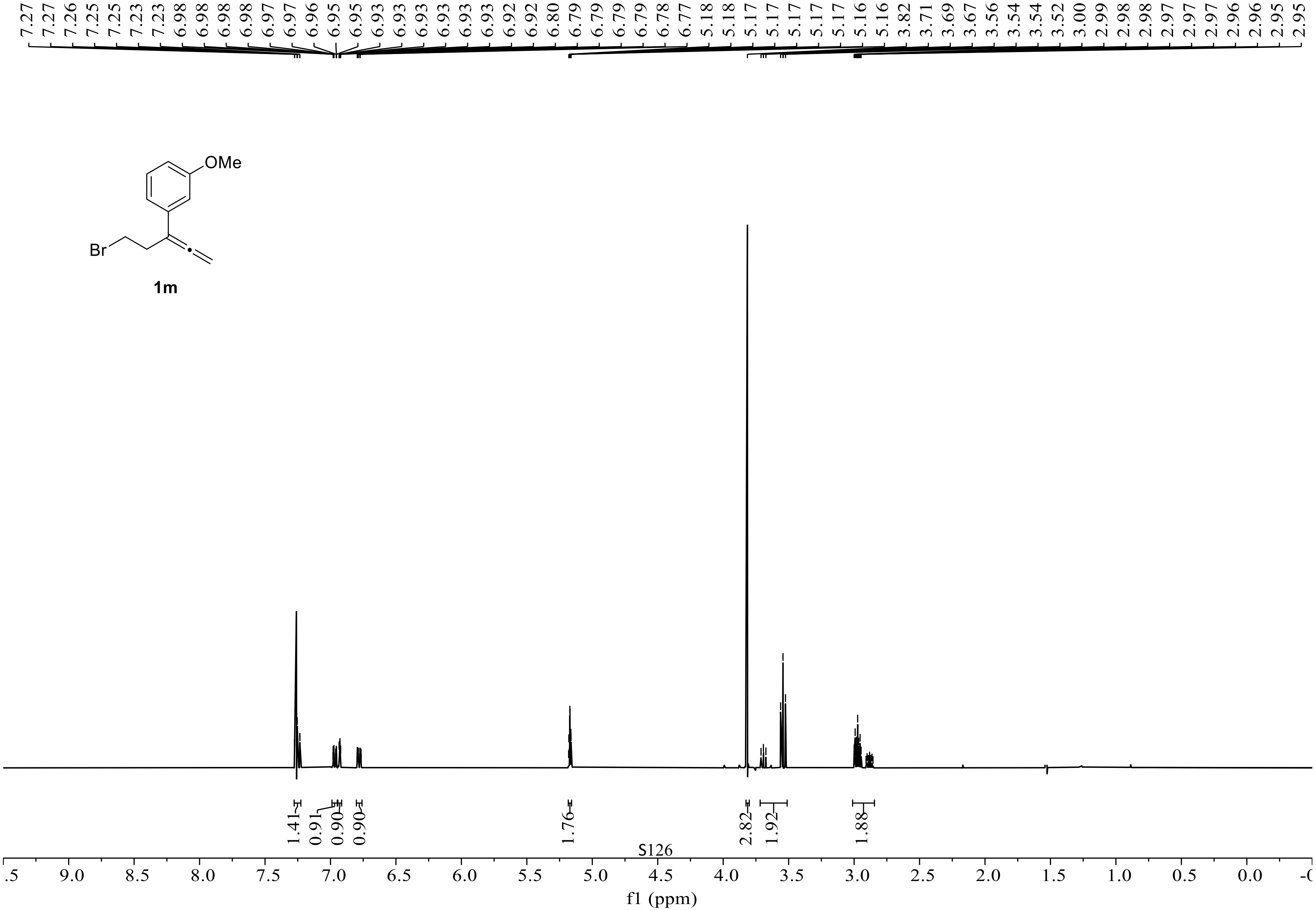
7.27 7.27 7.26 7.25 7.25 7.23 7.23 6.98 6.98 6.98 6.98 6.97 6.97 6.96 6.95 6.95 6.93 6.93 6.93 6.93 6.93 6.93 6.92 6.92 6.80 6.79 6.79 6.79 6.79 6.78 6.77 5.18 5.18 5.17 5.17 5.17 5.17 5.17 5.17 5.16 5.16 3.82 3.71 3.69 3.67 3.56 3.54 3.54 3.52 3.00 2.99 2.98 2.98 2.97 2.97 2.97 2.96 2.96 2.95 2.95
OMe
Br
1m
1.41
0.91
0.90
0.90
1.76
2.82
1.92
1.88
.5 9.0 8.5 8.0 7.5 7.0 6.5 6.0 5.5 5.0 4.5 4.0 3.5 3.0 2.5 2.0 1.5 1.0 0.5 0.0
f1 (ppm)

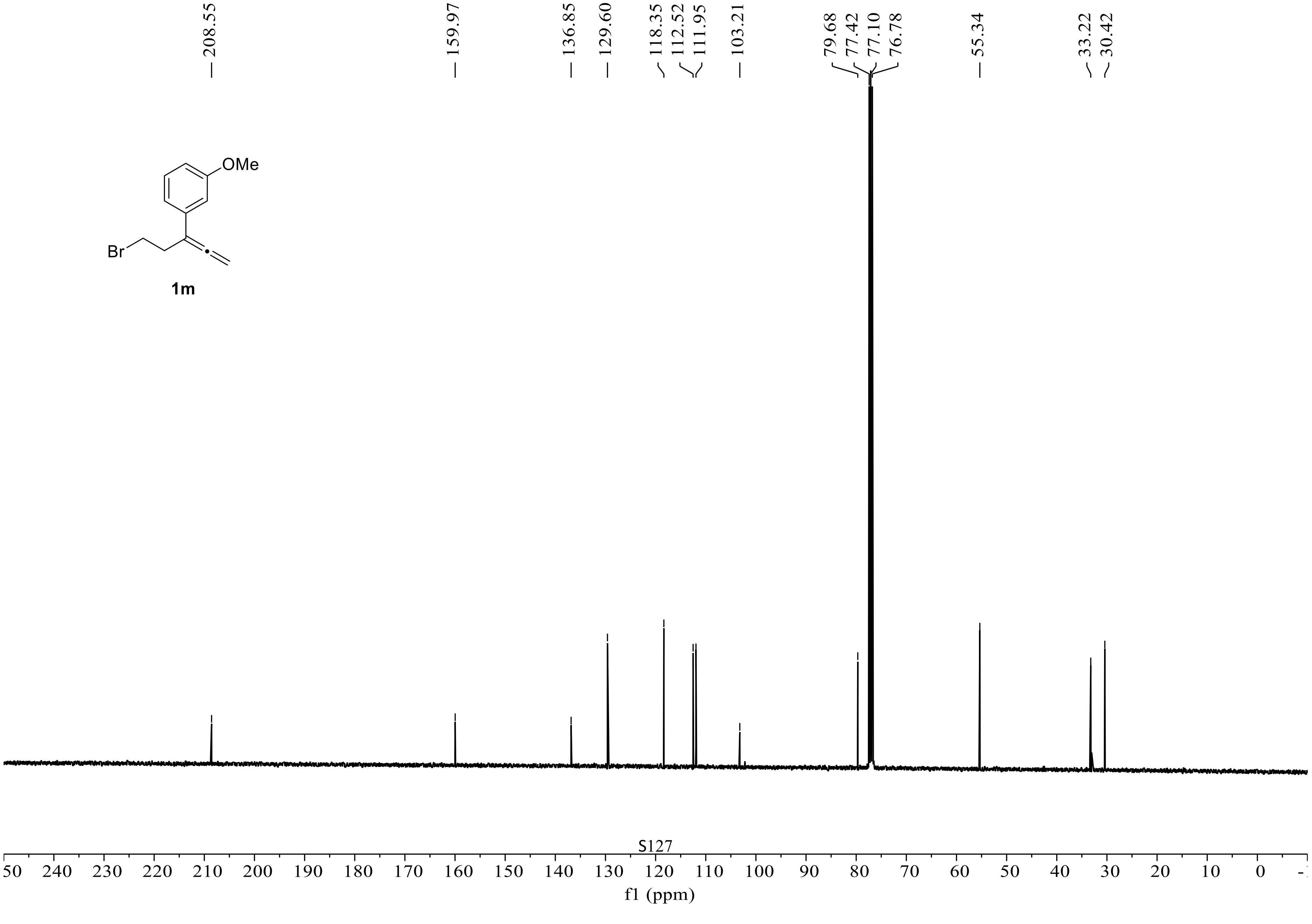
208.55
159.97
136.85
129.60
118.35
112.52
111.95
103.21
79.68
77.42
77.10
76.78
55.34
33.22
30.42
OMe
Br
1m
50 240 230 220 210 200 190 180 170 160 150 140 130 120 110 100 90 80 70 60 50 40 30 20 10 0
f1 (ppm)

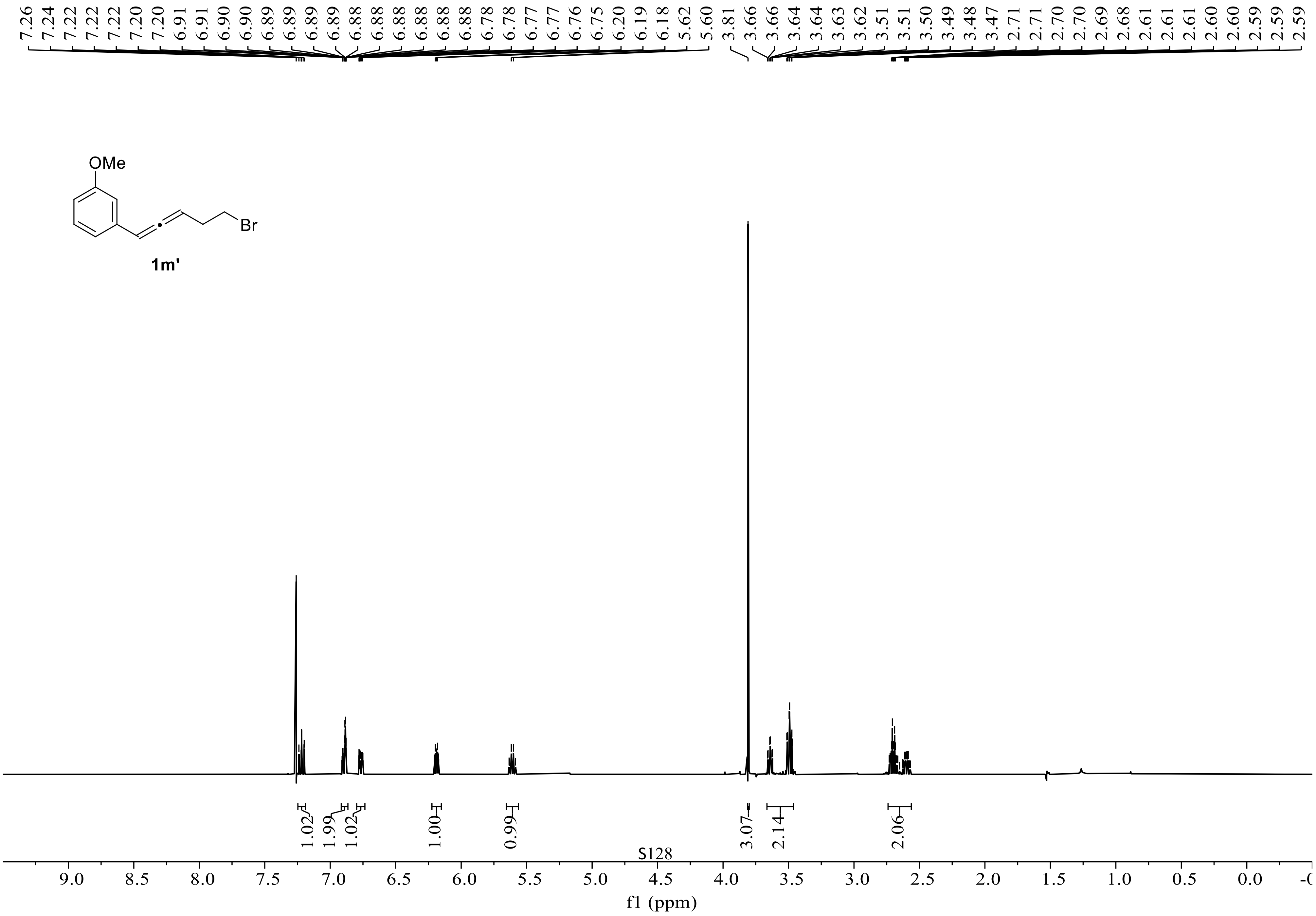
7.26 7.24 7.22 7.22 7.22 7.20 7.20 6.91 6.90 6.90 6.89 6.89 6.89 6.89 6.88 6.88 6.88 6.88 6.88 6.88 6.78 6.78 6.77 6.77 6.76 6.75 6.20 6.19 6.18 5.62 5.60 3.81 3.66 3.66 3.64 3.64 3.63 3.62 3.51 3.51 3.50 3.49 3.48 3.47 2.71 2.71 2.70 2.70 2.69 2.68 2.61 2.61 2.61 2.60 2.60 2.59 2.59 2.59
OMe
Br
1m'
1.02
1.99
1.02
1.00
0.99
3.07
2.14
2.06
9.0 8.5 8.0 7.5 7.0 6.5 6.0 5.5 5.0 4.5 4.0 3.5 3.0 2.5 2.0 1.5 1.0 0.5 0.0
f1 (ppm)

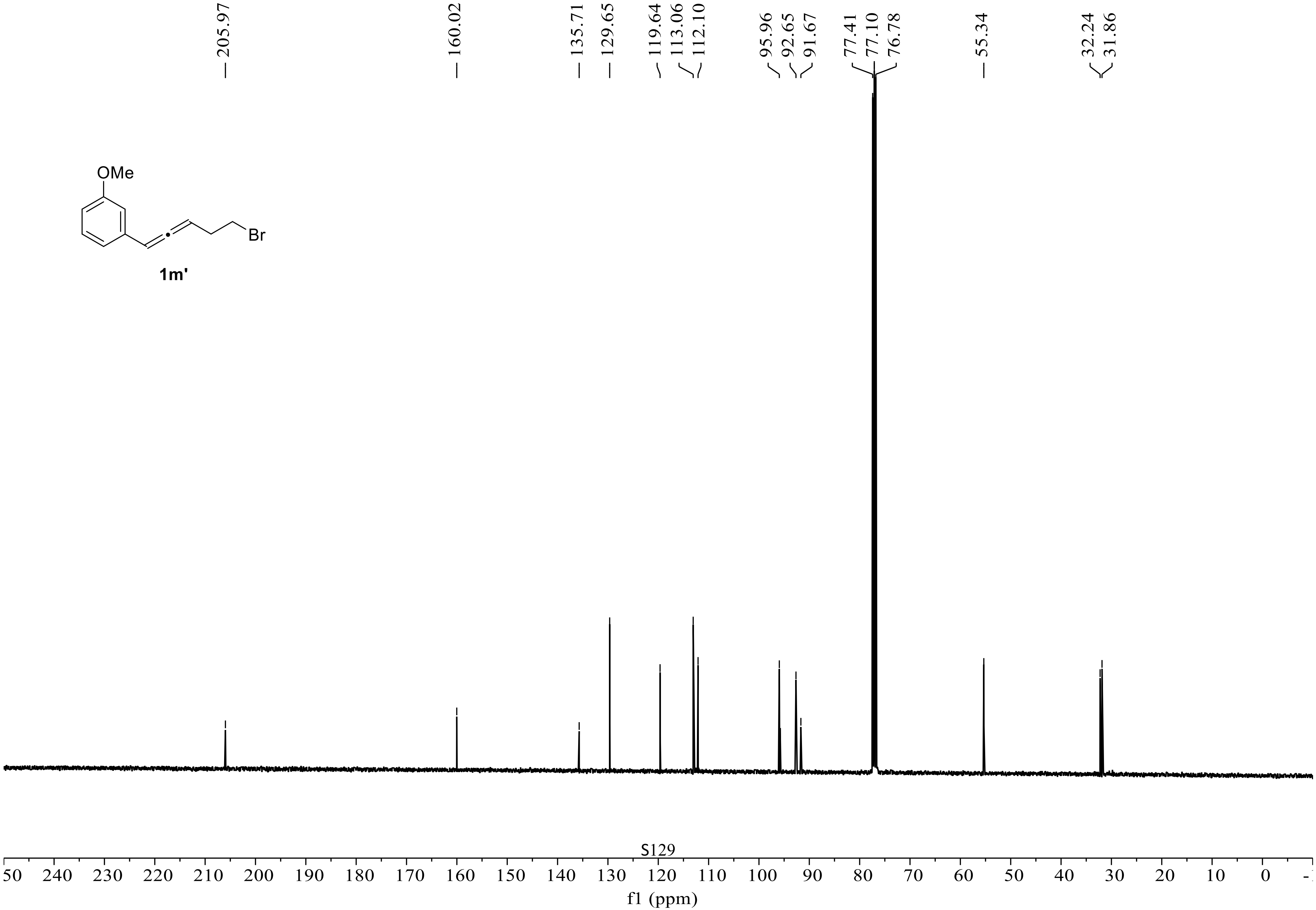

205.97
160.02
135.71
129.65
119.64
113.06
112.10
95.96
92.65
91.67
77.41
77.10
76.78
55.34
32.24
31.86
OMe
Br
50
240
230
220
210
200
190
180
170
160
150
140
130
120
110
100
90
80
70
60
50
40
30
20
10
0
f1 (ppm)

1m'

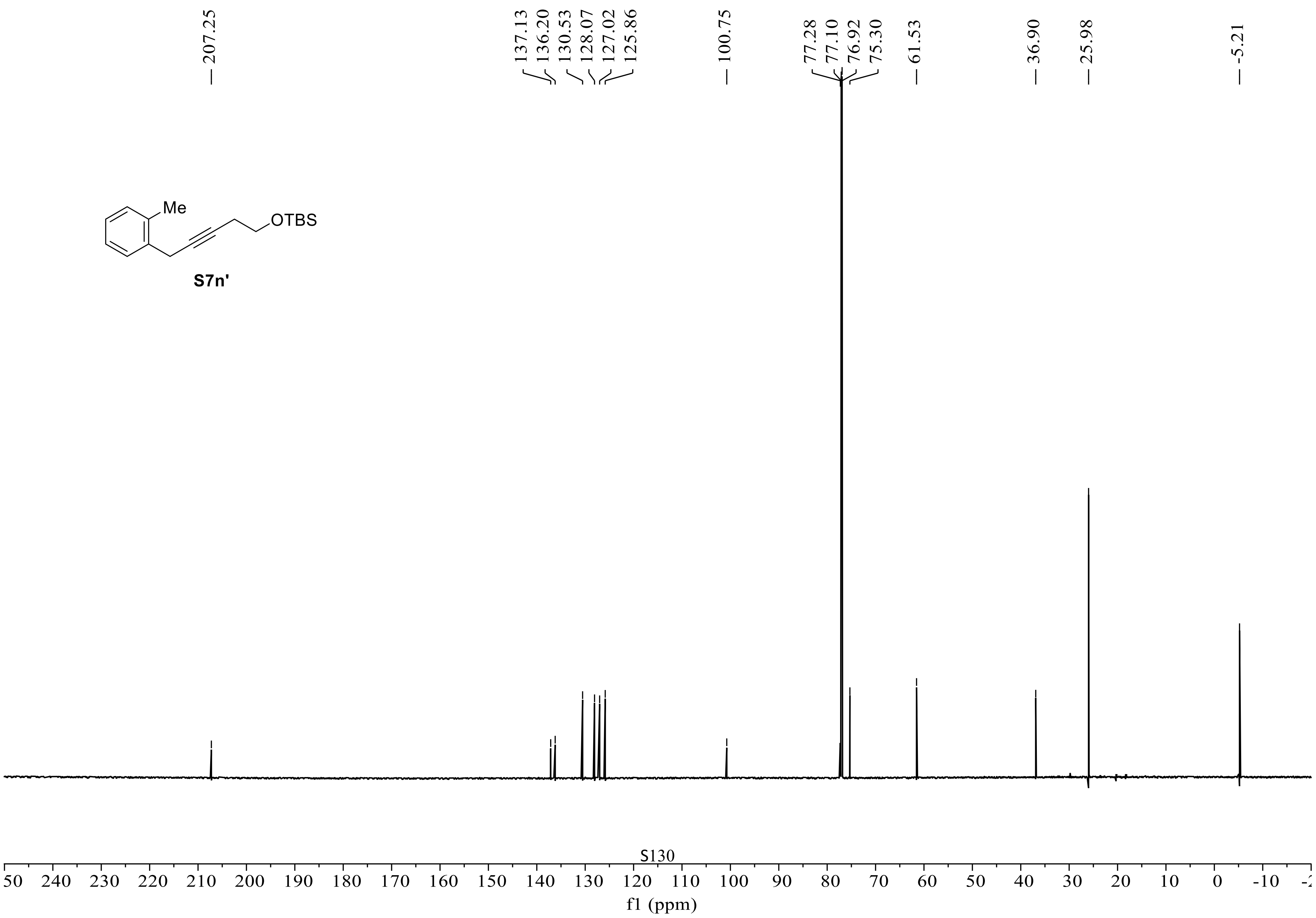
207.25
137.13
136.20
130.53
128.07
127.02
125.86
100.75
77.28
77.10
76.92
75.30
61.53
36.90
25.98
-5.21
Me
OTBS
S7n'
50 240 230 220 210 200 190 180 170 160 150 140 130 120 110 100 90 80 70 60 50 40 30 20 10 0 -10
f1 (ppm)

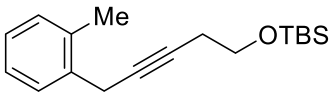
Me
OTBS

S7n'

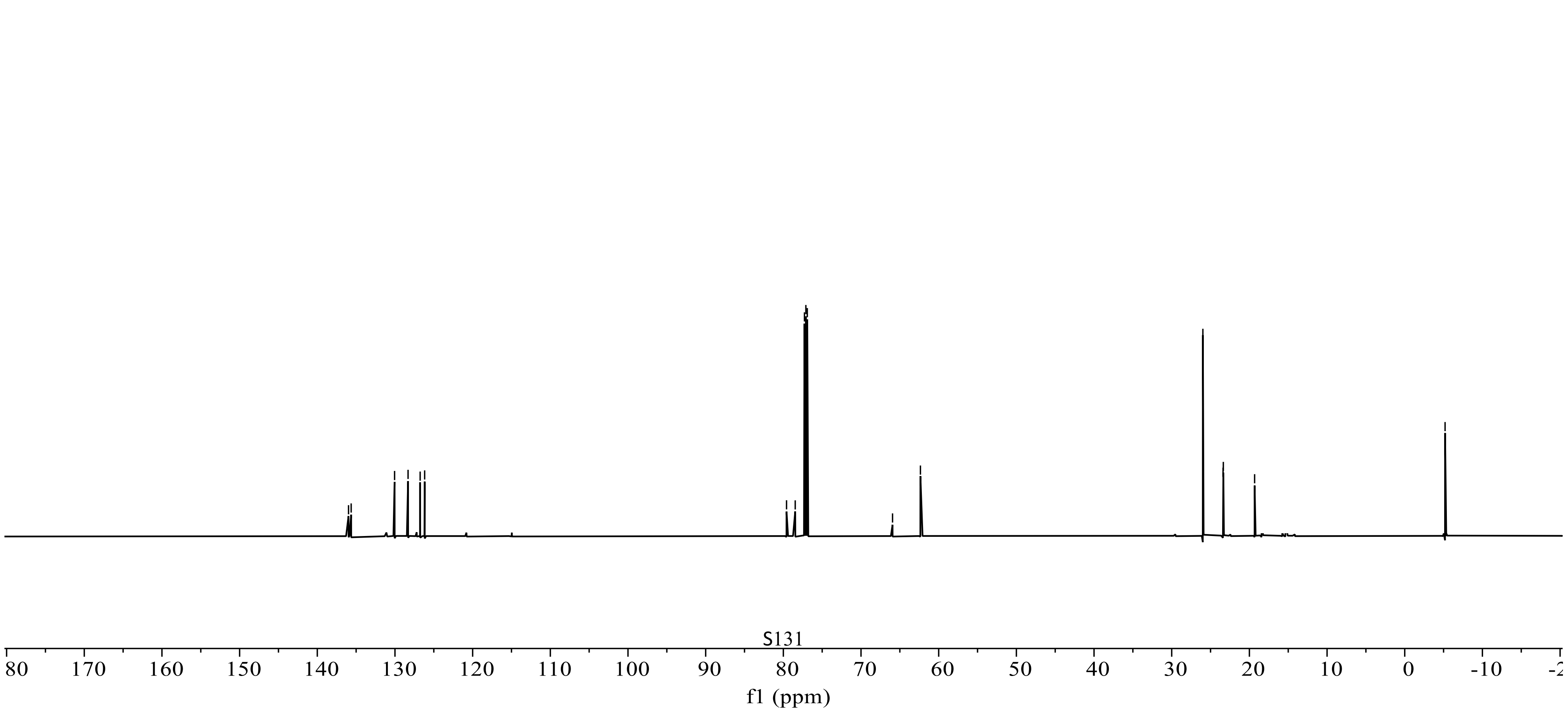
135.98
135.64
130.05
128.31
126.76
126.18
79.59
78.47
77.28
77.10
76.92
65.94
62.35
25.99
23.39
23.36
19.33
-5.19
170
160
150
140
130
120
110
100
90
80
70
60
50
40
30
20
10
0
-10
f1 (ppm)

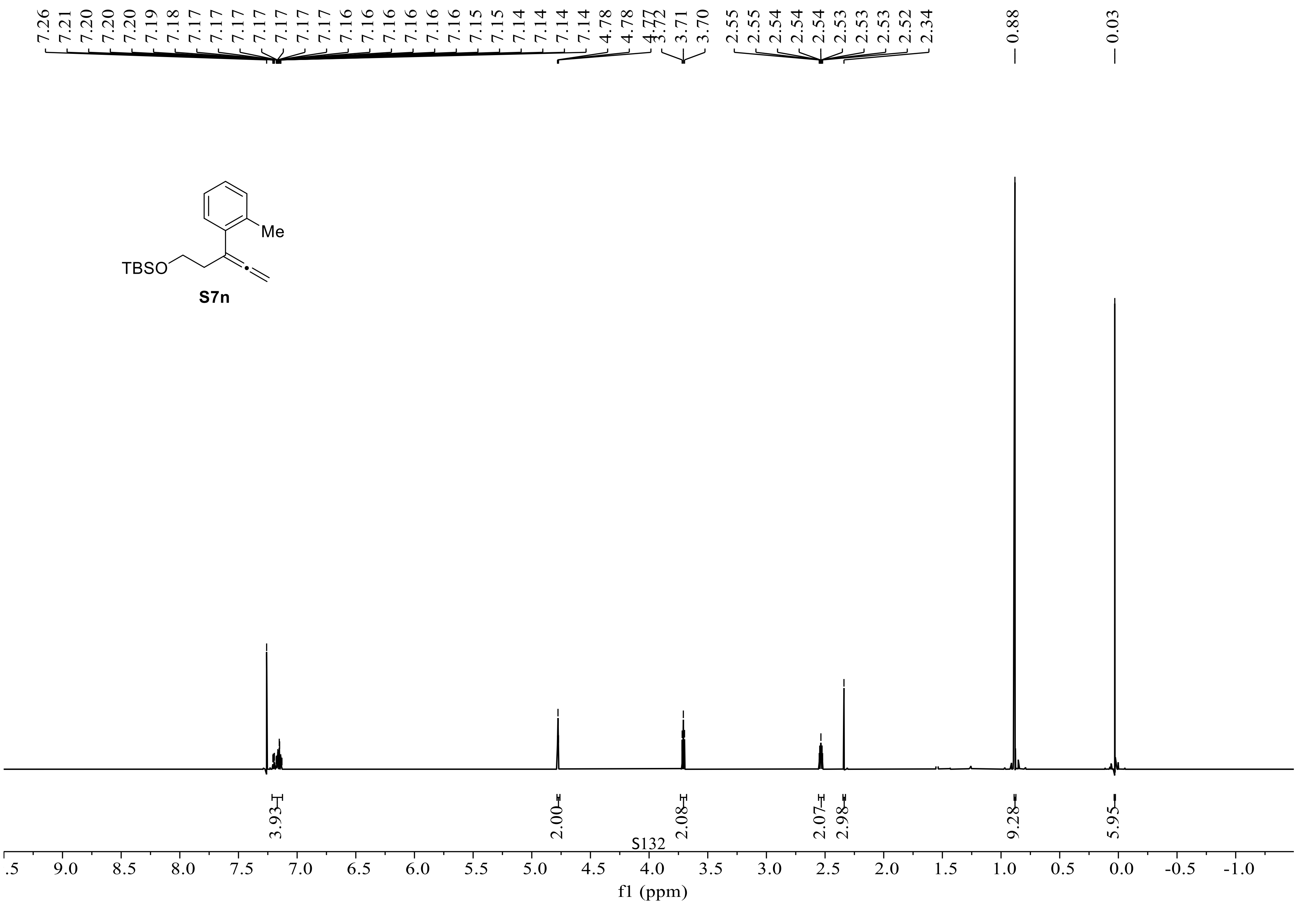
7.26 7.21 7.20 7.20 7.20 7.19 7.18 7.17 7.17 7.17 7.17 7.17 7.17 7.17 7.16 7.16 7.16 7.16 7.16 7.16 7.15 7.15 7.14 7.14 7.14 7.14 4.78 4.78 4.77 3.72 3.71 3.70 2.55 2.55 2.54 2.54 2.54 2.53 2.53 2.53 2.52 2.34 0.88 0.03
Me
TBSO
S7n
3.93 2.00 2.08 2.07 2.98 9.28 5.95
.5 9.0 8.5 8.0 7.5 7.0 6.5 6.0 5.5 5.0 4.5 4.0 3.5 3.0 2.5 2.0 1.5 1.0 0.5 0.0 -0.5 -1.0
f1 (ppm)

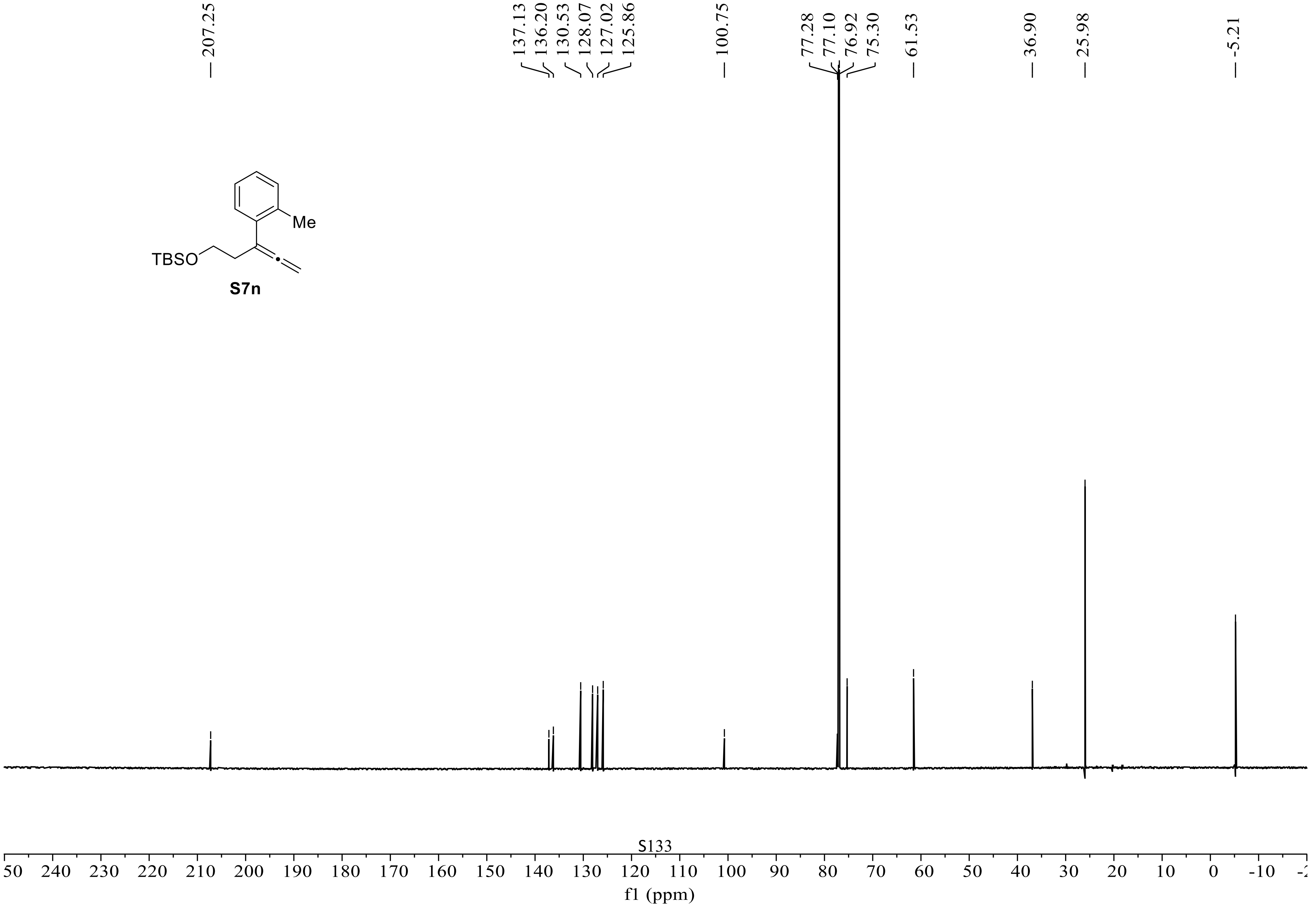
207.25
137.13
136.20
130.53
128.07
127.02
125.86
100.75
77.28
77.10
76.92
75.30
61.53
36.90
25.98
-5.21
Me
TBSO
S7n
50 240 230 220 210 200 190 180 170 160 150 140 130 120 110 100 90 80 70 60 50 40 30 20 10 0 -10
f1 (ppm)

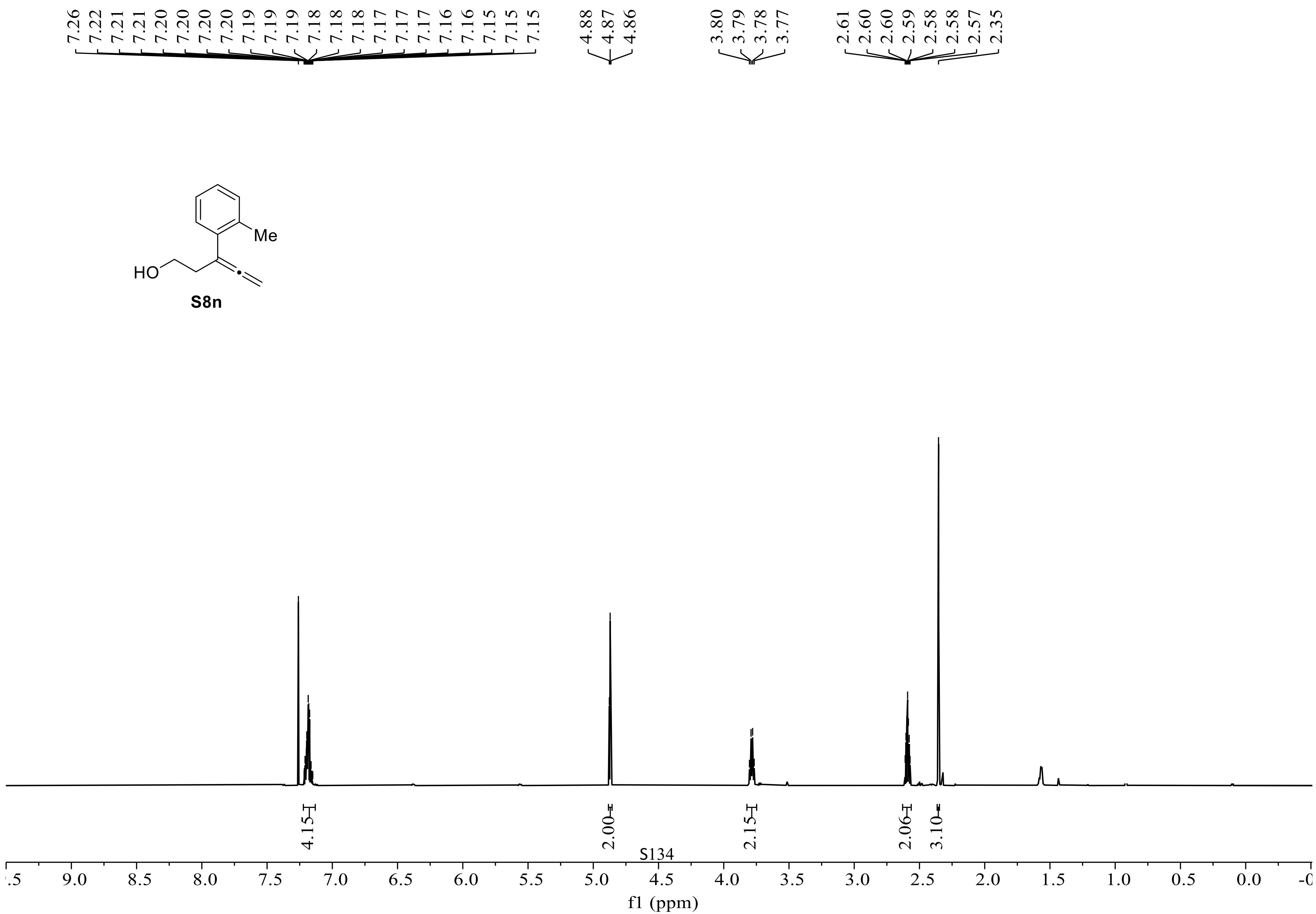
7.26
7.22
7.21
7.21
7.20
7.20
7.20
7.19
7.19
7.19
7.18
7.18
7.18
7.17
7.17
7.17
7.16
7.16
7.15
7.15
7.15
4.88
4.87
4.86
3.80
3.79
3.78
3.77
2.61
2.60
2.60
2.59
2.58
2.58
2.57
2.35
Me
HO
S8n
4.15
2.00
2.15
2.06
3.10
9.0
8.5
8.0
7.5
7.0
6.5
6.0
5.5
5.0
4.5
4.0
3.5
3.0
2.5
2.0
1.5
1.0
0.5
0.0
f1 (ppm)

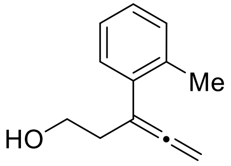
Me
HO

S8n

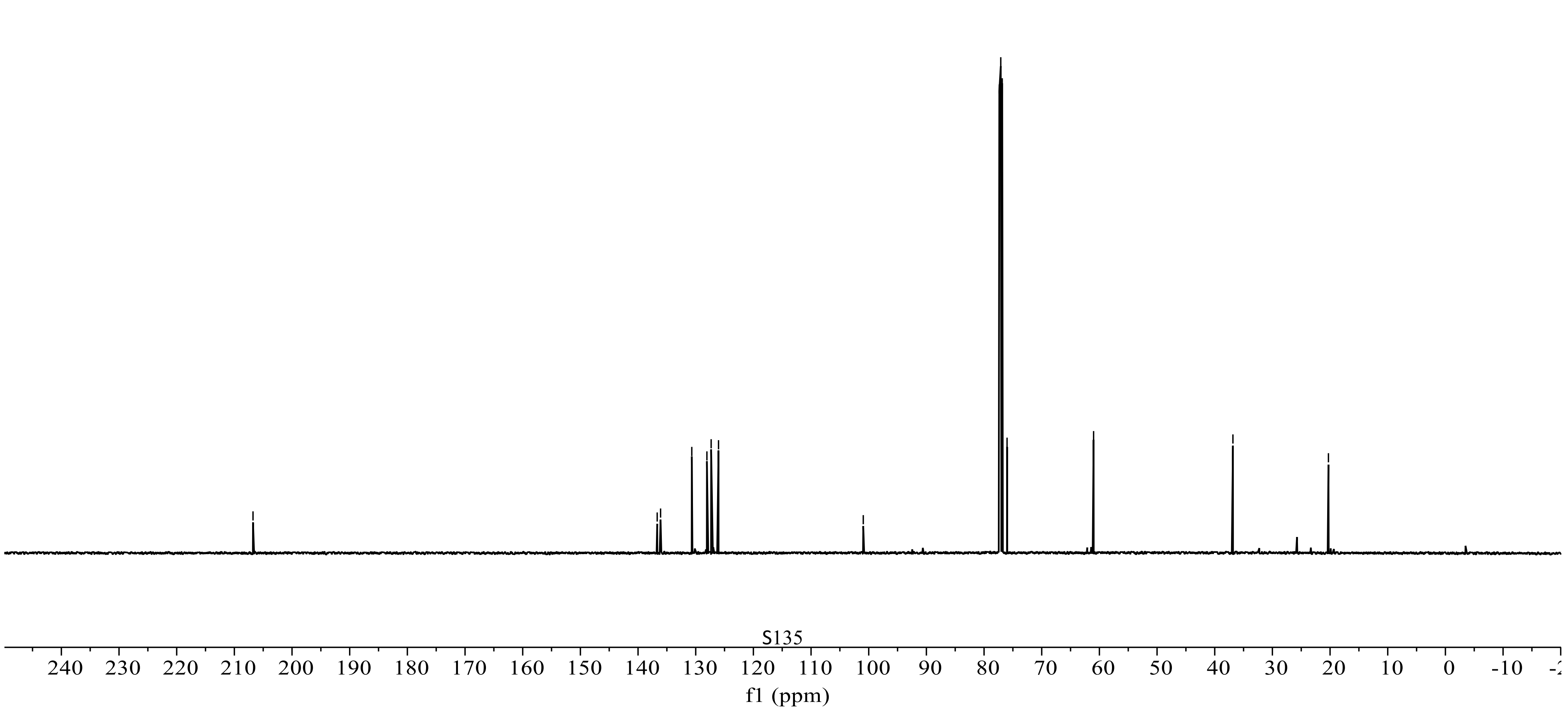
206.77
136.68
136.09
130.68
128.06
127.32
126.05
100.94
77.36
77.10
76.85
76.01
61.01
36.85
20.28
240 230 220 210 200 190 180 170 160 150 140 130 120 110 100 90 80 70 60 50 40 30 20 10 0 -10
f1 (ppm)

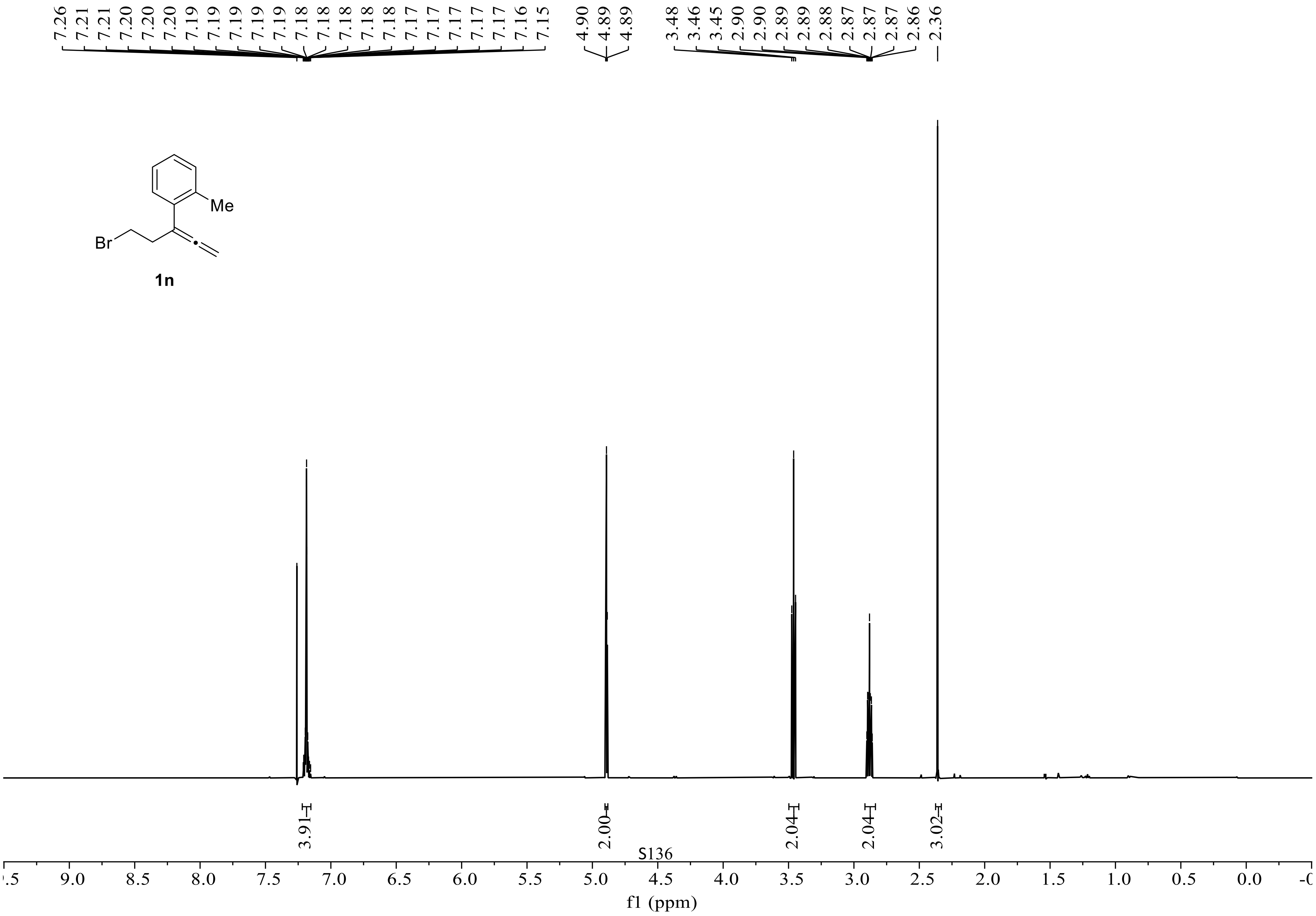
7.26 7.21 7.21 7.20 7.20 7.20 7.19 7.19 7.19 7.19 7.19 7.18 7.18 7.18 7.18 7.18 7.17 7.17 7.17 7.17 7.17 7.16 7.15
4.90 4.89 4.89
3.48 3.46 3.45 2.90 2.90 2.89 2.89 2.88 2.87 2.87 2.87 2.86
2.36
Me
Br
1n
3.91
2.00
2.04
2.04
3.02
9.0 8.5 8.0 7.5 7.0 6.5 6.0 5.5 5.0 4.5 4.0 3.5 3.0 2.5 2.0 1.5 1.0 0.5 0.0
f1 (ppm)

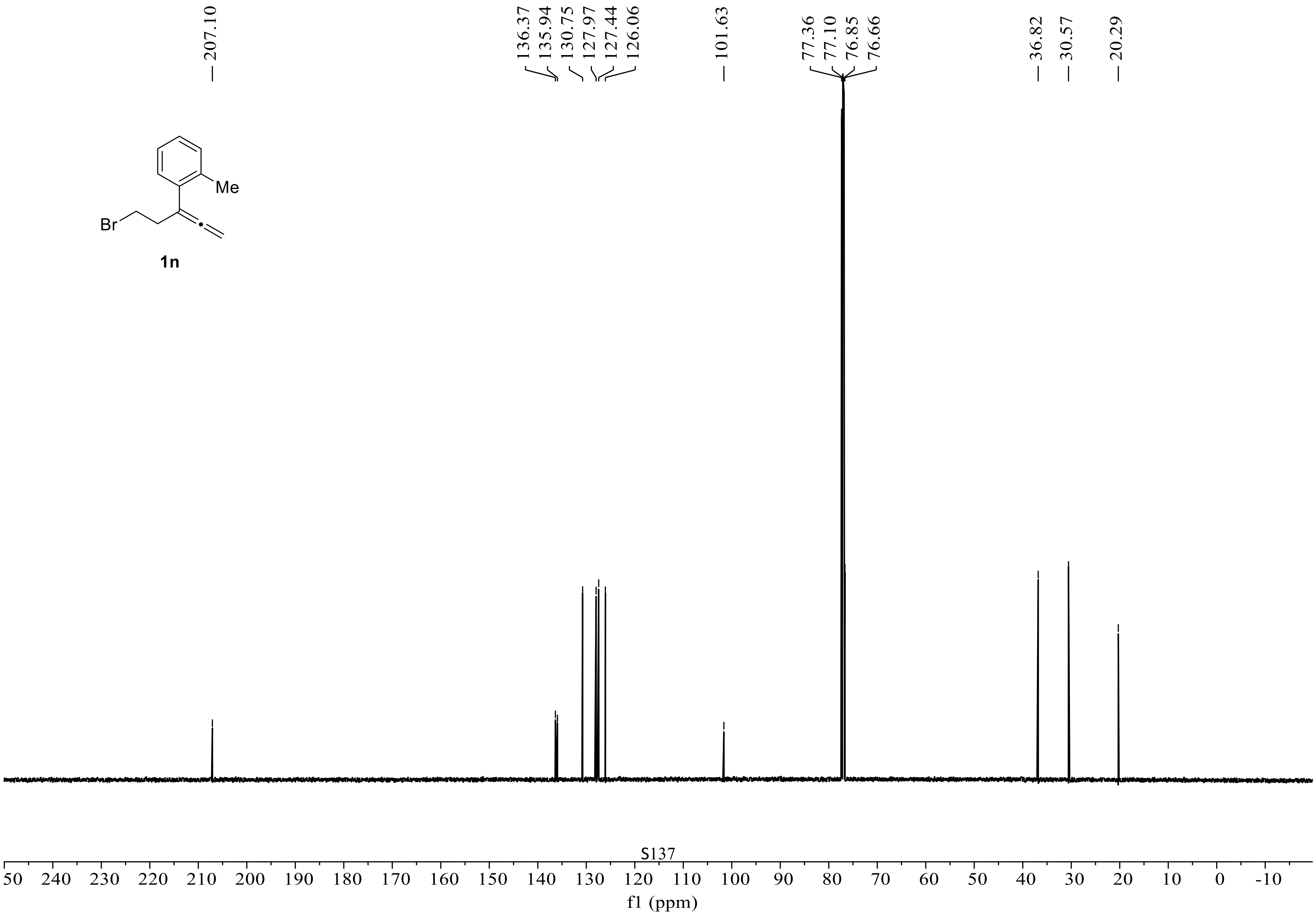
207.10
136.37
135.94
130.75
127.97
127.44
126.06
101.63
77.36
77.10
76.85
76.66
36.82
30.57
20.29
Me
Br
1n
50 240 230 220 210 200 190 180 170 160 150 140 130 120 110 100 90 80 70 60 50 40 30 20 10 0 -10
f1 (ppm)

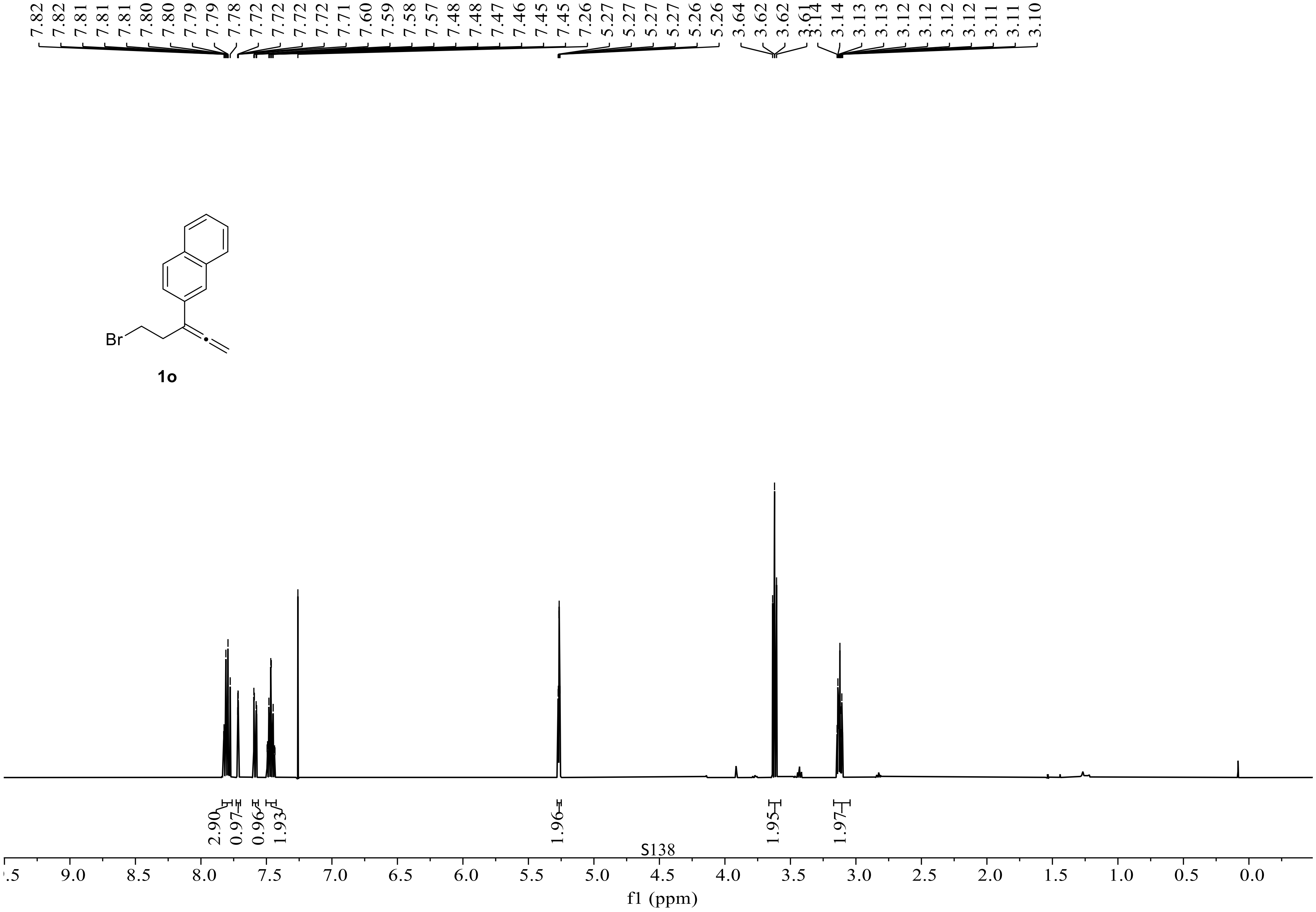

7.82 7.82 7.81 7.81 7.81 7.80 7.80 7.79 7.79 7.78 7.72 7.72 7.72 7.72 7.71 7.60 7.59 7.58 7.57 7.48 7.48 7.47 7.46 7.45 7.45 7.26 5.27 5.27 5.27 5.27 5.26 5.26 3.64 3.62 3.62 3.61 3.14 3.14 3.13 3.13 3.12 3.12 3.12 3.12 3.11 3.11 3.10
Br
1o
2.90 0.97 0.96 1.93 1.96 1.95 1.97
9.0 8.5 8.0 7.5 7.0 6.5 6.0 5.5 5.0 4.5 4.0 3.5 3.0 2.5 2.0 1.5 1.0 0.5 0.0
f1 (ppm)

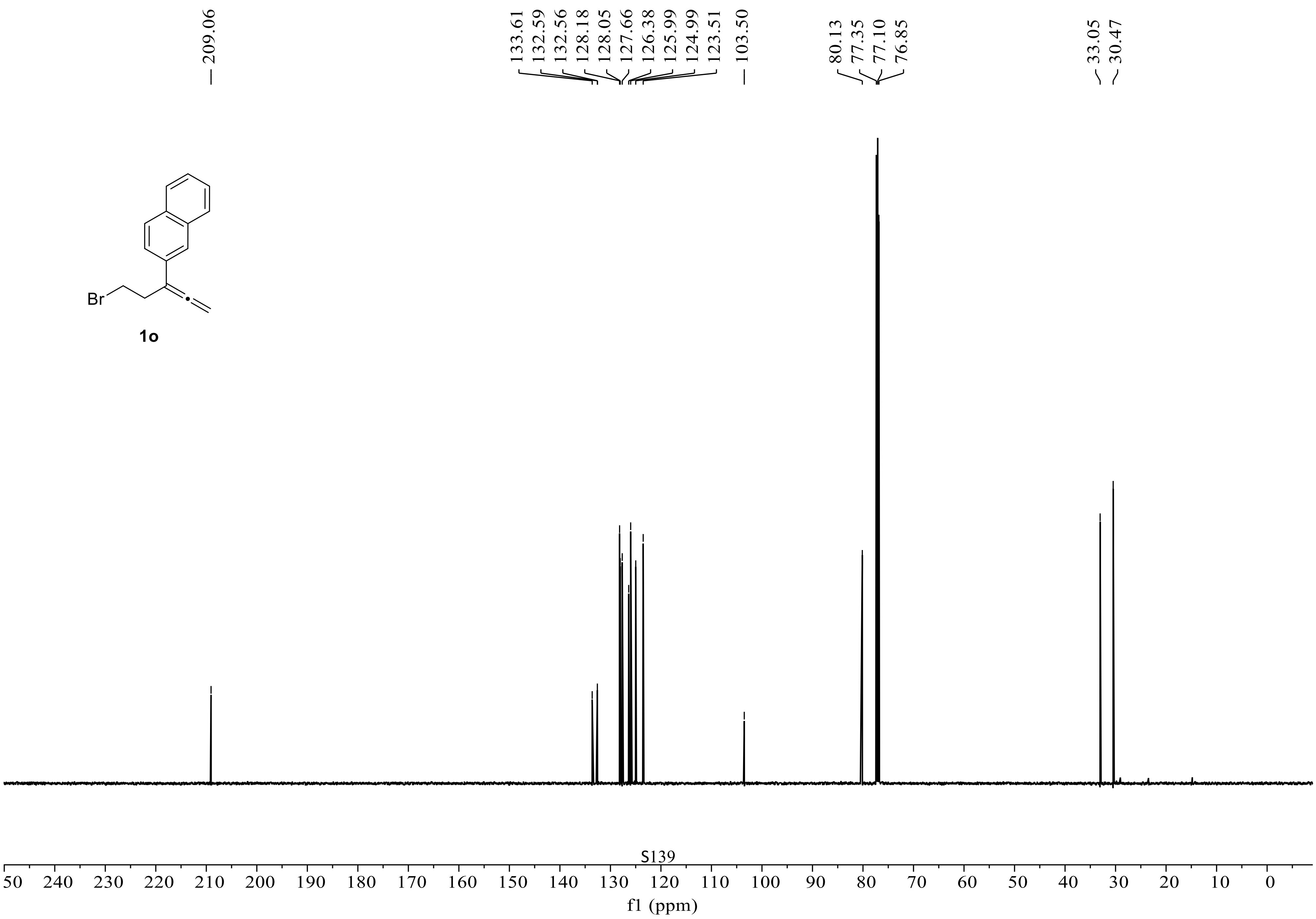

209.06
133.61
132.59
132.56
128.18
128.05
127.66
126.38
125.99
124.99
123.51
103.50
80.13
77.35
77.10
76.85
33.05
30.47
Br
1o
50 240 230 220 210 200 190 180 170 160 150 140 130 120 110 100 90 80 70 60 50 40 30 20 10 0
f1 (ppm)

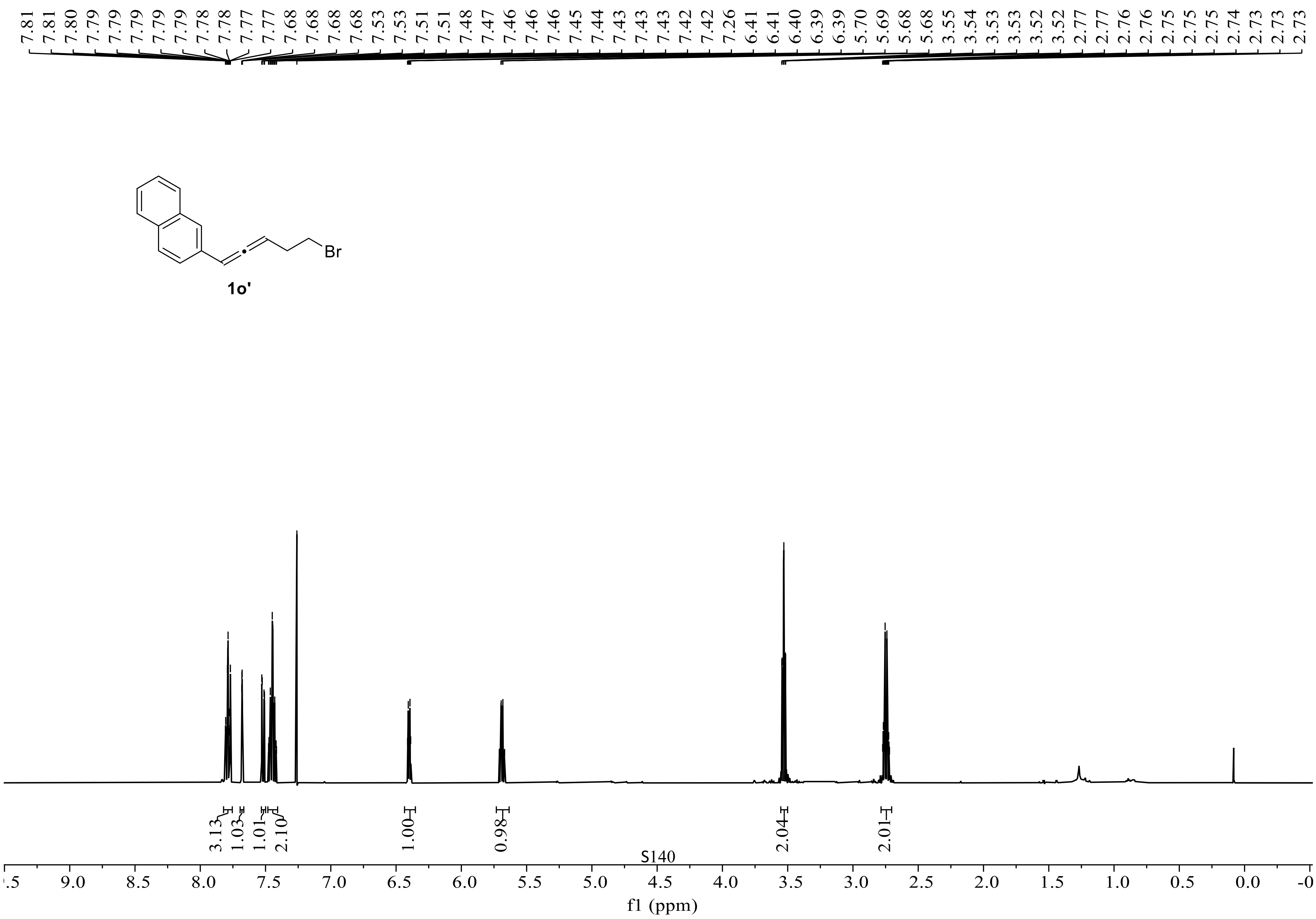
7.81 7.81 7.80 7.79 7.79 7.79 7.79 7.79 7.78 7.78 7.77 7.77 7.68 7.68 7.68 7.68 7.53 7.53 7.51 7.51 7.48 7.47 7.46 7.46 7.46 7.45 7.44 7.43 7.43 7.43 7.42 7.42 7.26 6.41 6.41 6.40 6.39 6.39 5.70 5.69 5.68 5.68 3.55 3.54 3.53 3.53 3.52 3.52 2.77 2.77 2.76 2.76 2.75 2.75 2.75 2.74 2.73 2.73 2.73
Br
1o'
3.13
1.03
1.01
2.10
1.00
0.98
2.04
2.01
9.0 8.5 8.0 7.5 7.0 6.5 6.0 5.5 5.0 4.5 4.0 3.5 3.0 2.5 2.0 1.5 1.0 0.5 0.0
f1 (ppm)

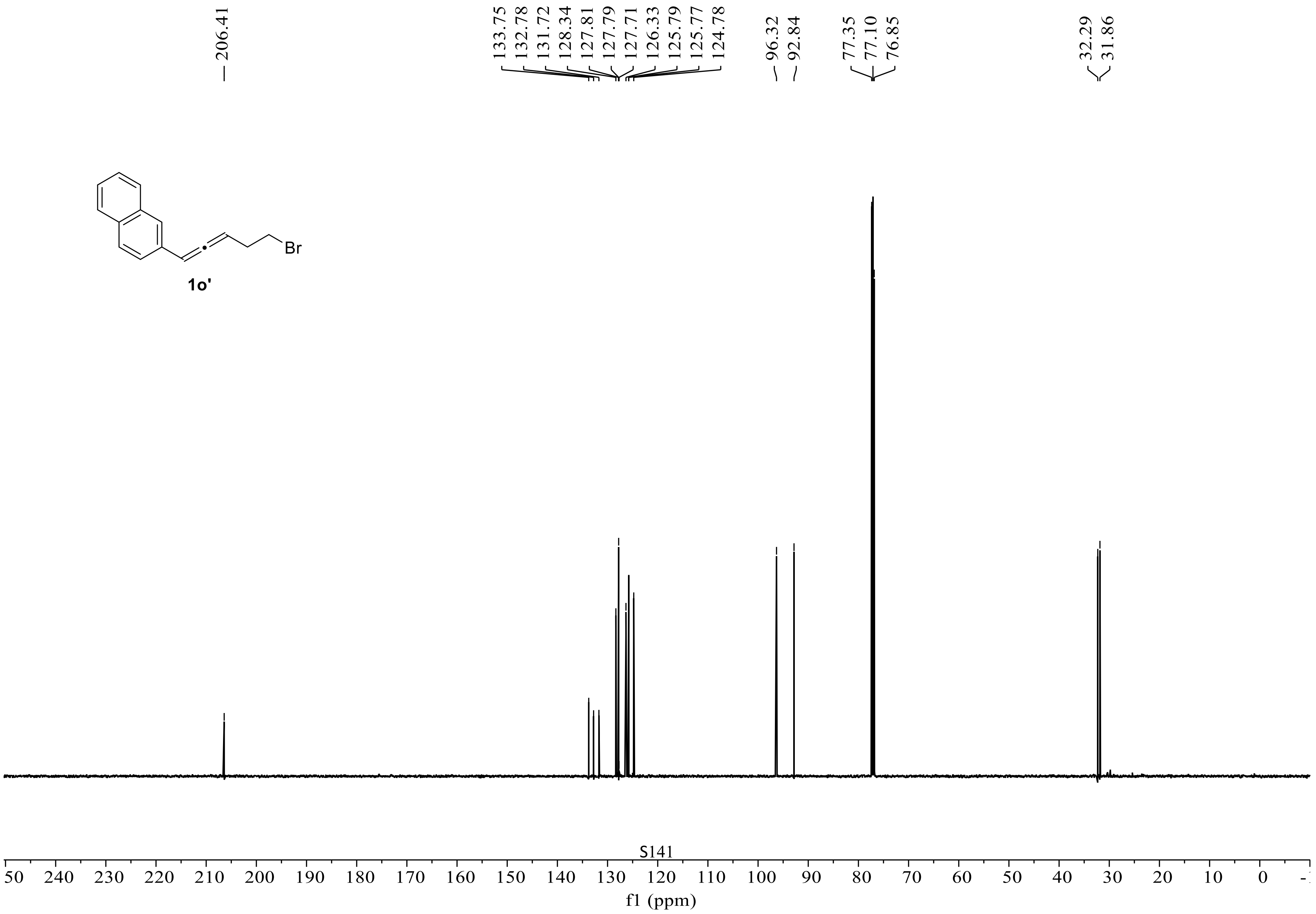
206.41
133.75
132.78
131.72
128.34
127.81
127.79
127.71
126.33
125.79
125.77
124.78
96.32
92.84
77.35
77.10
76.85
32.29
31.86
Br
1o'
50 240 230 220 210 200 190 180 170 160 150 140 130 120 110 100 90 80 70 60 50 40 30 20 10 0
f1 (ppm)

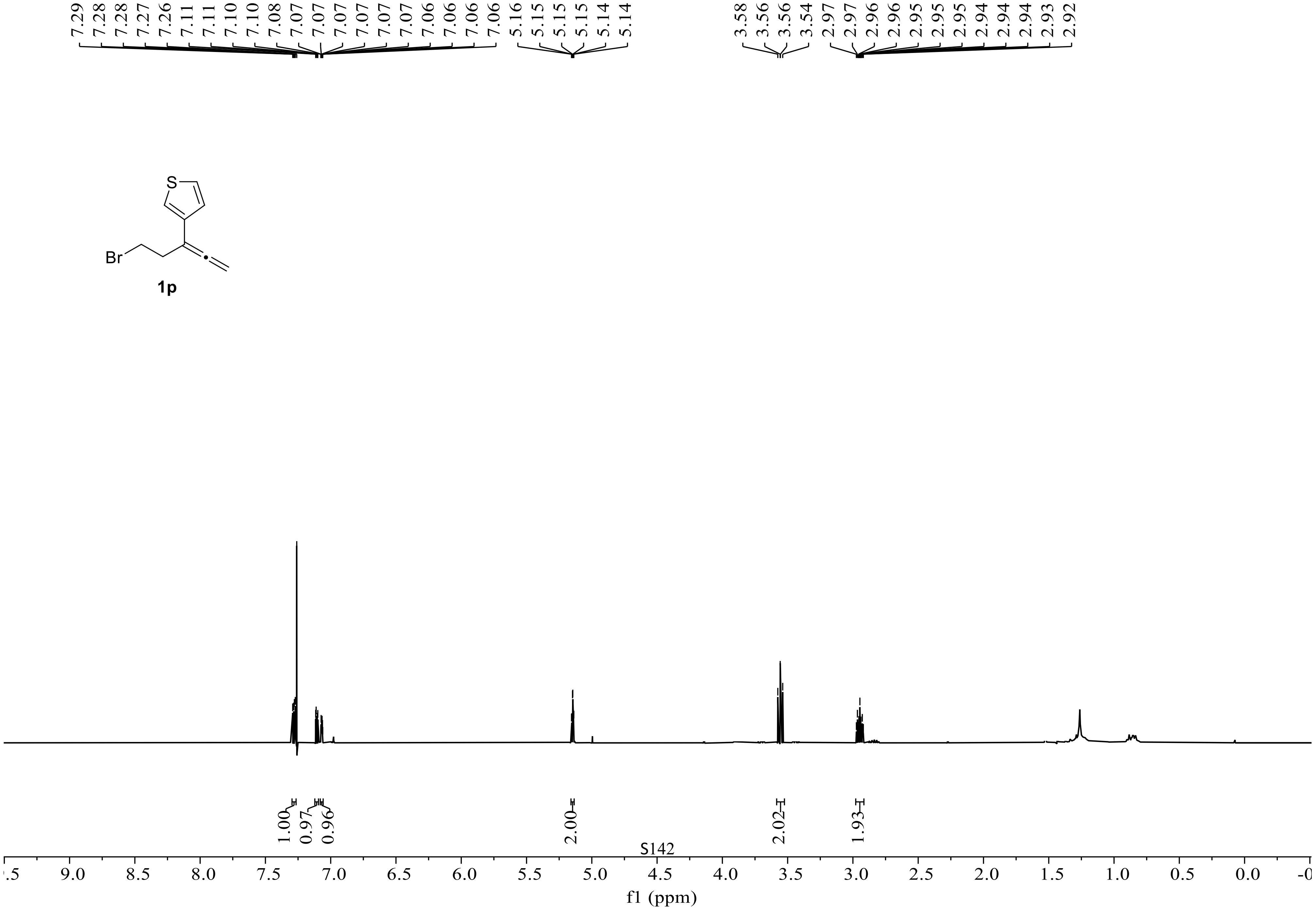
7.29 7.28 7.28 7.27 7.26 7.11 7.11 7.10 7.10 7.08 7.07 7.07 7.07 7.07 7.07 7.06 7.06 7.06 7.06 5.16 5.15 5.15 5.15 5.14 5.14
3.58 3.56 3.56 3.54 2.97 2.97 2.96 2.96 2.95 2.95 2.95 2.94 2.94 2.94 2.93 2.92
S
Br
1p
1.00
0.97
0.96
2.00
2.02
1.93
9.0 8.5 8.0 7.5 7.0 6.5 6.0 5.5 5.0 4.5 4.0 3.5 3.0 2.5 2.0 1.5 1.0 0.5 0.0
f1 (ppm)

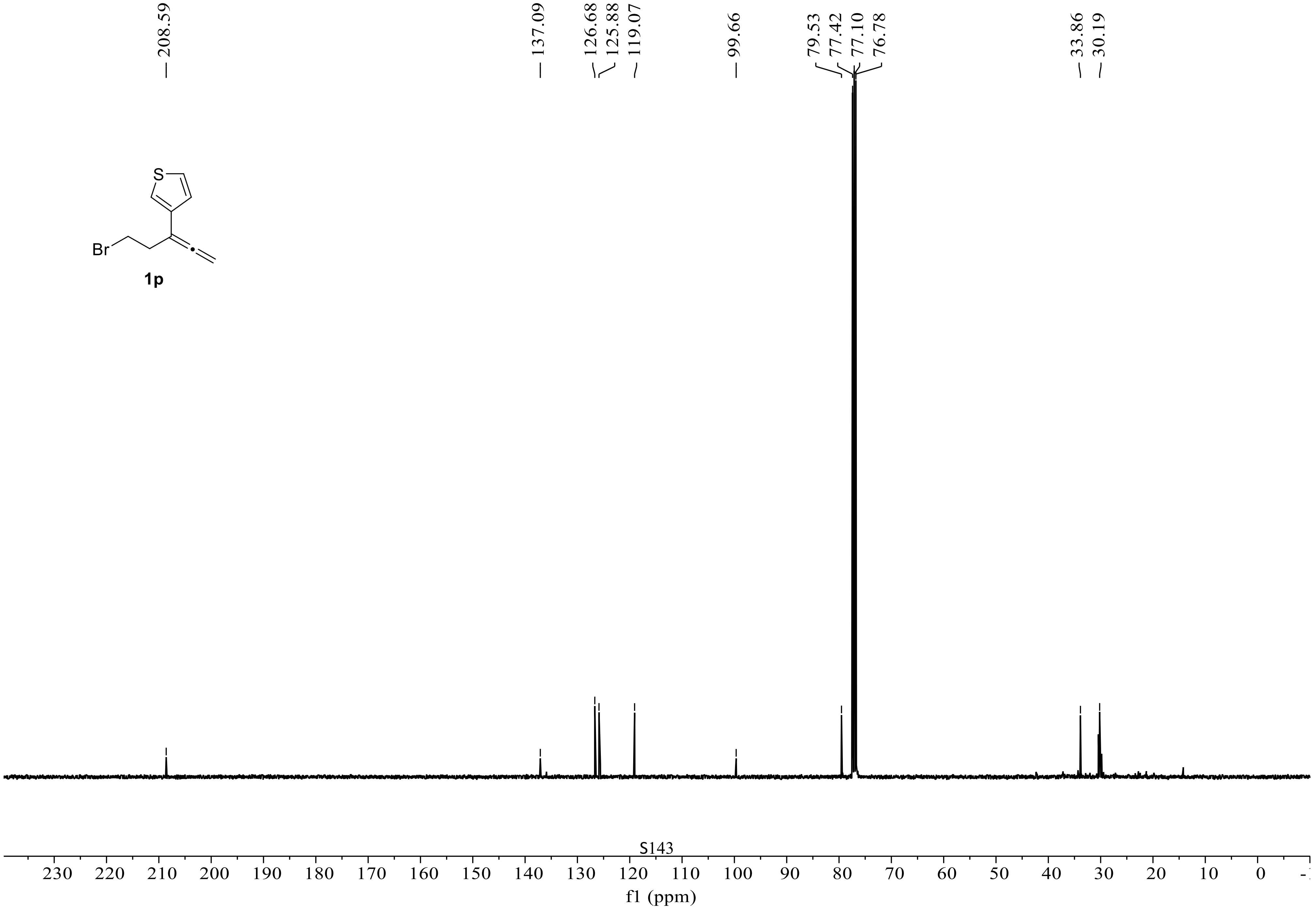
208.59
137.09
126.68
125.88
119.07
99.66
79.53
77.42
77.10
76.78
33.86
30.19
S
Br
230 220 210 200 190 180 170 160 150 140 130 120 110 100 90 80 70 60 50 40 30 20 10 0
f1 (ppm)

1p

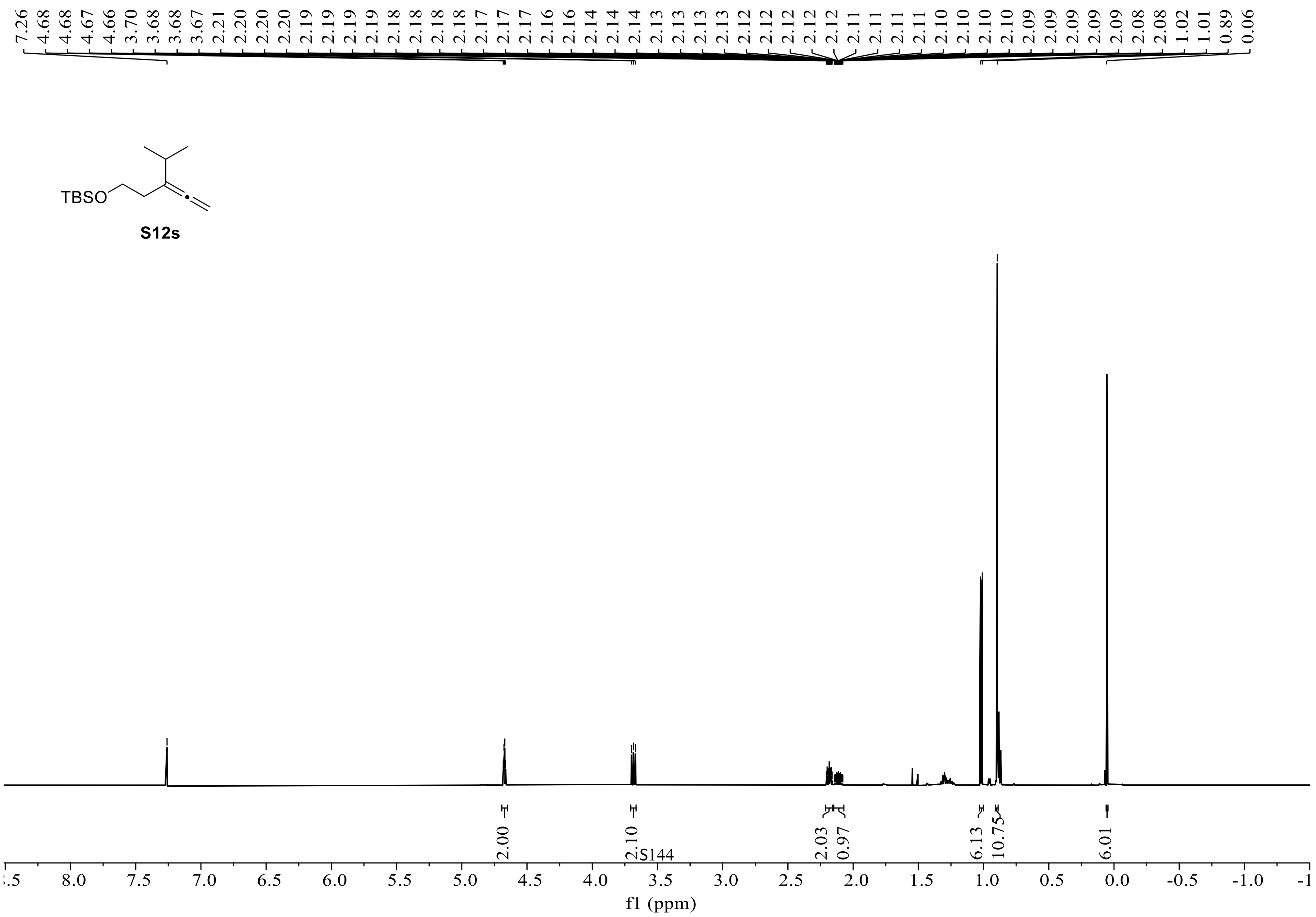
7.26 4.68 4.68 4.67 4.66 3.70 3.68 3.68 3.67 2.21 2.20 2.20 2.20 2.19 2.19 2.19 2.19 2.18 2.18 2.18 2.18 2.17 2.17 2.17 2.16 2.16 2.14 2.14 2.14 2.13 2.13 2.13 2.13 2.12 2.12 2.12 2.12 2.11 2.11 2.11 2.11 2.10 2.10 2.10 2.10 2.09 2.09 2.09 2.09 2.09 2.08 2.08 1.02 1.01 0.89 0.06
TBSO
2.00
2.10
2.03
0.97
6.13
10.75
6.01
8.0 7.5 7.0 6.5 6.0 5.5 5.0 4.5 4.0 3.5 3.0 2.5 2.0 1.5 1.0 0.5 0.0 -0.5 -1.0
f1 (ppm)
S12s

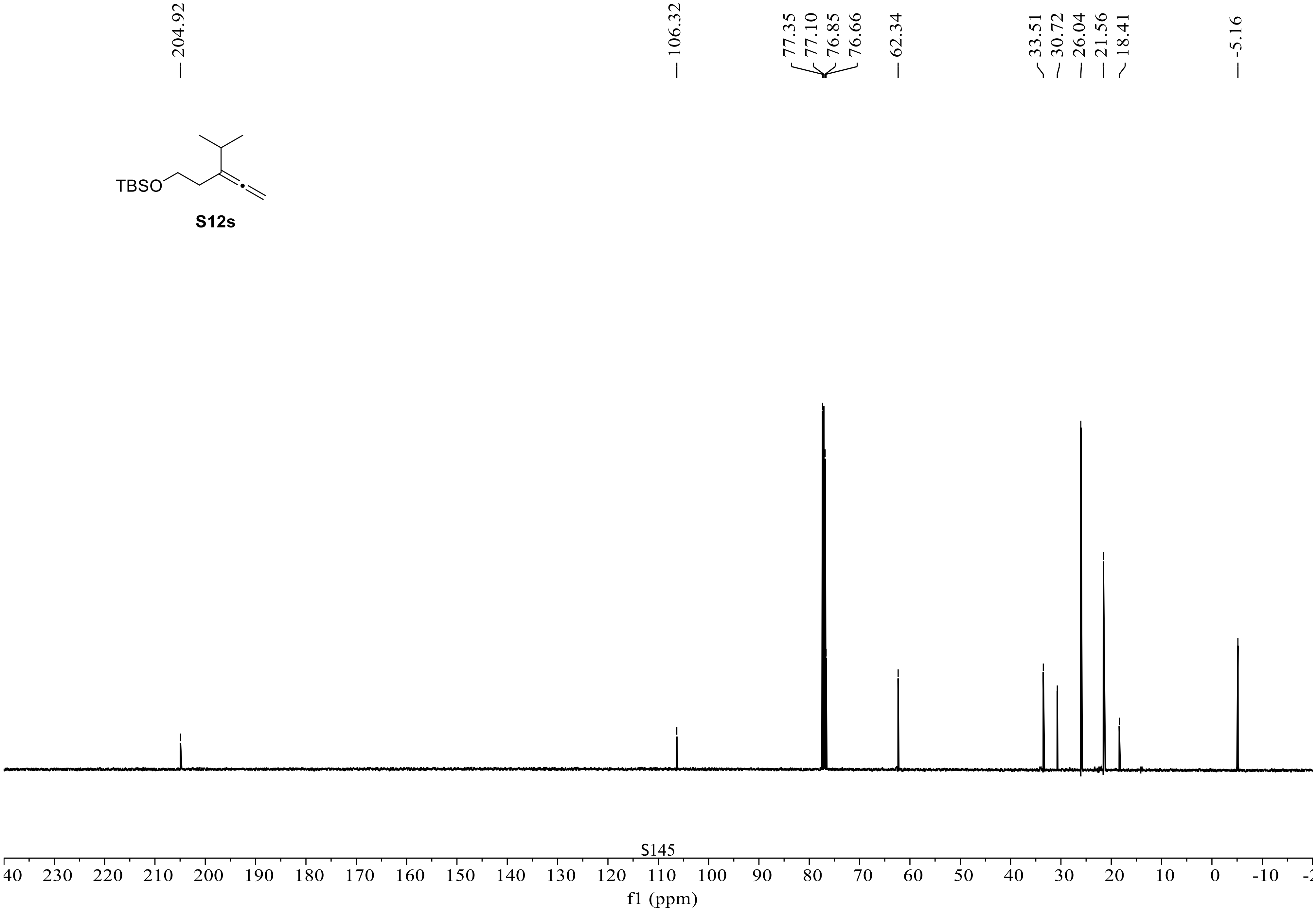
204.92
106.32
77.35
77.10
76.85
76.66
62.34
33.51
30.72
26.04
21.56
18.41
-5.16
TBSO
S12s
40 230 220 210 200 190 180 170 160 150 140 130 120 110 100 90 80 70 60 50 40 30 20 10 0 -10
f1 (ppm)

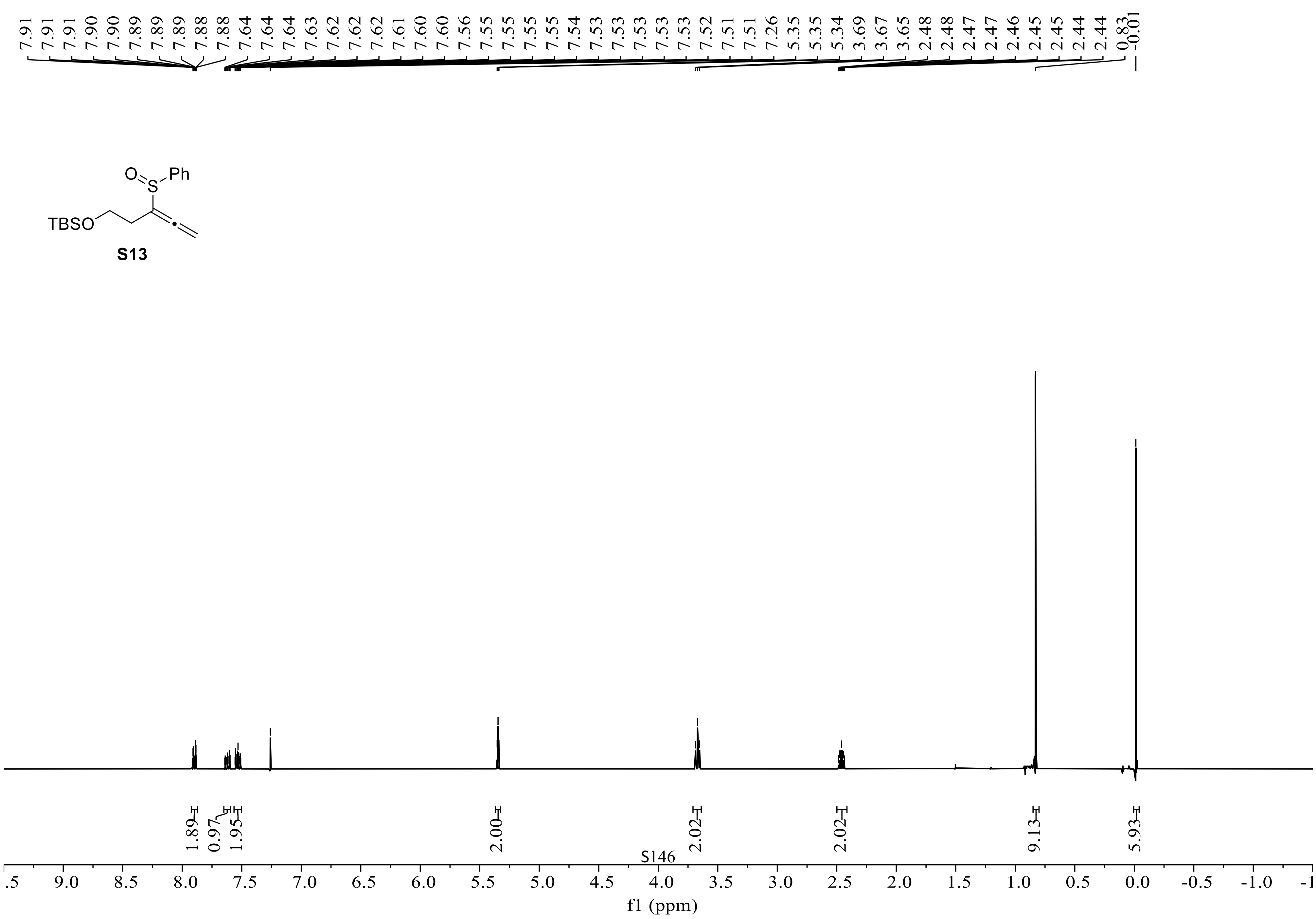
O
S
Ph
TBSO
S13
7.91 7.91 7.91 7.90 7.90 7.89 7.89 7.89 7.88 7.88 7.64 7.64 7.64 7.63 7.62 7.62 7.62 7.61 7.60 7.60 7.56 7.55 7.55 7.55 7.55 7.54 7.53 7.53 7.53 7.53 7.53 7.52 7.51 7.51 7.26 5.35 5.35 5.34 3.69 3.67 3.65 2.48 2.48 2.47 2.47 2.46 2.45 2.45 2.44 2.44 0.83 -0.01
1.89
0.97
1.95
2.00
2.02
2.02
9.13
5.93
.5 9.0 8.5 8.0 7.5 7.0 6.5 6.0 5.5 5.0 4.5 4.0 3.5 3.0 2.5 2.0 1.5 1.0 0.5 0.0 -0.5 -1.0 -1
f1 (ppm)

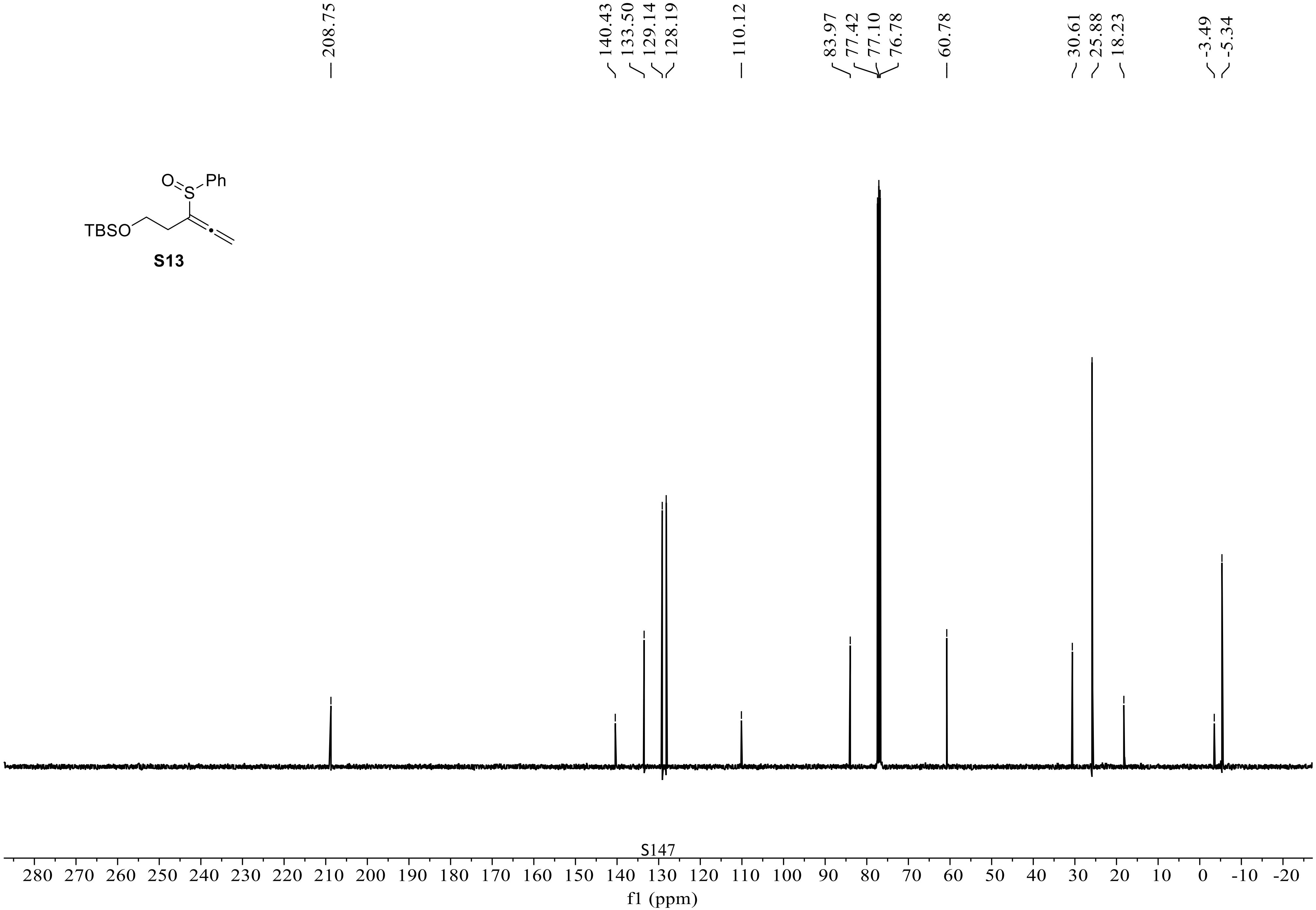
208.75
140.43
133.50
129.14
128.19
110.12
83.97
77.42
77.10
76.78
60.78
30.61
25.88
18.23
-3.49
-5.34
280 270 260 250 240 230 220 210 200 190 180 170 160 150 140 130 120 110 100 90 80 70 60 50 40 30 20 10 0 -10 -20
f1 (ppm)
O
S
Ph
TBSO

S13

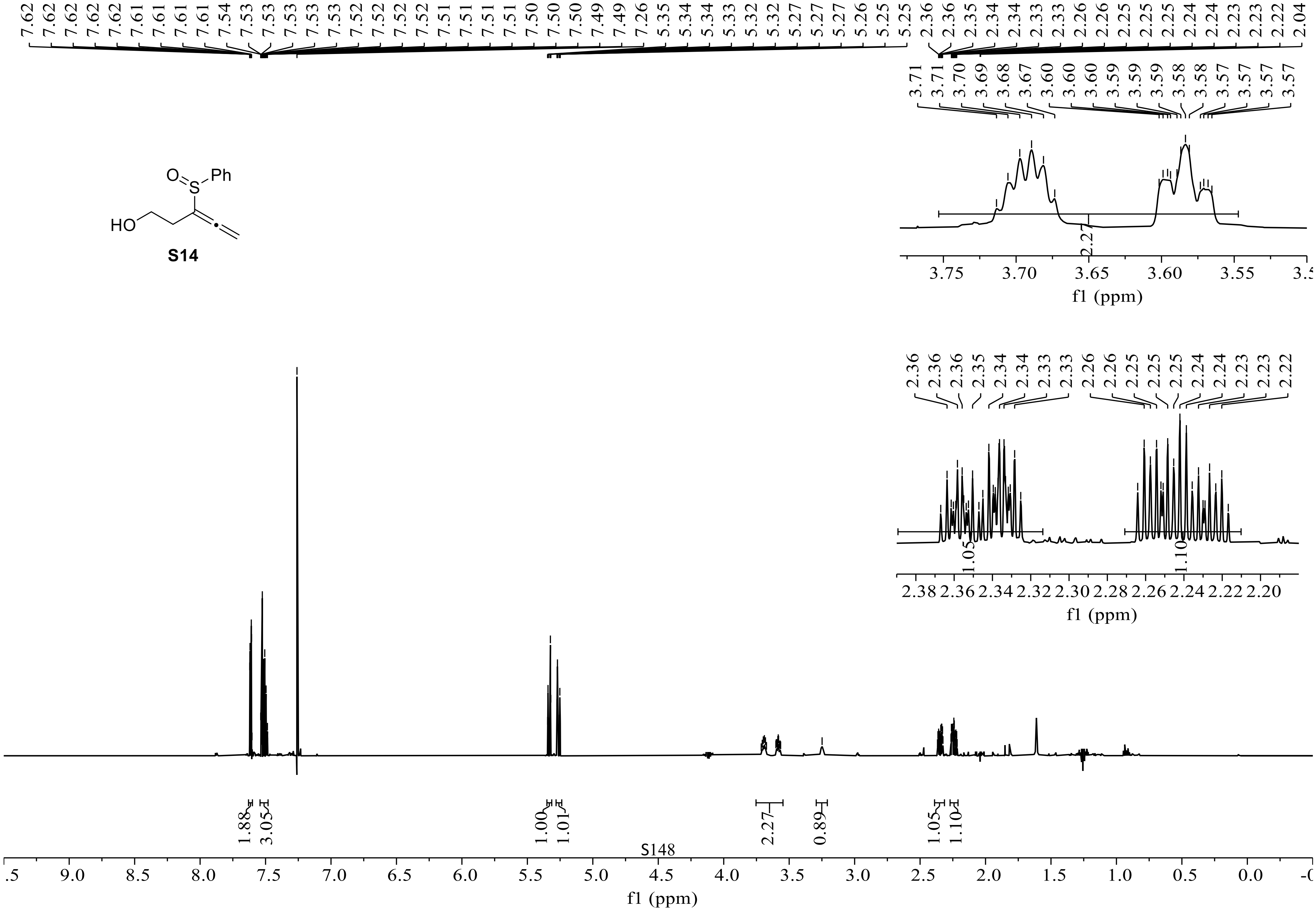

S14

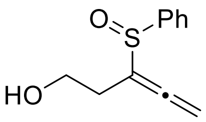
S14

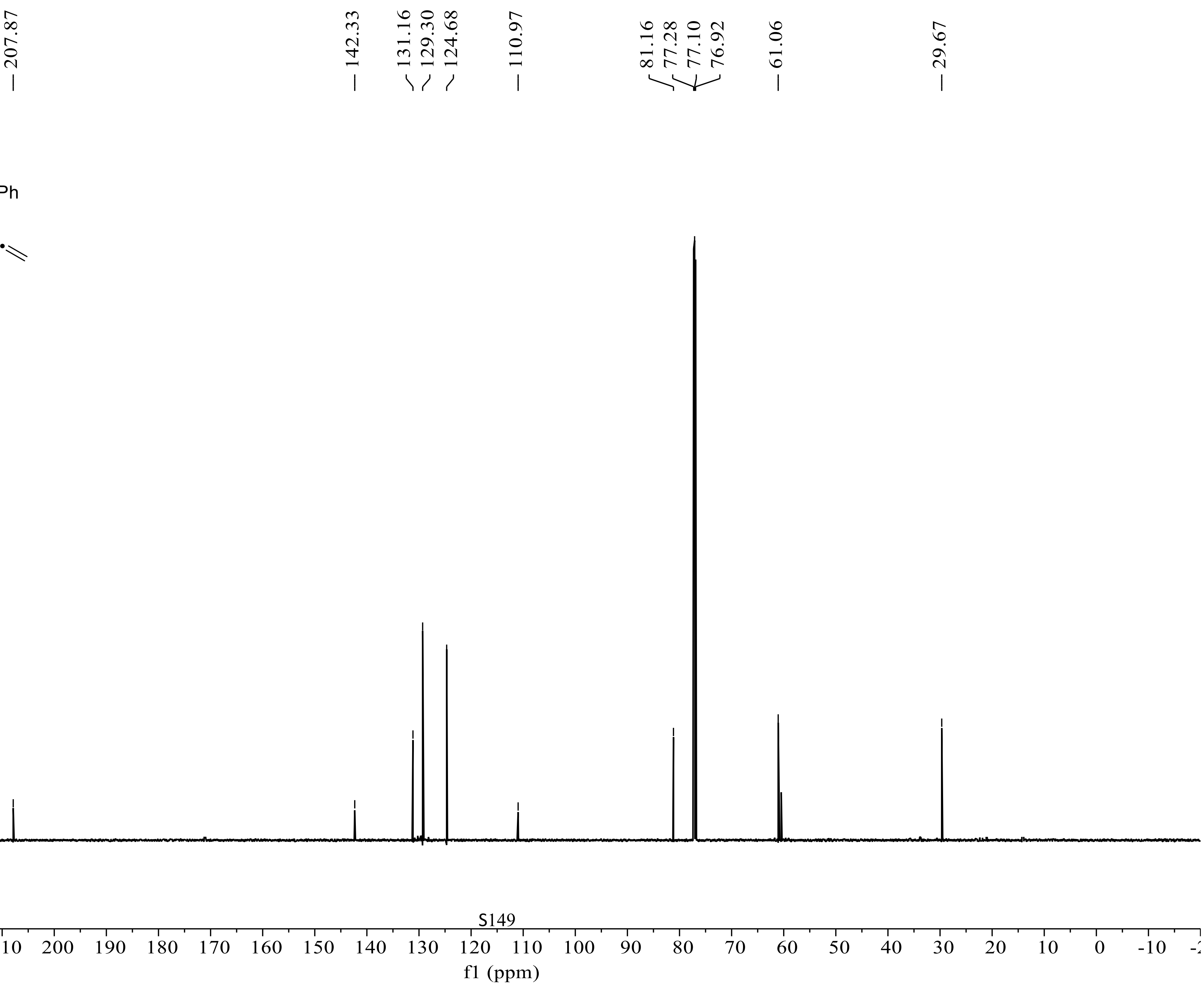

207.87
142.33
131.16
129.30
124.68
110.97
81.16
77.28
77.10
76.92
61.06
29.67
50 240 230 220 210 200 190 180 170 160 150 140 130 120 110 100 90 80 70 60 50 40 30 20 10 0 -10
f1 (ppm)

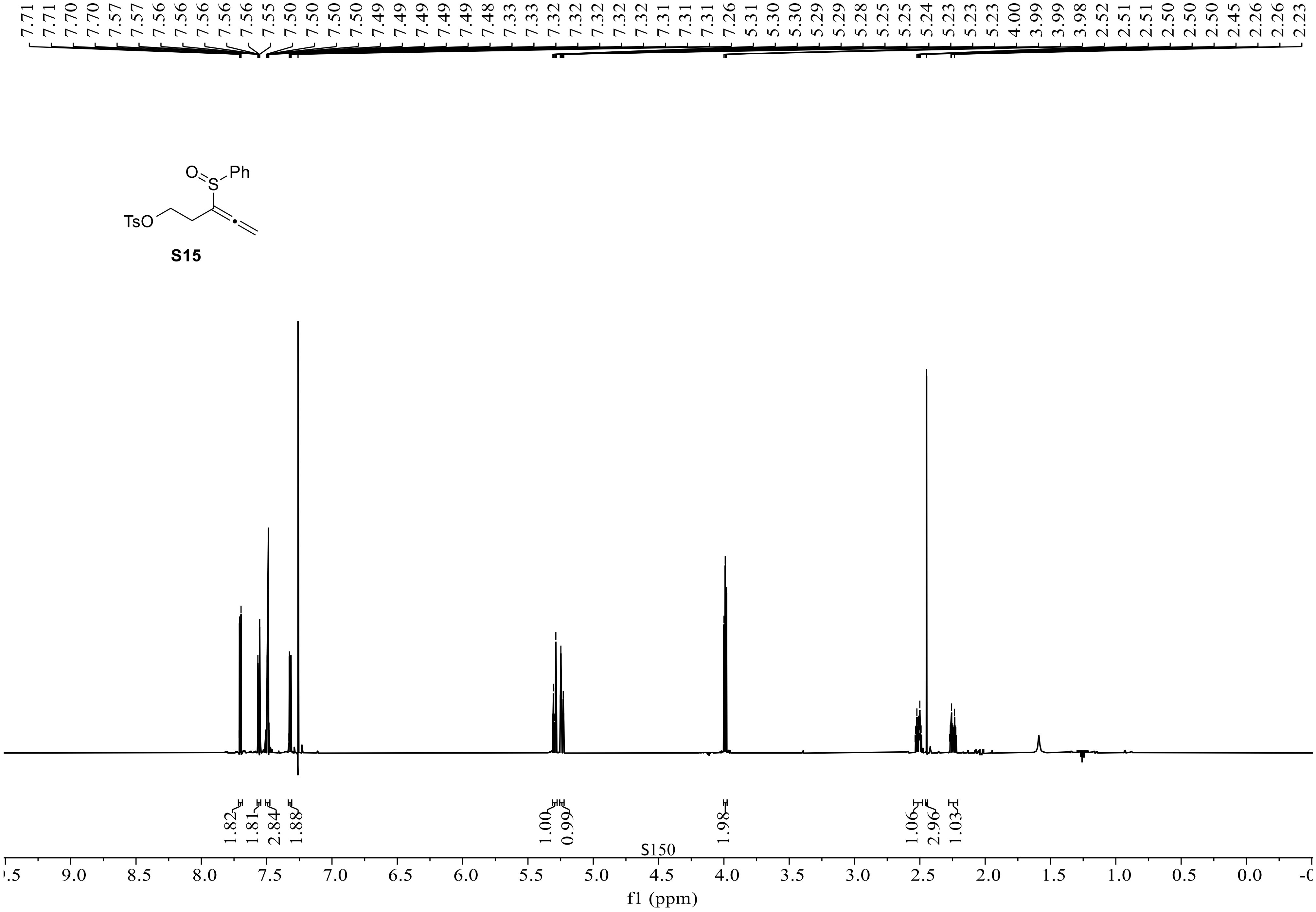
7.71 7.71 7.70 7.70 7.57 7.57 7.56 7.56 7.56 7.56 7.56 7.55 7.50 7.50 7.50 7.50 7.49 7.49 7.49 7.49 7.49 7.48 7.33 7.33 7.32 7.32 7.32 7.32 7.32 7.31 7.31 7.31 7.26 5.31 5.30 5.30 5.29 5.29 5.28 5.25 5.25 5.24 5.23 5.23 5.23 4.00 3.99 3.99 3.98 2.52 2.51 2.51 2.50 2.50 2.50 2.45 2.26 2.26 2.23
O
S
Ph
TsO
1.82
1.81
2.84
1.88
1.00
0.99
1.98
1.06
2.96
1.03
9.0 8.5 8.0 7.5 7.0 6.5 6.0 5.5 5.0 4.5 4.0 3.5 3.0 2.5 2.0 1.5 1.0 0.5 0.0
f1 (ppm)

S15

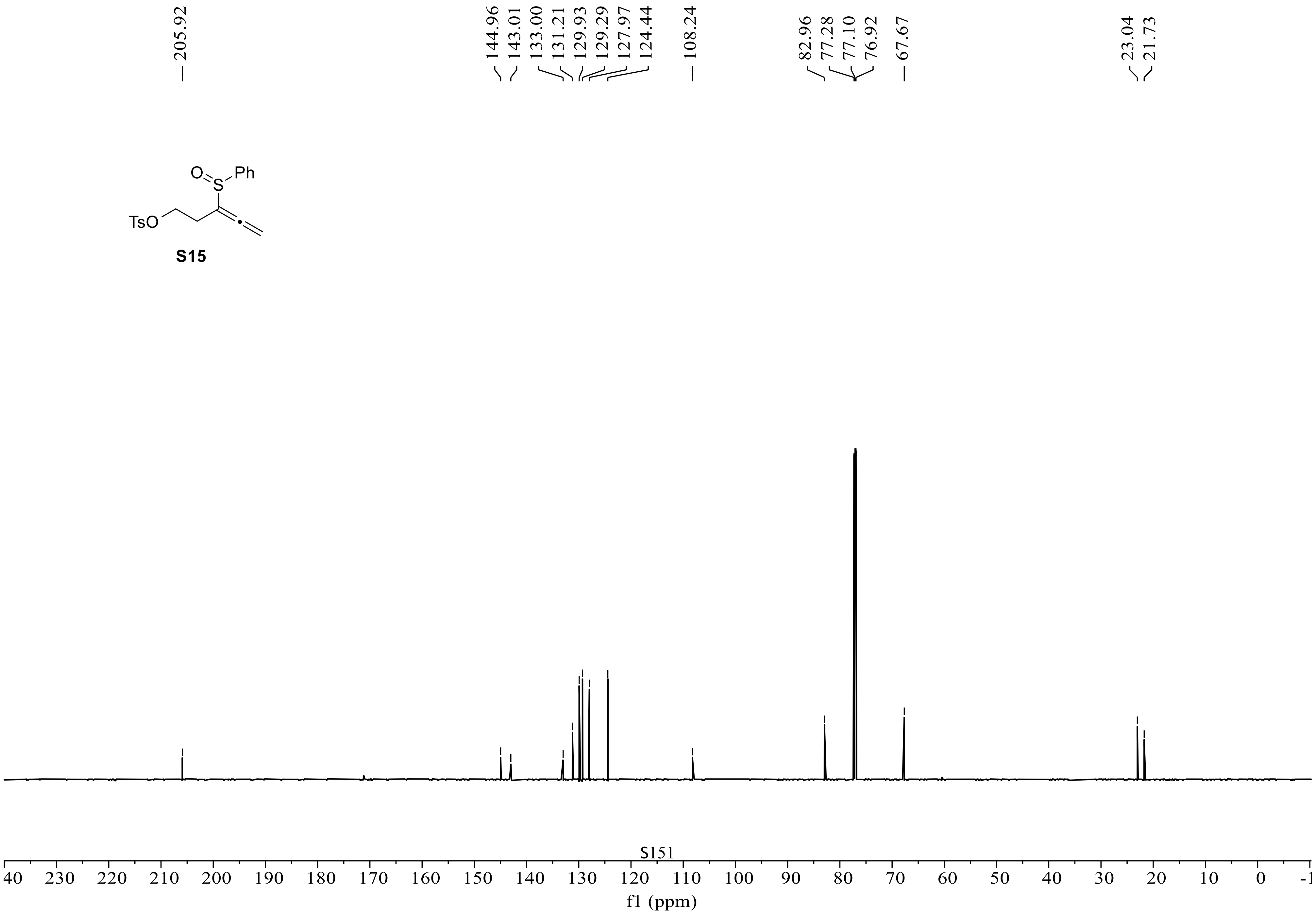
205.92
144.96
143.01
133.00
131.21
129.93
129.29
127.97
124.44
108.24
82.96
77.28
77.10
76.92
67.67
23.04
21.73
O
S
Ph
TsO
S15
40 230 220 210 200 190 180 170 160 150 140 130 120 110 100 90 80 70 60 50 40 30 20 10 0 -1
f1 (ppm)

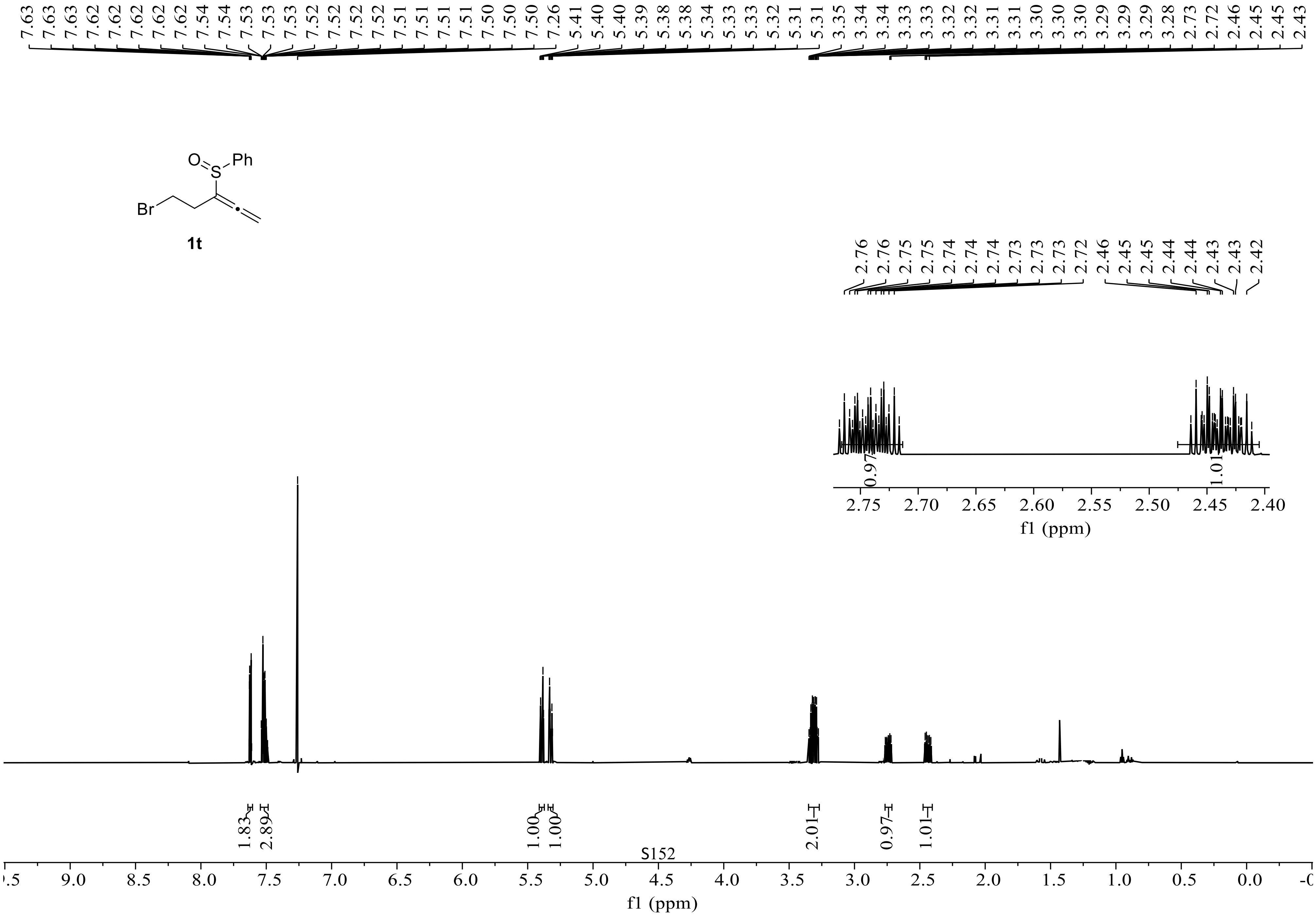
O
S
Ph
Br
1t
7.63
7.63
7.63
7.62
7.62
7.62
7.62
7.62
7.54
7.54
7.53
7.53
7.53
7.52
7.52
7.52
7.52
7.51
7.51
7.51
7.50
7.50
7.26
5.41
5.40
5.40
5.39
5.38
5.38
5.34
5.33
5.33
5.32
5.31
5.31
3.35
3.34
3.34
3.33
3.33
3.32
3.32
3.31
3.31
3.30
3.30
3.30
3.29
3.29
3.29
3.28
2.73
2.72
2.46
2.45
2.45
2.43
2.76
2.76
2.75
2.75
2.74
2.74
2.74
2.73
2.73
2.73
2.72
2.46
2.45
2.45
2.44
2.44
2.43
2.43
2.42
0.97
1.01
2.75
2.70
2.65
2.60
2.55
2.50
2.45
2.40
f1 (ppm)
1.83
2.89
1.00
1.00
2.01
0.97
1.01
9.0
8.5
8.0
7.5
7.0
6.5
6.0
5.5
5.0
4.5
4.0
3.5
3.0
2.5
2.0
1.5
1.0
0.5
0.0
f1 (ppm)

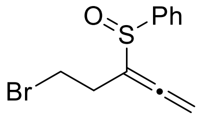
O
S
Ph
Br

1t

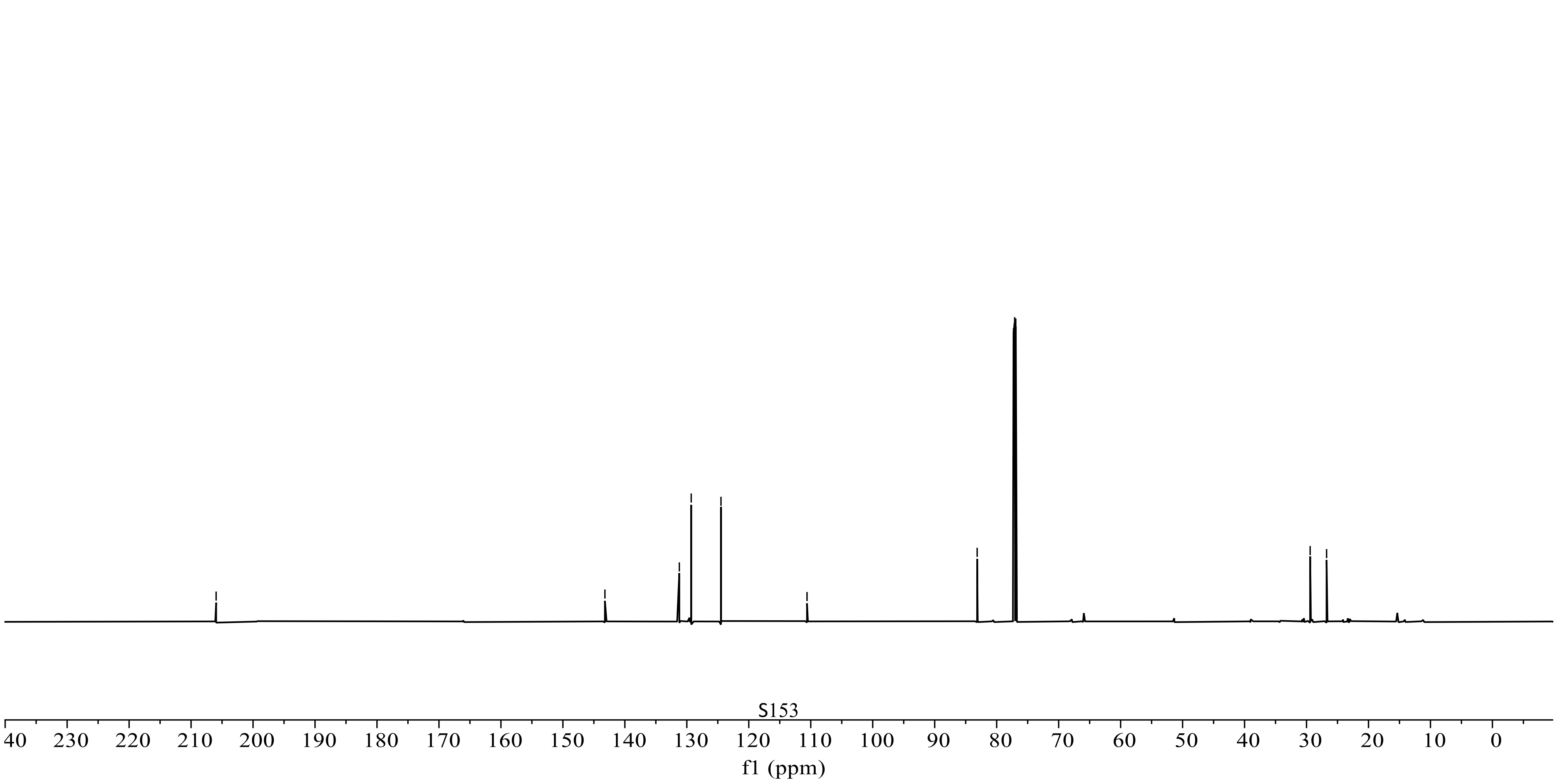
205.96
143.22
131.21
129.29
124.49
110.60
83.15
77.28
77.10
76.92
29.41
26.76
40 230 220 210 200 190 180 170 160 150 140 130 120 110 100 90 80 70 60 50 40 30 20 10 0
f1 (ppm)

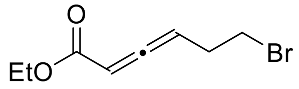
EtO
O
Br

1u

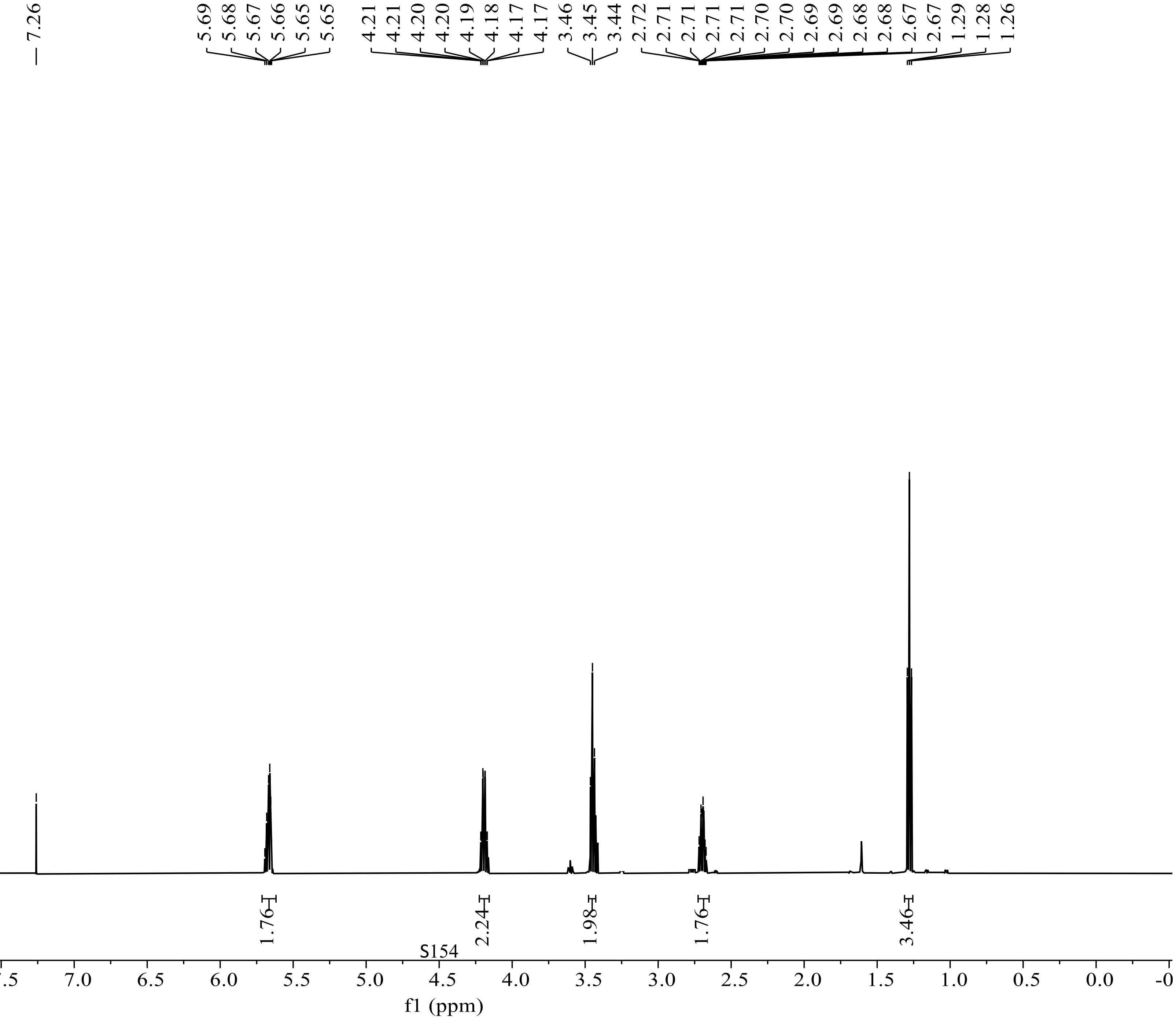
7.26
5.69 5.68 5.67 5.66 5.65 5.65
4.21 4.21 4.20 4.20 4.19 4.18 4.17 4.17
3.46 3.45 3.44
2.72 2.71 2.71 2.71 2.71 2.70 2.70 2.69 2.69 2.68 2.68 2.67 2.67
1.29 1.28 1.26
1.76
2.24
1.98
1.76
3.46
9.0 8.5 8.0 7.5 7.0 6.5 6.0 5.5 5.0 4.5 4.0 3.5 3.0 2.5 2.0 1.5 1.0 0.5 0.0
f1 (ppm)

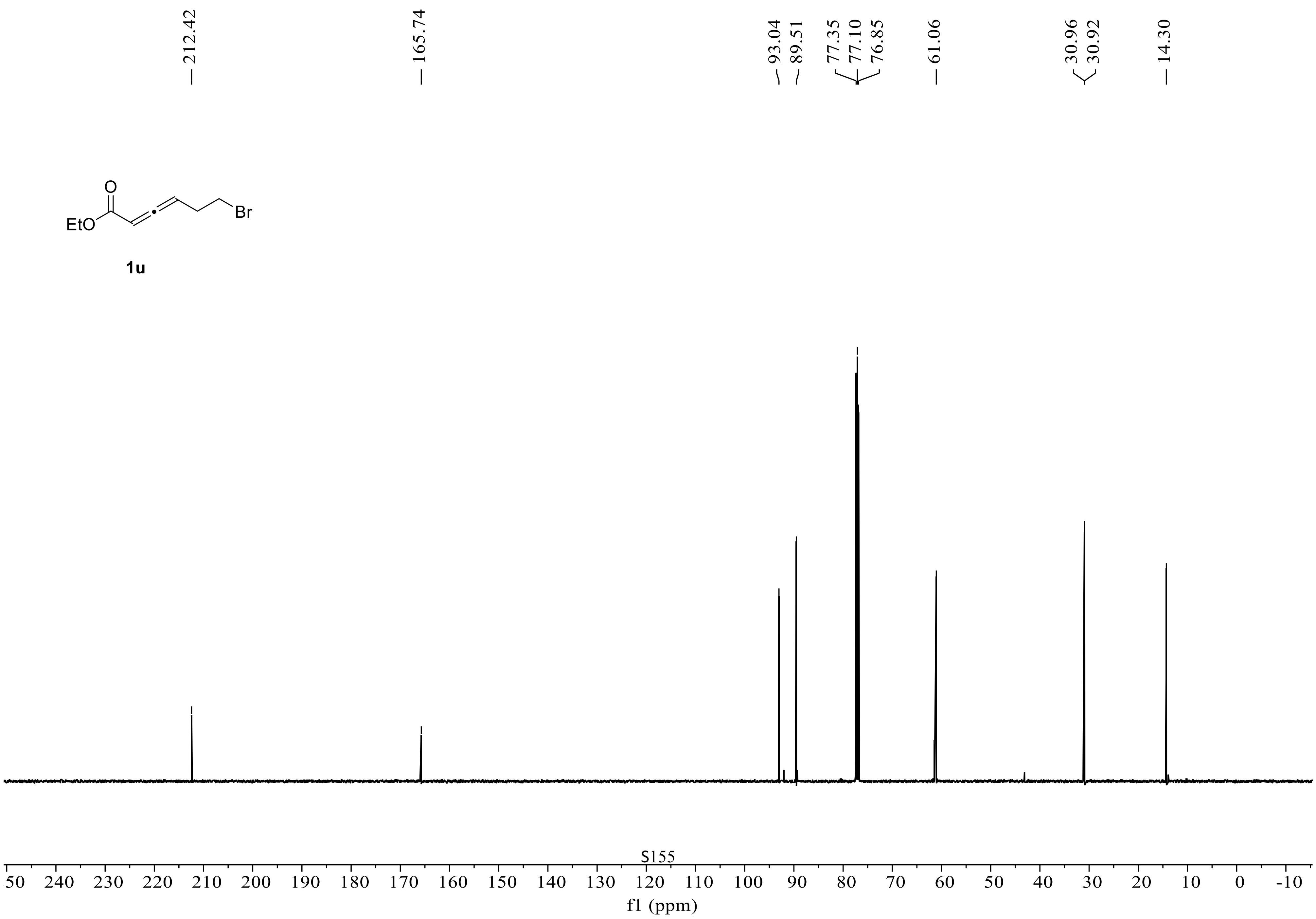
212.42
165.74
93.04
89.51
77.35
77.10
76.85
61.06
30.96
30.92
14.30
EtO
O
Br
1u
50 240 230 220 210 200 190 180 170 160 150 140 130 120 110 100 90 80 70 60 50 40 30 20 10 0 -10
f1 (ppm)

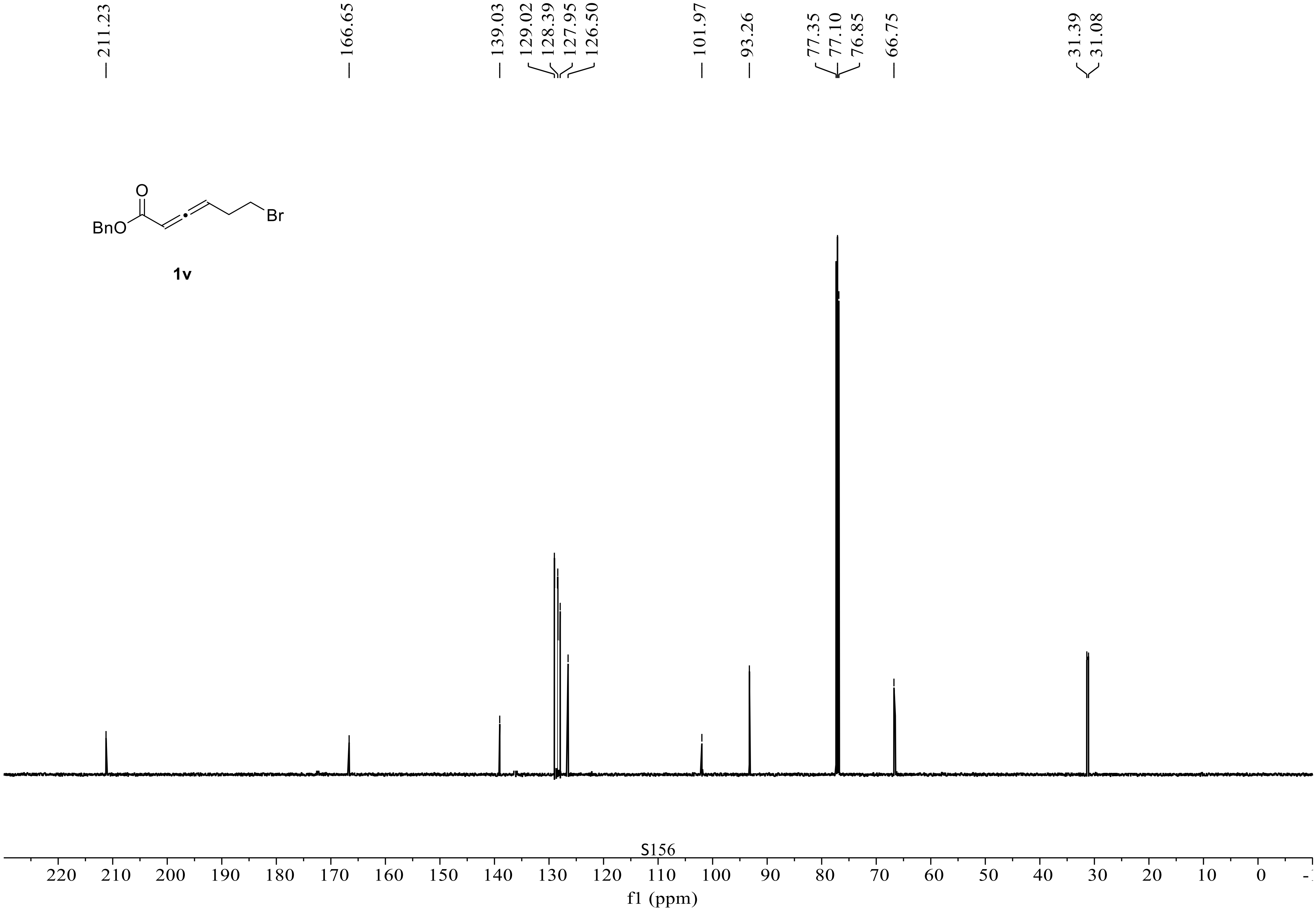
211.23
166.65
139.03
129.02
128.39
127.95
126.50
101.97
93.26
77.35
77.10
76.85
66.75
31.39
31.08
O
BnO
Br
1v
220 210 200 190 180 170 160 150 140 130 120 110 100 90 80 70 60 50 40 30 20 10 0
f1 (ppm)

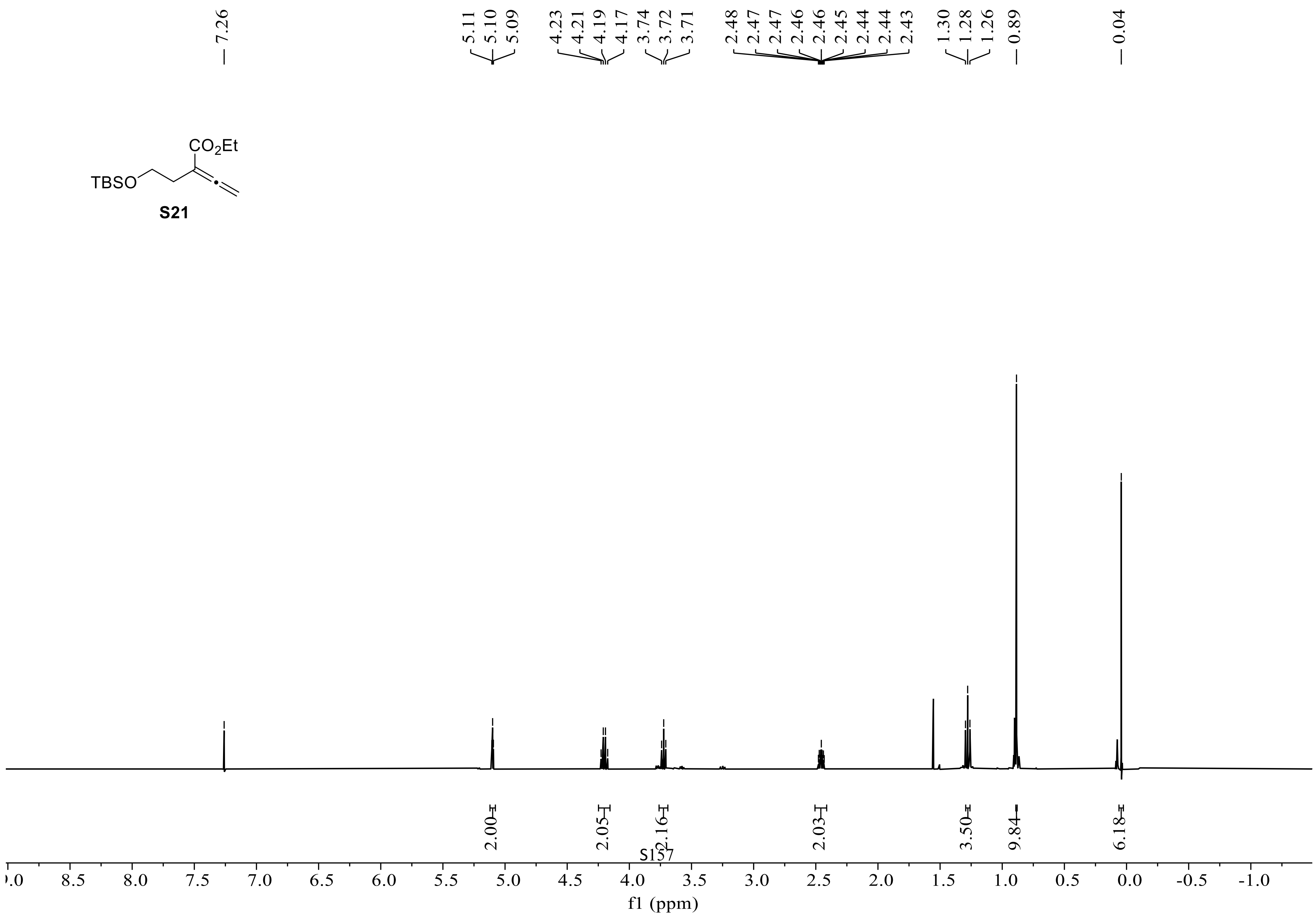
CO2Et
TBSO
S21
7.26
5.11 5.10 5.09
4.23 4.21 4.19 4.17
3.74 3.72 3.71
2.48 2.47 2.47 2.46 2.46 2.45 2.44 2.44 2.43
1.30 1.28 1.26
0.89
0.04
2.00 2.05 2.16 2.03 3.50 9.84 6.18
8.5 8.0 7.5 7.0 6.5 6.0 5.5 5.0 4.5 4.0 3.5 3.0 2.5 2.0 1.5 1.0 0.5 0.0 -0.5 -1.0
f1 (ppm)

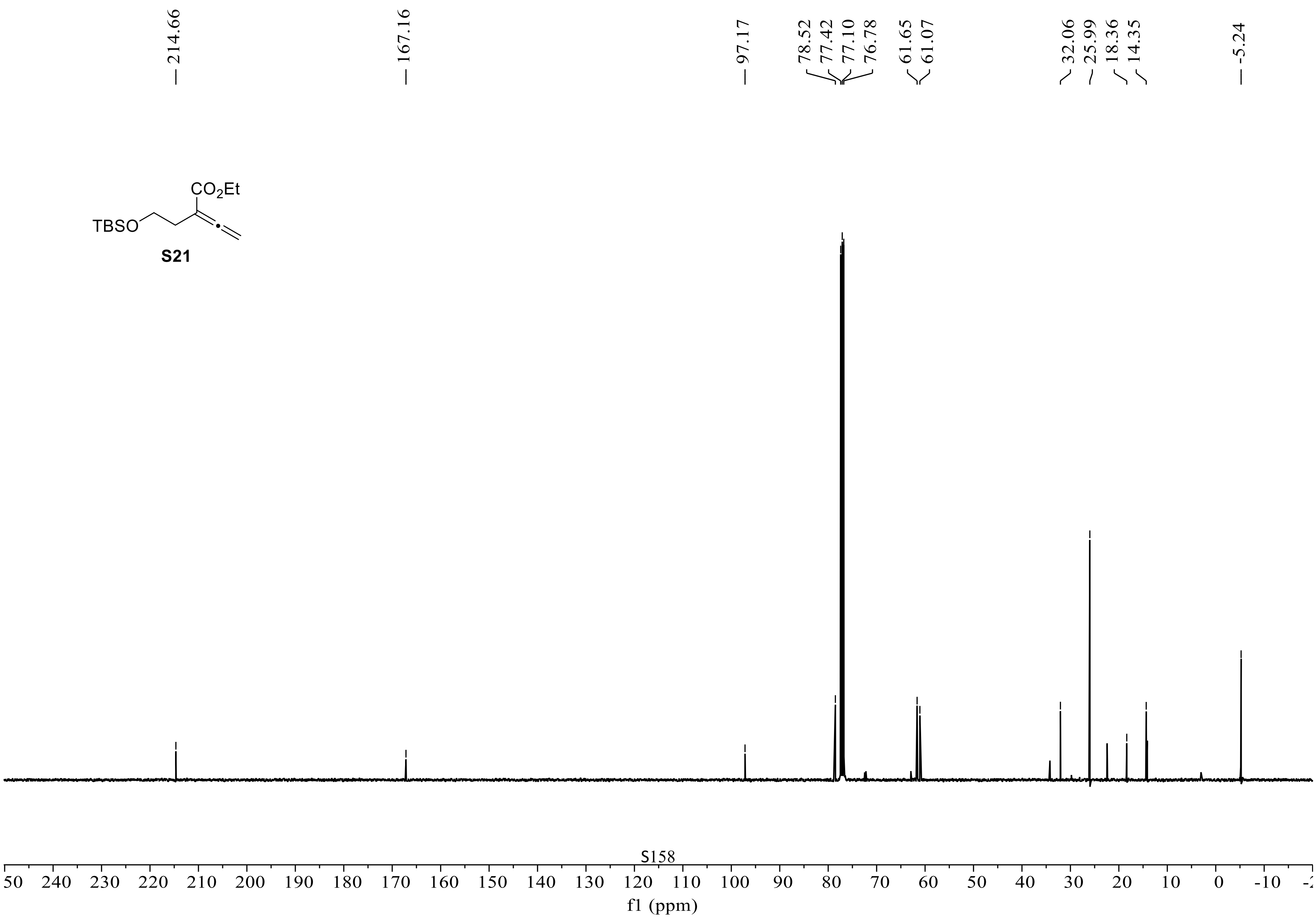

214.66
167.16
97.17
78.52
77.42
77.10
76.78
61.65
61.07
32.06
25.99
18.36
14.35
-5.24
CO2Et
TBSO
S21
f1 (ppm)

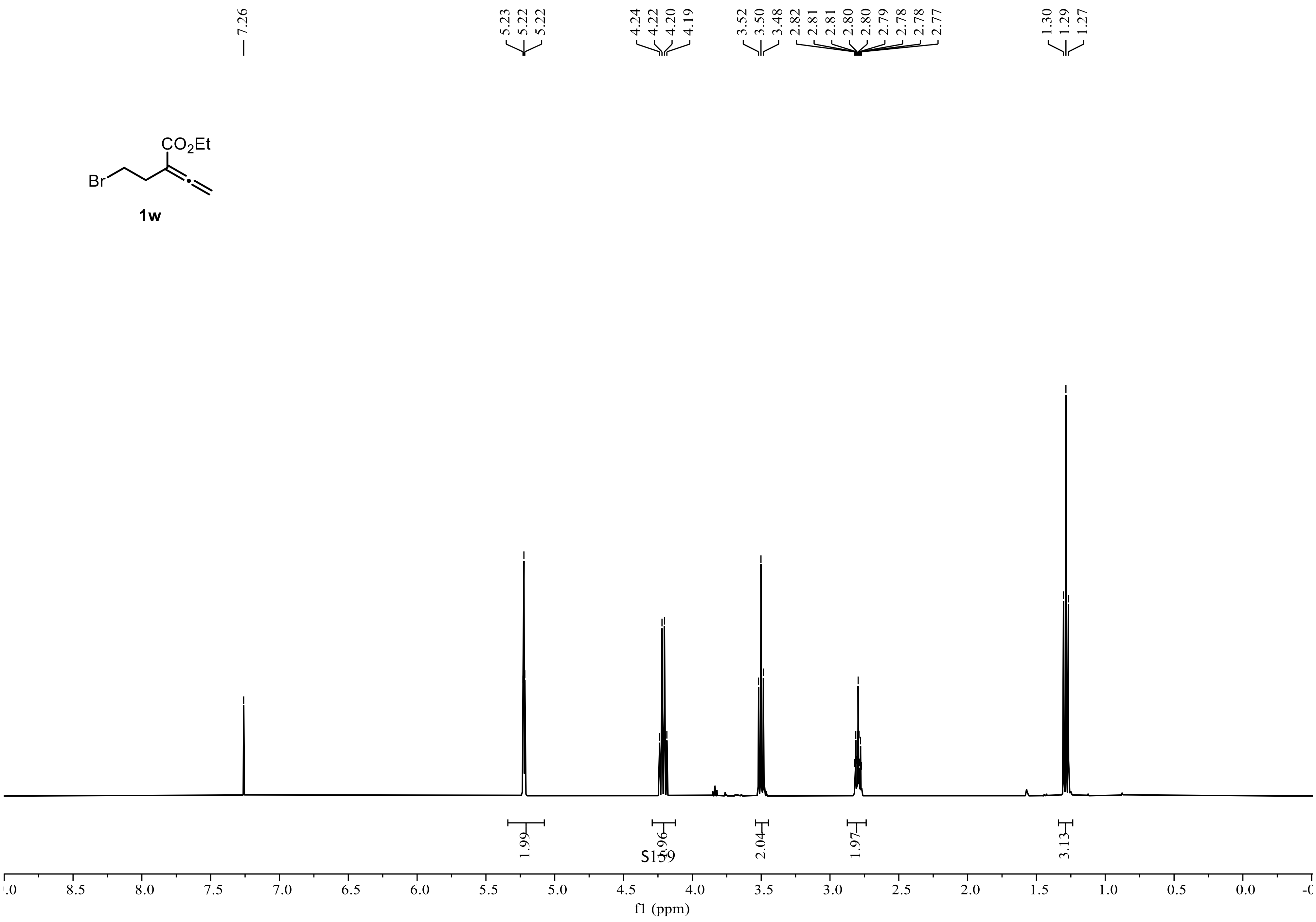
7.26
5.23
5.22
5.22
4.24
4.22
4.20
4.19
3.52
3.50
3.48
2.82
2.81
2.81
2.80
2.80
2.79
2.78
2.78
2.77
1.30
1.29
1.27
1.99
1.96
2.04
1.97
3.13
8.5
8.0
7.5
7.0
6.5
6.0
5.5
5.0
4.5
4.0
3.5
3.0
2.5
2.0
1.5
1.0
0.5
0.0
f1 (ppm)

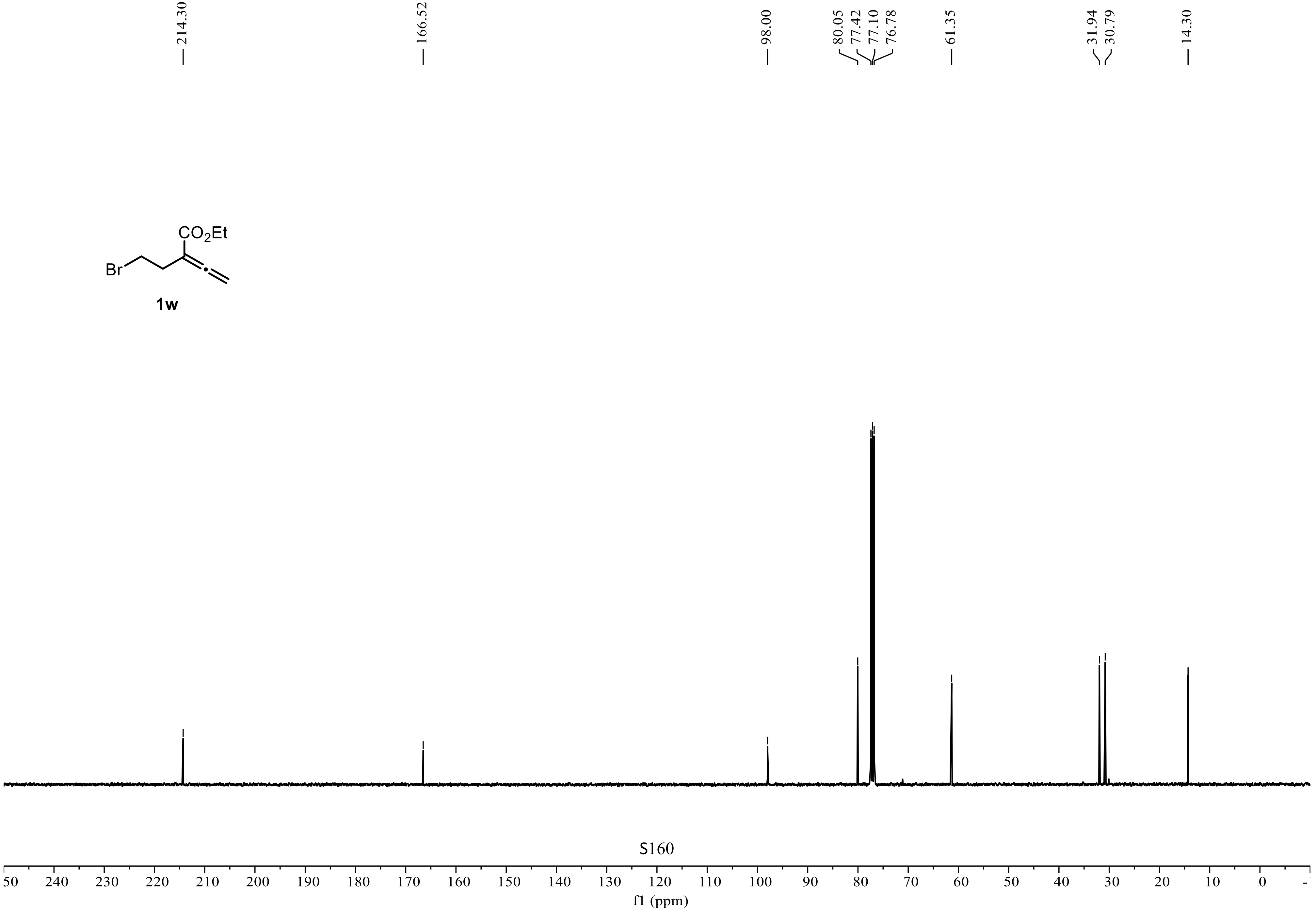
CO2Et
Br
1w
214.30
166.52
98.00
80.05
77.42
77.10
76.78
61.35
31.94
30.79
14.30
50 240 230 220 210 200 190 180 170 160 150 140 130 120 110 100 90 80 70 60 50 40 30 20 10 0
f1 (ppm)

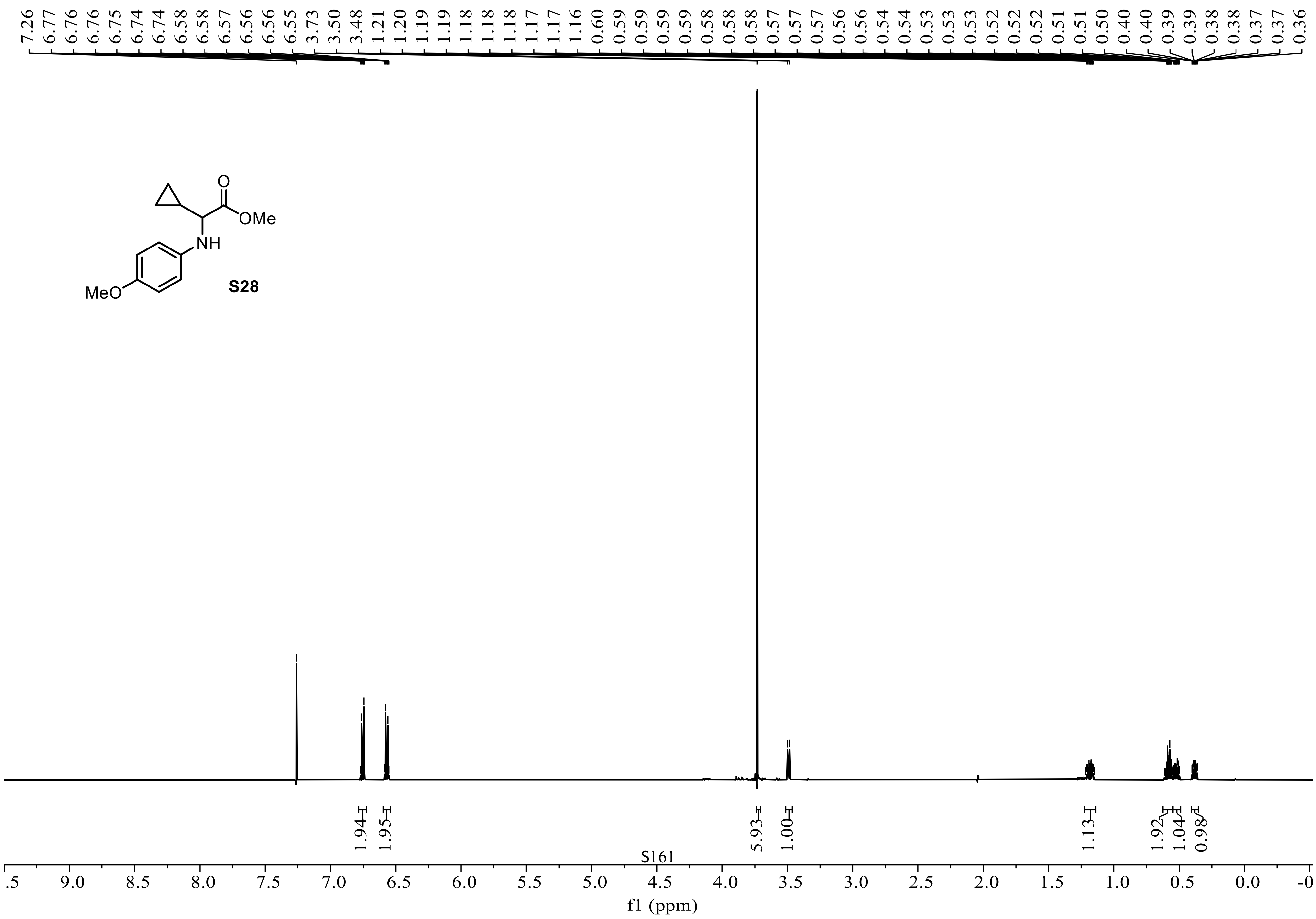
7.26 6.77 6.76 6.76 6.75 6.74 6.74 6.58 6.58 6.57 6.56 6.56 6.55 3.73 3.50 3.48 1.21 1.20 1.19 1.19 1.18 1.18 1.18 1.17 1.17 1.16 0.60 0.59 0.59 0.59 0.59 0.58 0.58 0.58 0.57 0.57 0.57 0.56 0.56 0.54 0.54 0.53 0.53 0.53 0.52 0.52 0.52 0.51 0.51 0.50 0.40 0.40 0.39 0.39 0.38 0.38 0.37 0.37 0.36
O
OMe
NH
MeO
S28
1.94
1.95
5.93
1.00
1.13
1.92
1.04
0.98
f1 (ppm)

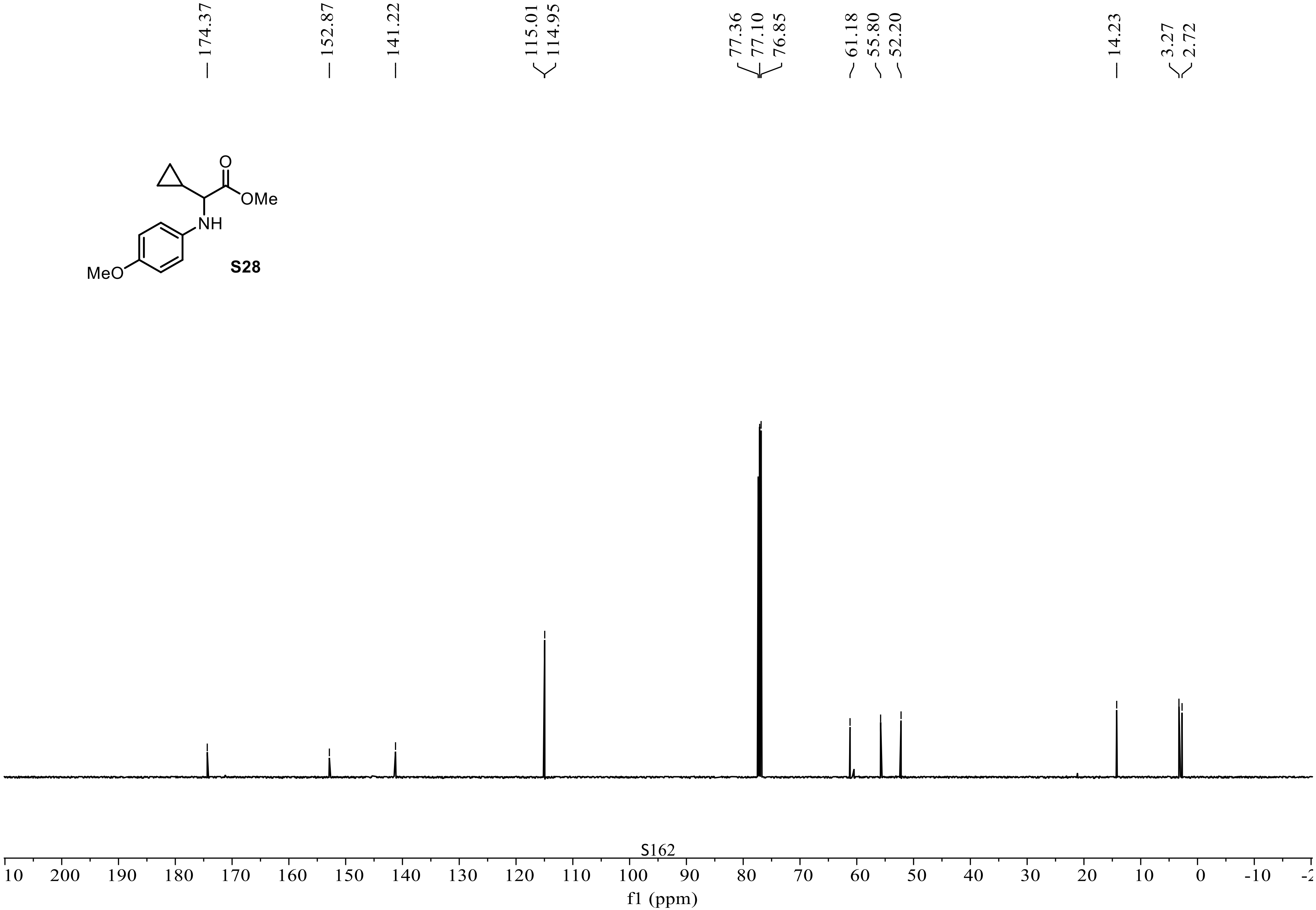
174.37
152.87
141.22
115.01
114.95
77.36
77.10
76.85
61.18
55.80
52.20
14.23
3.27
2.72
O
OMe
NH
MeO
f1 (ppm)

S28

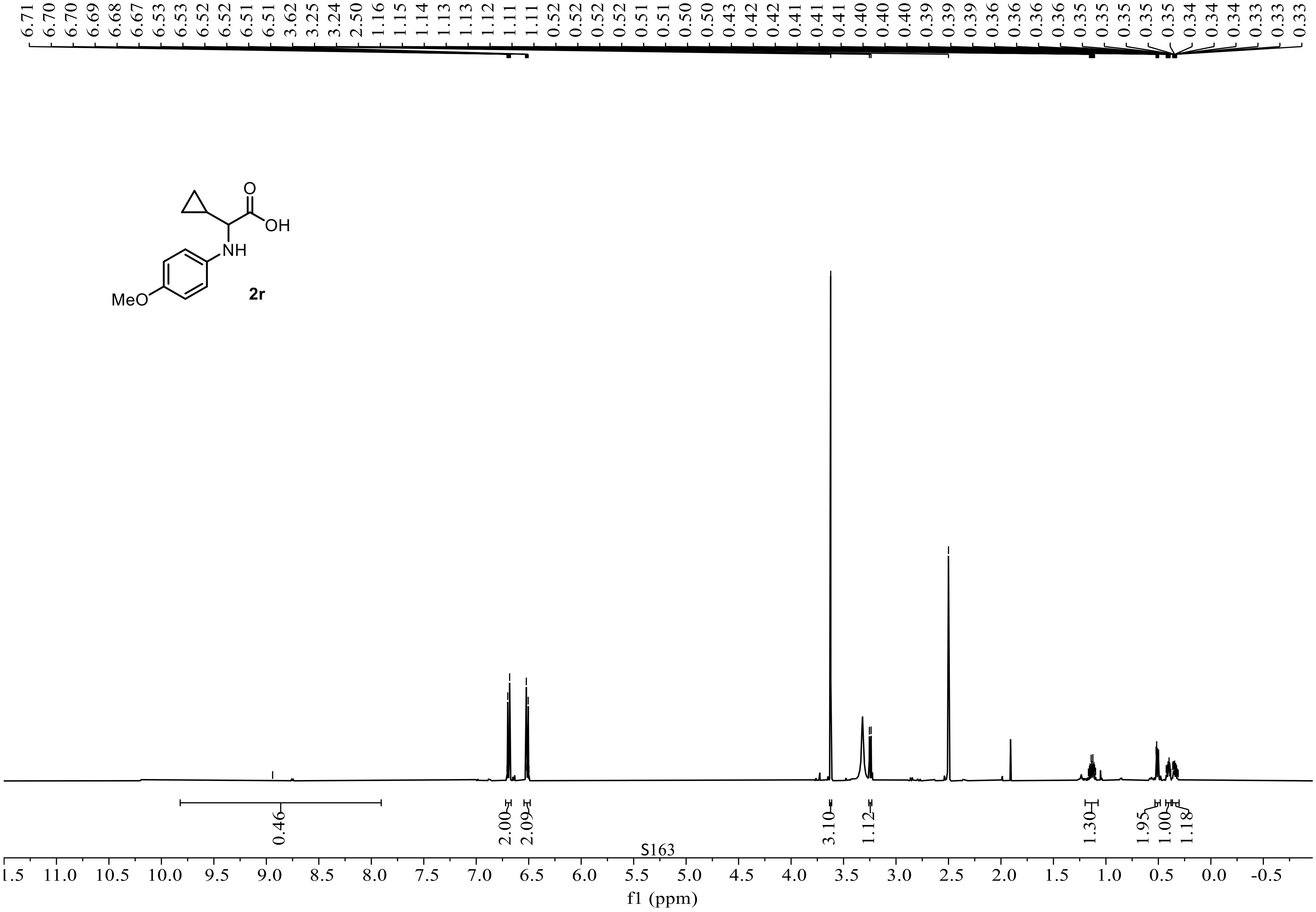
6.71 6.70 6.70 6.69 6.68 6.67 6.53 6.53 6.52 6.52 6.51 6.51 3.62 3.25 3.24 2.50 1.16 1.15 1.14 1.13 1.13 1.12 1.11 1.11 0.52 0.52 0.52 0.52 0.51 0.51 0.50 0.50 0.43 0.42 0.42 0.41 0.41 0.41 0.40 0.40 0.40 0.39 0.39 0.39 0.36 0.36 0.36 0.36 0.35 0.35 0.35 0.35 0.35 0.34 0.34 0.34 0.33 0.33 0.33
O
OH
NH
MeO
2r
0.46
2.00
2.09
3.10
1.12
1.30
1.95
1.00
1.18
1.5 11.0 10.5 10.0 9.5 9.0 8.5 8.0 7.5 7.0 6.5 6.0 5.5 5.0 4.5 4.0 3.5 3.0 2.5 2.0 1.5 1.0 0.5 0.0 -0.5
f1 (ppm)

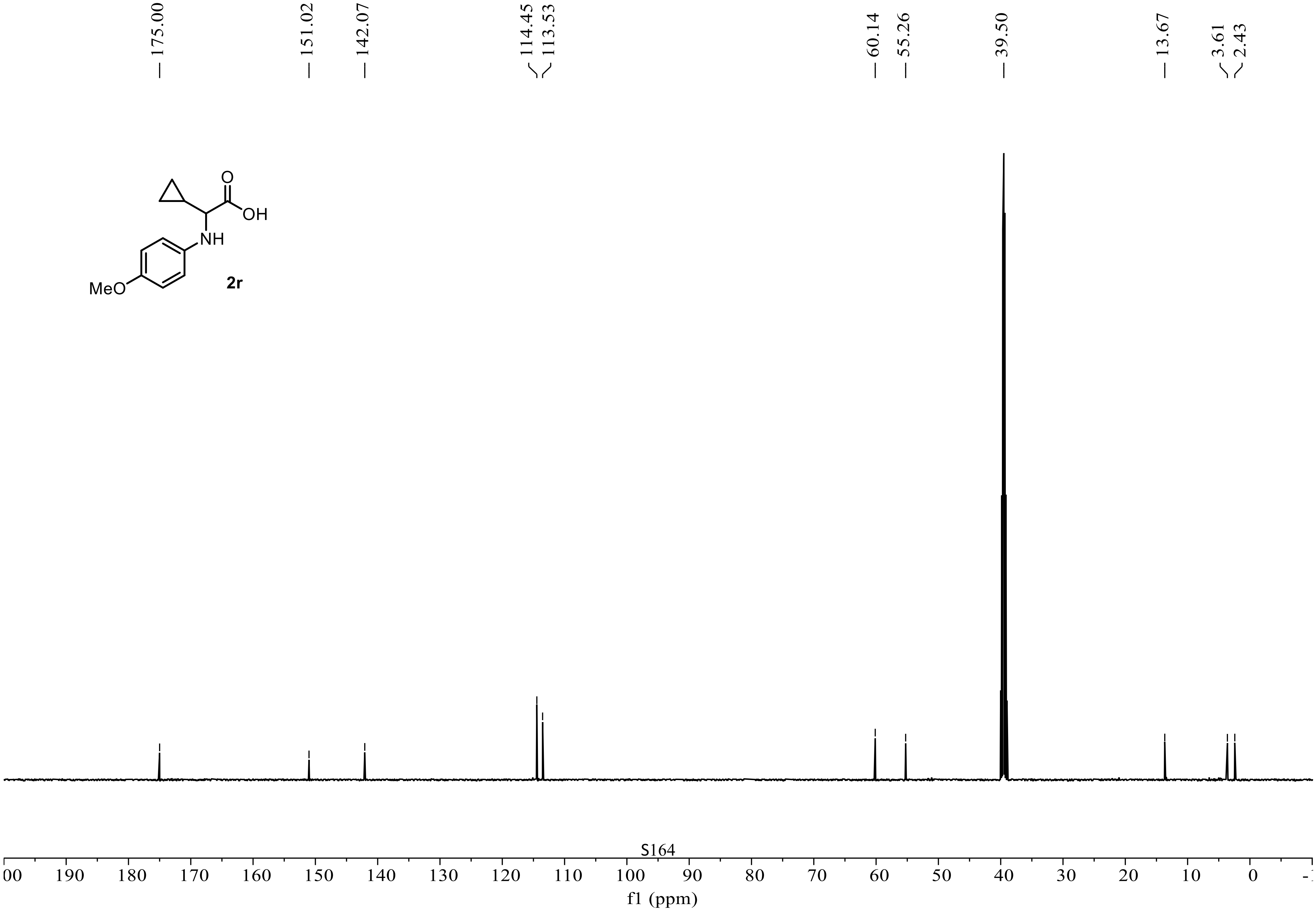
175.00
151.02
142.07
114.45
113.53
60.14
55.26
39.50
13.67
3.61
2.43
O
OH
NH
MeO
2r
00 190 180 170 160 150 140 130 120 110 100 90 80 70 60 50 40 30 20 10 0
f1 (ppm)

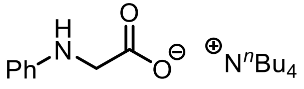

2s

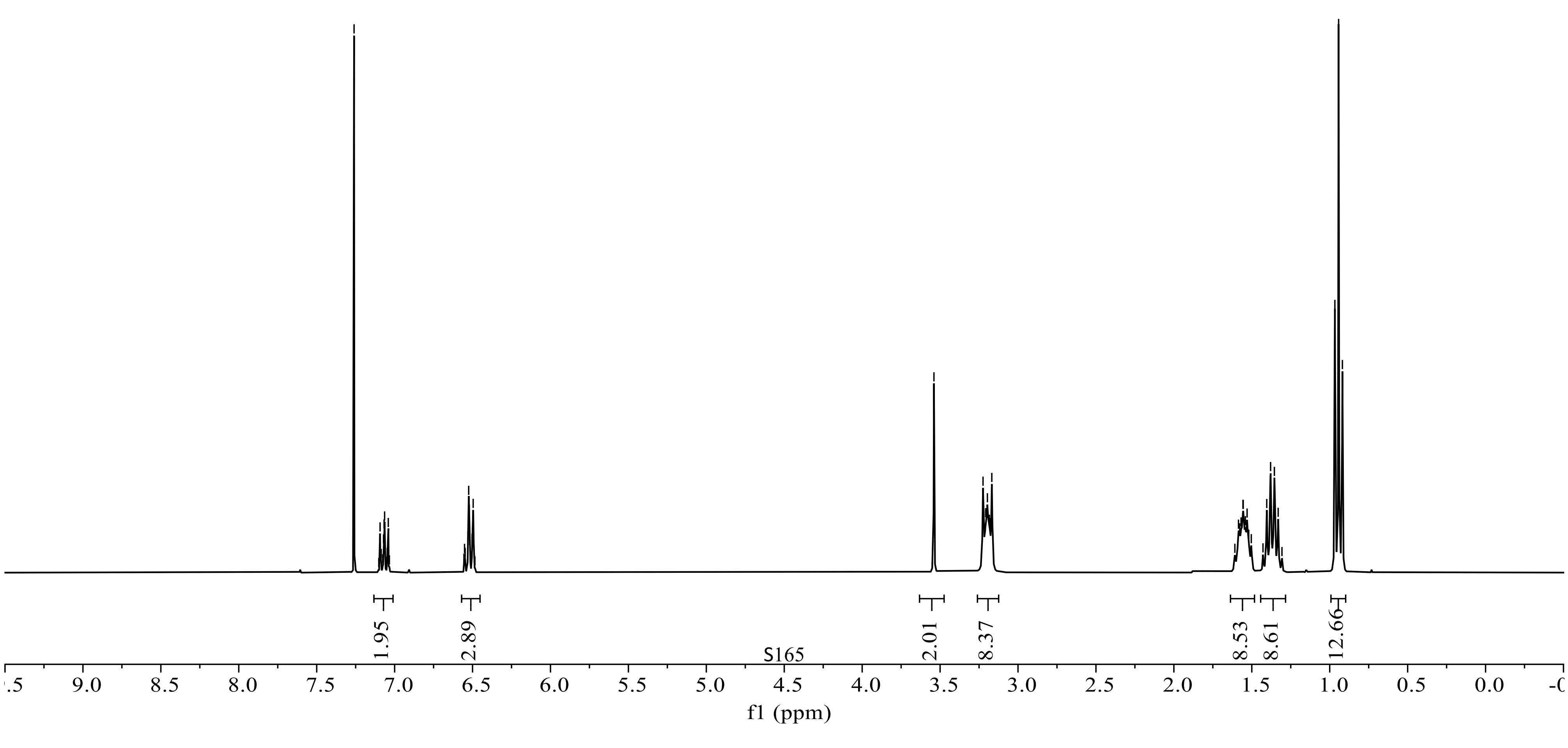
7.26 7.10 7.09 7.09 7.09 7.08 7.07 7.07 7.06 7.06 7.05 7.04 7.03 6.56 6.55 6.55 6.53 6.52 6.52 6.52 6.51 6.51 6.50 6.50 6.50 6.49 6.49 6.48
3.54 3.22 3.21 3.21 3.20 3.18 3.18 3.17 1.61 1.59 1.58 1.57 1.56 1.55 1.54 1.53 1.52 1.50 1.43 1.40 1.38 1.36 1.33 1.31 0.97 0.94 0.92
1.95
2.89
2.01
8.37
8.53
8.61
12.66
9.0 8.5 8.0 7.5 7.0 6.5 6.0 5.5 5.0 4.5 4.0 3.5 3.0 2.5 2.0 1.5 1.0 0.5 0.0
f1 (ppm)

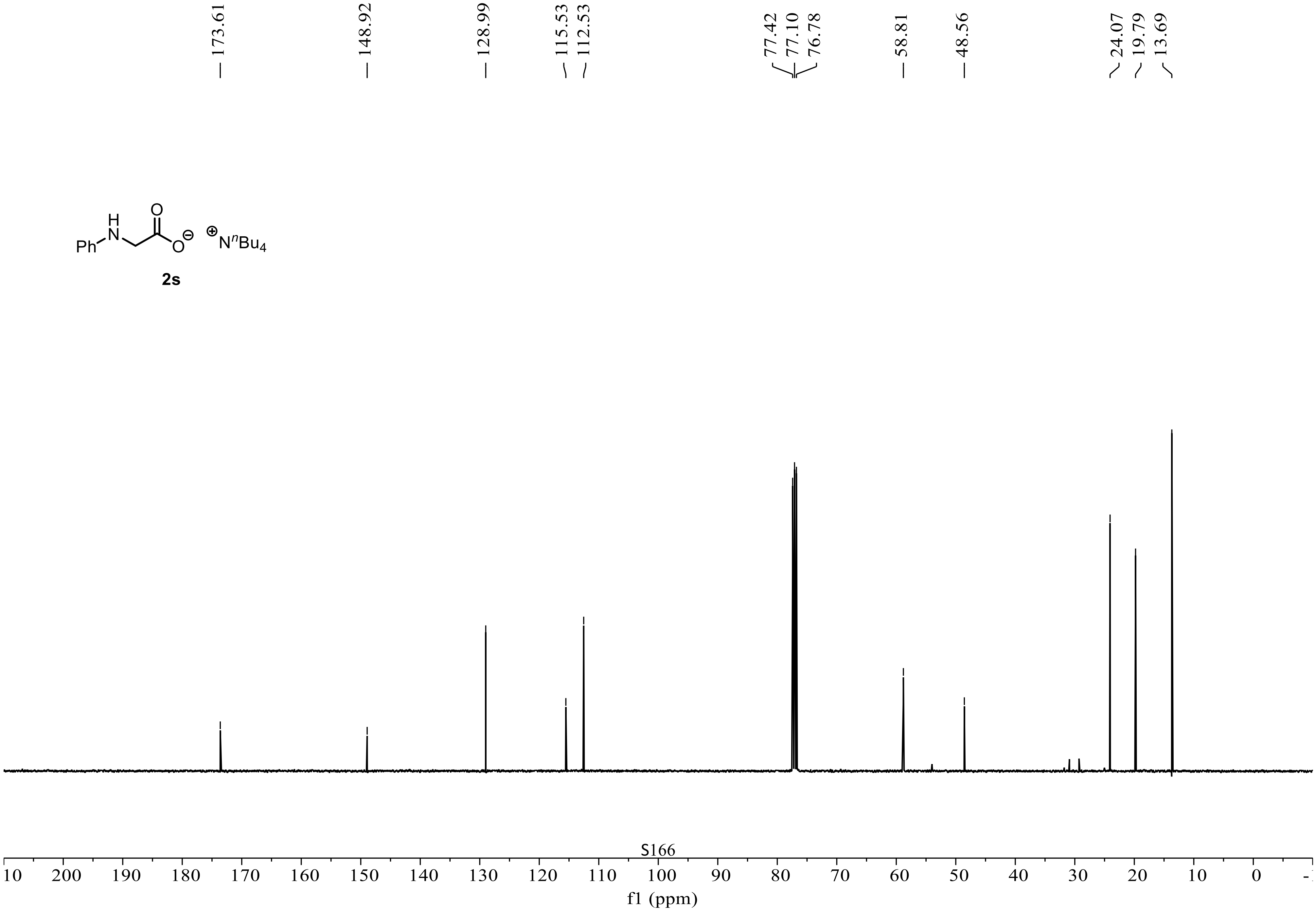

Ph
N
H
O
O
N^{n}Bu$_4$

2s

173.61
148.92
128.99
115.53
112.53
77.42
77.10
76.78
58.81
48.56
24.07
19.79
13.69
10
200
190
180
170
160
150
140
130
120
110
100
90
80
70
60
50
40
30
20
10
0
f1 (ppm)

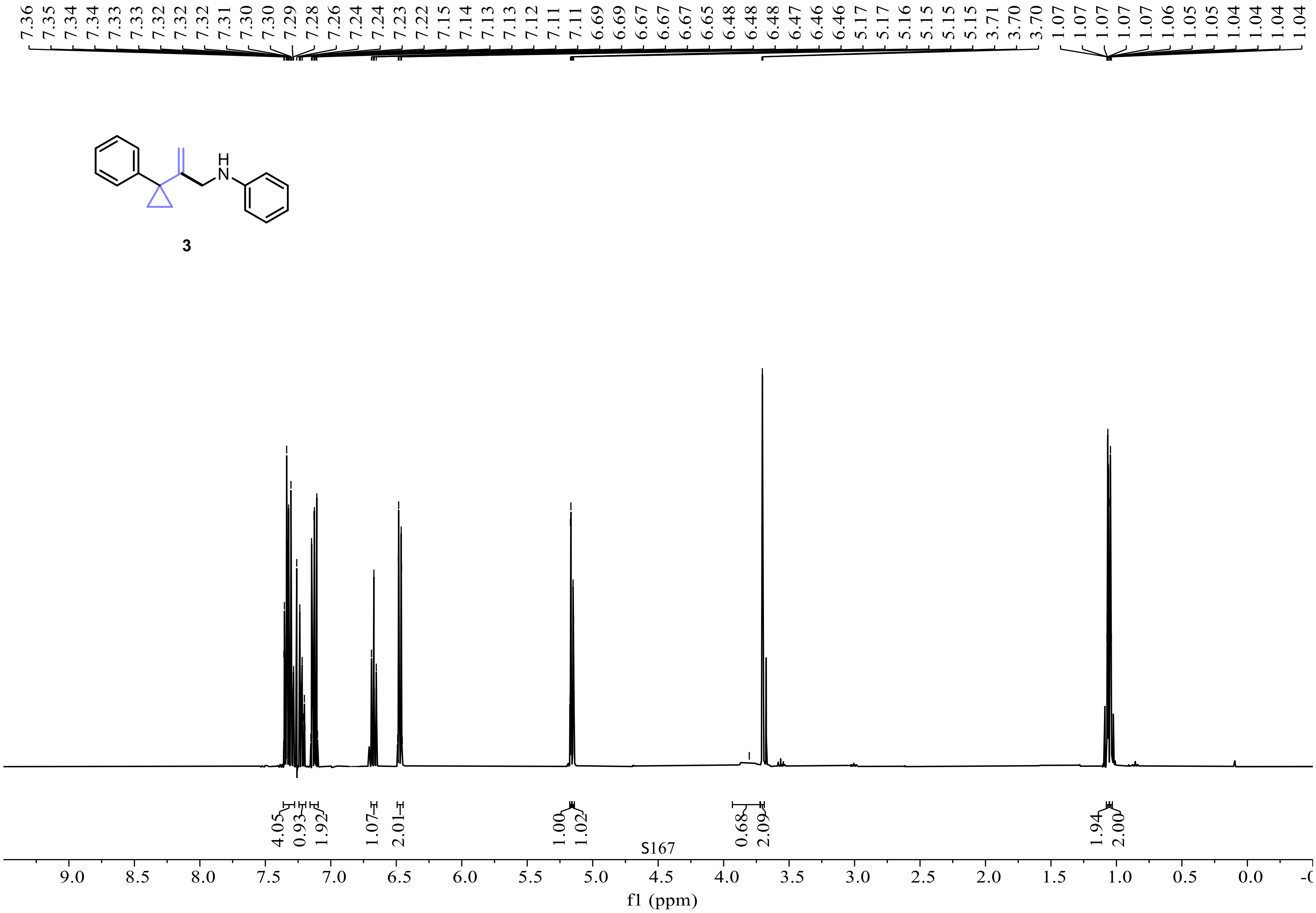
7.36 7.35 7.34 7.34 7.33 7.33 7.32 7.32 7.32 7.31 7.30 7.30 7.29 7.28 7.26 7.24 7.24 7.23 7.22 7.15 7.14 7.13 7.13 7.12 7.11 7.11 6.69 6.69 6.67 6.67 6.67 6.65 6.48 6.48 6.48 6.47 6.46 6.46 5.17 5.17 5.16 5.15 5.15 5.15 3.71 3.70 3.70 1.07 1.07 1.07 1.07 1.07 1.06 1.05 1.05 1.04 1.04 1.04 1.04
H
N
3
4.05 0.93 1.92 1.07 2.01 1.00 1.02 0.68 2.09 1.94 2.00
9.0 8.5 8.0 7.5 7.0 6.5 6.0 5.5 5.0 4.5 4.0 3.5 3.0 2.5 2.0 1.5 1.0 0.5 0.0
f1 (ppm)

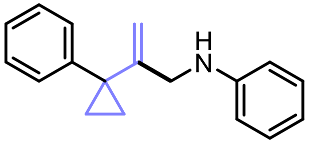

3

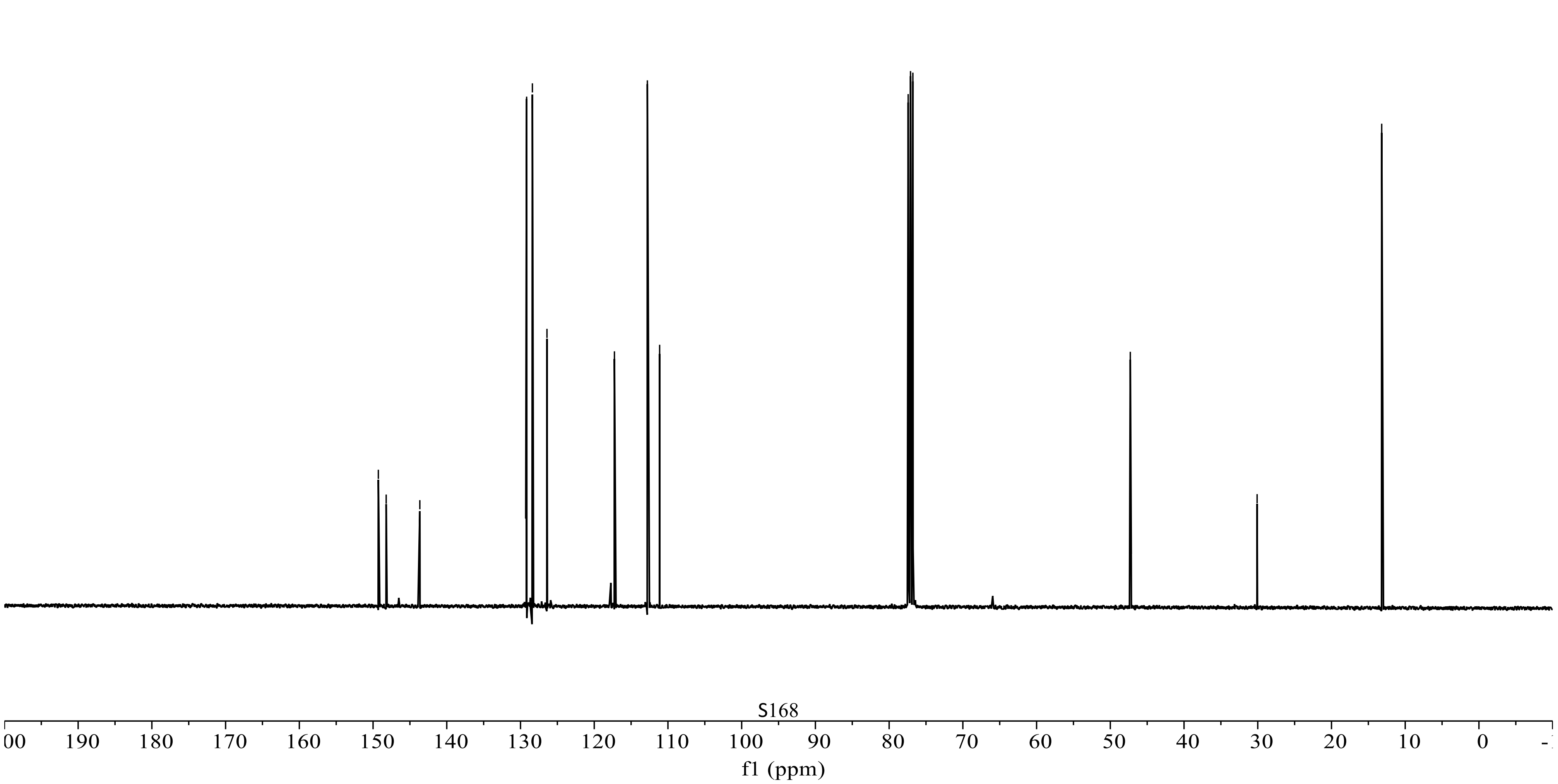
149.28
148.22
143.65
129.17
128.41
128.39
126.41
117.26
112.79
111.13
77.41
77.10
76.78
47.29
30.09
13.19
f1 (ppm)

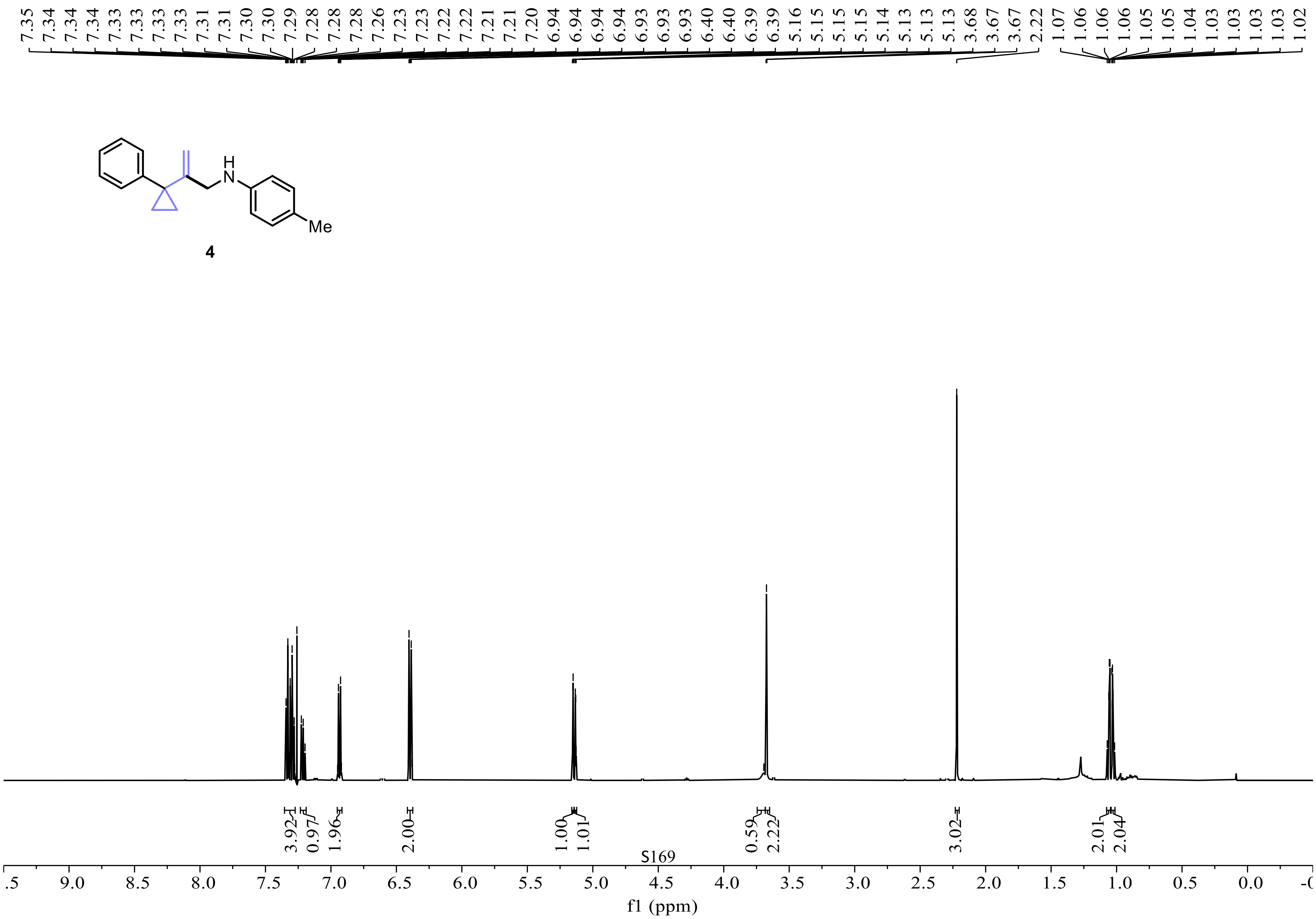
7.35 7.34 7.34 7.34 7.33 7.33 7.33 7.33 7.31 7.31 7.30 7.30 7.29 7.28 7.28 7.28 7.26 7.23 7.23 7.22 7.22 7.21 7.21 7.20 6.94 6.94 6.94 6.93 6.93 6.93 6.40 6.40 6.39 6.39 5.16 5.15 5.15 5.15 5.14 5.13 5.13 5.13 3.68 3.67 3.67 2.22 1.07 1.06 1.06 1.06 1.05 1.05 1.04 1.03 1.03 1.03 1.03 1.02
H
N
Me
4
3.92 0.97 1.96 2.00 1.00 1.01 0.59 2.22 3.02 2.01 2.04
f1 (ppm)

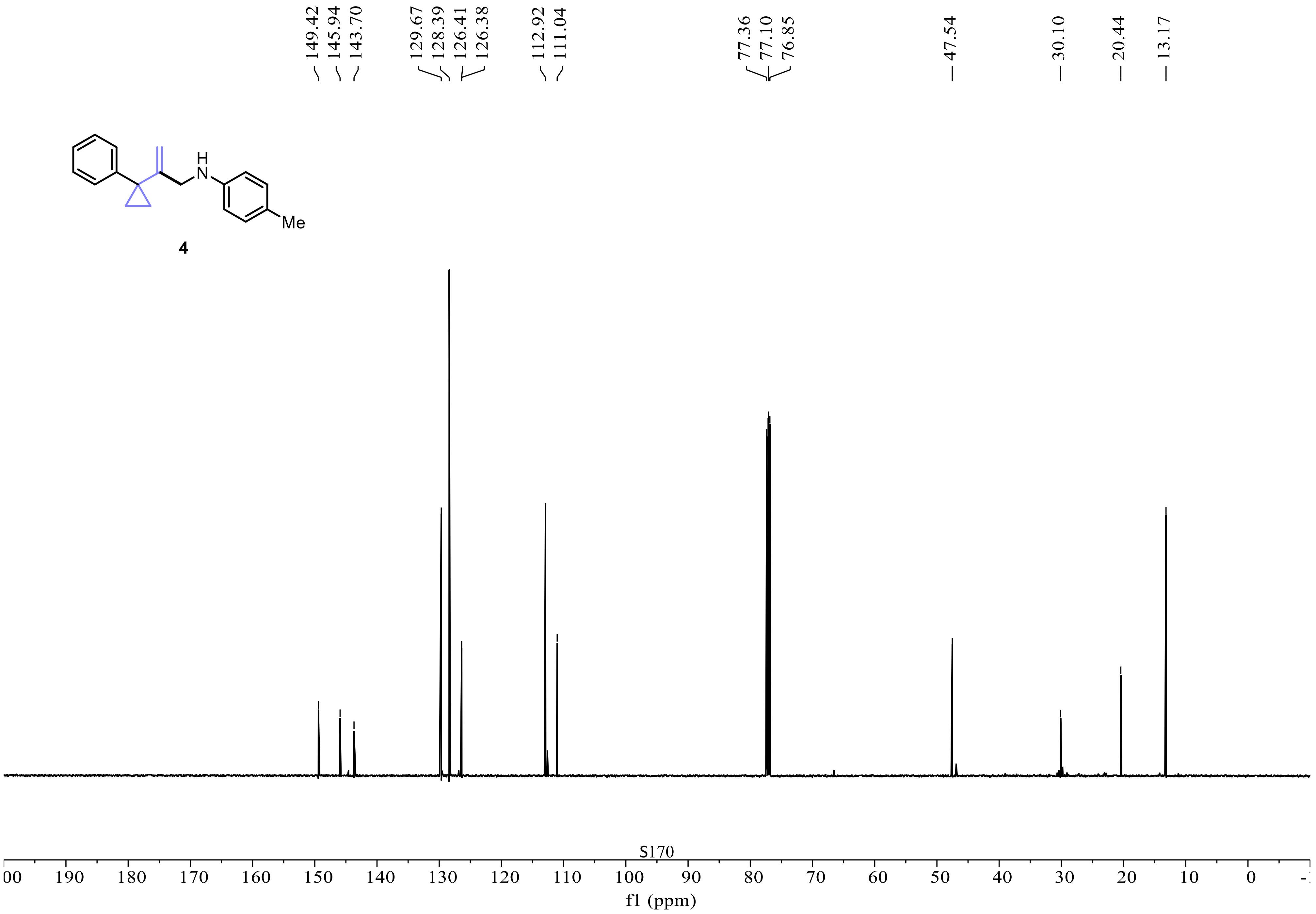
149.42
145.94
143.70
129.67
128.39
126.41
126.38
112.92
111.04
77.36
77.10
76.85
47.54
30.10
20.44
13.17
H
N
Me
4
f1 (ppm)

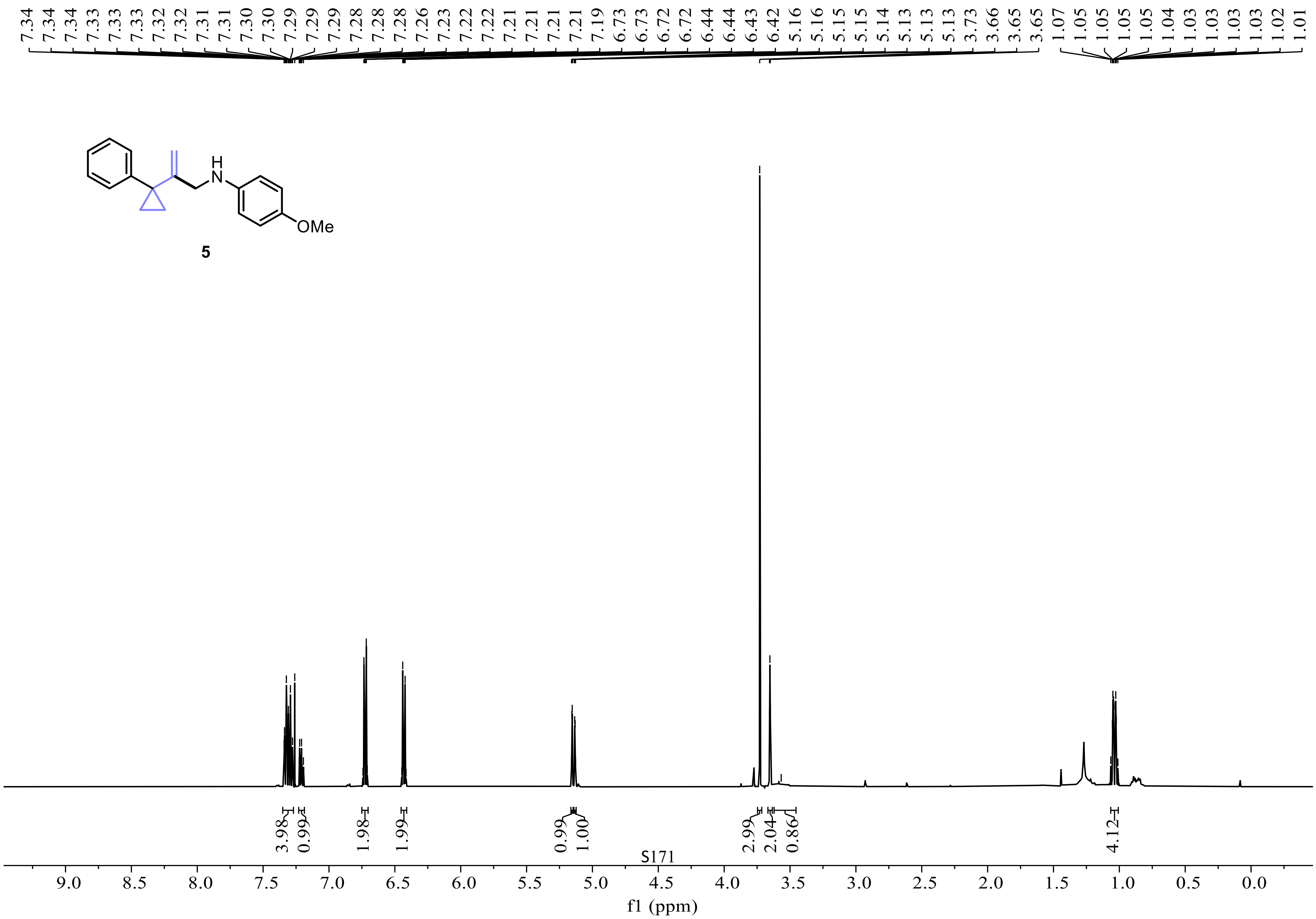
7.34 7.34 7.34 7.33 7.33 7.33 7.32 7.32 7.31 7.31 7.30 7.30 7.29 7.29 7.29 7.28 7.28 7.28 7.26 7.23 7.22 7.22 7.21 7.21 7.21 7.21 7.19 6.73 6.73 6.72 6.72 6.44 6.44 6.43 6.42 5.16 5.16 5.15 5.15 5.14 5.13 5.13 5.13 3.73 3.66 3.65 3.65 1.07 1.05 1.05 1.05 1.05 1.04 1.03 1.03 1.03 1.03 1.02 1.01
H
N
OMe
5
3.98
0.99
1.98
1.99
0.99
1.00
2.99
2.04
0.86
4.12
9.0 8.5 8.0 7.5 7.0 6.5 6.0 5.5 5.0 4.5 4.0 3.5 3.0 2.5 2.0 1.5 1.0 0.5 0.0
f1 (ppm)

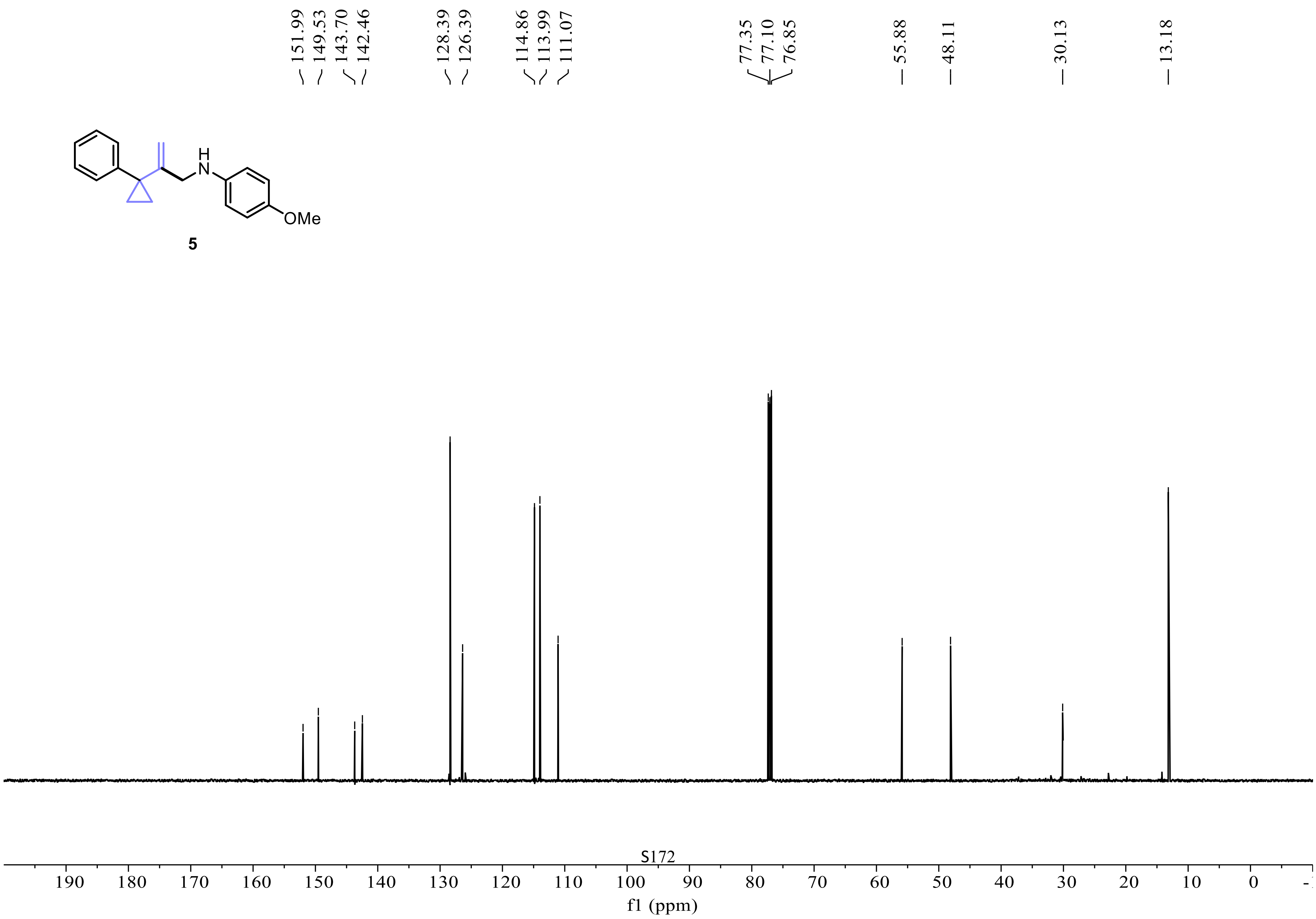

OMe
151.99
149.53
143.70
142.46
128.39
126.39
114.86
113.99
111.07
77.35
77.10
76.85
55.88
48.11
30.13
13.18
190 180 170 160 150 140 130 120 110 100 90 80 70 60 50 40 30 20 10 0
f1 (ppm)

5

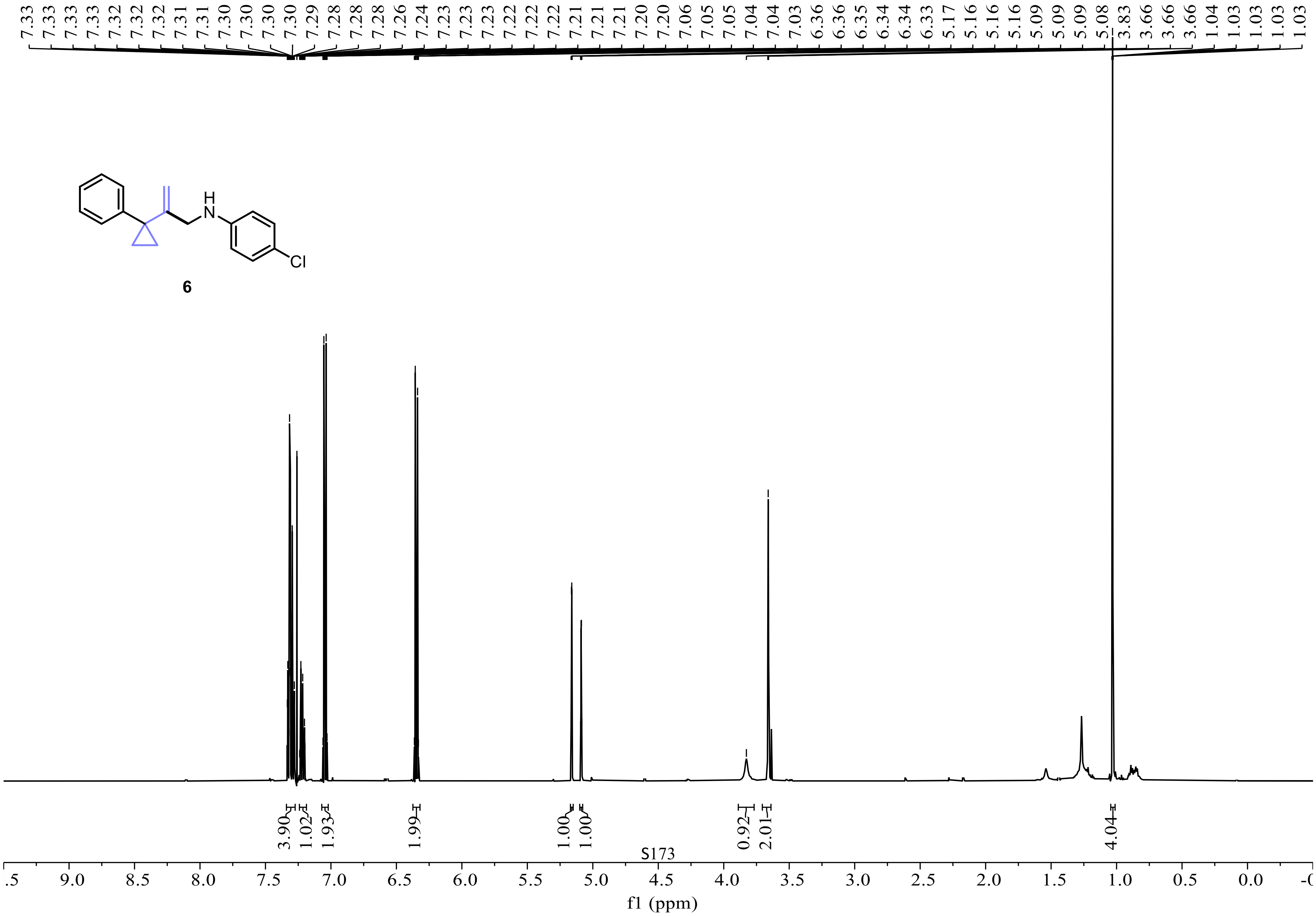
7.33 7.33 7.33 7.33 7.32 7.32 7.32 7.31 7.31 7.30 7.30 7.30 7.30 7.29 7.28 7.28 7.28 7.26 7.24 7.23 7.23 7.23 7.22 7.22 7.22 7.21 7.21 7.21 7.20 7.20 7.06 7.05 7.05 7.04 7.04 7.03 6.36 6.36 6.35 6.34 6.34 6.33 5.17 5.16 5.16 5.16 5.09 5.09 5.09 5.08 3.83 3.66 3.66 3.66 1.04 1.03 1.03 1.03 1.03
H
N
Cl
6
3.90
1.02
1.93
1.99
1.00
1.00
0.92
2.01
4.04
.5 9.0 8.5 8.0 7.5 7.0 6.5 6.0 5.5 5.0 4.5 4.0 3.5 3.0 2.5 2.0 1.5 1.0 0.5 0.0 -(
f1 (ppm)

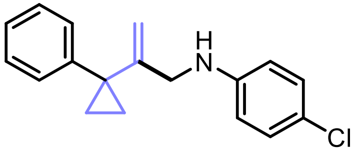
H
N
Cl

6

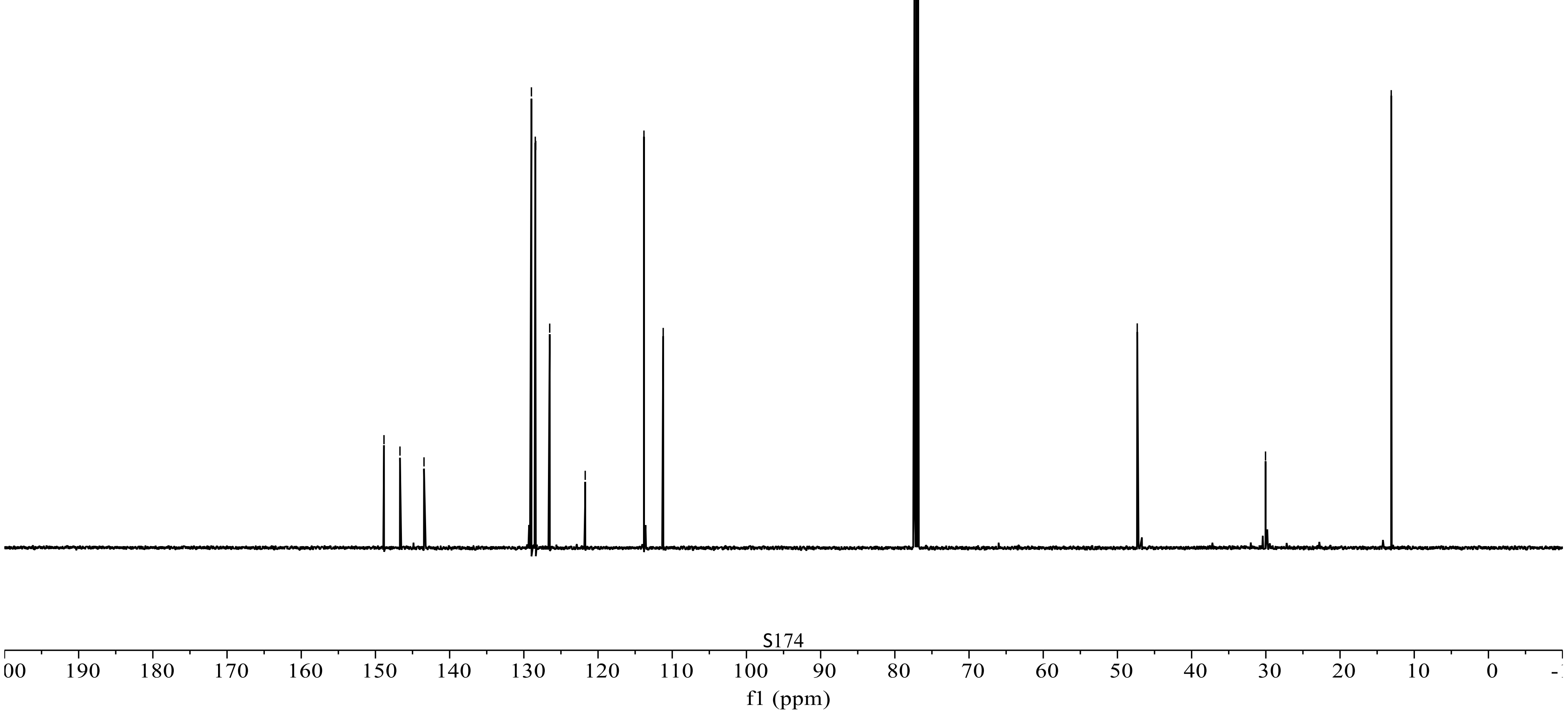
148.85
146.70
143.45
128.98
128.46
128.40
126.51
121.73
113.81
111.22
77.36
77.10
76.85
47.33
30.04
13.10
00
190
180
170
160
150
140
130
120
110
100
90
80
70
60
50
40
30
20
10
0
f1 (ppm)

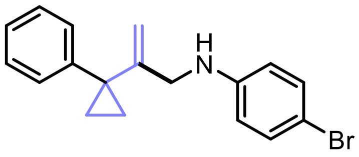

7

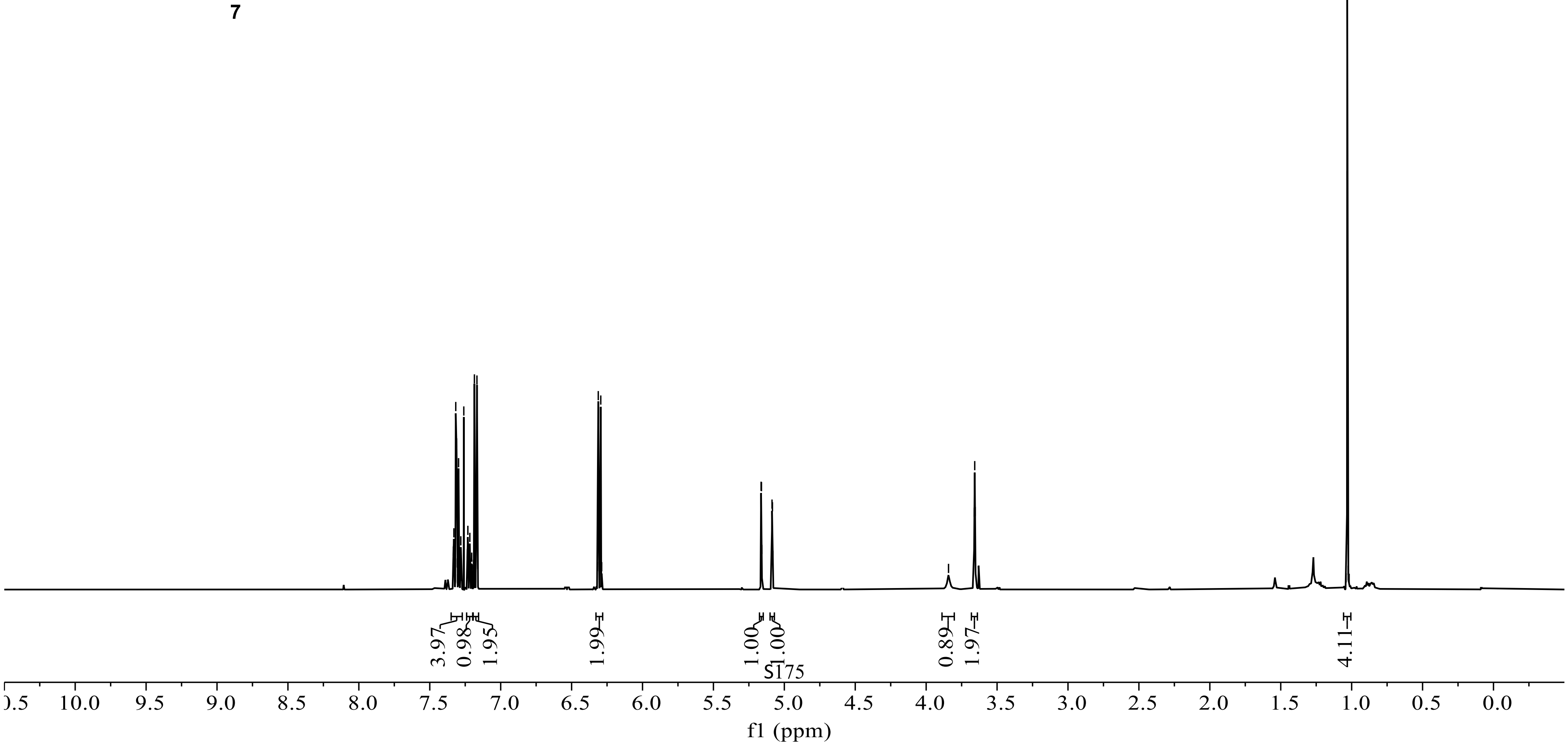

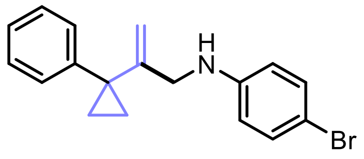
H
N
Br

7

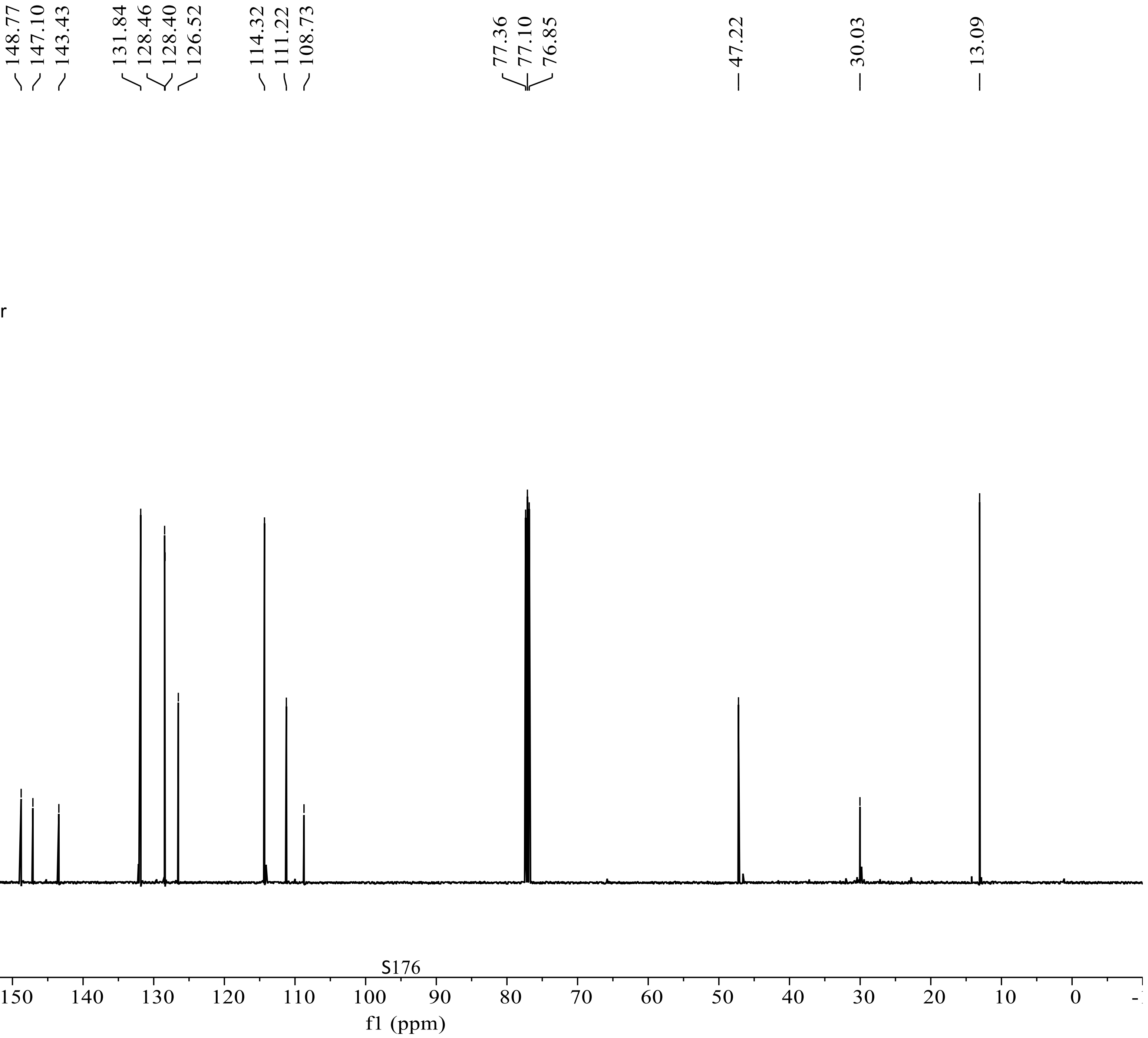
148.77
147.10
143.43
131.84
128.46
128.40
126.52
114.32
111.22
108.73
77.36
77.10
76.85
47.22
30.03
13.09
190
180
170
160
150
140
130
120
110
100
90
80
70
60
50
40
30
20
10
0
f1 (ppm)

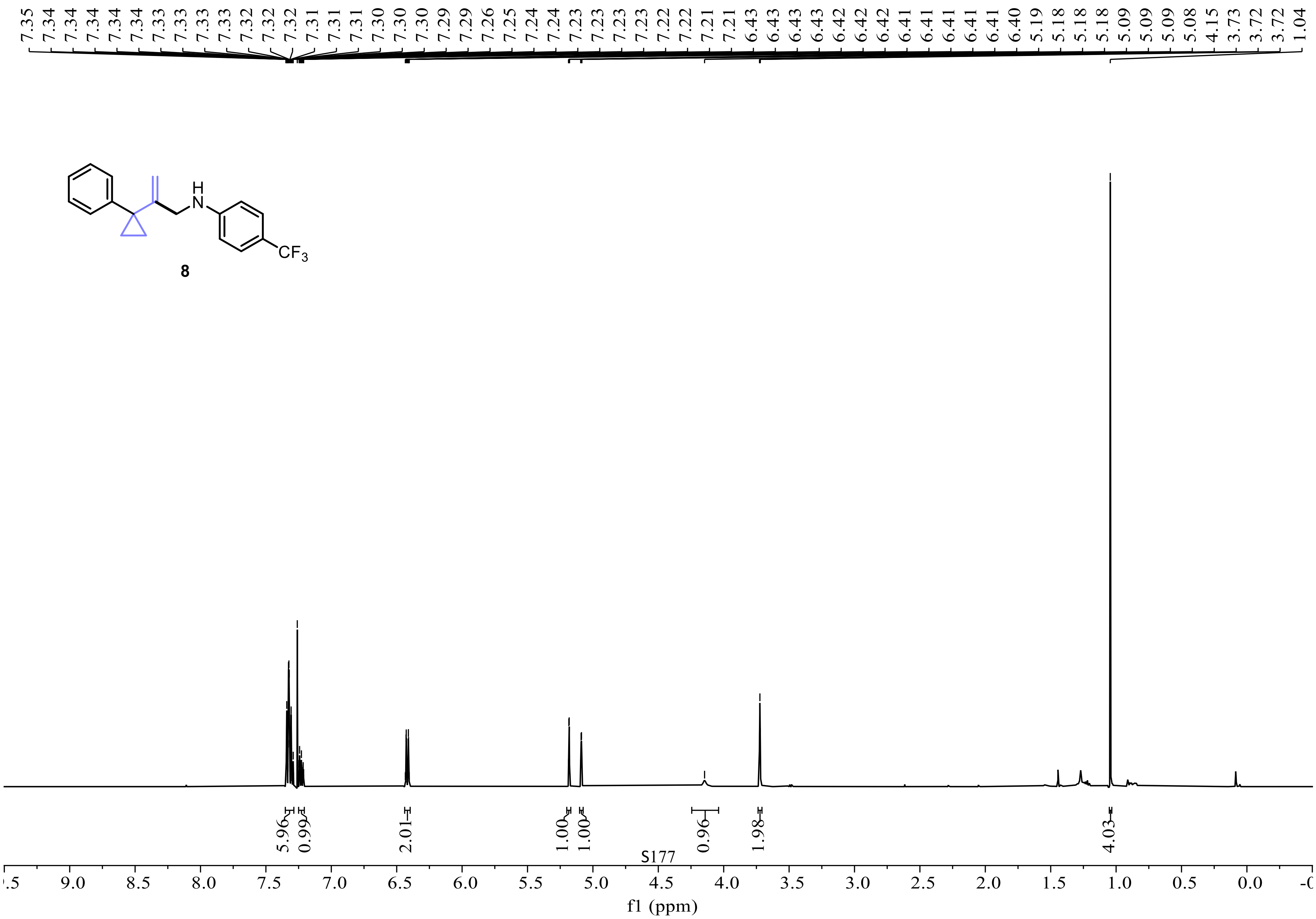

7.35 7.34 7.34 7.34 7.34 7.34 7.33 7.33 7.33 7.33 7.32 7.32 7.32 7.31 7.31 7.31 7.30 7.30 7.30 7.29 7.29 7.26 7.25 7.24 7.24 7.23 7.23 7.23 7.23 7.22 7.22 7.21 7.21 6.43 6.43 6.43 6.43 6.42 6.42 6.42 6.41 6.41 6.41 6.41 6.41 6.40 5.19 5.18 5.18 5.18 5.09 5.09 5.09 5.08 4.15 3.73 3.72 3.72 1.04
H
N
CF3
8
5.96
0.99
2.01
1.00
1.00
0.96
1.98
4.03
9.0 8.5 8.0 7.5 7.0 6.5 6.0 5.5 5.0 4.5 4.0 3.5 3.0 2.5 2.0 1.5 1.0 0.5 0.0
f1 (ppm)

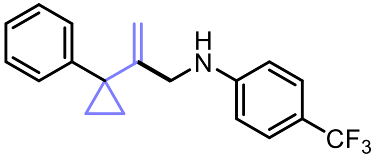
H
N
CF3

8

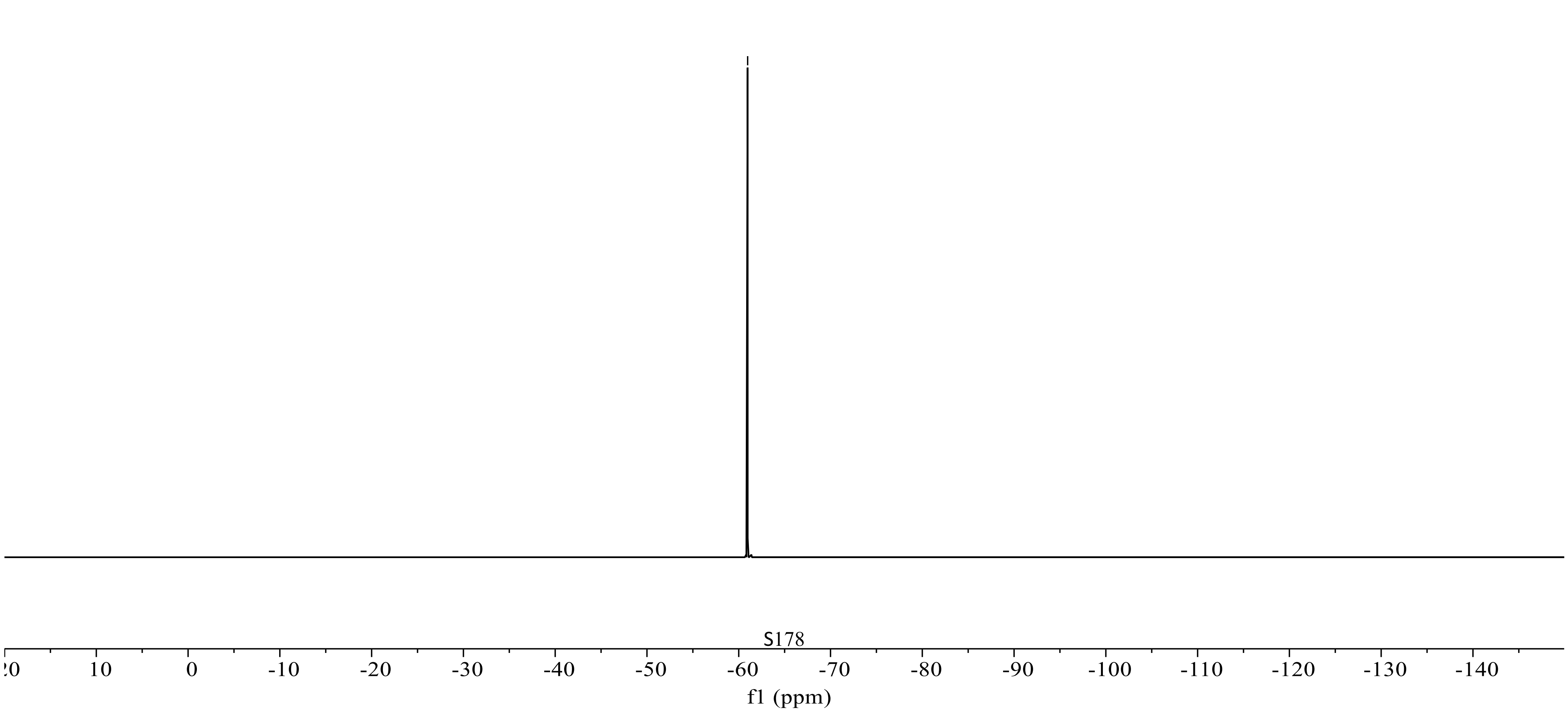
-60.97
20
10
0
-10
-20
-30
-40
-50
-60
-70
-80
-90
-100
-110
-120
-130
-140
f1 (ppm)

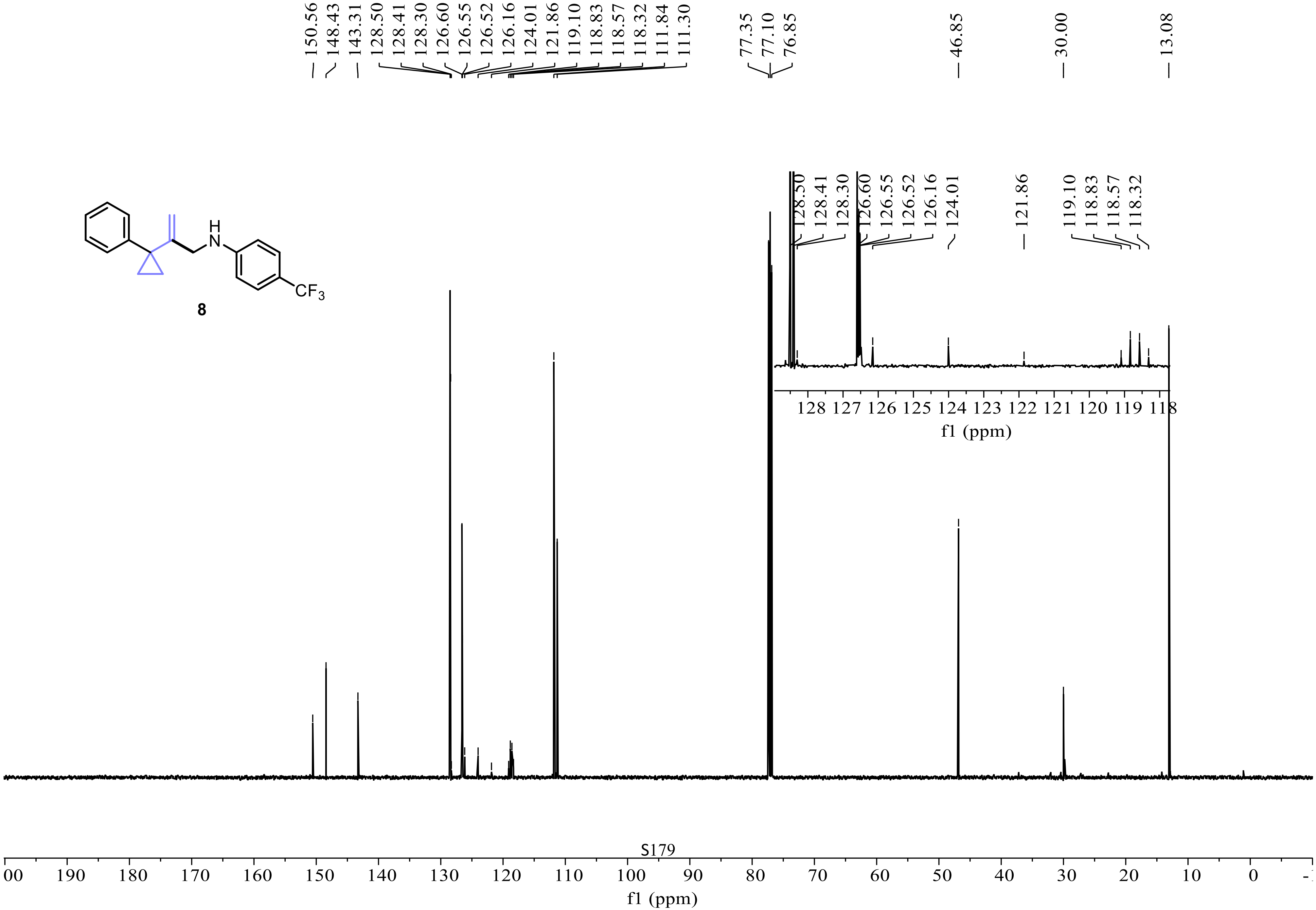
150.56
148.43
143.31
128.50
128.41
128.30
126.60
126.55
126.52
126.16
124.01
121.86
119.10
118.83
118.57
118.32
111.84
111.30
77.35
77.10
76.85
46.85
30.00
13.08
H
N
CF3
8
128 127 126 125 124 123 122 121 120 119 118
f1 (ppm)
00 190 180 170 160 150 140 130 120 110 100 90 80 70 60 50 40 30 20 10 0
f1 (ppm)

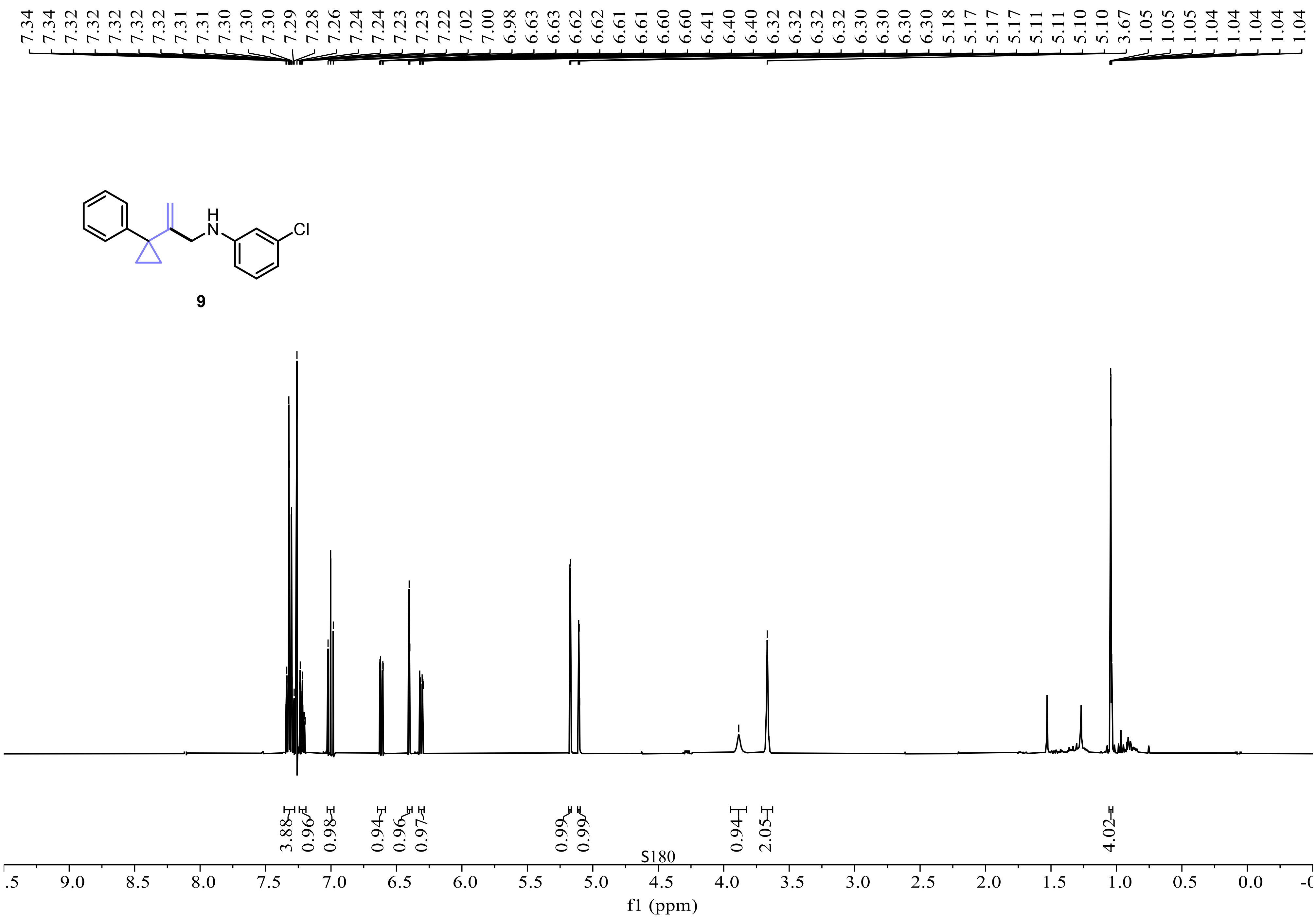
7.34 7.34 7.32 7.32 7.32 7.32 7.32 7.31 7.31 7.30 7.30 7.30 7.29 7.28 7.26 7.24 7.24 7.23 7.23 7.22 7.02 7.00 6.98 6.63 6.63 6.62 6.62 6.61 6.61 6.60 6.60 6.41 6.40 6.40 6.32 6.32 6.32 6.32 6.30 6.30 6.30 6.30 5.18 5.17 5.17 5.17 5.11 5.11 5.10 5.10 3.67 1.05 1.05 1.05 1.04 1.04 1.04 1.04 1.04
H
N
Cl
9
3.88
0.96
0.98
0.94
0.96
0.97
0.99
0.99
0.94
2.05
4.02
f1 (ppm)

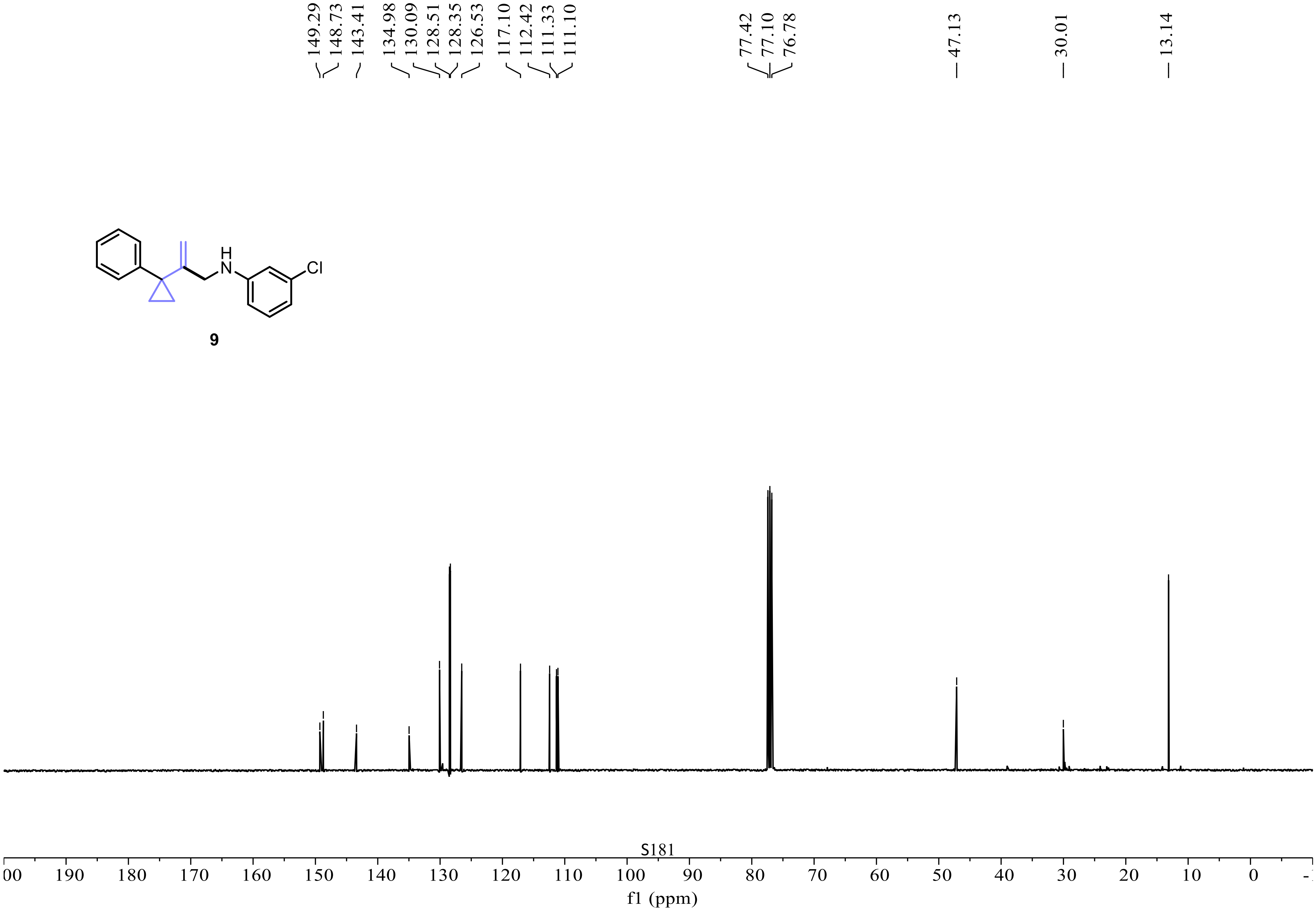
149.29
148.73
143.41
134.98
130.09
128.51
128.35
126.53
117.10
112.42
111.33
111.10
77.42
77.10
76.78
47.13
30.01
13.14
H
N
Cl
9
f1 (ppm)

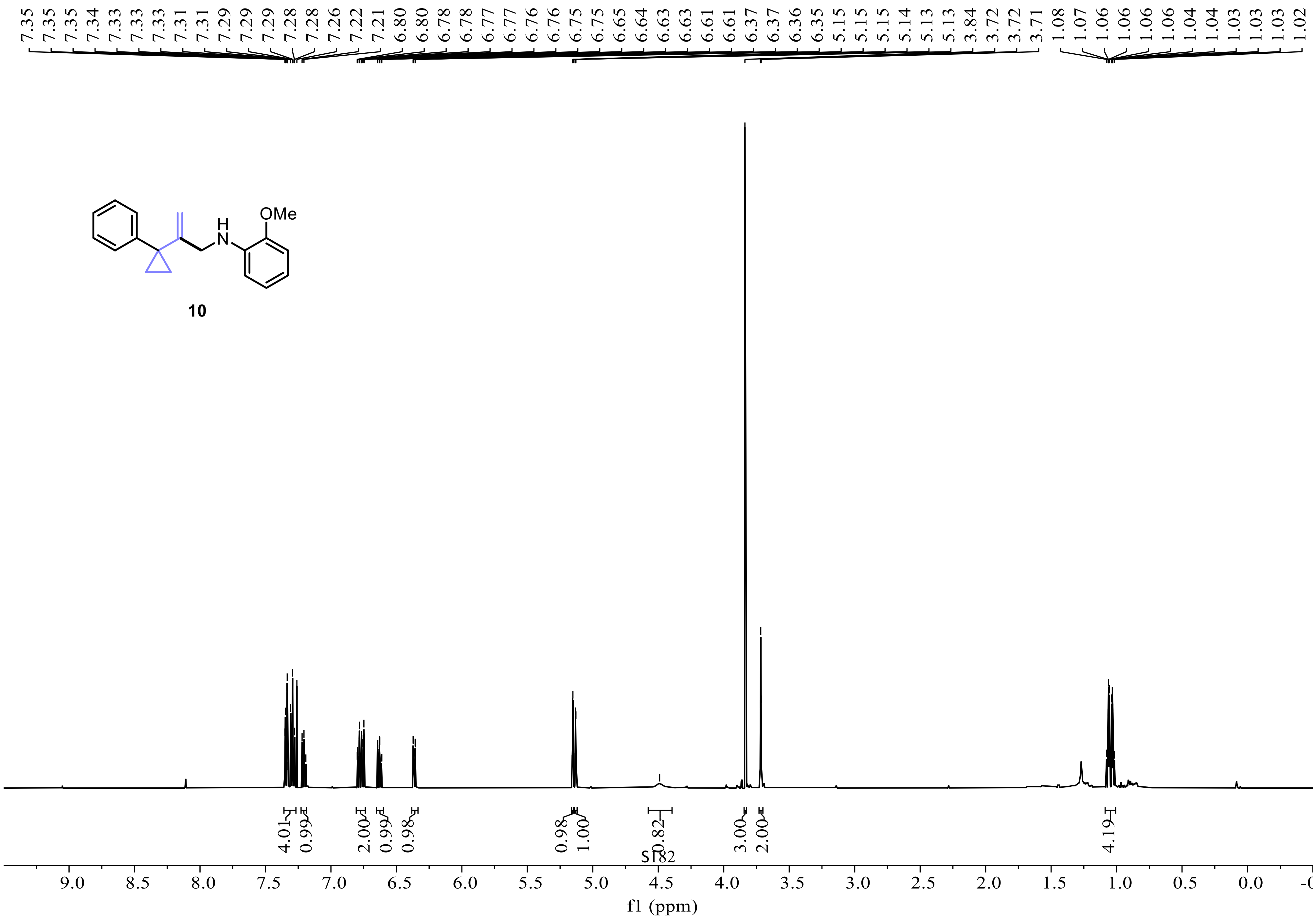
7.35 7.35 7.35 7.34 7.33 7.33 7.33 7.31 7.31 7.29 7.29 7.29 7.28 7.28 7.26 7.22 7.21 6.80 6.80 6.78 6.78 6.77 6.77 6.76 6.76 6.75 6.75 6.65 6.64 6.63 6.63 6.61 6.61 6.37 6.37 6.36 6.35 5.15 5.15 5.15 5.14 5.13 5.13 3.84 3.72 3.72 3.71 1.08 1.07 1.06 1.06 1.06 1.06 1.04 1.04 1.03 1.03 1.03 1.02
OMe
H
N
10
4.01
0.99
2.00
0.99
0.98
0.98
1.00
0.82
3.00
2.00
4.19
9.0 8.5 8.0 7.5 7.0 6.5 6.0 5.5 5.0 4.5 4.0 3.5 3.0 2.5 2.0 1.5 1.0 0.5 0.0
f1 (ppm)

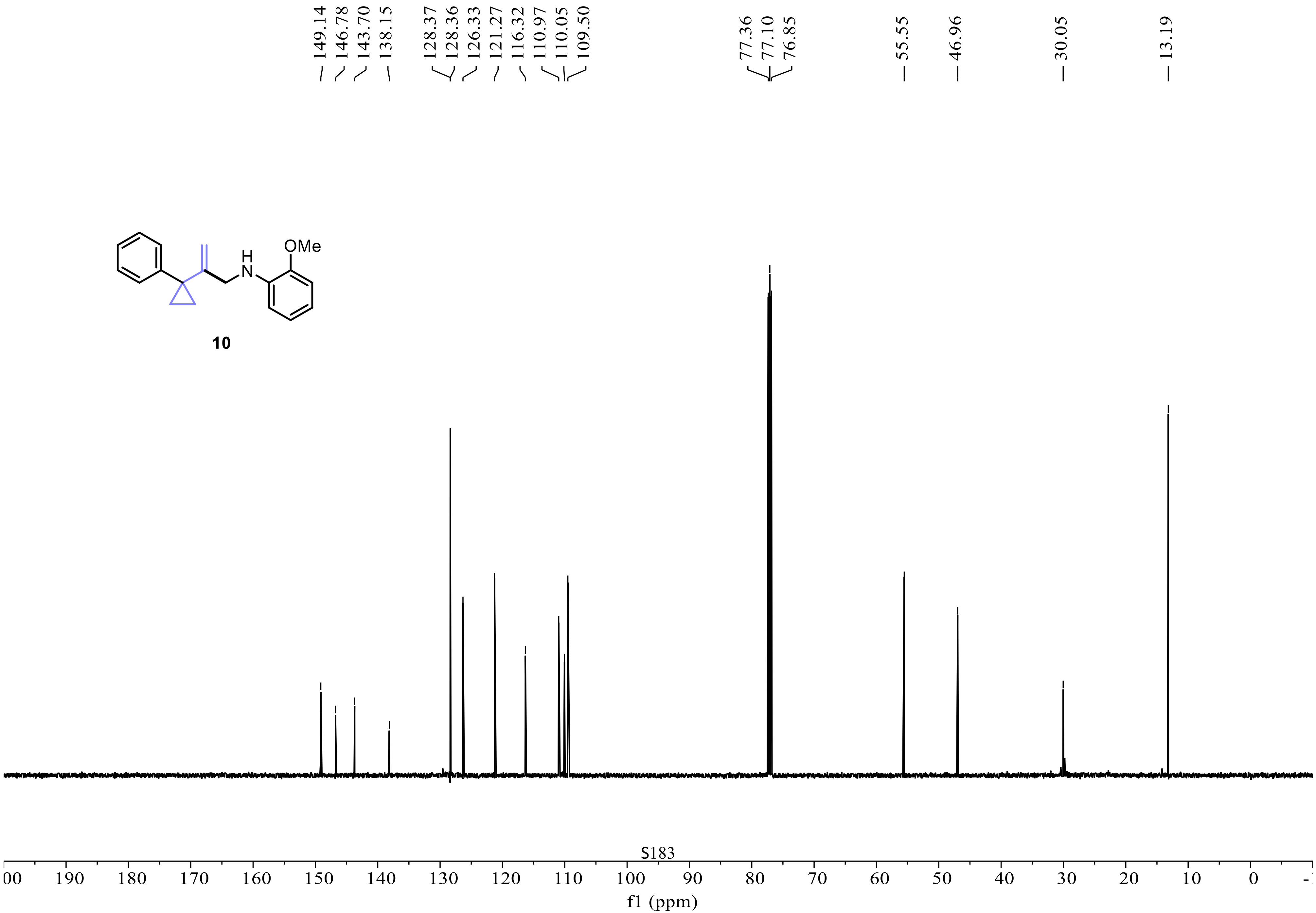

149.14
146.78
143.70
138.15
128.37
128.36
126.33
121.27
116.32
110.97
110.05
109.50
77.36
77.10
76.85
55.55
46.96
30.05
13.19
OMe
H
N
10
f1 (ppm)

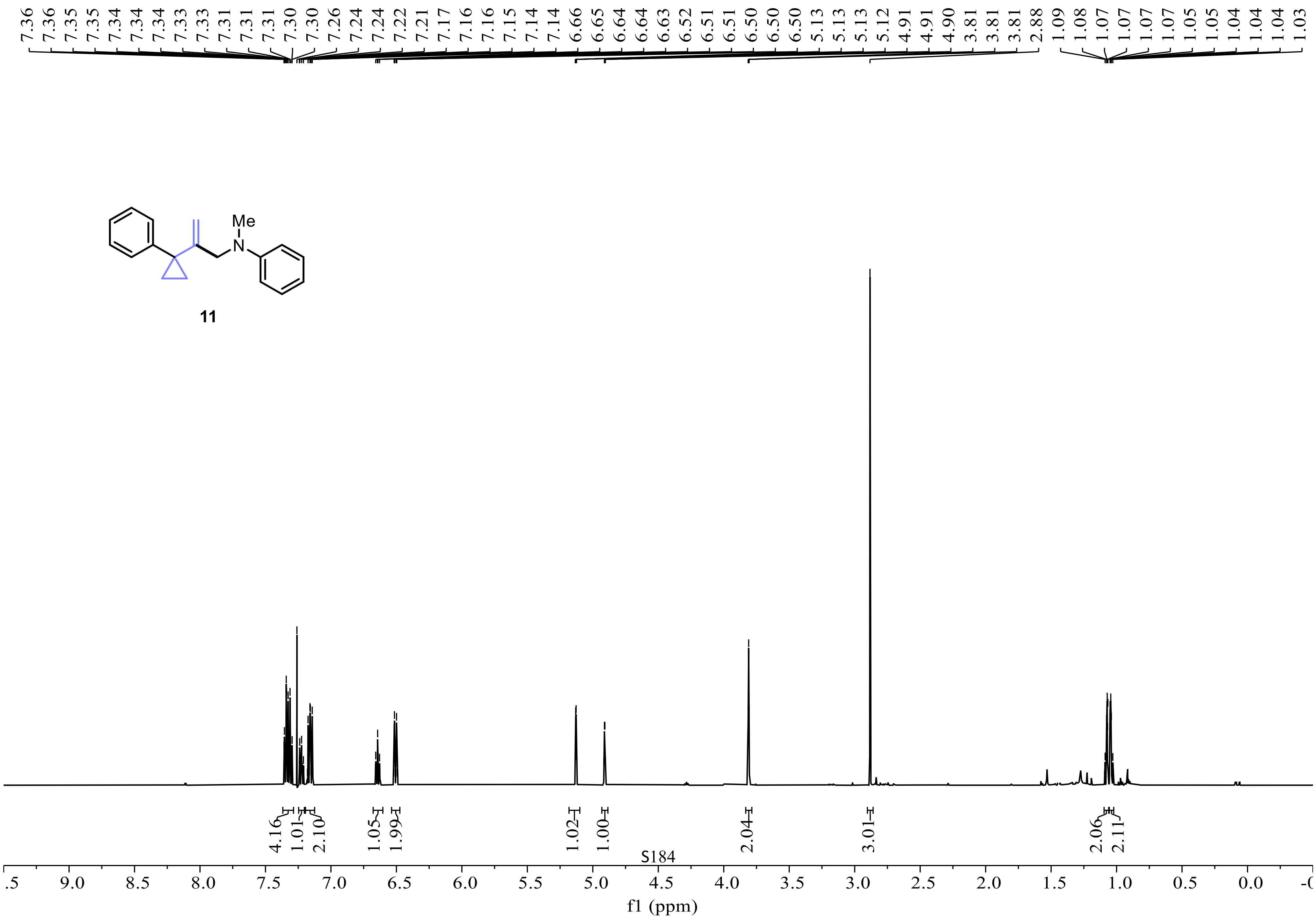
7.36 7.36 7.35 7.35 7.34 7.34 7.34 7.33 7.33 7.31 7.31 7.31 7.30 7.30 7.26 7.24 7.24 7.22 7.21 7.17 7.16 7.16 7.15 7.14 7.14 6.66 6.65 6.64 6.64 6.63 6.52 6.51 6.51 6.50 6.50 6.50 5.13 5.13 5.13 5.12 4.91 4.91 4.90 3.81 3.81 3.81 2.88 1.09 1.08 1.07 1.07 1.07 1.07 1.05 1.05 1.04 1.04 1.04 1.03
Me
N
11
4.16
1.01
2.10
1.05
1.99
1.02
1.00
2.04
3.01
2.06
2.11
f1 (ppm)

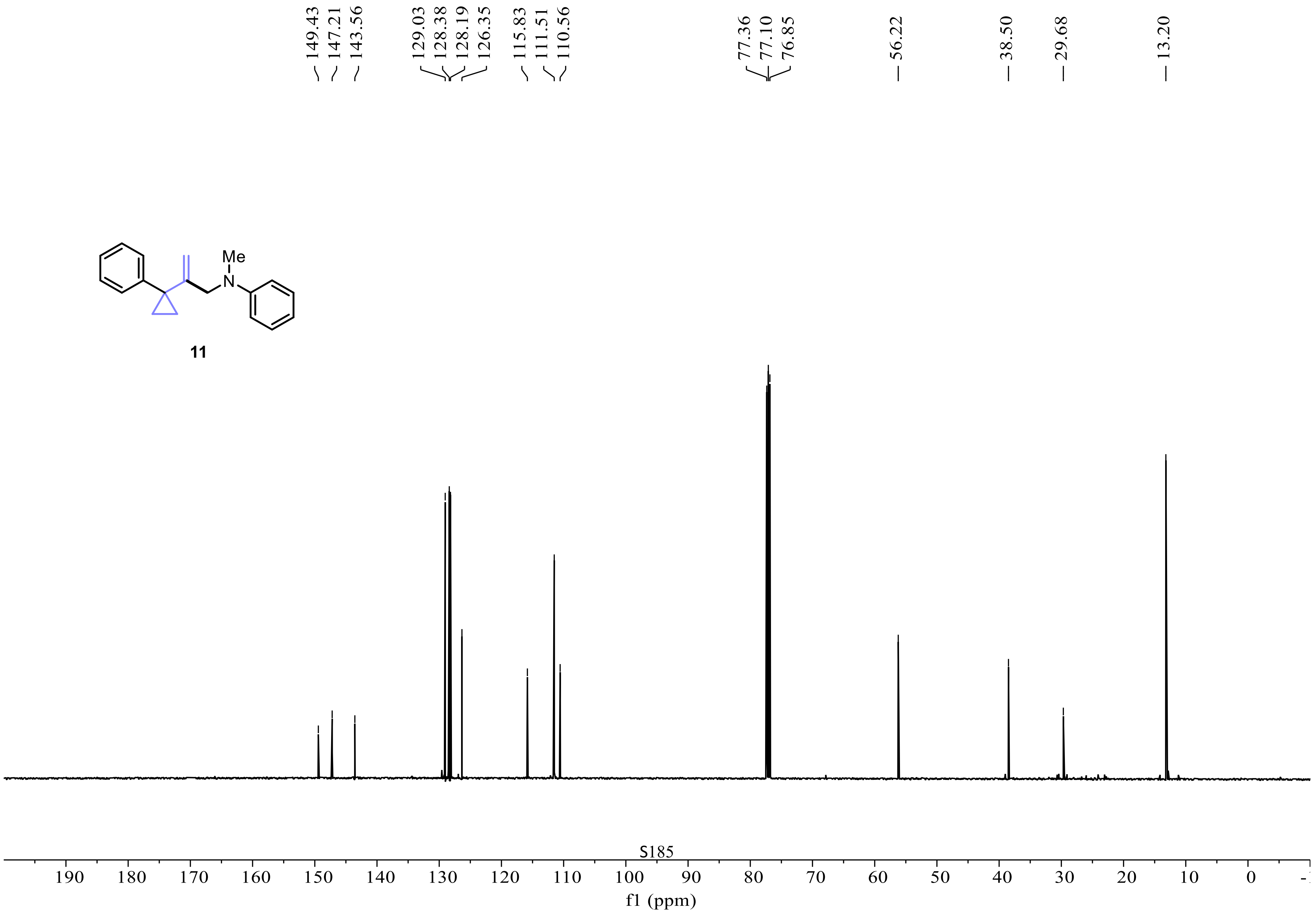
149.43
147.21
143.56
129.03
128.38
128.19
126.35
115.83
111.51
110.56
77.36
77.10
76.85
56.22
38.50
29.68
13.20
Me
N
11
190 180 170 160 150 140 130 120 110 100 90 80 70 60 50 40 30 20 10 0
f1 (ppm)

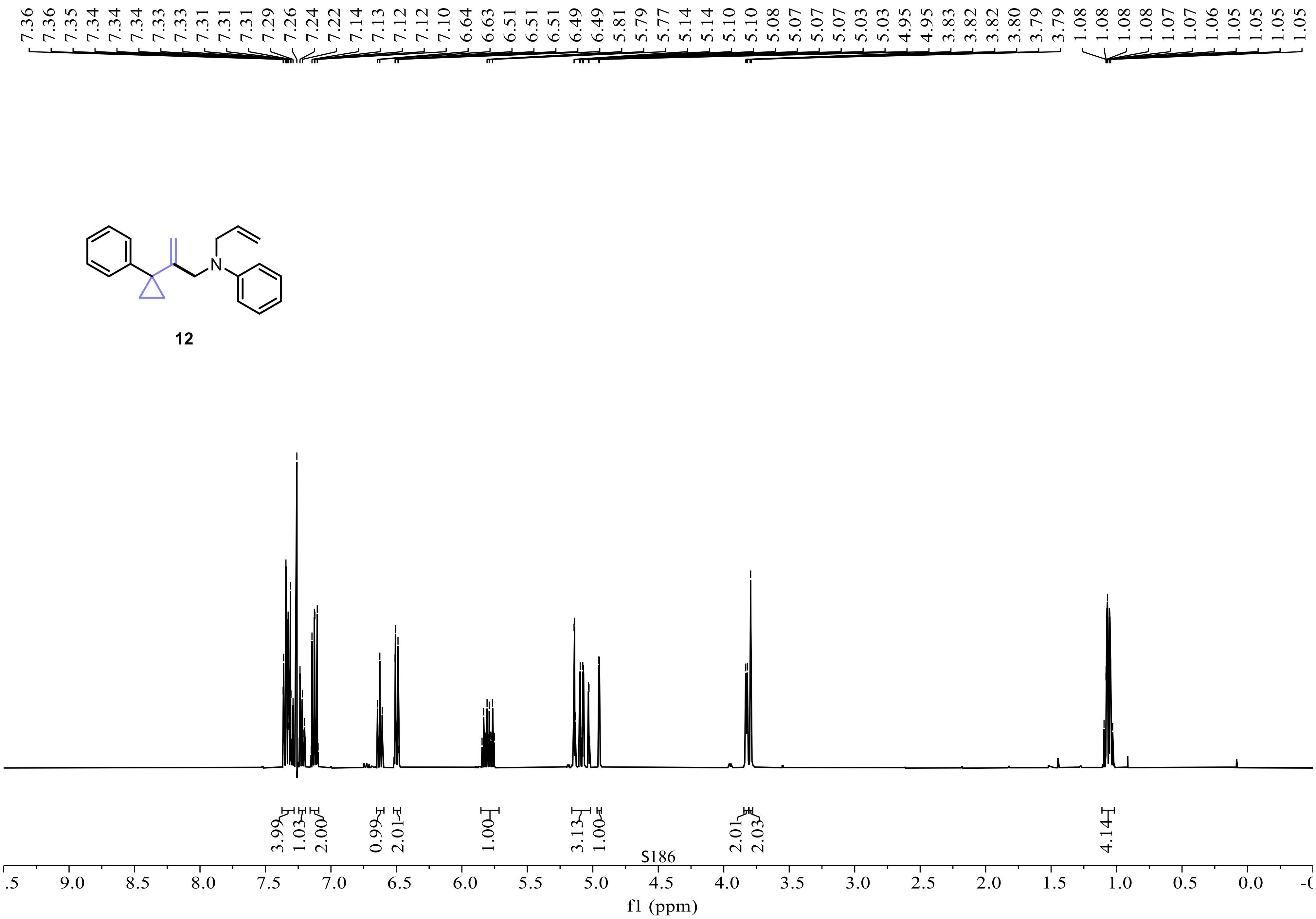

12

7.36 7.36 7.35 7.34 7.34 7.34 7.33 7.33 7.31 7.31 7.31 7.29 7.26 7.24 7.22 7.14 7.13 7.12 7.12 7.10 6.64 6.63 6.51 6.51 6.51 6.49 6.49 5.81 5.79 5.77 5.14 5.14 5.10 5.10 5.08 5.07 5.07 5.07 5.03 5.03 4.95 4.95 3.83 3.82 3.82 3.80 3.79 3.79 1.08 1.08 1.08 1.08 1.07 1.07 1.06 1.05 1.05 1.05 1.05
3.99
1.03
2.00
0.99
2.01
1.00
3.13
1.00
2.01
2.03
4.14
9.0 8.5 8.0 7.5 7.0 6.5 6.0 5.5 5.0 4.5 4.0 3.5 3.0 2.5 2.0 1.5 1.0 0.5 0.0
f1 (ppm)

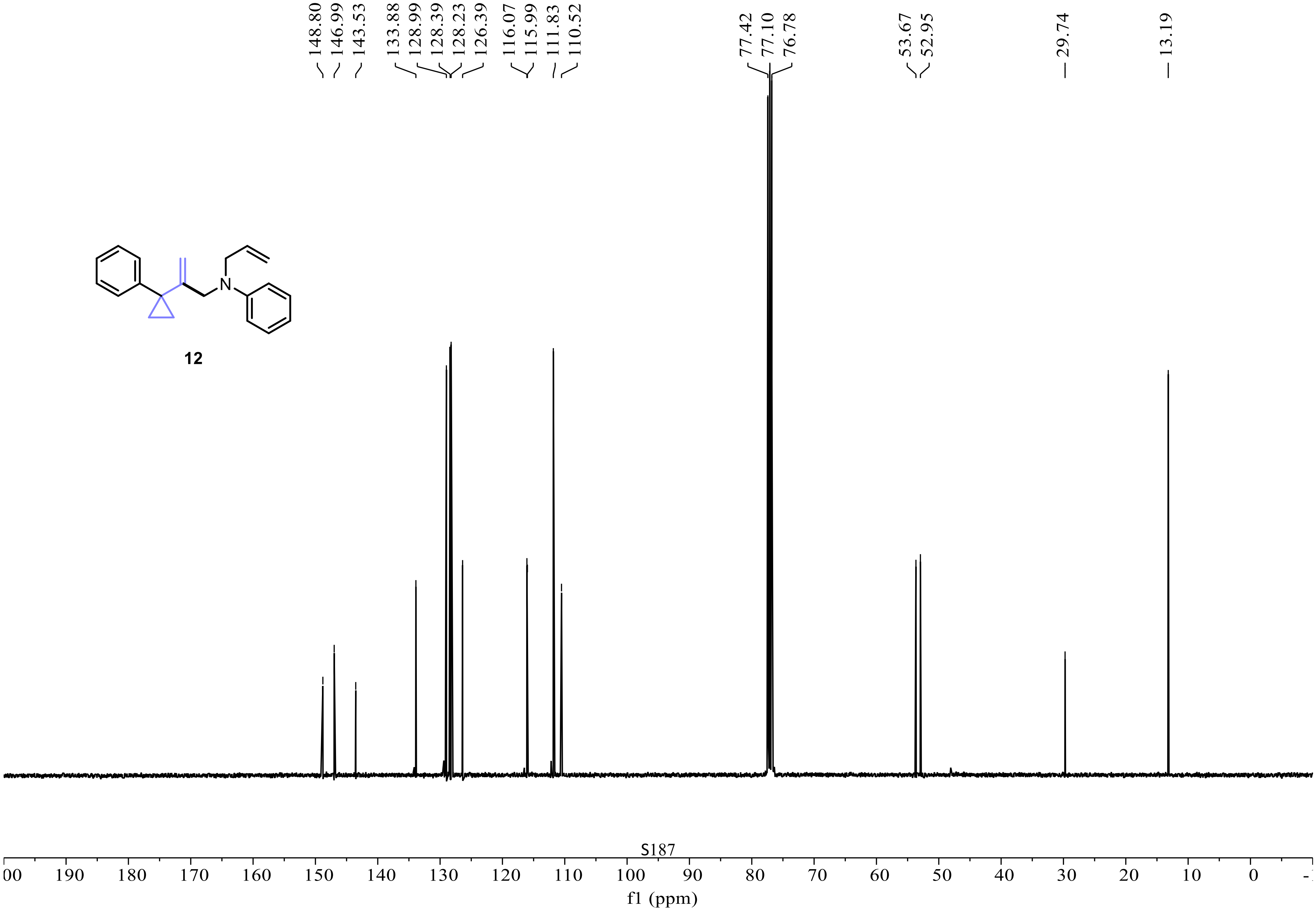
148.80
146.99
143.53
133.88
128.99
128.39
128.23
126.39
116.07
115.99
111.83
110.52
77.42
77.10
76.78
53.67
52.95
29.74
13.19
12
00
190
180
170
160
150
140
130
120
110
100
90
80
70
60
50
40
30
20
10
0
f1 (ppm)

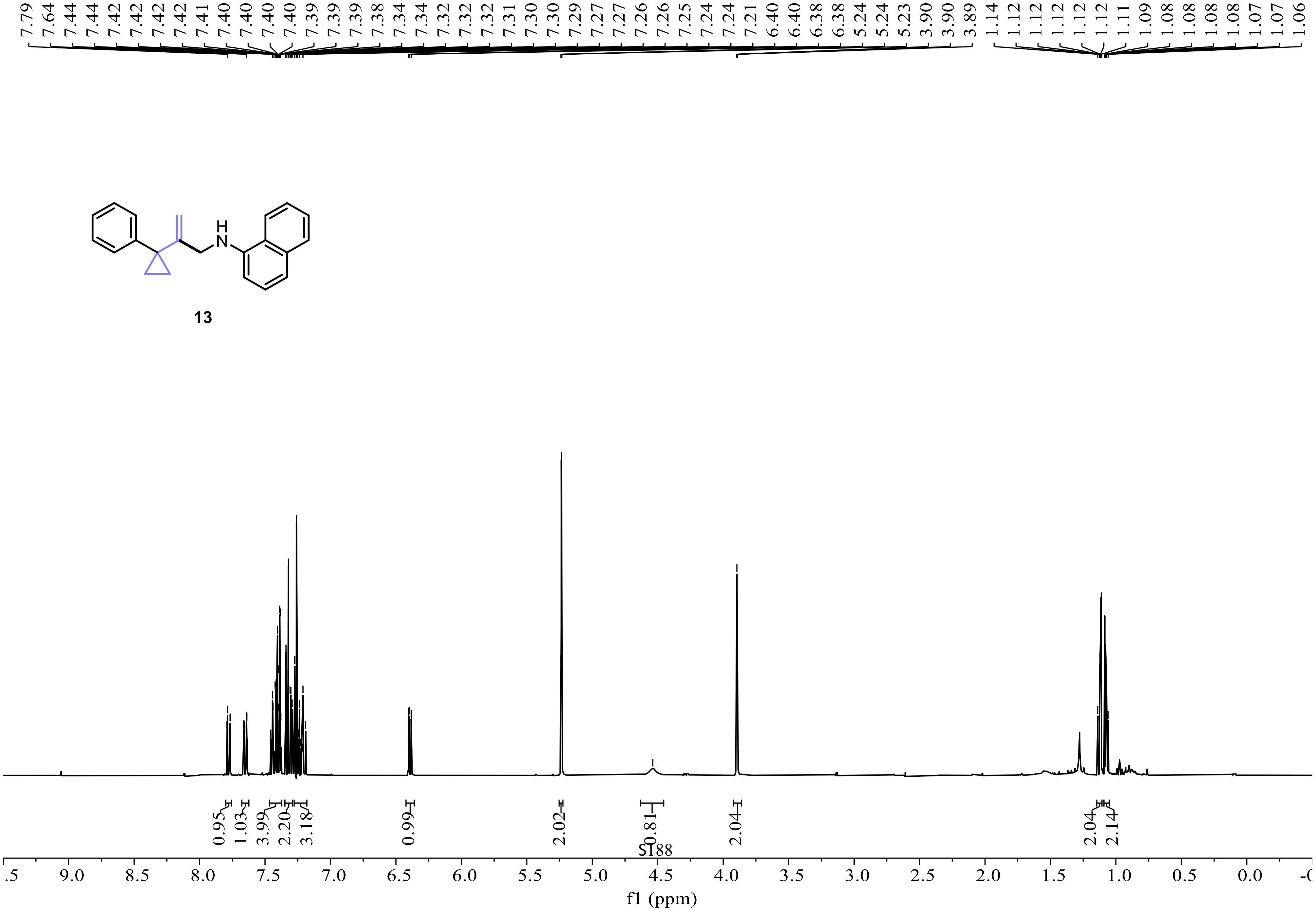
7.79 7.64 7.44 7.44 7.42 7.42 7.42 7.42 7.41 7.40 7.40 7.40 7.40 7.39 7.39 7.39 7.38 7.34 7.34 7.32 7.32 7.32 7.31 7.30 7.30 7.29 7.27 7.27 7.26 7.26 7.25 7.24 7.24 7.21 6.40 6.40 6.38 6.38 5.24 5.24 5.23 3.90 3.90 3.89 1.14 1.12 1.12 1.12 1.12 1.12 1.11 1.09 1.08 1.08 1.08 1.08 1.07 1.07 1.06
H
N
13
0.95
1.03
3.99
2.20
3.18
0.99
2.02
0.81
2.04
2.04
2.14
f1 (ppm)

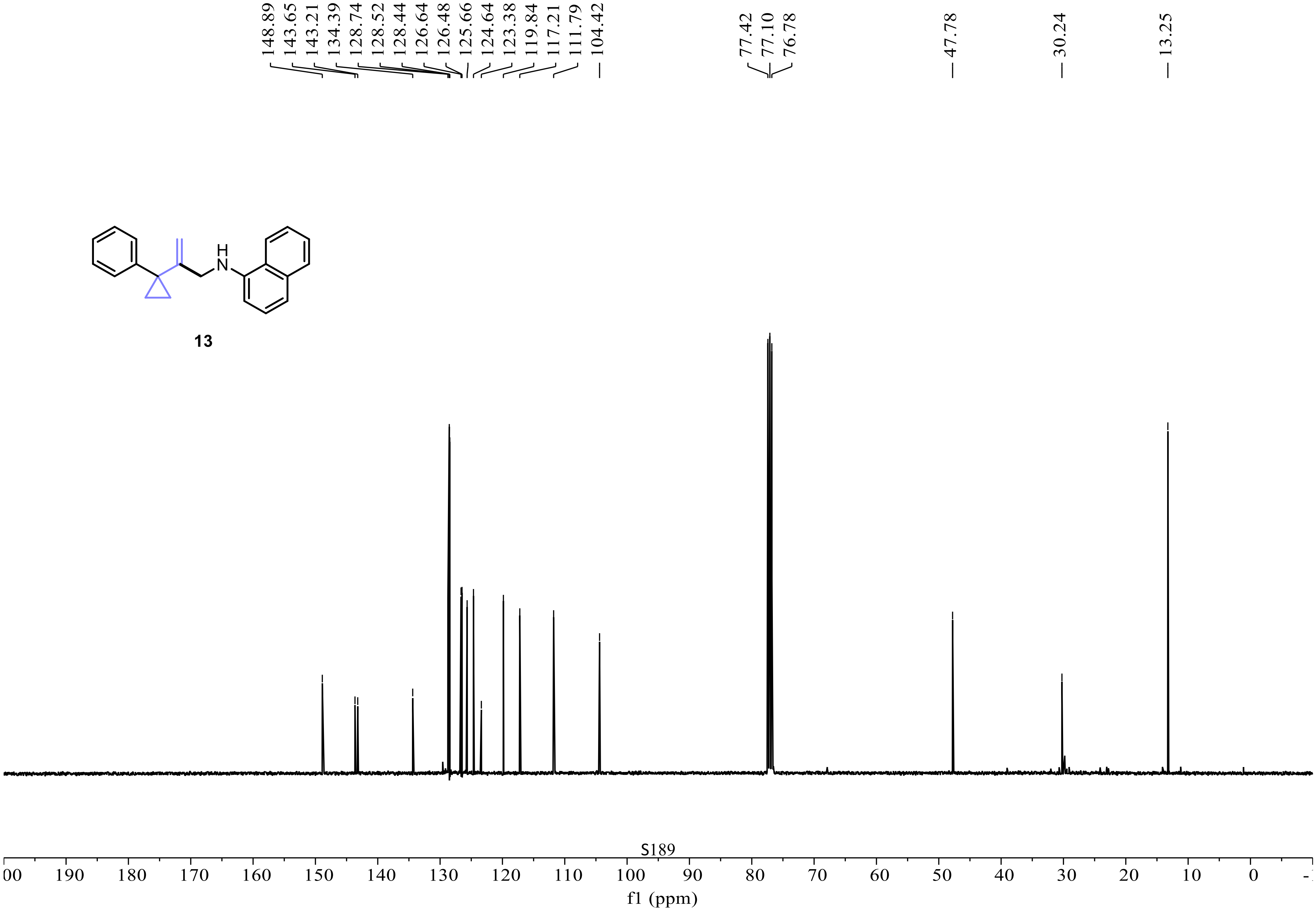
148.89
143.65
143.21
134.39
128.74
128.52
128.44
126.64
126.48
125.66
124.64
123.38
119.84
117.21
111.79
104.42
77.42
77.10
76.78
47.78
30.24
13.25
H
N
13
190 180 170 160 150 140 130 120 110 100 90 80 70 60 50 40 30 20 10 0
f1 (ppm)

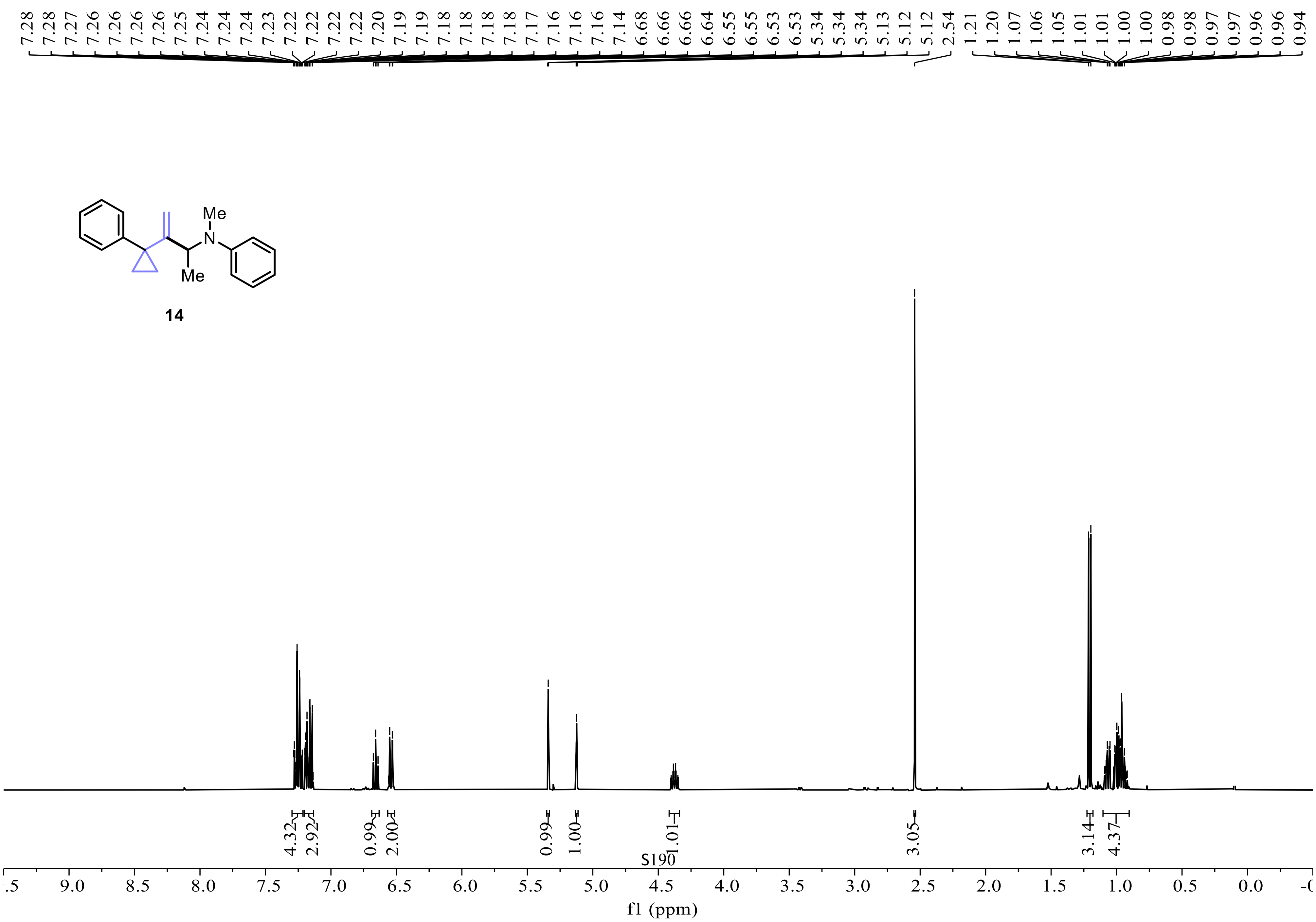
Me
N
Me
14
7.28 7.28 7.27 7.26 7.26 7.26 7.25 7.24 7.24 7.24 7.23 7.22 7.22 7.22 7.22 7.22 7.20 7.19 7.19 7.18 7.18 7.18 7.18 7.17 7.16 7.16 7.16 7.14 6.68 6.66 6.66 6.64 6.55 6.55 6.53 6.53 5.34 5.34 5.34 5.13 5.12 5.12 2.54 1.21 1.20 1.07 1.06 1.05 1.01 1.01 1.00 1.00 0.98 0.98 0.97 0.97 0.96 0.96 0.94
4.32
2.92
0.99
2.00
0.99
1.00
1.01
3.05
3.14
4.37
.5 9.0 8.5 8.0 7.5 7.0 6.5 6.0 5.5 5.0 4.5 4.0 3.5 3.0 2.5 2.0 1.5 1.0 0.5 0.0
f1 (ppm)

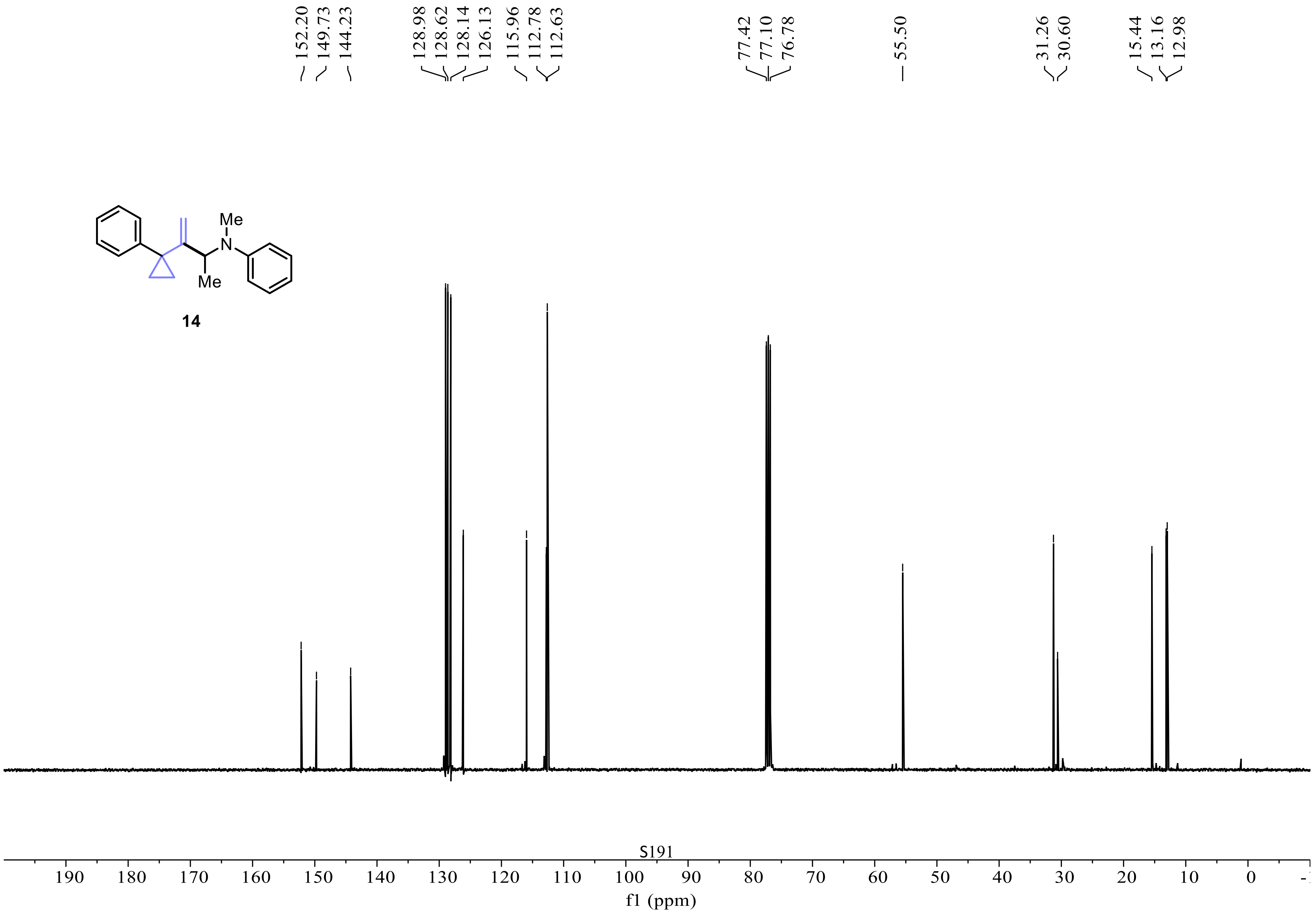
152.20
149.73
144.23
128.98
128.62
128.14
126.13
115.96
112.78
112.63
77.42
77.10
76.78
55.50
31.26
30.60
15.44
13.16
12.98
Me
N
Me
14
190 180 170 160 150 140 130 120 110 100 90 80 70 60 50 40 30 20 10 0
f1 (ppm)

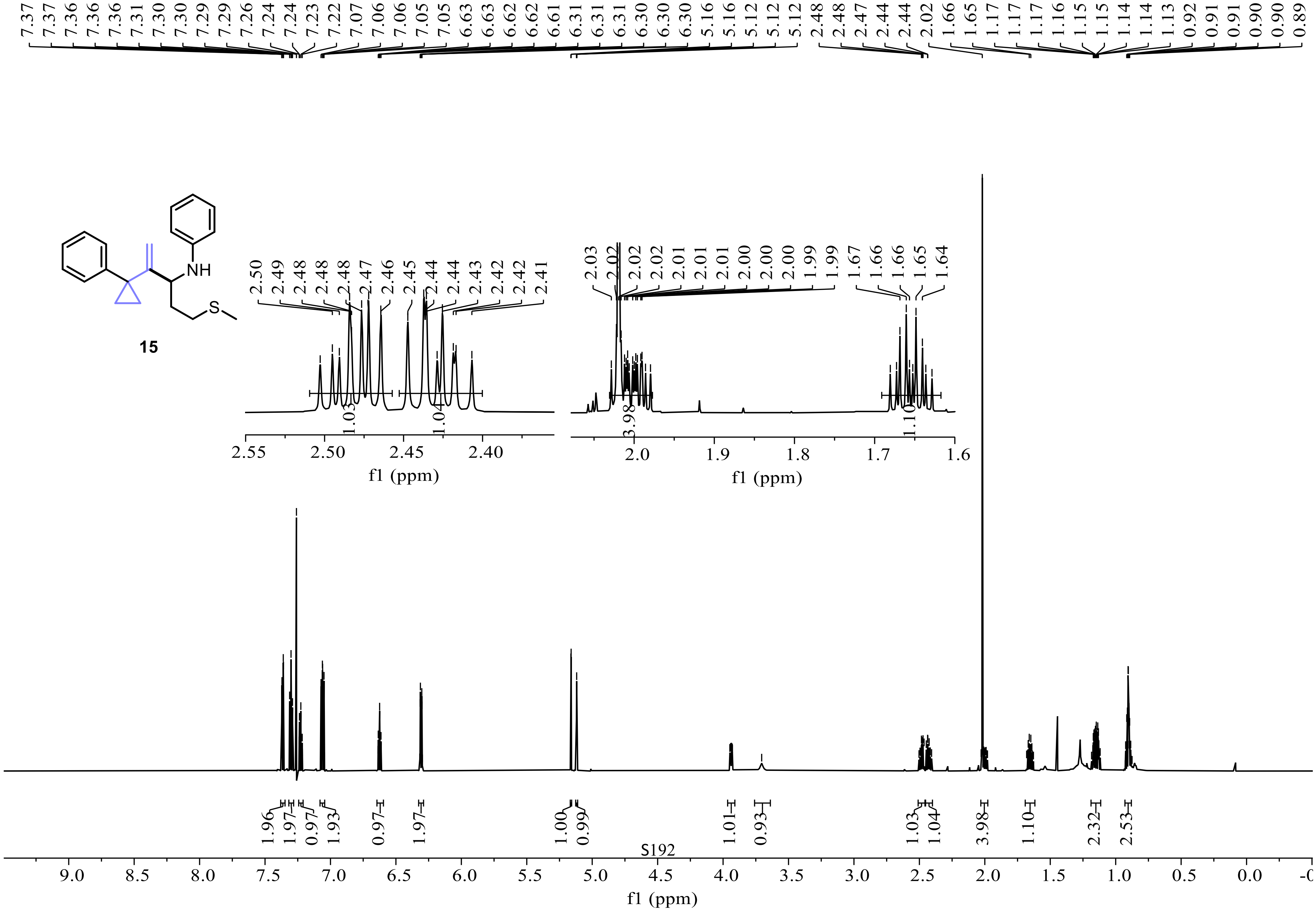
7.37 7.37 7.36 7.36 7.36 7.31 7.30 7.30 7.29 7.29 7.26 7.24 7.24 7.23 7.22 7.07 7.06 7.06 7.05 7.05 6.63 6.63 6.62 6.62 6.61 6.31 6.31 6.31 6.30 6.30 6.30 5.16 5.16 5.12 5.12 5.12 2.48 2.48 2.47 2.44 2.44 2.02 1.66 1.65 1.17 1.17 1.17 1.16 1.15 1.15 1.14 1.14 1.13 0.92 0.91 0.91 0.90 0.90 0.89
NH
S
15
2.50 2.49 2.48 2.48 2.48 2.47 2.46 2.45 2.44 2.44 2.43 2.42 2.42 2.41
1.03
1.04
2.55 2.50 2.45 2.40
f1 (ppm)
2.03 2.02 2.02 2.02 2.01 2.01 2.01 2.00 2.00 2.00 1.99 1.99 1.67 1.66 1.66 1.65 1.64
3.98
1.10
2.0 1.9 1.8 1.7 1.6
f1 (ppm)
1.96 1.97 0.97 1.93 0.97 1.97 1.00 0.99 1.01 0.93 1.03 1.04 3.98 1.10 2.32 2.53
9.0 8.5 8.0 7.5 7.0 6.5 6.0 5.5 5.0 4.5 4.0 3.5 3.0 2.5 2.0 1.5 1.0 0.5 0.0
f1 (ppm)

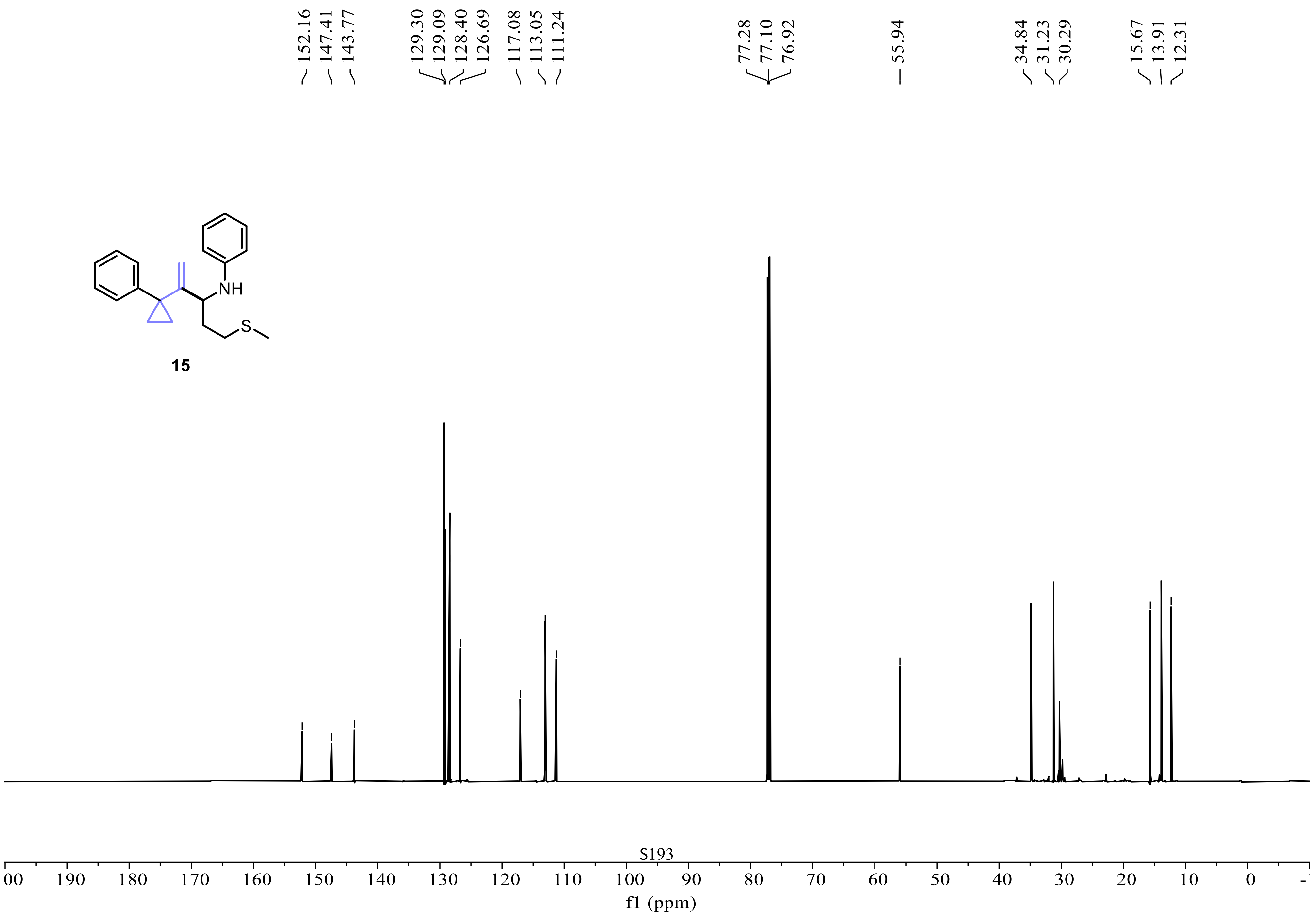
152.16
147.41
143.77
129.30
129.09
128.40
126.69
117.08
113.05
111.24
77.28
77.10
76.92
55.94
34.84
31.23
30.29
15.67
13.91
12.31
NH
S
15
f1 (ppm)

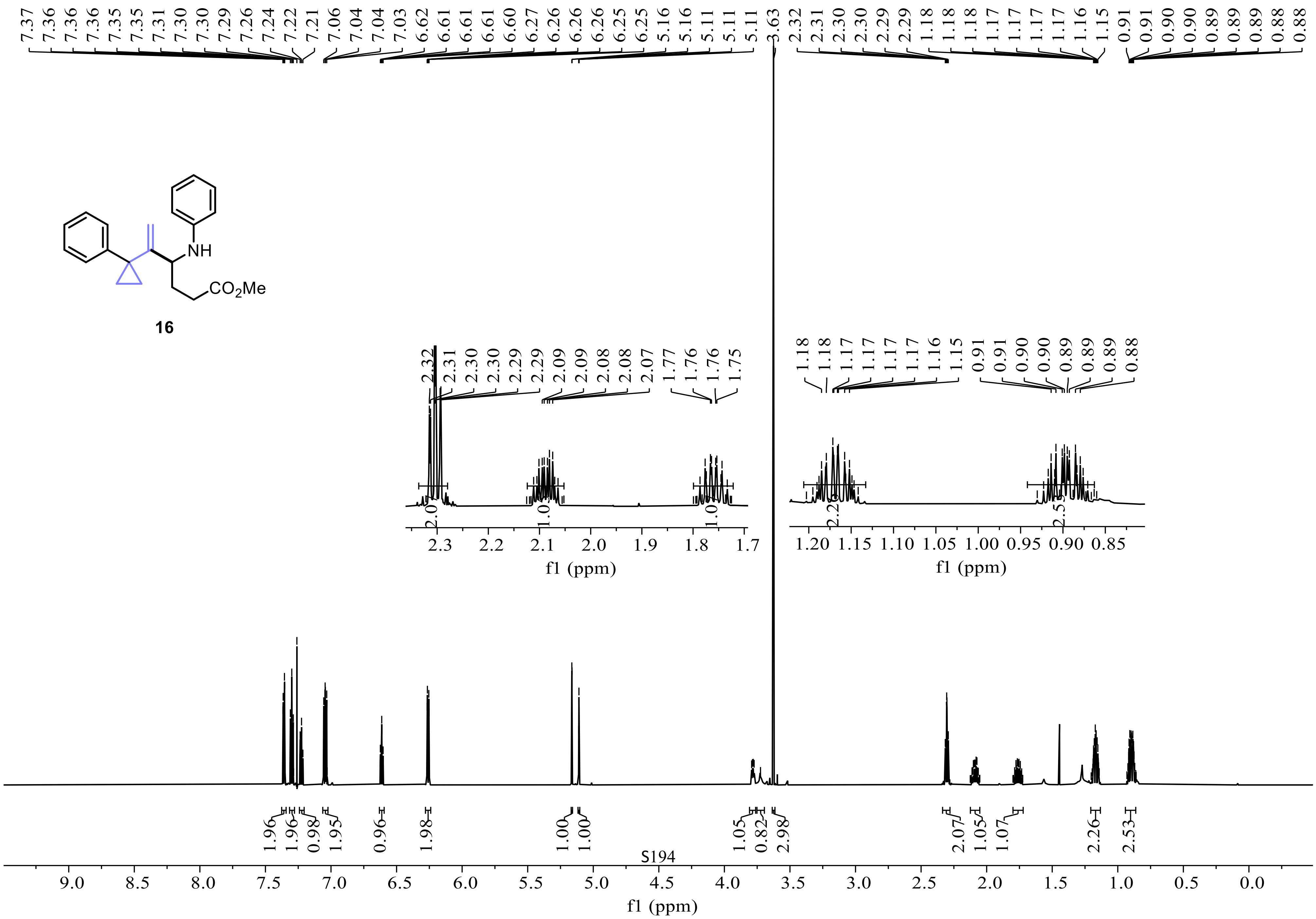

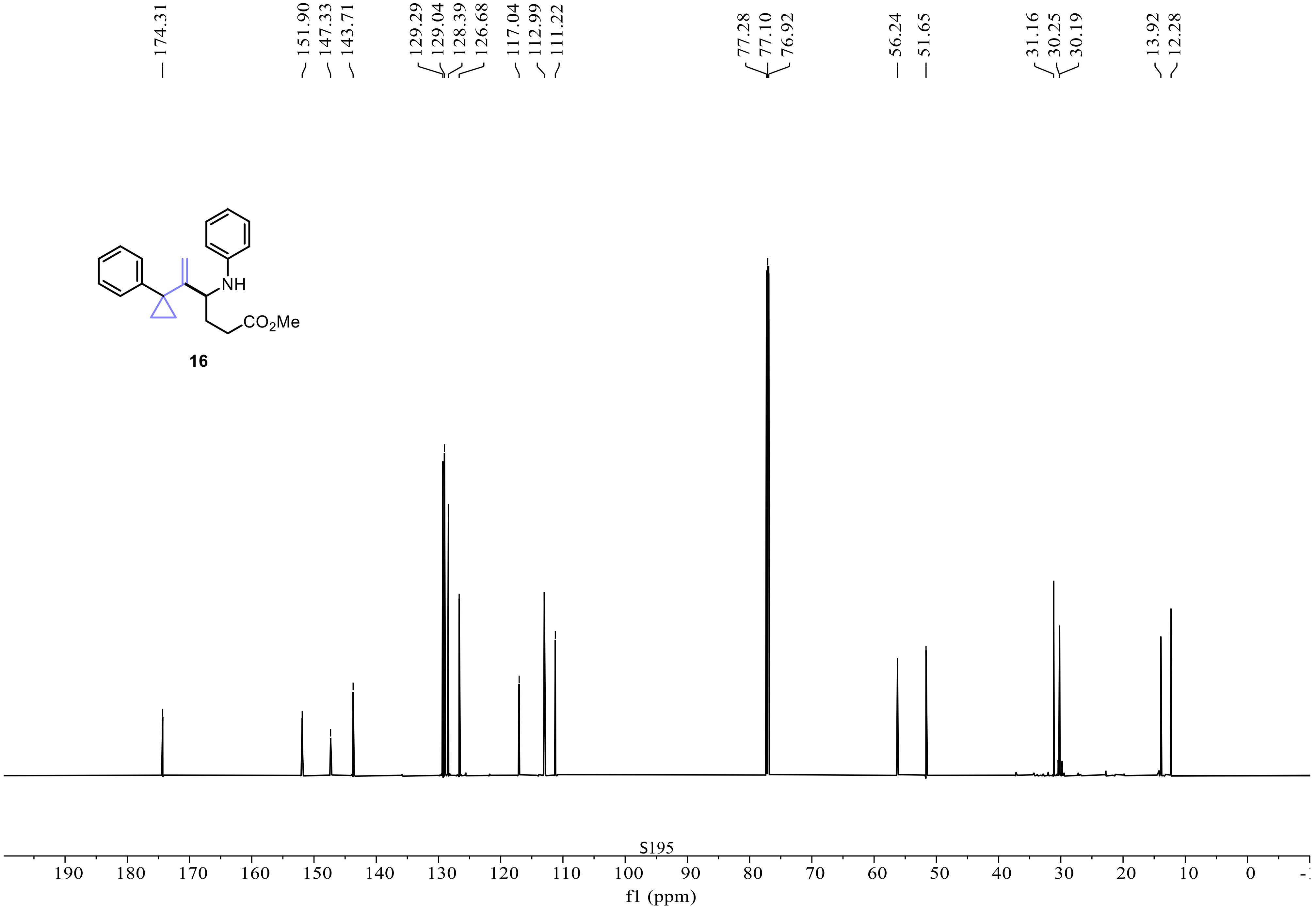
174.31
151.90
147.33
143.71
129.29
129.04
128.39
126.68
117.04
112.99
111.22
77.28
77.10
76.92
56.24
51.65
31.16
30.25
30.19
13.92
12.28
NH
CO_2Me
16
190 180 170 160 150 140 130 120 110 100 90 80 70 60 50 40 30 20 10 0
f1 (ppm)

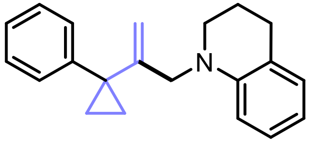

17

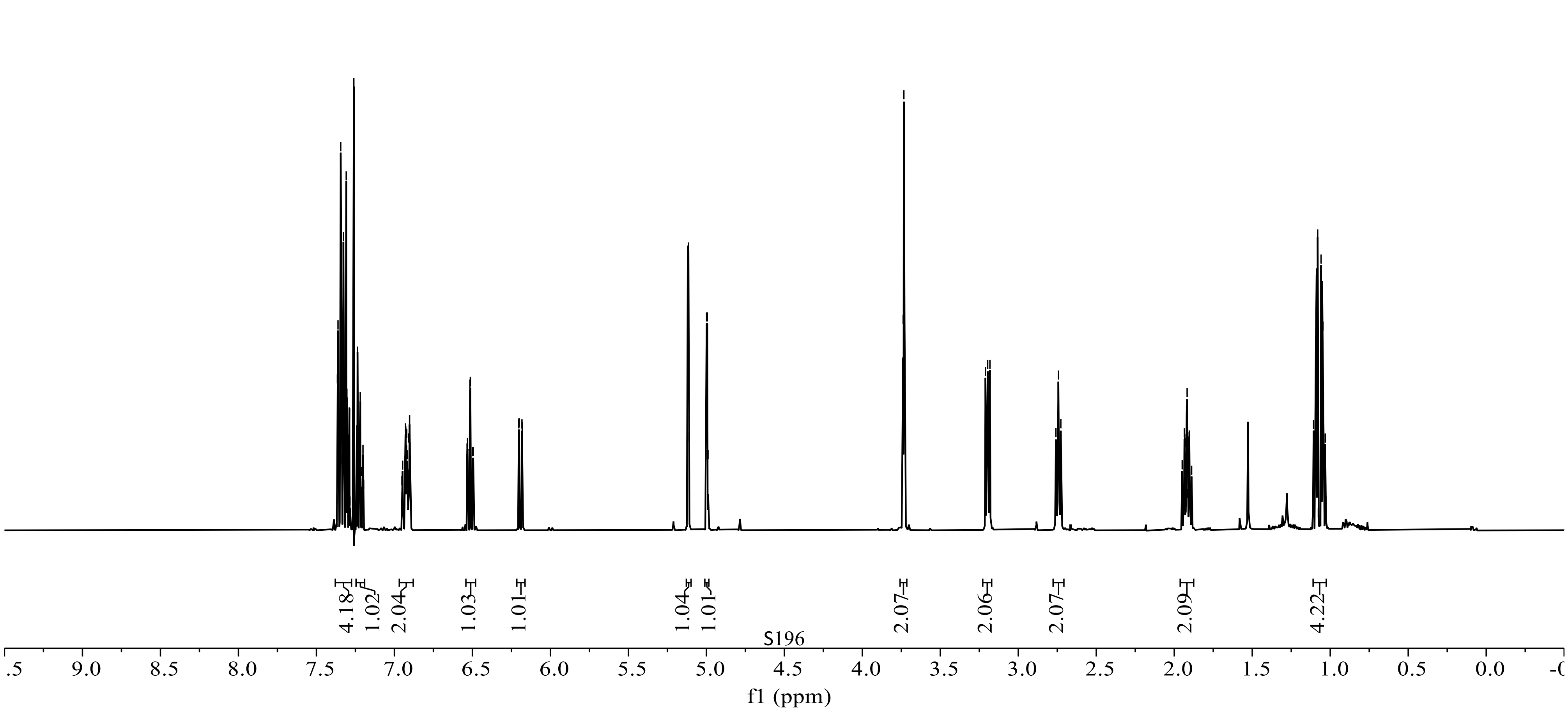
7.37 7.36 7.35 7.34 7.34 7.34 7.33 7.33 7.31 7.31 7.30 7.29 7.26 7.24 7.24 7.23 7.22 6.93 6.93 6.92 6.90 6.52 6.51 6.20 6.20 6.18 6.18 5.12 5.11 5.00 4.99 3.74 3.73 3.73 3.21 3.20 3.20 3.19 3.18 2.76 2.74 2.73 1.93 1.92 1.92 1.90 1.11 1.09 1.09 1.09 1.09 1.09 1.08 1.08 1.06 1.06 1.05 1.05 1.05
4.18 1.02 2.04 1.03 1.01 1.04 1.01 2.07 2.06 2.07 2.09 4.22
.5 9.0 8.5 8.0 7.5 7.0 6.5 6.0 5.5 5.0 4.5 4.0 3.5 3.0 2.5 2.0 1.5 1.0 0.5 0.0
f1 (ppm)

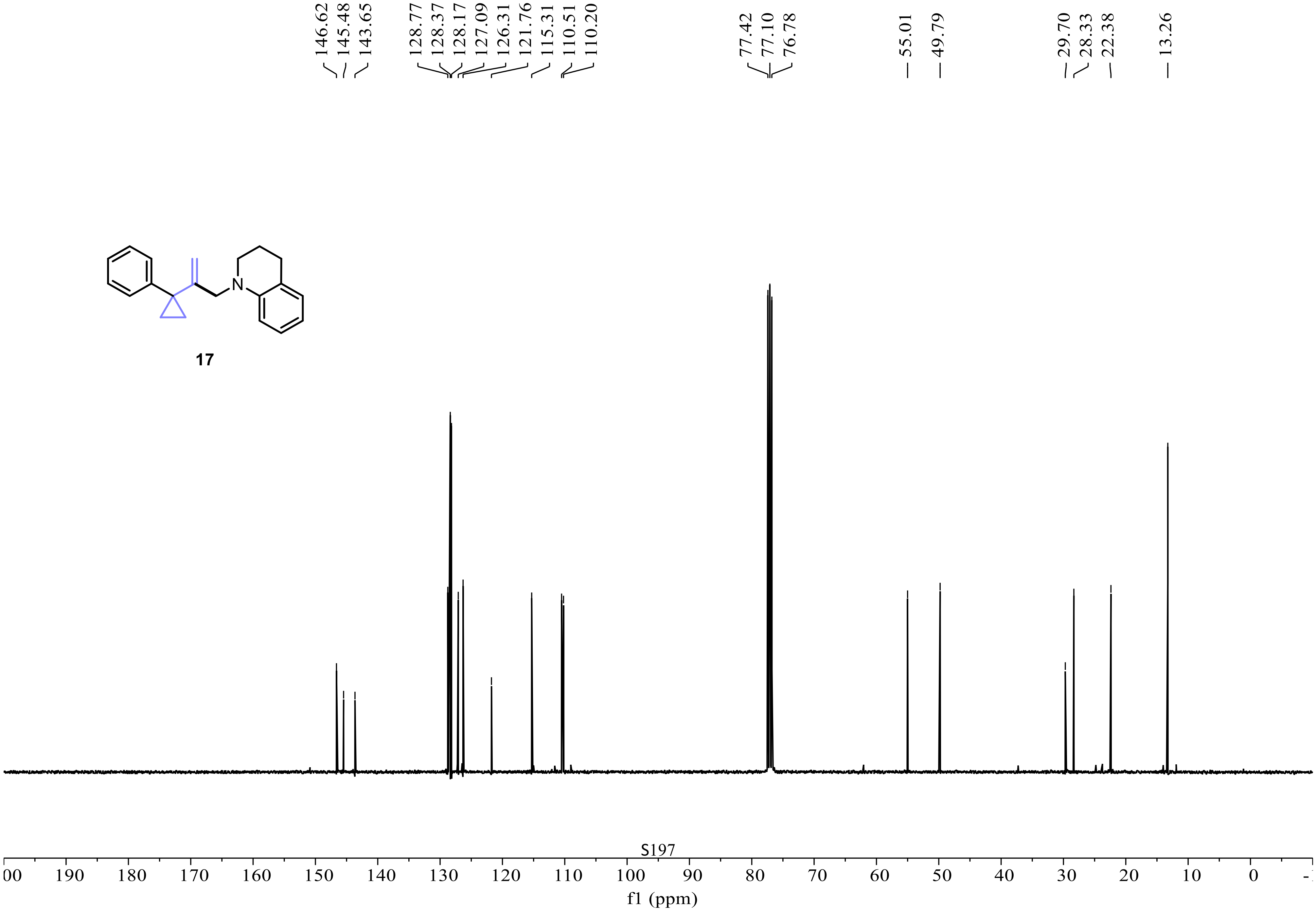
146.62
145.48
143.65
128.77
128.37
128.17
127.09
126.31
121.76
115.31
110.51
110.20
77.42
77.10
76.78
55.01
49.79
29.70
28.33
22.38
13.26
N
17
00
190
180
170
160
150
140
130
120
110
100
90
80
70
60
50
40
30
20
10
0
f1 (ppm)

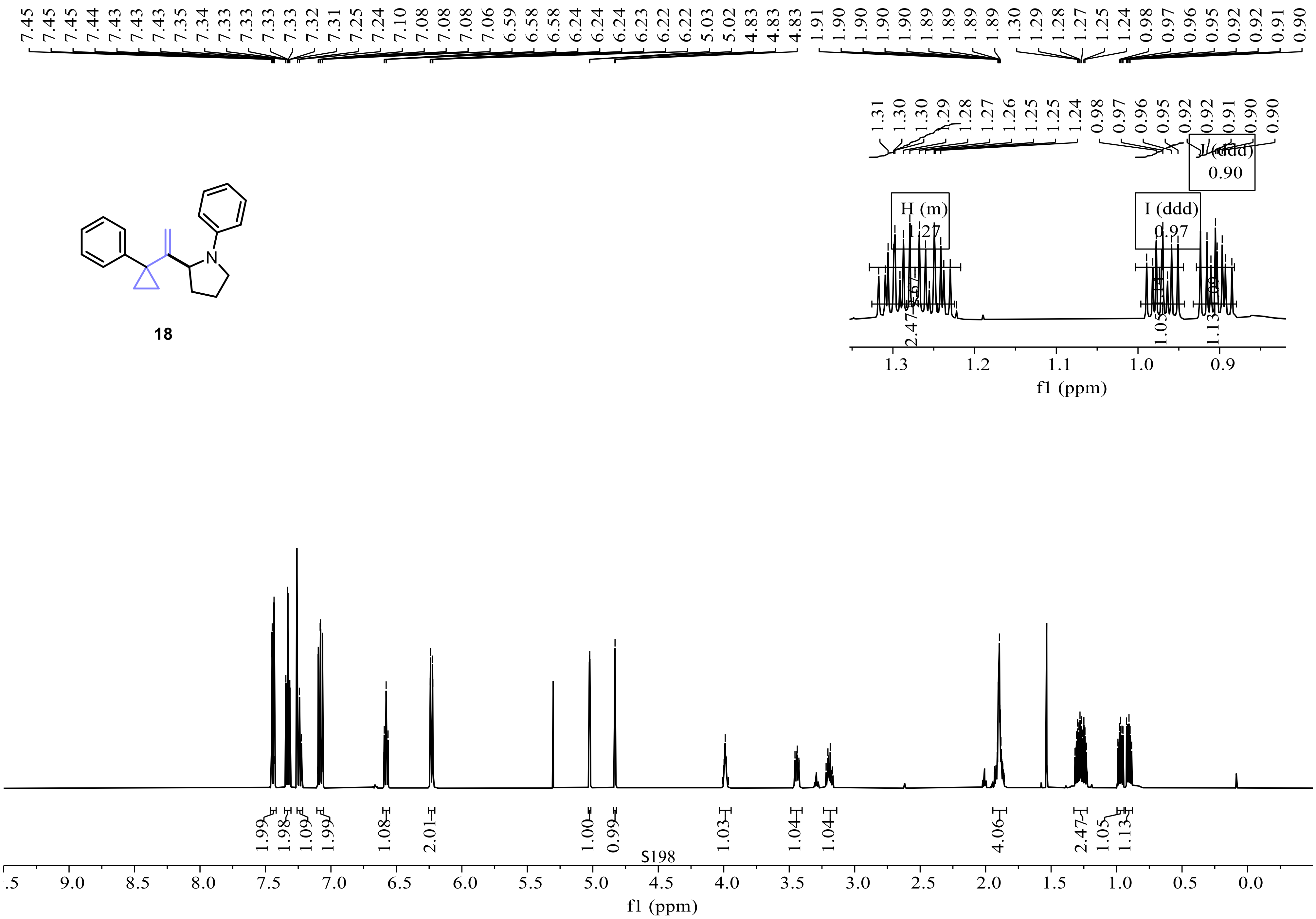

18

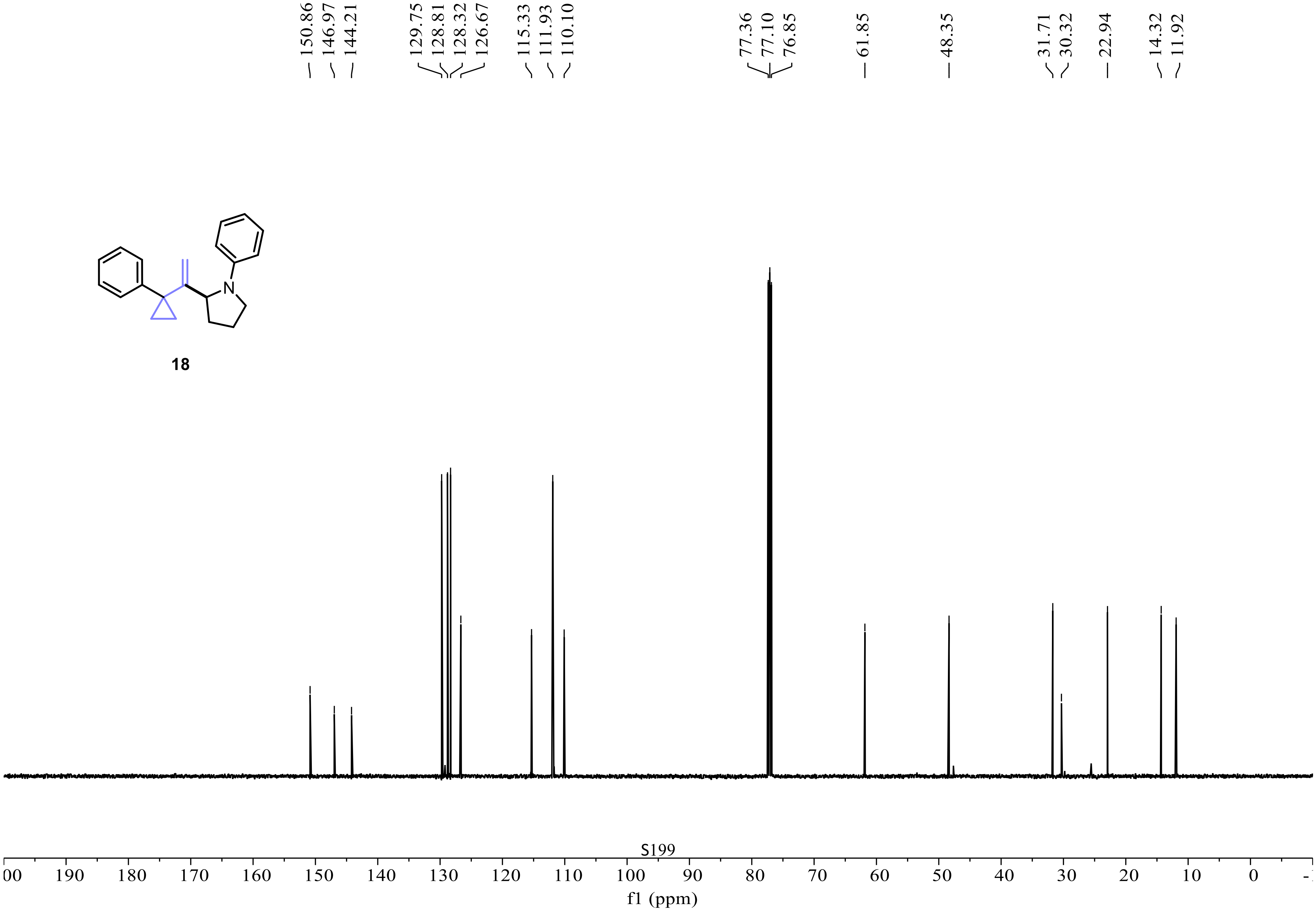
150.86
146.97
144.21
129.75
128.81
128.32
126.67
115.33
111.93
110.10
77.36
77.10
76.85
61.85
48.35
31.71
30.32
22.94
14.32
11.92
18
00 190 180 170 160 150 140 130 120 110 100 90 80 70 60 50 40 30 20 10 0
f1 (ppm)

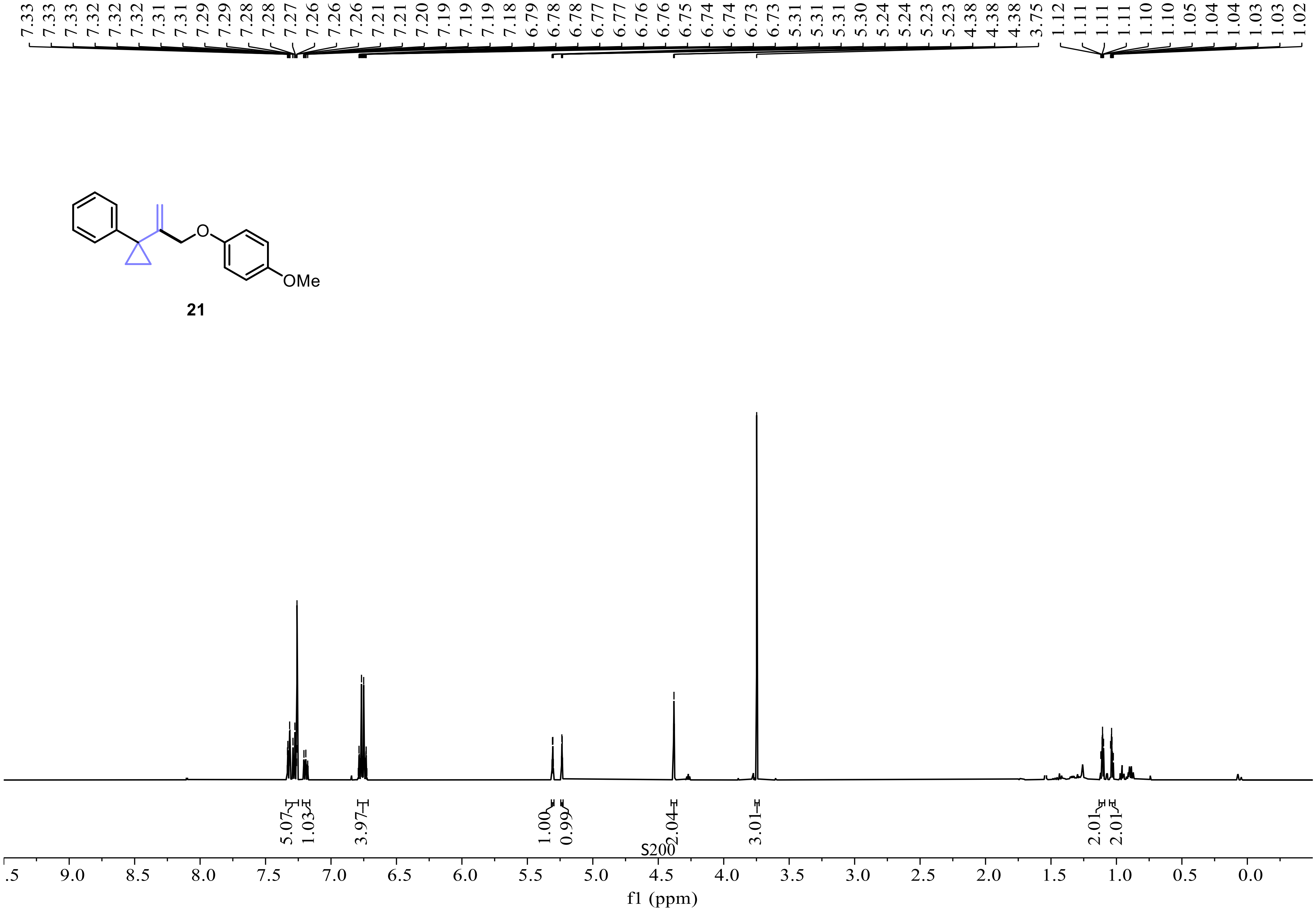
7.33 7.33 7.33 7.32 7.32 7.32 7.31 7.31 7.29 7.29 7.28 7.28 7.27 7.26 7.26 7.26 7.21 7.21 7.20 7.19 7.19 7.19 7.18 6.79 6.78 6.78 6.77 6.77 6.76 6.76 6.75 6.74 6.74 6.73 6.73 5.31 5.31 5.31 5.30 5.24 5.24 5.23 5.23 4.38 4.38 4.38 3.75 1.12 1.11 1.11 1.11 1.10 1.10 1.05 1.04 1.04 1.03 1.03 1.02
O
OMe
21
5.07
1.03
3.97
1.00
0.99
2.04
3.01
2.01
2.01
.5 9.0 8.5 8.0 7.5 7.0 6.5 6.0 5.5 5.0 4.5 4.0 3.5 3.0 2.5 2.0 1.5 1.0 0.5 0.0
f1 (ppm)

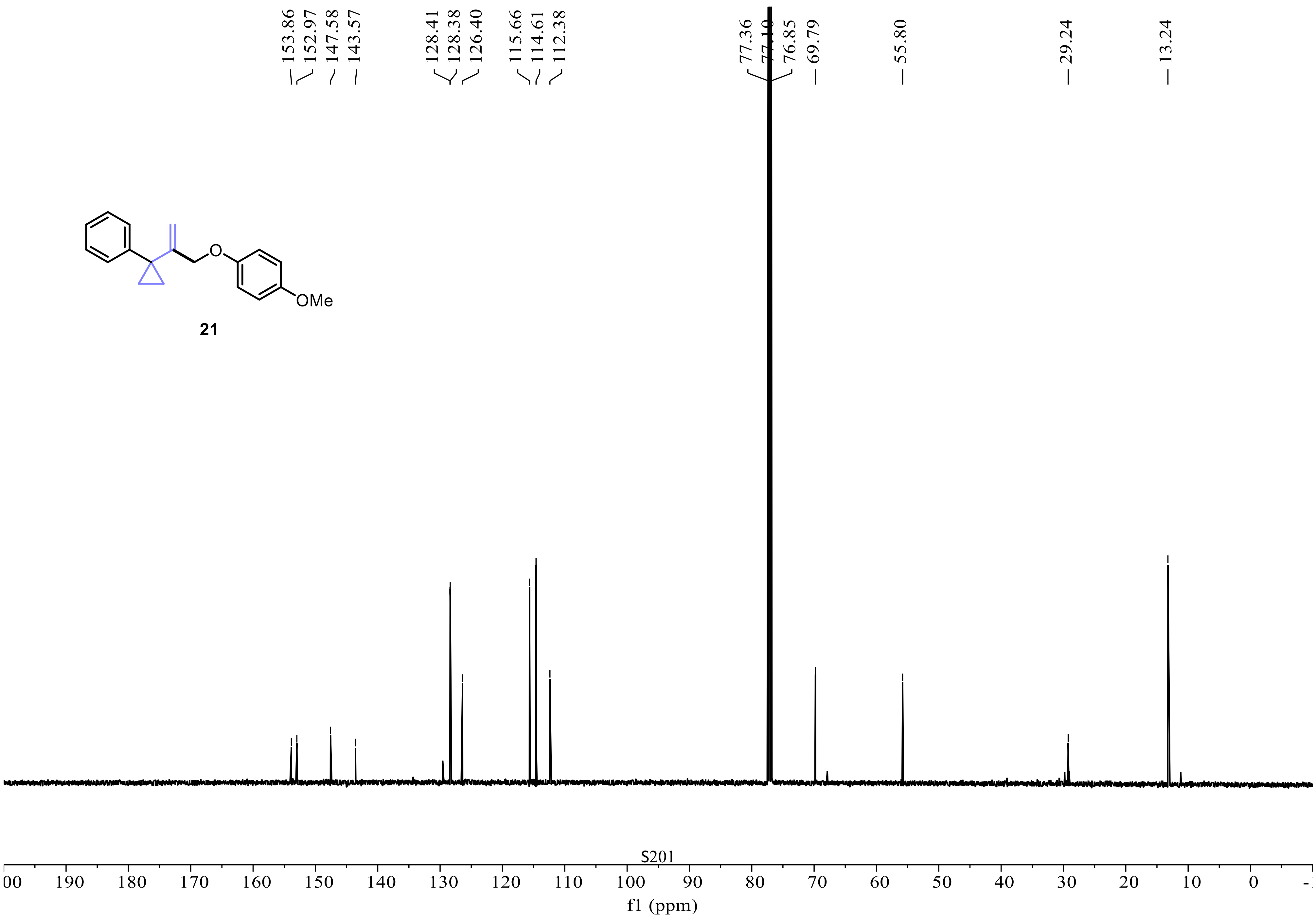
153.86
152.97
147.58
143.57
128.41
128.38
126.40
115.66
114.61
112.38
77.36
76.85
69.79
55.80
29.24
13.24
O
OMe
21
190 180 170 160 150 140 130 120 110 100 90 80 70 60 50 40 30 20 10 0
f1 (ppm)

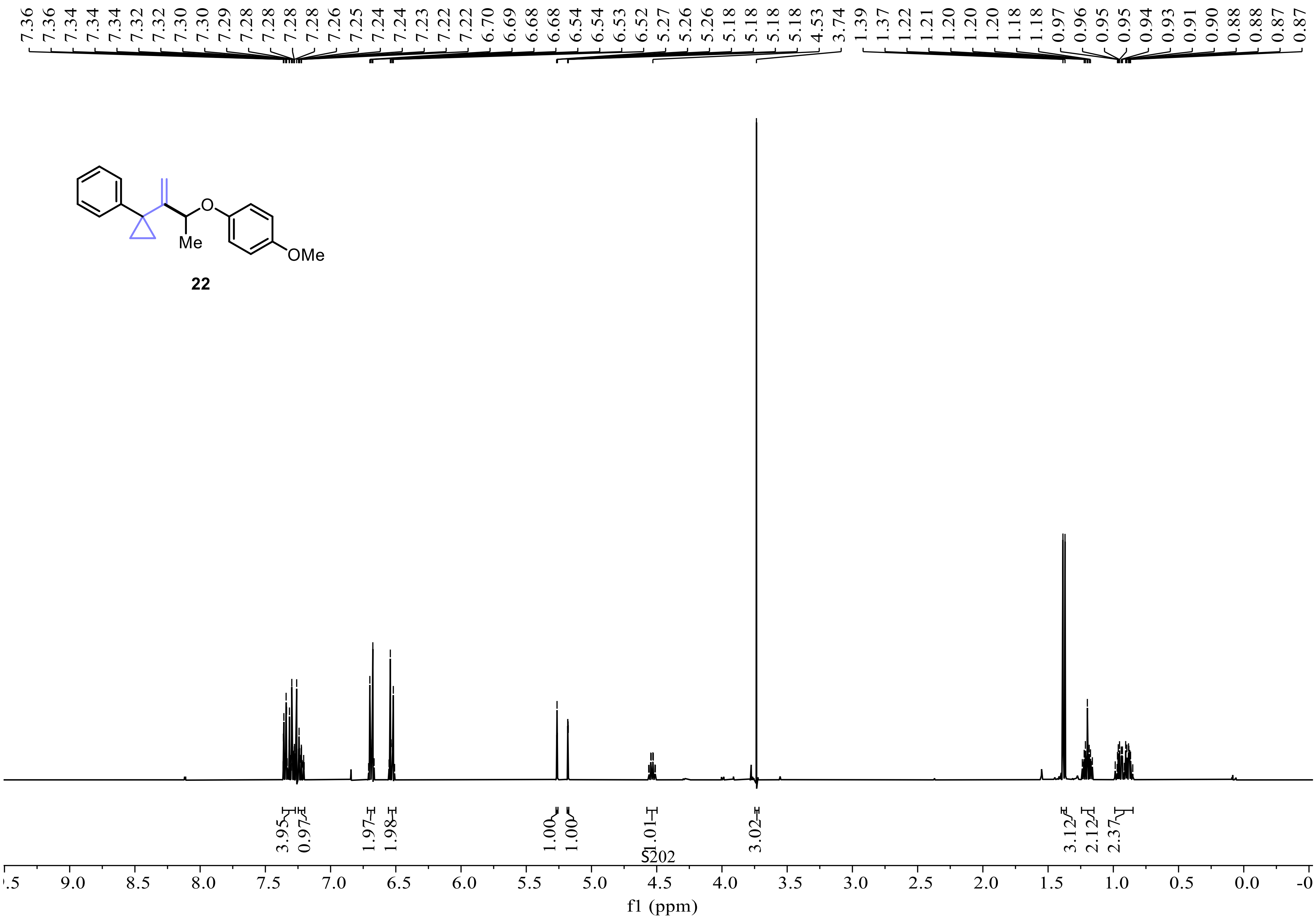
Me
OMe
22
7.36 7.36 7.34 7.34 7.34 7.32 7.32 7.30 7.30 7.29 7.28 7.28 7.28 7.28 7.26 7.25 7.24 7.24 7.23 7.22 7.22 6.70 6.69 6.68 6.68 6.54 6.54 6.53 6.52 5.27 5.26 5.26 5.18 5.18 5.18 5.18 4.53 3.74 1.39 1.37 1.22 1.21 1.20 1.20 1.20 1.18 1.18 0.97 0.96 0.95 0.95 0.94 0.93 0.91 0.90 0.88 0.88 0.87 0.87
3.95 0.97 1.97 1.98 1.00 1.00 1.01 3.02 3.12 2.12 2.37
9.0 8.5 8.0 7.5 7.0 6.5 6.0 5.5 5.0 4.5 4.0 3.5 3.0 2.5 2.0 1.5 1.0 0.5 0.0
f1 (ppm)

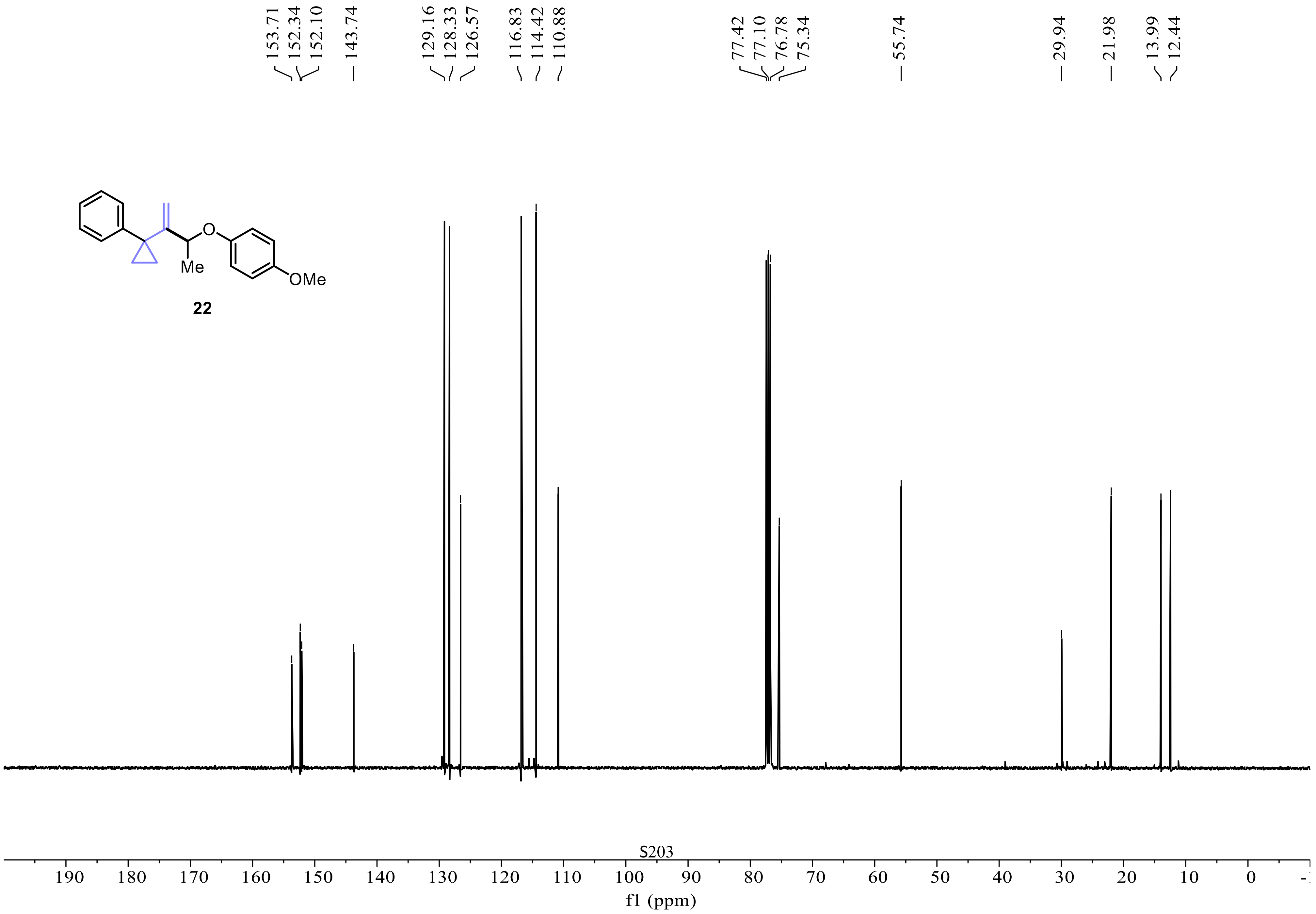
153.71
152.34
152.10
143.74
129.16
128.33
126.57
116.83
114.42
110.88
77.42
77.10
76.78
75.34
55.74
29.94
21.98
13.99
12.44
O
Me
OMe
22
190 180 170 160 150 140 130 120 110 100 90 80 70 60 50 40 30 20 10 0
f1 (ppm)

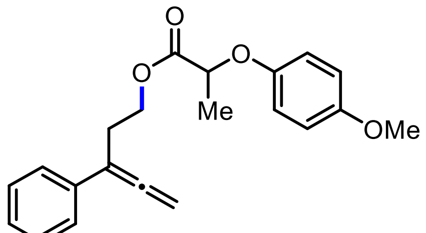
O
O
O
Me
OMe

22-bP

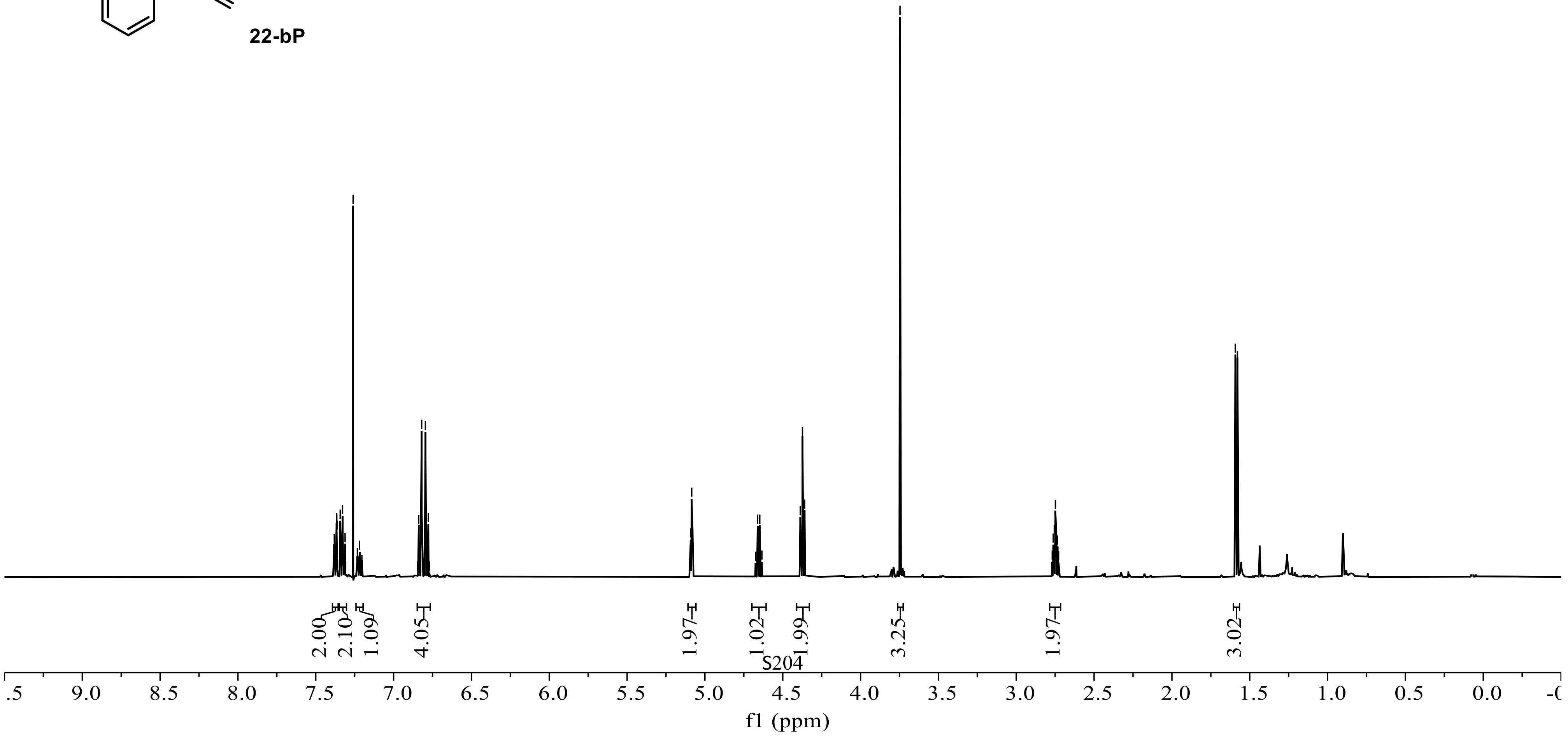
7.38 7.38 7.38 7.38 7.37 7.37 7.36 7.36 7.35 7.34 7.34 7.33 7.33 7.33 7.33 7.32 7.32 7.31 7.31 7.26 7.24 7.23 7.23 7.22 7.22 7.21 7.20 6.84 6.84 6.83 6.83 6.82 6.81 6.80 6.80 6.79 6.78 6.78 6.78 5.09 5.08 5.08 4.67 4.66 4.65 4.63 4.39 4.37 4.36 3.75 2.77 2.76 2.76 2.75 2.74 2.73 2.73 1.59 1.58
2.00
2.10
1.09
4.05
1.97
1.02
1.99
3.25
1.97
3.02
.5 9.0 8.5 8.0 7.5 7.0 6.5 6.0 5.5 5.0 4.5 4.0 3.5 3.0 2.5 2.0 1.5 1.0 0.5 0.0
f1 (ppm)

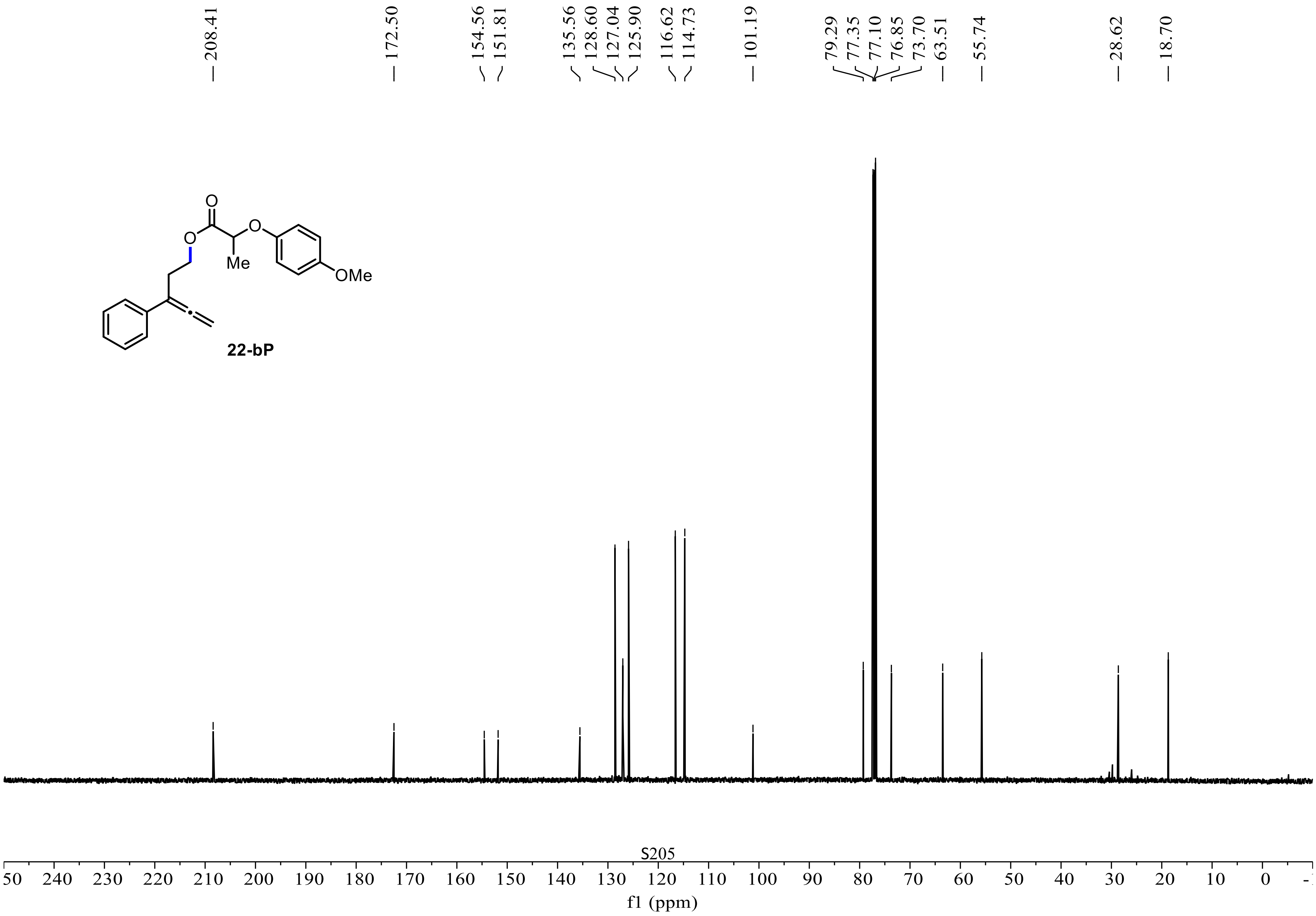
208.41
172.50
154.56
151.81
135.56
128.60
127.04
125.90
116.62
114.73
101.19
79.29
77.35
77.10
76.85
73.70
63.51
55.74
28.62
18.70
O
O
O
Me
OMe
22-bP
50 240 230 220 210 200 190 180 170 160 150 140 130 120 110 100 90 80 70 60 50 40 30 20 10 0
f1 (ppm)

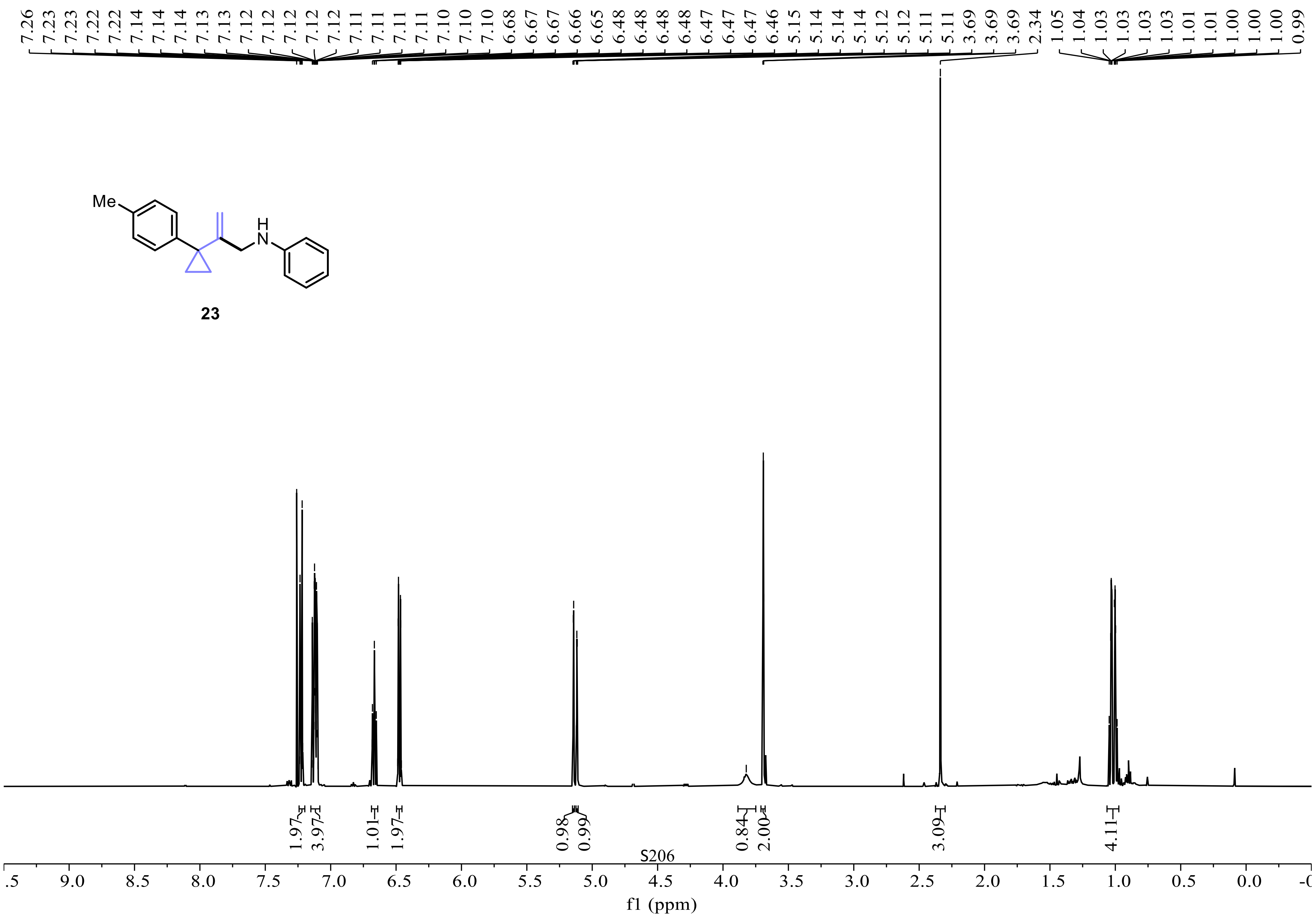
7.26 7.23 7.23 7.22 7.22 7.14 7.14 7.14 7.13 7.13 7.12 7.12 7.12 7.12 7.12 7.11 7.11 7.11 7.11 7.10 7.10 7.10 6.68 6.67 6.67 6.66 6.65 6.48 6.48 6.48 6.48 6.47 6.47 6.47 6.46 5.15 5.14 5.14 5.14 5.12 5.12 5.11 5.11 3.69 3.69 3.69 2.34 1.05 1.04 1.03 1.03 1.03 1.03 1.01 1.01 1.00 1.00 1.00 0.99
Me
H
N
23
1.97
3.97
1.01
1.97
0.98
0.99
0.84
2.00
3.09
4.11
.5 9.0 8.5 8.0 7.5 7.0 6.5 6.0 5.5 5.0 4.5 4.0 3.5 3.0 2.5 2.0 1.5 1.0 0.5 0.0
f1 (ppm)

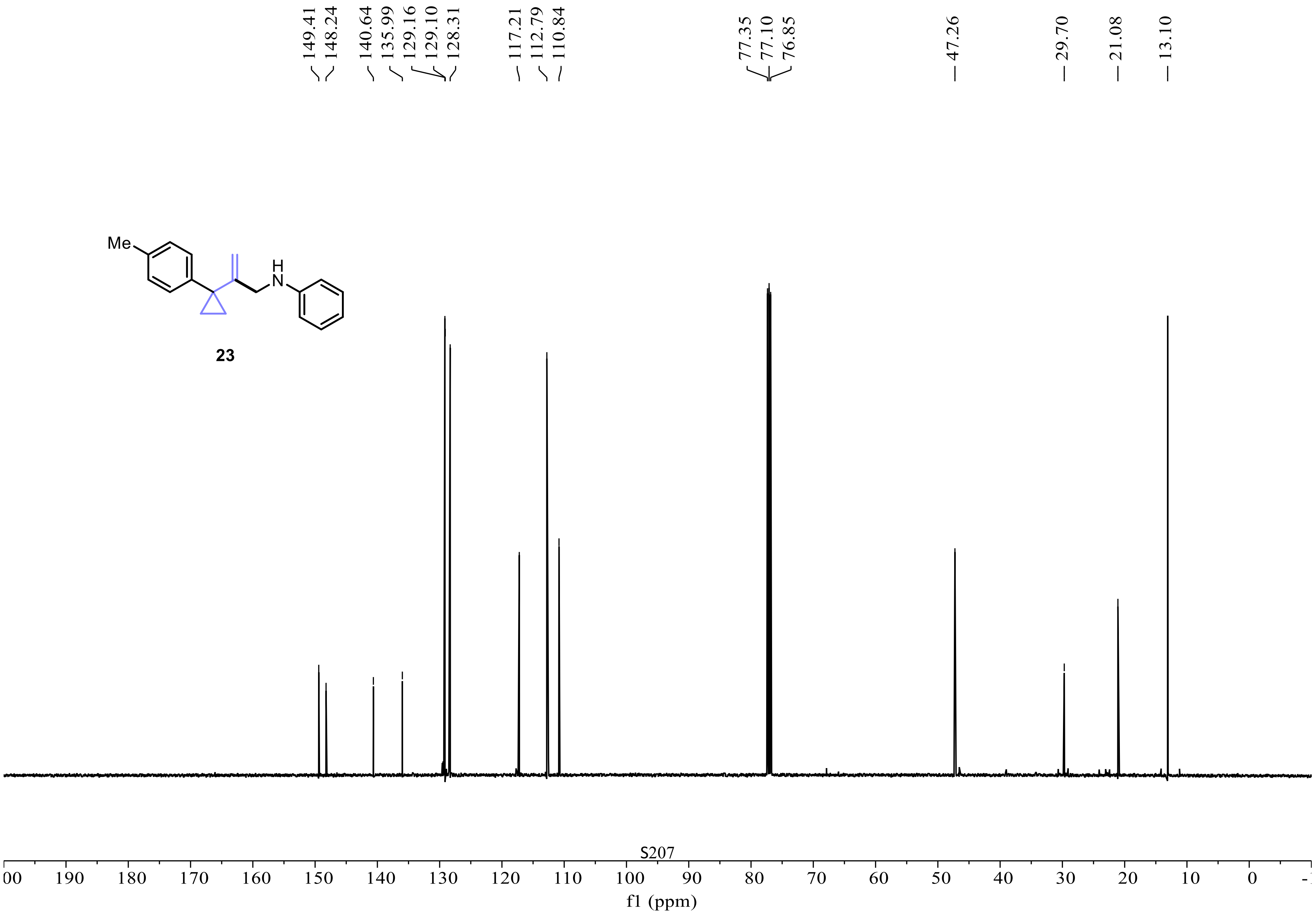
149.41
148.24
140.64
135.99
129.16
129.10
128.31
117.21
112.79
110.84
77.35
77.10
76.85
47.26
29.70
21.08
13.10
Me
H
N
23
00 190 180 170 160 150 140 130 120 110 100 90 80 70 60 50 40 30 20 10 0
f1 (ppm)

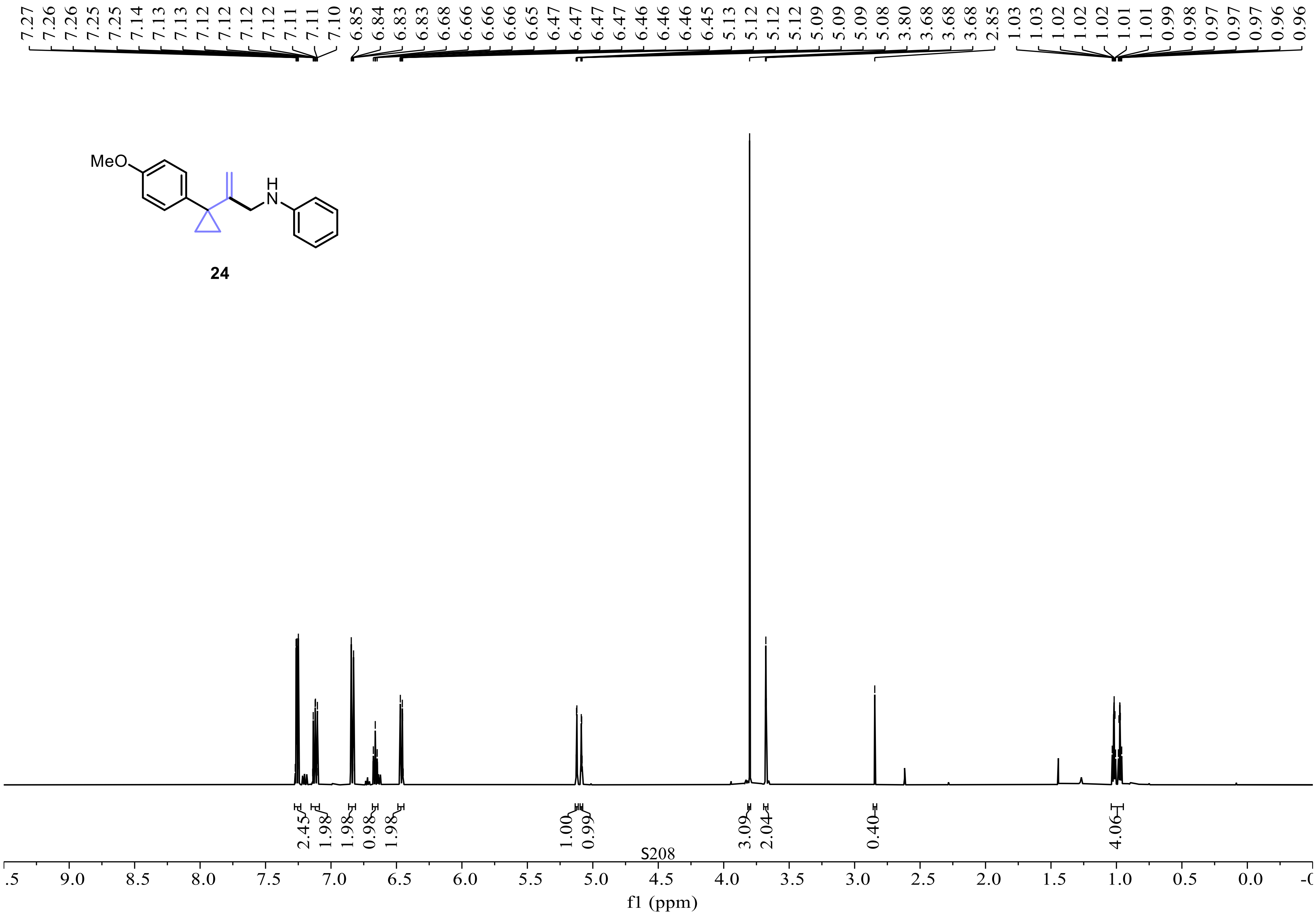
MeO
H
N
24
7.27
7.26
7.26
7.25
7.25
7.14
7.13
7.13
7.12
7.12
7.12
7.12
7.11
7.11
7.10
6.85
6.84
6.83
6.83
6.68
6.66
6.66
6.66
6.65
6.47
6.47
6.47
6.46
6.46
6.46
6.45
5.13
5.12
5.12
5.09
5.09
5.09
5.08
3.80
3.68
3.68
3.68
2.85
1.03
1.03
1.02
1.02
1.02
1.01
1.01
0.99
0.98
0.97
0.97
0.97
0.96
0.96
2.45
1.98
1.98
0.98
1.98
1.00
0.99
3.09
2.04
0.40
4.06
f1 (ppm)

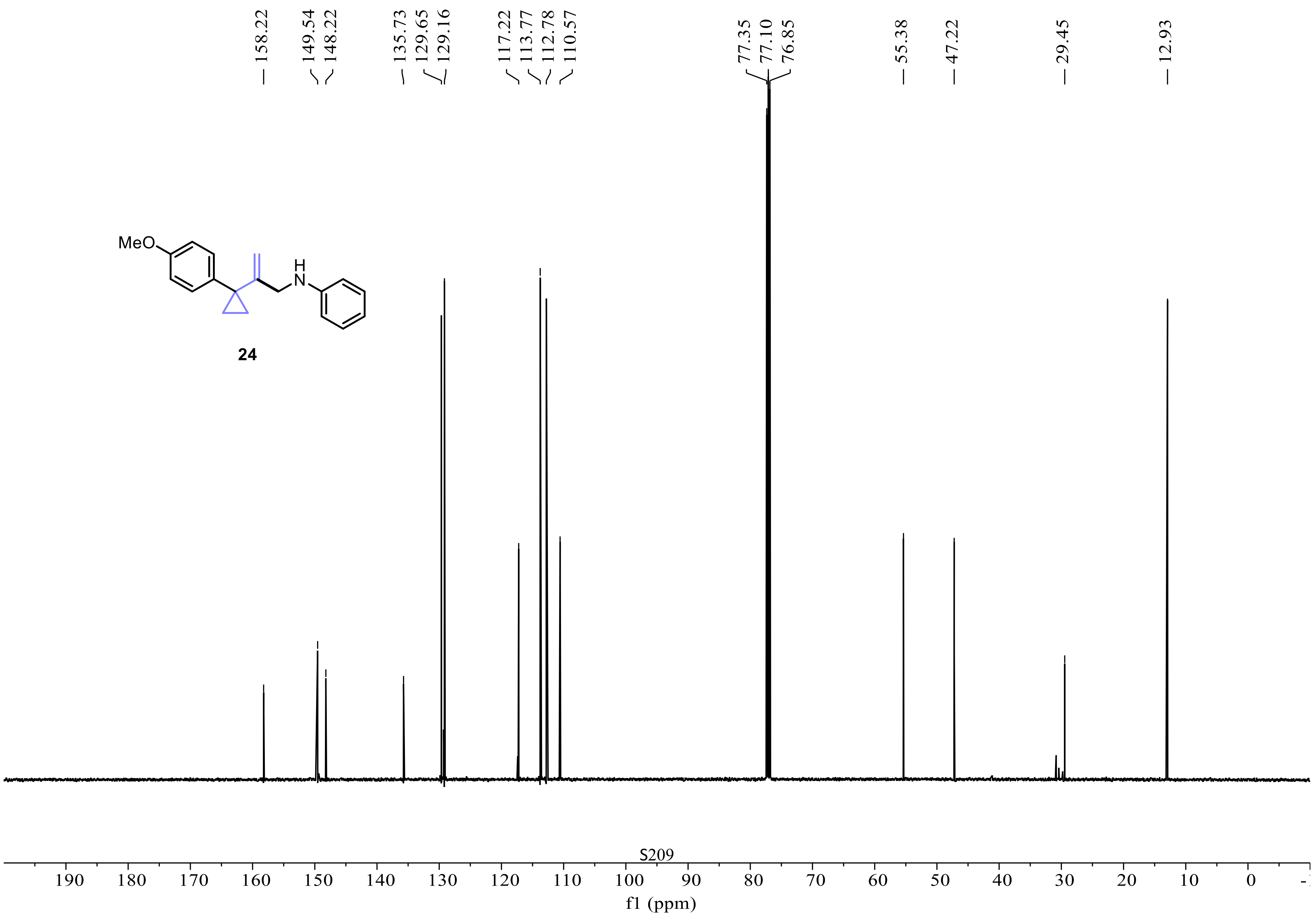
158.22
149.54
148.22
135.73
129.65
129.16
117.22
113.77
112.78
110.57
77.35
77.10
76.85
55.38
47.22
29.45
12.93
MeO
H
N
24
190
180
170
160
150
140
130
120
110
100
90
80
70
60
50
40
30
20
10
0
f1 (ppm)

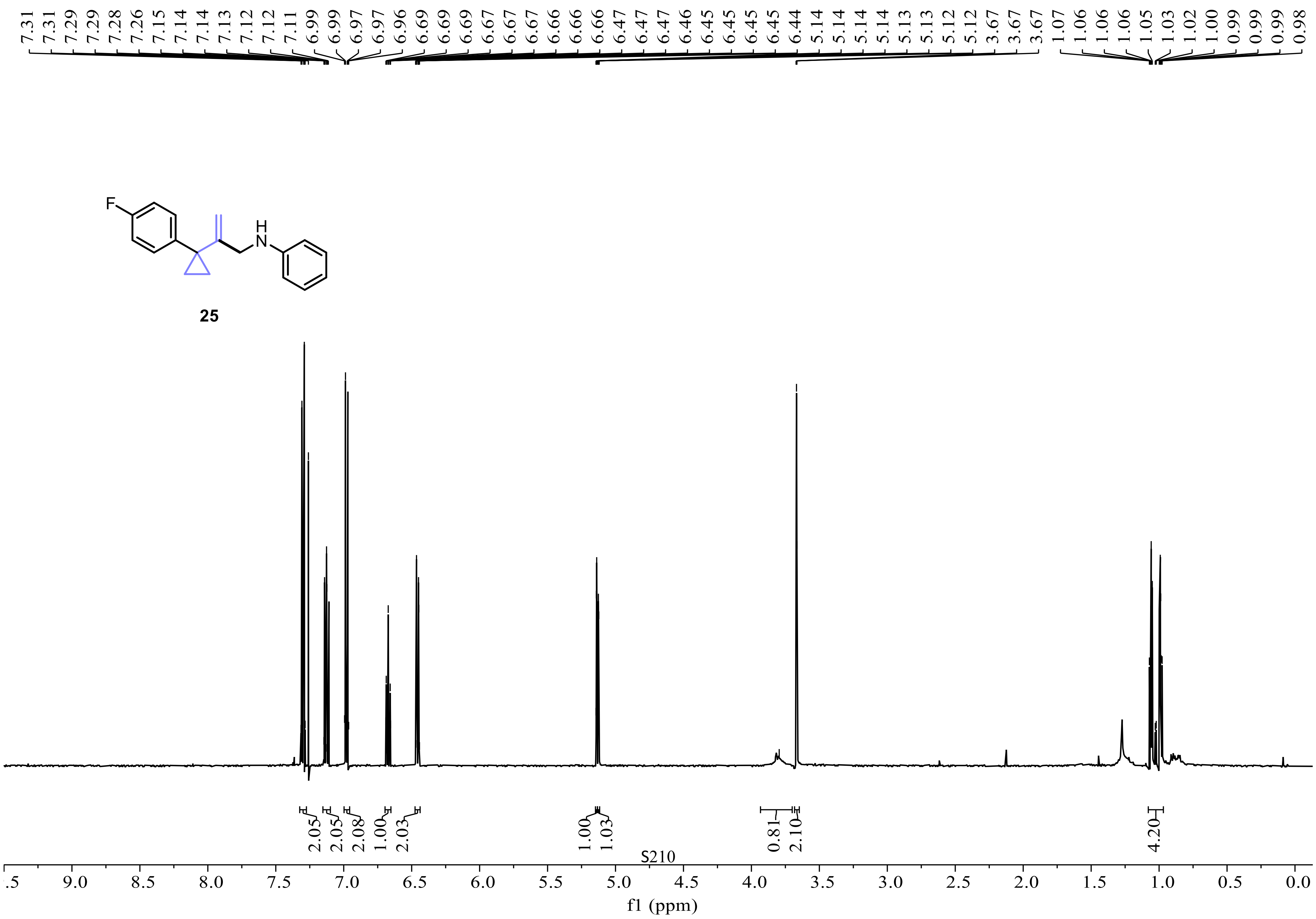
7.31
7.31
7.29
7.29
7.28
7.26
7.15
7.14
7.14
7.13
7.12
7.12
7.11
6.99
6.99
6.97
6.97
6.96
6.69
6.69
6.69
6.67
6.67
6.67
6.66
6.66
6.66
6.47
6.47
6.47
6.46
6.45
6.45
6.45
6.45
6.44
5.14
5.14
5.14
5.14
5.13
5.13
5.12
5.12
3.67
3.67
3.67
1.07
1.06
1.06
1.06
1.05
1.03
1.02
1.00
0.99
0.99
0.99
0.98
F
H
N
25
2.05
2.05
2.08
1.00
2.03
1.00
1.03
0.81
2.10
4.20
f1 (ppm)

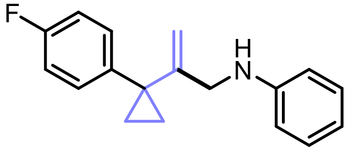
F
H
N

25

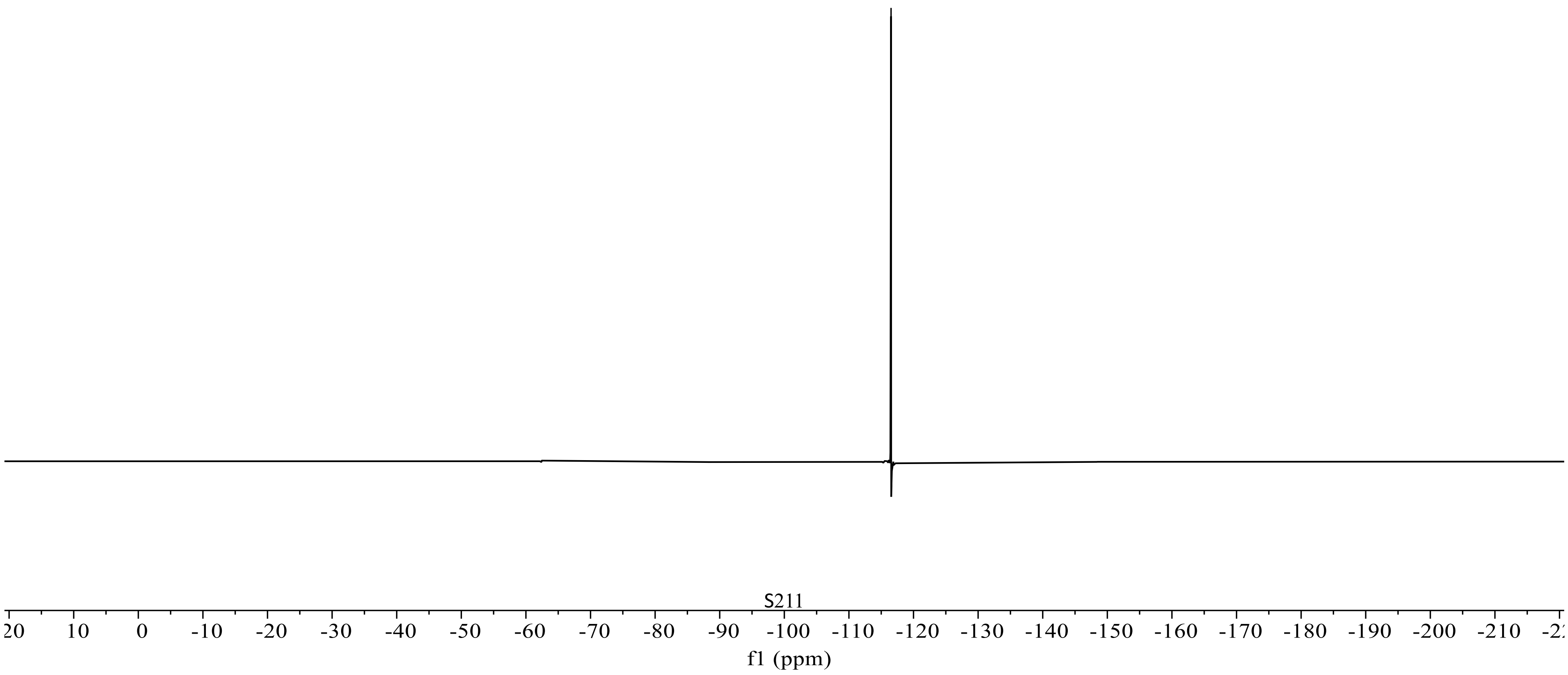
-116.52
20
10
0
-10
-20
-30
-40
-50
-60
-70
-80
-90
-100
-110
-120
-130
-140
-150
-160
-170
-180
-190
-200
-210
f1 (ppm)

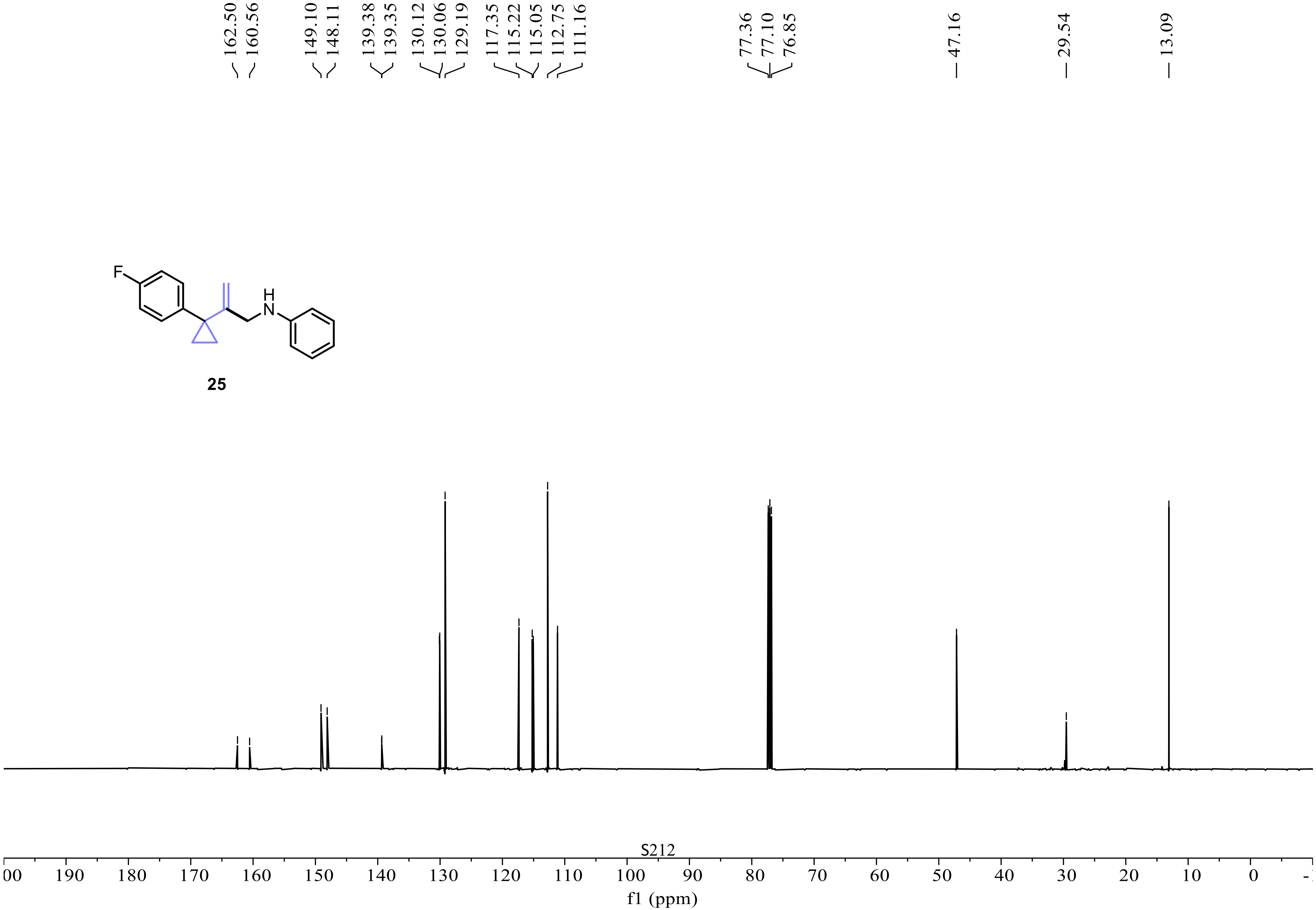
162.50
160.56
149.10
148.11
139.38
139.35
130.12
130.06
129.19
117.35
115.22
115.05
112.75
111.16
77.36
77.10
76.85
47.16
29.54
13.09
F
H
N
25
00 190 180 170 160 150 140 130 120 110 100 90 80 70 60 50 40 30 20 10 0
f1 (ppm)

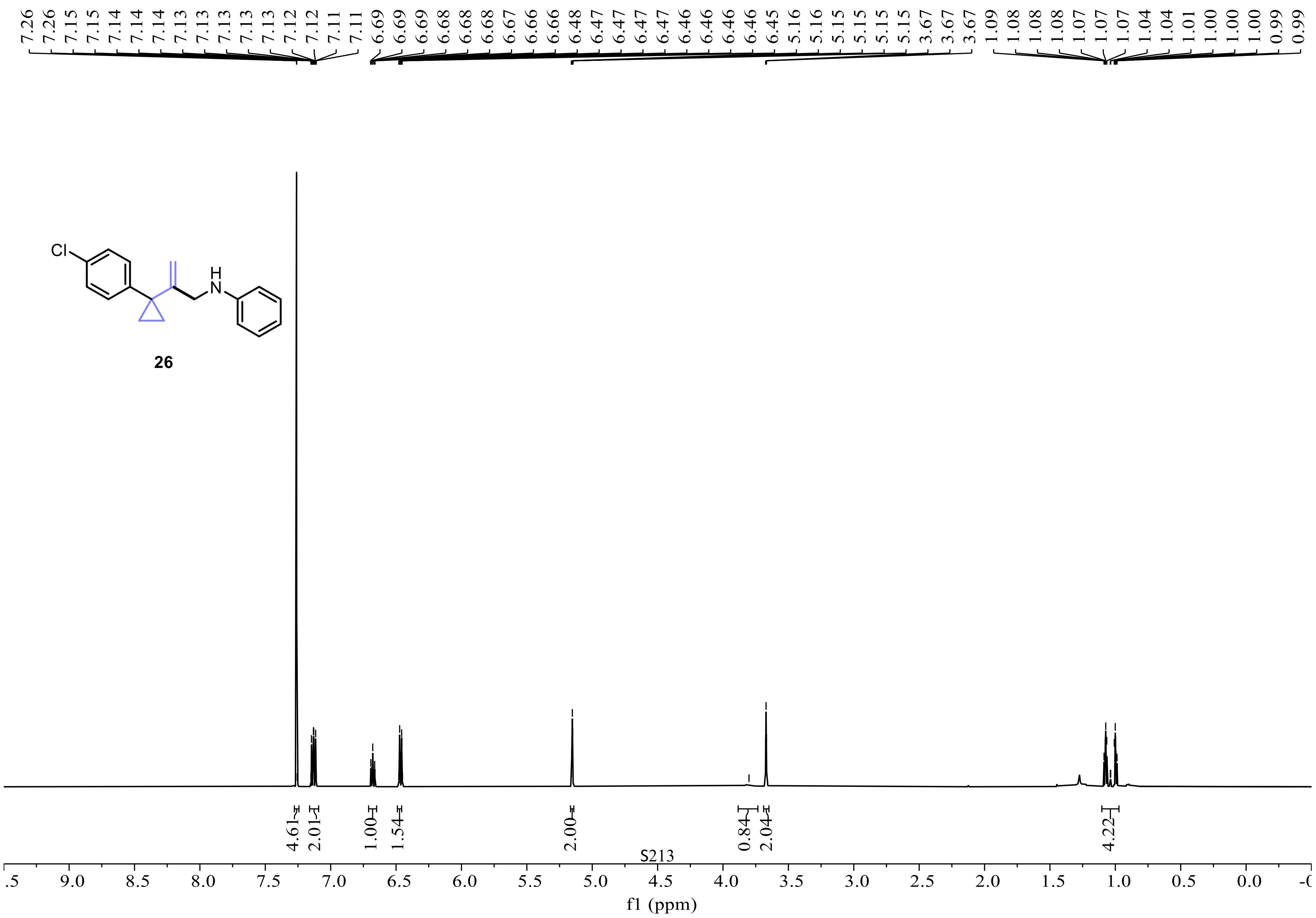
7.26 7.26 7.15 7.15 7.14 7.14 7.14 7.13 7.13 7.13 7.13 7.13 7.12 7.12 7.11 7.11 6.69 6.69 6.69 6.68 6.68 6.68 6.67 6.66 6.66 6.48 6.47 6.47 6.47 6.47 6.46 6.46 6.46 6.46 6.45 5.16 5.16 5.15 5.15 5.15 5.15 3.67 3.67 3.67 1.09 1.08 1.08 1.08 1.07 1.07 1.07 1.04 1.04 1.01 1.00 1.00 1.00 0.99 0.99
Cl
H
N
26
4.61
2.01
1.00
1.54
2.00
0.84
2.04
4.22
.5 9.0 8.5 8.0 7.5 7.0 6.5 6.0 5.5 5.0 4.5 4.0 3.5 3.0 2.5 2.0 1.5 1.0 0.5 0.0
f1 (ppm)

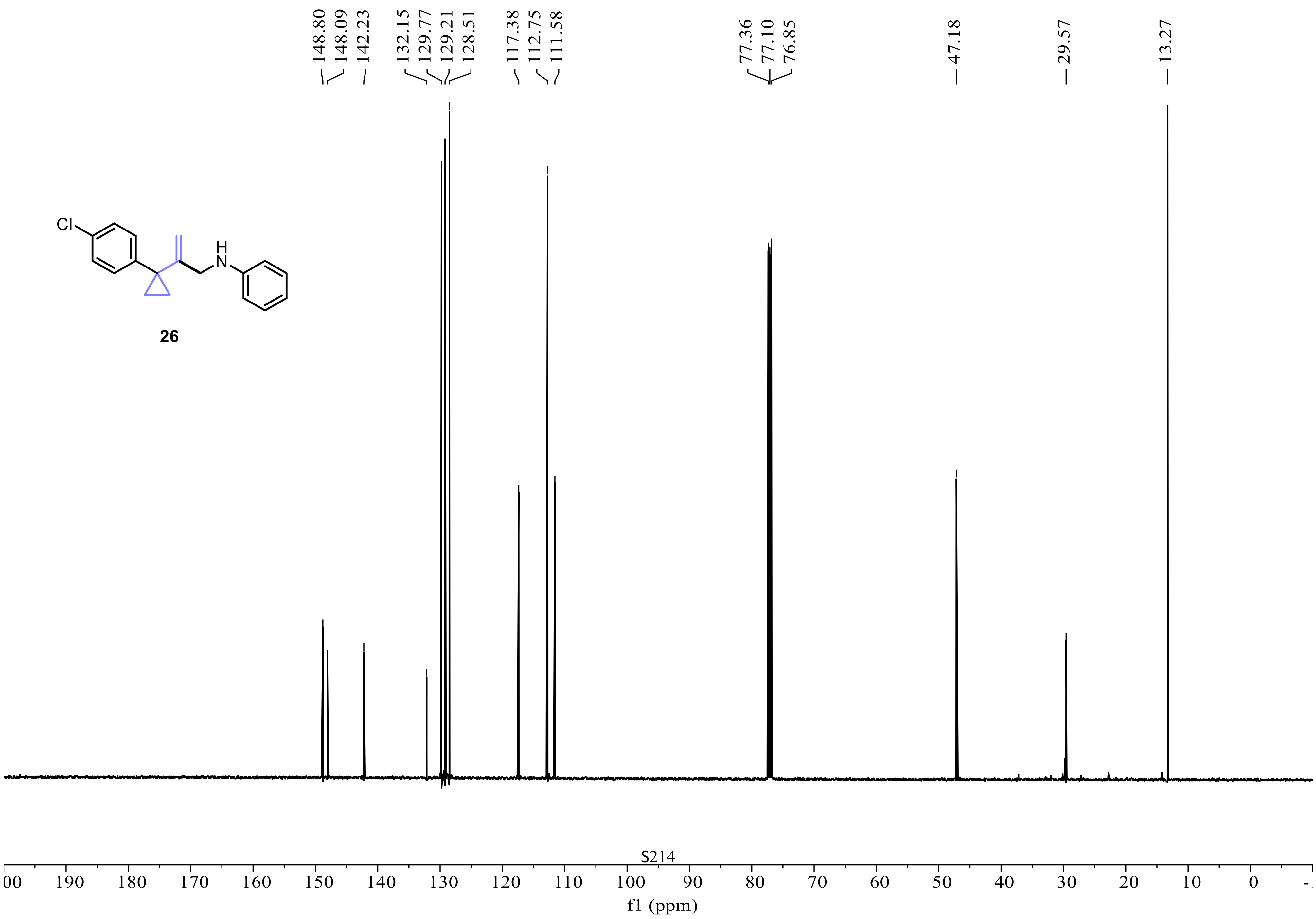
148.80
148.09
142.23
132.15
129.77
129.21
128.51
117.38
112.75
111.58
77.36
77.10
76.85
47.18
29.57
13.27
Cl
H
N
26
190
180
170
160
150
140
130
120
110
100
90
80
70
60
50
40
30
20
10
0
f1 (ppm)

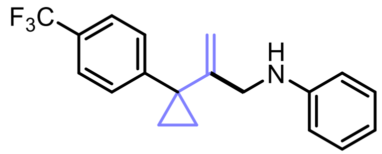
F3C
H
N

27

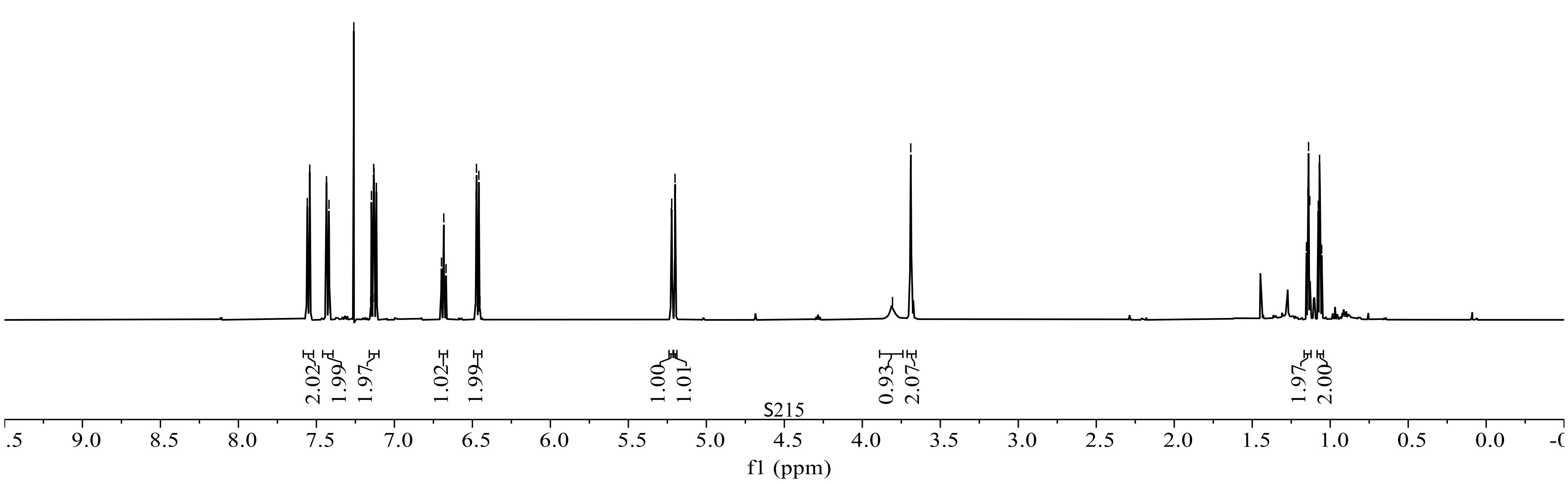
7.56 7.56 7.56 7.55 7.54 7.54 7.44 7.44 7.43 7.42 7.42 7.42 7.42 7.26 7.15 7.15 7.14 7.13 7.13 7.12 7.12 7.12 6.70 6.70 6.69 6.68 6.68 6.67 6.48 6.47 6.47 6.47 6.46 6.46 6.46 6.46 5.23 5.22 5.22 5.22 5.20 5.20 5.20 5.20 3.69 3.69 3.69 1.15 1.15 1.14 1.14 1.14 1.13 1.08 1.07 1.07 1.07 1.06 1.05
2.02 1.99 1.97 1.02 1.99 1.00 1.01 0.93 2.07 1.97 2.00
.5 9.0 8.5 8.0 7.5 7.0 6.5 6.0 5.5 5.0 4.5 4.0 3.5 3.0 2.5 2.0 1.5 1.0 0.5 0.0
f1 (ppm)

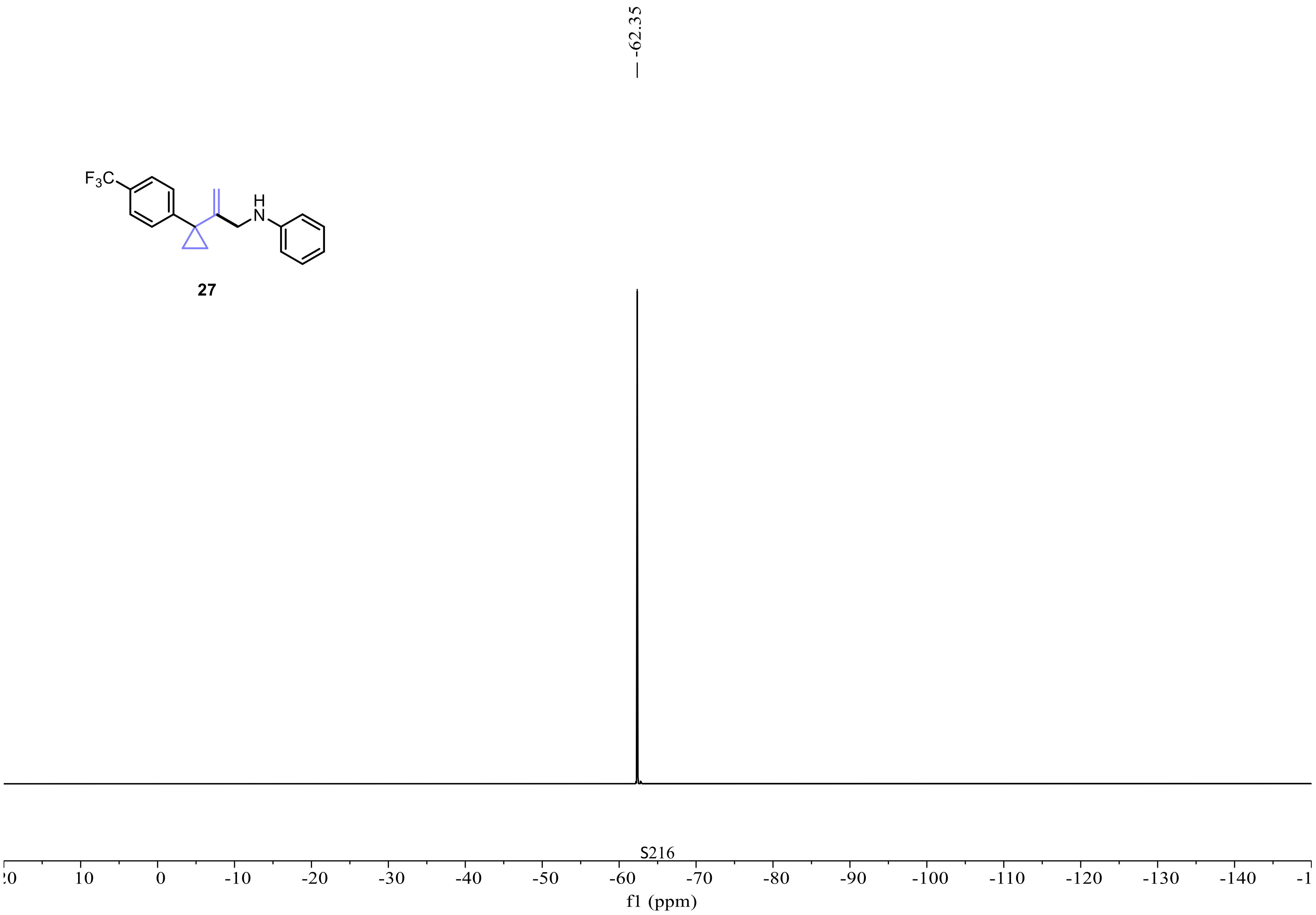
-62.35
F3C
H
N
27
20
10
0
-10
-20
-30
-40
-50
-60
-70
-80
-90
-100
-110
-120
-130
-140
-1
f1 (ppm)

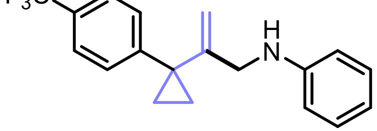
F3C
H
N

27

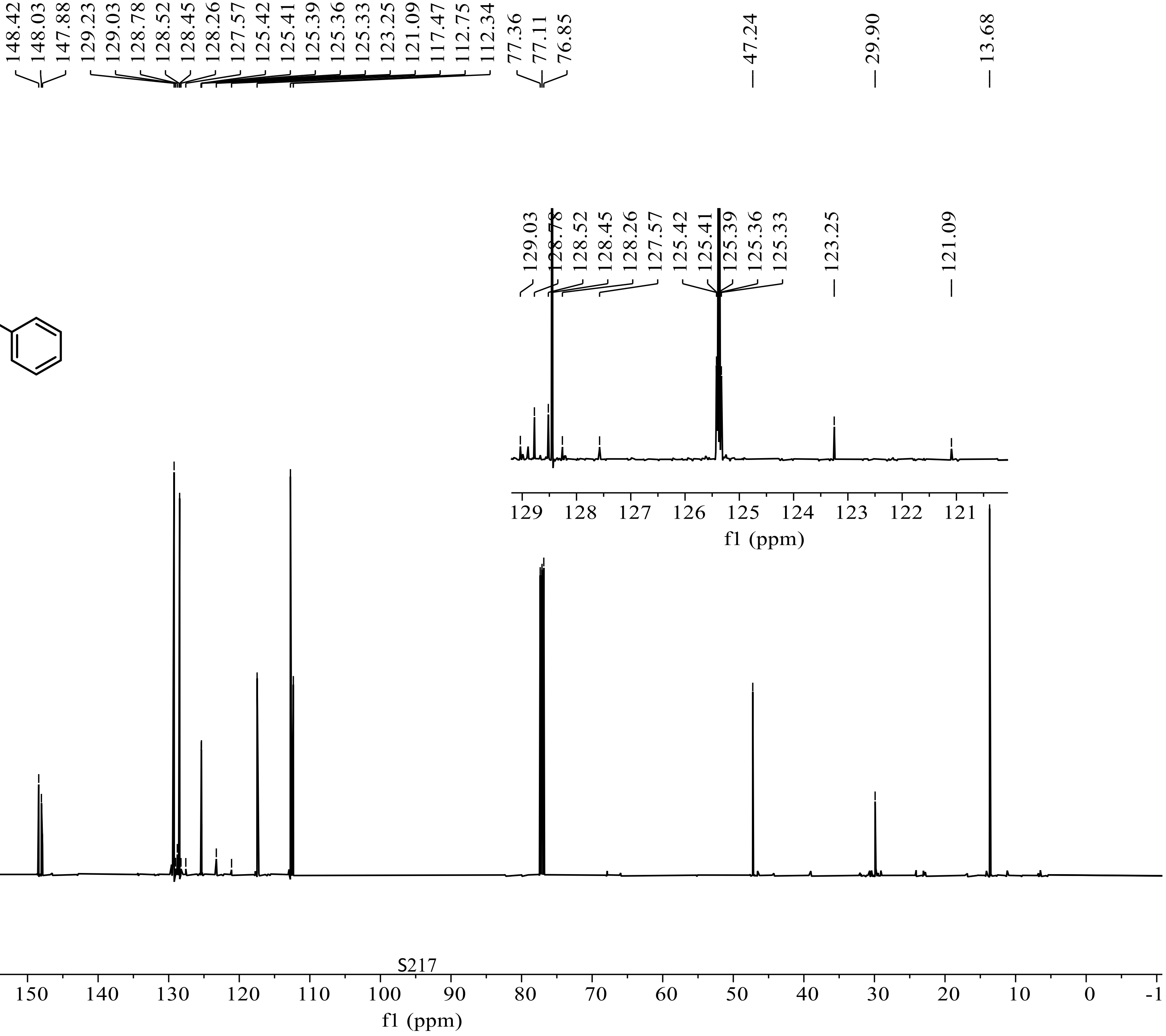
148.42
148.03
147.88
129.23
129.03
128.78
128.52
128.45
128.26
127.57
125.42
125.41
125.39
125.36
125.33
123.25
121.09
117.47
112.75
112.34
77.36
77.11
76.85
47.24
29.90
13.68
129.03
128.78
128.52
128.45
128.26
127.57
125.42
125.41
125.39
125.36
125.33
123.25
121.09
129 128 127 126 125 124 123 122 121
f1 (ppm)
00 190 180 170 160 150 140 130 120 110 100 90 80 70 60 50 40 30 20 10 0 -1
f1 (ppm)

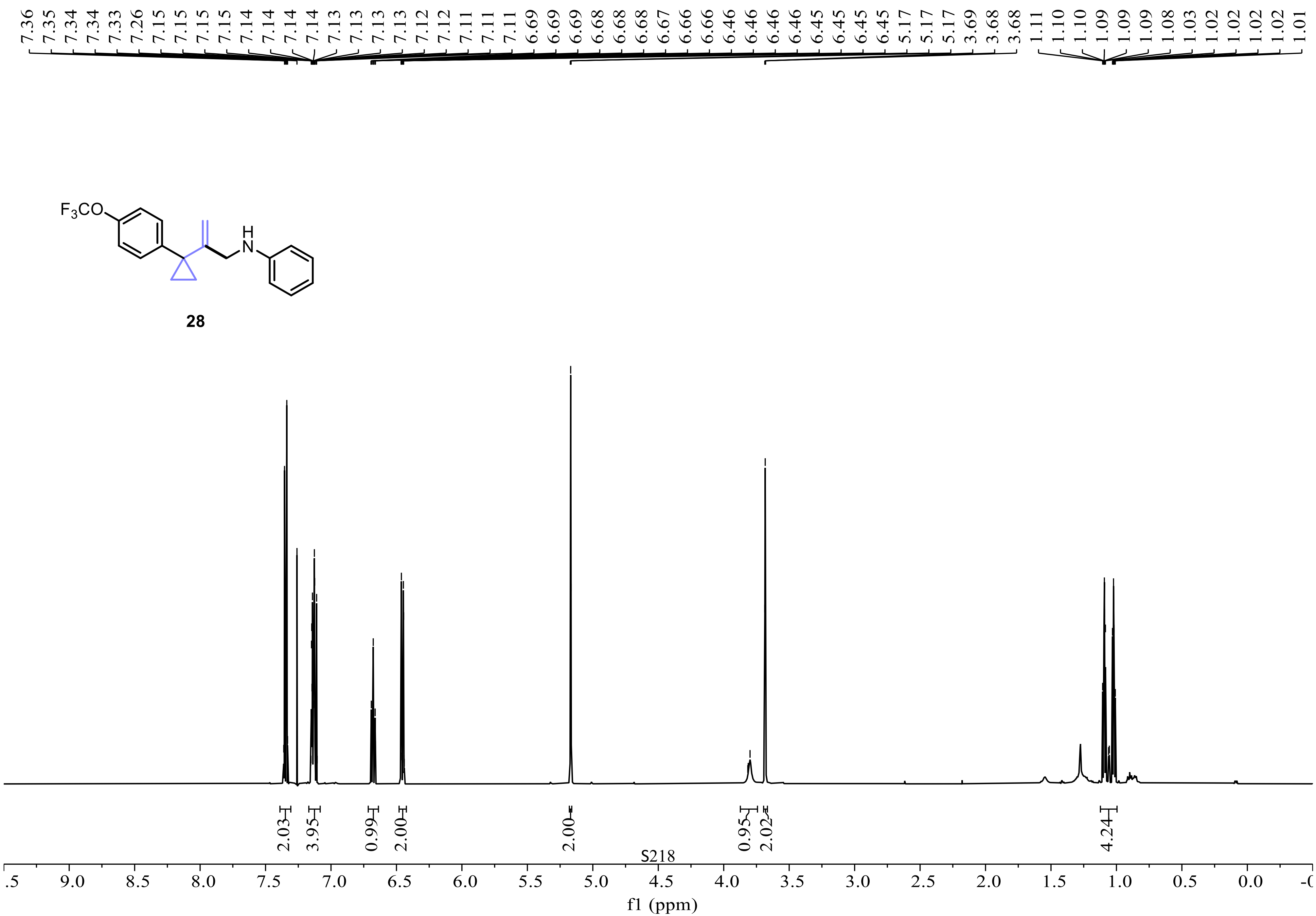
7.36 7.35 7.34 7.34 7.33 7.26 7.15 7.15 7.15 7.15 7.14 7.14 7.14 7.14 7.13 7.13 7.13 7.13 7.12 7.12 7.11 7.11 7.11 6.69 6.69 6.69 6.68 6.68 6.68 6.67 6.66 6.66 6.46 6.46 6.46 6.46 6.45 6.45 6.45 6.45 5.17 5.17 5.17 3.69 3.68 3.68 1.11 1.10 1.10 1.09 1.09 1.09 1.08 1.03 1.02 1.02 1.02 1.02 1.01
F3CO
H
N
28
2.03
3.95
0.99
2.00
2.00
0.95
2.02
4.24
.5 9.0 8.5 8.0 7.5 7.0 6.5 6.0 5.5 5.0 4.5 4.0 3.5 3.0 2.5 2.0 1.5 1.0 0.5 0.0 -(
f1 (ppm)

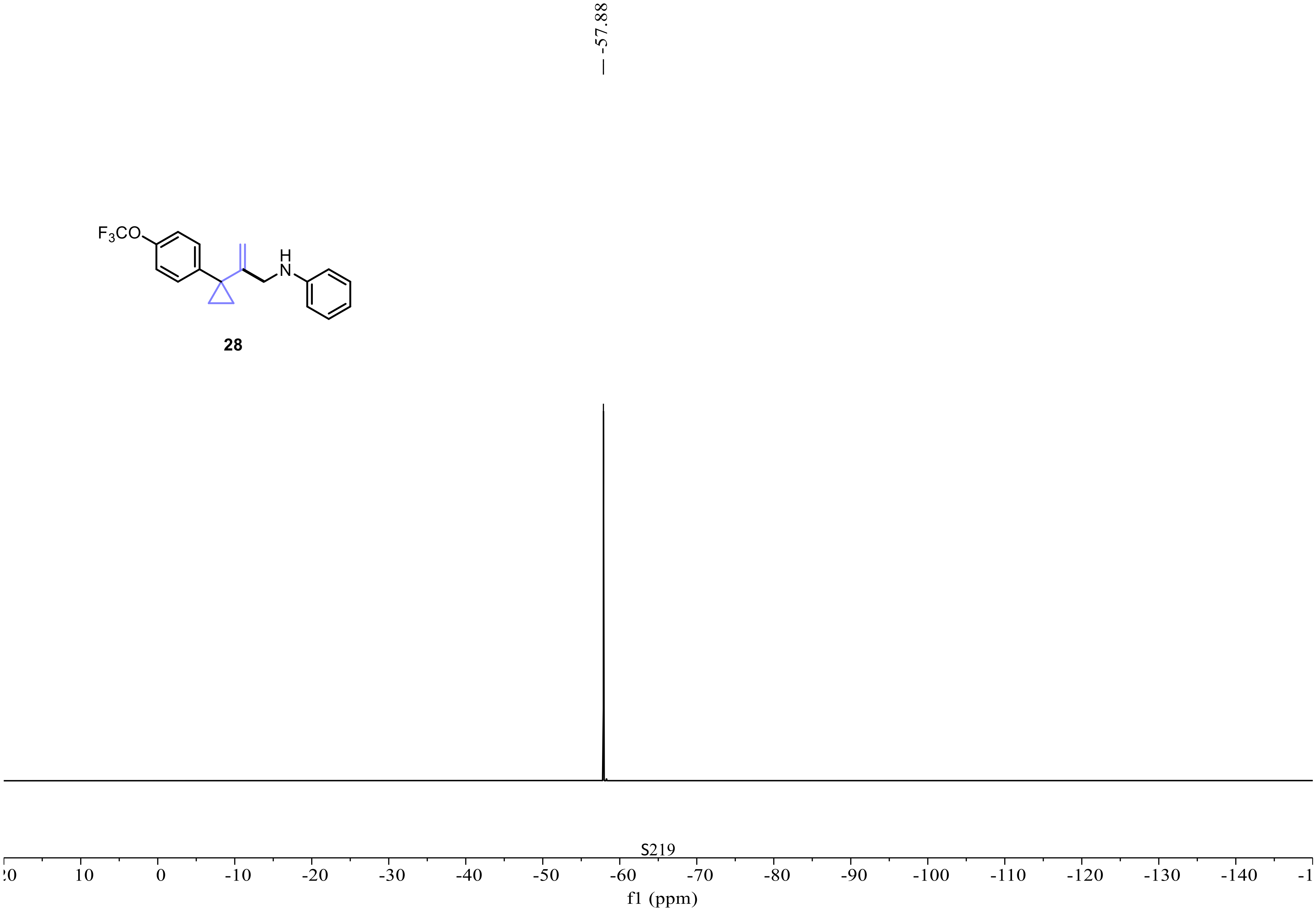
-57.88
F3CO
H
N
28
20
10
0
-10
-20
-30
-40
-50
-60
-70
-80
-90
-100
-110
-120
-130
-140
f1 (ppm)

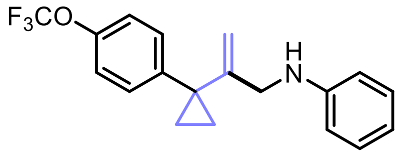
F3CO
H
N

28

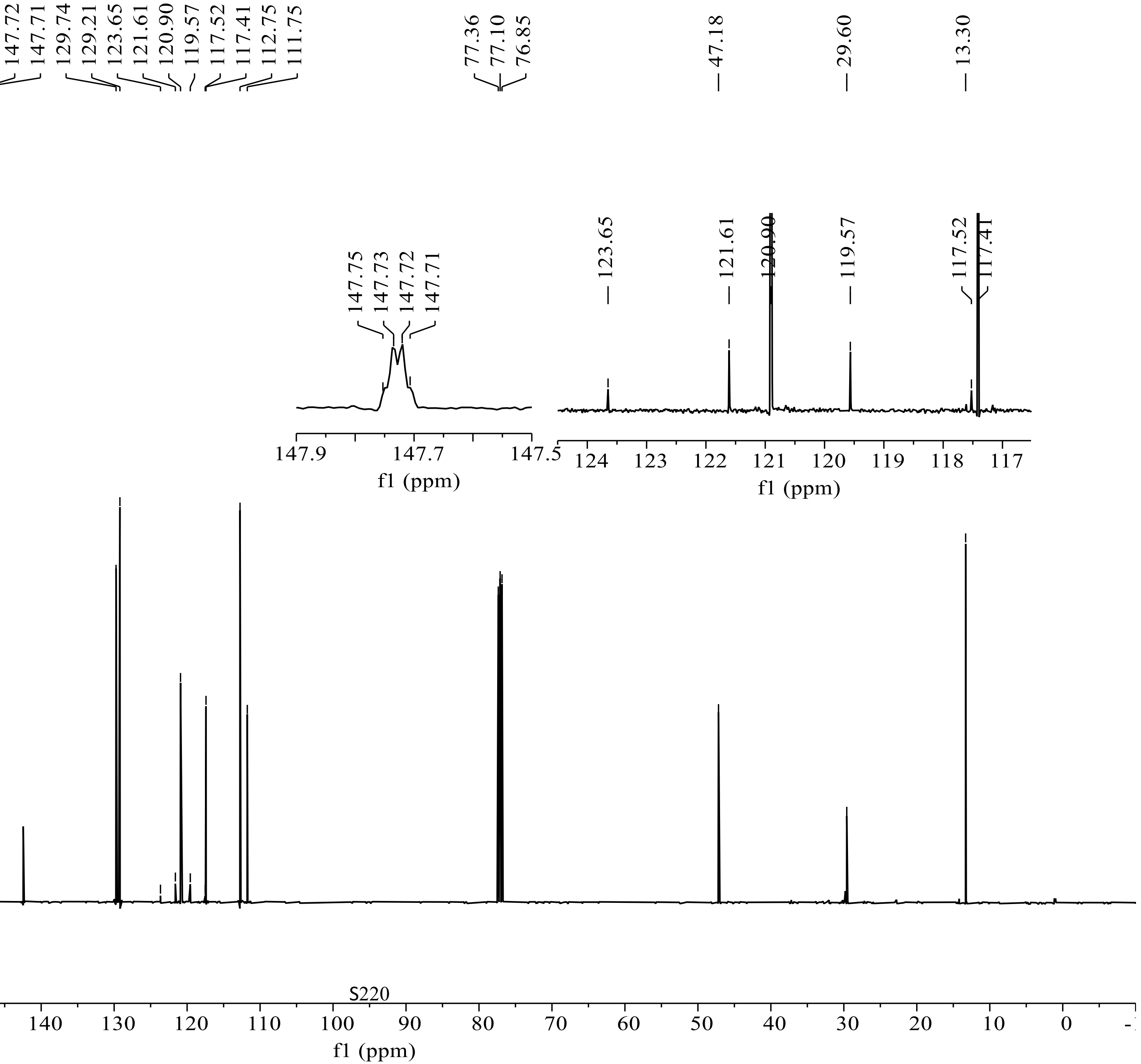
148.78
148.07
147.75
147.73
147.72
147.71
129.74
129.21
123.65
121.61
120.90
119.57
117.52
117.41
112.75
111.75
77.36
77.10
76.85
47.18
29.60
13.30
147.9
147.7
147.5
f1 (ppm)
123.65
121.61
120.90
119.57
117.52
117.41
124
123
122
121
120
119
118
117
f1 (ppm)
190
180
170
160
150
140
130
120
110
100
90
80
70
60
50
40
30
20
10
0
f1 (ppm)

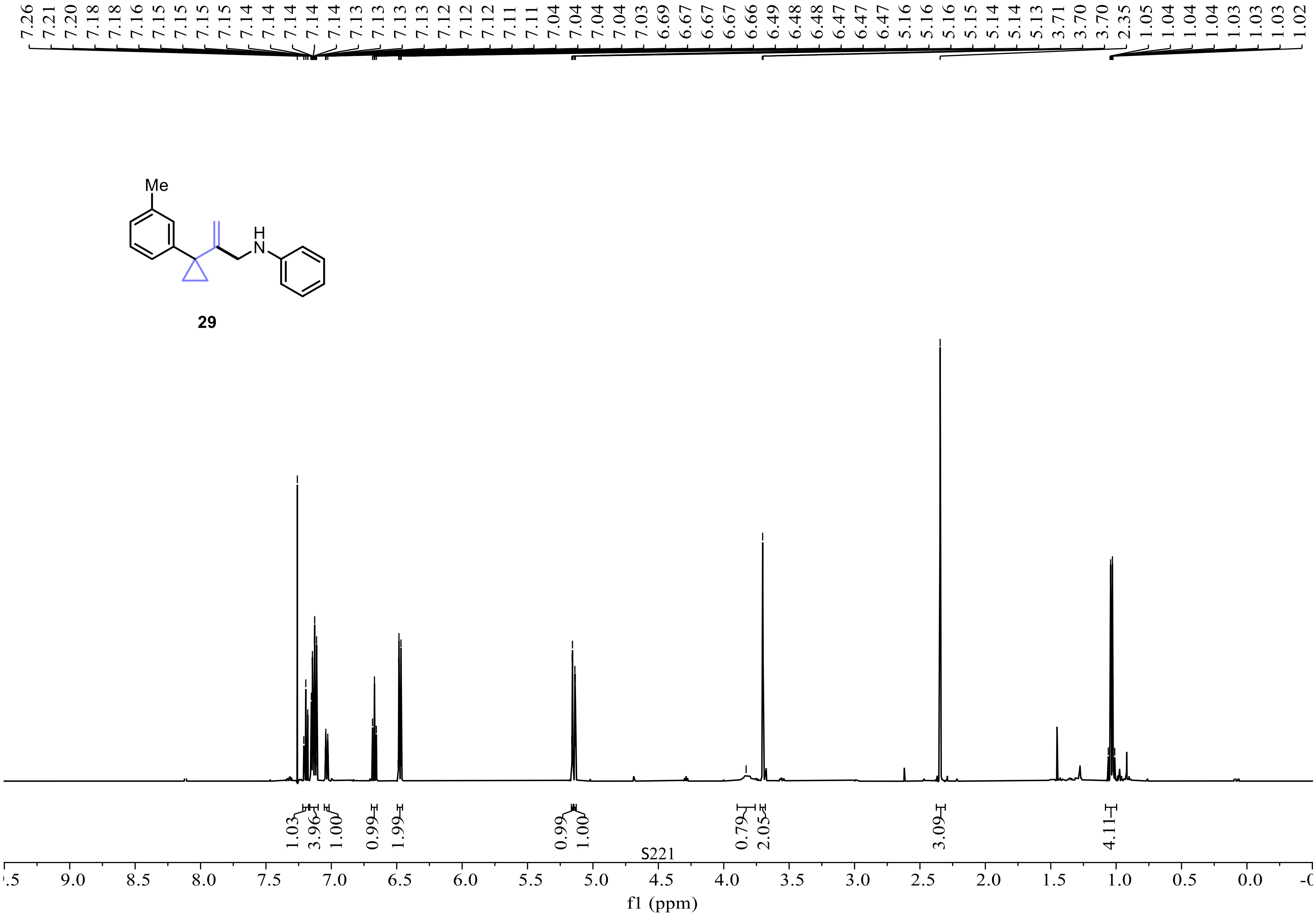
7.26 7.21 7.20 7.18 7.18 7.16 7.15 7.15 7.15 7.15 7.14 7.14 7.14 7.14 7.14 7.13 7.13 7.13 7.13 7.12 7.12 7.12 7.11 7.11 7.04 7.04 7.04 7.03 6.69 6.67 6.67 6.67 6.66 6.49 6.48 6.48 6.47 6.47 6.47 5.16 5.16 5.16 5.15 5.14 5.14 5.13 3.71 3.70 3.70 2.35 1.05 1.04 1.04 1.04 1.03 1.03 1.03 1.02
Me
H
N
29
1.03
3.96
1.00
0.99
1.99
0.99
1.00
0.79
2.05
3.09
4.11
9.0 8.5 8.0 7.5 7.0 6.5 6.0 5.5 5.0 4.5 4.0 3.5 3.0 2.5 2.0 1.5 1.0 0.5 0.0
f1 (ppm)

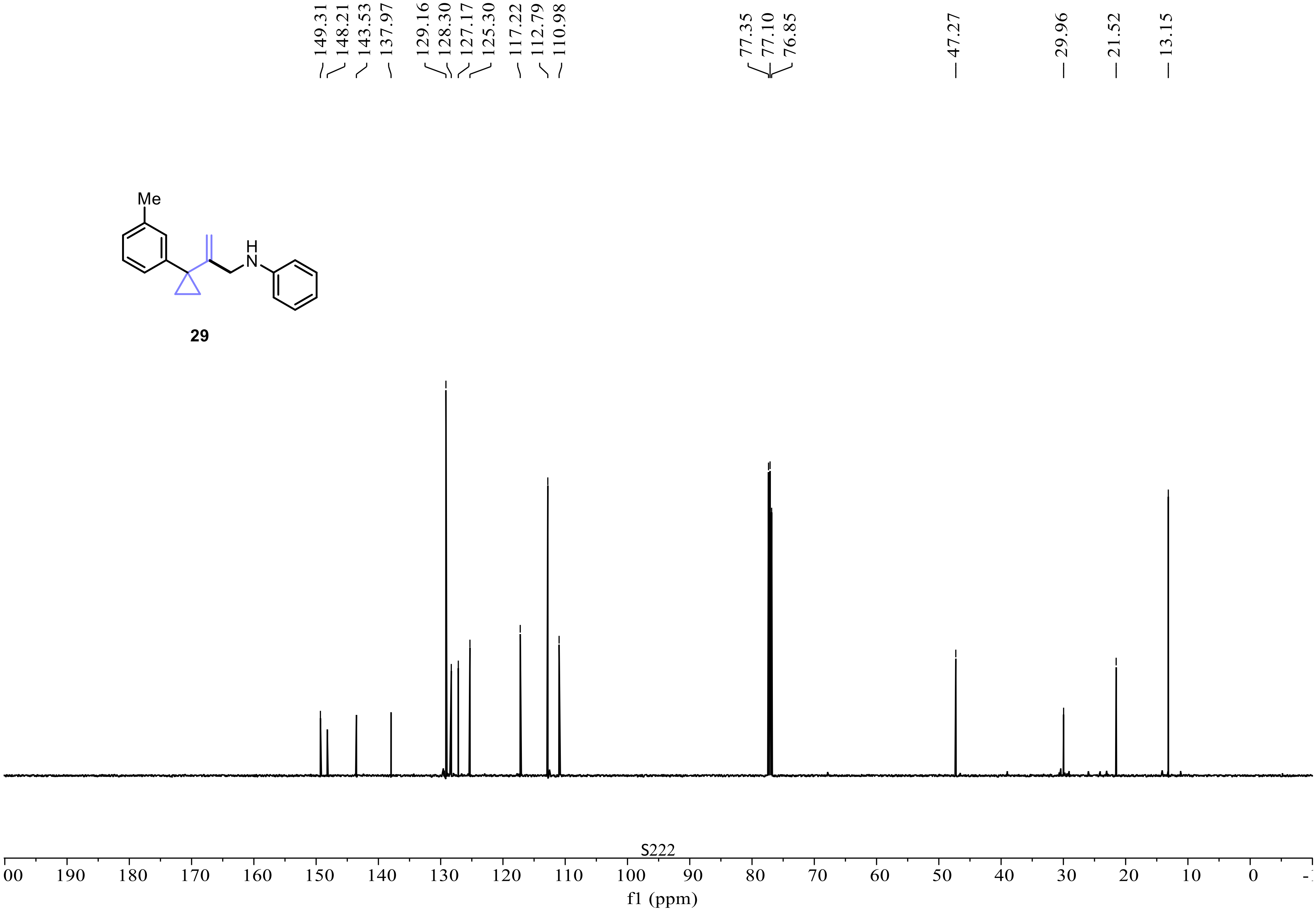
149.31
148.21
143.53
137.97
129.16
128.30
127.17
125.30
117.22
112.79
110.98
77.35
77.10
76.85
47.27
29.96
21.52
13.15
f1 (ppm)
Me
H
N

29

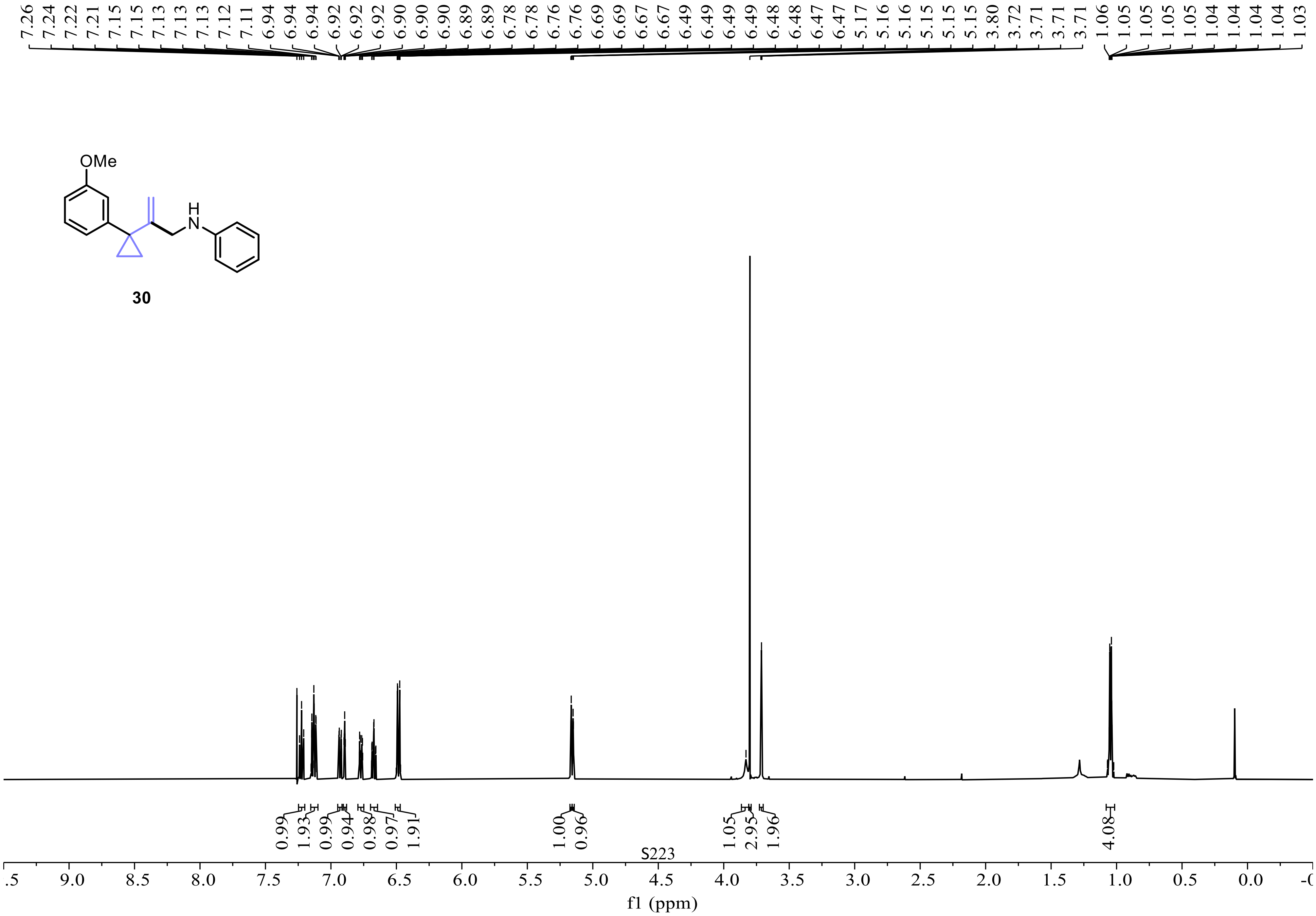

30

7.26
7.24
7.22
7.21
7.15
7.15
7.13
7.13
7.13
7.12
7.11
6.94
6.94
6.94
6.92
6.92
6.92
6.90
6.90
6.90
6.89
6.89
6.78
6.78
6.76
6.76
6.69
6.69
6.67
6.67
6.49
6.49
6.49
6.49
6.48
6.48
6.47
6.47
5.17
5.16
5.16
5.15
5.15
5.15
3.80
3.72
3.71
3.71
3.71
1.06
1.05
1.05
1.05
1.05
1.04
1.04
1.04
1.04
1.03
0.99
1.93
0.99
0.94
0.98
0.97
1.91
1.00
0.96
1.05
2.95
1.96
4.08
f1 (ppm)

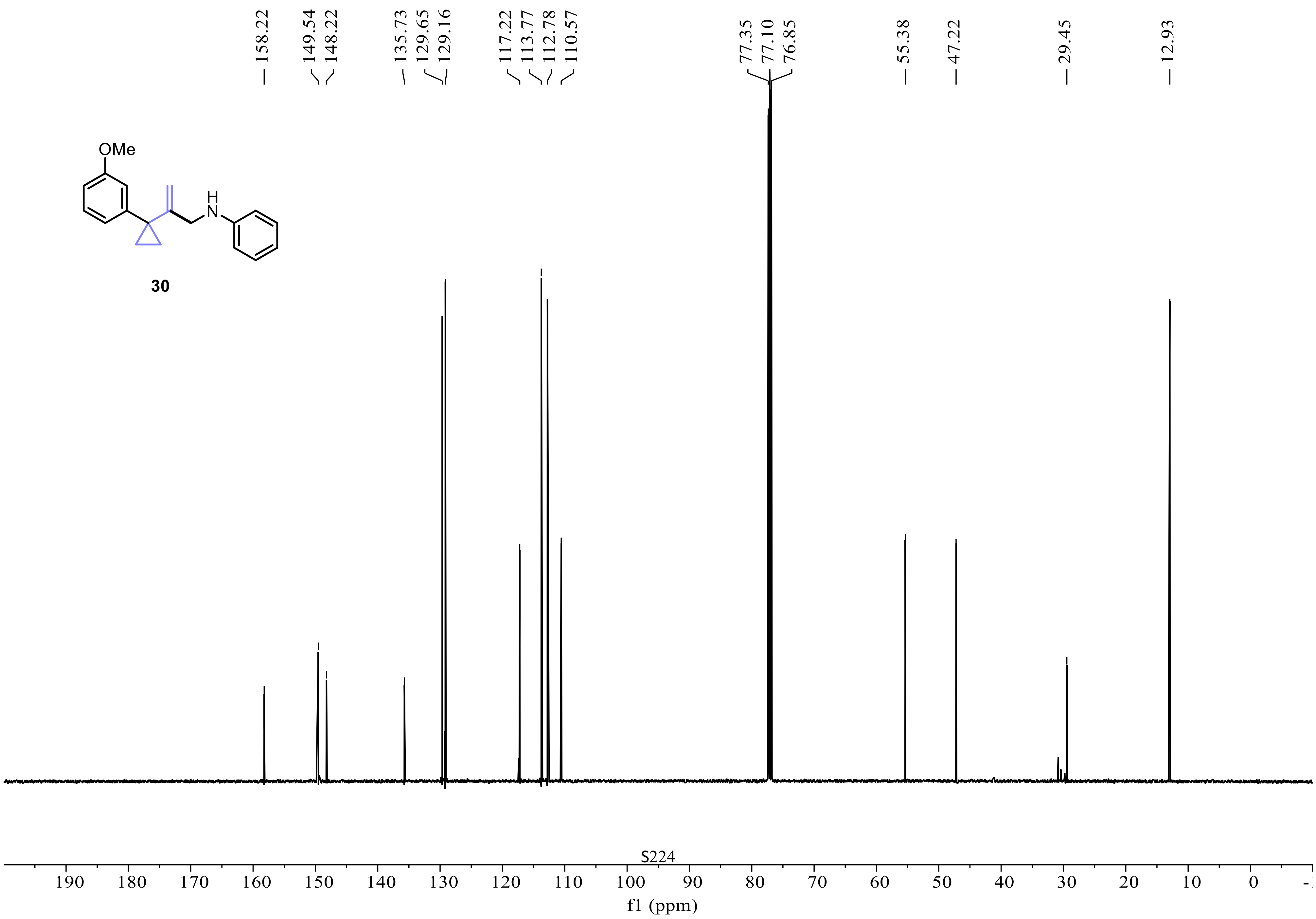

158.22
149.54
148.22
135.73
129.65
129.16
117.22
113.77
112.78
110.57
77.35
77.10
76.85
55.38
47.22
29.45
12.93
OMe
H
N
30
190
180
170
160
150
140
130
120
110
100
90
80
70
60
50
40
30
20
10
0
f1 (ppm)

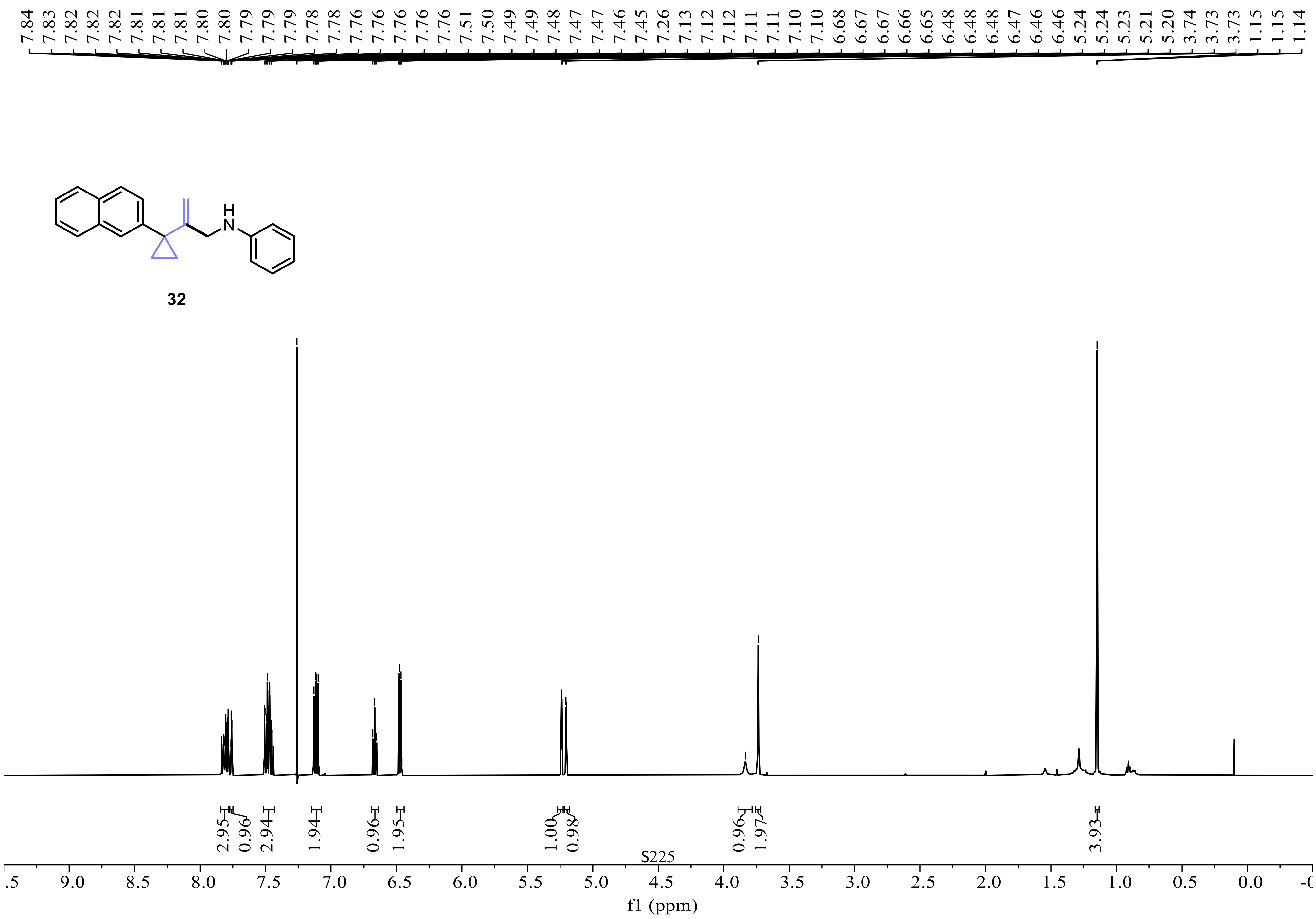

7.84 7.83 7.82 7.82 7.82 7.81 7.81 7.81 7.80 7.80 7.79 7.79 7.79 7.78 7.78 7.76 7.76 7.76 7.76 7.76 7.51 7.50 7.49 7.49 7.48 7.47 7.47 7.46 7.45 7.26 7.13 7.12 7.12 7.11 7.11 7.10 7.10 6.68 6.67 6.67 6.66 6.65 6.48 6.48 6.48 6.47 6.46 6.46 5.24 5.24 5.23 5.21 5.20 3.74 3.73 3.73 1.15 1.15 1.14
H
N
32
2.95 0.96 2.94 1.94 0.96 1.95 1.00 0.98 0.96 1.97 3.93
.5 9.0 8.5 8.0 7.5 7.0 6.5 6.0 5.5 5.0 4.5 4.0 3.5 3.0 2.5 2.0 1.5 1.0 0.5 0.0
f1 (ppm)

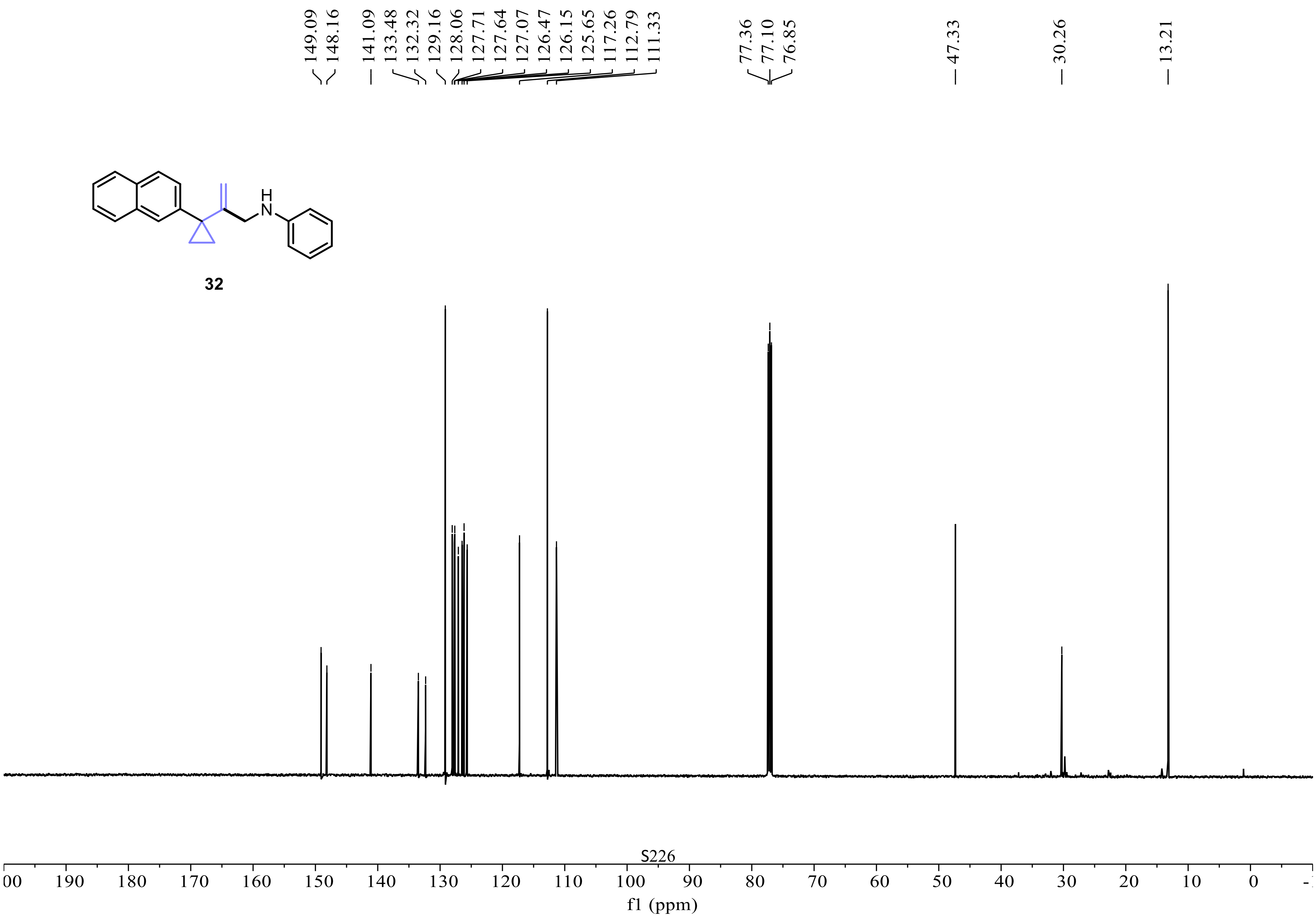
149.09
148.16
141.09
133.48
132.32
129.16
128.06
127.71
127.64
127.07
126.47
126.15
125.65
117.26
112.79
111.33
77.36
77.10
76.85
47.33
30.26
13.21
H
N
32
f1 (ppm)

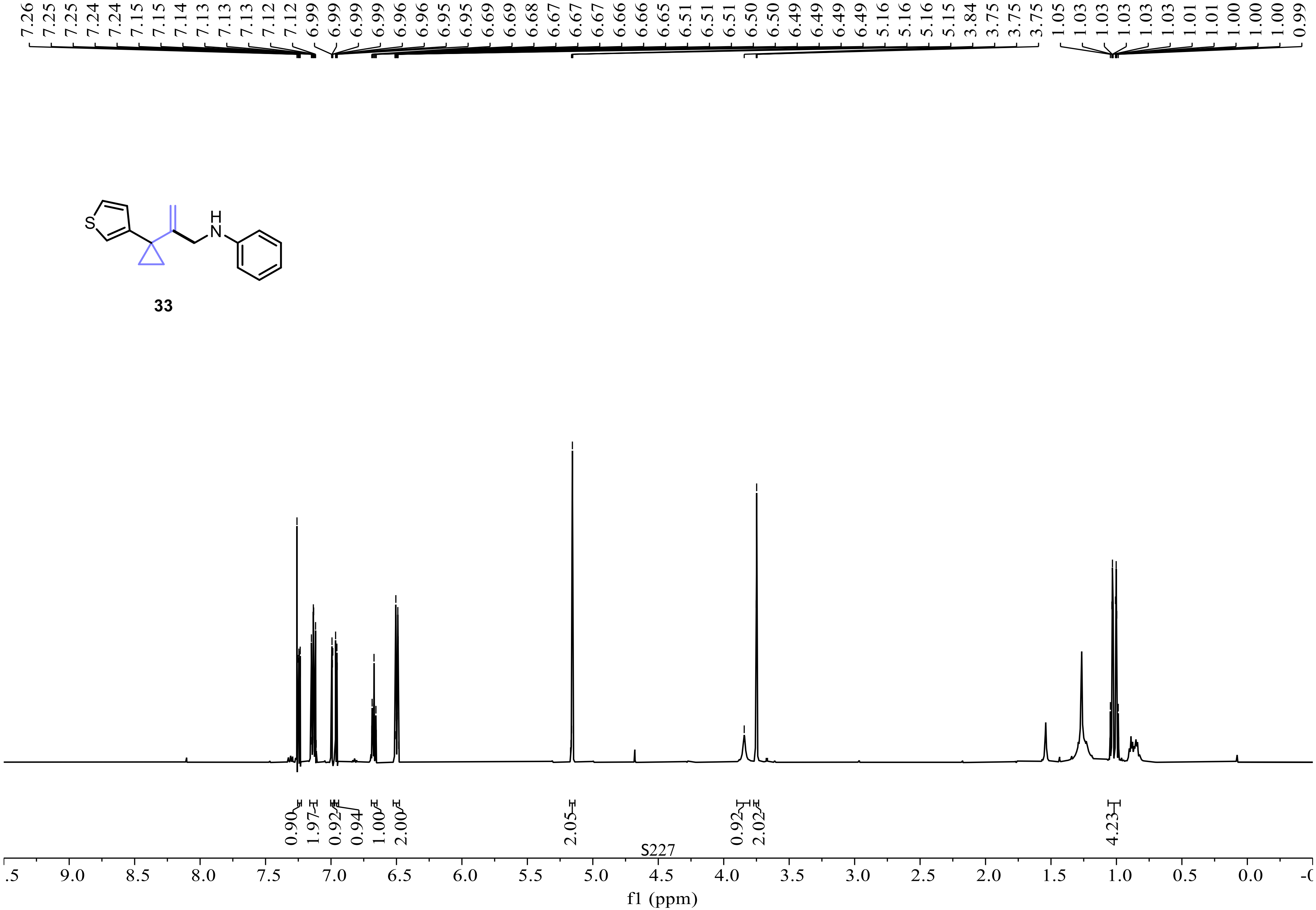
7.26 7.25 7.25 7.24 7.24 7.15 7.15 7.14 7.13 7.13 7.13 7.12 7.12 6.99 6.99 6.99 6.99 6.96 6.96 6.95 6.95 6.69 6.69 6.68 6.67 6.67 6.67 6.66 6.66 6.65 6.51 6.51 6.51 6.50 6.50 6.49 6.49 6.49 6.49 5.16 5.16 5.16 5.15 3.84 3.75 3.75 3.75 1.05 1.03 1.03 1.03 1.03 1.03 1.01 1.01 1.00 1.00 1.00 0.99
33
0.90 1.97 0.92 0.94 1.00 2.00 2.05 0.92 2.02 4.23
f1 (ppm)

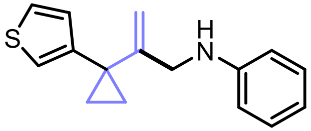

33

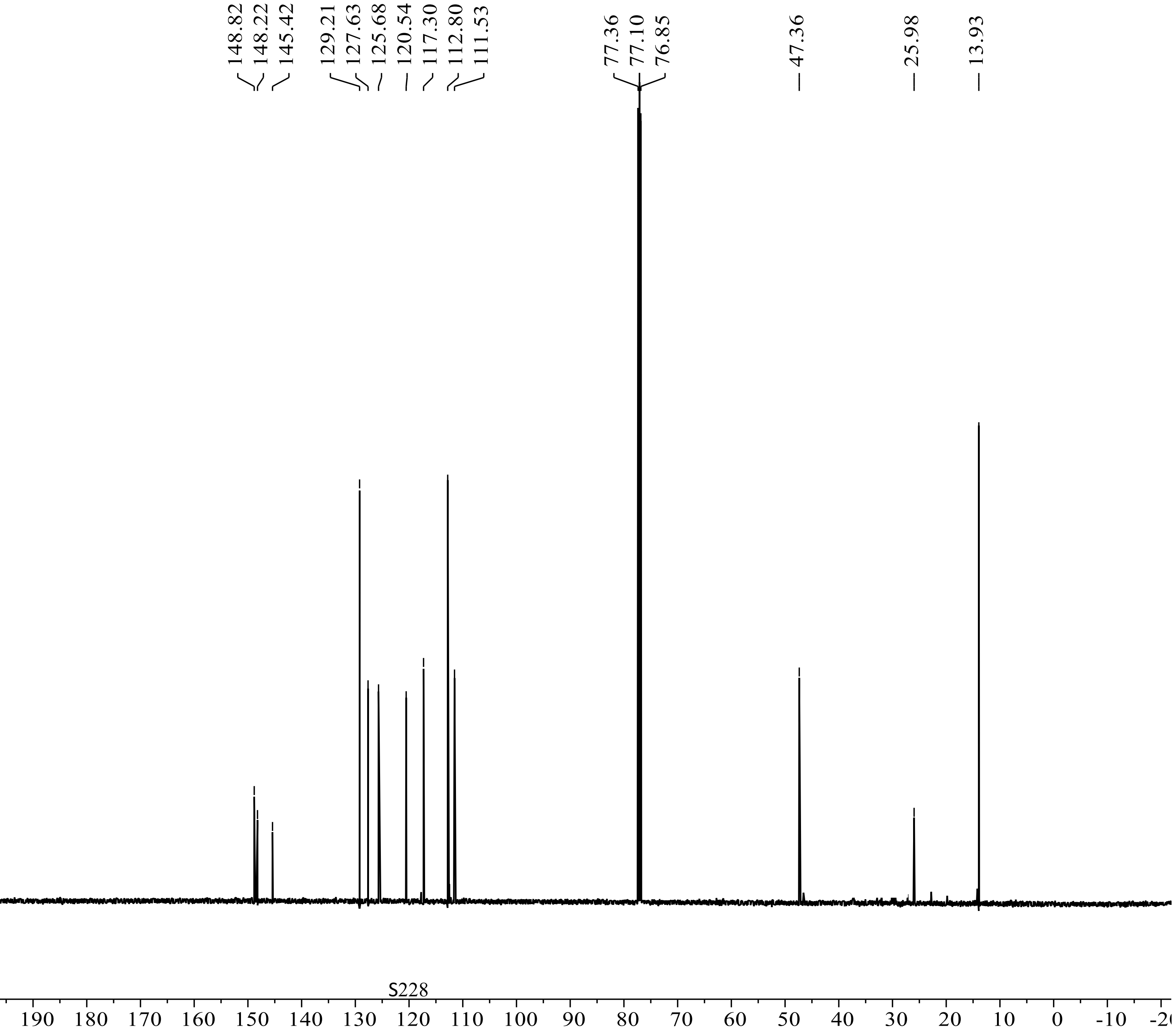

148.82
148.22
145.42
129.21
127.63
125.68
120.54
117.30
112.80
111.53
77.36
77.10
76.85
47.36
25.98
13.93
260 250 240 230 220 210 200 190 180 170 160 150 140 130 120 110 100 90 80 70 60 50 40 30 20 10 0 -10
f1 (ppm)

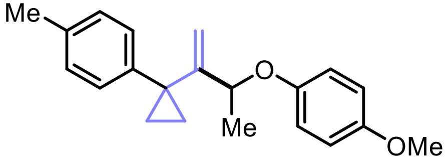
Me
O
Me
OMe

34

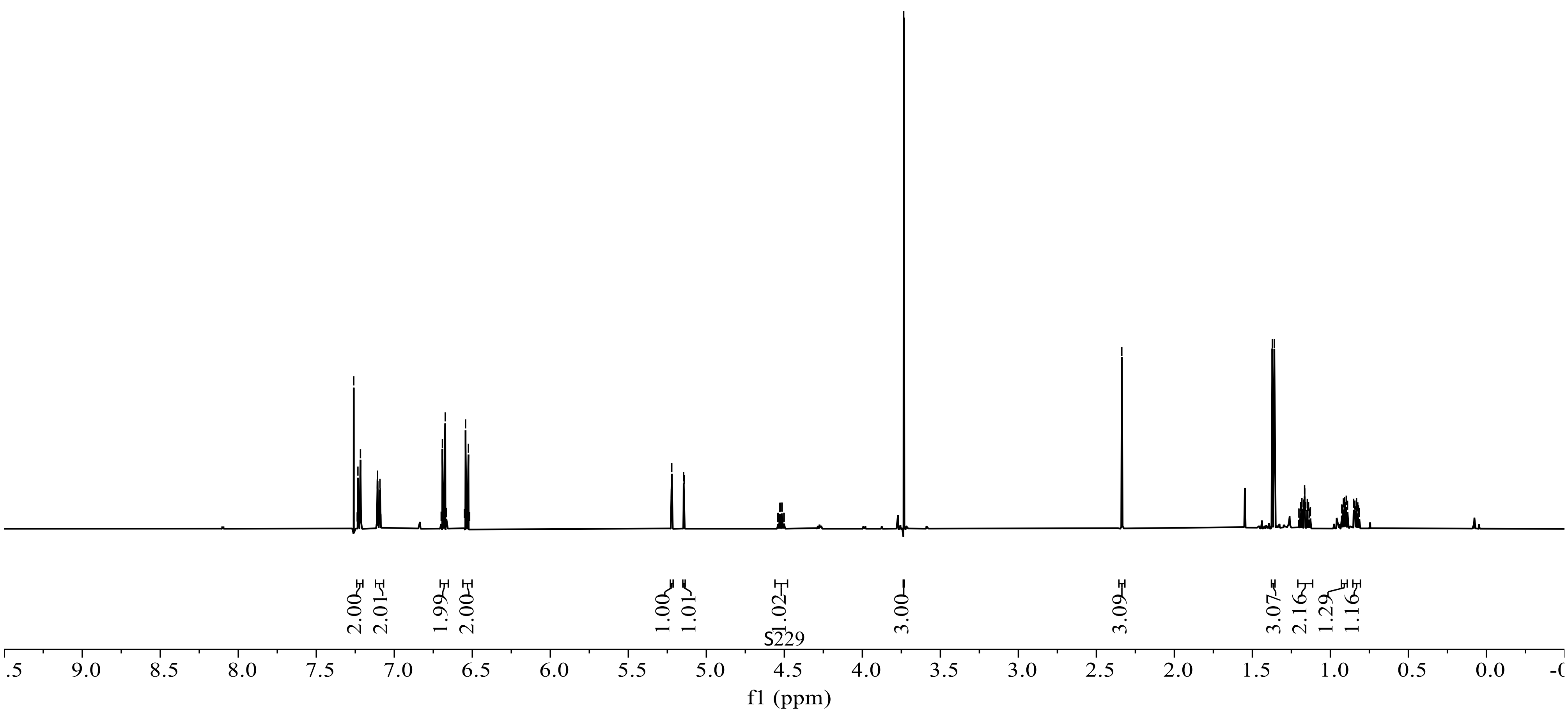
7.26
7.23
7.23
7.22
7.22
7.11
7.11
7.11
7.10
7.10
7.09
7.09
7.09
7.09
6.69
6.69
6.68
6.67
6.54
6.54
6.53
6.53
5.22
5.22
5.22
5.15
5.14
4.53
4.53
4.52
4.51
3.73
2.34
1.37
1.36
1.19
1.18
1.18
1.17
1.17
1.16
1.16
1.15
1.14
0.93
0.92
0.92
0.91
0.91
0.90
0.90
0.89
0.85
0.84
0.84
0.83
0.83
0.82
0.82
2.00
2.01
1.99
2.00
1.00
1.01
1.02
3.00
3.09
3.07
2.16
1.29
1.16
f1 (ppm)

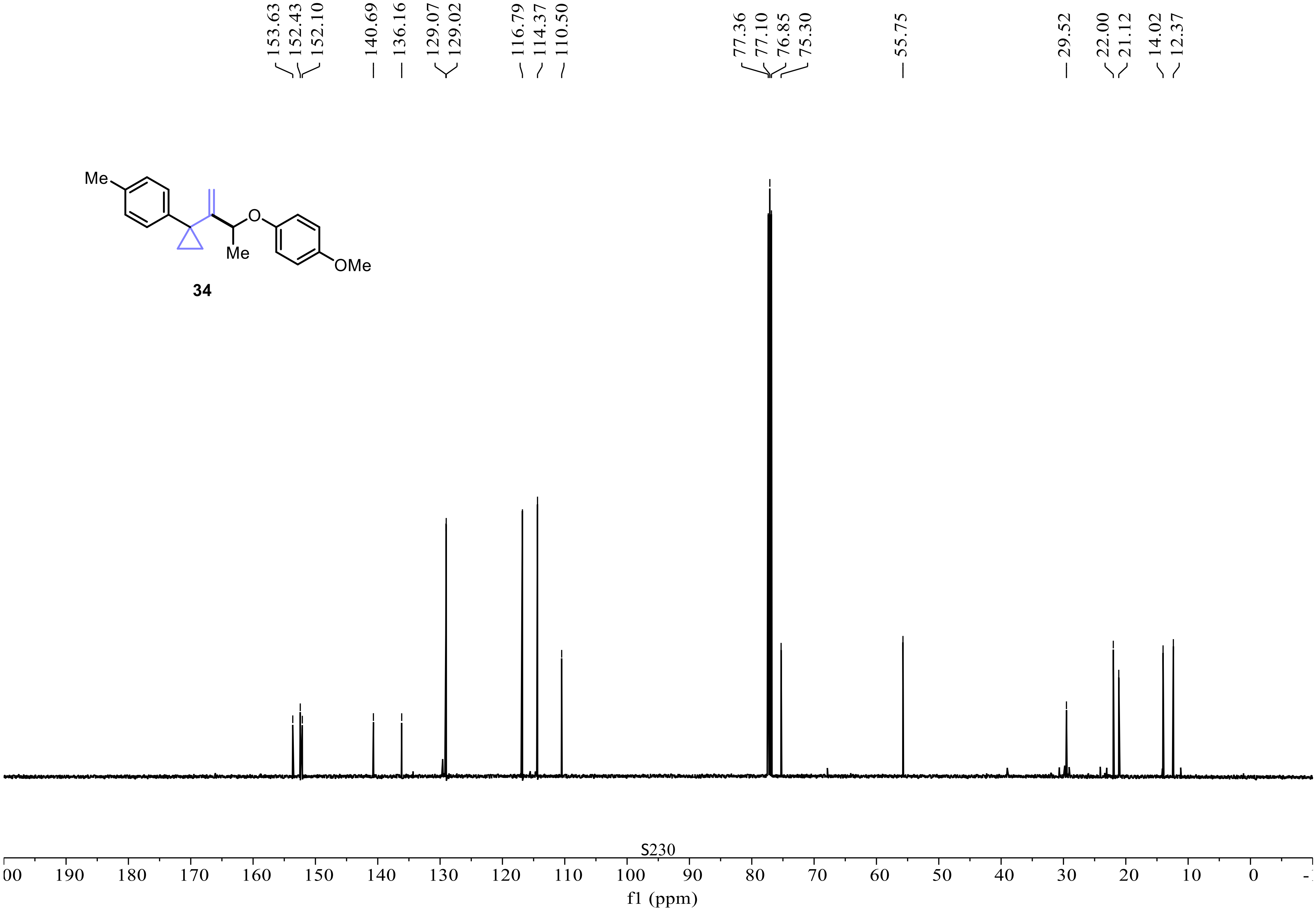

153.63
152.43
152.10
140.69
136.16
129.07
129.02
116.79
114.37
110.50
77.36
77.10
76.85
75.30
55.75
29.52
22.00
21.12
14.02
12.37
Me
O
Me
OMe
34
00 190 180 170 160 150 140 130 120 110 100 90 80 70 60 50 40 30 20 10 0
f1 (ppm)

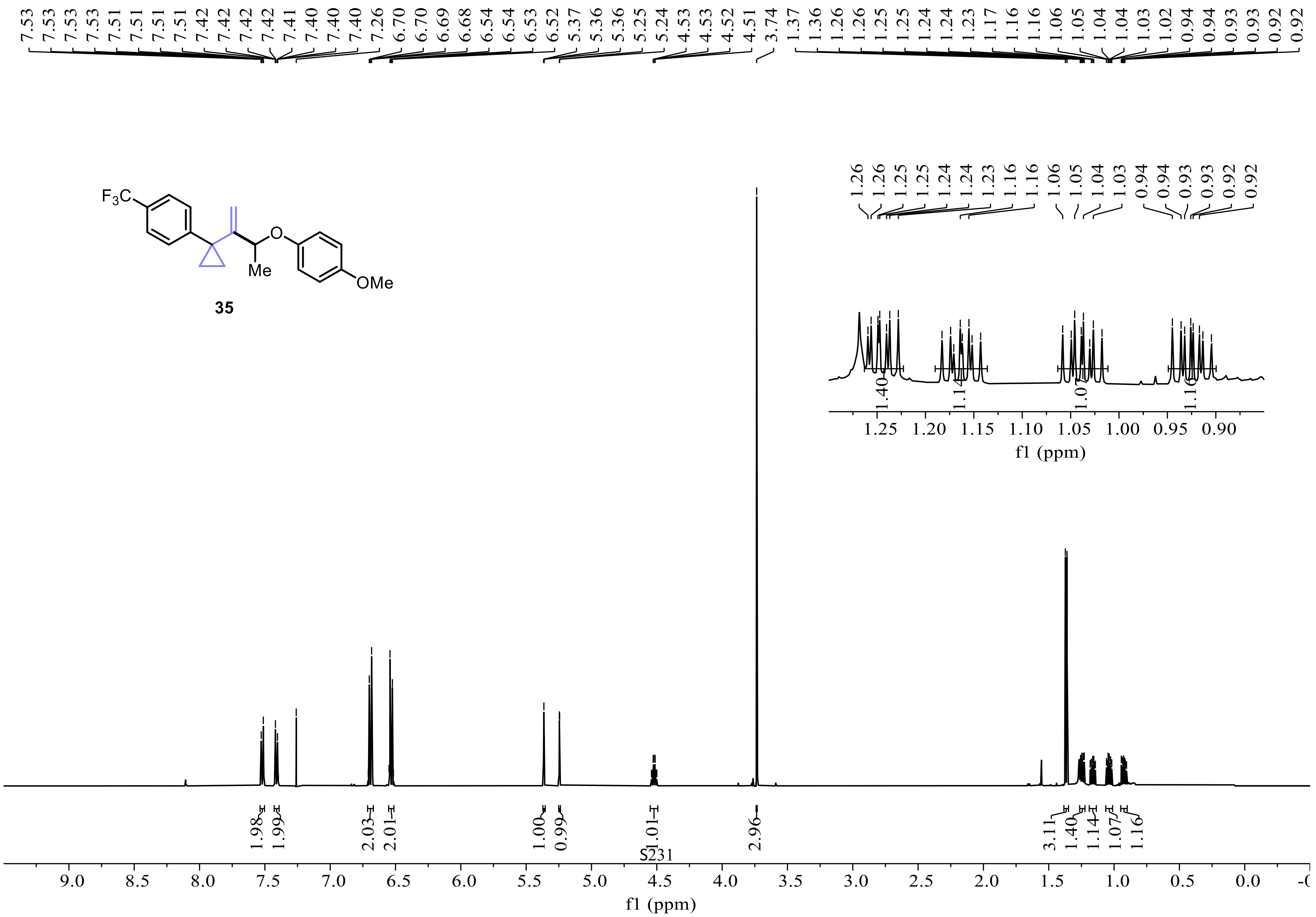

35

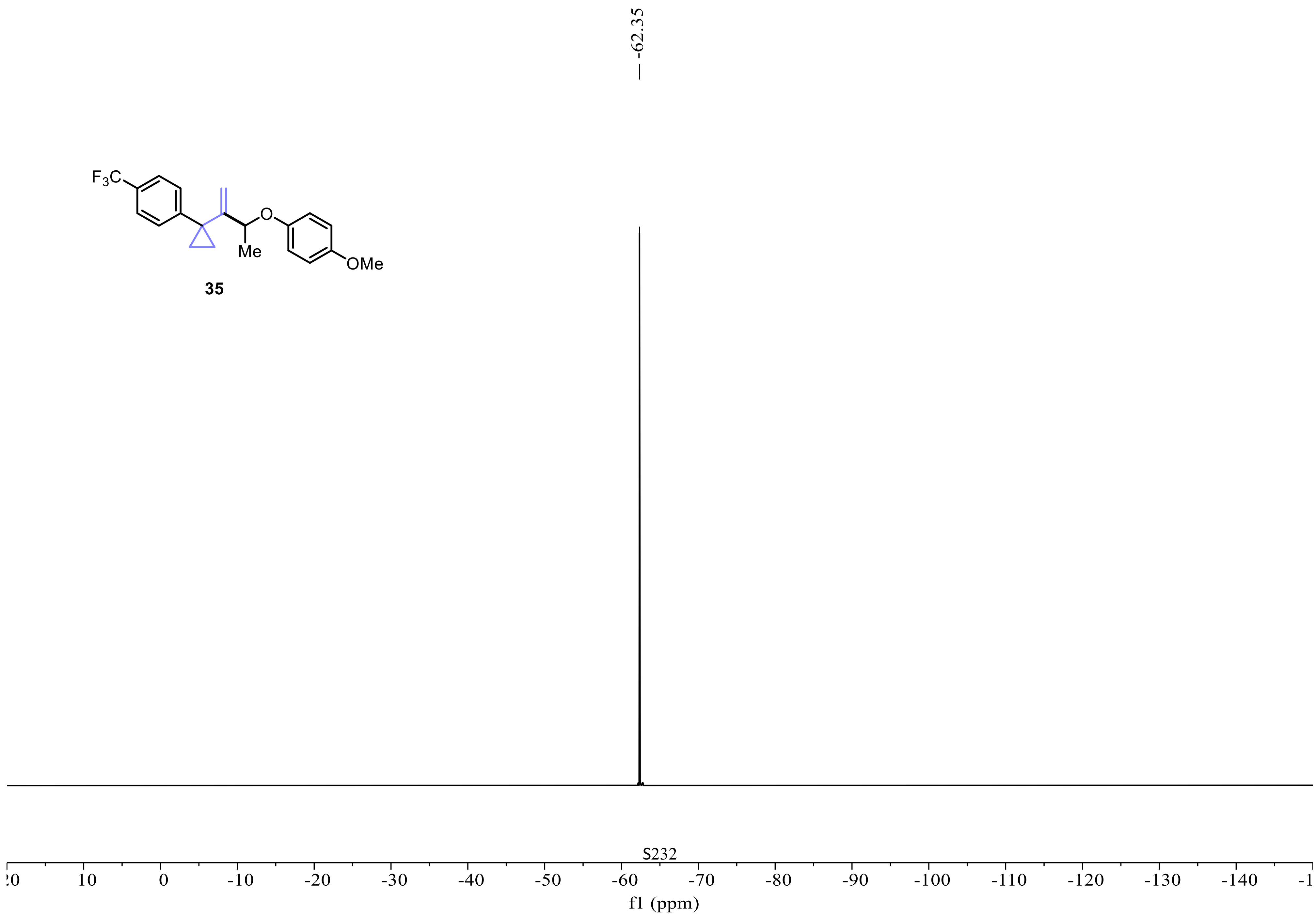
-62.35
F3C
O
Me
OMe
35
20
10
0
-10
-20
-30
-40
-50
-60
-70
-80
-90
-100
-110
-120
-130
-140
f1 (ppm)

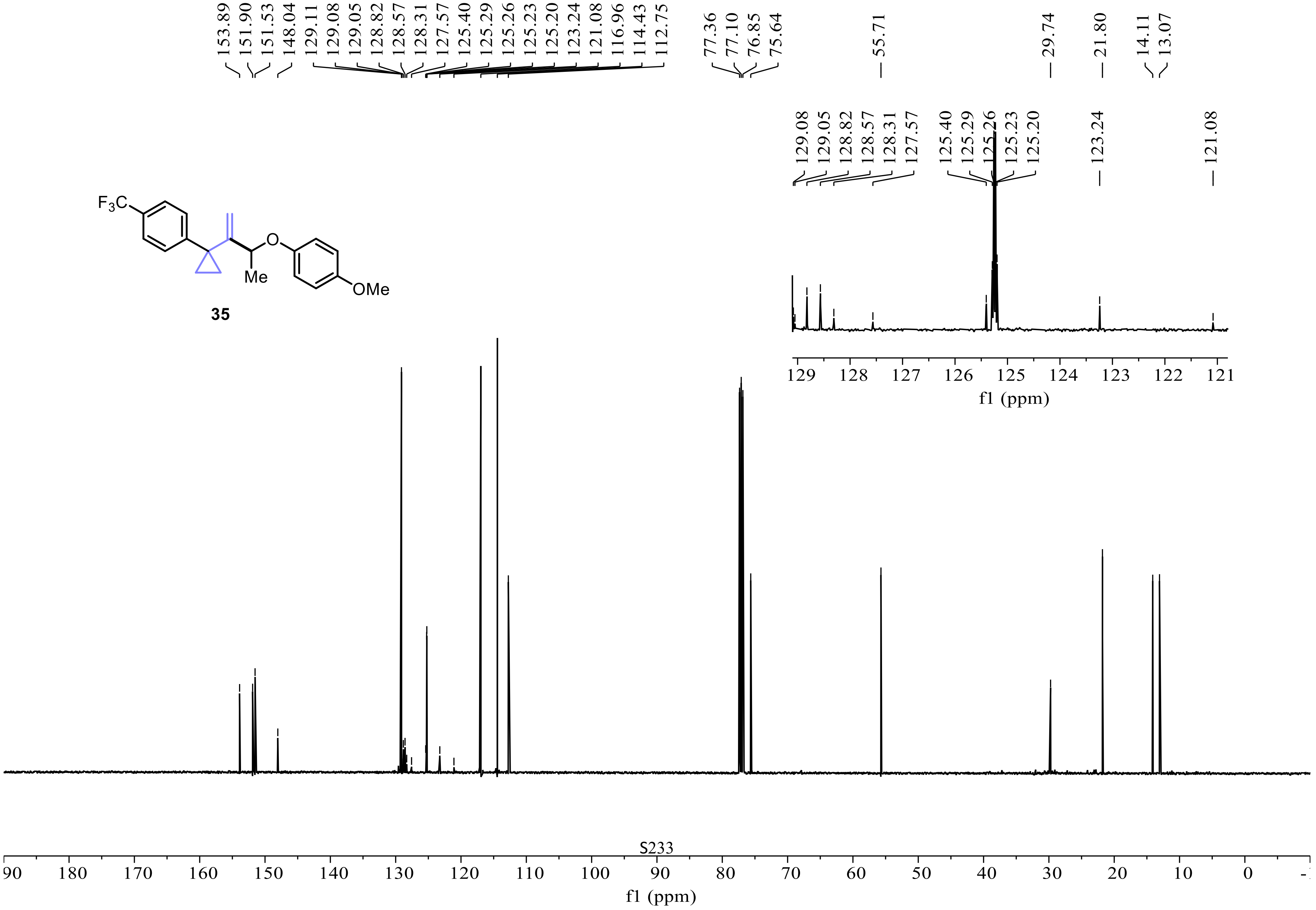
153.89
151.90
151.53
148.04
129.11
129.08
129.05
128.82
128.57
128.31
127.57
125.40
125.29
125.26
125.23
125.20
123.24
121.08
116.96
114.43
112.75
77.36
77.10
76.85
75.64
55.71
29.74
21.80
14.11
13.07
F3C
O
Me
OMe
35
f1 (ppm)

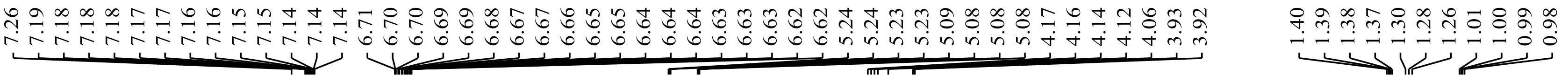
7.26
7.19
7.18
7.18
7.18
7.17
7.17
7.16
7.16
7.15
7.15
7.14
7.14
7.14
6.71
6.70
6.70
6.69
6.69
6.68
6.67
6.67
6.66
6.65
6.65
6.64
6.64
6.63
6.63
6.63
6.62
6.62
5.24
5.24
5.23
5.23
5.09
5.08
5.08
5.08
4.17
4.16
4.14
4.12
4.06
3.93
3.92
1.40
1.39
1.38
1.37
1.30
1.28
1.26
1.01
1.00
0.99
0.98

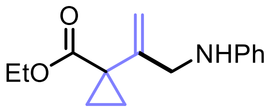
O
EtO
NHPh

38

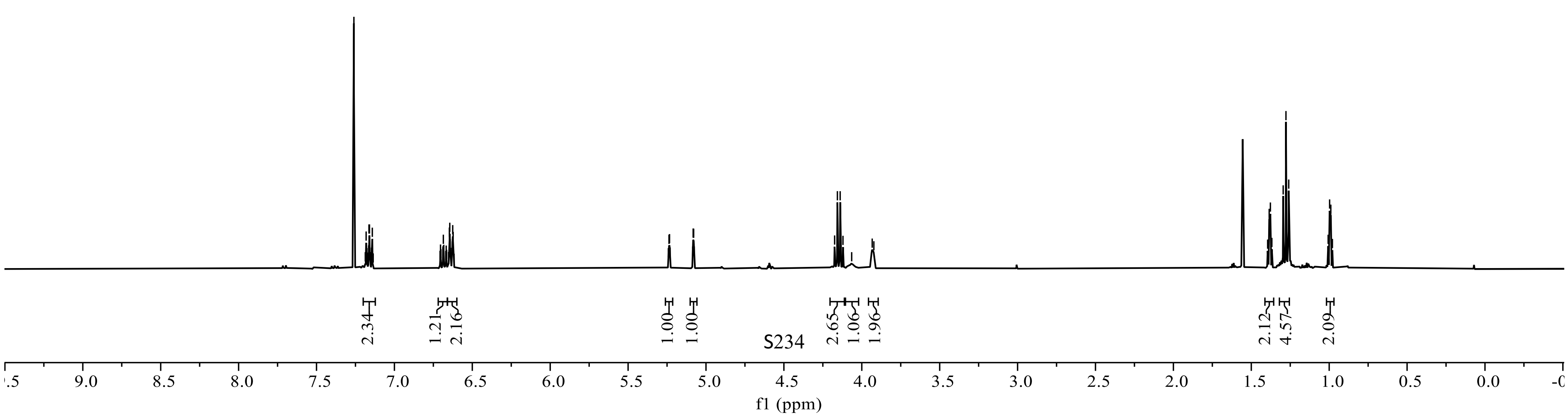
2.34
1.21
2.16
1.00
1.00
2.65
1.06
1.96
2.12
4.57
2.09
9.0
8.5
8.0
7.5
7.0
6.5
6.0
5.5
5.0
4.5
4.0
3.5
3.0
2.5
2.0
1.5
1.0
0.5
0.0
f1 (ppm)

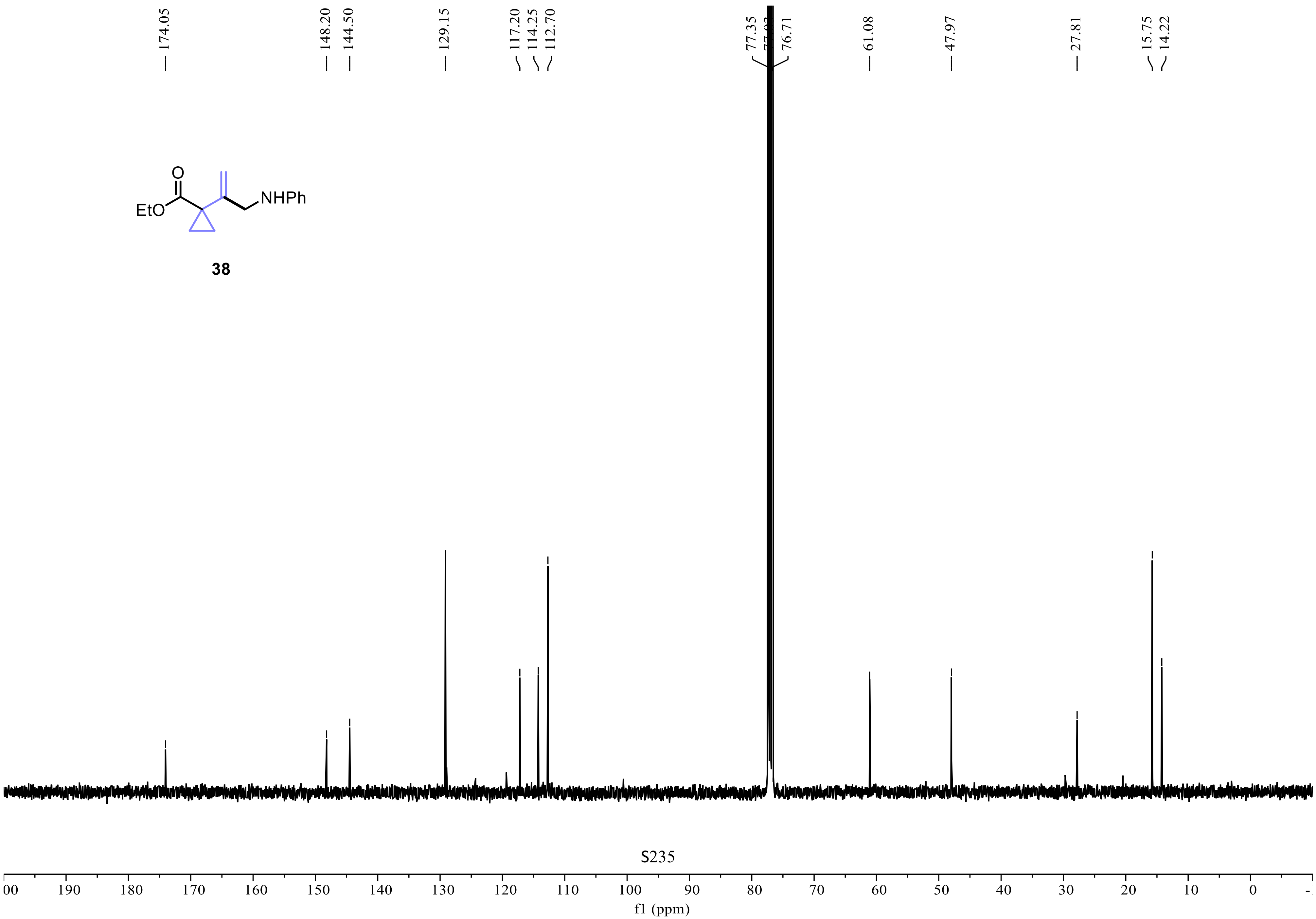
174.05
148.20
144.50
129.15
117.20
114.25
112.70
77.35
77.03
76.71
61.08
47.97
27.81
15.75
14.22
O
EtO
NHPh
38
f1 (ppm)

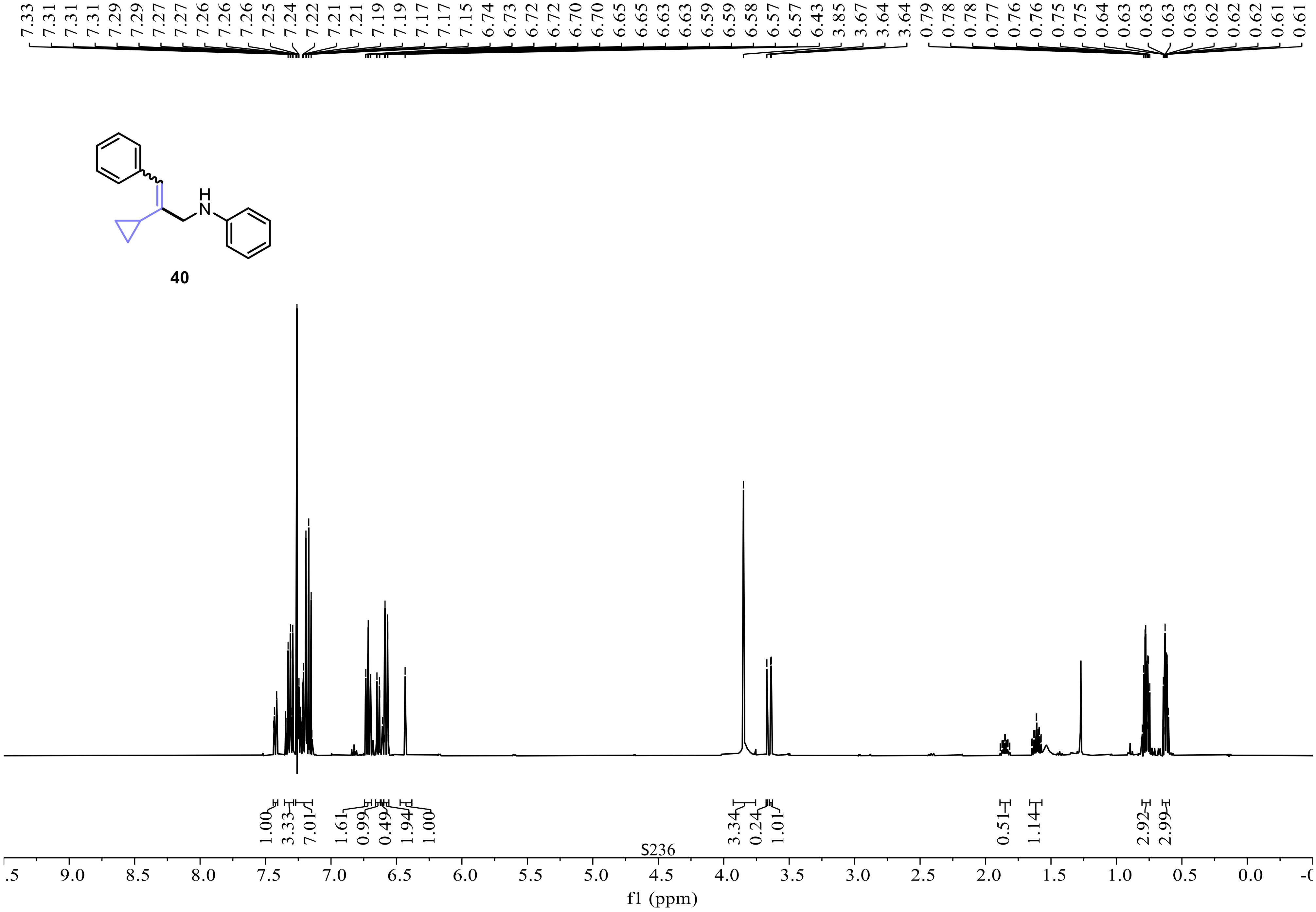
7.33 7.31 7.31 7.31 7.29 7.29 7.27 7.27 7.26 7.26 7.26 7.25 7.24 7.22 7.21 7.21 7.19 7.19 7.17 7.17 7.15 6.74 6.73 6.72 6.72 6.70 6.70 6.65 6.65 6.63 6.63 6.59 6.59 6.58 6.57 6.57 6.43 3.85 3.67 3.64 3.64 0.79 0.78 0.78 0.77 0.76 0.76 0.75 0.75 0.64 0.63 0.63 0.63 0.63 0.62 0.62 0.62 0.61 0.61
H
N
40
1.00 3.33 7.01 1.61 0.99 0.49 1.94 1.00 3.34 0.24 1.01 0.51 1.14 2.92 2.99
.5 9.0 8.5 8.0 7.5 7.0 6.5 6.0 5.5 5.0 4.5 4.0 3.5 3.0 2.5 2.0 1.5 1.0 0.5 0.0
f1 (ppm)

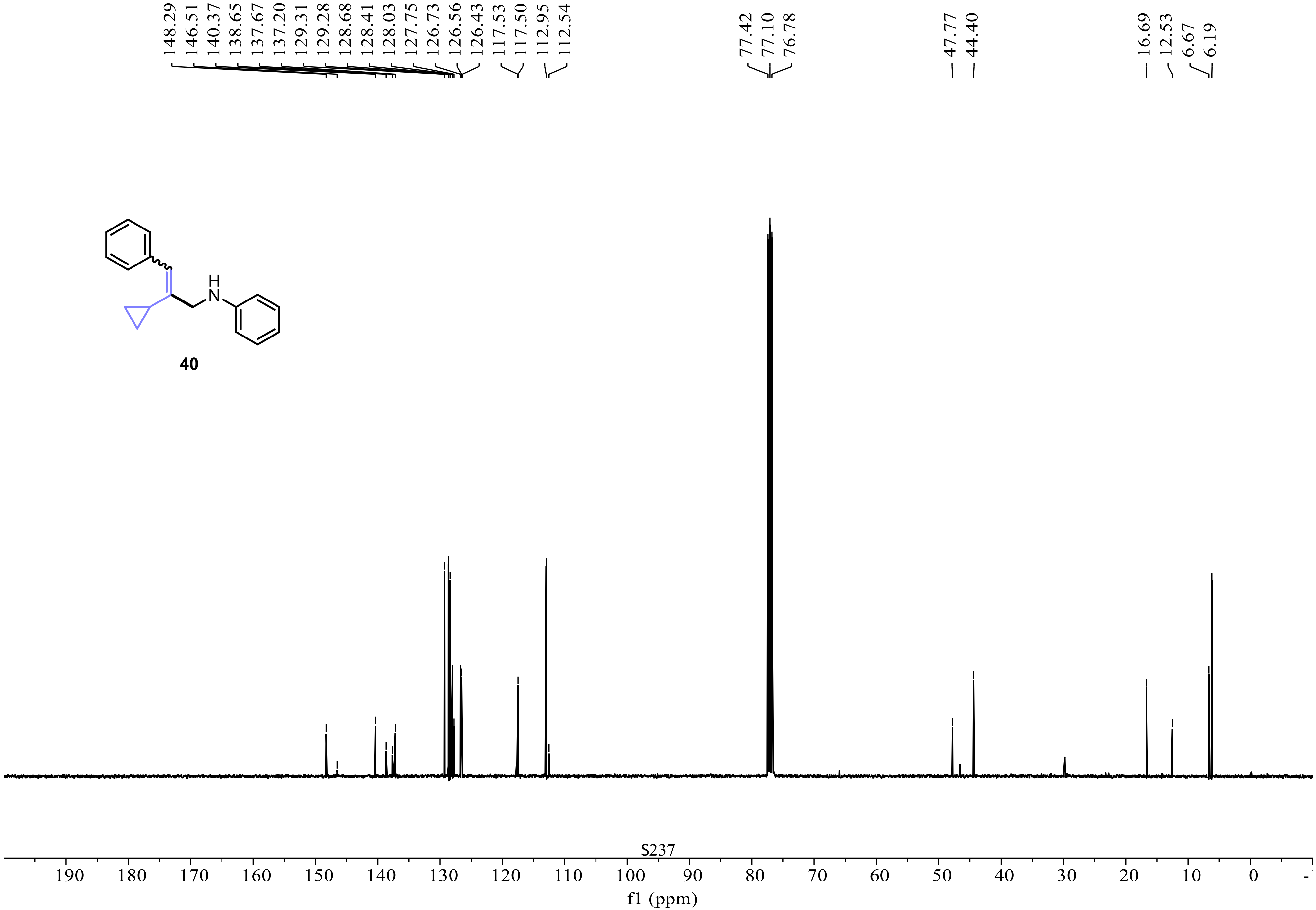
40
148.29
146.51
140.37
138.65
137.67
137.20
129.31
129.28
128.68
128.41
128.03
127.75
126.73
126.56
126.43
117.53
117.50
112.95
112.54
77.42
77.10
76.78
47.77
44.40
16.69
12.53
6.67
6.19
190 180 170 160 150 140 130 120 110 100 90 80 70 60 50 40 30 20 10 0
f1 (ppm)

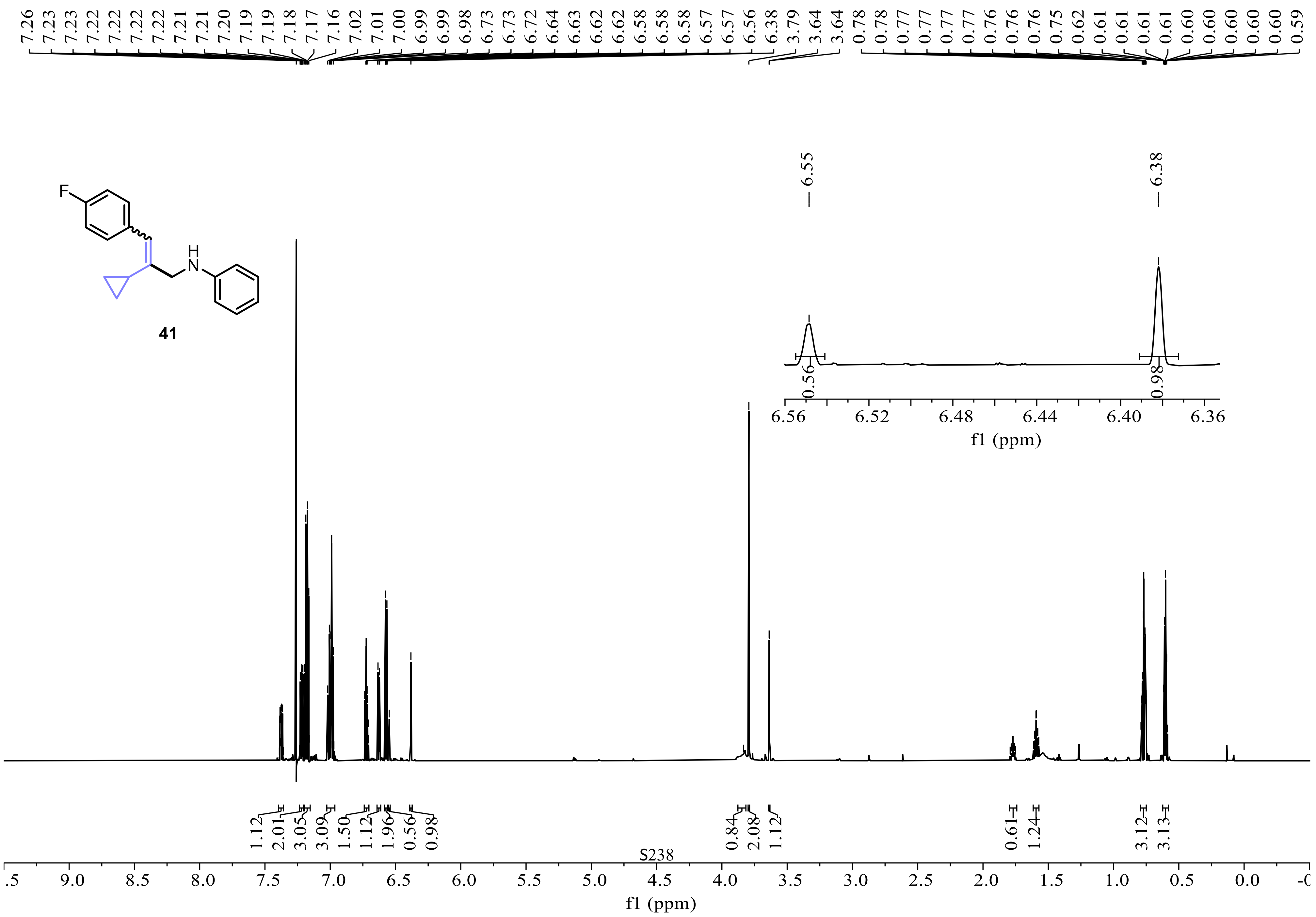
7.26 7.23 7.23 7.22 7.22 7.22 7.21 7.21 7.20 7.19 7.19 7.18 7.17 7.16 7.02 7.01 7.00 6.99 6.99 6.98 6.73 6.73 6.72 6.64 6.63 6.62 6.62 6.58 6.58 6.58 6.57 6.57 6.56 6.38 3.79 3.64 3.64 0.78 0.78 0.77 0.77 0.77 0.77 0.76 0.76 0.76 0.75 0.62 0.61 0.61 0.61 0.61 0.60 0.60 0.60 0.60 0.59
F
H
N
41
6.55
6.38
0.56
0.98
6.56 6.52 6.48 6.44 6.40 6.36
f1 (ppm)
1.12 2.01 3.05 3.09 1.50 1.12 1.96 0.56 0.98 0.84 2.08 1.12 0.61 1.24 3.12 3.13
.5 9.0 8.5 8.0 7.5 7.0 6.5 6.0 5.5 5.0 4.5 4.0 3.5 3.0 2.5 2.0 1.5 1.0 0.5 0.0
f1 (ppm)

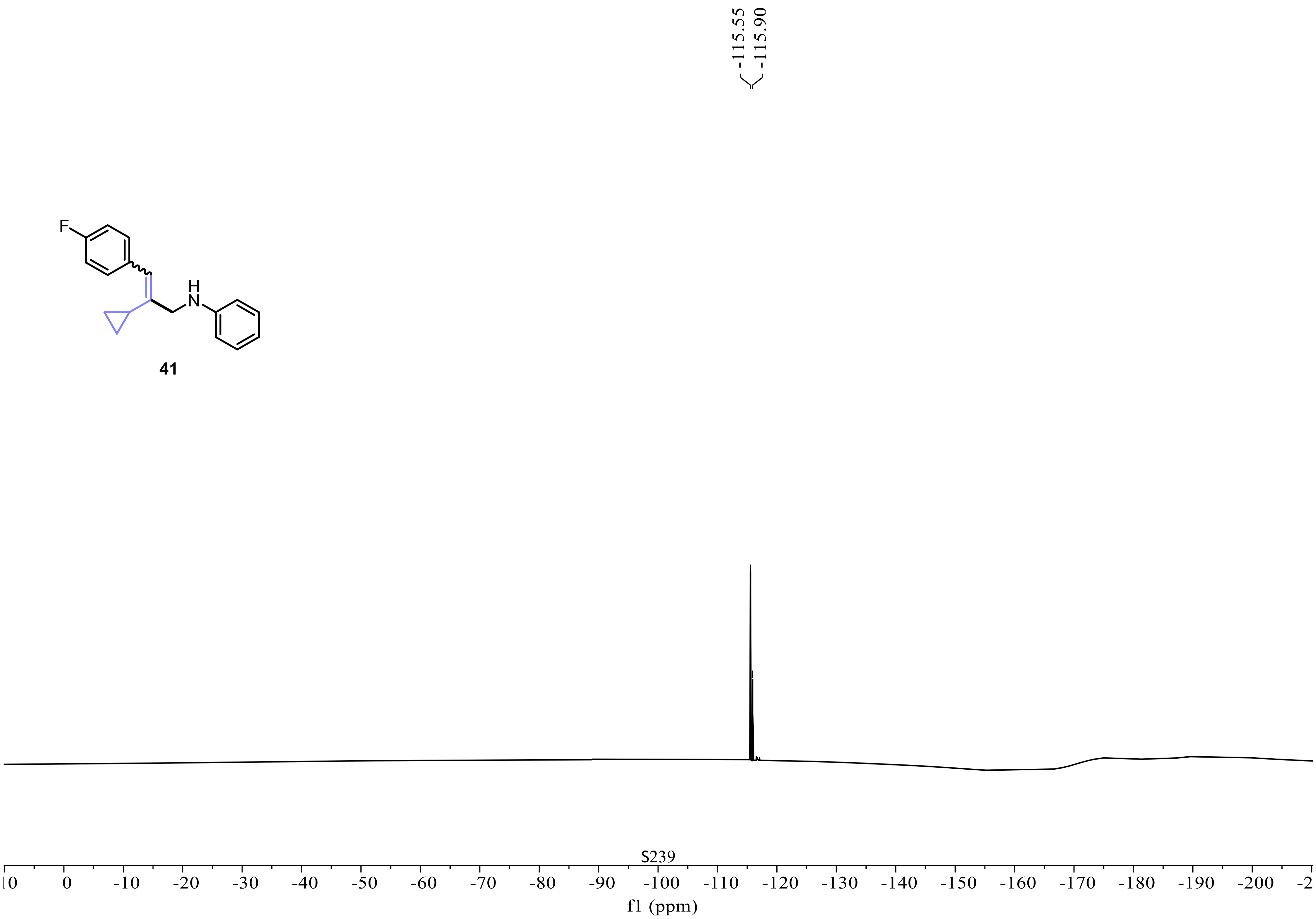
-115.55
-115.90
F
H
N
41
10
0
-10
-20
-30
-40
-50
-60
-70
-80
-90
-100
-110
-120
-130
-140
-150
-160
-170
-180
-190
-200
-2
f1 (ppm)

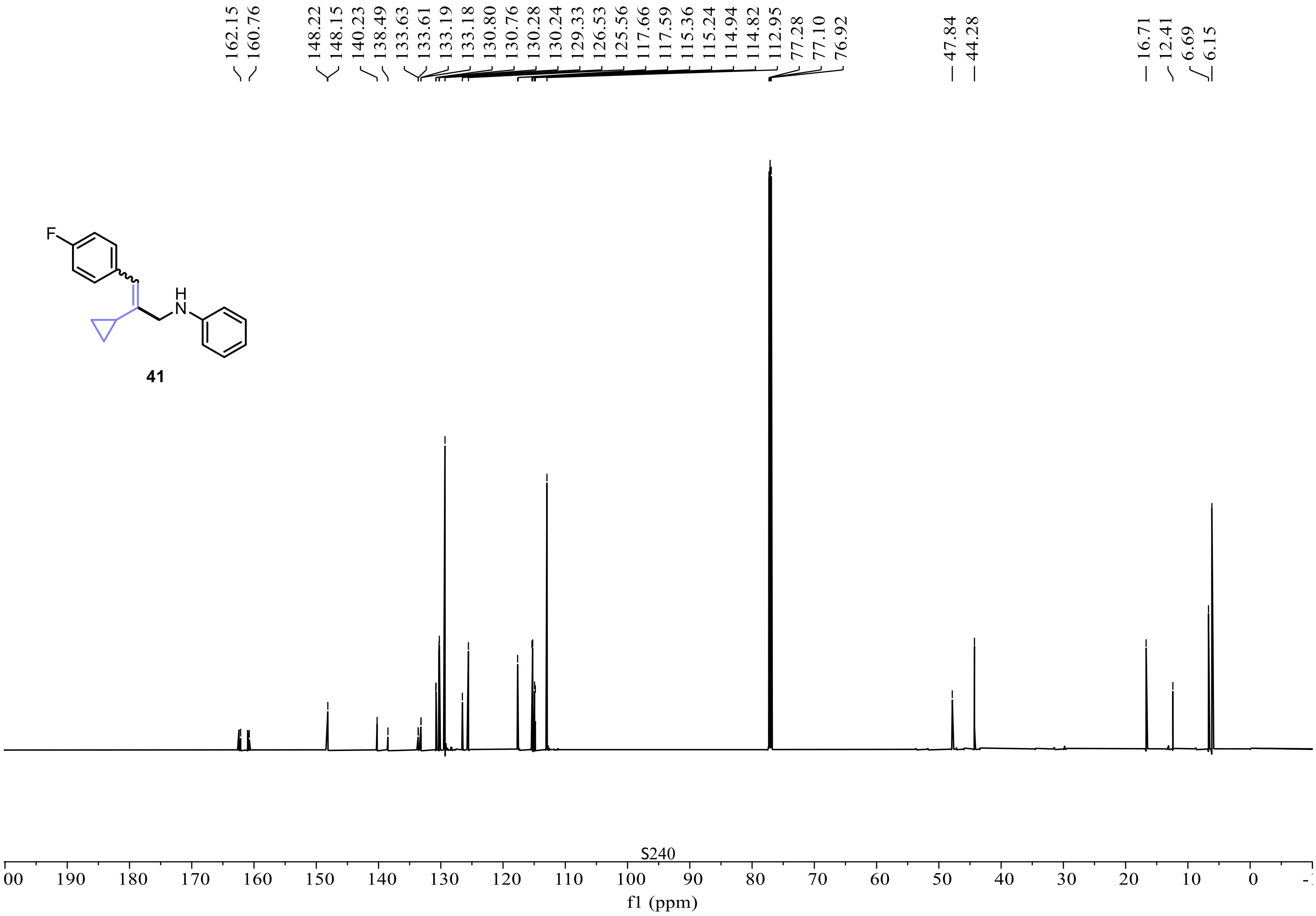
162.15
160.76
148.22
148.15
140.23
138.49
133.63
133.61
133.19
133.18
130.80
130.76
130.28
130.24
129.33
126.53
125.56
117.66
117.59
115.36
115.24
114.94
114.82
112.95
77.28
77.10
76.92
47.84
44.28
16.71
12.41
6.69
6.15
F
H
N
41
f1 (ppm)

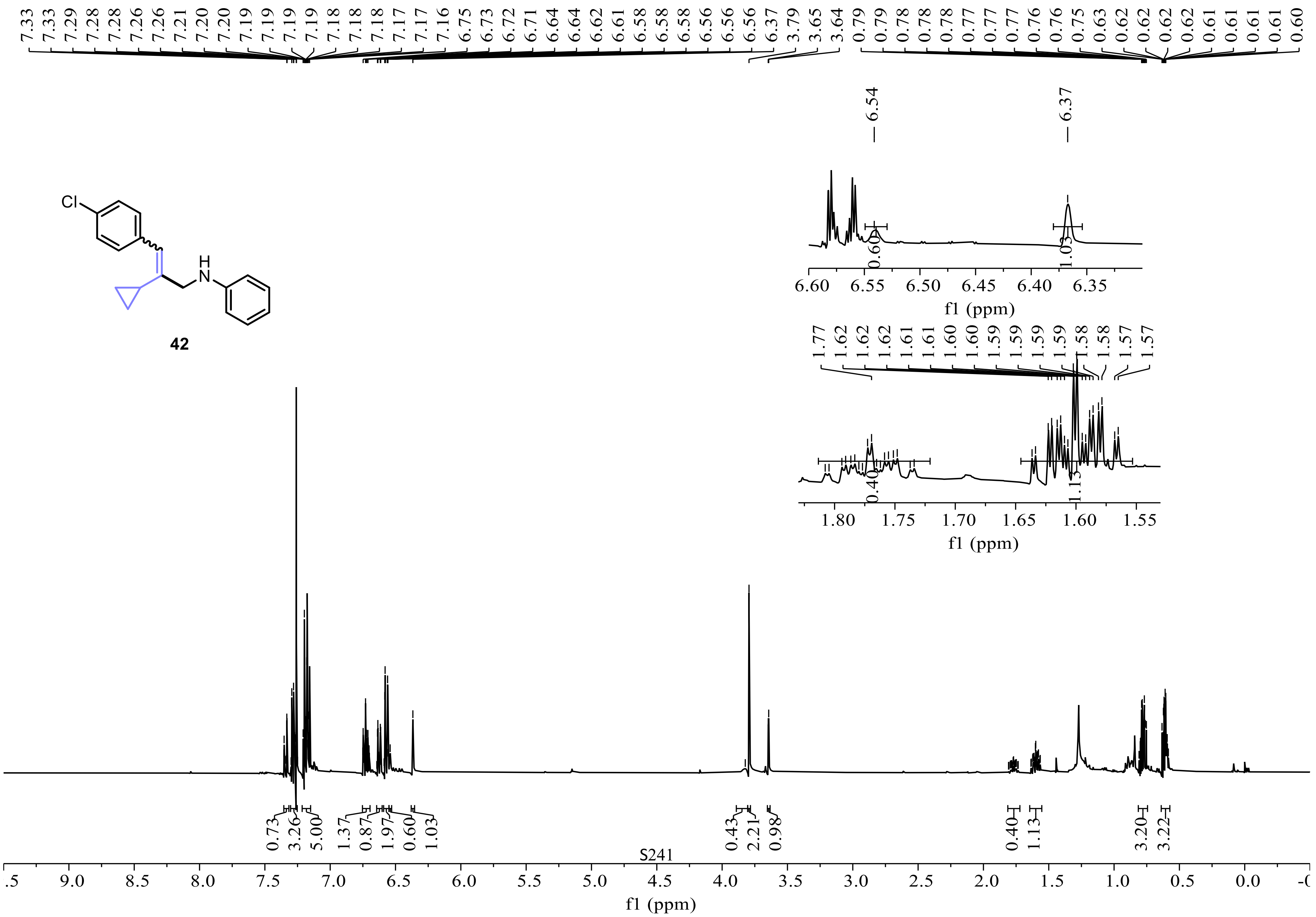
Cl
H
N
42
7.33 7.33 7.29 7.28 7.28 7.26 7.26 7.21 7.20 7.20 7.19 7.19 7.19 7.19 7.18 7.18 7.18 7.17 7.17 7.16 6.75 6.73 6.72 6.71 6.64 6.64 6.62 6.61 6.58 6.58 6.58 6.56 6.56 6.56 6.37 3.79 3.65 3.64 0.79 0.79 0.78 0.78 0.78 0.77 0.77 0.77 0.76 0.76 0.75 0.63 0.62 0.62 0.62 0.62 0.61 0.61 0.61 0.61 0.60
6.54
6.37
0.60
1.03
6.60 6.55 6.50 6.45 6.40 6.35
f1 (ppm)
1.77 1.62 1.62 1.62 1.61 1.61 1.60 1.60 1.59 1.59 1.59 1.59 1.58 1.58 1.57 1.57
0.40
1.13
1.80 1.75 1.70 1.65 1.60 1.55
f1 (ppm)
0.73 3.26 5.00 1.37 0.87 1.97 0.60 1.03 0.43 2.21 0.98 0.40 1.13 3.20 3.22
.5 9.0 8.5 8.0 7.5 7.0 6.5 6.0 5.5 5.0 4.5 4.0 3.5 3.0 2.5 2.0 1.5 1.0 0.5 0.0
f1 (ppm)

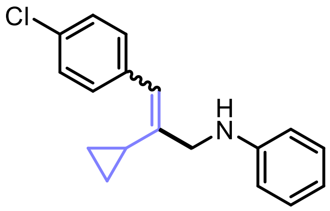
Cl
H
N

42

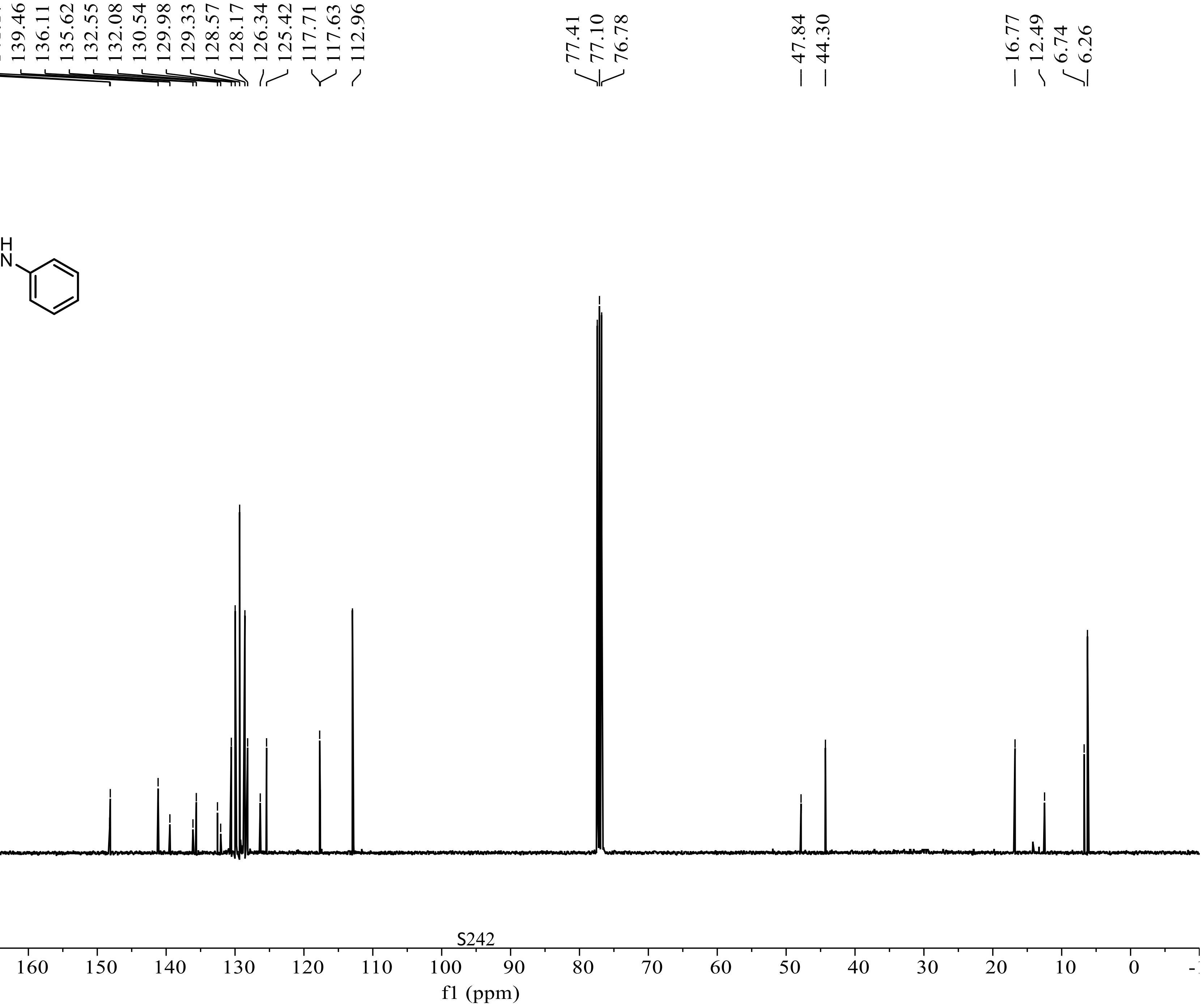
148.20
148.11
141.17
139.46
136.11
135.62
132.55
132.08
130.54
129.98
129.33
128.57
128.17
126.34
125.42
117.71
117.63
112.96
77.41
77.10
76.78
47.84
44.30
16.77
12.49
6.74
6.26
00
190
180
170
160
150
140
130
120
110
100
90
80
70
60
50
40
30
20
10
0
f1 (ppm)

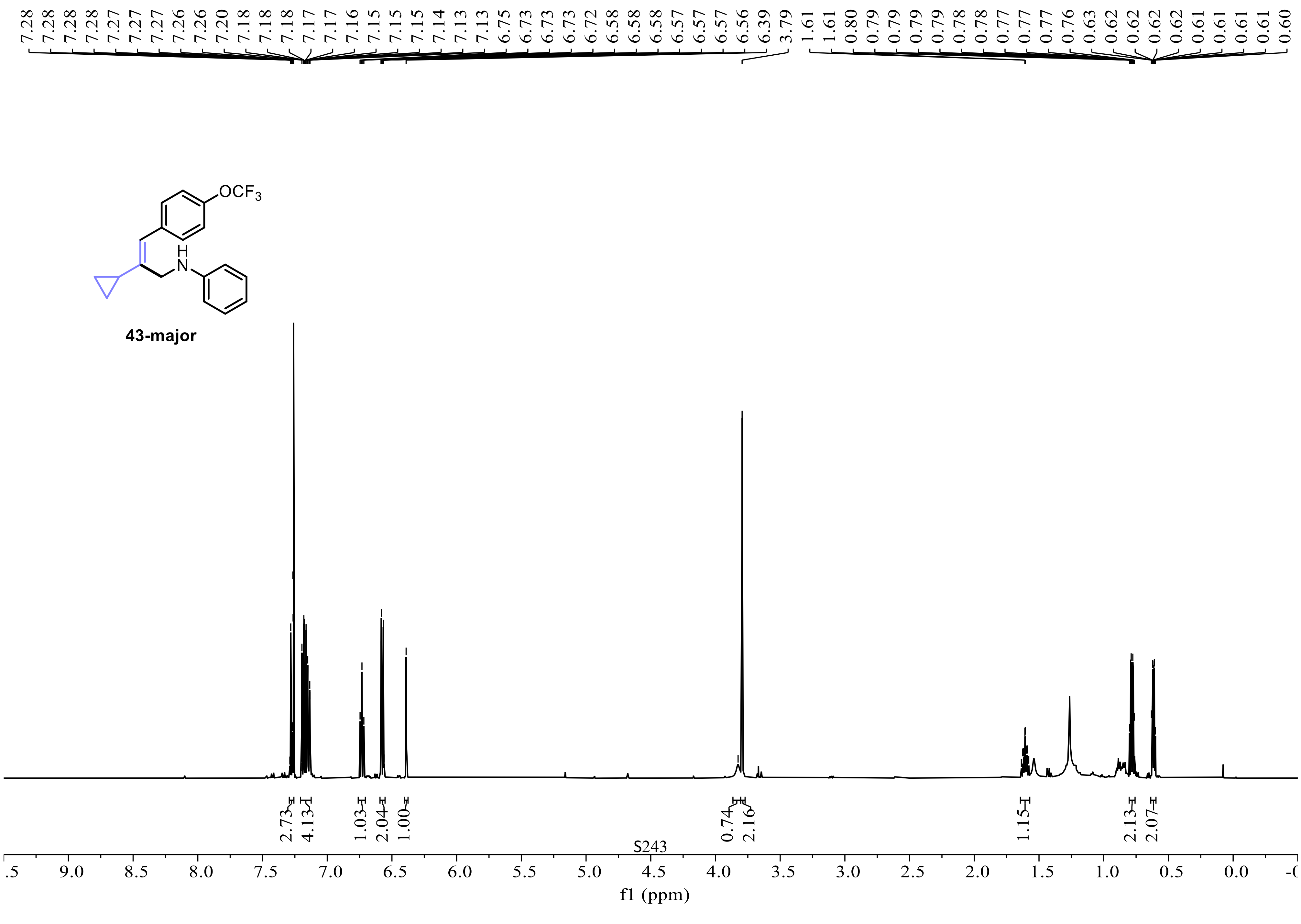

7.28
7.28
7.28
7.28
7.27
7.27
7.27
7.26
7.26
7.20
7.18
7.18
7.18
7.17
7.17
7.16
7.15
7.15
7.15
7.14
7.13
7.13
6.75
6.73
6.73
6.73
6.72
6.58
6.58
6.58
6.57
6.57
6.57
6.56
6.39
3.79
1.61
1.61
0.80
0.79
0.79
0.79
0.79
0.78
0.78
0.77
0.77
0.77
0.76
0.63
0.62
0.62
0.62
0.62
0.61
0.61
0.61
0.61
0.60
OCF3
H
N
43-major
2.73
4.13
1.03
2.04
1.00
0.74
2.16
1.15
2.13
2.07
9.0
8.5
8.0
7.5
7.0
6.5
6.0
5.5
5.0
4.5
4.0
3.5
3.0
2.5
2.0
1.5
1.0
0.5
0.0
f1 (ppm)

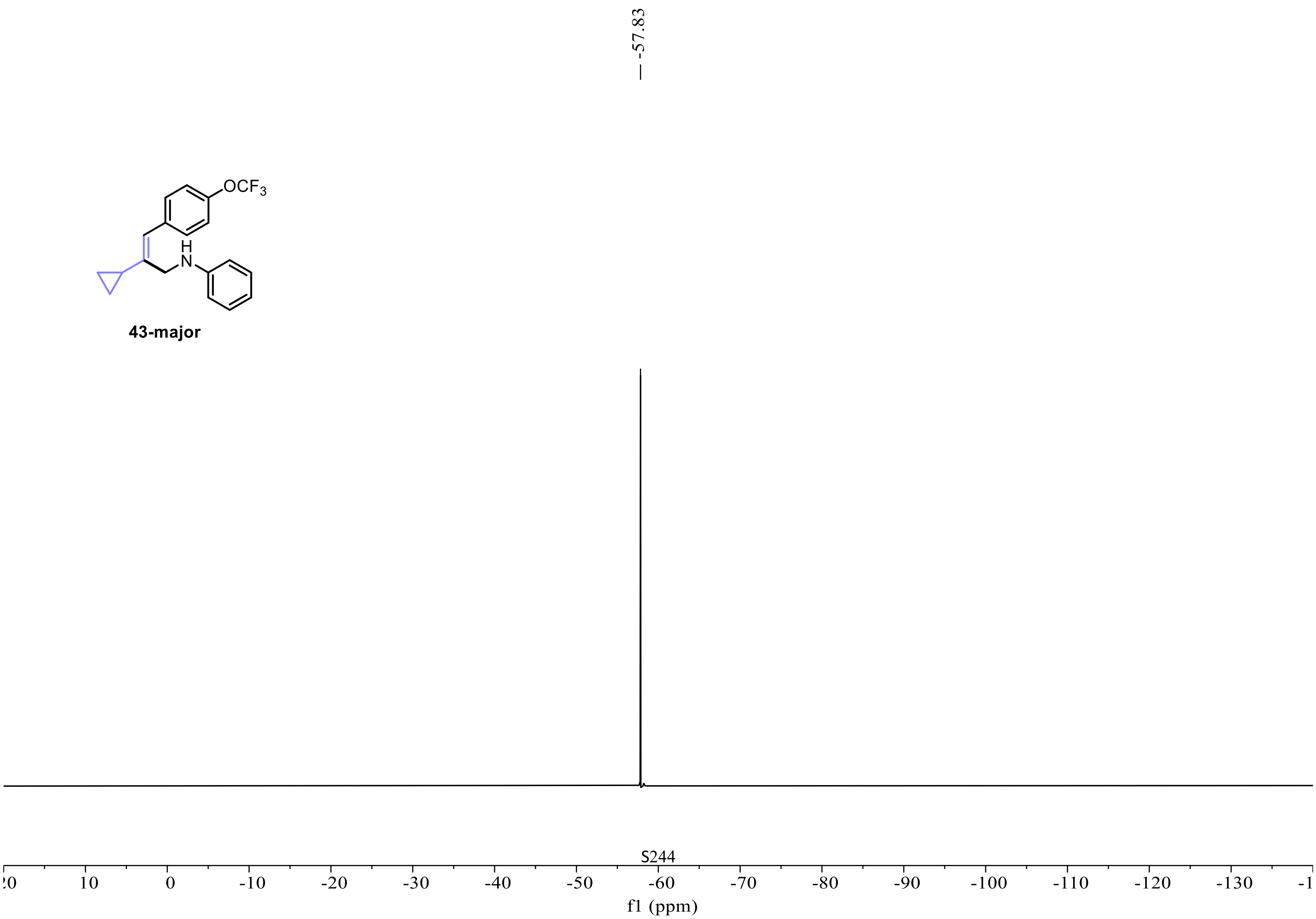

-57.83
OCF3
H
N
43-major
20
10
0
-10
-20
-30
-40
-50
-60
-70
-80
-90
-100
-110
-120
-130
f1 (ppm)

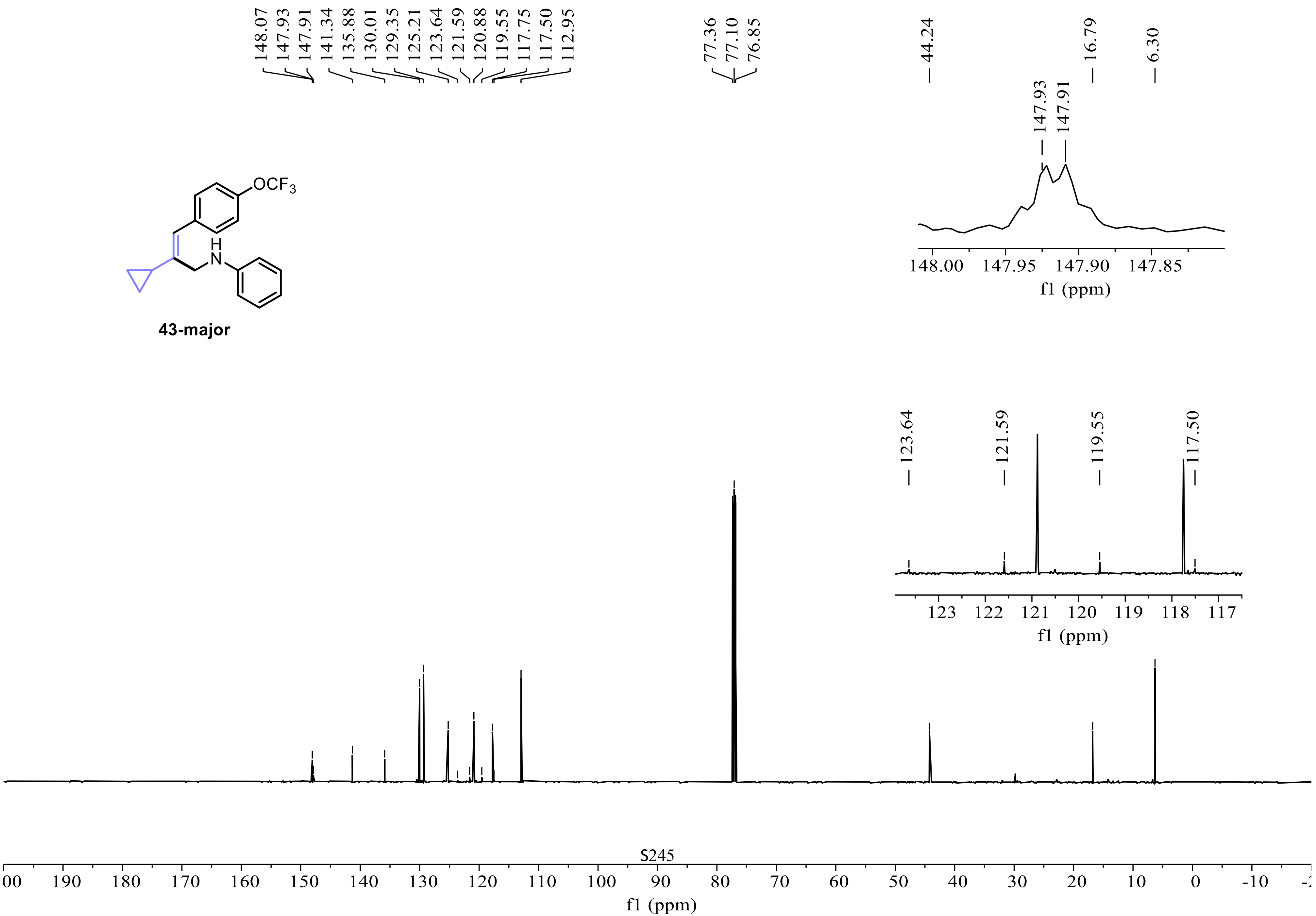
148.07
147.93
147.91
141.34
135.88
130.01
129.35
125.21
123.64
121.59
120.88
119.55
117.75
117.50
112.95
77.36
77.10
76.85
44.24
16.79
6.30
OCF3
H
N
43-major
147.93
147.91
148.00 147.95 147.90 147.85
f1 (ppm)
123.64
121.59
119.55
117.50
123 122 121 120 119 118 117
f1 (ppm)
00 190 180 170 160 150 140 130 120 110 100 90 80 70 60 50 40 30 20 10 0 -10
f1 (ppm)

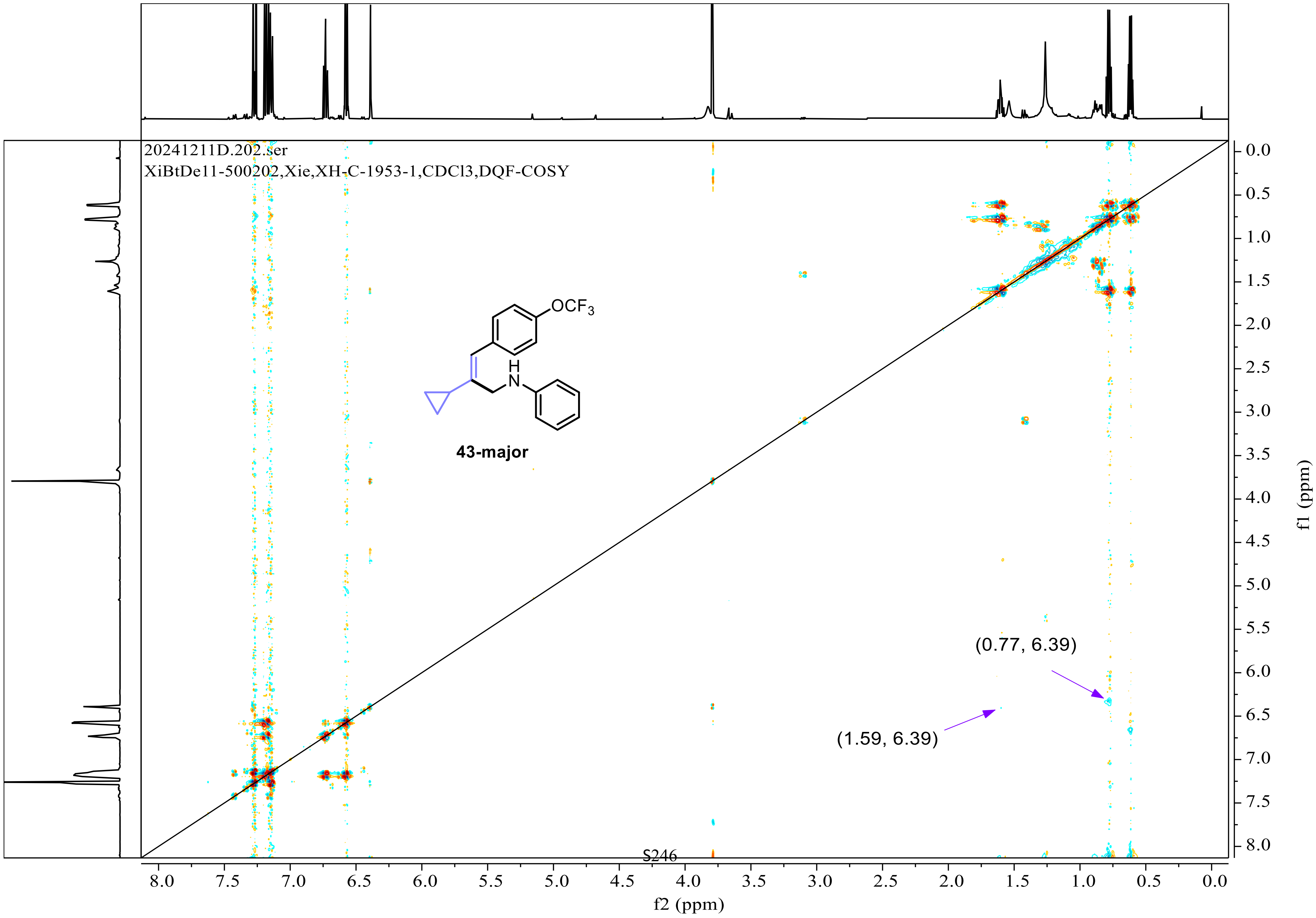
20241211D.202.ser
XiBtDe11-500202,Xie,XH-C-1953-1,CDCl3,DQF-COSY
OCF3
H
N
43-major
(0.77, 6.39)
(1.59, 6.39)
f1 (ppm)
f2 (ppm)

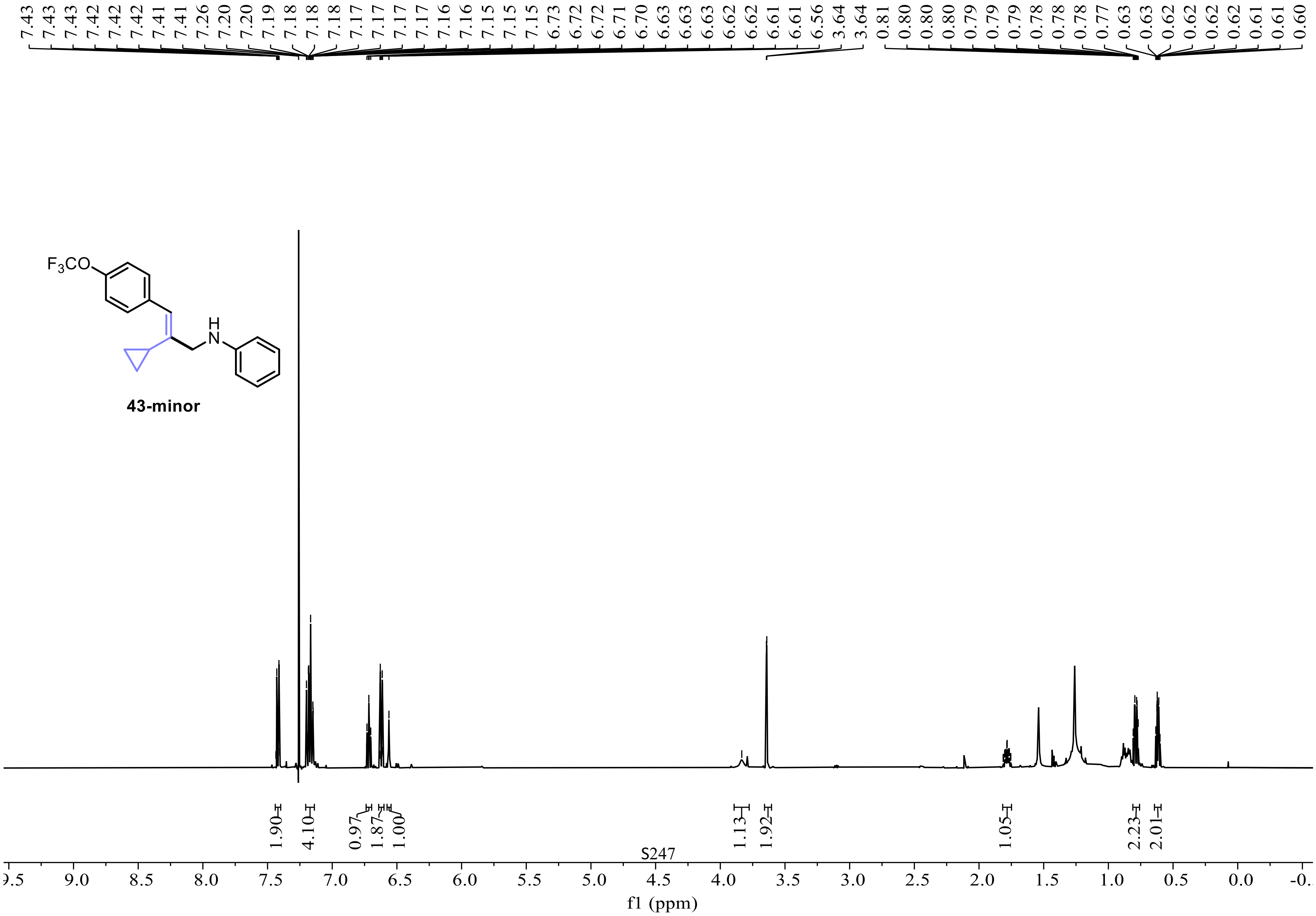
7.43 7.43 7.43 7.42 7.42 7.42 7.41 7.41 7.26 7.20 7.20 7.19 7.18 7.18 7.18 7.17 7.17 7.17 7.17 7.16 7.16 7.15 7.15 7.15 6.73 6.72 6.72 6.71 6.70 6.63 6.63 6.63 6.62 6.62 6.61 6.61 6.56 3.64 3.64 0.81 0.80 0.80 0.80 0.79 0.79 0.79 0.78 0.78 0.78 0.77 0.63 0.63 0.62 0.62 0.62 0.62 0.61 0.61 0.60
F3CO
H
N
43-minor
1.90
4.10
0.97
1.87
1.00
1.13
1.92
1.05
2.23
2.01
9.5 9.0 8.5 8.0 7.5 7.0 6.5 6.0 5.5 5.0 4.5 4.0 3.5 3.0 2.5 2.0 1.5 1.0 0.5 0.0 -0.
f1 (ppm)

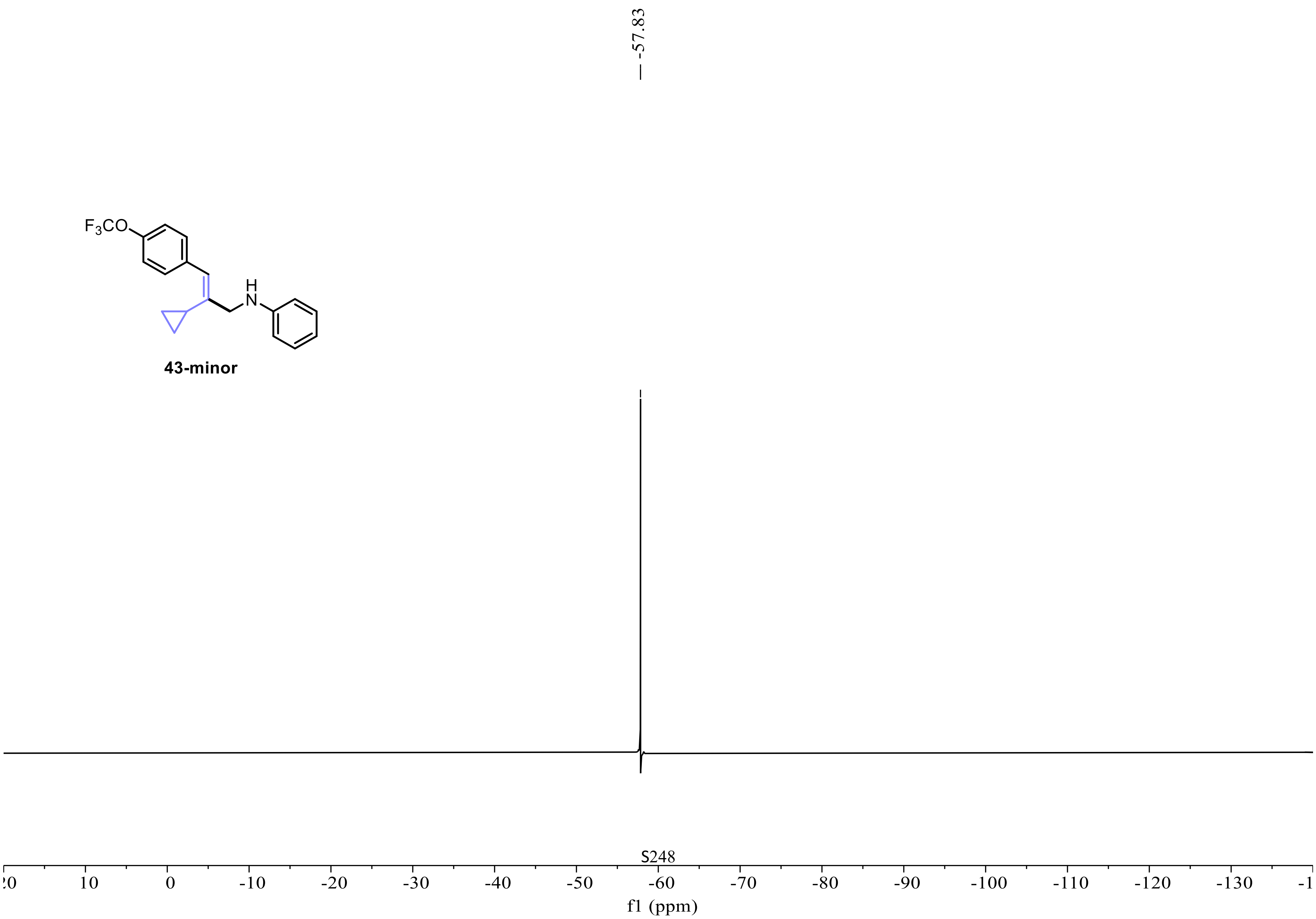
-57.83
F3CO
H
N
43-minor
10
0
-10
-20
-30
-40
-50
-60
-70
-80
-90
-100
-110
-120
-130
f1 (ppm)

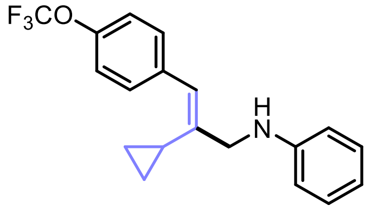
F3CO
H
N

43-minor

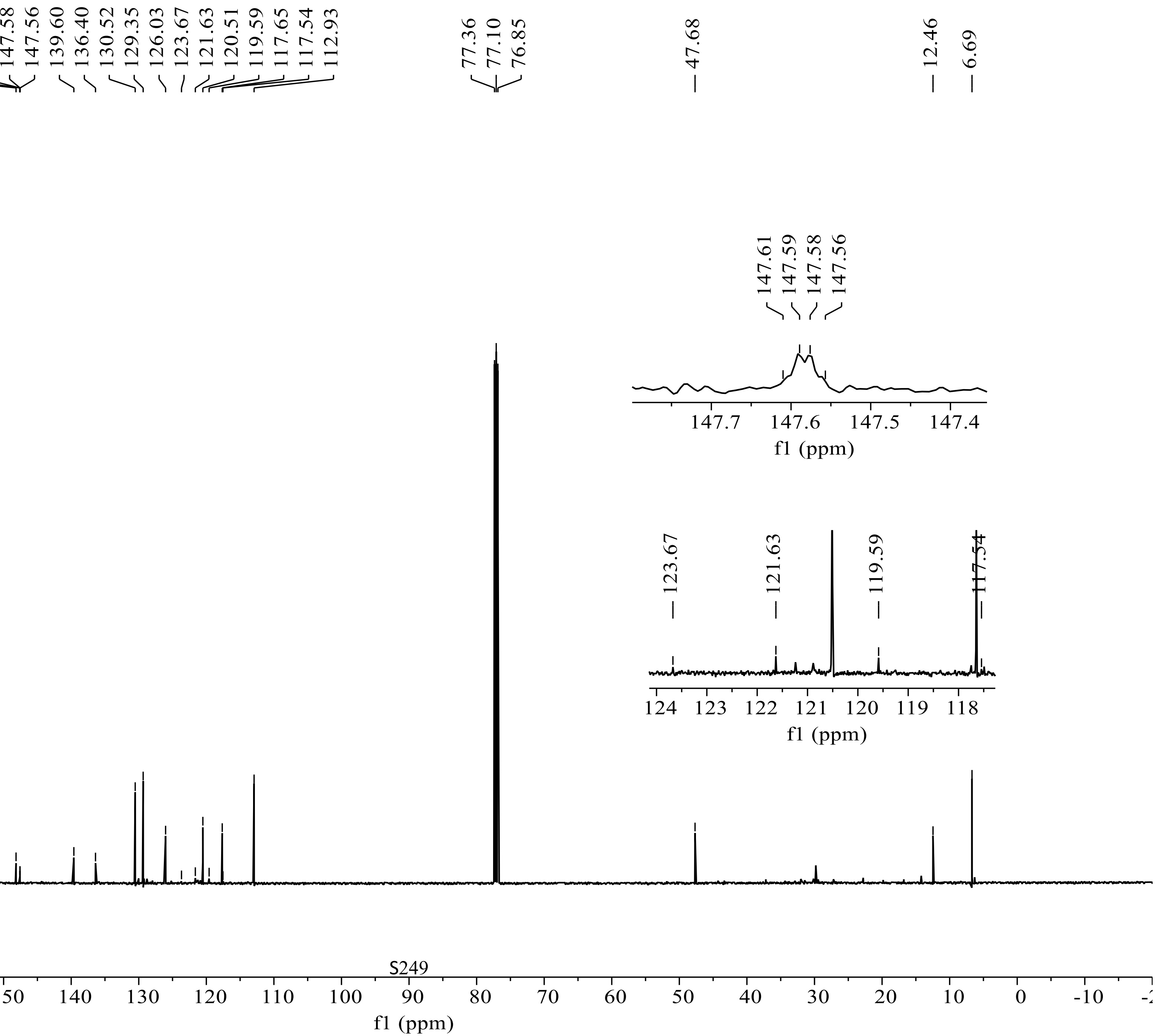
148.16
147.61
147.59
147.58
147.56
139.60
136.40
130.52
129.35
126.03
123.67
121.63
120.51
119.59
117.65
117.54
112.93
77.36
77.10
76.85
47.68
12.46
6.69
147.61
147.59
147.58
147.56
147.7
147.6
147.5
147.4
f1 (ppm)
123.67
121.63
119.59
117.54
124
123
122
121
120
119
118
f1 (ppm)
190
180
170
160
150
140
130
120
110
100
90
80
70
60
50
40
30
20
10
0
-10
f1 (ppm)

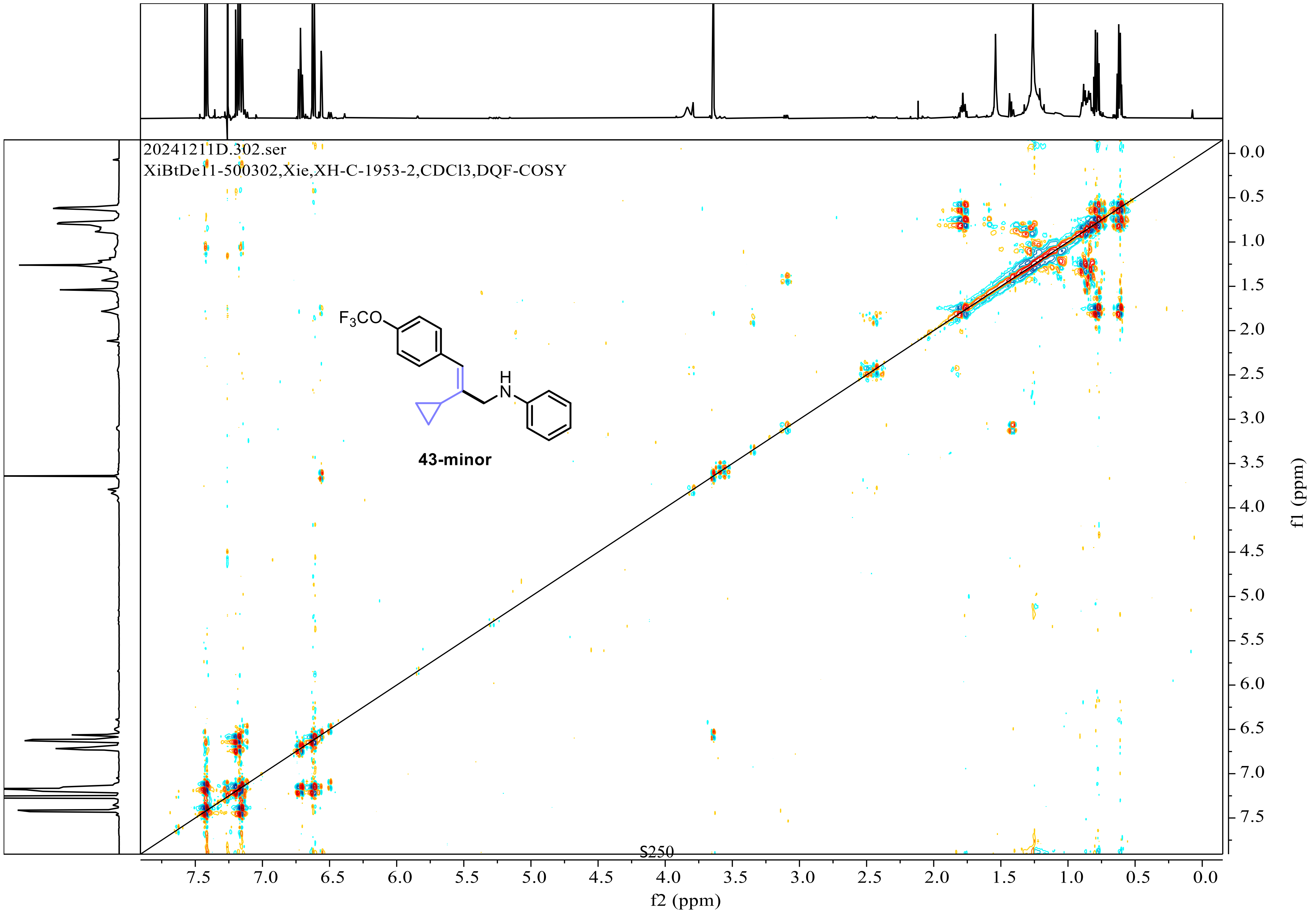

20241211D.302.ser
XiBtDe11-500302,Xie,XH-C-1953-2,CDCl3,DQF-COSY
F3CO
H
N
43-minor
f2 (ppm)
f1 (ppm)

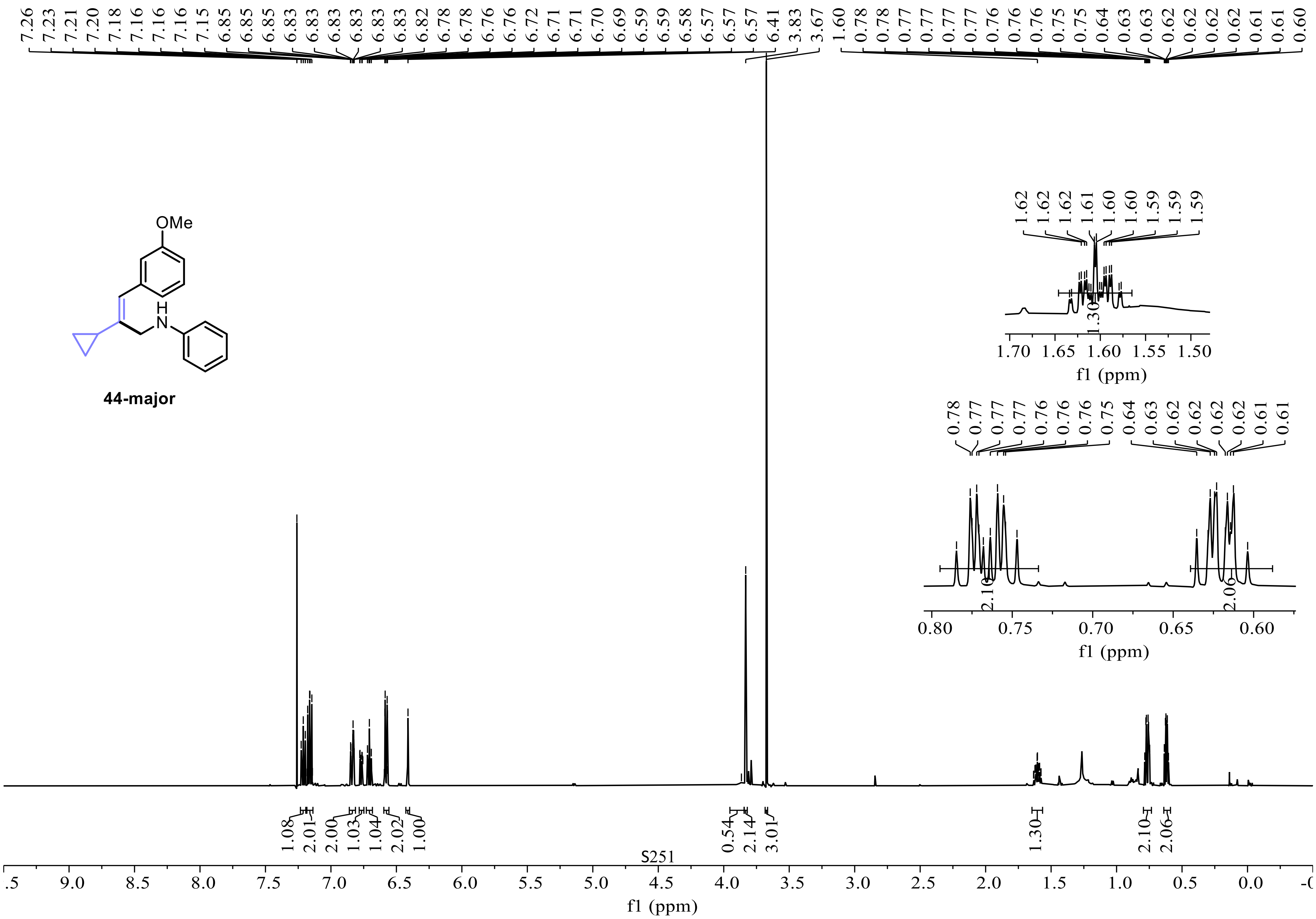
7.26 7.23 7.21 7.20 7.18 7.16 7.16 7.16 7.15 6.85 6.85 6.85 6.83 6.83 6.83 6.83 6.83 6.83 6.82 6.78 6.78 6.76 6.76 6.72 6.71 6.71 6.70 6.69 6.59 6.59 6.58 6.57 6.57 6.57 6.41 3.83 3.67 1.60 0.78 0.78 0.77 0.77 0.77 0.77 0.76 0.76 0.76 0.75 0.75 0.64 0.63 0.63 0.62 0.62 0.62 0.62 0.61 0.61 0.60
OMe
H
N
44-major
1.62 1.62 1.62 1.61 1.60 1.60 1.59 1.59 1.59
1.30
1.70 1.65 1.60 1.55 1.50
f1 (ppm)
0.78 0.77 0.77 0.77 0.76 0.76 0.76 0.75 0.64 0.63 0.62 0.62 0.62 0.62 0.61 0.61
2.10
2.06
0.80 0.75 0.70 0.65 0.60
f1 (ppm)
1.08 2.01 2.00 1.03 1.04 2.02 1.00 0.54 2.14 3.01 1.30 2.10 2.06
9.0 8.5 8.0 7.5 7.0 6.5 6.0 5.5 5.0 4.5 4.0 3.5 3.0 2.5 2.0 1.5 1.0 0.5 0.0
f1 (ppm)

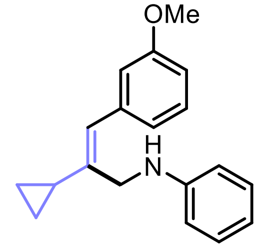
OMe
H
N

44-major

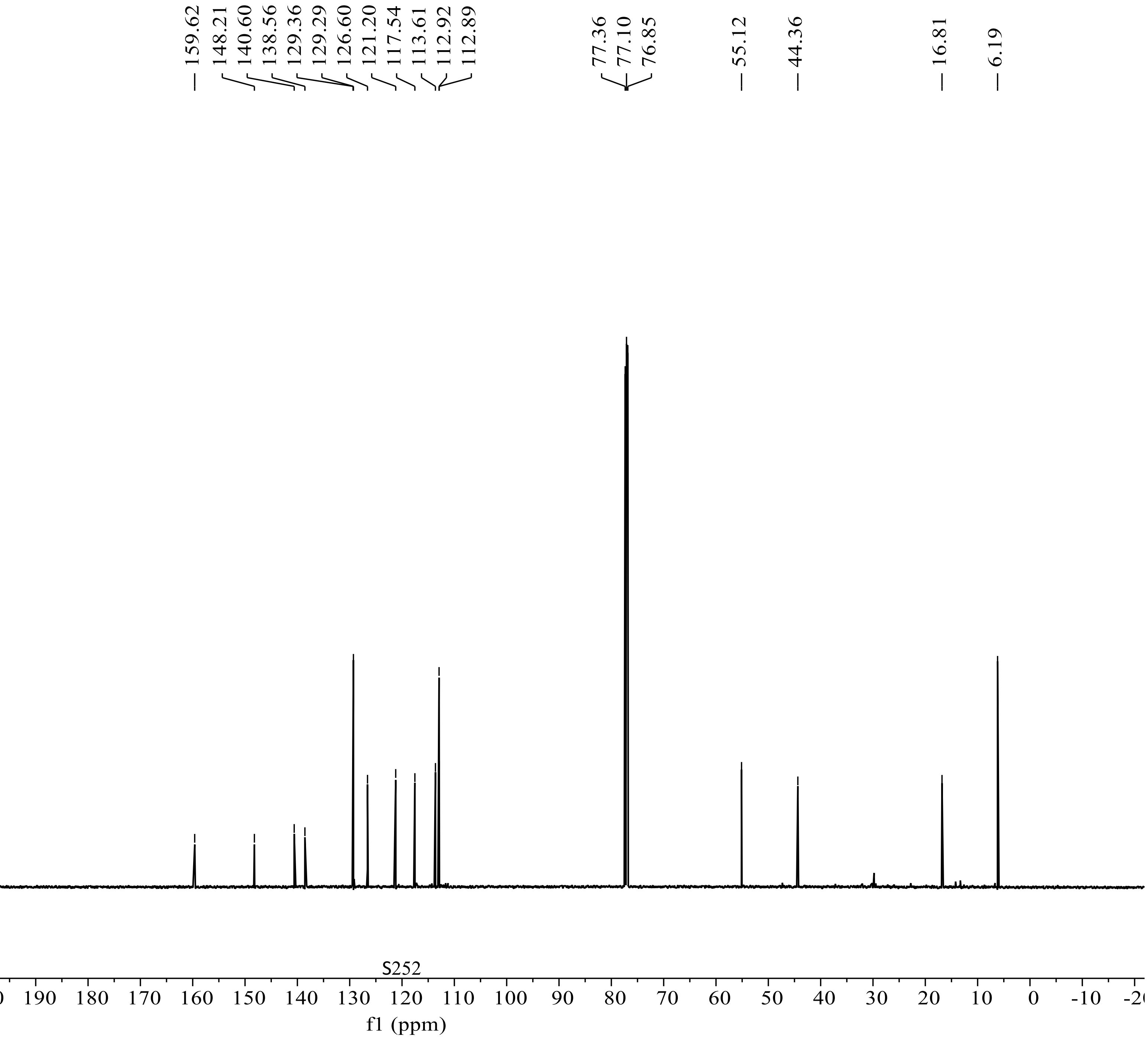
159.62
148.21
140.60
138.56
129.36
129.29
126.60
121.20
117.54
113.61
112.92
112.89
77.36
77.10
76.85
55.12
44.36
16.81
6.19
260 250 240 230 220 210 200 190 180 170 160 150 140 130 120 110 100 90 80 70 60 50 40 30 20 10 0 -10 -2
f1 (ppm)

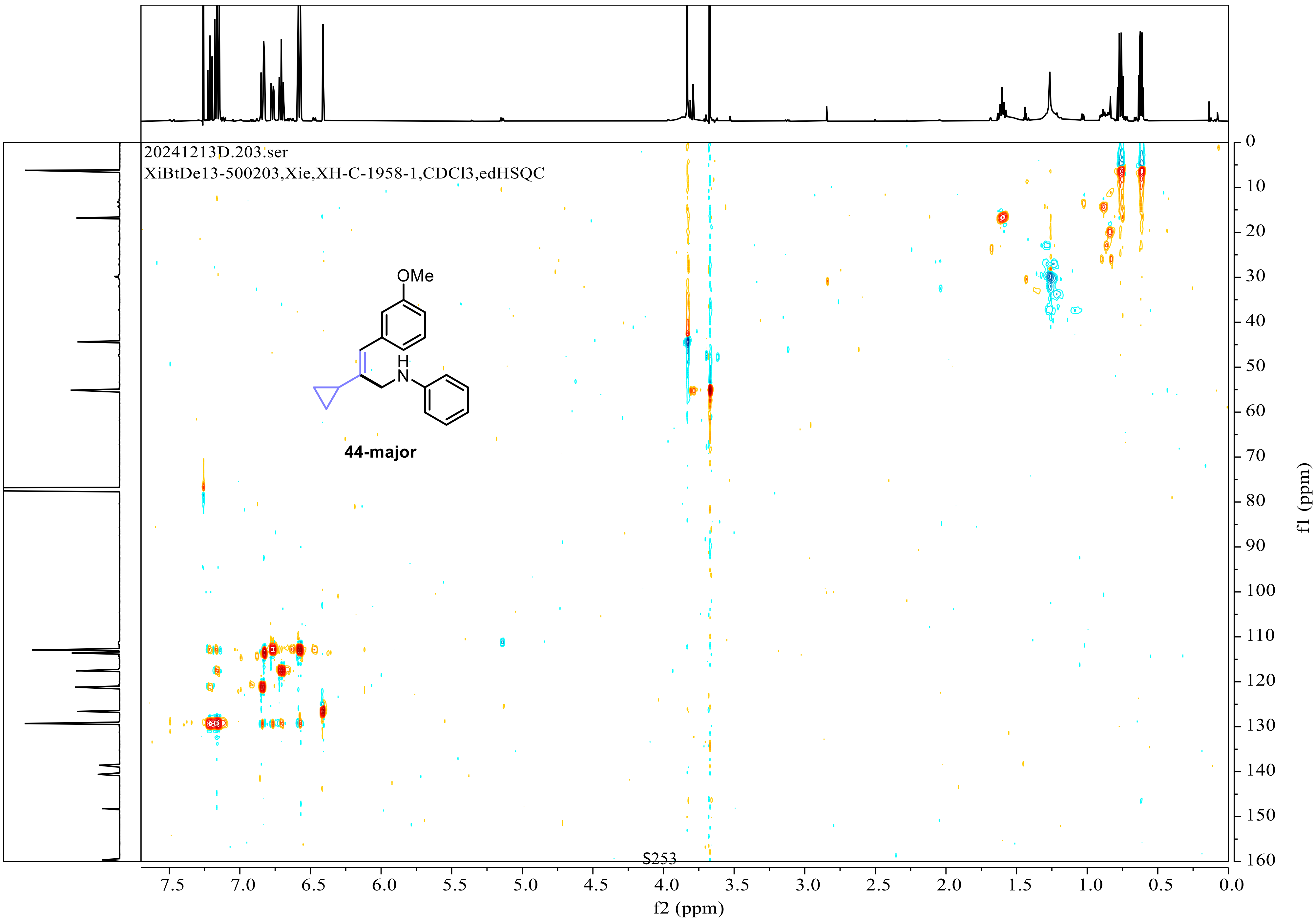
20241213D.203.ser
XiBtDe13-500203,Xie,XH-C-1958-1,CDCl3,edHSQC
OMe
H
N
44-major
f1 (ppm)
f2 (ppm)

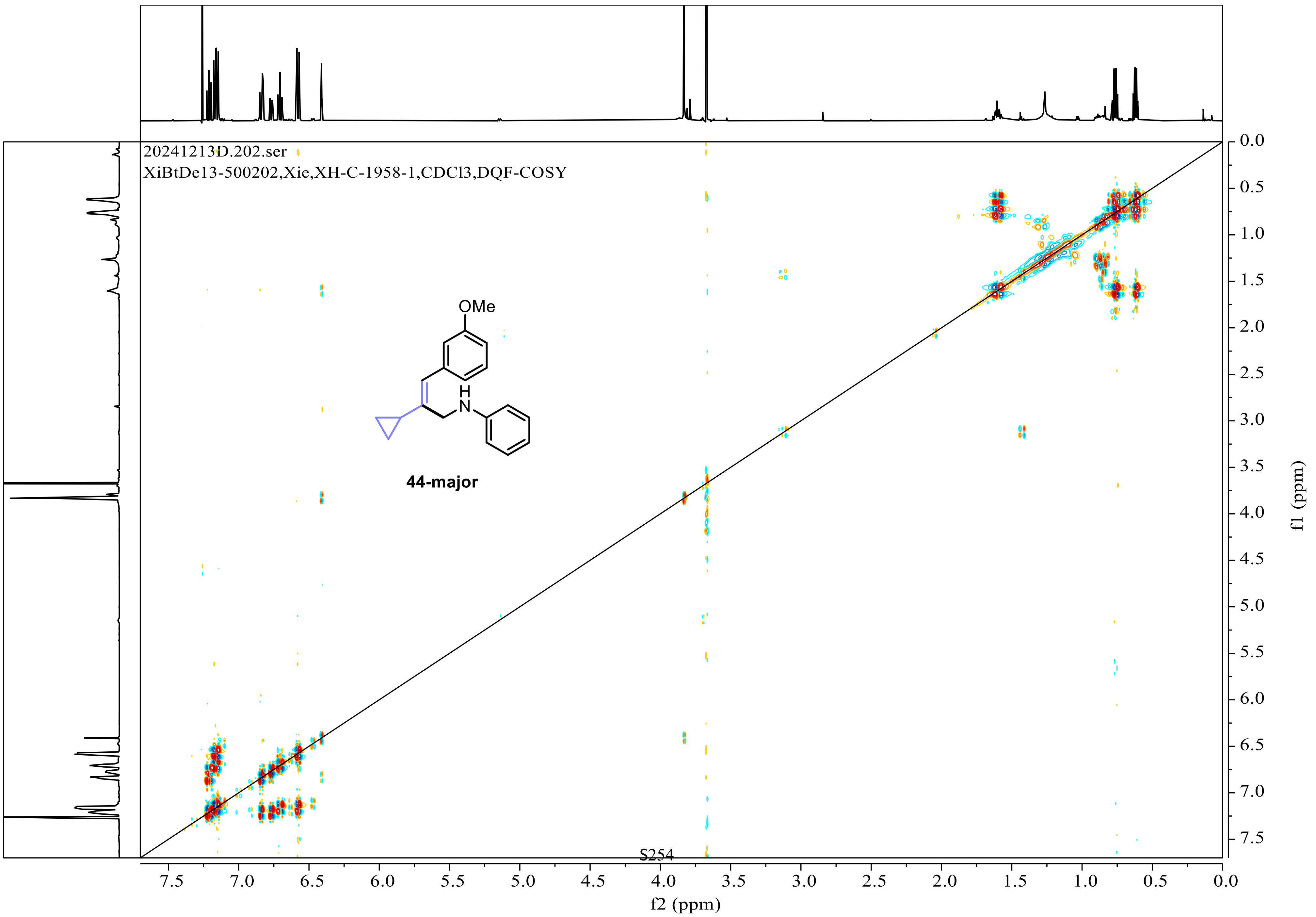
20241213D.202.ser
XiBtDe13-500202,Xie,XH-C-1958-1,CDCl3,DQF-COSY
OMe
H
N
44-major
f1 (ppm)
f2 (ppm)

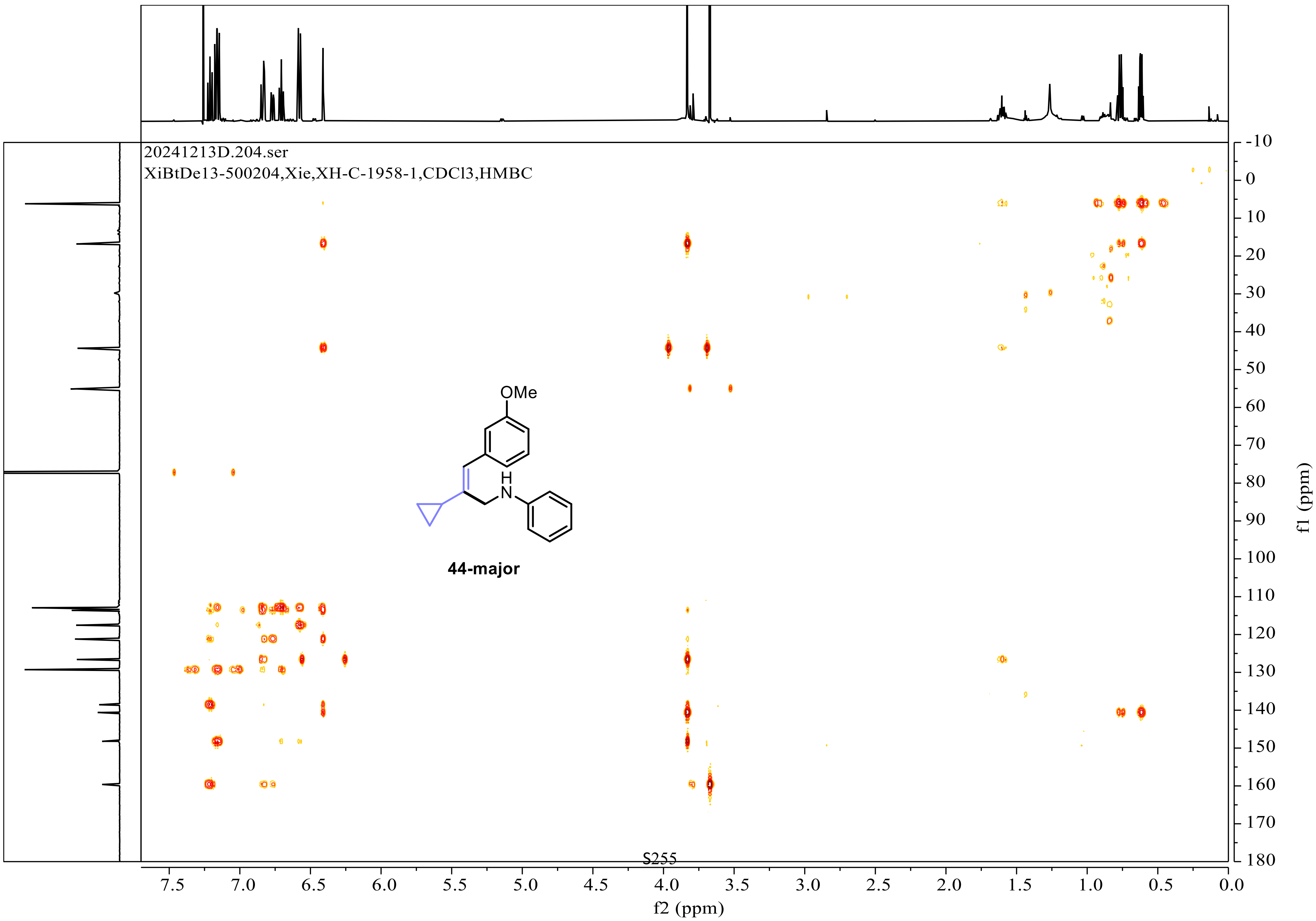
20241213D.204.ser
XiBtDe13-500204,Xie,XH-C-1958-1,CDCl3,HMBC
OMe
H
N
44-major
f2 (ppm)
f1 (ppm)

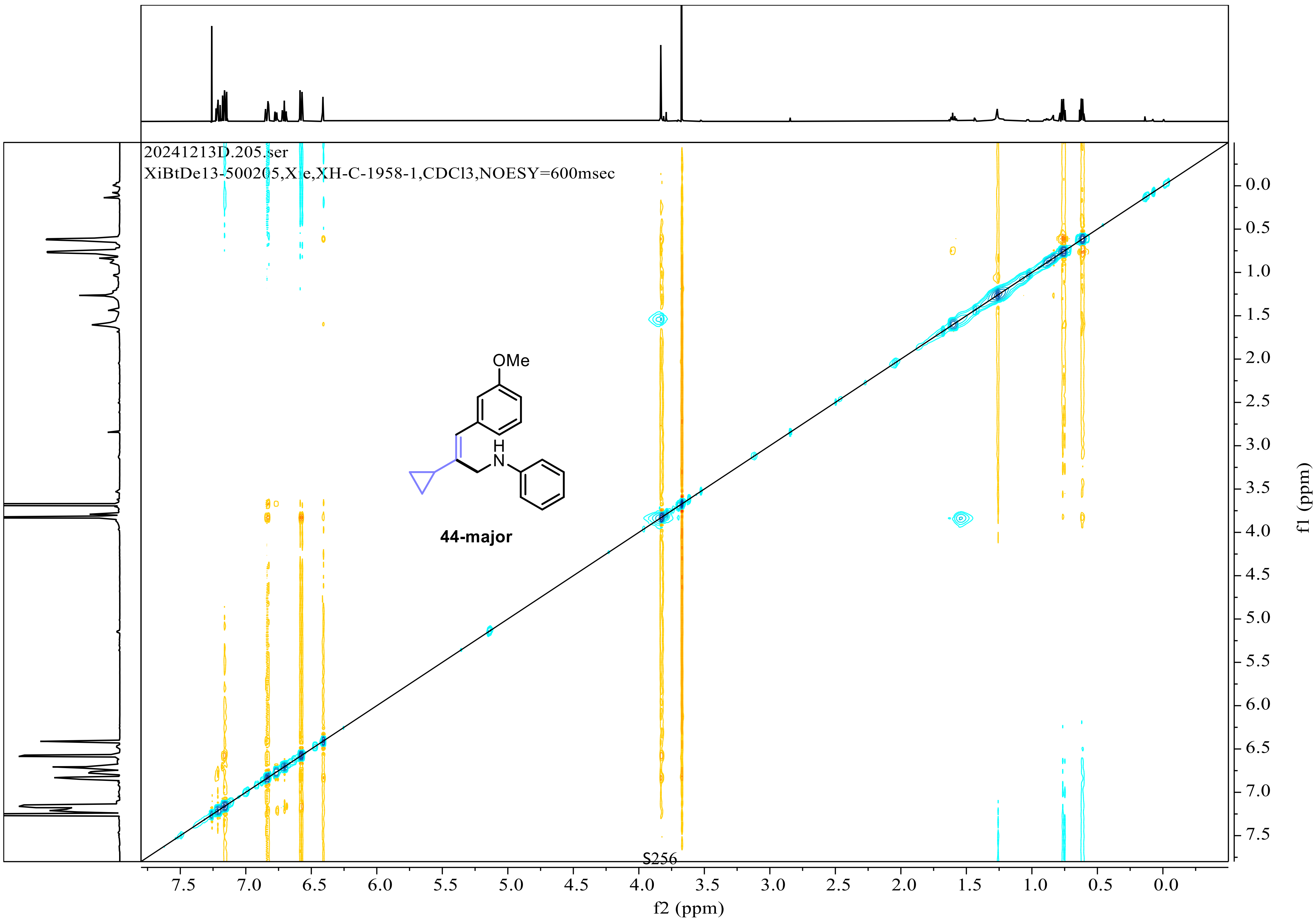
20241213D.205.ser
XiBtDe13-500205,Xie,XH-C-1958-1,CDCl3,NOESY=600msec
OMe
H
N
44-major
f2 (ppm)
f1 (ppm)

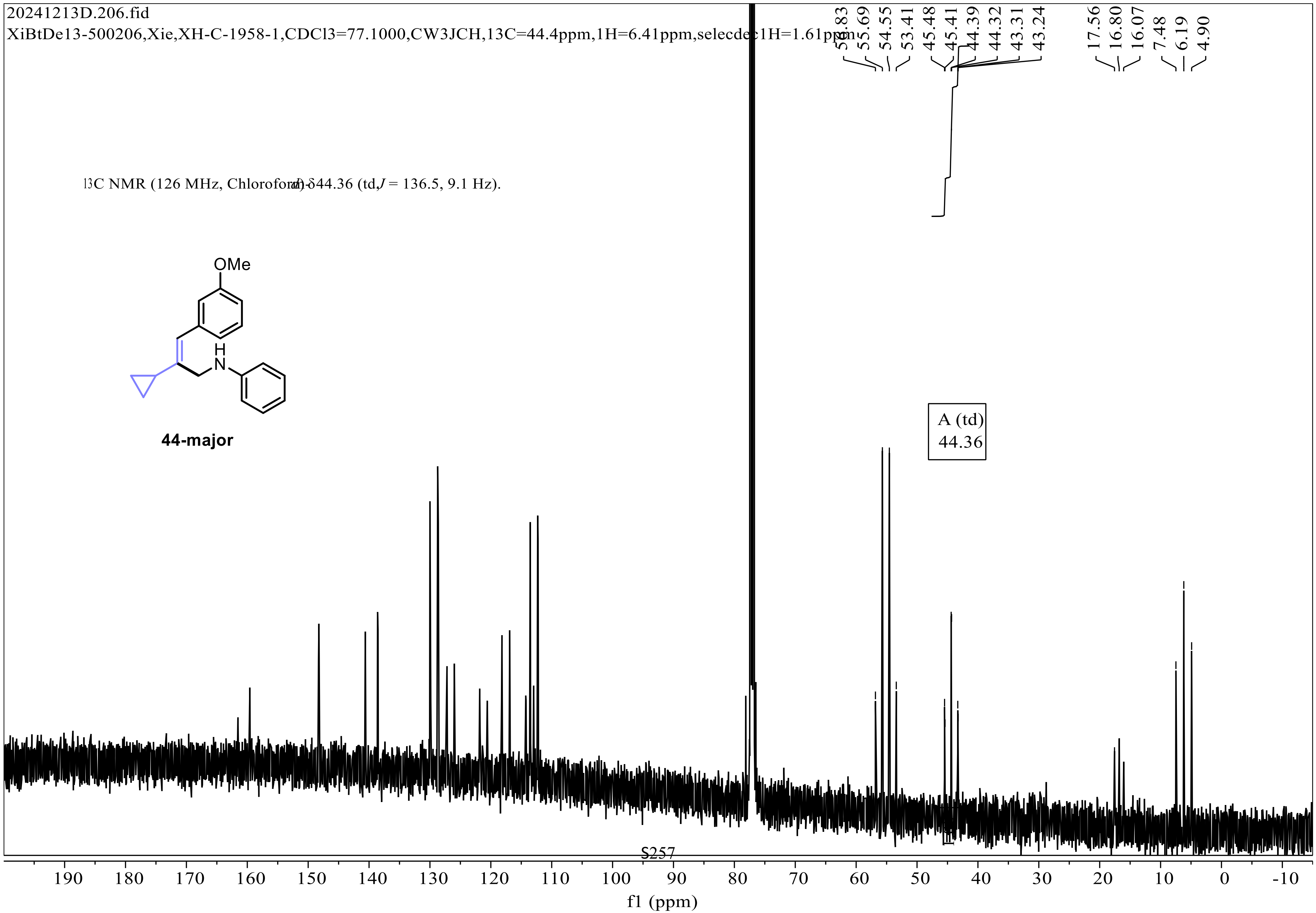
20241213D.206.fid
XiBtDe13-500206,Xie,XH-C-1958-1,CDCl3=77.1000,CW3JCH,13C=44.4ppm,1H=6.41ppm,selecdec1H=1.61ppm
55.69
54.55
53.41
45.48
45.41
44.39
44.32
43.31
43.24
17.56
16.80
16.07
7.48
6.19
4.90
13C NMR (126 MHz, Chloroform-d) δ 44.36 (td, J = 136.5, 9.1 Hz).
OMe
H
N
44-major
A (td)
44.36
190 180 170 160 150 140 130 120 110 100 90 80 70 60 50 40 30 20 10 0 -10
f1 (ppm)

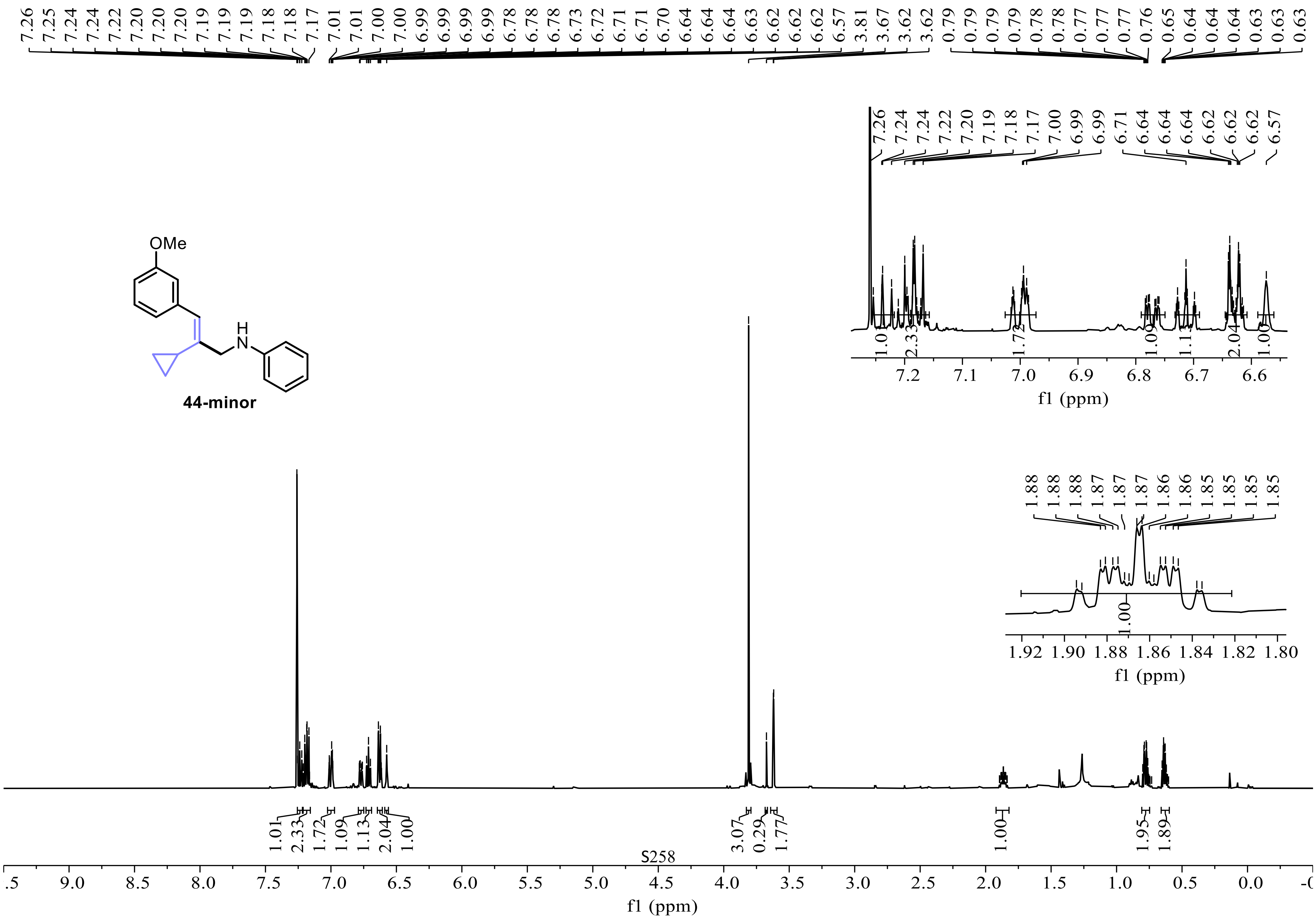
7.26 7.25 7.24 7.24 7.22 7.20 7.20 7.20 7.19 7.19 7.19 7.18 7.18 7.17 7.01 7.01 7.00 7.00 6.99 6.99 6.99 6.99 6.78 6.78 6.78 6.73 6.72 6.71 6.71 6.70 6.64 6.64 6.64 6.63 6.62 6.62 6.62 6.57 3.81 3.67 3.62 3.62 0.79 0.79 0.79 0.79 0.78 0.78 0.77 0.77 0.77 0.76 0.65 0.64 0.64 0.64 0.63 0.63 0.63
OMe
H
N
44-minor
7.26 7.24 7.24 7.22 7.20 7.19 7.18 7.17 7.00 6.99 6.99 6.71 6.64 6.64 6.64 6.62 6.62 6.62 6.57
1.01 2.33 1.72 1.09 1.13 2.04 1.00
7.2 7.1 7.0 6.9 6.8 6.7 6.6
f1 (ppm)
1.88 1.88 1.88 1.87 1.87 1.87 1.86 1.86 1.85 1.85 1.85 1.85
1.00
1.92 1.90 1.88 1.86 1.84 1.82 1.80
f1 (ppm)
1.01 2.33 1.72 1.09 1.13 2.04 1.00 3.07 0.29 1.77 1.00 1.95 1.89
.5 9.0 8.5 8.0 7.5 7.0 6.5 6.0 5.5 5.0 4.5 4.0 3.5 3.0 2.5 2.0 1.5 1.0 0.5 0.0 -(
f1 (ppm)

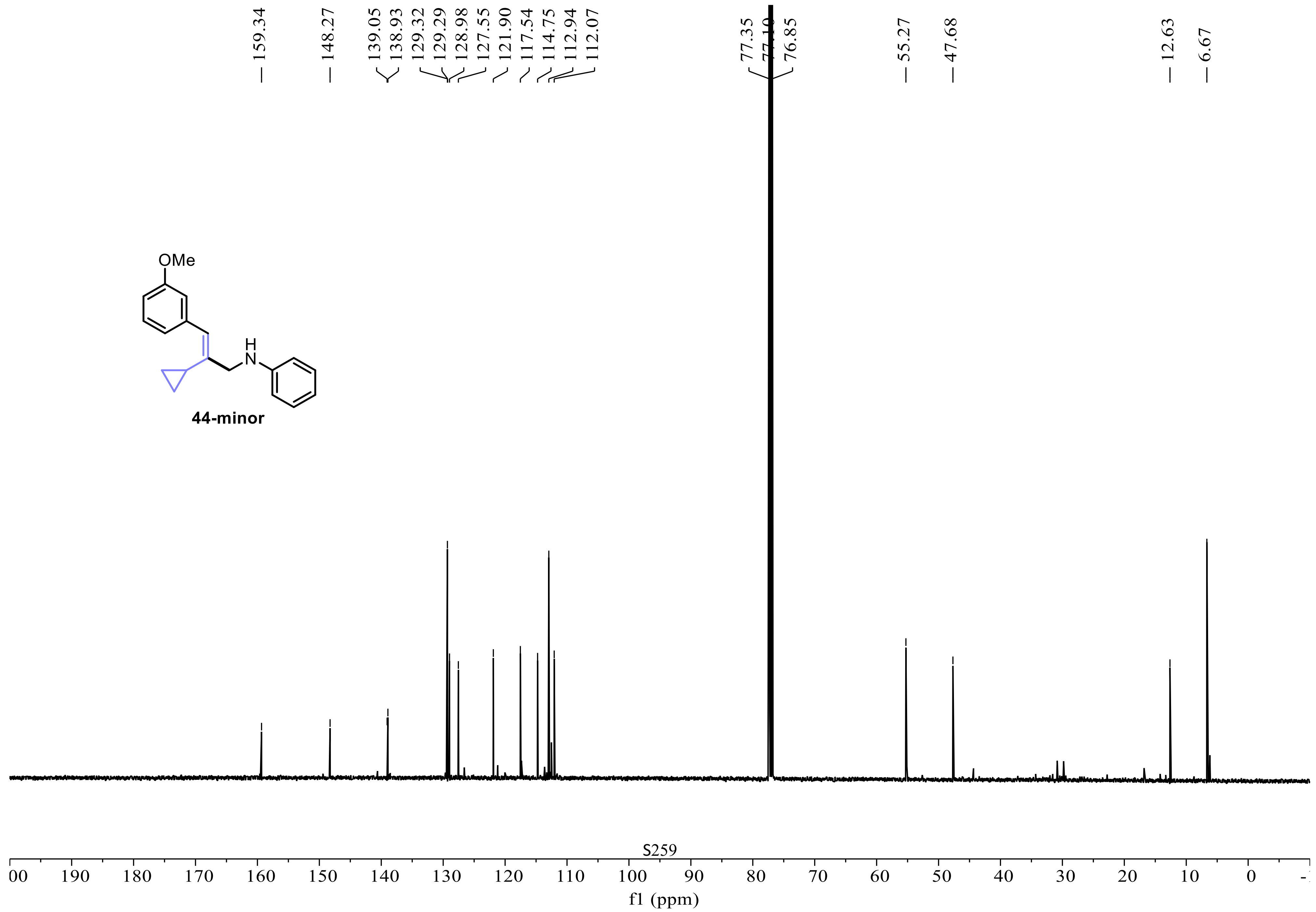
159.34
148.27
139.05
138.93
129.32
129.29
128.98
127.55
121.90
117.54
114.75
112.94
112.07
77.35
77.10
76.85
55.27
47.68
12.63
6.67
OMe
H
N
44-minor
00 190 180 170 160 150 140 130 120 110 100 90 80 70 60 50 40 30 20 10 0
f1 (ppm)

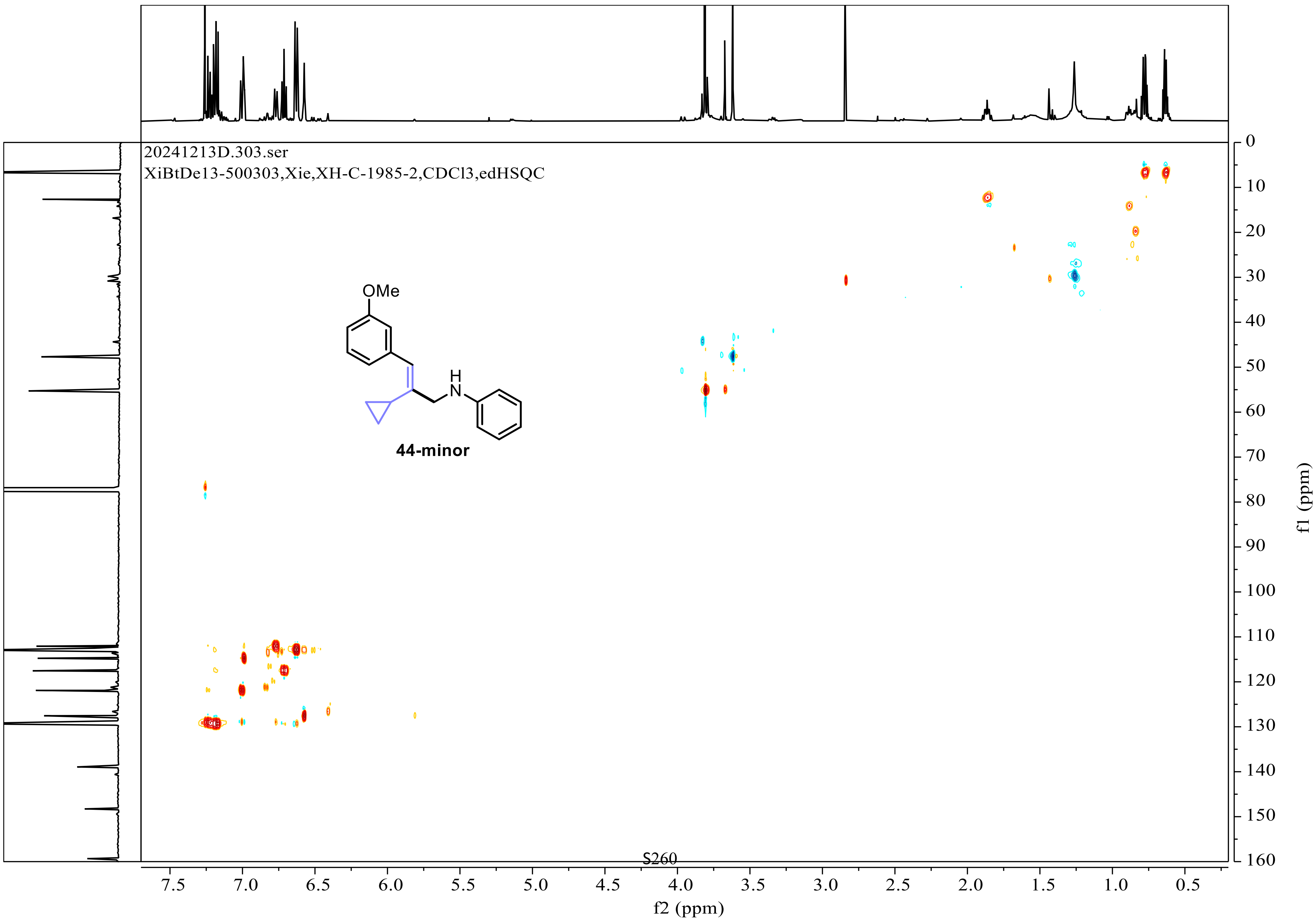
20241213D.303.ser
XiBtDe13-500303,Xie,XH-C-1985-2,CDCl3,edHSQC
OMe
H
N
44-minor
f2 (ppm)
f1 (ppm)

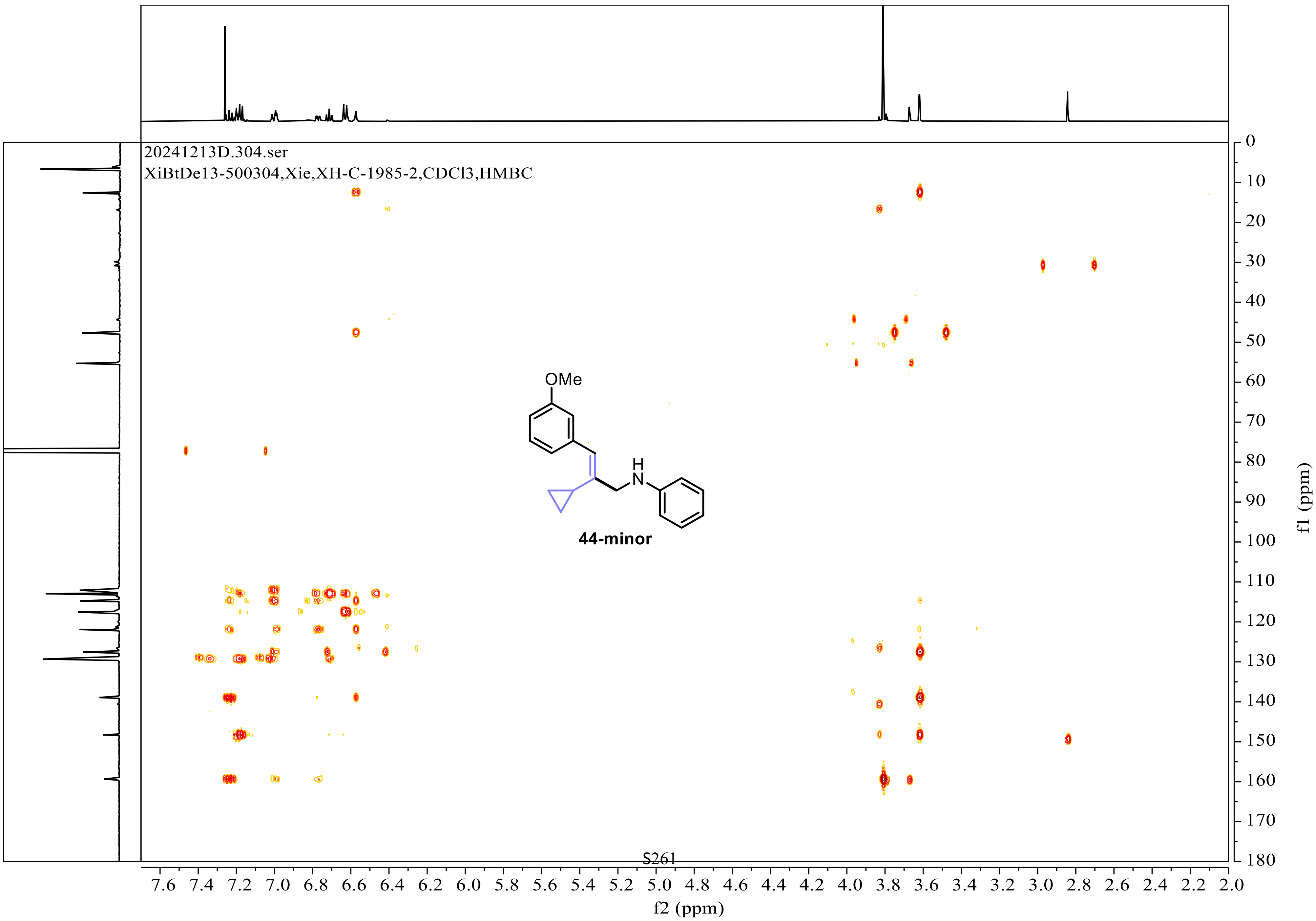
20241213D.304.ser
XiBtDe13-500304,Xie,XH-C-1985-2,CDCl3,HMBC
OMe
H
N
44-minor
f2 (ppm)
f1 (ppm)

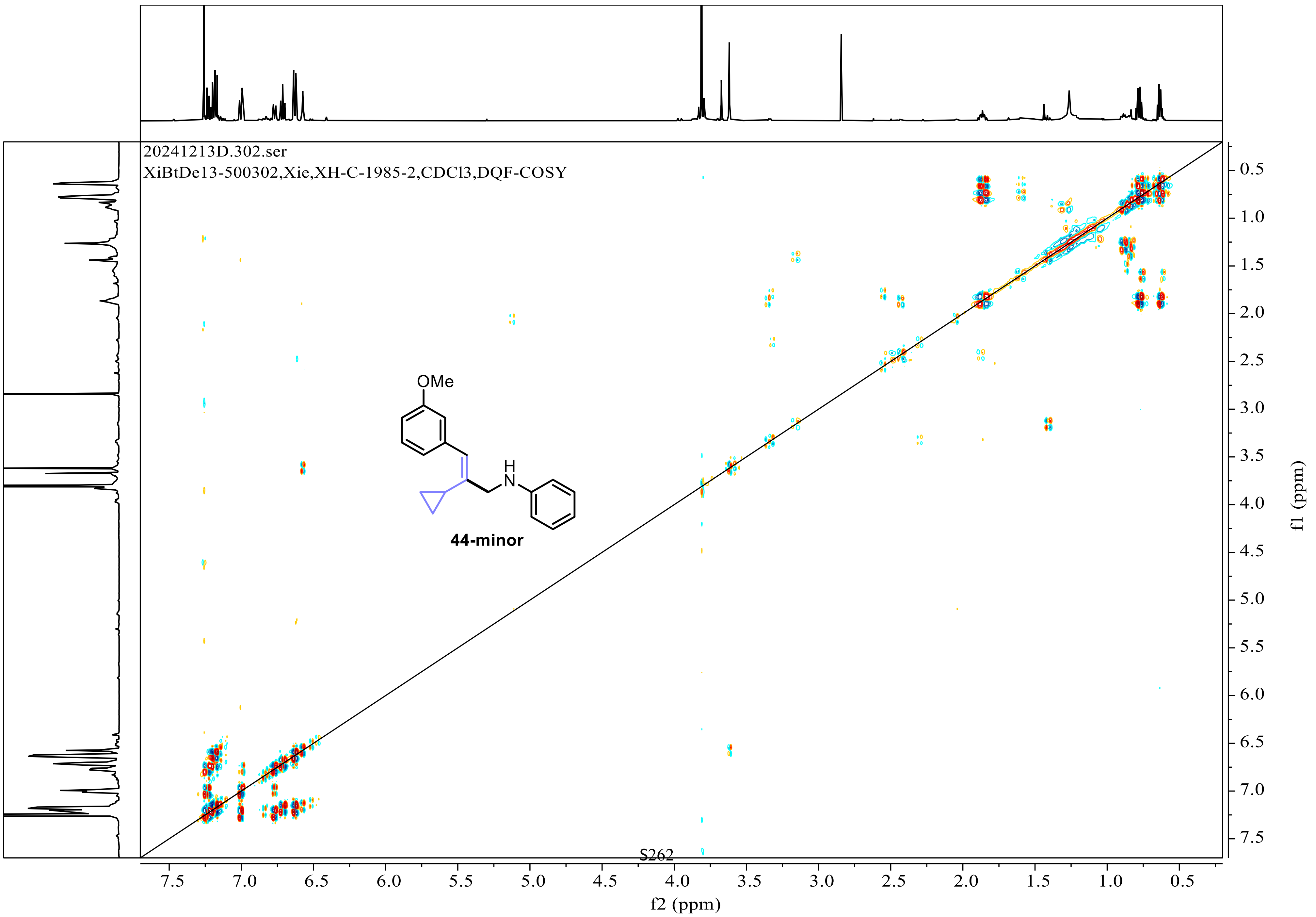

20241213D.302.ser
XiBtDe13-500302,Xie,XH-C-1985-2,CDCl3,DQF-COSY
OMe
H
N
44-minor
f2 (ppm)
f1 (ppm)

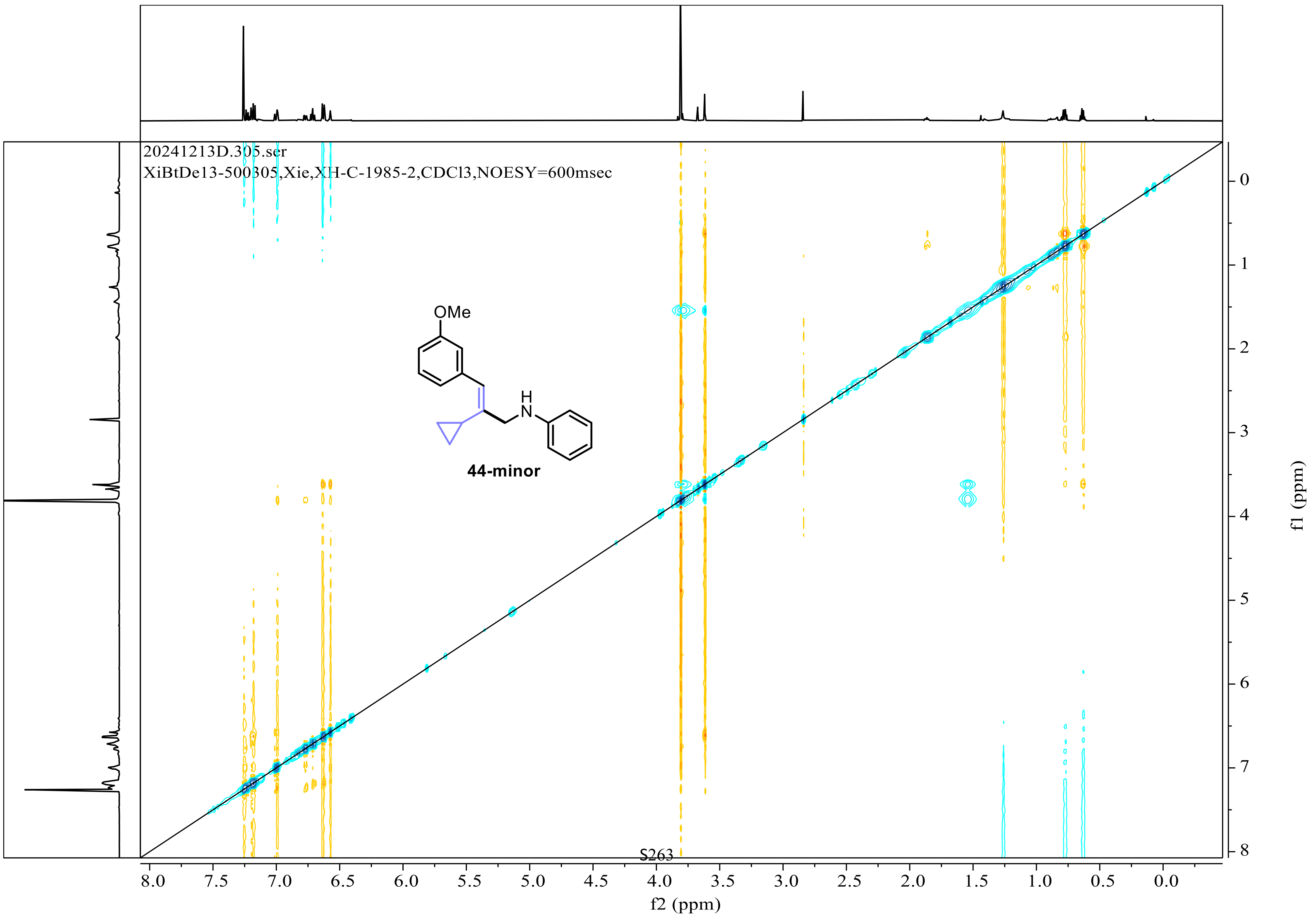

20241213D.305.ser
XiBtDe13-500305,Xie,XH-C-1985-2,CDCl3,NOESY=600msec
OMe
H
N
44-minor
f2 (ppm)
f1 (ppm)

20241213D.306.fid
XiBtDe13-500306,Xie,XH-C-1985-2,CDCl3=77.1000,CW3JCH,13C=47.7ppm,1H=6.57,1H=1.86=selecdec

13C NMR (126 MHz, Chloroform-*d*) δ 47.68 (td, *J* = 135.1, 6.6 Hz).

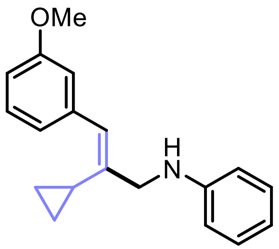
44-minor

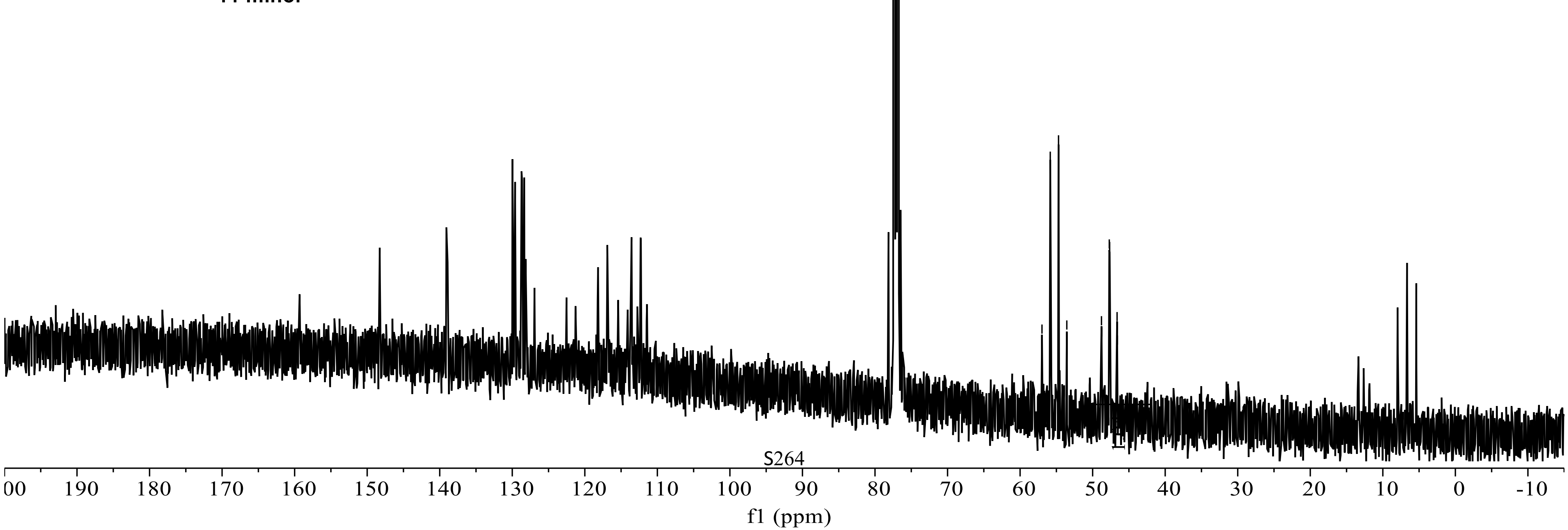

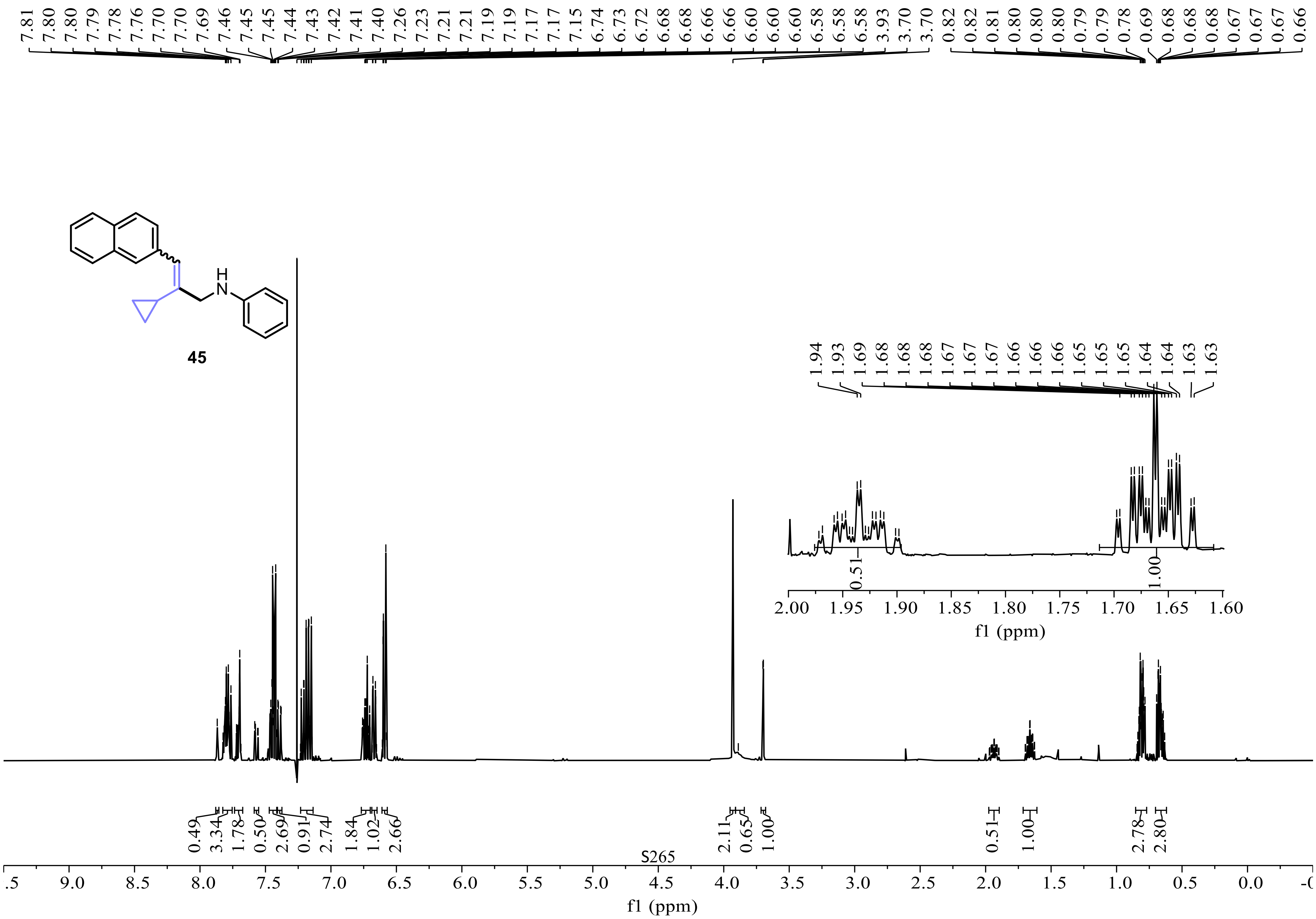

7.81 7.80 7.80 7.79 7.78 7.76 7.70 7.70 7.69 7.46 7.45 7.45 7.44 7.43 7.42 7.41 7.40 7.26 7.23 7.21 7.21 7.19 7.19 7.17 7.17 7.15 6.74 6.73 6.72 6.68 6.68 6.66 6.66 6.60 6.60 6.60 6.58 6.58 6.58 3.93 3.70 3.70 0.82 0.82 0.81 0.80 0.80 0.80 0.79 0.79 0.78 0.69 0.68 0.68 0.68 0.67 0.67 0.67 0.66
H
N
45
1.94 1.93 1.69 1.68 1.68 1.68 1.67 1.67 1.67 1.66 1.66 1.66 1.65 1.65 1.65 1.64 1.64 1.63 1.63
0.51
1.00
2.00 1.95 1.90 1.85 1.80 1.75 1.70 1.65 1.60
f1 (ppm)
0.49 3.34 1.78 0.50 2.69 0.91 2.74 1.84 1.02 2.66 2.11 0.65 1.00 0.51 1.00 2.78 2.80
.5 9.0 8.5 8.0 7.5 7.0 6.5 6.0 5.5 5.0 4.5 4.0 3.5 3.0 2.5 2.0 1.5 1.0 0.5 0.0
f1 (ppm)

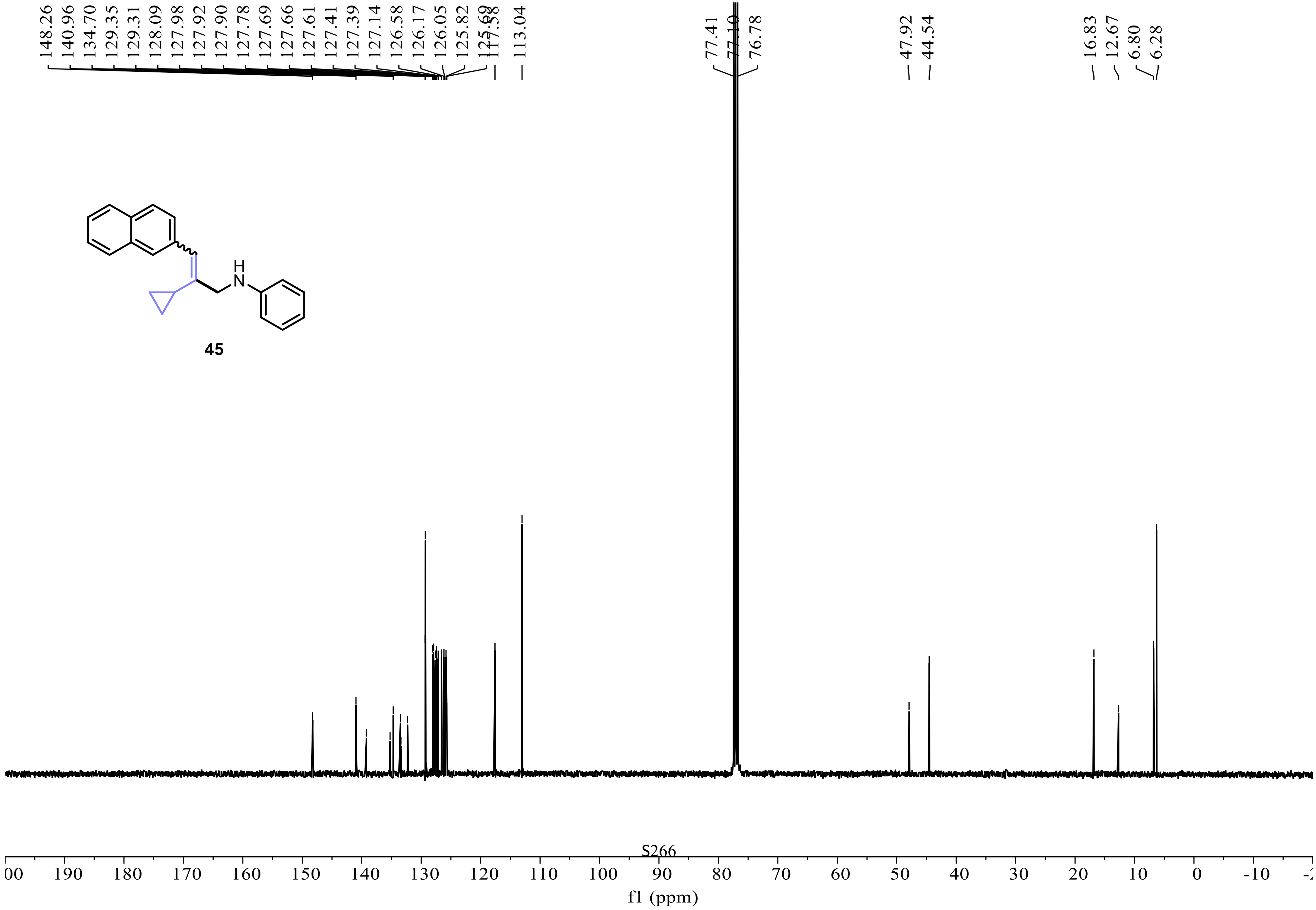
148.26
140.96
134.70
129.35
129.31
128.09
127.98
127.92
127.90
127.78
127.69
127.66
127.61
127.41
127.39
127.14
126.58
126.17
126.05
125.82
125.69
117.58
113.04
77.41
77.10
76.78
47.92
44.54
16.83
12.67
6.80
6.28
H
N
45
f1 (ppm)

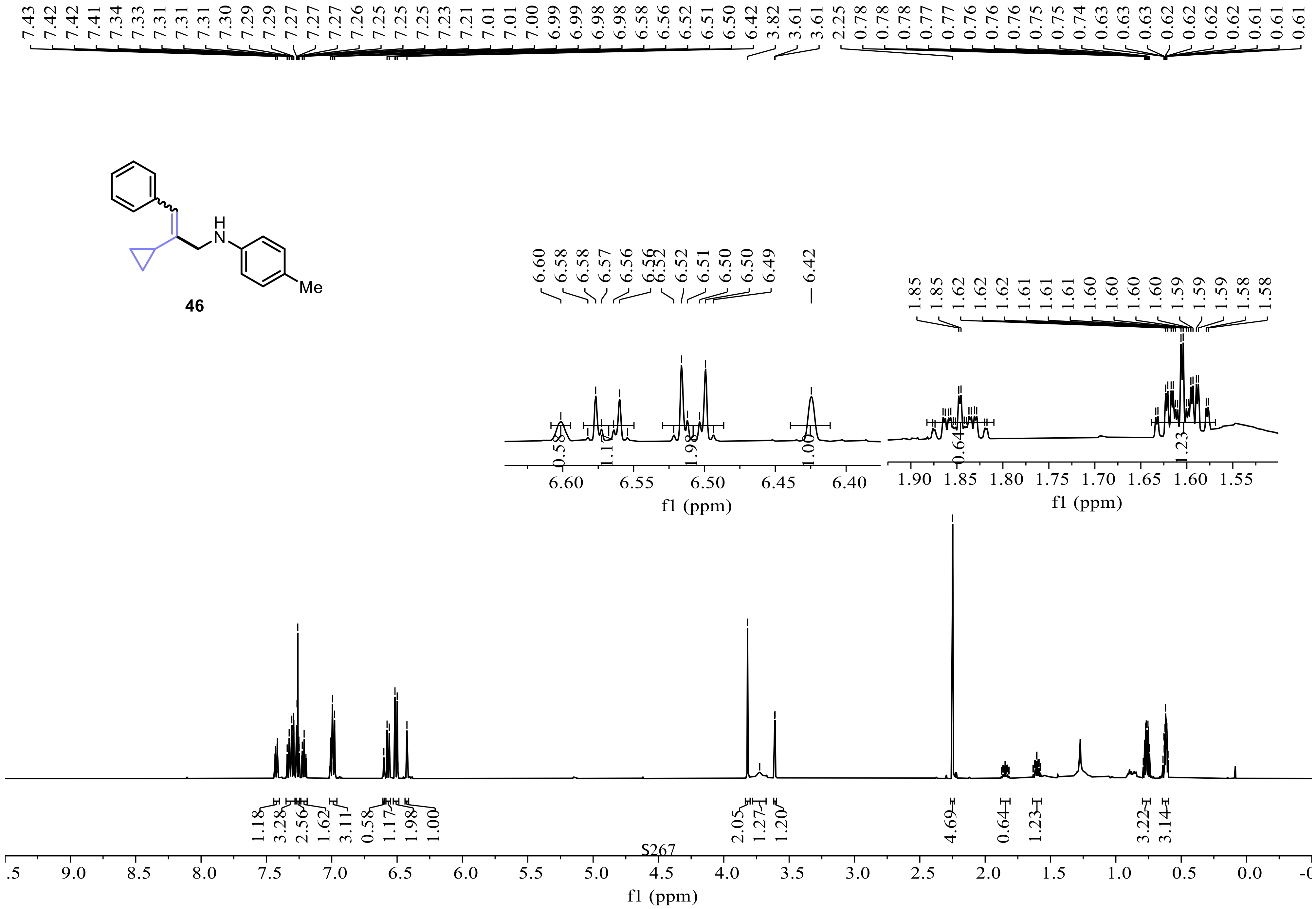

46

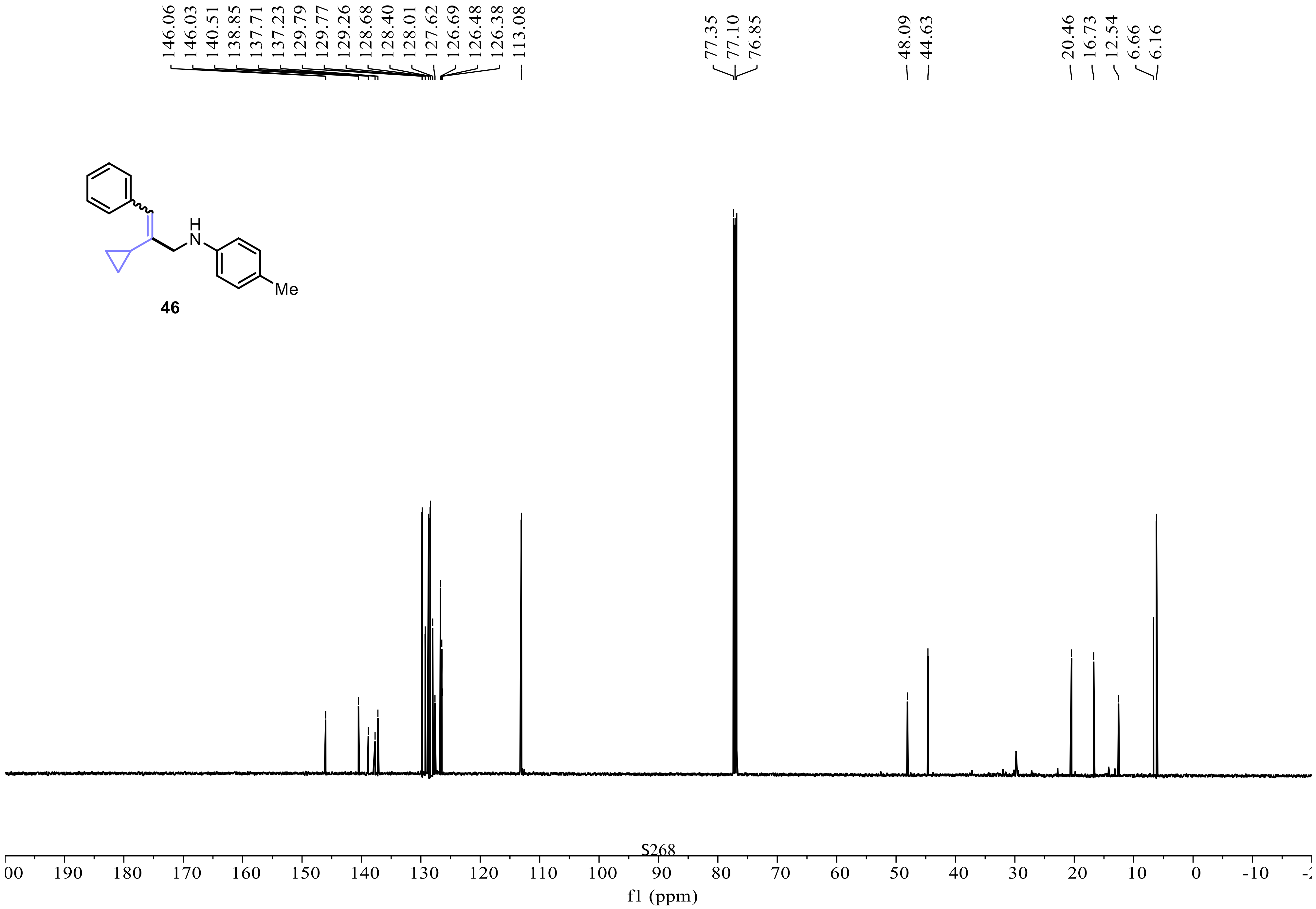
146.06
146.03
140.51
138.85
137.71
137.23
129.79
129.77
129.26
128.68
128.40
128.01
127.62
126.69
126.48
126.38
113.08
77.35
77.10
76.85
48.09
44.63
20.46
16.73
12.54
6.66
6.16
H
N
Me
46
f1 (ppm)

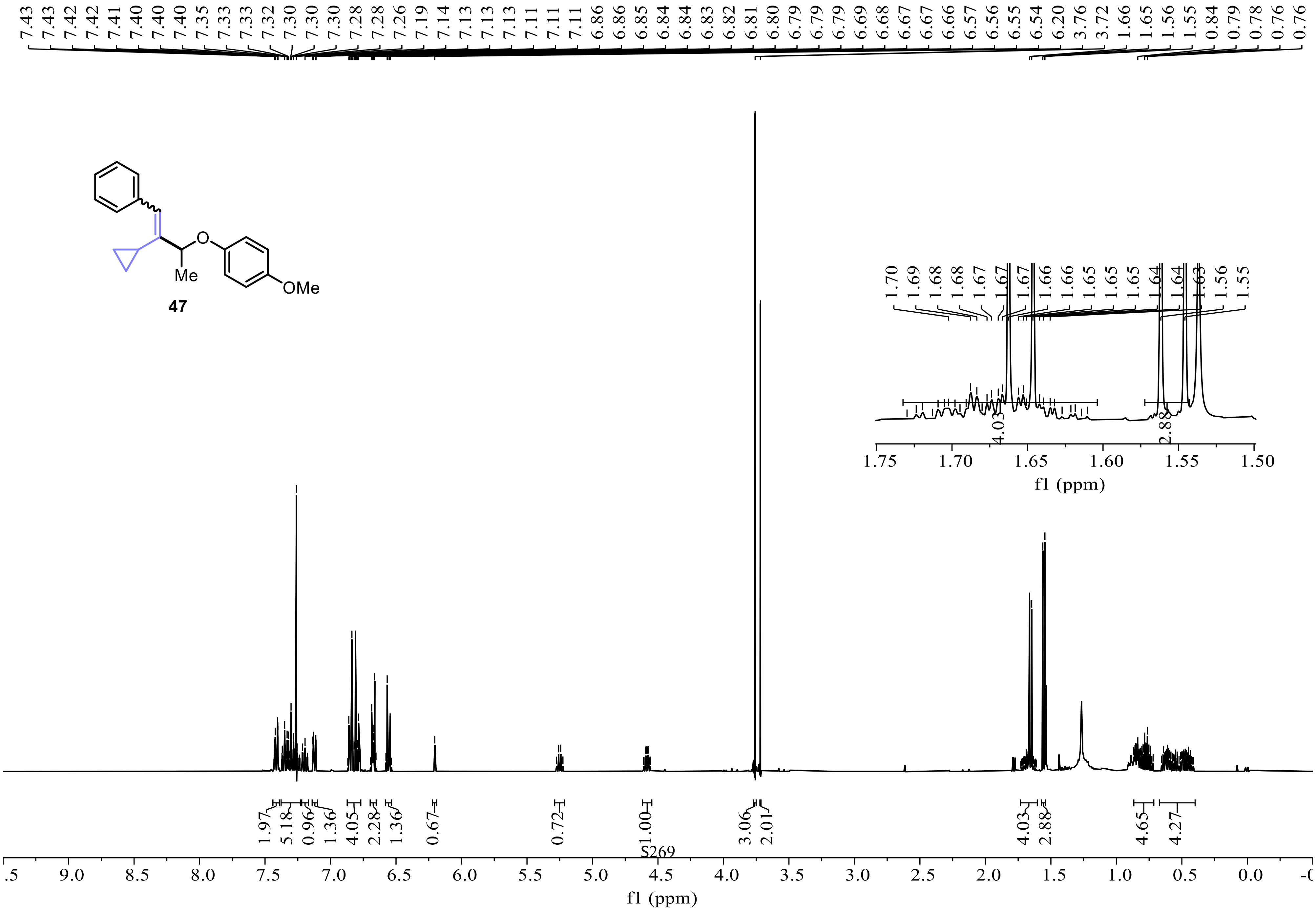
7.43 7.43 7.42 7.42 7.41 7.40 7.40 7.40 7.35 7.33 7.33 7.32 7.30 7.30 7.30 7.28 7.28 7.26 7.19 7.14 7.13 7.13 7.13 7.11 7.11 7.11 6.86 6.86 6.85 6.84 6.84 6.83 6.82 6.81 6.80 6.79 6.79 6.79 6.69 6.68 6.67 6.67 6.66 6.57 6.56 6.55 6.54 6.20 3.76 3.72 1.66 1.65 1.56 1.55 0.84 0.79 0.78 0.76 0.76
O
Me
OMe
47
1.70 1.69 1.68 1.68 1.67 1.67 1.67 1.66 1.66 1.65 1.65 1.65 1.64 1.64 1.63 1.56 1.55
4.03
2.88
1.75 1.70 1.65 1.60 1.55 1.50
f1 (ppm)
1.97 5.18 0.96 1.36 4.05 2.28 1.36 0.67 0.72 1.00 3.06 2.01 4.03 2.88 4.65 4.27
.5 9.0 8.5 8.0 7.5 7.0 6.5 6.0 5.5 5.0 4.5 4.0 3.5 3.0 2.5 2.0 1.5 1.0 0.5 0.0
f1 (ppm)

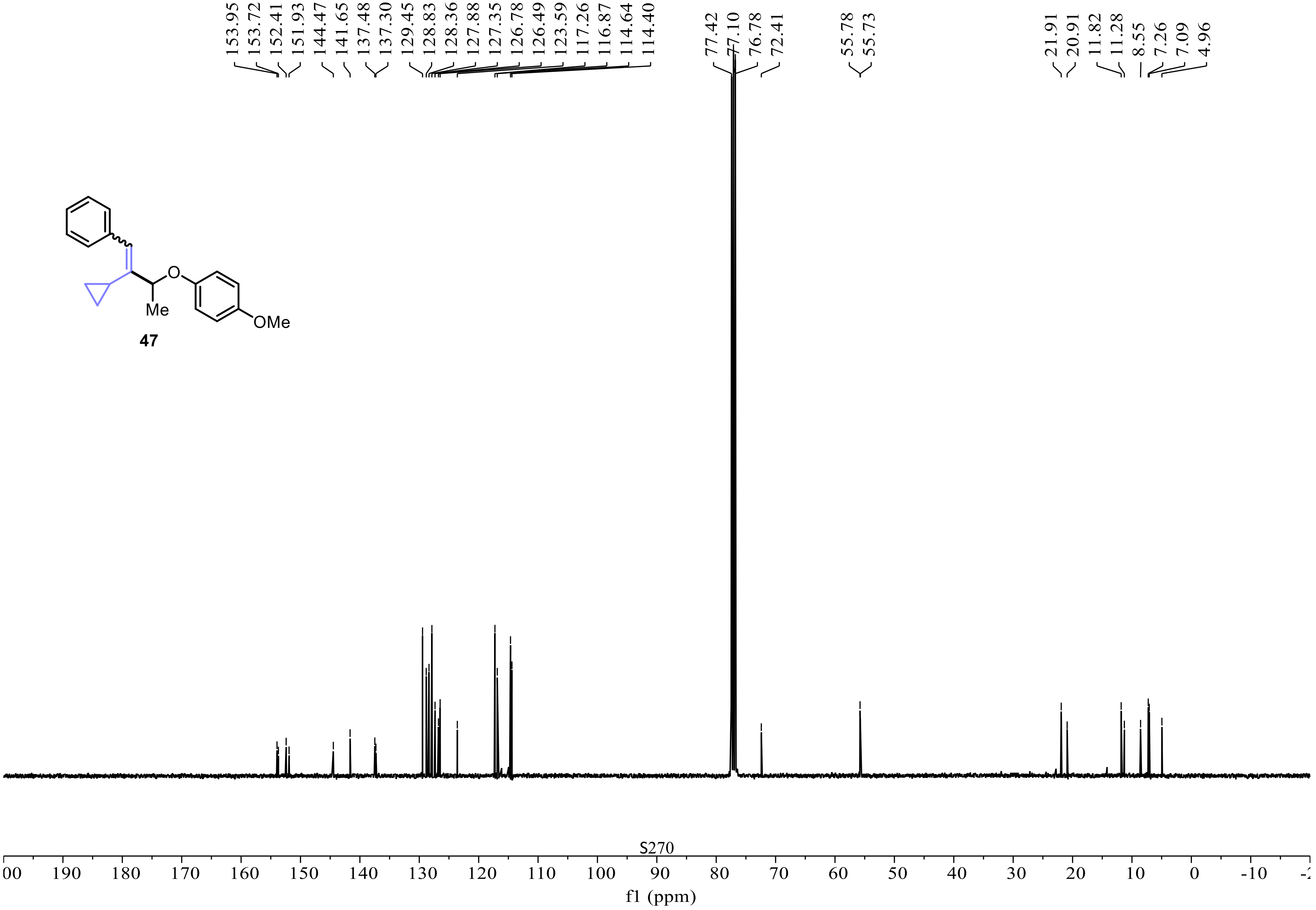
153.95
153.72
152.41
151.93
144.47
141.65
137.48
137.30
129.45
128.83
128.36
127.88
127.35
126.78
126.49
123.59
117.26
116.87
114.64
114.40
77.42
77.10
76.78
72.41
55.78
55.73
21.91
20.91
11.82
11.28
8.55
7.26
7.09
4.96
O
Me
OMe
47
00 190 180 170 160 150 140 130 120 110 100 90 80 70 60 50 40 30 20 10 0 -10
f1 (ppm)

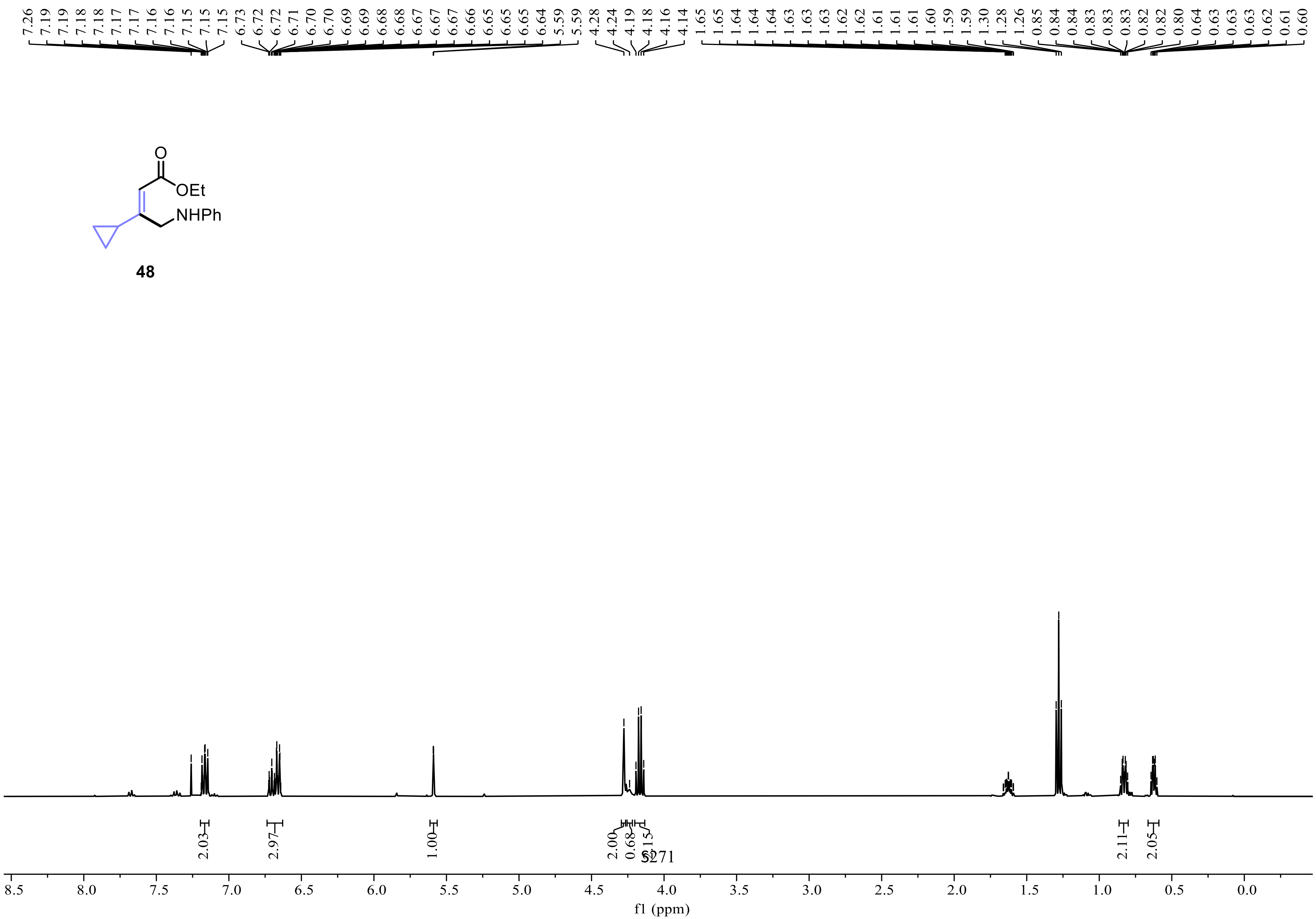
7.26 7.19 7.19 7.18 7.18 7.17 7.17 7.16 7.16 7.15 7.15 7.15 6.73 6.72 6.72 6.71 6.70 6.70 6.69 6.69 6.68 6.68 6.67 6.67 6.67 6.66 6.65 6.65 6.65 6.64 5.59 5.59 4.28 4.24 4.19 4.18 4.16 4.14 1.65 1.65 1.64 1.64 1.64 1.63 1.63 1.63 1.62 1.62 1.61 1.61 1.61 1.60 1.59 1.59 1.30 1.28 1.26 0.85 0.84 0.84 0.83 0.83 0.83 0.82 0.82 0.80 0.64 0.63 0.63 0.63 0.62 0.61 0.60
O
OEt
NHPh
48
2.03
2.97
1.00
2.00
0.68
2.15
2.11
2.05
8.5 8.0 7.5 7.0 6.5 6.0 5.5 5.0 4.5 4.0 3.5 3.0 2.5 2.0 1.5 1.0 0.5 0.0
f1 (ppm)

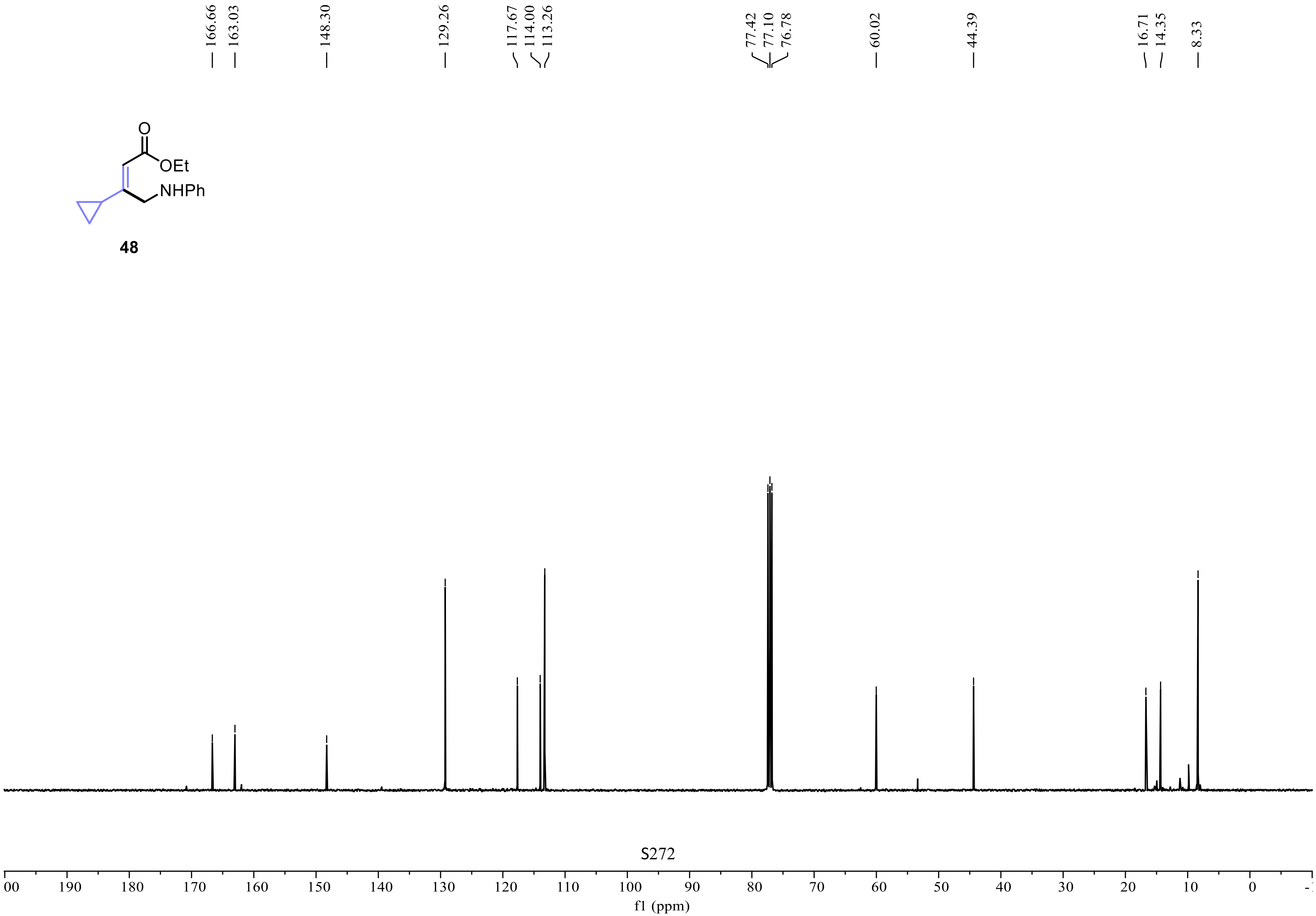
166.66
163.03
148.30
129.26
117.67
114.00
113.26
77.42
77.10
76.78
60.02
44.39
16.71
14.35
8.33
O
OEt
NHPh
200 190 180 170 160 150 140 130 120 110 100 90 80 70 60 50 40 30 20 10 0 -10
f1 (ppm)

48

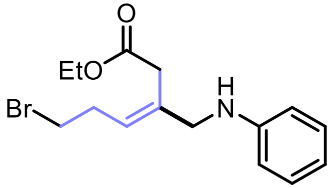
EtO
O
Br
H
N

48'

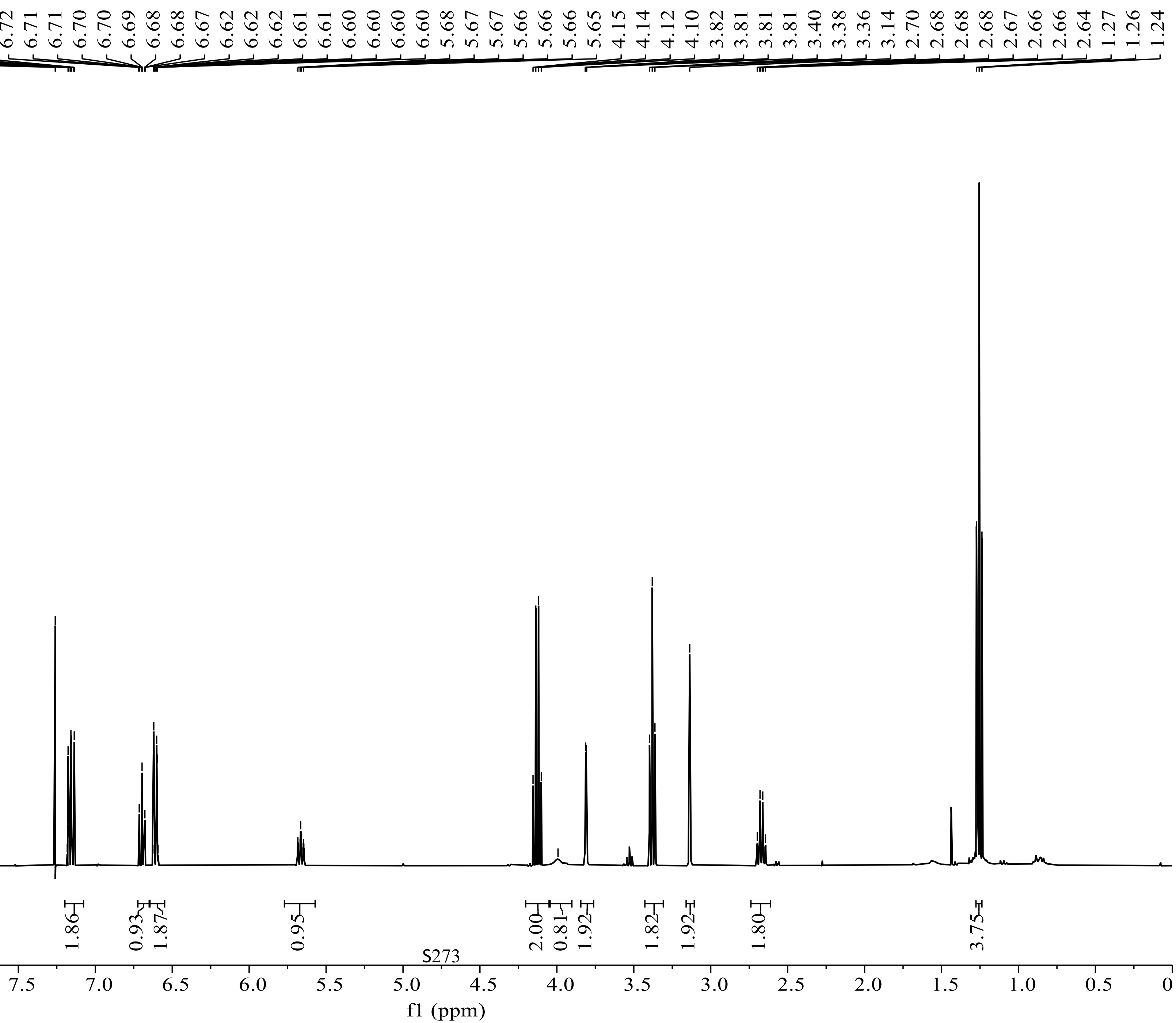
7.26 7.18 7.17 7.17 7.16 7.16 7.15 7.15 7.14 7.14 7.14 6.72 6.71 6.71 6.70 6.70 6.69 6.68 6.68 6.67 6.62 6.62 6.62 6.61 6.61 6.60 6.60 6.60 6.60 5.68 5.67 5.67 5.66 5.66 5.66 5.65 4.15 4.14 4.12 4.10 3.82 3.81 3.81 3.81 3.40 3.38 3.36 3.14 2.70 2.68 2.68 2.68 2.67 2.66 2.66 2.64 1.27 1.26 1.24
1.86
0.93
1.87
0.95
2.00
0.81
1.92
1.82
1.92
1.80
3.75
9.0 8.5 8.0 7.5 7.0 6.5 6.0 5.5 5.0 4.5 4.0 3.5 3.0 2.5 2.0 1.5 1.0 0.5 0
f1 (ppm)

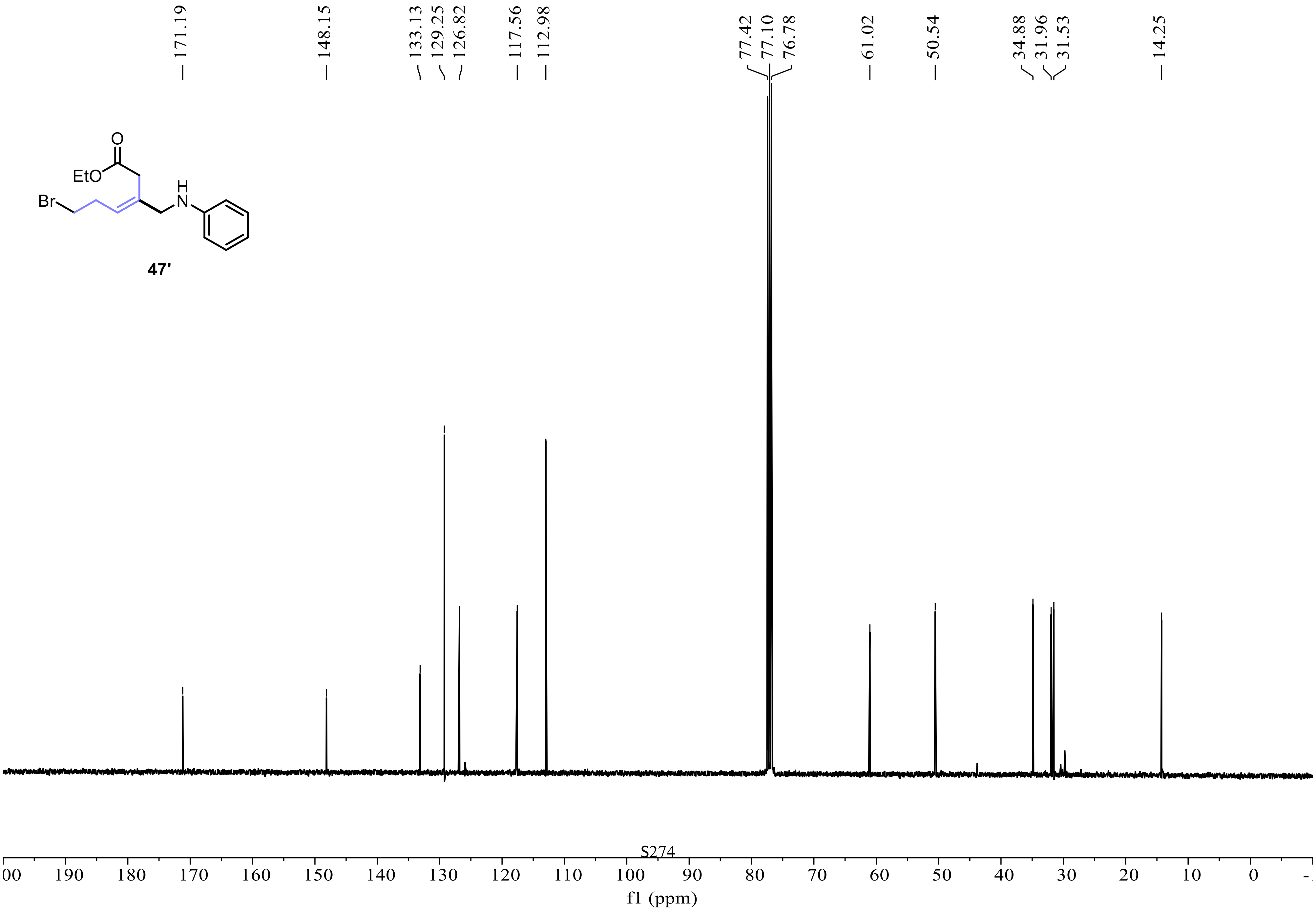

EtO
O
Br
H
N
47'
171.19
148.15
133.13
129.25
126.82
117.56
112.98
77.42
77.10
76.78
61.02
50.54
34.88
31.96
31.53
14.25
00
190
180
170
160
150
140
130
120
110
100
90
80
70
60
50
40
30
20
10
0
f1 (ppm)

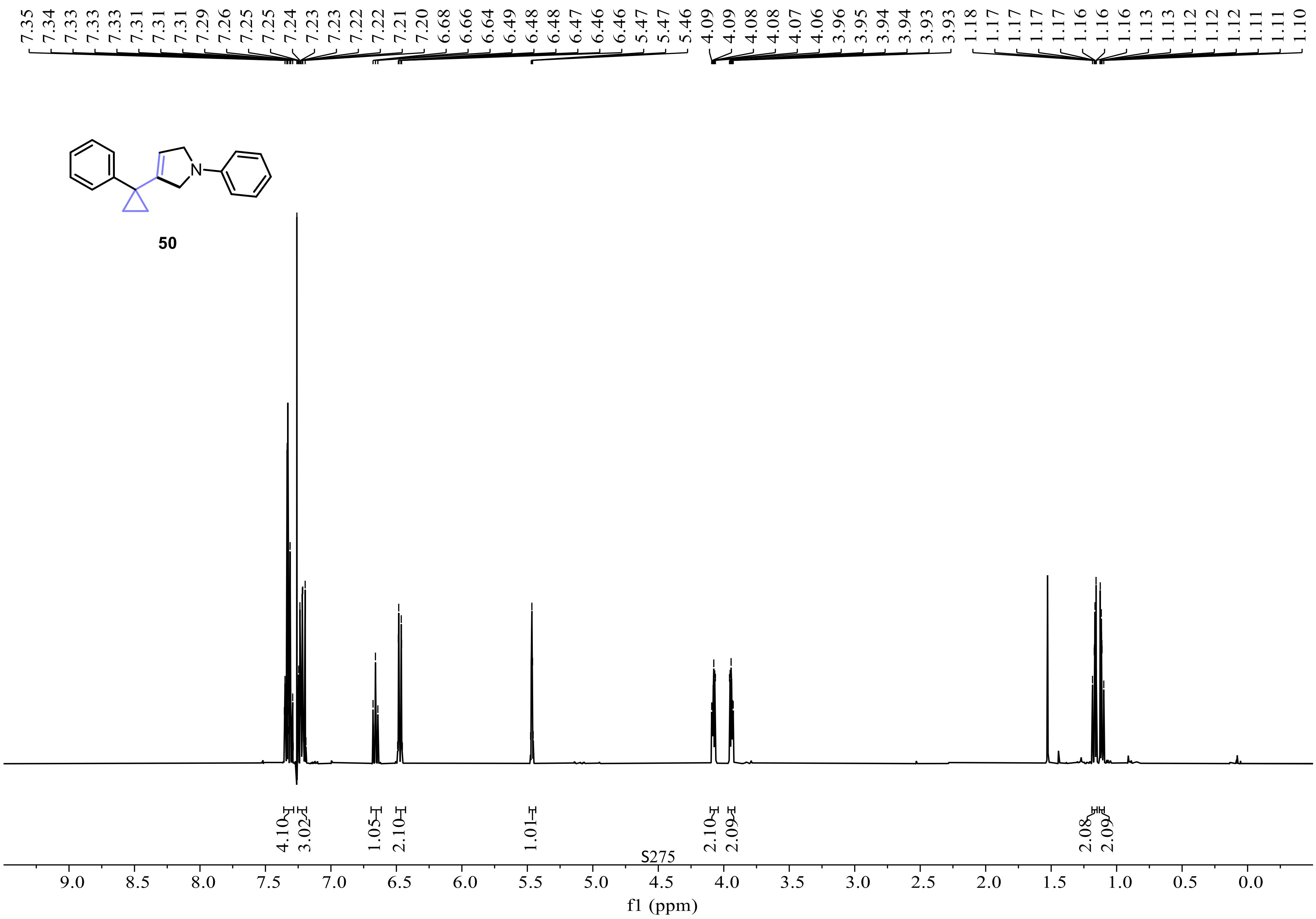
7.35
7.34
7.33
7.33
7.33
7.31
7.31
7.31
7.29
7.26
7.25
7.25
7.24
7.23
7.23
7.22
7.22
7.21
7.20
6.68
6.66
6.64
6.49
6.48
6.48
6.47
6.46
6.46
5.47
5.47
5.46
4.09
4.09
4.08
4.08
4.07
4.06
3.96
3.95
3.94
3.93
3.93
1.18
1.17
1.17
1.17
1.17
1.16
1.16
1.16
1.13
1.13
1.12
1.12
1.12
1.11
1.11
1.10
N
50
4.10
3.02
1.05
2.10
1.01
2.10
2.09
2.08
2.09
9.0
8.5
8.0
7.5
7.0
6.5
6.0
5.5
5.0
4.5
4.0
3.5
3.0
2.5
2.0
1.5
1.0
0.5
0.0
f1 (ppm)

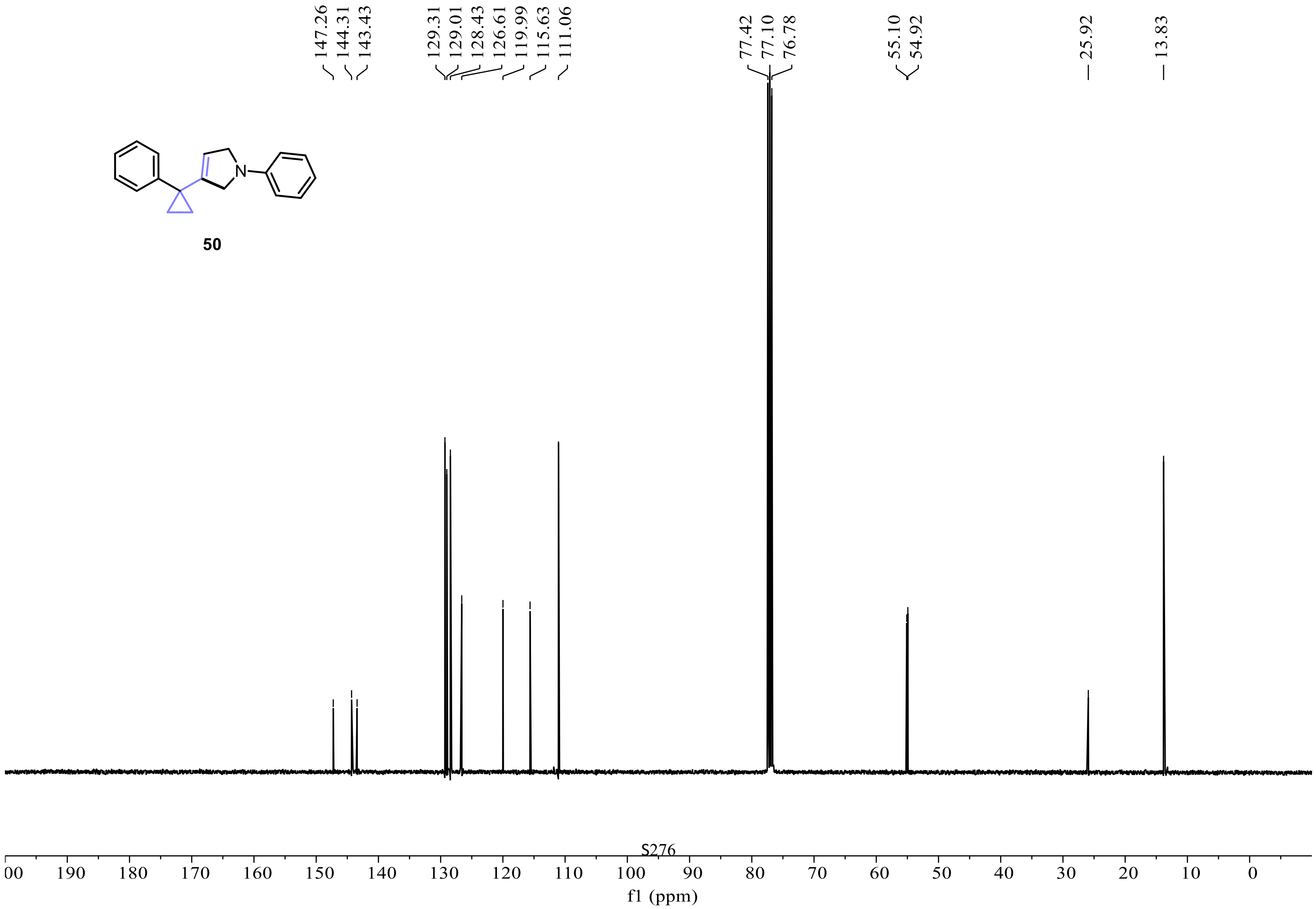
147.26
144.31
143.43
129.31
129.01
128.43
126.61
119.99
115.63
111.06
77.42
77.10
76.78
55.10
54.92
25.92
13.83
N
50
00 190 180 170 160 150 140 130 120 110 100 90 80 70 60 50 40 30 20 10 0
f1 (ppm)

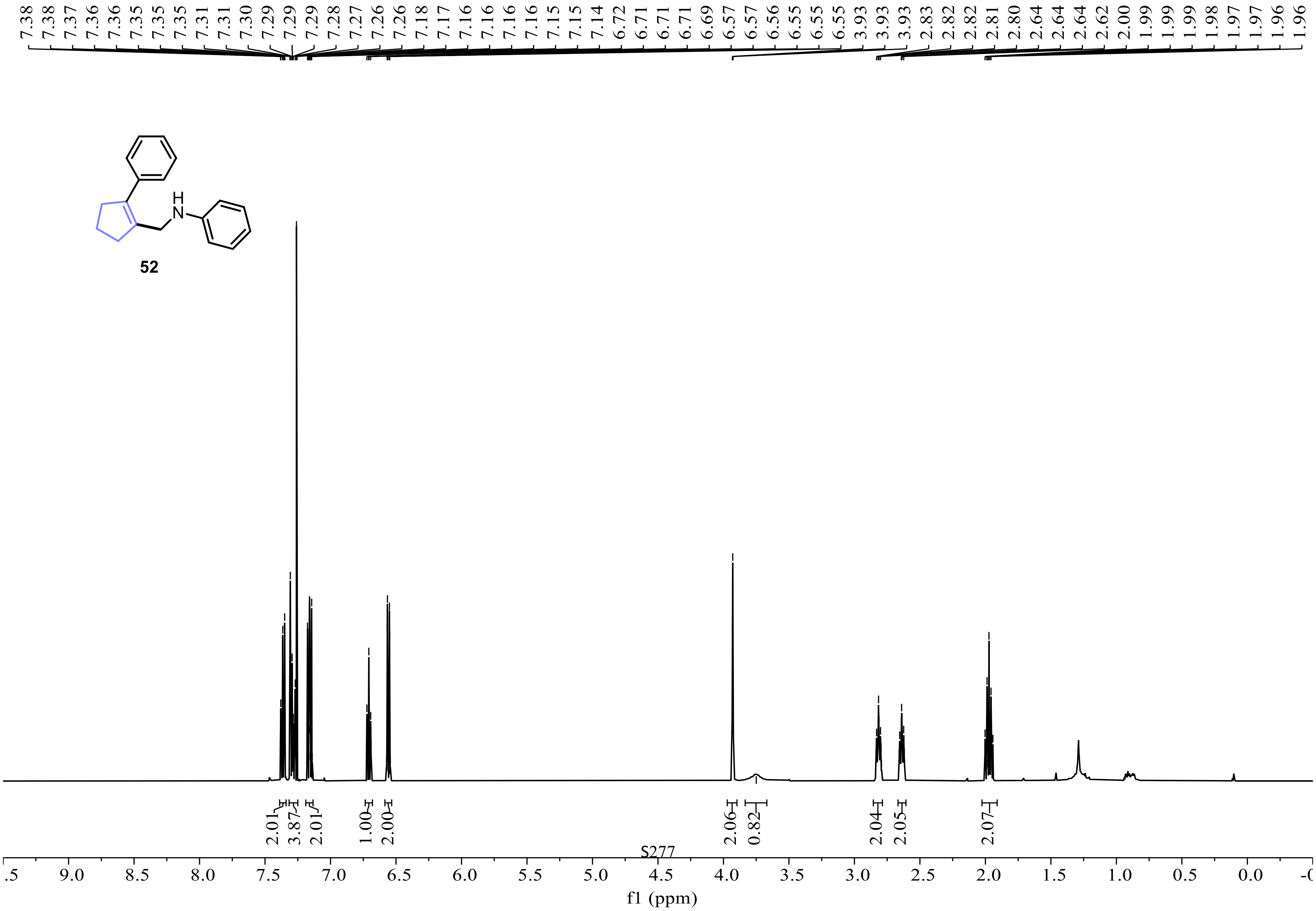
7.38 7.38 7.37 7.36 7.36 7.35 7.35 7.35 7.31 7.31 7.30 7.29 7.29 7.29 7.28 7.27 7.26 7.26 7.18 7.17 7.16 7.16 7.16 7.16 7.15 7.15 7.14 6.72 6.71 6.71 6.71 6.69 6.57 6.57 6.56 6.55 6.55 6.55 3.93 3.93 3.93 2.83 2.82 2.82 2.81 2.80 2.64 2.64 2.64 2.62 2.00 1.99 1.99 1.99 1.98 1.97 1.97 1.96 1.96
H
N
52
2.01
3.87
2.01
1.00
2.00
2.06
0.82
2.04
2.05
2.07
9.0 8.5 8.0 7.5 7.0 6.5 6.0 5.5 5.0 4.5 4.0 3.5 3.0 2.5 2.0 1.5 1.0 0.5 0.0
f1 (ppm)

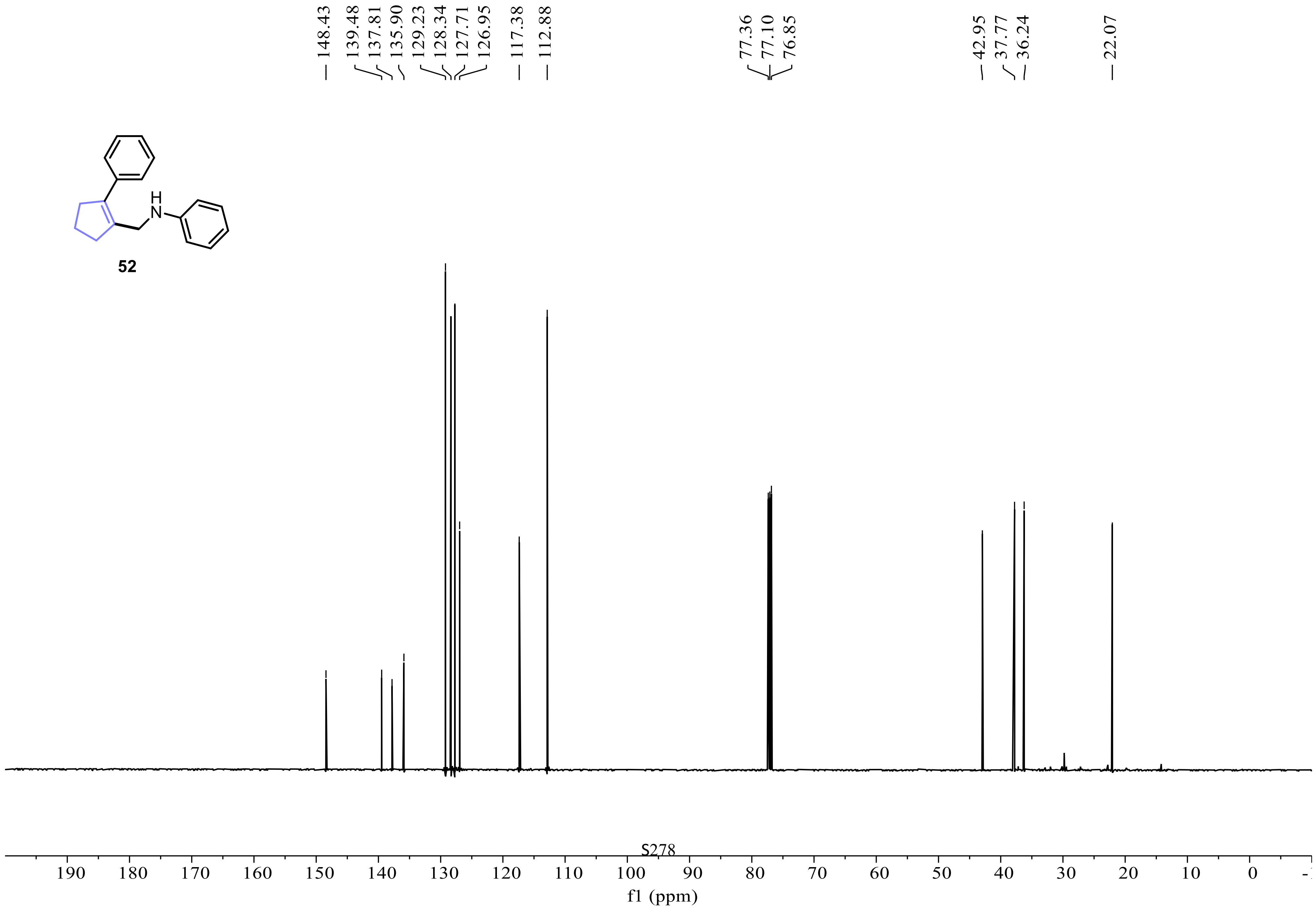
52
148.43
139.48
137.81
135.90
129.23
128.34
127.71
126.95
117.38
112.88
77.36
77.10
76.85
42.95
37.77
36.24
22.07
190 180 170 160 150 140 130 120 110 100 90 80 70 60 50 40 30 20 10 0
f1 (ppm)

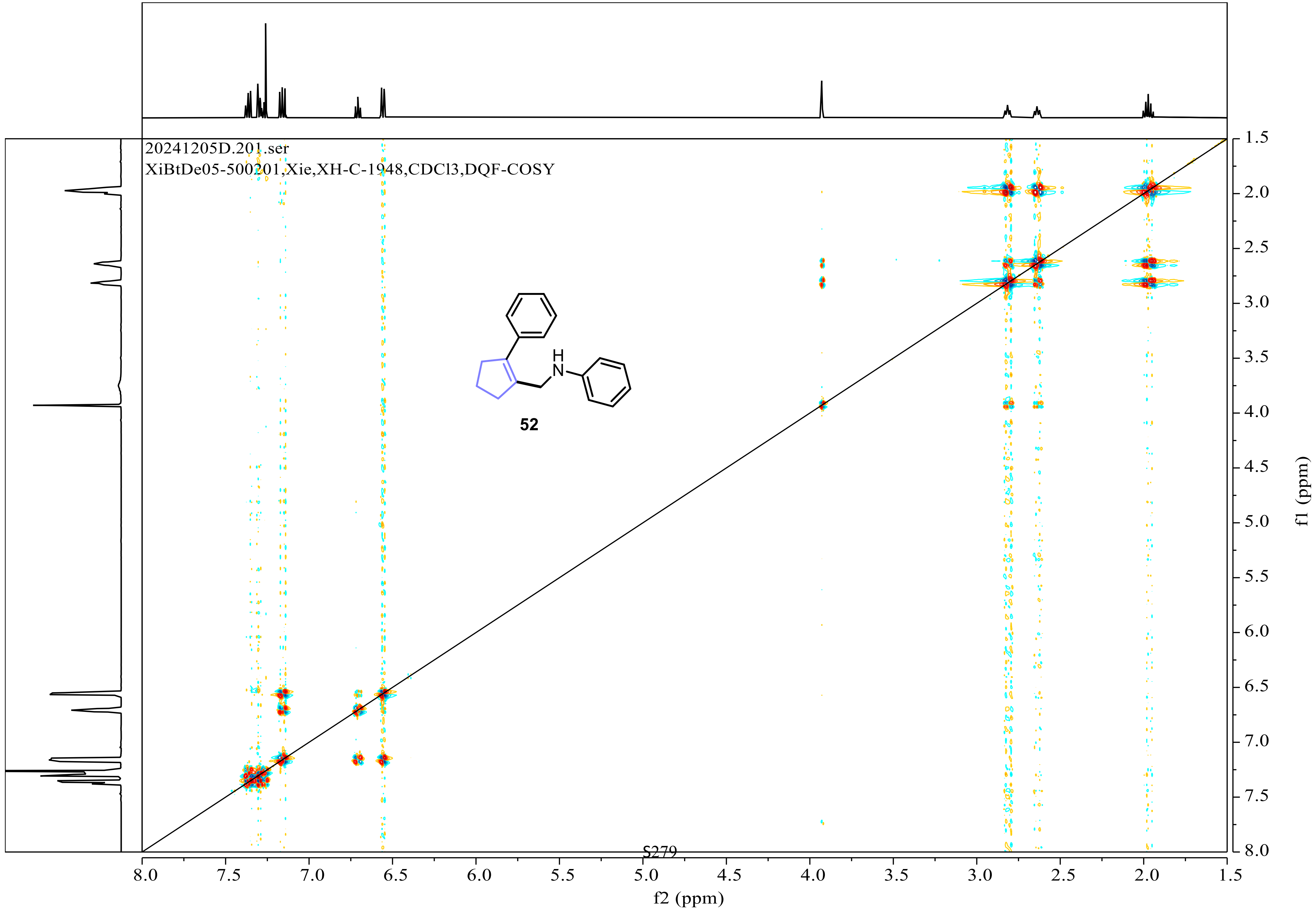
20241205D.201.ser
XiBtDe05-500201,Xie,XH-C-1948,CDCl3,DQF-COSY
H
N
52
f2 (ppm)
f1 (ppm)
8.0
7.5
7.0
6.5
6.0
5.5
5.0
4.5
4.0
3.5
3.0
2.5
2.0
1.5

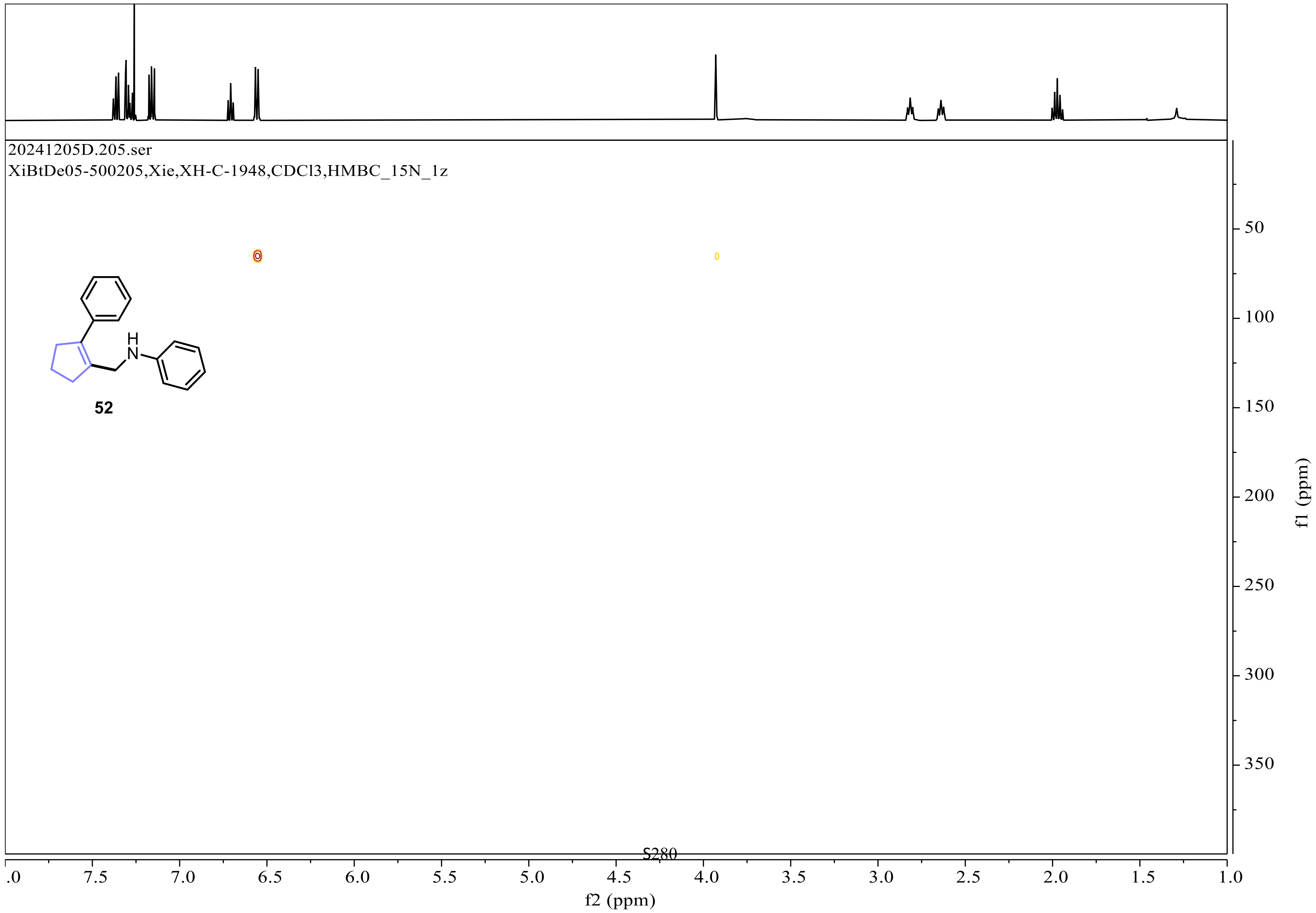
20241205D.205.ser
XiBtDe05-500205,Xie,XH-C-1948,CDCl3,HMBC_15N_1z
52
f2 (ppm)
f1 (ppm)

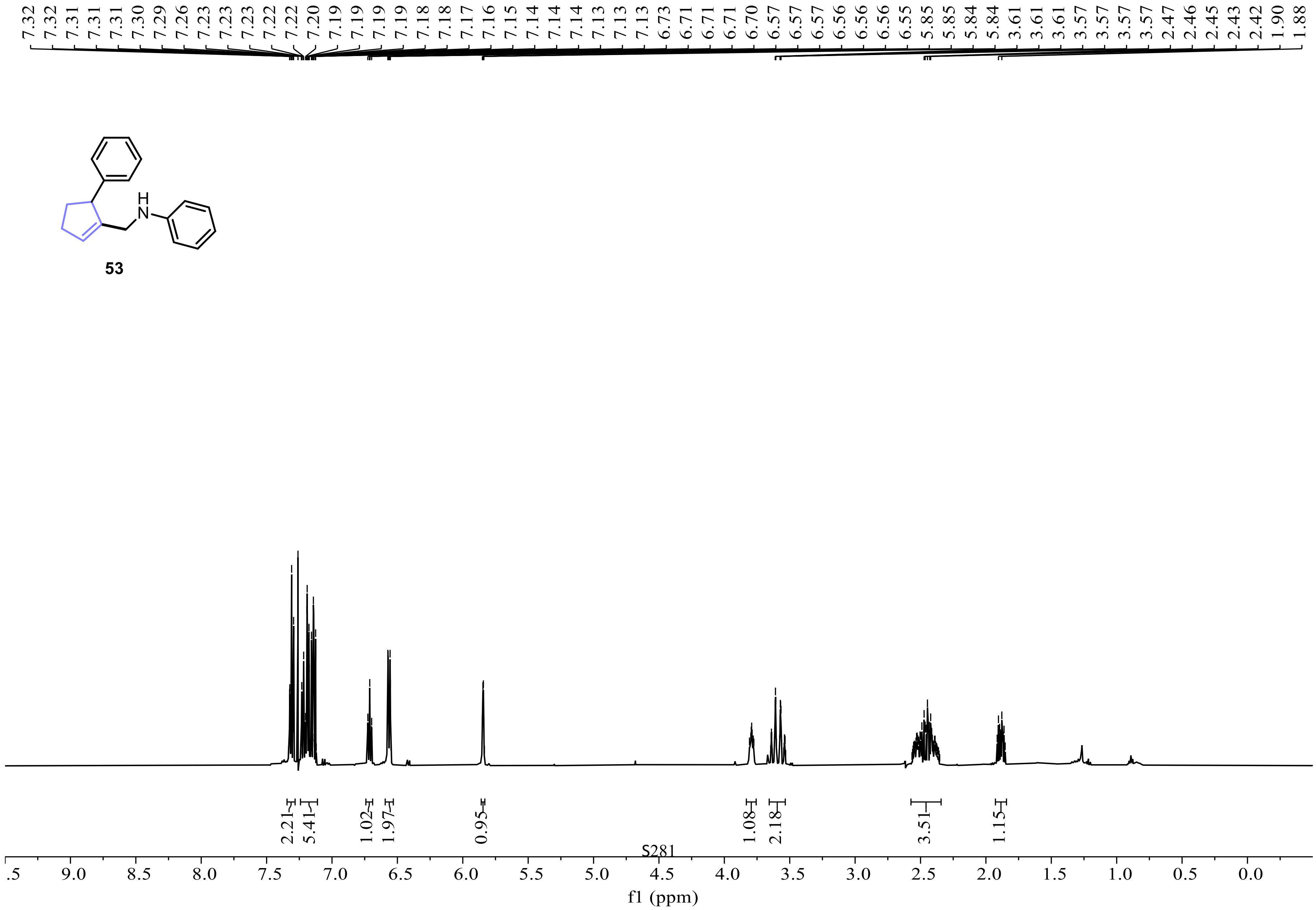
7.32 7.32 7.31 7.31 7.31 7.30 7.29 7.26 7.23 7.23 7.23 7.22 7.22 7.20 7.19 7.19 7.19 7.19 7.18 7.18 7.17 7.16 7.15 7.14 7.14 7.14 7.13 7.13 7.13 6.73 6.71 6.71 6.71 6.70 6.57 6.57 6.57 6.56 6.56 6.56 6.55 5.85 5.85 5.84 5.84 3.61 3.61 3.61 3.57 3.57 3.57 3.57 2.47 2.46 2.45 2.43 2.42 1.90 1.88
H
N
53
2.21
5.41
1.02
1.97
0.95
1.08
2.18
3.51
1.15
.5 9.0 8.5 8.0 7.5 7.0 6.5 6.0 5.5 5.0 4.5 4.0 3.5 3.0 2.5 2.0 1.5 1.0 0.5 0.0
f1 (ppm)

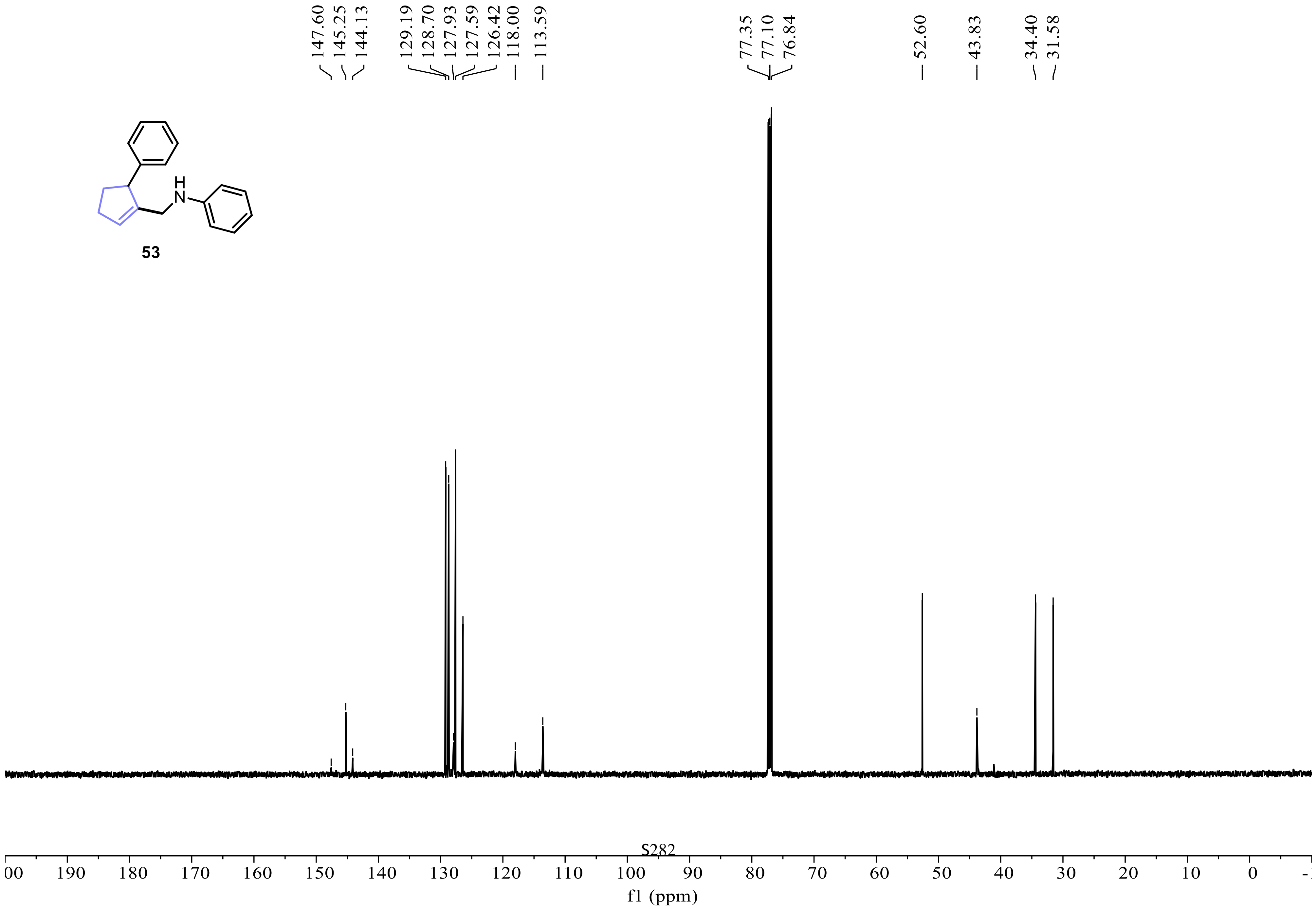
147.60
145.25
144.13
129.19
128.70
127.93
127.59
126.42
118.00
113.59
77.35
77.10
76.84
52.60
43.83
34.40
31.58
H
N
53
00 190 180 170 160 150 140 130 120 110 100 90 80 70 60 50 40 30 20 10 0
f1 (ppm)

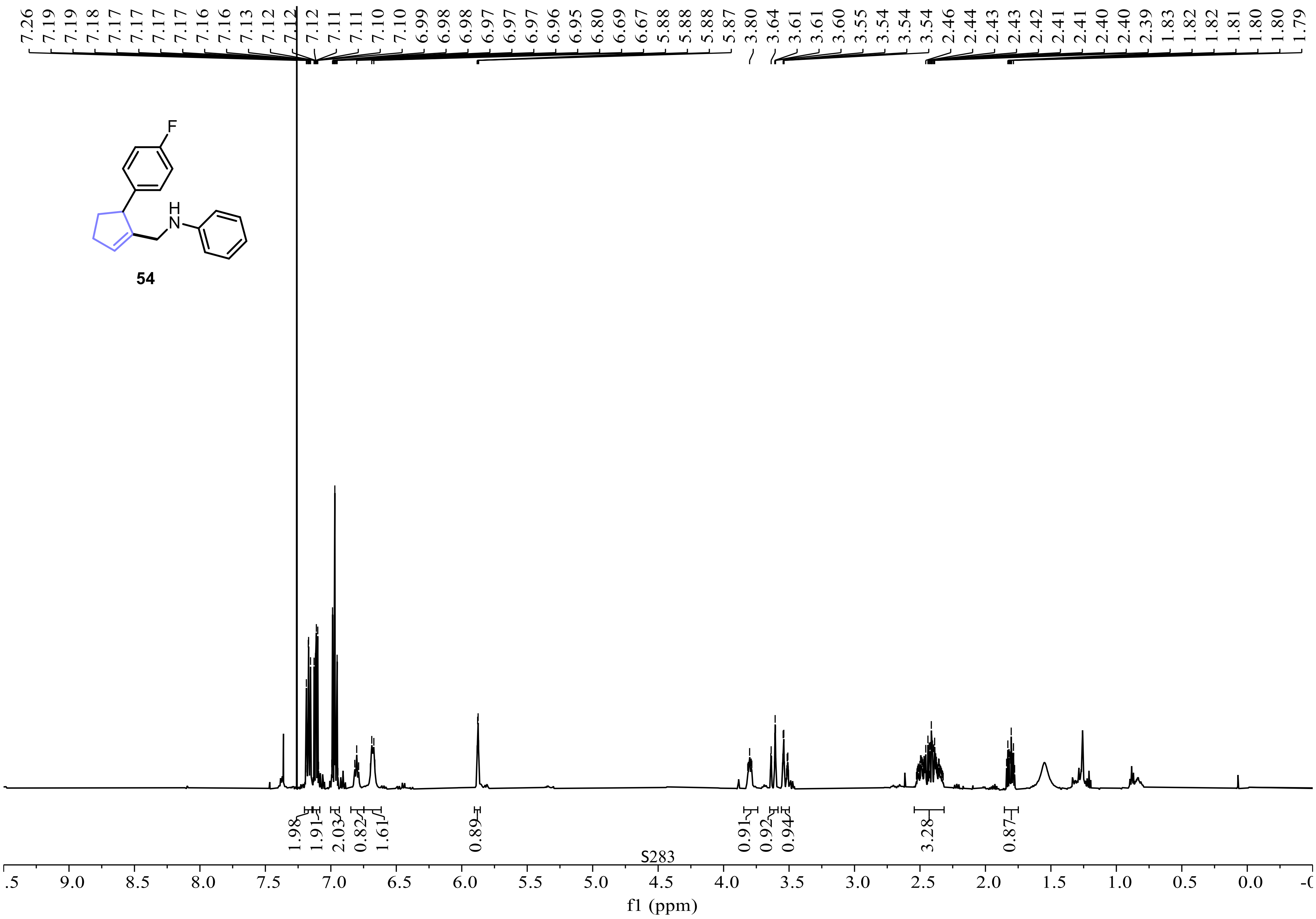
7.26 7.19 7.19 7.18 7.17 7.17 7.17 7.17 7.16 7.16 7.13 7.12 7.12 7.12 7.11 7.11 7.10 7.10 6.99 6.98 6.98 6.97 6.97 6.97 6.96 6.95 6.80 6.69 6.67 5.88 5.88 5.88 5.87 3.80 3.64 3.61 3.61 3.60 3.55 3.54 3.54 3.54 2.46 2.44 2.43 2.43 2.42 2.41 2.41 2.40 2.40 2.39 1.83 1.82 1.82 1.81 1.80 1.80 1.79
F
H
N
54
1.98 1.91 2.03 0.82 1.61 0.89 0.91 0.92 0.94 3.28 0.87
.5 9.0 8.5 8.0 7.5 7.0 6.5 6.0 5.5 5.0 4.5 4.0 3.5 3.0 2.5 2.0 1.5 1.0 0.5 0.0
f1 (ppm)

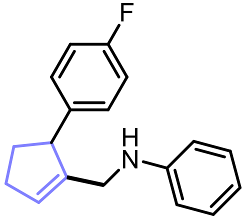

54

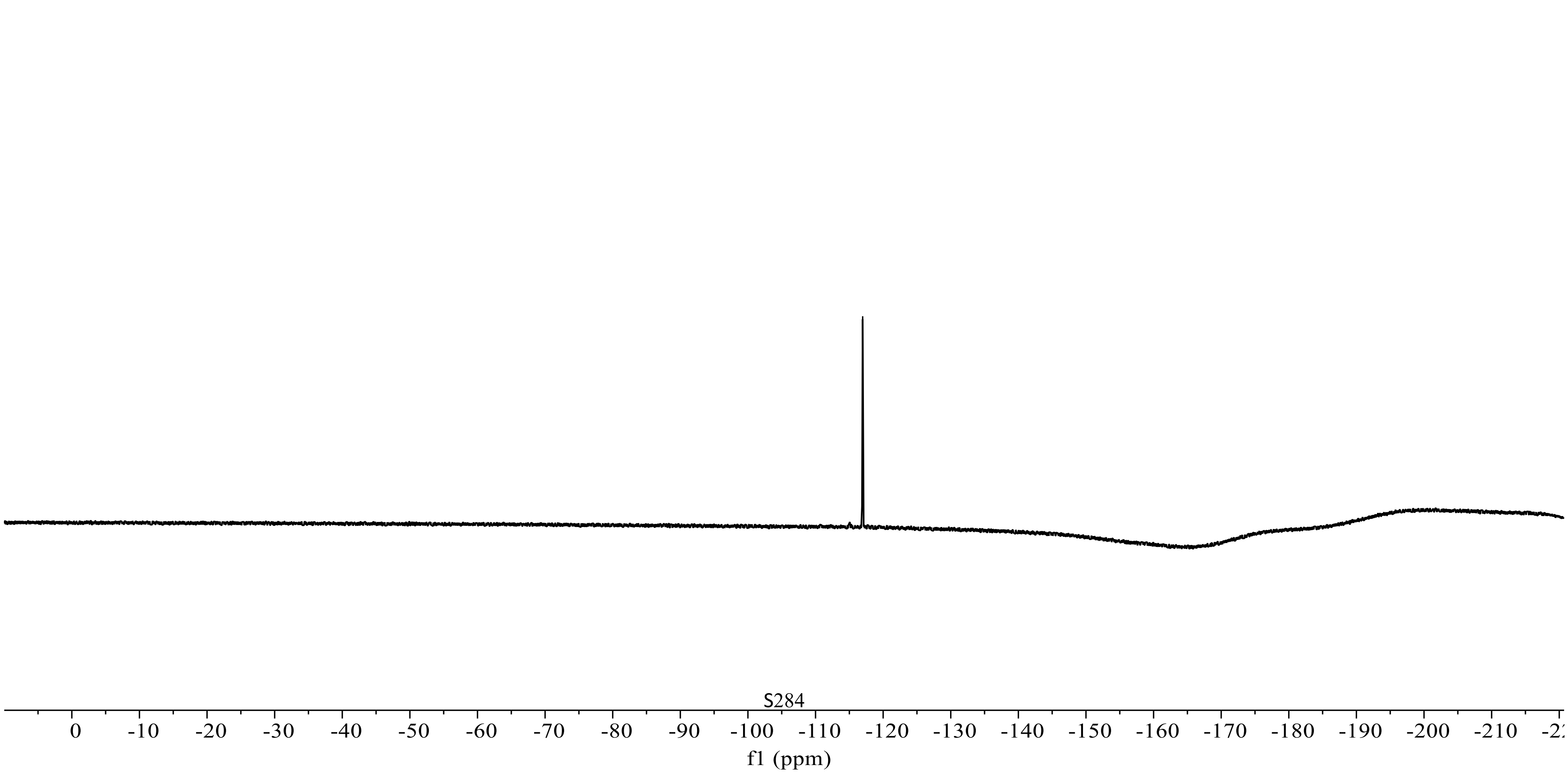
-116.97
0
-10
-20
-30
-40
-50
-60
-70
-80
-90
-100
-110
-120
-130
-140
-150
-160
-170
-180
-190
-200
-210
f1 (ppm)

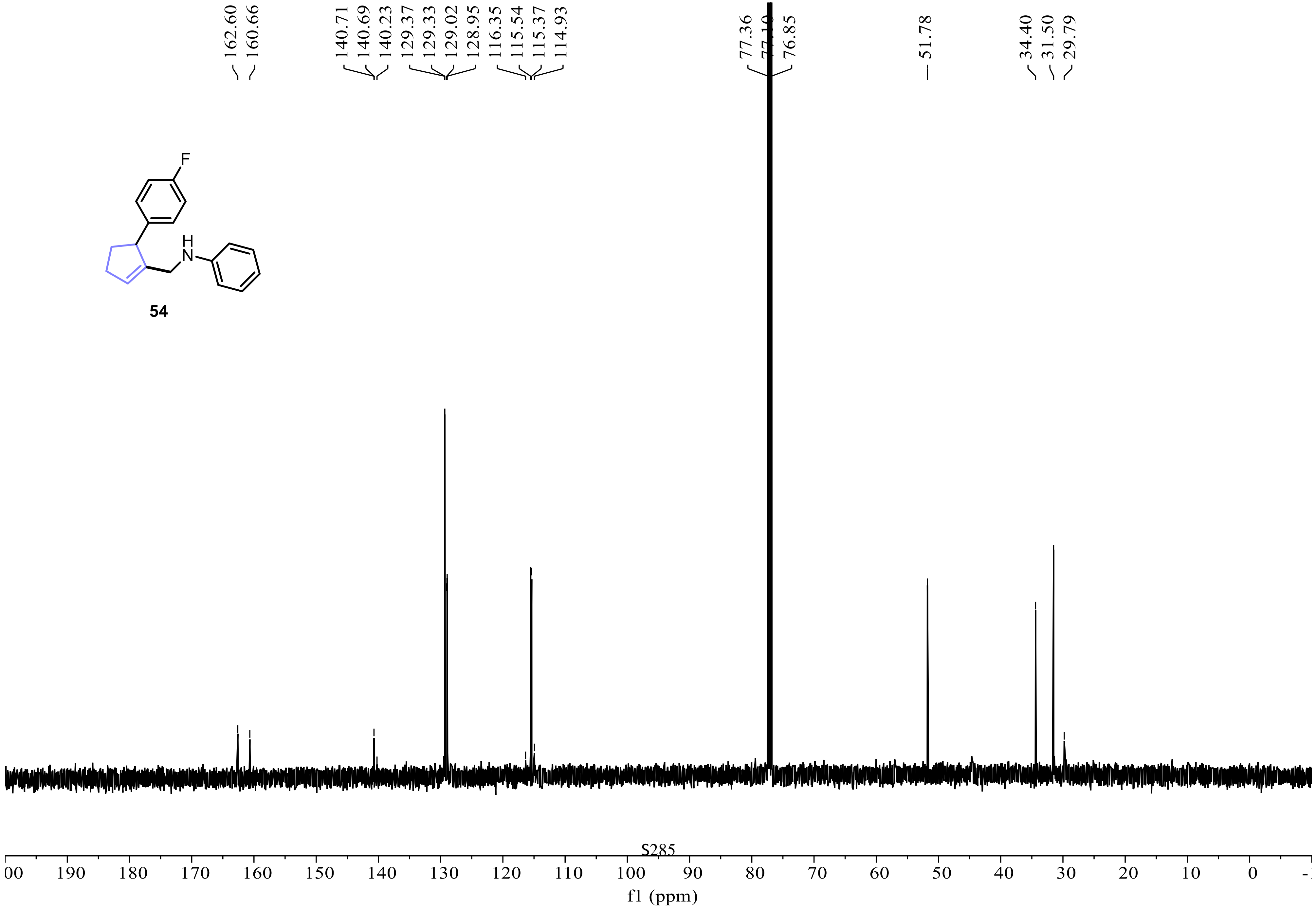
162.60
160.66
140.71
140.69
140.23
129.37
129.33
129.02
128.95
116.35
115.54
115.37
114.93
77.36
76.85
51.78
34.40
31.50
29.79
F
H
N
54
00 190 180 170 160 150 140 130 120 110 100 90 80 70 60 50 40 30 20 10 0
f1 (ppm)

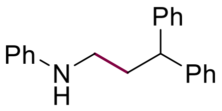
Ph
N
H
Ph
Ph

56

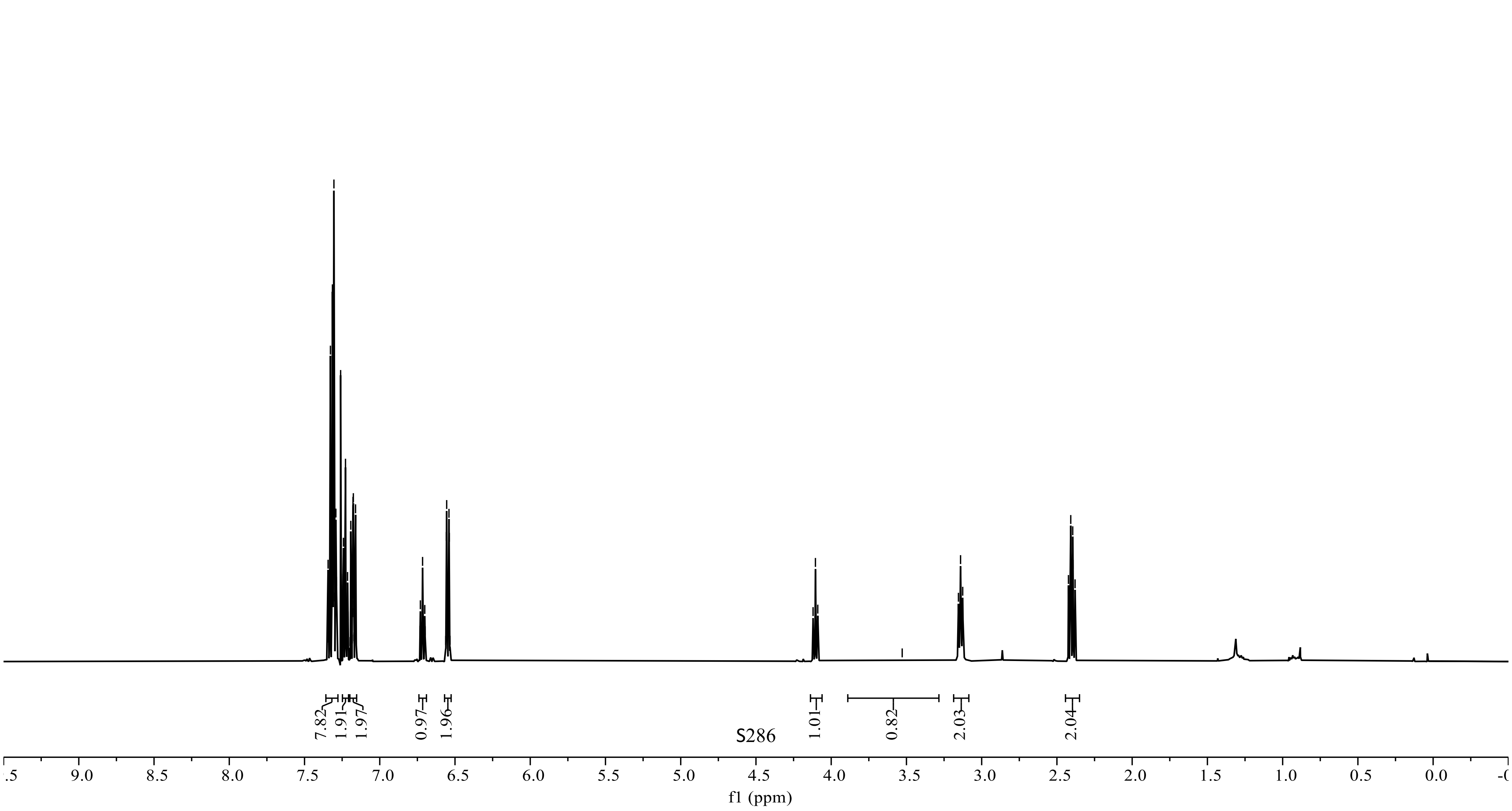
7.35 7.35 7.34 7.34 7.33 7.33 7.33 7.32 7.31 7.31 7.31 7.30 7.30 7.29 7.29 7.29 7.26 7.25 7.24 7.24 7.23 7.23 7.23 7.23 7.22 7.22 7.21 7.21 7.19 7.19 7.19 7.18 7.18 7.18 7.18 7.17 7.17 7.16 7.16 7.16 7.16 6.73 6.73 6.73 6.72 6.72 6.71 6.70 6.70 6.70 6.56 6.56 6.56 6.55 6.55 6.54 6.54 6.54 6.54 6.54 6.53 4.12 4.10 4.09 3.15 3.14 3.13 2.42 2.41 2.41 2.39 2.39 2.38
7.82
1.91
1.97
0.97
1.96
1.01
0.82
2.03
2.04
.5 9.0 8.5 8.0 7.5 7.0 6.5 6.0 5.5 5.0 4.5 4.0 3.5 3.0 2.5 2.0 1.5 1.0 0.5 0.0 -(
f1 (ppm)

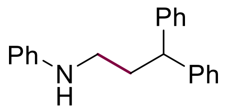

56

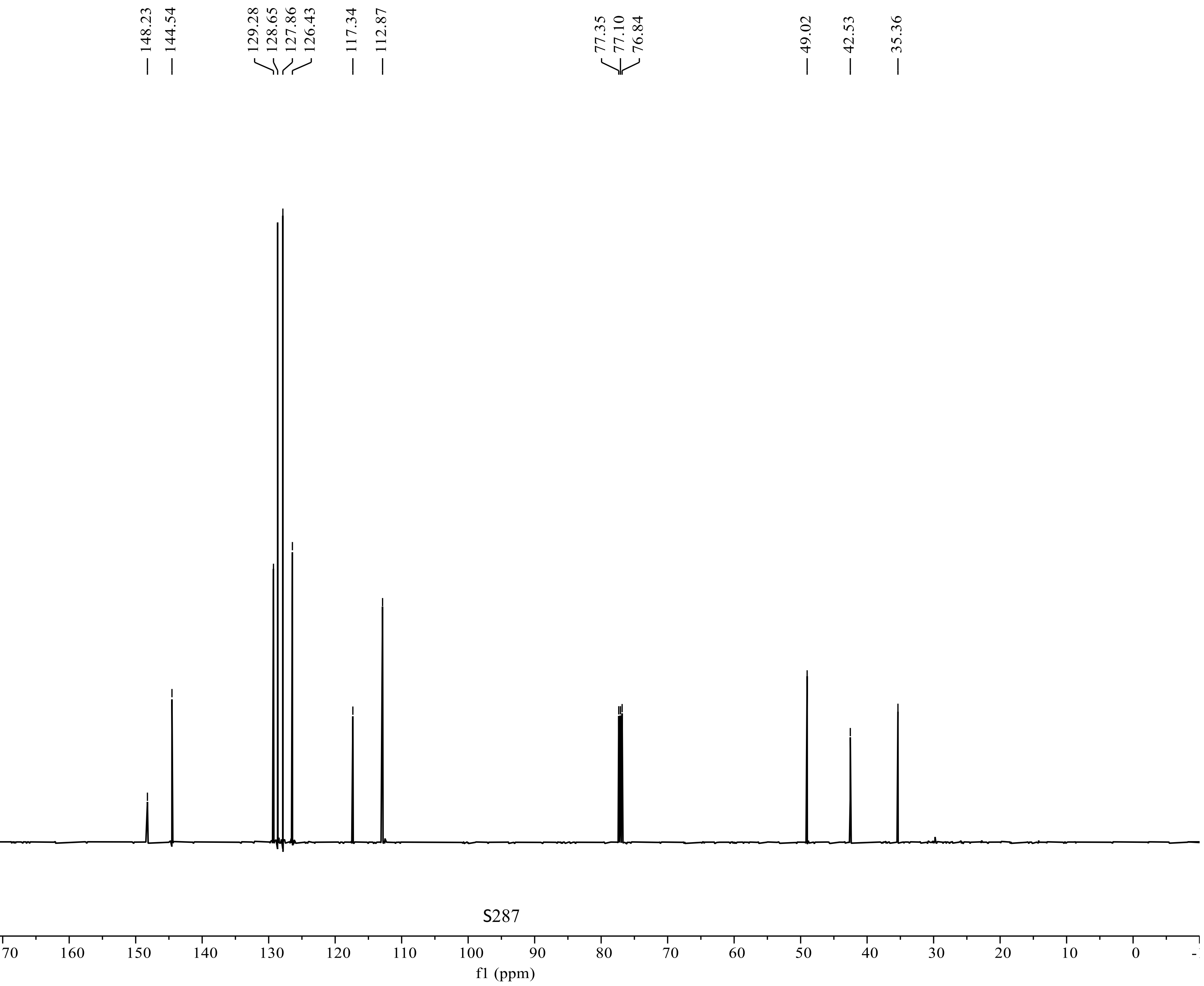

148.23
144.54
129.28
128.65
127.86
126.43
117.34
112.87
77.35
77.10
76.84
49.02
42.53
35.36
f1 (ppm)

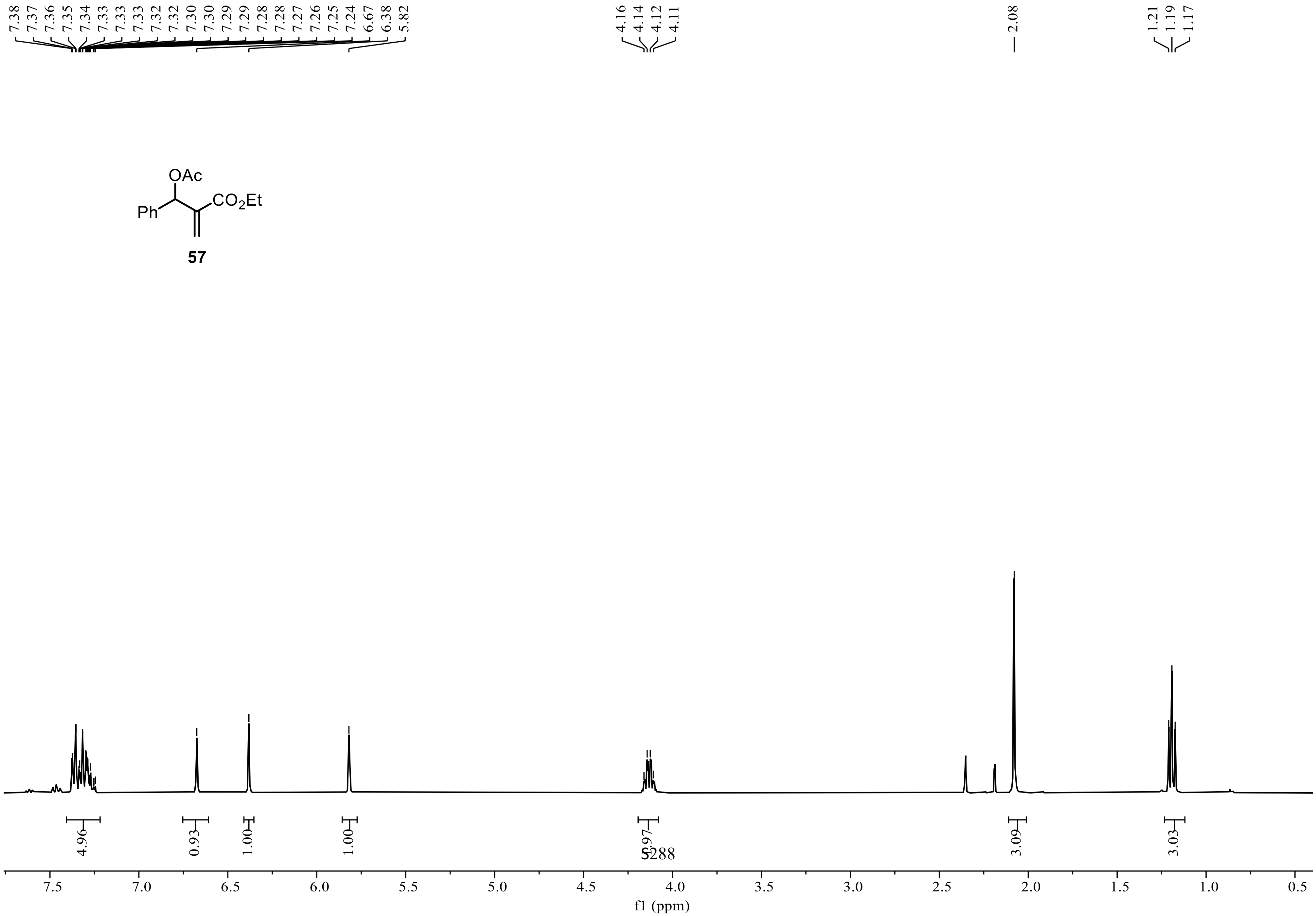
7.38 7.37 7.36 7.35 7.34 7.33 7.33 7.33 7.32 7.32 7.30 7.30 7.29 7.29 7.28 7.28 7.27 7.26 7.25 7.24 6.67 6.38 5.82
4.16 4.14 4.12 4.11
2.08
1.21 1.19 1.17
OAc
Ph
CO_2Et
57
4.96
0.93
1.00
1.00
1.97
3.09
3.03
7.5 7.0 6.5 6.0 5.5 5.0 4.5 4.0 3.5 3.0 2.5 2.0 1.5 1.0 0.5
f1 (ppm)

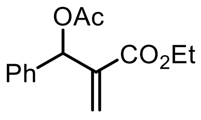
OAc
Ph
CO2Et

57

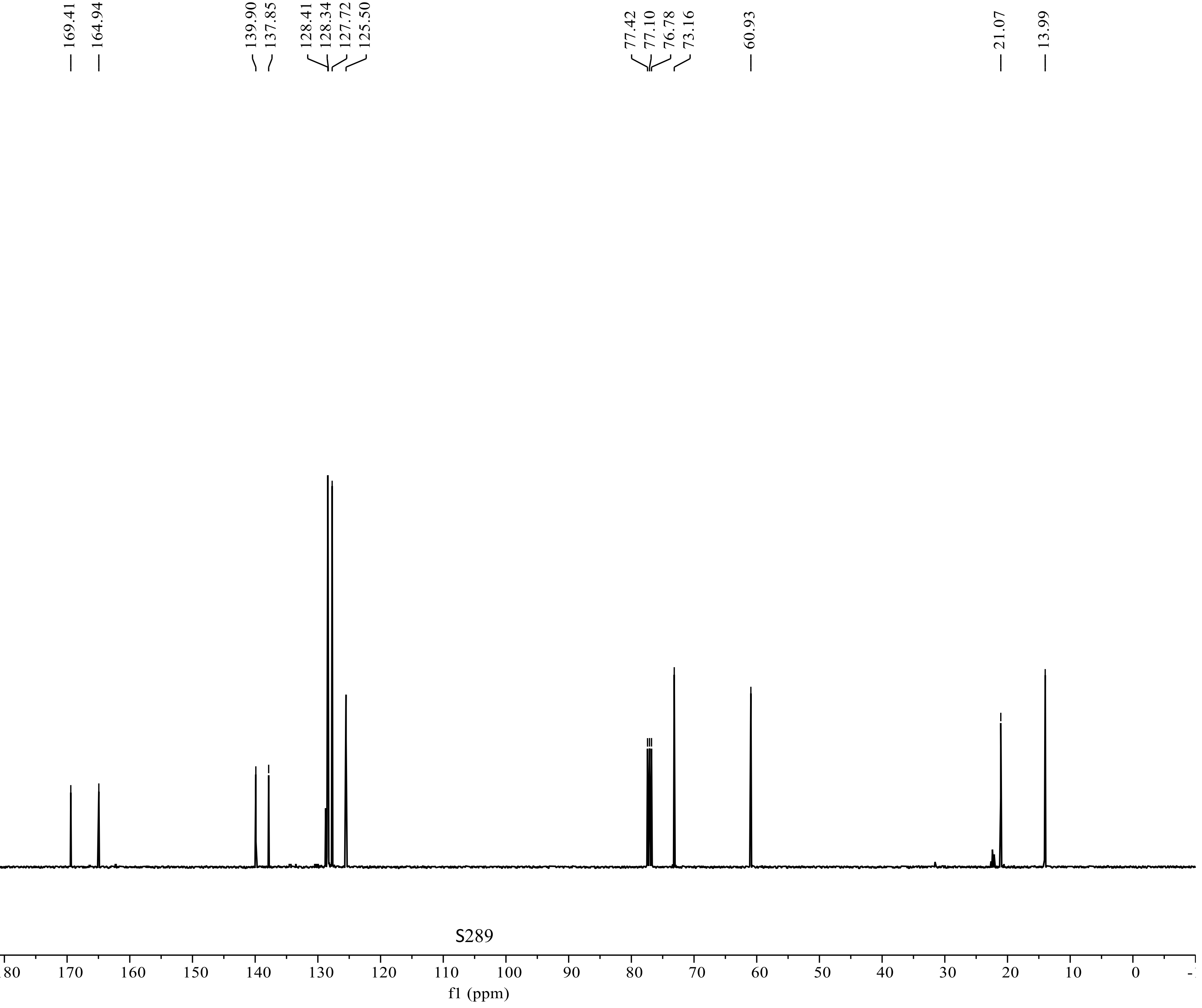
169.41
164.94
139.90
137.85
128.41
128.34
127.72
125.50
77.42
77.10
76.78
73.16
60.93
21.07
13.99
210
200
190
180
170
160
150
140
130
120
110
100
90
80
70
60
50
40
30
20
10
0
f1 (ppm)

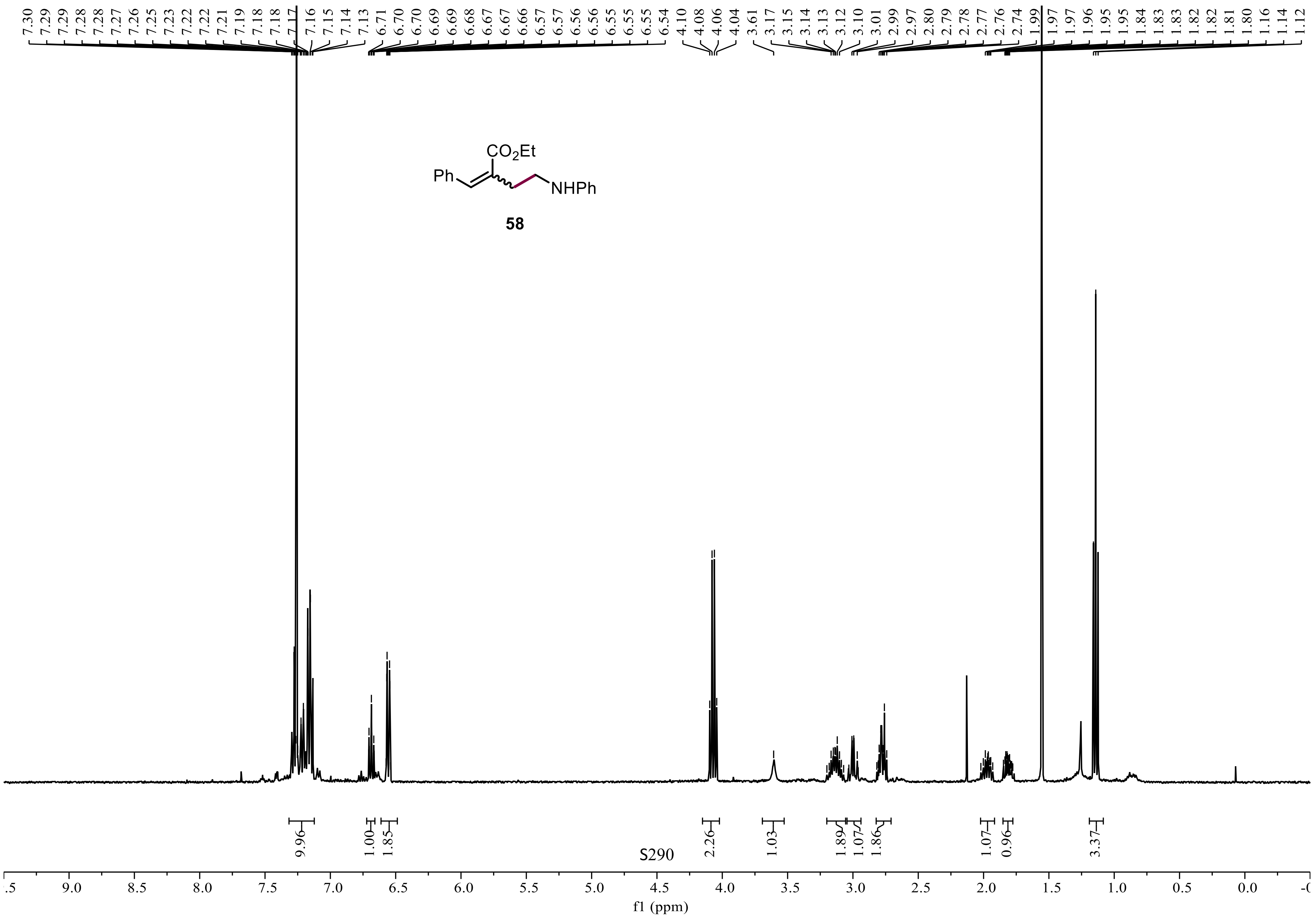
CO2Et
Ph
NHPh
58
f1 (ppm)

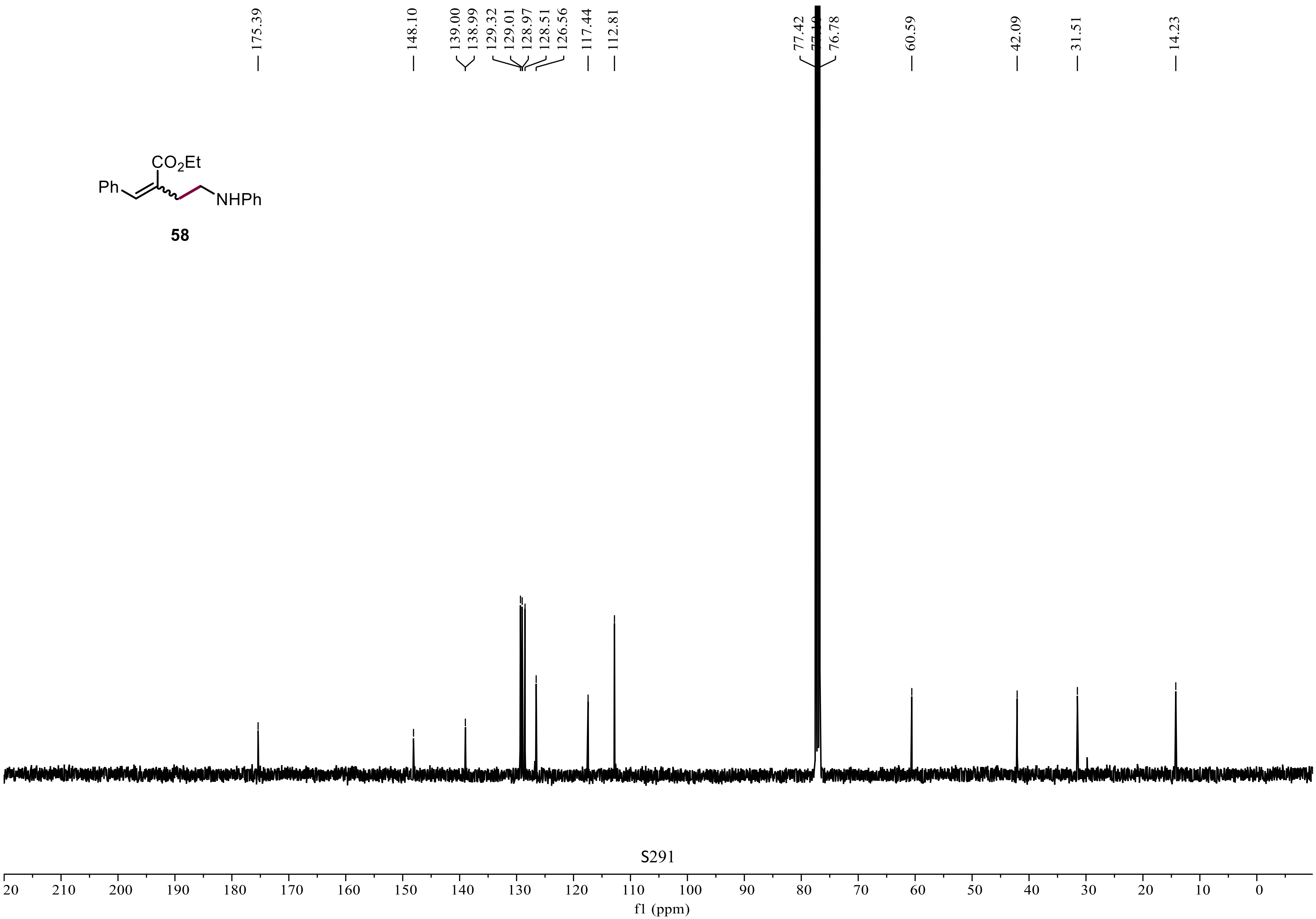
175.39
148.10
139.00
138.99
129.32
129.01
128.97
128.51
126.56
117.44
112.81
77.42
76.78
60.59
42.09
31.51
14.23
CO_2Et
Ph
NHPh
58
20
210
200
190
180
170
160
150
140
130
120
110
100
90
80
70
60
50
40
30
20
10
0
f1 (ppm)
